# Non-enzymatic methylcyclization of alkenes

**DOI:** 10.1038/s41557-025-01774-3

**Published:** 2025-03-07

**Authors:** Immanuel Plangger, Elias Schmidhammer, Sebastian Schaar, Klaus Wurst, Maren Podewitz, Thomas Magauer

**Affiliations:** 1 Department of Organic Chemistry and Center for Molecular Biosciences, University of Innsbruck, Innsbruck, Austria; 2 Department of General, Inorganic and Theoretical Chemistry, University of Innsbruck, Innsbruck, Austria; 3 Department of Materials Chemistry, TU Wien, Vienna, Austria

## Abstract

Methyltransferases are a broad class of enzymes that catalyse the transfer of methyl groups onto a wide variety of substrates and functionalities. In their most striking variant, bifunctional methyltransferase–cyclases both transfer a methyl group onto alkenes and induce cyclization (methylcyclization). Although recent years have seen substantial advances in the methylation of alkenes, especially hydromethylation, the reactivity demonstrated by bifunctional methyltransferase–cyclases in nature has yet to be developed into a synthetically viable method. Here we report a silver(I)-mediated electrophilic methylcyclization that rivals selectivities found in enzymes while not being limited by their inherent substrate specificity. Our method benefits from the use of commercial reagents, is applicable to a wide range of substrates, including heterocycles, and affords unique structures that are difficult to access via conventional synthetic methods. Furthermore, computational studies have been utilized to unravel the underlying mechanism and ultimately support a stepwise cationic reaction pathway with a rate-limiting methyltransfer.

In nature methyltransferases (MT) play an integral role in cell signalling, gene regulation and the biosynthesis of secondary metabolites^1–14^. For the latter, the selective methylation of alkenes by *S*-adenosyl methionine (SAM)-dependent *C*-methyltransferases provides access to new chemical space and allows escape from the canonical C_5_-isoprene pattern of terpenoid carbon scaffolds. In most cases, *C*-methyltransferases catalyse the methylation of simple linear building blocks, such as isopentenyl pyrophosphate and geranyl pyrophosphate^6–8,11,12,15,16^. Sterol methyltransferases introduce methyl groups onto linear alkenes that are linked to a polycyclic backbone^9,10^. Bifunctional methyltransferase–cyclases (bMTC) stand out due to their ability to induce cyclization by an initial methyltransfer (methylcyclization), which allows for the rapid build-up of molecular complexity. So far, only two bMTCs have been identified: an MT in the sodorifen biosynthesis found in *Serratia plymuthica* and TleD in the teleocidin biosynthesis^13,14,17^. TleD catalyses the SAM-dependent conversion of lyngbyatoxin A to teleocidin B-4 (Fig. 1a). Another methyltransferase–cyclase was proposed by Jaenicke and co-workers in 1988 for the biosynthesis of the iridals, which release irones through oxidative degradation^18^. These valuable degradation products a re found in exclusive fragrances and are the subject of numerous patents and publications^19–25^. While the responsible methyltransferase still remains elusive, the groups of Chen and André recently engineered TleD to efficiently convert psi-ionone to *cis*-α-irone in a process similar to the proposed biosynthesis (Fig. 1b)^26^. Although there have been substantial advances in the C methylation of alkenes^27–39^, especially hydromethylation, non-enzymatic methyltransfer-initiated cyclization reactions have remained elusive and, until now, access to methylated, non-canonical terpenoids has required lengthy chemical syntheses or knowledge of a suitable methyltransferase.cyclase. To the best of our knowledge, there are only two reports describing transformations akin to those catalysed by *C*-methyltransferases. In 1984, Barradas and co-workers disclosed an electrochemical protocol employing sodium acetate as the methyl donor to convert oct-1-ene to a complex mixture of mono- and dimethylated products^39^. Notably, cyclohexene, an alkene with a lower oxidation potential, was not compatible. Shortly thereafter, Julia and Marazano disclosed conditions employing diaryl methyl sulfonium salts at high temperatures (135–170 °C) to convert 2-methyloct-2-ene to a mixture of eight mono- and dimethylation products36. Further investigations of heteroaromatic sulfonium salts by the groups of Julia and Shiraishi failed to improve the low efficiency and poor selectivity^37,38^. The reported methods are limited to pure hydrocarbons void of any functional group other than a single alkene. This is in stark contrast to *C*-methyltransferases in nature, which operate undisturbed in the presence of various nucleophilic functionalities, such as heteroatoms. To fill this longstanding gap in the synthetic toolbox, we envisioned the development of an electrophilic methylation system, which exhibits high chemoselectivity towards alkenes over arenes and other nucleophiles—a challenging endeavour owing to the comparably low nucleophilicity of alkenes (Fig. 1c). Carbocations generated in this way would be primed for a subsequent nucleophilic attack (cyclization) by a nearby C(*sp*^2^) system. Herein, we report the development of a non-enzymatic methyltransfer–cyclization (NEMTC) reaction, which proceeds not only at ambient temperatures with commercially available reagents, but also exhibits a wide functional group tolerance, including the presence of oxygen-, nitrogen- and sulfur-based functionalities. Products of this bioinspired transformation feature unique, semisaturated structural motifs with two new C–C bonds along with a quaternary carbon centre, which would otherwise require step-intensive preparations. This powerful methodology enables access to a wide area of unexplored chemical space (methyl-substituted polycycles, including bi-, tri-, tetra- and spirocycles) through variation of the initiating group (for example, substitution degree of the alkene), the terminating groups ((hetero)arenes, alkenes) and the linker chain (for example, monoversus bicyclizations).

## Results and discussion

**Fig. 1:**
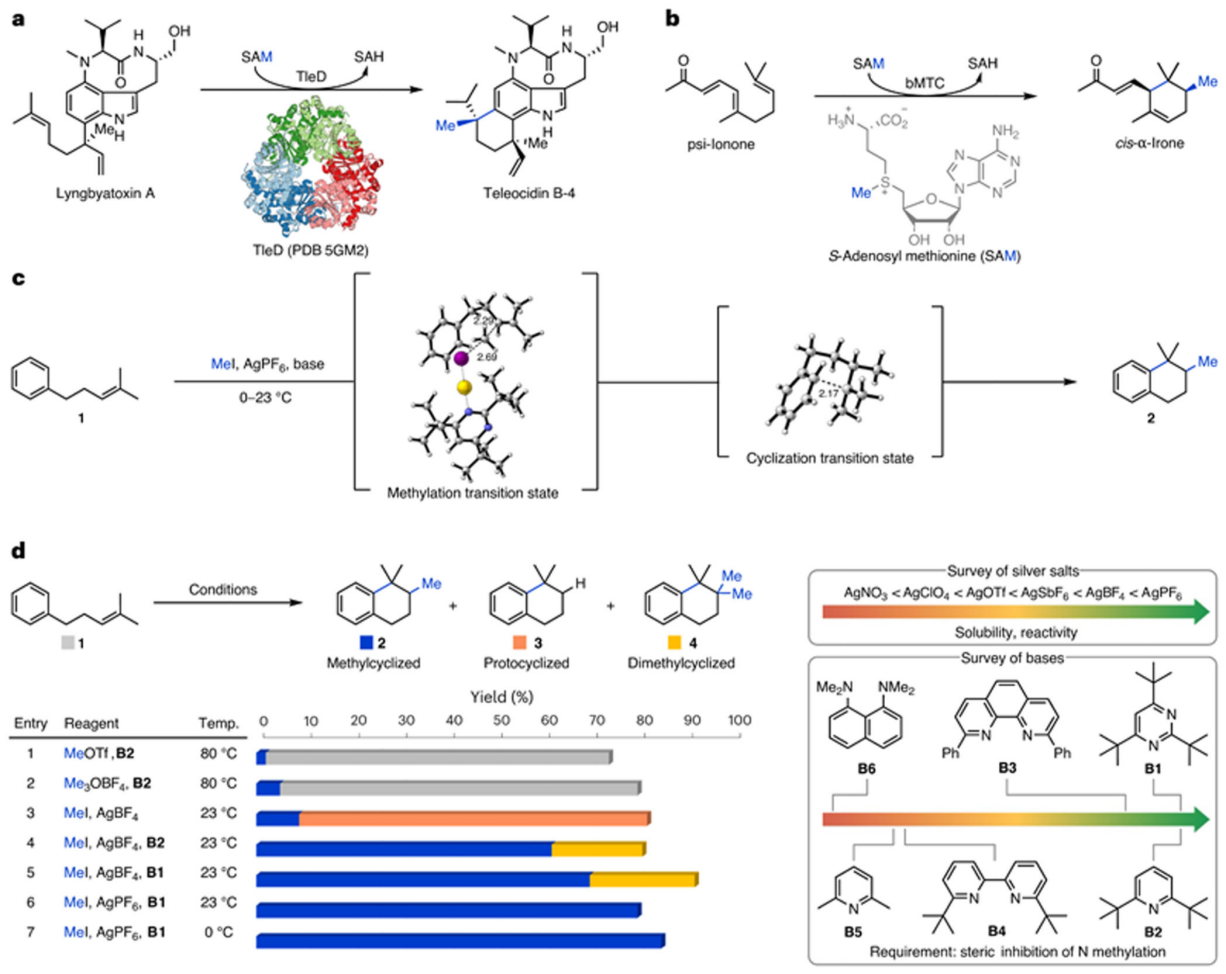
Methylcyclization of alkenes. **a**, The natural bifunctional methyltransferase–cyclase TleD transforms lyngbyatoxin A to teleocidin B-4. The natural methyl donor SAM is converted to *S*-adenosyl homocysteine (SAH) upon losing a methyl group^13,17^. **b**, An engineered bifunctional methyltransferase–cyclase converts psiionone to *cis*-α-irone, the major olfactory component of Irone Alpha^26^. **c**, This work discloses a non-enzymatic methyltransfer–cyclization reaction that uses silver hexafluorophosphate and methyl iodide instead of enzymes and SAM. Numbers in transition states indicate selected distances in angstrom. **d**, Development of the NEMTC reaction. NMR yields are given. Choice of the silver salt and base are pivotal for product selectivity and reaction efficiency (see Supplementary Sections 2.1.2 and 2.1.3). Temp., temperature.

For reaction development we subjected alkene **1** to a variety of classical electrophilic methylating reagents (Fig. 1d). While reagents reminiscent of SAM, namely trimethylsulfonium iodide (Me_3_SI) and its sulfur(VI) analogue trimethylsulfoxonium iodide (Me_3_S(O)I), remained unsuccessful, the use of methyl triflate (MeOTf) and trimethyloxonium tetrafluoroborate (Me_3_OBF_4_, Meerwein’s salt) was encouraging as, for the first time, traces of methylcyclized product **2** (2–5%) were obtained (Fig. 1d, entries 1 and 2). In an effort to improve the yield of **2**, we turned our attention to the seminal work of Meerwein, which describes the synthesis of trialkyloxonium tetrafluoroborates from dialkylethers, alkyl halides and silver(I) tetrafluoroborate^40,41^. Subjection of alkene **1** to a combination of MeI and AgBF_4_ increased the yield of methylcyclized product **2** to 9% together with protocyclized product **3** (73%) (Fig. 1d, entry 3). Introduction of 2,6-di-*tert*-butyl-pyridine (**B2**), an exceptionally hindered base resistant to methylation^42^, prevented the competing protocyclization and improved the yield for the desired product **2** to 62% (Fig. 1d, entry 4). Unexpectedly, dimethylcyclized product **4** (19%) was also formed under these conditions. We speculate that **4** originates from a premature deprotonation allowing for a second methylation and subsequent cyclization. To probe the influence of the base on premature deprotonation, **B2** was exchanged for 2,4,6-tri -*tert*-butyl-pyrimidine (**B1**) (Fig. 1d, entry 5). Although the exchange from **B1** to **B2** had no substantial influence on the formation of the dimethylcyclized product **4** (22%), we were pleased to obtain **2** in a modestly increased yield of 70%. Other bases, such as **B3, B4, B5** and **B6** suffered from low conversions due to competitive N methylation. Finally, we investigated the role of the silver(I) counterion on the formation of undesired product **4**. From this screen, silver(I) hexafluorophosphate emerged as ideal, affording methylcyclized product **2** in 80% yield without detectable traces of dimethylcyclized product **4** (Fig. 1d, entry 6) while various other silver(I) salts exhibited low solubility and slow conversion rates. Lastly, reducing the reaction temperature to 0 °C afforded methylcyclized product **2** in 85% yield (Fig. 1d, entry 7, 84% yield after isolation).

**Fig. 2:**
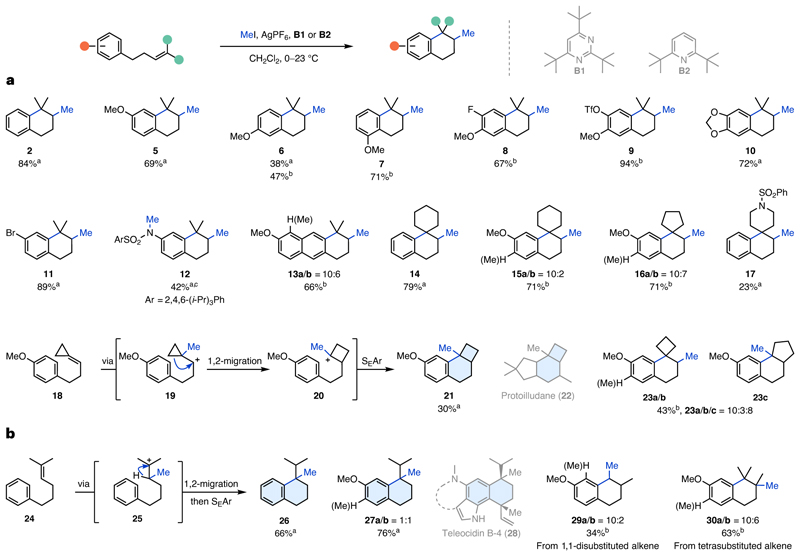
Scope of the silver(I) methyltransfer–cyclization reaction. **a**, Investigation into arene substituents and cyclic alkylidenes to access methylated bi-, tri- and spirocyclic compounds. For cyclopropylidenes and cyclobutylidenes ring expansions are observed. **b**, Investigation into substrates favouring the formation of six-membered rings via 1,2-migration, as well as an exploration of alternative alkene substitution patterns. Reactions were performed on 0.125 mmol scale and all yields are isolated. For substrates with additional arene methylation, the direct product is designated with ‘**a**’, whereas the arene-methylated product is designated with ‘**b**’. ^a^**B1** used. ^b^**B2** used. ^c^From free N–H with increased reagent equivalents.

With the optimized conditions in hand, we investigated the scope of this reaction while varying the temperature to account for substrate-dependent reaction rates. We found that *para*-, *meta*- and *ortho-*methoxy substituents on the arene provided the desired tetralins **5**–**7** in 38–71% yield (Fig. 2a). Unexpectedly, unselective Friedel–Crafts-type arene methylation was observed as a side reaction, especially for **6** and **7**. Gratifyingly, the use of **B2** was able to partially suppress this reactivity, increasing the yield for **6** from 38% to 47% (see Supplementary Section 2.5.1). The presence of an additional F or TfO substituent was well tolerated providing **8** and **9** in 67% and 94% yield, respectively. To our delight, a 1,3-dioxole moiety gave the cyclized product **10** in 72% yield without apparent arene methylation. A *para*-bromo substituent afforded tetralin **11** in excellent yield (89%). An aniline required prior protection with a sulfonyl group (2,4,6-(*i*-Pr)_3_PhSO_2_), but otherwise underwent the desired NEMTC reaction with concomitant N methylation to **12** (42% yield). Exchanging the arene for a naphthalene gave methoxy-substituted tetrahydroanthracene **13a**/**b** in 66% yield. The use of cyclic alkylidenes enabled access to the tricyclic spiro-motifs **14** (79%), **15a**/**b** (71%) and **16a**/**b** (71%) in good yields. Introduction of a protected piperidine led to formation of spirocycle **17** (23%) but was accompanied by several unidentified side products. Subjecting cyclopropylidene **18** to the reactions conditions provided tricycle **21** (30%), whose 6/4-subunit is featured in the protoilludane scaffold (**22**) of natural products^43^. The formation of **21** can be rationalized through formation of the most stable cyclopropyl cation **19**, followed by Wagner–Meerwein shift to **20** and nucleophilic attack by the arene. For the cyclobutylidene, both methylation and cyclization to the spirocycle **23a**/**b** and ring expansion to tricycle **23c** were observed. Interestingly, similar reactivity was observed when formation of a seven-membered ring was attempted (Fig. 2b). Exposure of alkene **24** to the reaction conditions led to exclusive formation of tetralin **26**, which features a quaternary centre substituted with an isopropyl and a methyl group. We assume that the initially formed tertiary cation **25** undergoes a 1,2-hydride shift followed by six-membered ring closure. This sequence is in accordance with the proposed biosynthesis of teleocidin B-4 (**28**)^13^. For the analogous *para*-methoxy substrate the same transformation leading to **27a**/**b** (76%) was observed. With regard to the degree of alkene substitution, a 1,1-disubstituted alkene was suitable for productive methyltransfer leading to the formation of tetralin **29a**/**b** (34%) via a methylation/1,2-hydride shift/cyclization cascade. A tetrasubstituted alkene also underwent NEMTC reaction to give **30a**/**b** in 63% yield (for the comprehensive substrate scope, including limitations such as ring sizes, alkene substitution and different initiating groups, see Supplementary Sections 2.2 and 2.3).

**Fig. 3:**
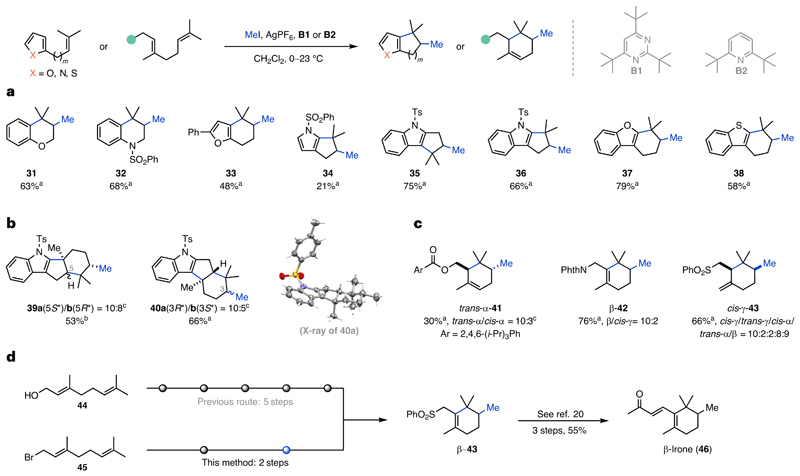
Extended scope of the non-enzymatic methyltransfer–cyclization reaction. **a**, Construction of heterocycles and termination by heteroarenes. **b**, Bicyclization of indoles. **c**, Synthesis of functionalized methylated cyclohexenes bearing the irone core motif. For simplicity only the major product is depicted. **d**, Streamlined formal synthesis of β-irone (**46**). Reactions were performed on 0.125 mmol scale and all yields are isolated. ^a^**B1** used. ^b^**B2** used. ^c^Inseparable.

Further investigation of the potential of this reaction prompted us towards substrates with increased complexity. Prenylated phenols and anilines were well tolerated affording chromane **31** (63%) and tetrahydroquinoline **32** (68%) in good yields (Fig. 3a). To our delight, both a furan and an *N*-sulfonylated pyrrole were competent terminating groups, affording the corresponding bicycles **33** (48%) and **34** (21%). *N*-Sulfonylated indoles, a benzofuran and a benzothiophene were also suitable substrates for the NEMTC reaction, furnishing the desired tricycles **35, 36, 37** and **38** in yields ranging from 58 to 79%. We were pleased to find that 2-geranyl- and 3-geranyl-substituted indoles underwent bicyclization to afford tetracycles **39a**/**b** and **40a**/**b** in good yields (53–66%) (Fig. 3b). Pure **40a** could be obtained through crystallization, allowing structure validation by single-crystal X-ray analysis. Based on the importance of the family of irones for the perfume industry, we also explored multiple functionalized geranyl cyclization precursors to access the irone motif (Fig. 3c). To our surprise the choice of the functional group had a dramatic effect on the relative stereochemistry (*cis* versus *trans*) and the alkene regioselectivity (α, trisubstituted; β, tetrasubstituted and γ, 1,1-disubstituted). Subjecting 2,4,6-triisopropylbenzoyl-protected geraniol to the NEMTC conditions resulted in a mixture of *cis*- and *trans*-α-cyclohexenes **41** (30%). Notably, the sterically encumbered ester is not completely tolerated as methyl 2,4,6-triisopropylbenzoate was also isolated. Surprisingly, a phthalimide moiety was superior in the reaction and furnished methylated β-cyclohexene **42** in good yield (65%) and high selectivity, along with *cis*-γ-cyclohexene **42** (11%). Employing a phenyl sulfone instead afforded a mixture of products **43** (α, β, γ), including the *cis* and *trans* isomers in a combined yield of 66%. The formation of β-**43** represents a shortened formal synthesis (five steps) of β-irone (**46**), which was previously synthesized in eight steps from geraniol (**44**) (Fig. 3d)^20^. While the previously established protocol requires substantial skeleton editing before cyclization, the NEMTC platform makes direct use of canonical terpenoid buildings block (two steps to **43** from geranyl bromide (**45**)).

**Fig. 4:**
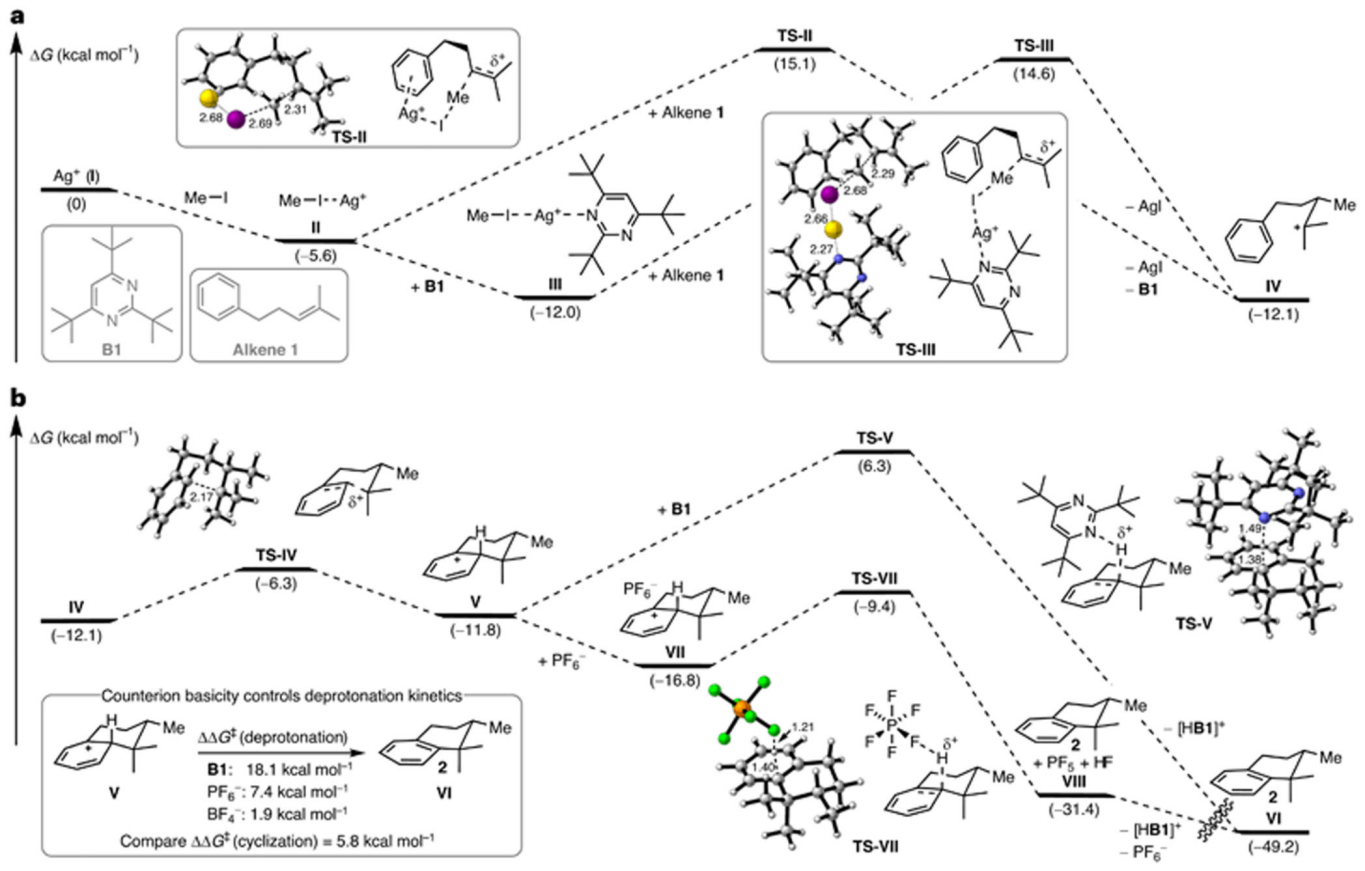
Mechanistic density functional theory studies. **a**, Complexation of the silver(I) ion (**I**) with either methyl iodide to complex **II** or methyl iodide and **B1** to complex **III** generates electrophilic methylating agents. Methyltransfer to alkene **1** proceeds via **TS-II** and **TS-III** through an S_N_2-type mechanism and was found to be rate limiting. Tertiary cation **IV** and silver(I) iodide are formed after complex dissociation. **b**, Tertiary cation **IV** undergoes nucleophilic attack by the arene via **TS-IV** to give Wheland intermediate **V**. Deprotonation of Wheland intermediate **V** by base **B1** via **TS-V** is disfavoured compared to deprotonation via contact ion pair **VII** (adduct of PF_6_^−^ and **V**) and **TS-VII**. The choice of the counterion is crucial for controlling the deprotonation kinetics. The conformational space was explored using CREST^49^ and low energy conformers were further refined with wB97XD/def2-TZVP(IEFPCM(CH_2_Cl_2_)). For transition states selected distances are given in angstrom.

The observed ring expansions and 1,2-hydride shifts during our substrate scope investigations led us to propose a cationic reaction pathway. To test this hypothesis and gain further insights into the reaction mechanism, we conducted density functional theory calculations (wB97XD^44^/def2-TZVP^45,46^(IEFPCM(CH_2_Cl_2_))). Starting from an implicitly solvated silver(I) ion (**I**), we compared various silver(I) complexes containing at least one methyl iodide ligand, which are expected to resemble reactive methylating species (Fig. 4a). Indeed, for complex [Ag(IMe)]^+^ (**II**) and complex [Ag(**B1**)(IMe)]^+^ (**III**) we were able to identify energetically comparable transition states, **TS-II** and **TS-III**, both of which are presumably operative (the energy difference is below the accuracy of density functional theory). A comprehensive overview of additional silver(I) complexes and transition states can be found in Supplementary Section 3.2 and Supplementary Figs. 1 and 2. Methyltransfer proceeds with an S_N_2-type mechanism with the methyl group adopting a trigonal planar conformation with elongated perpendicular ‘bonds’ to the alkene and the iodine. Assuming a Curtin–Hammett-type scenario with comparatively fast interconversion between **II** and **III**, the difference in reaction rate is only determined by ΔΔ*G*^‡^ indicating approximately twofold faster conversion via **TS-III** compared to **TS-II** (ΔΔ*G*^‡^ = 0.5 kcal mol^−1^). Of note, during the reaction development a combination of AgBF_4_ and MeI was found to be sufficient for product formation, indicating that **B1** is not essential for methyltransfer. Dissociation of silver(I) iodide (and **B1**) gives the tertiary cation **IV**, which is poised to undergo nucleophilic attack by the arene (**TS-IV**, Δ*G*^‡^ = 5.8 kcal mol^−1^) to form the Wheland intermediate **V** (Fig. 4b). Search for an alternative concerted methyltransfer–cyclization transition state resulted only in identification of a higher lying methyltransfer transition state (see Supplementary Section 3.2 and Supplementary Fig. 2). The profound influence of the silver(I) counterion on the formation of dimethylcyclized product **4** (compare Fig. 1) indicated that the counterion may play a crucial role for the deprotonation rather than **B1**. Indeed, deprotonation of **V** by pyrimidine **B1** to rearomatized tetralin **2** and pyrimidinium [H**B1**]^+^ (**VI**) exhibits an exceedingly high transition state (**TS-V**, Δ*G*^‡^ = 18.1 kcal mol^−1^) compared to deprotonation from the contact ion pair **VII** (adduct of PF_6_^−^ and **V**) via **TS-VII** (Δ*G*^‡^ = 7.4 kcal mol^−1^). **TS-VII** collapses to tetralin **2**, PF_5_ and HF (**VIII**), which undergoes proton exchange with **B1** to regenerate PF_6_^−^ (**VI**). In agreement with our reaction development, BF_4_^−^ exhibits a substantially lower deprotonation barrier (see Supplementary Section 3.2 and Supplementary Fig. 3), which presumably causes premature deprotonation of tertiary cation **IV** leading to dimethylcyclized product **4**. Overall, the proposed mechanism involves three distinct steps: (1) rate-limiting methyltransfer; (2) cyclization; and (3) deprotonation/rearomatization. Notably, pyrimidine **B1** has little effect on the methyltransfer step and is primarily required to act as the terminal proton acceptor while being resistant to N methylation.

## Conclusion

A non-enzymatic alternative to bMTCs found in nature has been developed, directly enabling the generation of the corresponding naturally occurring substitution patterns. The NEMTC reaction reported here represents a fundamental advancement in (poly)cyclization methodology and provides simple and selective access to methylated spiro-, bi-, tri- and tetracyclic compounds using commercially available reagents. The unique products of this transformation may be especially interesting with regard to the profound efficiency boost often observed in drug discovery programmes upon introduction of methyl groups (‘magic methyl effect’)^47,48^. The impact of this methodology is further emphasized by its compatibility with a diverse array of substrates and its complementarity to previously established modes for C methylation (hydromethylation of alkenes and acidic C–H alkylation). Given its simplicity, ease of operation and uniqueness, we expect widespread application and further research of the NEMTC reaction.

## Online content

Any methods, additional references, Nature Portfolio reporting summaries, source data, extended data, supplementary information, acknowledgements, peer review information; details of author contributions and competing interests; and statements of data and code availability are available at https://doi.org/10.1038/s41557-025-01774-3.

## Methods

### General procedure for the non-enzymatic methyltransfer– cyclization reaction

A vial was charged with silver hexafluorophosphate (AgPF_6_, 63.2 mg, 250 µmol, 2.00 equiv.) in the glovebox and sealed under argon atmosphere using a rubber septum. To this vial was added, in succession, a solution of base **B1** or **B2** (100 mM in dichloromethane, 2.50 ml, 250 µmol, 2.00 equiv.), a solution of the alkene (50.0 mM in dichloromethane, 2.50 ml, 125 µmol, 1 equiv.) and a solution of methyl iodide (625 mM in dichloromethane, 600 µl, 375 µmol, 3.00 equiv.) at 0, 10 or 23 °C. The reaction was monitored by thin-layer chromatography or NMR spectroscopy and stopped by addition of triethylamine (100 µl, 717 µmol, 5.74 equiv.) after no further conversion was observed. The reaction mixture was stirred for 10 min at 23 °C, after which the solvent was removed under reduced pressure. The residue was purified either by flash column chromatography on silica gel or by semipreparative normal-phase high-performance liquid chromatography to afford the methylcyclized product.

## Supplementary Material

SI

## Data Availability

All data are available in Supplementary Information. Supplementary crystallographic data for **40a** (CCDC 2365218) can be obtained free of charge from the Cambridge Crystallographic Data Centre CCDC deposition service via www.ccdc.cam.ac.uk/structures on quoting the deposition number CCDC 2365218. Associated data from the quantum chemical calculations, such as coordinates of optimized structures, are available in Supplementary Information and as separate XMOL files.
