# Supplementary material for: Non-enzymatic methylcyclization of alkenes
Source: Nat Chem. 2025 Mar 7;17(6):904–10. doi: 10.1038/s41557-025-01774-3 (PMC7617478; doi:10.1038/s41557-025-01774-3)
Supplement: Supplementary file 1 — Supplementary Figs. 1–233, reaction development, experimental procedures, characterization data, substrate limitations and computational details. [file 41557_2025_1774_MOESM1_ESM.pdf]

nature chemistry

Supplementary information https://doi.org/10.1038/s41557-025-01774-3

Non-enzymatic methylcyclization of alkenes

In the format provided by the authors and unedited

Table of Content

1. General experimental details 5
2. Experimental part 8
2.1 Reaction development & optimization 8
2.1.1 Screening of electrophilic methylation agents.................... 8
2.1.2 Silver salts screening 10
2.1.3 Base additive screening 11
2.1.4 Solvent screening....................12
2.1.5 Concentration screening 12
2.1.6 Temperature screening 13
2.2 General procedure and substrate scope....................14
2.2.1 General procedure 1 (GP1) 14
2.2.2 General procedure 2 (GP2) 14
2.2.3 Tetralin **2** 15
2.2.4 Tetralin **5** 15
2.2.5 Tetralin **6** 16
2.2.6 Tetralin **7** 17
2.2.7 Fluorotetralin **8** 18
2.2.8 Tetralin **9** 18
2.2.9 1,3-Dioxole **10** 19
2.2.10 Tetralin **11** 20
2.2.11 Tetralin **12** 20
2.2.12 Tetrahydroanthracenes **13** 21
2.2.13 Spirocycle **14**....................22
2.2.14 Spirocycles **15**....................23
2.2.15 Spirocycles **16**....................24
2.2.16 Piperidine **17** 25
2.2.17 Cyclobutane **21** 26
2.2.18 Spirocycles **23** and cyclopentane **23c** 27
2.2.19 Tetralin **26** 29
2.2.20 Tetralins **27** 29
2.2.21 Tetralins **29** 31
2.2.22 Tetralins **30** 33
2.2.23 Chromane **31** 34
2.2.24 Tetrahydroquinoline **32**....................34
2.2.25 Furan **33**....................35
2.2.26 Pyrrole **34**....................36

2.2.27 Indole **35** 37
2.2.28 Indole **36** 37
2.2.29 Benzofuran **37** 39
2.2.30 Benzothiophene **38** 39
2.2.31 Tetracycle **39** 40
2.2.32 Tetracycle **40** 41
2.2.33 Cyclohexenes **41** 43
2.2.34 Cyclohexenes **42** 44
2.2.35 Cyclohexenes **43** 46
2.3 Limitations 49
2.3.1 Attempted cyclization of alkene **S35** 49
2.3.2 Attempted cyclization of alkyne **43** 50
2.3.3 Attempted cyclization of cyclopentene ether **S54** 51
2.4 Substrate synthesis 53
2.4.1 Alkene **1** 53
2.4.2 Alkene **S1** 53
2.4.3 Alkene **S2** 54
2.4.4 Alkene **S3** 55
2.4.5 Alkene **S4** 56
2.4.6 Alkene **S5** 58
2.4.7 Alkene **S6** 60
2.4.8 Alkene **S7** 61
2.4.9 Alkene **S8** 62
2.4.10 Alkene **S9** 64
2.4.11 Phosphonium salt **S83** 66
2.4.12 Alkene **S10** 66
2.4.13 Phosphonium salt **S87** 67
2.4.14 Alkene **S11** 68
2.4.15 Alkene **S12** 69
2.4.16 Piperidine **S13** 70
2.4.17 Alkene **18** 71
2.4.18 Alkene **S14** 72
2.4.19 Alkene **24** and aldehyde **S41** 73
2.4.20 Alkene **S15** 74
2.4.21 1,1-Disubstituted alkene **S16** 75
2.4.22 Tetrasubstituted alkene **S18** 76
2.4.23 Phenyl prenyl ether **S19** 76
2.4.24 Alkene **S20** 77

2.4.25 Alkene **S21** ... 78
2.4.26 Prenyl pyrrole **S22** ... 80
2.4.27 2-Prenyl indole **S23** ... 82
2.4.28 3-Prenyl indole **S24** ... 83
2.4.29 Alkene **S25** ... 84
2.4.30 Alkene **S26** ... 85
2.4.31 3-Geranyl indole **S27** ... 86
2.4.32 2-Geranyl indole **S28** ... 87
2.4.33 TIB-protected geraniol **S29** ... 88
2.4.34 Alkene **S31** ... 89
2.4.35 Geranyl phenyl sulfone (**S33**) ... 89
2.4.36 Alkene **S35** ... 90
2.4.37 Alkene **S36** ... 90
2.4.38 Alkene **S37** ... 92
2.4.39 Alkene **S38** ... 94
2.4.40 Alkene **S39** ... 95
2.4.41 Enol ether **S40** ... 97
2.4.42 Epoxide **S42** ... 98
2.4.43 Alkyne **S43** ... 98
2.4.44 Alkyne **S44** ... 99
2.4.45 Allene **S45** ... 100
2.4.46 Monosubstituted alkene **S46** ... 100
2.4.47 Alkene **S47** ... 101
2.4.48 Alkene **S48** ... 101
2.4.49 Diene **S49** ... 102
2.4.50 Diene **S50** ... 103
2.4.51 Alkene **S51** ... 106
2.4.52 Alkene **S52** ... 108
2.4.53 Alkene **S53** ... 109
2.4.54 Cyclopentene ether **S54** ... 111
2.4.55 TIPS-protected geraniol **S55** ... 113
2.4.56 Boc-protected geraniol **S56** ... 114
2.5 Miscellaneous ... 114
2.5.1 Influence of base additive on arene methylation ... 114
2.5.2 Screening of electrophiles ... 115
2.5.3 Synthesis of 2,6-(*t*-Bu)$_2$-4-MeO-pyridine **B8** ... 117
2.5.4 Tetralin **4** ... 119
2.5.5 Ketone **S148** ... 121

2.5.6 Ketone **S149** 122
2.5.7 Standard conditions without methyl iodide 123
2.5.8 Complexation of the silver(I)-ion by **B1** 123
3. Computational studies 125
3.1 Computational Methodology 125
3.2 Reaction pathway 125
3.3 Cartesian Coordinates 129
4. X–ray 163
4.1 Tetracycle **40a** 163
5. NMR Spectra 165
6. References 290

1. General experimental details

All reactions were performed in oven-dried glassware (110 °C oven temperature) with magnetic stirring under argon or nitrogen atmosphere, unless otherwise noted, using standard Schlenk techniques. If necessary, glassware was further dried under high-vacuum with a heat-gun at 650 °C. Temperature control was performed by external bath thermometers. High temperature reactions were either carried out using a reaction flask connected to a reflux condenser or in sealed pressure tubes while heating with a silicon oil bath or a metal block. Low temperature reactions were either conducted using a distilled water/ice bath (0 °C) or using an acetone bath (Dewar vessel) in combination with an electronically controlled cryostat (−78 °C to 0 °C, 10 °C) or a Dewar vessel filled with dry ice/acetone (−78 °C). Diethyl ether and tetrahydrofuran (THF) were dried over molecular sieves (4Å) prior to use. All other solvents were purchased from Acros Organics (Fisher Scientific) or Sigma Aldrich as 'extra dry' reagents. If required, solvents were either degassed by bubbling argon through the solvent under simultaneous sonication for at least 30 min. Solvents for extractions and flash column chromatography (FCC) were purchased in technical grade and purified by distillation prior to use. All reagents were obtained from commercial sources (Sigma Aldrich, Acros Organics (Fisher Scientific), Alfa Aesar, Tokyo Chemical Industry, BLD Pharmatech, Fluorochem, abcr, and ChemPUR) with a purity >95% and used without further purification unless otherwise noted. Particularly moisture or air sensitive reagents were handled in a glovebox. Transfer of these sensitive reagents or solutions of these was performed under argon atmosphere via syringes through rubber septa. If not noted otherwise, concentration of reaction mixtures or combined organic layers after extraction was performed on rotary evaporators with a bath temperature of 40 °C.

Flash column chromatography (FCC) was carried out using Merck silica gel 60 (0.040–0.063 mm). Analytical thin layer chromatography (TLC) was carried out using Merck silica gel 60 F254 aluminum foils and visualized under UV light at 254 nm or by staining with either ceric ammonium molybdate (CAM) or an aqueous potassium permanganate ($KMnO_4$) solution and subsequent heating. Automated flash column chromatography was performed using a puriFlash XS520+ (Advion Interchim Scientific) with prepacked cartridges (irregular silica gel, 0.040–0.063 mm, 60 Å).

High performance liquid chromatography (HPLC) was conducted either on a normal-phase Varian Dynamax column (250 × 41.4 mm, Microsorb 60-8 Si column) for preparative separations or on a normal-phase Shimadzu Shim-pack PRC-SIL(H) column (250 x 20 mm, 5 µm particle diameter) for semipreparative separations.

NMR spectra (^{1}H NMR, ^{13}C NMR and ^{19}F NMR) were recorded in deuterated chloroform (chloroform-*d*) or deuterated benzene (benzene-d_6) on a Bruker Avance Neo 400 MHz spectrometer, a Bruker Avance II 600 MHz spectrometer, or a Bruker Avance 4 Neo 700 MHz spectrometer. For ^{1}H NMR spectra the residual proton peak of the respective solvent (chloroform-*d*: 7.26 ppm, benzene-d_6: 7.16 ppm) served as internal reference. ^{1}H spectroscopic data is reported as follows: chemical shift δ in ppm (multiplicity, coupling constant *J* in Hz, number of protons). Multiplicities are abbreviated as follows: s = singlet, d = doublet, t = triplet, q = quartet, p = quintet, h = hextet, hept = heptet, br = broad, m = multiplet, or combinations thereof. Combined multiplicities are listed in order of their respective coupling constant *J* starting with the highest one. For ^{13}C NMR the central ^{13}C resonance of the respective solvent (chloroform-*d*: 77.16 ppm, benzene-d_6: 128.06 ppm) served as internal reference and ^{13}C spectroscopic data is reported as follows: chemical shift δ in ppm (number of carbons in parenthesis if >1). NMR spectra were assigned using information ascertained from COSY, HMBC, HSQC and NOESY experiments. ^{19}F NMR spectra were externally referenced ($CFCl_3$) and are ^{1}H-decoupled if not stated otherwise.

High resolution mass spectra (HRMS) were recorded on a Thermo Scientific™ LTQ Orbitrap XL™ Hybrid Ion Trap-Orbitrap Mass Spectrometer at the Institute of Organic Chemistry and Center for Molecular Biosciences, University of Innsbruck.

Infrared spectra (IR) were recorded from 4000 cm^{-1} to 450 cm^{-1} on a Bruker™ ALPHA FT-IR spectrometer from Bruker. Samples were measured as a neat film by evaporation of a solution in chloroform-*d*. IR data is reported as follows: frequency of absorption in cm^{-1} (absorption intensity), whereby the absorption intensity is abbreviated as follows: w = weak, m = medium, s = strong, br = broad or combinations thereof.

Melting Points were measured with a SRS MPA120 EZ-Melt Melting Point Apparatus in open glass capillaries and are uncorrected.

For **X-ray diffraction analysis**, data collections were performed on a Bruker D8Quest using MoKα-radiation (λ = 0.71073 Å, Incoatec Microfocus). The Bruker Apex III software was applied for the integration, scaling and multi-scan absorption correction of the data. Structures were solved by direct methods with SHELXTL-XT-2014. Structure refinement was performed by least-squares methods against F2 with SHELXL-2014/7. All non-hydrogen atoms were refined anisotropically. The hydrogen atoms were placed in ideal geometry riding on their parent atoms. Relevant details of the data collection and evaluation are listed in tables at the corresponding sections. Supplementary crystallographic data for **40a** (CCDC 2365218) can be obtained from the Cambridge Crystallographic Data Centre CCDC deposition service via www.ccdc.cam.ac.uk/structures on quoting the deposition number CCDC 2365218. Further

details are summarized in the tables at the corresponding sections. Plotting of thermal ellipsoids in this document and in the main text was carried out using MERCURY for Windows at 50% probability level.

All yields are isolated, unless otherwise specified.

2. Experimental part

2.1 Reaction development & optimization

2.1.1 Screening of electrophilic methylation agents

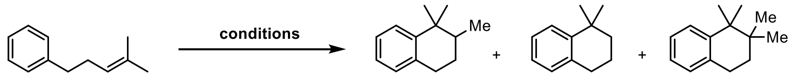

1 **2** *methylcyclized* **3** *protocyclized* **4** *dimethylcyclized*

entry	reagent	solvent	temp.	time	scale	NMR yield 2	3	4	1
1	$Me_3S^+I^-$	CH_2Cl_2 (22 mM)	40 °C	18 h	125 µmol	—	—	—	95%
2	$Me_3S^+I^-$	DCE (22 mM)	80 °C	18 h	125 µmol	—	—	—	89%
3	$Me_3S^+I^-$	HFIP (22 mM)	55 °C	18 h	125 µmol	—	54%	—	22%
4	$Me_3S^+I^-$, **B2**	HFIP (22 mM)	55 °C	18 h	125 µmol	—	—	—	7%[a]
5	$Me_3S^+I^-$, $AgBF_4$	CH_2Cl_2 (22 mM)	40 °C	18 h	125 µmol	—	—	—	97%
6	$Me_3S^+I^-$, $AgBF_4$	DCE (22 mM)	80 °C	18 h	125 µmol	—	32%	—	60%
7	$Me_3S^+I^-$, $AgBF_4$, **B2**	DCE (22 mM)	80 °C	18 h	125 µmol	—	—	—	91%
8	$Me_3SO^+I^-$	CH_2Cl_2 (22 mM)	40 °C	18 h	125 µmol	—	—	—	95%
9	$Me_3SO^+I^-$	DCE (22 mM)	80 °C	18 h	125 µmol	—	—	—	87%
10	$Me_3SO^+I^-$	HFIP (22 mM)	55 °C	18 h	125 µmol	—	51%	—	30%
11	$Me_3SO^+I^-$, **B2**	HFIP (22 mM)	55 °C	18 h	125 µmol	—	—	—	86%
12	$Me_3SO^+I^-$, $AgBF_4$	CH_2Cl_2 (22 mM)	40 °C	18 h	125 µmol	—	—	—	93%
13	$Me_3SO^+I^-$, $AgBF_4$	DCE (22 mM)	80 °C	18 h	125 µmol	—	6%	—	83%
14	$Me_3SO^+I^-$, $AgBF_4$, **B2**	DCE (22 mM)	80 °C	18 h	125 µmol	—	—	—	89%
15	Me_3OBF_4, **B2**	DCE (22 mM)	80 °C	18 h	125 µmol	5%	—	—	75%
16	MeOTf, **B2**	DCE (22 mM)	80 °C	18 h	125 µmol	2%	—	—	72%
17	$EtAlCl_2$, ClC(O)OMe	CH_2Cl_2 (56 mM)	–20 → 23 °C	15 h	141 µmol	—	79%	—	—
18	MeI, $AgBF_4$	CH_2Cl_2 (22 mM)	23 °C	15 h	278 µmol	9%	73%	—	—
19	MeI, $AgBF_4$, **B2**	CH_2Cl_2 (22 mM)	23 °C	18 h	125 µmol	62%	—	19%	—
20	MeI, $AgBF_4$, **B1**	CH_2Cl_2 (22 mM)	23 °C	18 h	125 µmol	70%	—	22%	—

[a]Loss of **1** due to volatility.

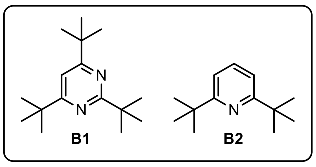

Summary: Electrophilic sulfur(IV) and sulfur(VI) reagents (entry 1–14) such as trimethylsulfonium iodide and trimethylsulfoxonium iodide, which resemble analogous reagents to Nature's *S*-adenosyl methionine (a sulfur (IV) reagent), exhibited either no conversion or exclusively protocyclization either in 1,1,1,3,3,3-hexafluoro-2-propanol (HFIP; entry 3 & 10) or upon exchange of the counterion with silver tetrafluoroborate (entry 6 & 13). Addition of a sterically demanding proton scavenger such as **B2** in combination with silver tetrafluoroborate resulted in no conversion (entry 7 & 14). Strong methylating reagents such as Meerwein's salt (trimethyloxonium tetrafluoroborate, entry 15) and methyl triflate (entry 16) in combination with **B2** proceeded with slow conversion to the desired product **2** (<10% NMR yield after 18 h at 80 °C). Employing aluminum Lewis-acids together with methyl chloroformate[1] exclusively culminated in protocyclized product **3** (entry 17). A reactive system constituted of silver tetrafluoroborate and methyl iodide (entry 18) enabled complete conversion already at 23 °C to protocyclization product **3** (73% NMR yield) and mono-methylation product **2** (9% NMR yield). Inclusion of a proton scavenger **B2** suppressed H^+-

mediated cyclization completely and afforded a mixture of methylcyclized and dimethylcyclized products **2** and **4** in 81% combined NMR yield (entry 19). Exchange of the proton scavenger to **B1** improved the combined NMR yield to 92% (entry 20). Further reaction optimization is described in the following subsections.

General procedure for entry 1–16 and 18–20: To a solution of alkene **1** (1 equiv) in the indicated solvent (dichloromethane, 1,2-dichloroethane (DCE) or 1,1,1,3,3,3-hexafluoro-2-propanol (HFIP); at the indicated concentration) were added the indicated reagents (2.00 equiv; for methyl iodide: 3.00 equiv; order of addition: first additives such as **B1**, **B2** or silver salts, then the electrophilic methyl source) at 23 °C. The reaction mixture was stirred at the indicated temperature for the indicated time, after which the reaction was stopped through addition of triethylamine (5.75 equiv). The reaction mixture was concentrated under reduced pressure (40 °C, down to 80 mbar) and the residue was filtered through a short silica plug, which was eluted with four column volumes of dichloromethane. The filtrate was concentrated under reduced pressure (40 °C, down to 80 mbar). To the residue was added 1,1,2,2-tetrachloroethane as an NMR standard and the yield was determined through quantitative NMR analysis.

Entry 17: To a solution of alkene **1** (22.6 mg, 141 µmol, 1 equiv) in dichloromethane (2.50 mL) were added in succession a solution of methyl chloroformate (13.3 mg, 141 µmol, 1.00 equiv) in dichloromethane (100 µL) and a solution of ethyl aluminum dichloride (1.00 M in hexanes, 141 µL, 141 µmol, 1.00 equiv) at –20 °C. The reaction mixture was allowed to warm up to 23 °C in the cooling bath and stirring was continued for 15 h, after which triethylamine (113 µL, 811 µmol, 5.75 equiv) was added. The reaction mixture was filtered through a short silica plug, which was eluted with four column volumes of dichloromethane. The filtrate was concentrated under reduced pressure (40 °C, down to 80 mbar). To the residue was added 1,1,2,2-tetrachloroethane as an NMR standard and the yield was determined through quantitative NMR analysis.

Analytical data for methylcyclized product **2** were in accordance with reported literature values.[2]

Analytical data for protocyclized product **3** were in accordance with reported literature values.[3]

For characterization data of dimethylcyclized product **4** see chapter 2.5.4.

2.1.2 Silver salts screening

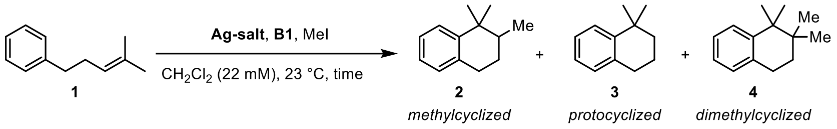

entry	Ag-salt	time	NMR yield 2	3	4	1	ratio 2:3
1	$AgPF_6$	4.5 h	80%	—	—	—	>20:1
2	$AgBF_4$	4.5 h	54%	—	14%	11%	4.0:1
3	$AgSbF_6$	20 h	76%	—	—	11%	>20:1
4	AgOTf	20 h	10%	—	2%	57%	4.3:1
5	$AgClO_4$	20 h	7%	—	2%	59%	3.1:1
6	$AgNO_3$	20 h	—	—	—	89%	—

Conclusion: $AgPF_6$ (entry 1) turned out to be the most suitable silver salt due to (a) the low basicity of the PF_6^- counterion compared to BF_4^- (entry 2), which suppresses formation of dimethylcyclized product **4**, and (b) its high solubility and reaction rate (as opposed to $AgSbF_6$, AgOTf, $AgClO_4$, and $AgNO_3$; entry 3–6).

General procedure: To a vial charged with the indicated silver salt (2.00 equiv) were added in succession a solution of 2,4,6-tri-*tert*-butylpyrimidine (**B1**) (100 mM in dichloromethane, 2.50 mL, 250 µmol, 2.00 equiv), a solution of alkene **1** (50.0 mM in dichloromethane, 2.50 mL, 125 µmol, 1 equiv), and a solution of methyl iodide (625 mM in dichloromethane, 600 µL, 375 µmol, 3.00 equiv) at 23 °C. The reaction was stirred for the indicated time at 23 °C and stopped by addition of triethylamine (100 µL, 717 µmol, 5.74 equiv). After stirring for 10 min at 23 °C, the solvent was removed under reduced pressure (40 °C, down to 100 mbar) and the residue was filtered through a silica plug, which was eluted with four column volumes of 10% dichloromethane in *n*-pentane. The filtrate was concentrated under reduced pressure (40 °C, down to 100 mbar). To the residue was added 1,1,2,2-tetrachloroethane as an NMR standard and the yield was determined through quantitative NMR analysis.

2.1.3 Base additive screening

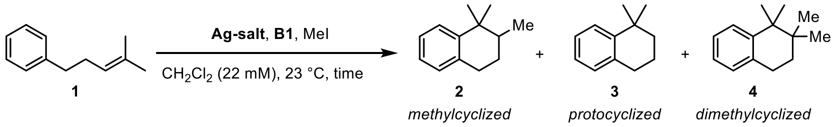

entry	base	time	NMR yield 2	3	4	1	ratio 2:3
1	**B1**	4.5 h	80%	—	—	—	>20:1
2	**B2**	4.5 h	80%	—	—	—	>20:1
3	**B7**	20 h	80%	—	—	—	>20:1
4	**B8**	20 h	82%	—	—	—	>20:1
5	**B3**	20 h	73%	—	—	15%	>20:1
6	**B4**	20 h	29%	—	—	58%	>20:1
7	**B5**	20 h	23%	—	—	62%	>20:1
8	**B6**	20 h	—	—	—	84%	>20:1

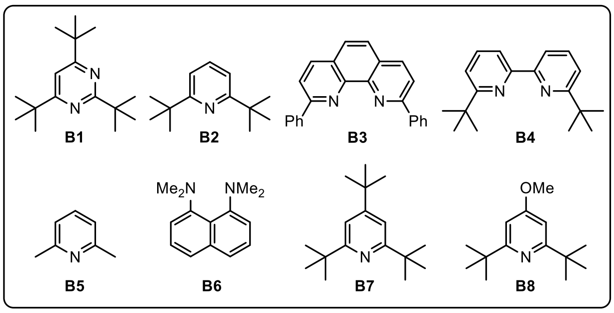

Conclusion: Base **B1**, **B2**, **B7** and **B8** (entry 1–4) are all highly compatible with the reaction conditions and give comparable results. **B8** might perform marginally better than **B1**, however, it is not commercially available and was thus excluded from further experimentation. In contrast, **B3**–**B6** (entry 5–8) suffer from slow conversion and/or methylation of the base additive.

General procedure: To a vial charged with silver hexafluorophosphate ($AgPF_6$, 63.2 mg, 250 µmol, 2.00 equiv) in the glovebox was added in succession a solution of the indicated base (100 mM in dichloromethane, 2.50 mL, 250 µmol, 2.00 equiv), a solution of alkene **1** (50.0 mM in dichloromethane, 2.50 mL, 125 µmol, 1 equiv), and a solution of methyl iodide (625 mM in dichloromethane, 600 µL, 375 µmol, 3.00 equiv) at 23 °C. The reaction was stirred for the indicated time at 23 °C and stopped by addition of triethylamine (100 µL, 717 µmol, 5.74 equiv). After stirring for 10 min at 23 °C, the solvent was removed under reduced pressure (40 °C, down to 100 mbar) and the residue was filtered through a silica plug, which was eluted with four column volumes of 10% dichloromethane in *n*-pentane. The filtrate was concentrated under reduced pressure (40 °C, down to 100 mbar). To the residue was added 1,1,2,2-tetrachloroethane as an NMR standard and the yield was determined through quantitative NMR analysis.

2.1.4 Solvent screening

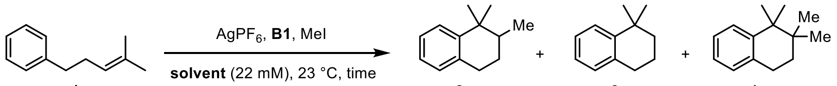

1 → 2 (*methylcyclized*) + 3 (*protocyclized*) + 4 (*dimethylcyclized*)

entry	solvent	time	NMR yield 2	3	4	1	ratio 2:3
1	CH_2Cl_2	4.5 h	80%	—	—	—	>20:1
2	DCE	4.5 h	63%	—	—	23%	>20:1
3	toluene	20 h	64%	—	—	21%	>20:1
4	$MeNO_2$	20 h	6%	—	—	40%	>20:1

Conclusion: While 1,2-dichloroethane and toluene (entry 2 & 3) are compatible with the reaction conditions, dichloromethane (entry 1) enables the fastest reaction rates.

General procedure: To a vial charged with silver hexafluorophosphate ($AgPF_6$, 63.2 mg, 250 µmol, 2.00 equiv) in the glovebox was added in succession a solution of 2,4,6-tri-*tert*-butylpyrimidine (**B1**) (100 mM in the indicated solvent, 2.50 mL, 250 µmol, 2.00 equiv), a solution of alkene **1** (50.0 mM in the indicated solvent, 2.50 mL, 125 µmol, 1 equiv), and a solution of methyl iodide (625 mM in the indicated solvent, 600 µL, 375 µmol, 3.00 equiv) at 23 °C. The reaction was stirred for the indicated time at 23 °C and stopped by addition of triethylamine (100 µL, 717 µmol, 5.74 equiv). After stirring for 10 min at 23 °C, the solvent was removed under reduced pressure (40 °C, down to 60 mbar) and the residue was filtered through a silica plug, which was eluted with four column volumes of 10% dichloromethane in *n*-pentane. The filtrate was concentrated under reduced pressure (40 °C, down to 60 mbar). To the residue was added 1,1,2,2-tetrachloroethane as an NMR standard and the yield was determined through quantitative NMR analysis.

2.1.5 Concentration screening

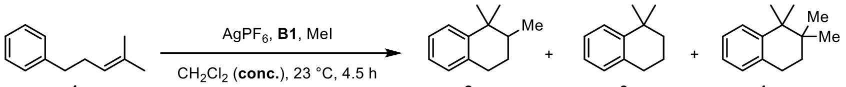

1 → 2 (*methylcyclized*) + 3 (*protocyclized*) + 4 (*dimethylcyclized*)

entry	conc.	NMR yield 2	3	4	1	ratio 2:3
1	22 mM	80%	—	—	—	>20:1
2	10 mM	63%	—	—	27%	>20:1
3	50 mM	67%	—	—	—	>20:1

Conclusion: A reaction concentration of 22 mM was found to be optimal (entry 1) compared to 10 mM (entry 2, slow conversion) and 50 mM (entry 3, decreased yield).

General procedure: To a vial charged with silver hexafluorophosphate ($AgPF_6$, 63.2 mg, 250 µmol, 2.00 equiv) in the glovebox was added in succession a solution of 2,4,6-tri-*tert*-butylpyrimidine (**B1**) (250 µmol, 2.00 equiv) and alkene **1** (125 µmol, 1 equiv) in

dichloromethane (entry 1: 5.00 mL, entry 2: 11.9 mL, entry 3: 1.90 mL) and a solution of methyl iodide (625 mM in dichloromethane, 600 µL, 375 µmol, 3.00 equiv) at 23 °C. The reaction was stirred for 4.5 h at 23 °C and stopped by addition of triethylamine (100 µL, 717 µmol, 5.74 equiv). After stirring for 10 min at 23 °C, the solvent was removed under reduced pressure (40 °C, down to 100 mbar) and the residue was filtered through a silica plug, which was eluted with four column volumes of 10% dichloromethane in *n*-pentane. The filtrate was concentrated under reduced pressure (40 °C, down to 100 mbar). To the residue was added 1,1,2,2-tetrachloroethane as an NMR standard and the yield was determined through quantitative NMR analysis.

2.1.6 Temperature screening

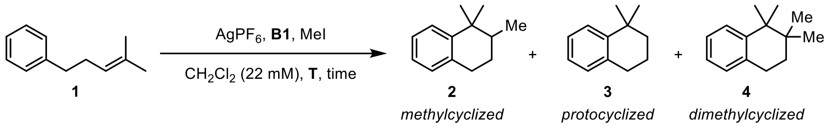

entry	T	time	NMR yield 2	3	4	1	ratio 2:3
1	0 °C	27 h	85%	—	—	—	>20:1
2	10 °C	22 h	78%	—	—	—	>20:1
3	23 °C	4.5 h	80%	—	—	—	>20:1

Conclusion: A lowered reaction temperature (e.g., 0 °C, entry 1) results in a slightly more selective reaction/improved yield at the cost of slower conversion rates.

General procedure: To a vial charged with silver hexafluorophosphate ($AgPF_6$, 63.2 mg, 250 µmol, 2.00 equiv) in the glovebox was added in succession a solution of 2,4,6-tri-*tert*-butylpyrimidine (**B1**) (100 mM in dichloromethane, 2.50 mL, 250 µmol, 2.00 equiv), a solution of alkene **1** (50.0 mM in dichloromethane, 2.50 mL, 125 µmol, 1 equiv), and a solution of methyl iodide (625 mM in dichloromethane, 600 µL, 375 µmol, 3.00 equiv) at the indicated temperature. The reaction was stirred for the indicated time at the indicated temperature and stopped by addition of triethylamine (100 µL, 717 µmol, 5.74 equiv). After stirring for 10 min at 23 °C, the solvent was removed under reduced pressure (40 °C, down to 60 mbar) and the residue was filtered through a silica plug, which was eluted with four column volumes of 10% dichloromethane in *n*-pentane. The filtrate was concentrated under reduced pressure (40 °C, down to 60 mbar). To the residue was added 1,1,2,2-tetrachloroethane as an NMR standard and the yield was determined through quantitative NMR analysis.

2.2 General procedure and substrate scope

2.2.1 General procedure 1 (GP1)

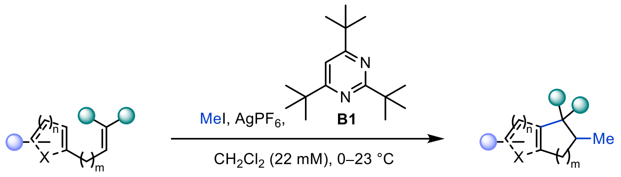

A vial was charged with silver hexafluorophosphate ($AgPF_6$, 63.2 mg, 250 µmol, 2.00 equiv) in the glovebox and sealed under Argon atmosphere using a rubber septum. To this vial were added in succession a solution of 2,4,6-tri-*tert*-butylpyrimidine (**B1**) (100 mM in dichloromethane, 2.50 mL, 250 µmol, 2.00 equiv), a solution of the alkene (50.0 mM in dichloromethane, 2.50 mL, 125 µmol, 1 equiv), and a solution of methyl iodide (625 mM in dichloromethane, 600 µL, 375 µmol, 3.00 equiv) at the indicated temperature. The reaction was monitored by TLC or NMR reaction control and stopped by addition of triethylamine (100 µL, 717 µmol, 5.74 equiv) after no further conversion was observed. If applicable, the reaction mixture was allowed to warm to 23 °C and stirred for 10 min, after which the solvent was removed under reduced pressure. The residue was purified either by flash column chromatography on silica gel or by semipreparative normal-phase high performance liquid chromatography (HPLC) to afford cyclized product.

2.2.2 General procedure 2 (GP2)

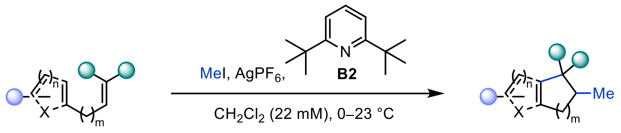

Same as general procedure 1 (GP1) with 2,6-di-*tert*-butylpyridine (**B2**) (100 mM in dichloromethane, 2.50 mL, 250 µmol, 2.00 equiv) instead of 2,4,6-tri-*tert*-butylpyrimidine (**B1**).

2.2.3 Tetralin **2**

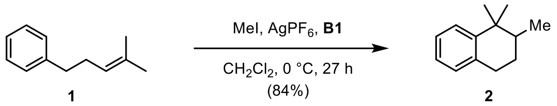

1 **2**

According to GP1, alkene **1** (20.0 mg, 125 µmol, 1 equiv) was converted at 0 °C over 27 h to tetralin **2**. Purification by flash column chromatography on silica gel (1% formic acid in *n*-pentane) afforded tetralin **2** (18.3 mg, 105 µmol, 84%) as a colorless oil.

Analytical data of tetralin **1**:

TLC (*n*-pentane): R_f = 0.74 (UV, CAM).

^{1}H NMR (400 MHz, $CDCl_3$): δ 7.38 (dd, J = 7.8, 1.3 Hz, 1H), 7.18 – 7.13 (m, 1H), 7.08 (td, J = 7.3, 1.4 Hz, 1H), 7.04 (dd, J = 7.9, 1.8 Hz, 1H), 2.81 (dd, J = 7.3, 4.9 Hz, 2H), 1.82 – 1.67 (m, 2H), 1.67 – 1.58 (m, 1H), 1.33 (s, 3H), 1.16 (s, 3H), 1.01 (d, J = 6.7 Hz, 3H).

^{13}C NMR (101 MHz, $CDCl_3$): δ 146.5, 136.0, 129.1, 127.2, 125.9, 125.3, 39.3, 37.3, 30.1, 29.4, 27.4, 26.0, 16.7.

IR (ATR, neat): $\tilde{\nu}$ = 3059 (w), 3015 (w), 2965 (s), 2924 (s), 2877 (m), 1490 (m), 1446 (m), 1388 (w), 1374 (w), 1363 (w), 1284 (w), 1240 (w), 1086 (w), 1043 (w), 781 (w), 758 (s), 728 (m), 554 (w), 455 (w) cm^{-1}.

HRMS (ESI): Mass could not be found due to insufficient ionization with electrospray ionization (ESI). However, after benzylic oxidation to ketone **S146** (chapter 2.5.4) high resolution mass spectrometry confirmed the molecular formula.

The obtained analytical data were in accordance with reported literature values.[2]

2.2.4 Tetralin **5**

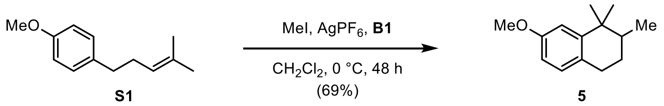

S1 **5**

According to GP1, alkene **S1** (23.8 mg, 125 µmol, 1 equiv) was converted at 0 °C over 48 h to tetralin **5**. Purification by flash column chromatography on silica gel (10% to 25% dichloromethane in *n*-pentane) afforded tetralin **5** (17.5 mg, 85.7 µmol, 69%) as a colorless oil.

Analytical data of tetralin **5**:

TLC (15% dichloromethane in *n*-pentane): R_f = 0.30 (UV, CAM).

^{1}H NMR (400 MHz, $CDCl_3$): δ 6.97 (dt, *J* = 8.2, 1.0 Hz, 1H), 6.92 (d, *J* = 2.6 Hz, 1H), 6.68 (dd, *J* = 8.4, 2.7 Hz, 1H), 3.80 (s, 3H), 2.74 (dd, *J* = 7.4, 5.4 Hz, 2H), 1.81 – 1.73 (m, 1H), 1.72 – 1.65 (m, 1H), 1.64 – 1.54 (m, 1H), 1.32 (s, 3H), 1.15 (s, 3H), 1.00 (d, *J* = 6.7 Hz, 3H).

^{13}C NMR (101 MHz, $CDCl_3$): δ 157.8, 147.8, 129.8, 128.3, 112.7, 111.0, 55.4, 39.1, 37.5, 30.1, 28.5, 27.6, 25.9, 16.7.

IR (ATR, neat): $\tilde{\nu}$ = 2965 (s), 2928 (s), 2878 (m), 2860 (m), 2833 (m), 1610 (s), 1575 (m), 1504 (s), 1495 (s),1464 (s), 1416 (w), 1388 (w), 1374 (w), 1362 (w), 1344 (w), 1311 (w), 1281 (s), 1255 (s), 1239 (s), 1214 (s), 1187 m), 1177 (w), 1132 (w), 1115 (w), 1074 (m), 1043 (s), 933 (w), 872 (w), 852 (w), 801 (m), 784 (w), 735 (w), 706 (w), 472 (w) cm^{-1}.

HRMS (ESI): calcd for $C_{14}H_{21}O^+$ $[M+H]^+$: 205.1587; found: 205.1585.

2.2.5 Tetralin **6**

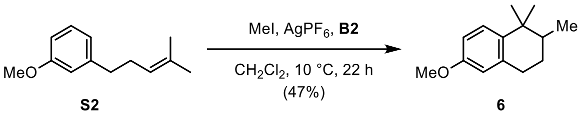

S2 **6**

According to GP2, alkene **S2** (23.8 mg, 125 µmol, 1 equiv) was converted at 10 °C over 22 h to tetralin **6**. Purification by flash column chromatography on silica gel (5% to 17% dichloromethane in *n*-pentane) afforded tetralin **6** (12.1 mg, 59.2 µmol, 47%) as a colorless oil.

For GP1 (47 h at 10 °C; purification as described above):
Tetralin **6** (9.7 mg, 48 µmol, 38%)
Alkene **S2** (6.9 mg, 36 µmol, 29%)

Analytical data of tetralin **6**:

TLC (10% dichloromethane in *n*-pentane): R_f = 0.50 (UV, CAM).

^{1}H NMR (400 MHz, $CDCl_3$): δ 7.29 – 7.26 (m, 1H), 6.73 (ddt, *J* = 8.6, 2.8, 0.7 Hz, 1H), 6.56 (dt, *J* = 2.9, 1.0 Hz, 1H), 3.77 (s, 3H), 2.84 – 2.73 (m, 2H), 1.79 – 1.72 (m, 1H), 1.71 – 1.63 (m, 1H), 1.63 – 1.55 (m, 1H), 1.29 (s, 3H), 1.11 (s, 3H), 0.98 (d, *J* = 6.6 Hz, 3H).

^{13}C NMR (101 MHz, $CDCl_3$): δ 157.1, 138.9, 137.3, 128.2, 113.1, 112.4, 55.3, 39.4, 36.7, 30.2, 29.8, 27.5, 26.0, 16.6.

IR (ATR, neat): $\tilde{\nu}$ = 2958 (s), 2923 (s), 2855 (m), 1609 (m), 1499 (s), 1463 (m), 1375 (w), 1362 (w), 1318 (w), 1283 (w) 1260 (m), 1248 (m), 1162 (w), 1142 (w), 1090 (w), 1040 (m), 865 (w), 850 (w), 832 (w), 815 (w), 804(w) cm^{-1}.

HRMS (ESI): calcd for $C_{14}H_{21}O^+$ [M+H]$^+$: 205.1587; found: 205.1584.

2.2.6 Tetralin **7**

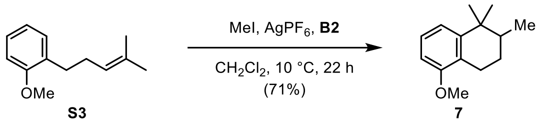

According to GP2, alkene **S3** (23.8 mg, 125 µmol, 1 equiv) was converted at 10 °C over 22 h to tetralin **7**. Purification by flash column chromatography on silica gel (5% dichloromethane in *n*-pentane) followed by semipreparative normal-phase high performance liquid chromatography (HPLC) (0.1% to 1.0% ethyl acetate in *n*-hexane over 40 min) afforded tetralin **7** (18.0 mg, 88.1 µmol, 71%) as a colorless oil.

For GP1 (22 h at 10 °C; purification as described above):
Tetralin **7** (8.9 mg, 44 µmol, 35%)

<u>Analytical data of tetralin **7**:</u>

TLC (10% dichloromethane in *n*-pentane): R_f = 0.33 (UV, CAM).

^{1}H NMR (400 MHz, $CDCl_3$): δ 7.14 (tt, *J* = 8.2, 0.8 Hz, 1H), 7.01 (d, *J* = 8.0 Hz, 1H), 6.65 (dd, *J* = 7.8, 0.8 Hz, 1H), 3.81 (s, 3H), 2.80 (dt, *J* = 17.7, 5.2 Hz, 1H), 2.54 (ddd, *J* = 17.4, 9.4, 6.4 Hz, 1H), 1.80 (dddd, *J* = 13.5, 6.5, 4.6, 2.7 Hz, 1H), 1.71 – 1.63 (m, 1H), 1.61 – 1.54 (m, 1H), 1.30 (s, 3H), 1.15 (s, 3H), 0.99 (d, *J* = 6.8 Hz, 3H).

^{13}C NMR (101 MHz, $CDCl_3$): δ 156.9, 147.9, 126.0, 125.2, 119.3, 106.4, 55.4, 38.6, 37.3, 30.2, 26.7, 25.9, 22.8, 16.6.

IR (ATR, neat): $\tilde{\nu}$ = 3067 (w), 2966 (m), 2932 (m), 2877 (w), 2834 (w), 2358 (w), 2340 (w), 1579 (m), 1458 (s), 1435 (m), 1388 (w), 1374 (w), 1362 (w), 1348 (w), 1309 (w), 1253 (s), 1233 (w), 1208 (w), 1190 (w), 1151 (w), 1096 (w), 1063 (s), 856 (w), 782 (m), 771 (w), 720 (m), 649 (w) cm^{-1}.

HRMS (ESI): calcd for $C_{14}H_{21}O^+$ [M+H]$^+$: 205.1587; found: 205.1588.

2.2.7 Fluorotetralin **8**

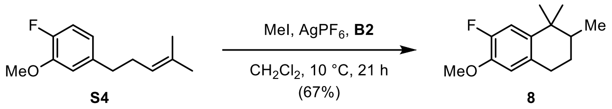

According to GP2, alkene **S4** (26.0 mg, 125 µmol, 1 equiv) was converted at 10 °C over 21 h to fluorotetralin **8**. Purification by flash column chromatography on silica gel (2% ethyl acetate in cyclohexane) followed by semipreparative normal-phase high performance liquid chromatography (HPLC) (0.1% to 2.0% ethyl acetate in *n*-hexane over 40 min) afforded fluorotetralin **8** (18.3 mg, 83.3 µmol, 67%) as a colorless oil.

For GP1 (22 h at 10 °C; purification as described above):
Fluorotetralin **8** (11.6 mg, 52.2 µmol, 42%)

<u>Analytical data of fluorotetralin **8**:</u>

TLC (2% ethyl acetate in cyclohexane): R_f = 0.36 (UV, CAM).

^{1}H NMR (400 MHz, $CDCl_3$): δ 7.04 (d, *J* = 13.6 Hz, 1H), 6.59 (d, *J* = 8.9 Hz, 1H), 3.84 (s, 3H), 2.77 – 2.69 (m, 2H), 1.76 (dtd, *J* = 12.9, 5.3, 2.2 Hz, 1H), 1.70 – 1.55 (m, 2H), 1.26 (s, 3H), 1.09 (s, 3H), 0.98 (d, *J* = 6.6 Hz, 3H).

^{13}C NMR (101 MHz, $CDCl_3$): δ 151.0 (d, *J* = 242.1 Hz), 145.0 (d, *J* = 11.1 Hz), 139.3 (d, *J* = 4.7 Hz), 131.6 (d, *J* = 3.3 Hz), 114.3 (d, *J* = 17.7 Hz), 113.3 (d, *J* = 1.9 Hz), 56.3, 38.9, 36.9, 30.1, 29.1, 27.4, 25.9, 16.6.

^{19}F NMR (376 MHz, $CDCl_3$) δ –139.3.

IR (ATR, neat): $\tilde{\nu}$ = 2964 (m), 2930 (m), 2877 (w), 1510 (s), 1463 (m), 1405 (w), 1363 (w),1328 (m), 1264 (s), 1237 (w), 1207 (w), 1191 (w), 1174 (m), 1156 (w), 1146 (w), 1134 (w), 1070 (m), 872 (w), 826 (m), 770 (w) cm^{-1}.

HRMS (ESI): calcd for $C_{14}H_{20}FO^+$ $[M+H]^+$: 223.1493; found: 223.1492.

2.2.8 Tetralin **9**

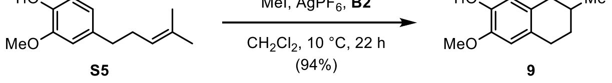

According to GP2, alkene **S5** (42.3 mg, 125 µmol, 1 equiv) was converted at 10 °C over 22 h to tetralin **9**. Purification by flash column chromatography on silica gel (10% dichloromethane in *n*-pentane) afforded tetralin **9** (41.2 mg, 117 µmol, 94%) as a colorless oil.

Analytical data of tetralin **9**:

TLC (20% dichloromethane in *n*-pentane): R_f = 0.45 (UV, CAM).

^{1}H NMR (400 MHz, $CDCl_3$): δ 7.14 (s, 1H), 6.66 (s, 1H), 3.86 (s, 3H), 2.78 (dd, *J* = 7.6, 5.2 Hz, 2H), 1.78 (dtd, *J* = 13.0, 5.2, 2.2 Hz, 1H), 1.72 – 1.54 (m, 2H), 1.27 (s, 3H), 1.10 (s, 3H), 0.99 (d, *J* = 6.6 Hz, 3H).

^{13}C NMR (101 MHz, $CDCl_3$): δ 148.5, 139.6, 137.3, 137.2, 120.9, 119.0 (q, *J* = 320.6 Hz), 113.0, 56.2, 38.7, 37.0, 29.9, 29.4, 27.1, 25.9, 16.5.

^{19}F NMR (376 MHz, $CDCl_3$) δ –73.8.

IR (ATR, neat): $\tilde{\nu}$ = 2967 (w), 1619 (w), 1506 (m), 1420 (s), 1366 (w), 1318 (m), 1267 (m), 1248 (m), 1203 (s), 1141 (s), 1123 (s), 1069 (m), 1032 (w), 986 (w), 949 (w), 910 (w), 870 (s), 807 (w), 749 (w), 685 (w), 654 (w), 625 (m), 604 (m), 509 (w), 490 (w) cm^{-1}.

HRMS (ESI): calcd for $C_{15}H_{19}F_3O_4SNa^+$ $[M+Na]^+$: 375.0848; found: 375.0845.

2.2.9 1,3-Dioxole **10**

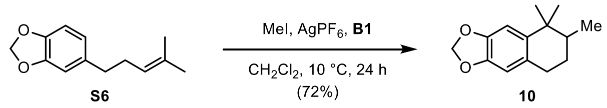

According to GP1, alkene **S6** (25.5 mg, 125 µmol, 1 equiv) was converted at 10 °C over 24 h to 1,3-dioxole **10**. Purification by flash column chromatography on silica gel (10% dichloromethane in *n*-pentane) afforded 1,3-dioxole **10** (19.7 mg, 90.3 µmol, 72%) as a colorless oil.

Analytical data of 1,3-dioxole **10**:

TLC (20% dichloromethane in *n*-pentane): R_f = 0.44 (UV, CAM).

^{1}H NMR (400 MHz, $CDCl_3$): δ 6.84 (s, 1H), 6.49 (s, 1H), 5.87 (s, 2H), 2.76 – 2.65 (m, 2H), 1.78 – 1.70 (m, 1H), 1.69 – 1.62 (m, 1H), 1.61 – 1.51 (m, 1H), 1.27 (s, 3H), 1.10 (s, 3H), 0.98 (d, *J* = 6.7 Hz, 3H).

^{13}C NMR (101 MHz, $CDCl_3$): δ 146.0, 145.2, 139.6, 129.0, 108.3, 106.9, 100.7, 39.2, 37.4, 30.3, 29.5, 27.5, 26.0, 16.7.

IR (ATR, neat): $\tilde{\nu}$ = 2965 (m), 2921 (m), 2877 (w), 1503 (m), 1483 (s), 1372 (m), 1237 (s), 1208 (w), 1190 (m), 1040 (s), 941 (m), 863 (m), 842 (m) cm^{-1}.

HRMS (ESI): calcd for $C_{14}H_{19}O_2^+$ $[M+H]^+$: 219.1380; found: 219.1380.

2.2.10 Tetralin **11**

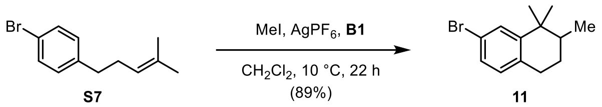

According to GP1, alkene **S7** (29.9 mg, 125 µmol, 1 equiv) was converted at 10 °C over 22 h to tetralin **11**. Purification by flash column chromatography on silica gel (1% formic acid in *n*-pentane) afforded tetralin **11** (28.1 mg, 111 µmol, 89%) as a colorless oil.

<u>Analytical data of tetralin **11**:</u>

TLC (*n*-pentane): R_f = 0.84 (UV, CAM).

^{1}H NMR (400 MHz, $CDCl_3$): δ 7.47 (d, *J* = 2.1 Hz, 1H), 7.18 (dd, *J* = 8.2, 2.1 Hz, 1H), 6.91 (d, *J* = 8.2 Hz, 1H), 2.78 – 2.69 (m, 2H), 1.78 (dtd, *J* = 13.3, 5.4, 2.5 Hz, 1H), 1.73 – 1.53 (m, 2H), 1.30 (s, 3H), 1.14 (s, 3H), 0.99 (d, *J* = 6.7 Hz, 3H).

^{13}C NMR (101 MHz, $CDCl_3$): δ 148.9, 134.9, 130.8, 130.1, 128.3, 119.6, 38.9, 37.5, 30.1, 28.7, 27.1, 25.9, 16.5.

IR (ATR, neat): $\tilde{\nu}$ = 2965 (s), 2926 (m), 2875 (m), 1588 (m), 1564 (w), 1484 (s), 1461 (s), 1432 (m), 1393 (m), 1375 (m), 1364 (m), 1271 (w), 1236 (m), 1206 (w), 1082 (m), 921 (m), 878 (m), 841 (m), 827 (s), 805 (s), 780 (w), 631 (w), 458 (m) cm^{-1}.

HRMS (ESI): calcd for $C_{13}H_{18}{}^{79}Br^+$ $[M+H]^+$: 253.0586; found: 253.0581.

2.2.11 Tetralin **12**

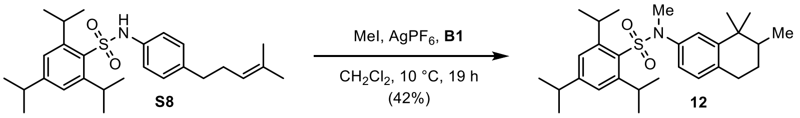

According to GP1, alkene **S8** (55.2 mg, 125 µmol, 1 equiv) was converted with increased equivalents of methyl iodide (42.5 µL, 656 µmol, 5.25 equiv), silver hexafluorophosphate (111 mg, 438 µmol, 3.50 equiv), and 2,4,6-tri-*tert*-butylpyrimidine (**B1**) (109 mg, 438 µmol, 3.50 equiv) at 10 °C over 19 h to tetralin **12**. Purification by flash column chromatography on silica gel (5% diethyl ether in *n*-pentane) afforded tetralin **12** (24.6 mg, 52.4 µmol, 42%) as a colorless oil.

<u>Analytical data of tetralin **12**:</u>

TLC (2% ethyl acetate in *n*-pentane): R_f = 0.32 (UV, CAM).

^{1}H NMR (400 MHz, $CDCl_3$): δ 7.09 (s, 2H), 7.07 (dd, *J* = 8.4, 2.4 Hz, 1H), 6.99 (d, *J* = 8.2 Hz, 1H), 6.90 (d, *J* = 2.2 Hz, 1H), 3.94 (hept, *J* = 6.7 Hz, 2H), 3.28 (s, 3H), 2.86 (hept, *J* = 6.9 Hz, 1H), 2.78 – 2.64 (m, 2H), 1.71 (dtd, *J* = 13.0, 5.1, 2.1 Hz, 1H), 1.64 – 1.44 (m, 2H), 1.22 (d, *J* = 6.9 Hz, 6H), 1.11 (d, *J* = 6.8 Hz, 6H), 1.11 (d, *J* = 6.7 Hz, 6H), 1.02 (s, 3H), 0.92 (d, *J* = 6.6 Hz, 3H), 0.86 (s, 3H).

^{13}C NMR (101 MHz, $CDCl_3$): δ 153.1, 151.6 (2C), 147.2, 139.0, 135.8, 131.6, 129.8, 128.3, 125.1, 123.8 (2C), 38.9, 37.5, 37.2, 34.3, 29.8, 29.6 (2C), 29.0, 27.2, 25.7, 24.9 (2C), 24.9 (2C), 23.8, 23.7, 16.5.

IR (ATR, neat): $\tilde{\nu}$ = 2960 (s), 2928 (m), 2868 (m), 1600 (m), 1566 (w), 1494 (m), 1461 (m), 1425 (m), 1383 (m), 1363 (m), 1316 (s), 1278 (w), 1257 (w), 1163 (s), 1147 (s), 1105 (w), 1071 (m), 1057 (m), 936 (m), 904 (s), 883 (m), 843 (m), 819 (m), 798 (m), 783 (m), 757 (w), 733 (s), 692 (s), 676 (m), 652 (s), 614 (m), 582 (m), 560 (s), 534 (m) cm^{-1}.

HRMS (ESI): calcd for $C_{29}H_{43}NO_2SNa^+$ $[M+Na]^+$: 492.2907; found: 492.2892.

2.2.12 Tetrahydroanthracenes **13**

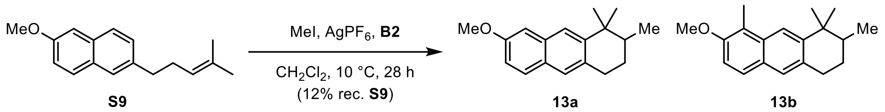

S9 **13a** (42%) **13b** (24%)

According to GP2, alkene **S9** (30.0 mg, 125 µmol, 1 equiv) was converted at 10 °C over 28 h to tetrahydroanthracene **13**. Purification by flash column chromatography on silica gel (100% dichloromethane) followed by semipreparative normal-phase high performance liquid chromatography (HPLC) (0.01% to 0.5% ethyl acetate in *n*-hexane over 40 min) afforded tetrahydroanthracene **13a** (13.3 mg, 52.3 µmol, 42%) as a colorless oil, tetrahydroanthracene **13b** (8.0 mg, 30 µmol, 24%) as a colorless oil, and alkene **S9** (3.6 mg, 15 µmol, 12%) as a colorless oil.

For GP1 (48 h at 10 °C; purification as described above):
Tetrahydroanthracene **13a** (9.4 mg, 35 µmol, 28%)
Tetrahydroanthracene **13b** (11.6 mg, 43.3 µmol, 35%)
Alkene **S9** (3.6 mg, 15 µmol, 12%)

<u>Analytical data of tetrahydroanthracene **13a**:</u>

TLC (2% ethyl acetate in cyclohexane): R_f = 0.32 (UV, CAM).

^{1}H NMR (400 MHz, $CDCl_3$): δ 7.73 (s, 1H), 7.59 (d, *J* = 8.8 Hz, 1H), 7.45 (s, 1H), 7.06 (d, *J* = 2.5 Hz, 1H), 7.03 (dd, *J* = 8.8, 2.5 Hz, 1H), 3.91 (s, 3H), 3.05 – 2.91 (m, 2H), 1.93 – 1.84 (m, 1H), 1.84 – 1.75 (m, 1H), 1.72 – 1.61 (m, 1H), 1.42 (s, 3H), 1.26 (s, 3H), 1.03 (d, *J* = 6.8 Hz, 3H).

^{13}C NMR (101 MHz, $CDCl_3$): δ 157.0, 146.3, 133.4, 132.7, 128.4, 127.5, 126.6, 124.4, 118.4, 105.2, 55.4, 39.4, 37.7, 30.8, 29.1, 27.5, 26.6, 16.7.

IR (ATR, neat): $\tilde{\nu}$ = 2964 (m), 2927 (m), 2875 (m), 1634 (m), 1604 (m), 1502 (m), 1464 (m), 1439 (m), 1398 (m), 1256 (m), 1226 (s), 1207 (m), 1172 (m), 1127 (m), 1035 (m) , 886 (w), 807 (w) cm^{-1}.

HRMS (ESI): calcd for $C_{18}H_{23}O^+$ $[M+H]^+$: 255.1743; found: 255.1743.

<u>Analytical data of tetrahydroanthracene **13b**:</u>

TLC (2% ethyl acetate in cyclohexane): R_f = 0.36 (UV, CAM).

^{1}H NMR (400 MHz, $CDCl_3$): δ 7.93 (s, 1H), 7.58 (d, *J* = 8.9 Hz, 1H), 7.46 (s, 1H), 7.18 (d, *J* = 9.0 Hz, 1H), 3.92 (s, 3H), 3.06 – 2.92 (m, 2H), 2.55 (s, 3H), 1.94 – 1.86 (m, 1H), 1.84 – 1.76 (m, 1H), 1.73 – 1.63 (m, 1H), 1.45 (s, 3H), 1.29 (s, 3H), 1.04 (d, *J* = 6.8 Hz, 3H).

^{13}C NMR (101 MHz, $CDCl_3$): δ 153.8, 146.0, 132.5, 132.4, 127.7, 127.1, 126.1, 121.1, 118.9, 114.0, 57.2, 39.5, 38.0, 31.0, 28.9, 27.5, 26.8, 16.8, 10.7.

IR (ATR, neat): $\tilde{\nu}$ = 2962 (s), 2926 (s), 2972 (m), 2860 (m), 1630 (w), 1598 (w), 1496 (m), 1462 (m), 1374 (w), 1317 (w), 1252 (s), 1174 (m), 1111 (s), 1041 (w), 1017 (w), 998 (w), 878 (w), 797 (w) cm^{-1}.

HRMS (ESI): calcd for $C_{19}H_{25}O^+$ $[M+H]^+$: 269.1900; found: 269.1899.

2.2.13 Spirocycle **14**

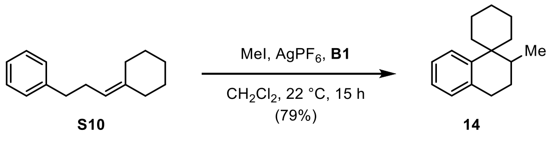

According to GP1, alkene **S10** (25.0 mg, 125 µmol, 1 equiv) was converted at 23 °C over 15 h to spirocycle **14**. Purification by flash column chromatography on silica gel (100% *n*-pentane) followed by normal-phase high performance liquid chromatography (HPLC) (0.01% to 0.5% ethyl acetate in *n*-hexane over 40 min) afforded spirocycle **14** (21.1 mg, 98.3 µmol, 79%) as a colorless oil.

Analytical data of spirocycle **14**:

TLC (100% *n*-pentane): R_f = 0.70 (UV, CAM).

^{1}H NMR (400 MHz, $CDCl_3$): δ 7.41 (d, *J* = 7.8 Hz, 1H), 7.19 – 7.13 (m, 1H), 7.11 – 7.03 (m, 2H), 2.92 (ddd, *J* = 17.4, 12.7, 6.8 Hz, 1H), 2.68 (dd, *J* = 17.3, 6.2 Hz, 1H), 2.45 (qt, *J* = 7.0, 3.5 Hz, 1H), 2.08 (tdd, *J* = 13.0, 6.6, 3.5 Hz, 1H), 1.97 – 1.88 (m, 2H), 1.80 – 1.71 (m, 2H), 1.69 – 1.48 (m, 5H), 1.33 (tdt, *J* = 12.9, 10.2, 5.2 Hz, 2H), 0.89 (d, *J* = 7.0 Hz, 3H).

^{13}C NMR (101 MHz, $CDCl_3$): δ 145.5, 136.0, 129.1, 126.5, 125.9, 125.1, 40.3, 39.9, 33.3, 27.4, 26.3, 25.0, 24.8, 22.2, 21.7, 14.4.

IR (ATR, neat): $\tilde{\nu}$ = 3061 (w), 3013 (2), 2919 (s), 2863 (s), 1488 (m), 1450 (s), 1377 (w), 1350 (w), 1214 (w),1051 (w), 1037 (w), 993 (w), 900 (w), 875 (w), 825 (w), 812 (w), 751 (s), 726 (m), 455 (w) cm^{-1}.

HRMS (ESI): Mass could not be found due to insufficient ionization with electrospray ionization (ESI). However, after benzylic oxidation to ketone **S150** (chapter 2.5.5) high resolution mass spectrometry confirmed the molecular formula.

2.2.14 Spirocycles **15**

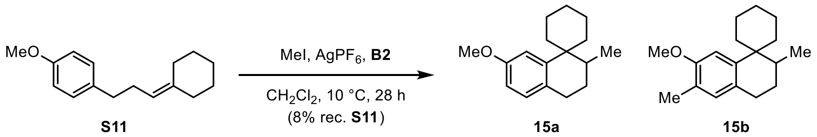

S11 **15a** (58%) **15b** (13%)

According to GP2, alkene **S11** (28.8 mg, 125 µmol, 1 equiv) was converted at 10 °C over 28 h to spirocycle **15a** and **15b**. Purification by flash column chromatography on silica gel (10% dichloromethane in *n*-pentane) followed by semipreparative normal-phase high performance liquid chromatography (HPLC) (0.1% to 1.8% ethyl acetate in *n*-hexane over 40 min) afforded spirocycle **15a** (17.2 mg, 72.5 µmol, 58%) as a colorless oil as well as an impure mixture of spirocycle **15b** and alkene **S11**. Further purification via semipreparative normal-phase high performance liquid chromatography (HPLC) (0.1% to 1.0% ethyl acetate in *n*-hexane over 40 min) afforded spirocycle **15b** (4.1 mg, 32 µmol, 13%) as a colorless oil and alkene **S11** (2.4 mg, 10 µmol, 8%) as a colorless oil.

For GP1 (46 h at 10 °C; purification as described above):
Spirocycle **15a** (15.5 mg, 63.4 µmol, 51%)
Spirocycle **15b** (6.5 mg, 25 µmol, 20%)
Alkene **S11** (1.0 mg, 4.3 µmol, 4%)

Analytical data of spirocycle **15a**:

TLC (10% dichloromethane in *n*-pentane): R_f = 0.23 (UV, CAM).

^{1}H NMR (400 MHz, $CDCl_3$): δ 6.98 (d, *J* = 8.7 Hz, 1H), 6.97 (d, *J* = 3.0 Hz, 1H), 6.67 (dd, *J* = 8.3, 2.7 Hz, 1H), 3.80 (s, 3H), 2.84 (ddd, *J* = 16.7, 12.6, 6.7 Hz, 1H), 2.63 (ddd, *J* = 17.0, 7.1, 1.6 Hz, 1H), 2.43 (qt, *J* = 7.0, 3.5 Hz, 1H), 2.05 (tdd, *J* = 13.0, 6.6, 3.5 Hz, 1H), 1.94 – 1.83 (m, 2H), 1.80 – 1.70 (m, 2H), 1.68 – 1.47 (m, 5H), 1.41 – 1.28 (m, 2H), 0.88 (d, *J* = 7.0 Hz, 3H).

^{13}C NMR (101 MHz, $CDCl_3$): δ 157.9, 146.9, 129.9, 128.1, 112.3, 110.6, 55.3, 40.2, 40.1, 33.4, 27.3, 26.3, 24.9, 24.1, 22.2, 21.7, 14.3.

IR (ATR, neat): $\tilde{v}$ = 2921 (s), 2862 (m), 1608 (m), 1574 (w), 1499 (m), 1451 (m), 1287 (m), 1273 (m), 1239 (s), 1210 (m), 1130 (w), 1050 (m), 863 (w), 844 (w), 808 (m) cm^{-1}.

HRMS (ESI): calcd for $C_{17}H_{25}O^+$ $[M+H]^+$: 245.1900; found: 245.1901.

Analytical data of spirocycle **15b**:

TLC (1% ethyl acetate in cyclohexane): R_f = 0.20 (UV, CAM).

^{1}H NMR (400 MHz, $CDCl_3$): δ 6.85 (s, 1H), 6.82 (s, 1H), 3.82 (s, 3H), 2.80 (ddd, *J* = 19.0, 12.7, 6.6 Hz, 1H), 2.56 (dd, *J* = 17.0, 6.3 Hz, 1H), 2.40 (tq, *J* = 7.0, 3.5 Hz, 1H), 2.16 (s, 3H), 2.02 (tdd, *J* = 12.9, 6.5, 3.4 Hz, 1H), 1.95 – 1.81 (m, 2H), 1.80 – 1.71 (m, 2H), 1.68 – 1.46 (m, 5H), 1.39 – 1.28 (m, 2H), 0.88 (d, *J* = 7.0 Hz, 3H).

^{13}C NMR (101 MHz, $CDCl_3$): δ 156.2, 143.8, 131.2, 127.6, 123.8, 108.2, 55.6, 40.2, 40.0, 33.7, 27.3, 26.3, 25.0, 24.1, 22.3, 21.8, 15.8, 14.3.

IR (ATR, neat): $\tilde{v}$ = 2933 (s), 2863 (m), 1511 (m), 1463 (m), 1452 (m), 1327 (m), 1247 (s), 1211 (m), 1113 (m), 1044 (w), 877 (w), 772 (w) cm^{-1}.

HRMS (ESI): calcd for $C_{18}H_{27}O^+$ $[M+H]^+$: 259.2056; found: 259.2055.

2.2.15 Spirocycles **16**

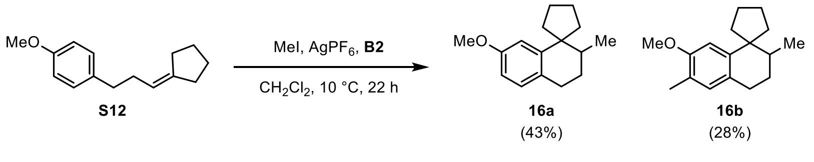

According to GP2, alkene **S12** (27.1 mg, 125 µmol, 1 equiv) was converted at 10 °C over 22 h to spirocycle **16a** and **16b**. Purification by flash column chromatography on silica gel (10%

dichloromethane in *n*-pentane) followed by semipreparative normal-phase high performance liquid chromatography (HPLC) (0.01% to 0.5% ethyl acetate in *n*-hexane over 40 min) afforded spirocycle **16a** (12.3 mg, 53.4 µmol, 43%) as a colorless oil and spirocycle **16b** (8.6 mg, 35 µmol, 28%) as a colorless oil.

Analytical data of spirocycle **16a**:

TLC (10% dichloromethane in *n*-pentane): R_f = 0.25 (UV, CAM).

^{1}H NMR (400 MHz, $CDCl_3$): δ 6.95 (d, *J* = 8.4 Hz, 1H), 6.77 (d, *J* = 2.7 Hz, 1H), 6.66 (dd, *J* = 8.4, 2.7 Hz, 1H), 3.78 (s, 3H), 2.79 (dt, *J* = 16.9, 7.3 Hz, 1H), 2.70 (dt, *J* = 16.8, 6.2 Hz, 1H), 2.00 – 1.71 (m, 10H), 1.61 (dq, *J* = 12.6, 6.2 Hz, 1H), 0.92 (d, *J* = 6.9 Hz, 3H).

^{13}C NMR (101 MHz, $CDCl_3$): δ 157.8, 148.2, 129.5, 127.9, 113.1, 110.5, 55.3, 50.2, 43.1, 38.4, 37.9, 27.9, 27.1, 26.5, 26.1, 16.3.

IR (ATR, neat): $\tilde{\nu}$ = 2953 (s), 2930 (s), 2871 (m), 1610 (m), 1575 (w), 1501 (s), 1465 (w), 1291 (w), 1277 (w), 1240 (s), 1215 (w), 1051 (w), 798 (w) cm^{-1}

HRMS (ESI): calcd for $C_{16}H_{23}O^+$ $[M+H]^+$: 231.1743; found: 231.1737.

Analytical data of spirocycle **16b**:

TLC (10% dichloromethane in *n*-pentane): R_f = 0.37 (UV, CAM).

^{1}H NMR (400 MHz, $CDCl_3$): δ 6.79 (s, 1H), 6.68 (s, 1H), 3.80 (s, 3H), 2.75 (ddd, *J* = 16.8, 8.4, 6.6 Hz, 1H), 2.65 (dt, *J* = 16.8, 6.2 Hz, 1H), 2.15 (s, 3H), 1.97 – 1.71 (m, 10H), 1.60 (dq, *J* = 12.7, 6.2 Hz, 1H), 0.92 (d, *J* = 6.9 Hz, 3H).

^{13}C NMR (101 MHz, $CDCl_3$): δ 156.1, 145.1, 130.9, 127.3, 123.7, 109.0, 55.5, 49.9, 43.2, 38.3, 38.0, 28.0, 27.2, 26.6, 26.1, 16.2, 15.8.

HRMS (ESI): calcd for $C_{17}H_{25}O^+$ $[M+H]^+$: 245.1900; found: 245.1894.

2.2.16 Piperidine **17**

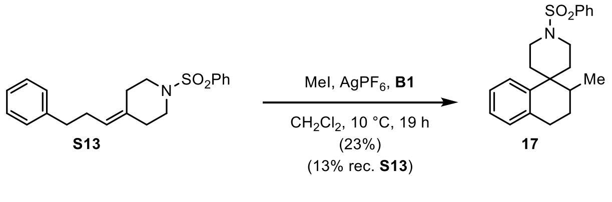

According to GP1, alkene **S13** (42.7 mg, 125 µmol, 1 equiv) was converted at 10 °C over 19 h to piperidine **17**. Purification by flash column chromatography on silica gel (50% dichloromethane in *n*-pentane) followed by semipreparative normal-phase high performance

liquid chromatography (HPLC) (3.0% to 10.0% ethyl acetate in *n*-hexane over 40 min) afforded piperidine **17** (10.2 mg, 23.0 µmol, 23%) as a white solid and alkene **S13** (5.7 mg, 8.3 µmol, 13%) as a colorless oil.

For GP2 (24 h at 10 °C; analysis via NMR standard):
Piperidine **17** (8.2 mg, 23 µmol, 18% NMR yield)
Alkene **S13** (2.8 mg, 6.6 µmol, 6% NMR yield)

Analytical data of piperidine **17**:

TLC (10% ethyl acetate in cyclohexane): R_f = 0.30 (UV, $KMnO_4$).

^{1}H NMR (400 MHz, $CDCl_3$): δ 7.82 (dd, *J* = 7.2, 1.8 Hz, 2H), 7.67 – 7.61 (m, 1H), 7.60 – 7.55 (m, 2H), 7.29 (d, *J* = 7.9 Hz, 1H), 7.16 (t, *J* = 7.5 Hz, 1H), 7.10 (t, *J* = 7.1 Hz, 1H), 7.04 (d, *J* = 7.5 Hz, 1H), 3.83 – 3.75 (m, 1H), 3.67 – 3.59 (m, 1H), 2.87 (ddd, *J* = 18.6, 12.6, 6.5 Hz, 1H), 2.63 (dddd, *J* = 23.8, 14.4, 12.3, 2.6 Hz, 3H), 2.33 (td, *J* = 13.2, 12.8, 4.2 Hz, 1H), 2.07 (qt, *J* = 6.9, 3.4 Hz, 1H), 1.98 – 1.85 (m, 2H), 1.85 – 1.71 (m, 2H), 1.56 – 1.48 (m, 1H), 0.79 (d, *J* = 7.0 Hz, 3H).

^{13}C NMR (101 MHz, $CDCl_3$): δ 142.5, 136.8, 136.1, 132.8, 129.4, 129.2 (2C), 127.8 (2C), 126.5, 126.4, 125.9, 42.3 (2C), 38.1, 37.7, 32.0, 26.9, 24.8, 24.7, 14.5.

IR (ATR, neat): $\tilde{\nu}$ = 3061 (w), 2927 (m), 2855 (w), 1489 (w), 1471 (w), 1445 (m), 1379 (w),1351 (m), 1342 (m), 1322 (m), 1289 (w), 1272 (w), 1250 (w), 1205 (w), 1168 (s), 1126 (w), 1093 (m), 1073 (w), 1060 (m), 1036 (w), 999 (w), 962 (w), 941 (m), 924 (w), 908 (w), 757 (m), 736 (s), 692 (m), 621 (w), 577 (s) cm^{-1}.

HRMS (ESI): calcd for $C_{21}H_{25}NO_2SNa^+$ $[M+Na]^+$: 378.1498; found: 378.1489.

mp: 154–155 °C.

2.2.17 Cyclobutane **21**

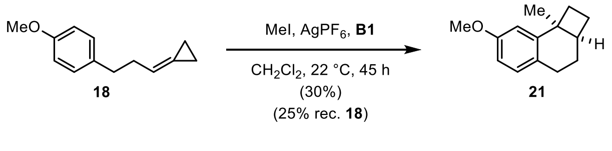

According to GP1, alkene **18** (23.6 mg, 125 µmol, 1 equiv) was converted at 23 °C over 45 h to cyclobutane **21**. Purification by flash column chromatography on silica gel (10% to 100% dichloromethane in *n*-pentane) followed by semipreparative normal-phase high performance liquid chromatography (HPLC) (0.5% to 3.5% ethyl acetate in *n*-hexane over 40 min) afforded

cyclobutane **21** (7.6 mg, 38 µmol, 30%) as a colorless oil and alkene **18** (5.8 mg, 31 µmol, 25%) as a colorless oil.

For GP2 (20 h at 10 °C, then 30 h at 23 °C; purification as described above):
Cyclobutane **21** (7.3 mg, 36 µmol, 29%)
Alkene **18** (5.2 mg, 28 µmol, 22%)

Analytical data of cyclobutane **21**:

TLC (10% dichloromethane in *n*-pentane): R_f = 0.39 (UV, CAM).

^{1}H NMR (400 MHz, $CDCl_3$): δ 7.01 (d, *J* = 8.3 Hz, 1H), 6.70 (d, *J* = 2.7 Hz, 1H), 6.66 (dd, *J* = 8.3, 2.7 Hz, 1H), 3.78 (s, 3H), 2.70 (ddd, *J* = 15.3, 7.4, 4.2 Hz, 1H), 2.54 (ddd, *J* = 15.2, 8.7, 4.1 Hz, 1H), 2.33 – 2.25 (m, 1H), 2.20 – 2.04 (m, 3H), 1.94 – 1.85 (m, 1H), 1.66 – 1.55 (m, 2H), 1.45 (s, 3H).

^{13}C NMR (101 MHz, $CDCl_3$): δ 158.4, 147.1, 130.0, 129.2, 111.6, 110.8, 55.4, 41.4, 39.8, 35.1, 29.9, 28.0, 27.6, 20.8.

IR (ATR, neat): $\tilde{\nu}$ = 2924 (s), 2854 (s), 1624 (m), 1570 (w), 1500 (s), 1455 (m), 1292 (m), 1234 (s), 1210 (w), 1196 (2C), 1044 (m), 802 (w) cm^{-1}.

HRMS (ESI): calcd for $C_{14}H_{19}O^+$ [M+H]$^+$: 203.1430; found: 203.1430.

2.2.18 Spirocycles **23** and cyclopentane **23c**

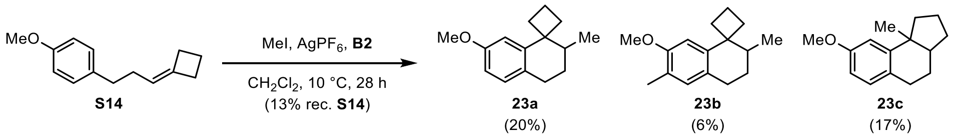

According to GP1, alkene **S14** (25.3 mg, 125 µmol, 1 equiv) was converted at 10 °C over 28 h to spirocycle **23a**, spirocycle **23b**, and cyclopentane **23c**. Purification by flash column chromatography on silica gel (10% dichloromethane in *n*-pentane) followed by semipreparative normal-phase high performance liquid chromatography (HPLC) (0.01% to 0.5% ethyl acetate in *n*-hexane over 40 min) afforded spirocycle **23a** (5.5 mg, 26 µmol, 20%) as a colorless oil, spirocycle **23b** (1.8 mg, 7.9 µmol, 6%) as a colorless oil, cyclopentane **23c** (4.6 mg, 21 µmol, 17%) as a colorless oil, and alkene **S14** (3.4 mg, 17 µmol, 13%) as a colorless oil.

Analytical data of spirocycle **23a**:

TLC (10% dichloromethane in *n*-pentane): R_f = 0.28 (UV, CAM).

^{1}H NMR (400 MHz, $CDCl_3$): δ 7.18 (d, *J* = 2.7 Hz, 1H), 6.96 (d, *J* = 8.3 Hz, 1H), 6.69 (dd, *J* = 8.3, 2.7 Hz, 1H), 3.83 (s, 3H), 2.76 (ddd, *J* = 16.1, 9.6, 6.2 Hz, 1H), 2.62 (dt, *J* = 16.8, 5.7 Hz, 1H), 2.38 – 2.28 (m, 1H), 2.23 – 2.04 (m, 4H), 2.03 – 1.91 (m, 2H), 1.90 – 1.82 (m, 1H), 1.56 – 1.47 (m, 1H), 0.91 (d, *J* = 6.9 Hz, 3H).

^{13}C NMR (101 MHz, $CDCl_3$) δ 158.2, 145.8, 129.6, 127.7, 112.3, 110.8, 55.4, 45.7, 37.9, 36.8, 30.4, 25.7, 25.5, 14.9, 14.3.

IR (ATR, neat): $\tilde{\nu}$ = 2957 (m), 2925 (s), 2855 (w), 1610 (w), 1575 (w), 1501 (s), 1464 (w), 1290 (w), 1240 (s), 1052 (w), 798 (w) cm^{-1}.

HRMS (ESI): calcd for $C_{15}H_{21}O^+$ $[M+H]^+$: 217.1587; found: 217.1581.

<u>Analytical data of spirocycle **23b**:</u>

TLC (10% dichloromethane in *n*-pentane): R_f = 0.35 (UV, CAM).

^{1}H NMR (700 MHz, $CDCl_3$) δ 7.08 (s, 1H), 6.80 (s, 1H), 3.88 (s, 3H), 2.72 (ddd, *J* = 16.2, 9.7, 6.2 Hz, 1H), 2.58 (dt, *J* = 16.7, 5.6 Hz, 1H), 2.32 (td, *J* = 10.9, 10.4, 6.3 Hz, 1H), 2.21 (td, *J* = 11.8, 11.1, 7.0 Hz, 1H), 2.17 (s, 3H), 2.16 – 2.10 (m, 2H), 2.09 – 2.03 (m, 1H), 2.02 – 1.98 (m, 1H), 1.98 – 1.92 (m, 1H), 1.84 (dddd, *J* = 13.0, 9.6, 6.4, 3.2 Hz, 1H), 1.53 – 1.49 (m, 1H), 0.92 (dd, *J* = 7.0, 1.2 Hz, 3H).

^{13}C NMR (176 MHz, $CDCl_3$) δ 156.4, 142.7, 131.0, 127.0, 124.1, 108.2, 55.6, 45.6, 37.9, 36.9, 30.5, 25.7, 25.6, 15.8, 15.1, 14.3.

IR (ATR, neat): $\tilde{\nu}$ = 2957 (m), 2924 (s), 2854 (w), 1514 (w), 1498 (m), 1464 (w), 1339 (w), 1250 (m), 1210 (m), 1116 (w), 1045 (w) cm^{-1}.

HRMS (ESI): calcd for $C_{16}H_{23}O^+$ $[M+H]^+$: 231.1743; found: 231.1736.

<u>Analytical data of cyclopentane **23c**:</u>[1]

TLC (10% dichloromethane in *n*-pentane): R_f = 0.28 (UV, CAM).

^{1}H NMR (400 MHz, $CDCl_3$): δ 6.97 (d, *J* = 8.3 Hz, 1H), 6.82 (d, *J* = 2.7 Hz, 1H), 6.64 (dd, *J* = 8.3, 2.7 Hz, 1H), 3.79 (s, 3H), 2.73 – 2.55 (m, 2H), 2.03 – 1.94 (m, 1H), 1.93 – 1.77 (m, 4H), 1.71 – 1.55 (m, 2H), 1.52 – 1.39 (m, 2H), 1.27 (s, 3H).

^{13}C NMR (101 MHz, $CDCl_3$) δ 158.1, 147.4, 129.2, 129.1, 113.2, 110.6, 55.4, 45.9, 45.5, 42.1, 31.2, 31.1, 27.9, 27.4, 23.3.

---

[1]The stereochemistry (*cis*/*trans* at the ring junction) could not be unambiguously assigned due to signal overlap in the NOE spectra.

IR (ATR, neat): $\tilde{\nu}$ = 2953 (s), 2924 (s), 2854 (w), 1611 (w), 1501 (w), 1464 (w), 1281 (w), 1241 (w), 1043 (w) cm^{-1}.

HRMS (ESI): calcd for $C_{15}H_{21}O^+$ [M+H]$^+$: 217.1587; found: 217.1582.

2.2.19 Tetralin **26**

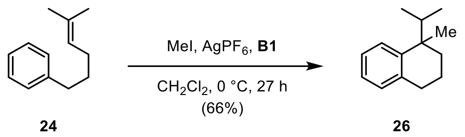

According to GP1, alkene **24** (21.8 mg, 125 µmol, 1 equiv) was converted at 0 °C over 27 h to tetralin **26**. Purification by flash column chromatography on silica gel (1% formic acid in 10% dichloromethane in *n*-pentane) afforded tetralin **26** (15.6 mg, 82.8 µmol, 66%) as a colorless oil.

<u>Analytical data of tetralin **26**:</u>

TLC (100% *n*-pentane): R_f = 0.85 (UV, CAM).

^{1}H NMR (400 MHz, $CDCl_3$) δ 7.28 – 7.25 (m, 1H), 7.16 – 7.10 (m, 1H), 7.08 – 7.00 (m, 2H), 2.76 – 2.65 (m, 2H), 2.15 (hept, *J* = 6.9 Hz, 1H), 1.91 – 1.80 (m, 1H), 1.77 – 1.63 (m, 2H), 1.54 – 1.48 (m, 1H), 1.27 (s, 3H), 0.97 (d, *J* = 6.8 Hz, 3H), 0.62 (d, *J* = 6.8 Hz, 3H).

^{13}C NMR (101 MHz, $CDCl_3$) δ 145.5, 137.5, 129.0, 126.9, 125.8, 124.9, 39.9, 37.3, 31.0, 30.4, 29.2, 19.8, 18.2, 17.2.

IR (ATR, neat): $\tilde{\nu}$ = 3060 (w), 3015 (w), 2961 (s), 2934 (s), 2866 (m), 2838 (w), 1602 (w), 1489 (m), 1461 (m), 1441 (m), 1374 (m), 1280 (w), 1224 (w), 1183 (w), 1157 (w), 1099 (w), 1044 (w), 1028 (w), 968 (w), 940 (w), 897 (w), 871 (w), 846 (w), 807 (w), 776 (w), 756 (s), 731 (s), 698 (w), 562 (w), 471 (w), 440 (w) cm^{-1}.

HRMS (ESI): Mass could not be found due to insufficient ionization with electrospray ionization (ESI). However, after benzylic oxidation to ketone **S151** (chapter 2.5.6) high resolution mass spectrometry confirmed the molecular formula.

2.2.20 Tetralins **27**

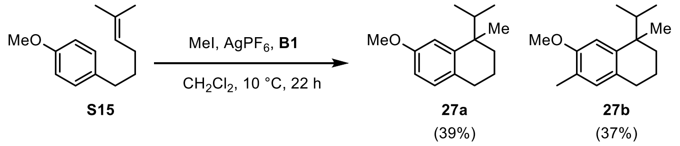

According to GP1, alkene **S15** (25.5 mg, 125 µmol, 1 equiv) was converted at 10 °C over 22 h to tetralin **27a**/**b**. Purification by flash column chromatography on silica gel (10% dichloromethane in *n*-pentane) followed by normal-phase high performance liquid chromatography (HPLC) (0.01% to 0.5% ethyl acetate in *n*-hexane over 40 min) afforded tetralin **27a** (10.6 mg, 48.4 µmol, 39%) as a colorless oil and tetralin **27b** (10.7 mg, 46.1 µmol, 37%) as a colorless oil.

For GP2 (24 h at 10 °C; purification as described above):
Tetralin **27a** (10.6 mg, 48.4 µmol, 39%)
Tetralin **27b** (8.1 mg, 35 µmol, 28%)

Analytical data of tetralin **27a**:

TLC (10% dichloromethane in *n*-pentane): R_f = 0.31 (UV, CAM).

^{1}H NMR (400 MHz, $CDCl_3$): δ 6.95 (dt, *J* = 8.4, 1.0 Hz, 1H), 6.81 (d, *J* = 2.7 Hz, 1H), 6.64 (dd, *J* = 8.4, 2.7 Hz, 1H), 3.78 (s, 3H), 2.69 – 2.56 (m, 2H), 2.11 (hept, *J* = 6.8 Hz, 1H), 1.88 – 1.78 (m, 1H), 1.72 – 1.62 (m, 2H), 1.51 – 1.45 (m, 1H), 1.25 (s, 3H), 0.96 (d, *J* = 6.8 Hz, 3H), 0.63 (d, *J* = 6.8 Hz, 3H).

^{13}C NMR (101 MHz, $CDCl_3$): δ 157.8, 146.8, 129.9, 129.7, 112.5, 110.5, 55.4, 40.2, 37.4, 30.2, 30.2, 29.3, 20.0, 18.3, 17.2.

IR (ATR, neat): $\tilde{\nu}$ = 2959 (m), 2933 (m), 2873 (w), 2833 (w), 1611 (m), 1503 (m), 1493 (m), 1462 (m), 1441 (w), 1374 (w), 1281 (m), 1237 (s), 1152 (w), 1051 (w), 797 (w) cm^{-1}.

HRMS (ESI): calcd for $C_{15}H_{23}O^+$ $[M+H]^+$: 219.1743; found: 219.1743.

Analytical data of tetralin **27b**:

TLC (10% dichloromethane in *n*-pentane): R_f = 0.46 (UV, CAM).

^{1}H NMR (400 MHz, $CDCl_3$): δ 6.79 (q, *J* = 0.9 Hz, 1H), 6.71 (s, 1H), 3.79 (s, 3H), 2.64 – 2.55 (m, 2H), 2.15 (s, 3H), 2.14 – 2.07 (m, 1H), 1.87 – 1.77 (m, 1H), 1.71 – 1.61 (m, 2H), 1.51 – 1.44 (m, 1H), 1.26 (s, 3H), 0.97 (d, *J* = 6.8 Hz, 3H), 0.63 (d, *J* = 6.8 Hz, 3H).

^{13}C NMR (101 MHz, $CDCl_3$): δ 156.1, 143.7, 131.1, 129.3, 123.6, 108.4, 55.6, 40.0, 37.5, 30.3, 30.1, 29.3, 20.0, 18.3, 17.2, 15.8.

IR (ATR, neat): $\tilde{\nu}$ = 2958 (s), 2931 (s), 2873 (m), 1617 (w), 1501 (s), 1463 (m), 1401 (w), 1373 (s), 1322 (s), 1249 (s), 1212 (s), 1136 (m), 1094 (w), 1050 (m), 1007 (w), 856 (w) cm^{-1}.

HRMS (ESI): calcd for $C_{16}H_{25}O^+$ $[M+H]^+$: 233.1900; found: 233.1898.

2.2.21 Tetralins **29**

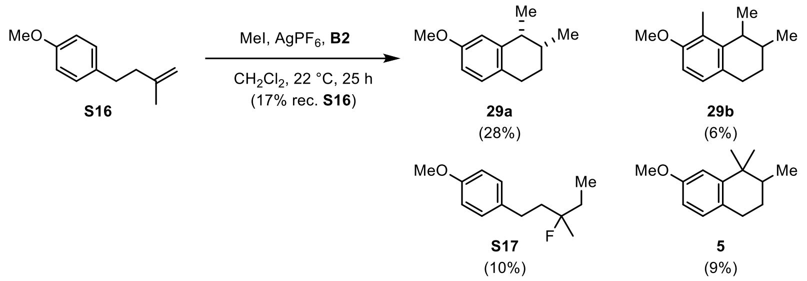

According to GP2, 1,1-disubstituted alkene **S16** (22.0 mg, 125 µmol, 1 equiv) was converted at 23 °C over 25 h to tetralin **29a**/**b**, fluoride **S17**, and tetralin **5**. Purification by flash column chromatography on silica gel (10% to 25% dichloromethane in *n*-pentane) afforded fluoride **S17** (2.6 mg, 12 µmol, 10%) as a colorless oil along with an impure product mixture. Further purification by semipreparative normal-phase high performance liquid chromatography (HPLC) (0.01% to 1.0% ethyl acetate in *n*-hexane over 40 min) afforded tetralin **29b** (1.6 mg, 7.8 µmol, 6%) as a colorless oil, recovered 1,1-disubstituted alkene **S16** (3.8 mg, 22 µmol, 17%) as a colorless oil, and an inseparable mixture of tetralin **29a** (6.7 mg, 35 µmol, 28%) and tetralin **5** (2.4 mg, 12 µmol, 9%) as a colorless oil.

For GP1 (24 h at 10 °C, then 23 h at 23 °C; purification as described above):
Tetralin **29a** (5.9 mg, 31 µmol, 25%)
Tetralin **29b** (2.1 mg, 10 µmol, 8%)
Fluoride **S17** (2.4 mg, 11 µmol, 9%)
Tetralin **5** (1.8 mg, 8.8 µmol, 7%)
1,1-Disubstituted alkene **S16** (3.0 mg, 17 µmol, 14%)

<u>Analytical data of tetralin **29a**:</u>

TLC (15% dichloromethane in *n*-pentane): R_f = 0.30 (UV, CAM).

^{1}H NMR (400 MHz, $CDCl_3$): δ 6.98 (d, J = 8.7 Hz, 1H), 6.68 (d, J = 7.1 Hz, 2H), 3.78 (s, 3H), 2.84 – 2.77 (m, 1H), 2.77 – 2.71 (m, 2H), 1.96 (hd, J = 6.9, 4.8 Hz, 1H), 1.66 – 1.59 (m, 2H), 1.12 (d, J = 7.2 Hz, 3H), 0.99 (d, J = 6.9 Hz, 3H).

^{13}C NMR (101 MHz, $CDCl_3$): δ 157.6, 144.6, 129.8, 128.3, 113.9, 111.8, 55.4, 38.2, 32.3, 28.3, 26.2, 18.5, 17.5.

IR (ATR, neat, mixture of **29a**/5 (3:1)): $\tilde{\nu}$ = 2957 (m), 2921 (m), 2871 (m), 2834 (w), 1610 (m), 1577 (w), 1501 (s), 1462 (m), 1369 (w), 1342 (w), 1298 (m), 1282 (m), 1260 (s), 1245 (s),

1218 (m), 1179 (w), 1153 (m), 1132 (w), 1073 (w), 1041 (s), 936 (w), 870 (m), 850 (m), 812 (m), 785 (w), 738 (w), 705 (w), 464 (w) cm^{-1}.

HRMS (ESI): calcd for $C_{13}H_{19}O^+$ $[M+H]^+$: 191.1430; found: 191.1431.

The obtained analytical data for tetralin **29a** were in accordance with reported literature values.[4]

Analytical data of tetralin **29b**:

TLC (20% dichloromethane in *n*-pentane): R_f = 0.52 (UV, CAM).

^{1}H NMR (400 MHz, $CDCl_3$): δ 6.91 (d, *J* = 8.4 Hz, 1H), 6.68 (d, *J* = 8.4 Hz, 1H), 3.80 (s, 3H), 2.98 – 2.89 (m, 1H), 2.85 – 2.76 (m, 2H), 2.18 (s, 3H), 1.95 – 1.82 (m, 1H), 1.70 – 1.55 (m, 2H), 1.05 (d, *J* = 6.9 Hz, 3H), 0.99 (d, *J* = 7.0 Hz, 3H).

^{13}C NMR (101 MHz, $CDCl_3$): δ 155.8, 143.4, 127.9, 127.0, 123.9, 108.4, 55.8, 35.3, 32.7, 29.4, 25.0, 19.8, 14.4, 10.9.

IR (ATR, neat): $\tilde{\nu}$ = 2956 (m), 2921 (m), 2869 (m), 2833 (w), 1598 (w), 1483 (s), 1464 (m), 1437 (w), 1261 (s), 1215 (w), 1111 (m), 1088 (w), 1054 (m), 797 (w), 720 (w), 510 (w) cm^{-1}.

HRMS (ESI): calcd for $C_{14}H_{21}O^+$ $[M+H]^+$: 205.1587; found: 205.1586.

Analytical data of fluoride **S17**:

TLC (20% dichloromethane in *n*-pentane): R_f = 0.22 (UV, CAM).

^{1}H NMR (400 MHz, $CDCl_3$): δ 7.12 (d, *J* = 8.6 Hz, 2H), 6.83 (d, *J* = 8.6 Hz, 2H), 3.79 (s, 3H), 2.64 (dd, *J* = 9.5, 7.8 Hz, 2H), 1.95 – 1.77 (m, 2H), 1.76 – 1.61 (m, 2H), 1.35 (d, *J* = 21.8 Hz, 3H), 0.95 (t, *J* = 7.5 Hz, 3H).

^{13}C NMR (101 MHz, $CDCl_3$): δ 158.0, 134.4, 129.3 (2C), 114.0 (2C), 97.6 (d, *J* = 167.6 Hz), 55.4, 41.5 (d, *J* = 23.1 Hz), 32.5 (d, *J* = 23.6 Hz), 29.2 (d, *J* = 5.5 Hz), 23.9 (d, *J* = 25.0 Hz), 8.2 (d, *J* = 7.1 Hz).

^{19}F NMR (376 MHz, $CDCl_3$) δ –146.5.

IR (ATR, neat): $\tilde{\nu}$ = 2975 (m), 2936 (m), 2834 (w), 1613 (w), 1584 (w), 1513 (s), 1465 (w), 1380 (w), 1300 (w), 1246 (s), 1178 (m), 1099 (w), 1038 (m), 877 (w), 822 (w), 529 (w) cm^{-1}.

HRMS (ESI): calcd for $C_{13}H_{19}FONa^+$ $[M+Na]^+$: 233.1312; found: 233.1310.

2.2.22 Tetralins **30**

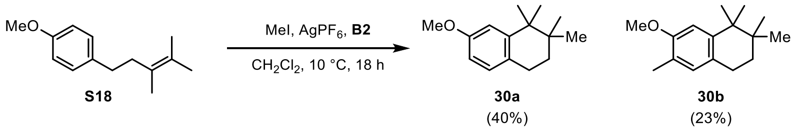

According to GP2, alkene **S18** (25.5 mg, 125 µmol, 1 equiv) was converted at 10 °C over 23 h to tetralin **30a** and tetralin **30b**. Purification by flash column chromatography on silica gel (2% diethyl ether in *n*-pentane) followed by semipreparative normal-phase high performance liquid chromatography (HPLC) (0.1% to 2.0% ethyl acetate in *n*-hexane) afforded tetralin **30a** (11.0 mg, 50.4 µmol, 40%) as a colorless oil and tetralin **30b** (6.7 mg, 29 µmol, 23%) as a colorless oil.

Using GP1 (23 h at 10 °C; purification as described above):
Tetralin **30a** (7.4 mg, 34 µmol, 27%)
Tetralin **30b** (5.5 mg, 24 µmol, 19%)

Analytical data of tetralin **30a**:

TLC (2% diethyl ether in *n*-pentane): R_f = 0.48 (UV, CAM).

^{1}H NMR (400 MHz, $CDCl_3$): δ 6.96 (d, *J* = 8.4 Hz, 1H), 6.91 (d, *J* = 2.7 Hz, 1H), 6.67 (dd, *J* = 8.3, 2.7 Hz, 1H), 3.79 (s, 3H), 2.74 (t, *J* = 6.4 Hz, 2H), 1.64 (t, *J* = 6.9 Hz, 2H), 1.22 (s, 6H), 0.93 (s, 6H).

^{13}C NMR (101 MHz, $CDCl_3$): δ 157.9, 148.1, 129.7, 127.4, 112.7, 110.7, 55.4, 40.2, 34.6, 33.4, 26.5 (2C), 25.8, 24.6 (2C).

IR (ATR, neat) $\tilde{\nu}$ = 3380 (w), 2953 (s), 2921 (s), 2852 (m), 1711 (w), 1618 (w), 1517 (w), 1501 (m), 1461 (m), 1402 (w), 1376 (m), 1364 (m), 1341 (w), 1320 (m), 1251 (m), 1209 (m), 1185 (m), 1158 (m), 1143 (m), 1115 (w), 1064 (m), 1001 (w), 986 (w), 941 (w), 877 (w), 848 (w), 829 (w), 791 (w), 490 (w).

HRMS (ESI): calcd for $C_{15}H_{23}O^+$ $[M+H]^+$: 219.1743; found: 219.1738

Analytical data of tetralin **30b**:

TLC (2% diethyl ether in *n*-pentane): R_f = 0.48 (UV, CAM).

^{1}H NMR (400 MHz, $CDCl_3$): δ 6.80 (s, 2H), 3.81 (s, 3H), 2.70 (t, *J* = 6.8 Hz, 2H), 2.16 (s, 3H), 1.63 (t, *J* = 6.8 Hz, 2H), 1.22 (s, 6H), 0.93 (s, 6H).

^{13}C NMR (101 MHz, $CDCl_3$): δ 156.2, 145.0, 130.9, 126.8, 123.8, 108.6, 55.6, 40.0, 34.6, 33.4, 26.6 (2C), 25.7, 24.6 (2C), 15.8.

IR (ATR, neat) $\tilde{\nu}$ = 2969 (m), 2922 (s), 1611 (m), 1574 (w), 1504 (s), 1461 (m), 1415 (w), 1393 (w), 1376 (m), 1364 (m), 1346 (w), 1314 (w), 1287 (m), 1263 (s), 1231 (m), 1210 (s), 1174 (m), 1159 (w), 1125 (w), 1076 (m), 1048 (s), 869 (m), 850 (w), 816 (m), 798 (m), 706 (w), 482 (w).

HRMS (ESI): calcd for $C_{16}H_{24}O^+$ $[M+Na]^+$: 256.1753; found: 256.1749

2.2.23 Chromane **31**

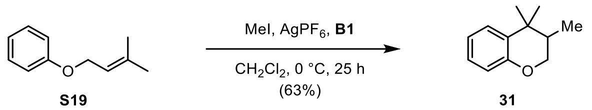

According to GP1, phenyl prenyl ether **S19** (20.3 mg, 125 µmol, 1 equiv) was converted at 0 °C over 25 h to chromane **31**. Purification by flash column chromatography on silica gel (10% to 20% dichloromethane in *n*-pentane) followed by semipreparative normal-phase high performance liquid chromatography (HPLC) (0.2% to 1.0% ethyl acetate in *n*-hexane over 40 min) afforded chromane **31** (13.9 mg, 78.9 µmol, 63%) as a colorless oil.

<u>Analytical data of chromane **31**:</u>

TLC (10% diethyl ether in *n*-pentane): R_f = 0.50 (UV, CAM).

^{1}**H NMR** (400 MHz, $CDCl_3$): δ 7.28 (dd, *J* = 7.8, 1.7 Hz, 1H), 7.07 (ddd, *J* = 8.1, 7.2, 1.7 Hz, 1H), 6.89 (td, *J* = 7.5, 1.4 Hz, 1H), 6.78 (dd, *J* = 8.1, 1.3 Hz, 1H), 4.10 (dd, *J* = 10.9, 3.4 Hz, 1H), 3.86 (dd, *J* = 10.9, 9.1 Hz, 1H), 1.93 (dqd, *J* = 9.0, 7.0, 3.4 Hz, 1H), 1.34 (s, 3H), 1.19 (s, 3H), 0.97 (d, *J* = 7.1 Hz, 3H).

^{13}**C NMR** (101 MHz, $CDCl_3$): δ 153.5, 131.9, 127.3, 127.1, 120.6, 116.8, 68.3, 37.5, 34.2, 29.3, 25.8, 12.0.

IR (ATR, neat): $\tilde{\nu}$ = 2964 (s), 2925 (s), 2880 (m), 1579 (w), 1489 (s), 1446 (m), 1311 (w), 1296 (w), 1286 (w), 1259 (w), 1224 (s), 1047 (m), 752 (s) cm^{-1}.

HRMS (ESI): calcd for $C_{12}H_{17}O^+$ $[M+H]^+$: 177.1274; found: 177.1271.

2.2.24 Tetrahydroquinoline **32**

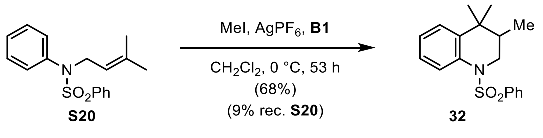

According to GP1, alkene **S20** (37.7 mg, 125 µmol, 1 equiv) was converted at 0 °C over 53 h to tetrahydroquinoline **32**. Purification by flash column chromatography on silica gel (5% to 15% ethyl acetate in *n*-pentane) afforded tetrahydroquinoline **32** (26.1 mg, 82.9 µmol, 68%) as a colorless oil and recovered alkene **S20** (3.39 mg, 11.2 µmol, 9%) as a colorless oil.

Analytical data of tetrahydroquinoline **32**:

TLC (5% ethyl acetate in cyclohexane): R_f = 0.33 (UV, CAM).

^{1}H NMR (400 MHz, $CDCl_3$): δ 7.81 (dd, *J* = 8.1, 1.5 Hz, 1H), 7.64 – 7.57 (m, 2H), 7.51 (tt, *J* = 7.4, 1.9, 1.3 Hz, 1H), 7.44 – 7.37 (m, 2H), 7.29 – 7.25 (m, 1H), 7.17 (td, *J* = 8.1, 7.5, 1.6 Hz, 1H), 7.11 (td, *J* = 7.5, 1.5 Hz, 1H), 3.99 (dd, *J* = 13.5, 3.7 Hz, 1H), 3.25 (dd, *J* = 13.5, 11.0 Hz, 1H), 1.43 (dqd, *J* = 10.8, 7.0, 3.7 Hz, 1H), 0.98 (s, 3H), 0.91 (s, 3H), 0.83 (d, *J* = 6.9 Hz, 3H).

^{13}C NMR (101 MHz, $CDCl_3$): δ 139.9, 139.0, 135.4, 132.9, 129.1 (2C), 127.4, 127.2 (2C), 126.4, 125.0, 123.9, 49.4, 36.3, 36.1, 28.2, 25.0, 13.5.

IR (ATR, neat): $\tilde{\nu}$ = 3065 (w), 2964 (m), 2926 (m), 1600 (w), 1511 (w), 1486 (m), 1446 (m), 1393 (w), 1354 (s), 1310 (w), 1292 (w), 1262 (w), 1224 (w), 1163 (s), 1090 (w) , 1074 (m), 1040 (w), 969 (w), 936 (w), 873 (w), 856 (w), 801(w), 758 (m), 730 (m), 705 (w), 689(m), 649 (w), 627 (w), 594 (s), 565 (m), 538 (w), 508 (w) cm^{-1}.

HRMS (ESI): calcd for $C_{18}H_{21}NO_2SNa^+$ $[M+Na]^+$: 338.1185; found: 338.1178.

2.2.25 Furan **33**

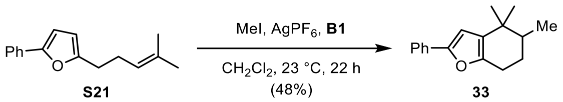

S21 **33**

According to GP1, alkene **S21** (28.3 mg, 125 µmol, 1 equiv) was converted at 23 °C over 22 h to furan **33**. Purification by flash column chromatography on silica gel (*n*-pentane) followed by semipreparative normal-phase high performance liquid chromatography (HPLC) (0.01% to 0.5% ethyl acetate in *n*-hexane over 40 min) afforded furan **33** (14.3 mg, 59.5 µmol, 48%) as a colorless oil.

Analytical data of furan **33**:

TLC (*n*-pentane): R_f = 0.40 (UV, CAM).

^{1}H NMR (400 MHz, $CDCl_3$): δ 7.64 – 7.58 (m, 2H), 7.37 – 7.31 (m, 2H), 7.23 – 7.14 (m, 1H), 6.54 (s, 1H), 2.68 – 2.62 (m, 2H), 1.84 – 1.59 (m, 3H), 1.22 (s, 3H), 1.03 (s, 3H), 0.97 (d, *J* = 6.5 Hz, 3H).

^{13}C NMR (101 MHz, $CDCl_3$): δ 152.1, 149.2, 131.6, 129.5, 128.7 (2C), 126.7, 123.3 (2C), 104.2, 39.3, 33.7, 28.5, 27.9, 24.0, 23.1, 15.6.

IR (ATR, neat): $\tilde{\nu}$ = 3033 (w), 2959 (m), 2929 (m), 2874 (w), 1602 (m), 1554 (w), 1487 (w), 1448 (w), 1402 (w), 1385 (w), 1373 (w), 1362 (w), 1340 (w), 1273 (w), 1231 (w), 1201 (w), 1149 (w), 1068 (m), 1040 (w), 1024 (w), 930 (w), 906 (w), 805 (w), 757 (s), 693 (s), 672 (w), 654 (w), 496 (w) cm^{-1}.

HRMS (ESI): calcd for $C_{17}H_{21}O^+$ $[M+H]^+$: 241.1587; found: 241.1583.

2.2.26 Pyrrole **34**

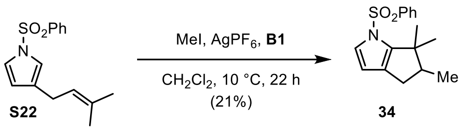

According to GP1, prenyl pyrrole **S22** (34.4 mg, 125 µmol, 1 equiv) was converted at 10 °C over 22 h to pyrrole **34**. Purification by flash column chromatography on silica gel (1% ethyl acetate in hexanes) followed by semipreparative normal-phase high performance liquid chromatography (HPLC) (1.0% to 2.0% ethyl acetate in *n*-hexane over 40 min) afforded pyrrole **34** (7.6 mg, 26 µmol, 21%) as a colorless oil.

Analytical data of pyrrole **34**:

TLC (5% diethyl ether in *n*-pentane): R_f = 0.47 (UV, CAM).

^{1}H NMR (400 MHz, $CDCl_3$): δ 7.74 – 7.69 (m, 2H), 7.59 – 7.54 (m, 1H), 7.51 – 7.43 (m, 2H), 7.18 (d, *J* = 3.3 Hz, 1H), 6.09 (d, *J* = 3.2 Hz, 1H), 2.55 (dd, *J* = 14.5, 7.5 Hz, 1H), 2.36 – 2.25 (m, 1H), 2.09 (dd, *J* = 14.6, 9.5 Hz, 1H), 1.18 (s, 3H), 1.01 – 0.97 (m, 6H).

^{13}C NMR (101 MHz, $CDCl_3$): δ 145.0, 140.4, 133.4, 131.0, 129.3 (2C), 126.6 (2C), 126.1, 108.9, 50.3, 43.8, 31.9, 26.0, 21.1, 14.1.

IR (ATR, neat): $\tilde{\nu}$ = 2959 (w), 2930 (w), 2871 (w), 2848 (w), 1448 (m), 1362 (s), 1329 (w), 1311 (w), 1288 (w), 1224 (m), 1174 (s), 1137 (m), 1120 (s), 1089 (w), 1074 (m), 1019 (w), 993 (w), 754 (w), 736 (m), 723 (s), 685 (m), 629 (s), 600 (m), 587 (s), 572 (m), 558 (m), 525 (w), 506 (w) cm^{-1}.

HRMS (ESI): calcd for $C_{16}H_{20}NO_2S^+$ $[M+H]^+$: 290.1209; found: 290.1200.

2.2.27 Indole **35**

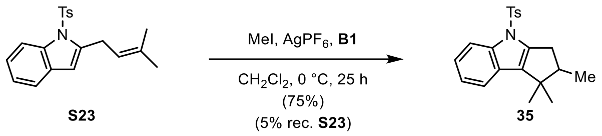

According to GP1, 2-prenyl indole **S23** (42.4 mg, 125 µmol, 1 equiv) was converted at 0 °C over 25 h to indole **35**. Purification by flash column chromatography on silica gel (3% diethyl ether in *n*-pentane) afforded indole **35** (33.1 mg, 93.6 µmol, 75%) as a colorless oil along with recovered 2-prenyl indole **S23** (1.9 mg, 5.6 µmol, 5%) as a colorless oil.

Analytical data of indole **35**:

TLC (5% diethyl ether in *n*-pentane): R_f = 0.32 (UV, CAM).

^{1}H NMR (400 MHz, $CDCl_3$): δ 8.04 – 7.99 (m, 1H), 7.74 – 7.68 (m, 2H), 7.44 – 7.39 (m, 1H), 7.24 – 7.16 (m, 4H), 3.30 (dd, *J* = 16.4, 7.7 Hz, 1H), 2.72 (dd, *J* = 16.4, 8.9 Hz, 1H), 2.55 (dp, *J* = 8.9, 7.1 Hz, 1H), 2.34 (s, 3H), 1.38 (s, 3H), 1.14 (d, *J* = 7.1 Hz, 3H), 1.06 (s, 3H).

^{13}C NMR (101 MHz, $CDCl_3$): δ 144.7, 140.4, 139.9, 136.0, 134.4, 130.0 (2C), 126.7 (2C), 126.7, 123.2, 123.2, 118.5, 114.7, 49.1, 41.6, 35.4, 27.2, 22.1, 21.7, 14.3.

IR (ATR, neat): $\tilde{\nu}$ = 3065 (w), 2956 (w), 2868 (w), 1598 (w), 1494 (w), 1466 (w), 1443 (m), 1396 (w), 1365 (s), 1341 (m), 1325 (w), 1306 (w), 1285 (w), 1220 (m), 1186 (m), 1170 (s), 1150 (m), 1120 (m), 1099 (m), 1076 (m), 1023 (w), 982 (w), 934 (w), 908 (w), 812 (m), 785 (m), 762 (m), 744 (s), 703 (w), 666 (s), 644 (w), 589 (s), 568 (s), 541 (s), 510 (w), 490 (w), 436 (w), 410 (w) cm^{-1}.

HRMS (ESI): calcd for $C_{21}H_{24}NO_2S^+$ $[M+H]^+$: 354.1522; found: 354.1516.

2.2.28 Indole **36**

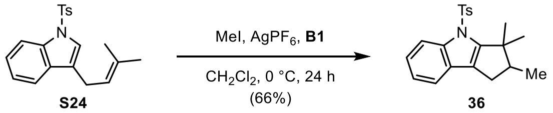

According to GP1, 3-prenyl indole **S24** (42.4 mg, 125 µmol, 1 equiv) was converted at 0 °C over 24 h to indole **36**. Purification by flash column chromatography on silica gel (2.5% diethyl ether in *n*-pentane) afforded indole **36** (29.3 mg, 82.9 µmol, 66%) as a colorless oil.

Analytical data of indole **36**:

TLC (10% diethyl ether in *n*-pentane): R_f = 0.58 (UV, CAM).

^{1}H NMR (400 MHz, $CDCl_3$): δ 8.12 – 8.07 (m, 1H), 7.67 – 7.62 (m, 2H), 7.37 – 7.33 (m, 1H), 7.26 – 7.18 (m, 2H), 7.20 – 7.16 (m, 2H), 2.81 (dd, *J* = 14.7, 7.6 Hz, 1H), 2.57 – 2.45 (m, 1H), 2.33 (s, 3H), 2.27 (dd, *J* = 14.7, 9.5 Hz, 1H), 1.47 (s, 3H), 1.23 (s, 3H), 1.13 (d, *J* = 7.0 Hz, 3H).

^{13}C NMR (101 MHz, $CDCl_3$): δ 150.9, 144.4, 140.8, 137.0, 129.8 (2C), 126.9, 126.5 (2C), 125.8, 123.8, 123.4, 119.2, 115.2, 50.3, 45.4, 30.4, 26.5, 21.7, 21.0, 14.4.

IR (ATR, neat): $\tilde{\nu}$ = 3052 (w), 2960 (w), 2926 (w), 2871 (w), 1597 (w), 1494 (w), 1481 (w), 1447 (m), 1402 (w), 1361 (s), 1322 (w), 1304 (w), 1273 (w), 1244 (w), 1221 (m), 1188 (m), 1173 (s), 1151 (m), 1125 (m), 1104 (m), 1079 (m), 1035 (w), 1022 (w), 986 (m), 937 (w), 909 (w), 830 (w), 810 (m), 745 (m), 703 (w), 667 (s), 641 (m), 608 (w), 575 (s), 544 (s), 457 (w), 431 (w) cm^{-1}.

HRMS (ESI): calcd for $C_{21}H_{23}NO_2SNa^+$ $[M+Na]^+$: 376.1342; found: 376.1336.

2.2.29 Benzofuran **37**

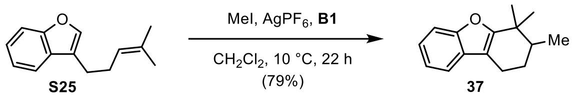

According to GP1, alkene **S25** (25.0 mg, 125 µmol, 1 equiv) was converted at 10 °C over 22 h to benzofuran **37**. Purification by flash column chromatography on silica gel (2% dichloromethane in *n*-pentane) followed by semipreparative normal-phase high performance liquid chromatography (HPLC) (0.1% to 2.0% ethyl acetate in *n*-hexane over 40 min) afforded benzofuran **37** (21.1 mg, 98.9 µmol, 79%) as a colorless oil.

For GP2 (22 h at 10 °C; purification as described above):
Benzofuran **37** (15.0 mg, 70.0 µmol, 56%)

Analytical data of benzofuran **37**:

TLC (100% *n*-pentane): R_f = 0.45 (UV, CAM).

^{1}H NMR (400 MHz, $CDCl_3$): δ 7.43 – 7.35 (m, 2H), 7.22 – 7.15 (m, 2H), 2.64 (ddd, *J* = 15.9, 5.9, 3.1 Hz, 1H), 2.56 (ddd, *J* = 15.8, 10.0, 5.6 Hz, 1H), 1.87 – 1.76 (m, 2H), 1.71 – 1.60 (m, 1H), 1.36 (s, 3H), 1.16 (s, 3H), 1.02 (d, *J* = 6.8 Hz, 3H).

^{13}C NMR (101 MHz, $CDCl_3$): δ 161.2, 154.7, 129.0, 123.0, 122.1, 118.7, 111.0, 111.0, 40.1, 35.7, 28.0, 25.9, 21.6, 20.1, 15.4.

IR (ATR, neat): $\tilde{\nu}$ = 3061 (w), 2966 (m), 2925 (m), 2874 (w), 1634 (w), 1613 (w), 1587 (w), 1477 (w), 1453 (s), 1395 (w), 1374 (w), 1361 (w), 1286 (w), 1260 (m), 1213 (w), 1195 (w), 1131 (m), 1091 (w), 1061 (w), 1042 (w), 1008 (w), 948 (w), 923 (w), 872 (w), 837 (w), 804 (w), 772 (w), 743 (s), 709 (w), 692 (w), 658 (w), 598 (w), 454 (w), 409 (w) cm^{-1}.

HRMS (ESI): calcd for $C_{15}H_{19}O^+$ $[M+H]^+$: 215.1430; found: 215.1429

2.2.30 Benzothiophene **38**

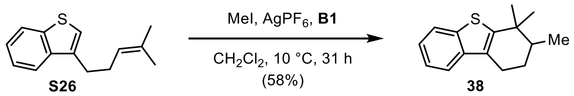

According to GP1, alkene **S26** (27.0 mg, 125 µmol, 1 equiv) was converted at 10 °C over 31 h to benzothiophene **38**. Purification by flash column chromatography on silica gel (100% in *n*-pentane) afforded benzothiophene **38** (16.7 mg, 72.5 µmol, 58%) as a colorless oil.

<u>Analytical data of benzothiophene **38**:</u>

TLC (100% *n*-pentane): R_f = 0.49 (UV, CAM).

^{1}H NMR (400 MHz, $CDCl_3$): δ 7.78 (dt, *J* = 7.9, 1.0 Hz, 1H), 7.55 (dt, *J* = 7.9, 1.1 Hz, 1H), 7.33 (ddd, *J* = 8.0, 7.1, 1.2 Hz, 1H), 7.29 – 7.23 (m, 1H), 2.83 (ddd, *J* = 16.4, 5.9, 3.5 Hz, 1H), 2.69 (ddd, *J* = 16.2, 9.7, 6.0 Hz, 1H), 1.94 – 1.84 (m, 2H), 1.80 – 1.71 (m, 1H), 1.42 (s, 3H), 1.25 (s, 3H), 1.05 (d, *J* = 6.7 Hz, 3H).

^{13}C NMR (101 MHz, $CDCl_3$): δ 149.4, 139.8, 138.8, 128.3, 123.9, 123.7, 122.5, 120.9, 40.1, 37.6, 31.0, 27.5, 25.8, 23.5, 16.0.

IR (ATR, neat): $\tilde{\nu}$ = 3060 (w), 2960 (m), 2924 (m), 2875 (m), 1461 (m), 1437 (m), 1389 (w), 1373 (w), 1363 (w), 1305 (w), 1264 (w), 1231 (w), 1204 (w), 1135 (w), 1097 (w), 1058 (w), 1044 (w), 1019 (w), 968 (w), 930 (w), 750 (s), 727 (s), 637 (w), 443 (w) cm^{-1}.

HRMS (ESI): calcd for $C_{15}H_{19}S^+$ $[M+H]^+$: 231.1202; found: 231.1200.

2.2.31 Tetracycle **39**

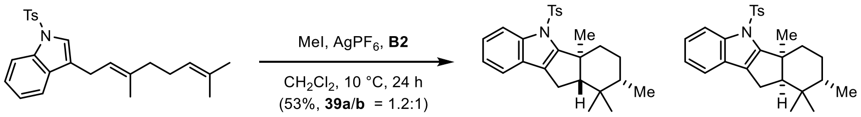

According to GP2, 3-geranyl indole **S27** (50.9 mg, 125 µmol, 1 equiv) was converted at 10 °C over 24 h to tetracycle **39a**/**b**. Purification by flash column chromatography on silica gel (20% to 50% dichloromethane in *n*-pentane) followed by high performance liquid chromatography (HPLC) (0.1% to 3.0% ethyl acetate in *n*-hexane over 40 min) afforded an inseparable mixture of tetracycle **39a/b** (27.8 mg, 65.9 µmol, 53%, **39a/b** = 1.20:1.00) as a colorless oil.

For GP1 (23 h at 10 °C; purification as described above):
Tetracycle **39a/b** (24.6 mg, 58.3 µmol, 47%, **39a/b** = 1.16:1.00)

<u>Analytical data of tetracycle **39a/b**:</u>

TLC (30% dichloromethane in *n*-pentane): R_f = 0.34 (UV, CAM).

^{1}H NMR (400 MHz, $CDCl_3$): δ 8.16 (dd, *J* = 7.5, 1.5 Hz, 1H, **39a**), 8.08 – 8.03 (m, 1H, **39b**), 7.70 – 7.65 (m, 2H, **39a**), 7.63 – 7.59 (m, 2H, **39b**), 7.38 – 7.32 (m, 1H, **39a**, 1H, **39b**), 7.28 – 7.14 (m, 4H, **39a**, 4H, **39b**), 2.74 – 2.69 (m, 1H, **39a**), 2.69 – 2.51 (m, 1H, **39a**, 2H, **39b**), 2.45 – 2.35 (m, 1H, **39a**, 1H, **39b**), 2.34 (s, 3H, **39a**), 2.33 (s, 3H, **39b**), 2.31 – 2.26 (m, 1H, **39b**), 2.15 (dd, *J* = 11.7, 6.2 Hz, 1H, **39a**), 1.66 (s, 3H, **39b**), 1.60 – 1.54 (m, 3H, **39a**, 1H, **39b**), 1.54

– 1.46 (m, 2H, **39b**), 1.37 – 1.30 (m, 1H, **39a**, 1H, **39b**), 1.21 (s, 3H, **39a**), 1.07 (s, 3H, **39b**), 1.02 (s, 3H, **39b**), 0.98 (s, 3H, **39a**), 0.92 (s, 3H, **39a**), 0.90 (d, *J* = 6.5 Hz, 3H, **39b**), 0.90 (d, *J* = 6.8 Hz, 3H, **39a**).

^{13}C NMR (101 MHz, $CDCl_3$): δ 152.5 (**39b**), 151.6 (**39a**), 144.5 (**39a**), 144.3 (**39b**), 140.6 (**39b**), 140.3 (**39a**), 136.9 (**39b**), 136.7 (**39a**), 129.8 (2C, **39a**), 129.7 (2C, **39b**), 127.4 (**39a**), 127.3 (**39b**), 127.1 (**39a**), 126.5 (2C, **39b**), 126.5 (2C, **39a**), 124.3 (**39b**), 123.8 (**39a**), 123.6 (**39b**), 123.5 (**39b**), 123.4 (**39a**), 119.2 (**39a**), 119.1 (**39b**), 115.4 (**39b**), 115.1 (**39a**), 64.5 (**39b**), 63.8 (**39a**), 47.6 (**39a**), 46.1 (**39b**), 43.1 (**39a**), 36.6 (**39a**), 36.5 (**39a**), 36.4 (**39b**), 35.9 (**39b**), 35.5 (**39b**), 29.8 (**39a**), 29.0 (**39a**), 28.7 (**39b**), 27.2 (**39b**), 25.8 (**39b**), 24.9 (**39b**), 24.5 (**39b**), 22.6 (**39a**), 21.7 (**39a**), 21.7 (**39b**), 19.0 (**39a**), 17.0 (**39a**), 15.8 (**39b**), 15.8 (**39a**).

IR (ATR, neat): $\tilde{\nu}$ = 2959 (w), 2864 (w), 1597 (w), 1448 (m), 1365 (m), 1296 (w), 1223 (w), 1187 (m), 1174 (s), 1153 (w), 1112 (w), 1023 (w), 988 (w), 908 (w), 829 (w), 811 (w), 744 (m), 704 (w), 669 (s), 621 (w), 575 (s), 545 (m) cm^{-1}.

HRMS (ESI): calcd for $C_{26}H_{31}NO_2SNa^+$ $[M+Na]^+$: 444.1968; found: 444.1959.

2.2.32 Tetracycle **40**

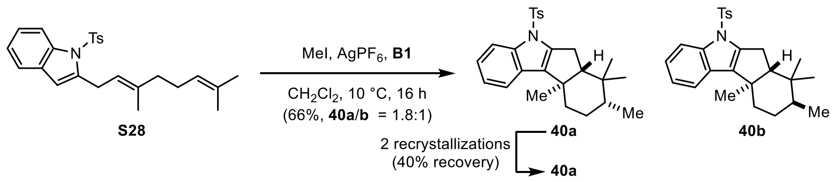

According to GP1, 2-geranyl indole **S28** (50.9 mg, 125 µmol, 1 equiv) was converted at 10 °C over 16 h to tetracycle **40a/b**. Purification by flash column chromatography on silica gel (15% to 40% dichloromethane in *n*-pentane) afforded an inseparable mixture of tetracycle **40a/b** (34.5 mg, 81.8 µmol, 66%, **40a/b** = 1.82:1.00) as an amorphous white solid.

Analytical data of tetracycle **40a/b**:

TLC (40% dichloromethane in *n*-pentane): R_f = 0.48 (UV, CAM).

^{1}H NMR (400 MHz, $CDCl_3$): δ 8.04 – 7.99 (m, 1H, **40a**, 1H, **40b**), 7.72 – 7.67 (m, 2H, **40a**, 2H, **40b**), 7.35 – 7.30 (m, 1H, **40a**, 1H, **40b**), 7.23 – 7.14 (m, 4H, **40a**, 4H, **40b**), 3.09 (dd, *J* = 15.5, 6.3 Hz, 1H, **40a**), 3.01 (dd, *J* = 15.4, 6.2 Hz, 1H, **40b**), 2.92 – 2.80 (m, 1H, **40a**, 1H, **40b**), 2.40 (dd, *J* = 11.7, 6.4 Hz, 1H, **40b**), 2.34 (s, 3H, **40a**, 3H, **40b**), 2.21 – 2.12 (m, 2H, **40a**), 2.07 – 1.98 (m, 1H, **40b**), 1.98 – 1.91 (m, 2H, **40b**), 1.79 – 1.69 (m, 1H, **40a**, 1H, **40b**), 1.62 – 1.54 (m, 2H, **40a**), 1.50 – 1.45 (m, 1H, **40b**), 1.41 – 1.33 (m, 1H, **40a**), 1.15 (s, 3H, **40b**), 1.10 (s,

3H, **40b**), 1.04 (s, 3H, **40a**), 1.03 (s, 3H, **40a**), 0.96 (d, *J* = 7.3 Hz, 3H, **40b**), 0.94 (s, 3H, **40a**), 0.94 (s, 3H, **40b**), 0.91 (d, *J* = 6.8 Hz, 3H, **40a**).

^{13}C NMR (101 MHz, $CDCl_3$): δ 144.7 (1C, **40a**, 1C, **40b**), 142.0 (**40a**), 141.9 (**40b**), 139.3 (1C, **40a**, 1C, **40b**), 136.7 (**40b**), 136.3 (**40a**), 136.0 (**40b**), 136.0 (**40a**), 130.0 (2C, **40a**, 2C, **40b**), 126.6 (2C, **40b**), 126.6 (2C, **40a**), 126.2 (**40a**), 126.1 (**40b**), 123.3 (1C, **40a**, 1C, **40b**), 123.2 (1C, **40a**, 1C, **40b**), 118.7 (**40a**), 118.7 (**40b**), 114.7 (1C, **40a**, 1C, **40b**), 64.2 (**40a**), 57.2 (**40b**), 43.3 (**40a**), 43.3 (**40a**), 43.0 (**40b**), 38.4 (**40b**), 36.3 (**40a**), 36.1 (**40a**), 34.9 (**40b**), 31.2 (**40b**), 29.9 (**40a**), 28.9 (**40a**), 28.2 (**40b**), 27.4 (**40a**), 27.1 (**40b**), 26.9 (**40b**), 24.4 (**40b**), 21.7 (1C, **40a**, 1C, **40b**), 21.4 (**40b**), 20.0 (**40a**), 16.8 (**40a**), 15.9 (**40b**), 15.9 (**40a**).

IR (ATR, neat): $\tilde{\nu}$ = 3065 (w), 2958 (m), 2921 (w), 2854 (w), 1598 (w), 1583 (w), 1494 (w), 1476 (w), 1443 (m), 1394 (w), 1368 (s), 1336 (w), 1311 (w), 1279 (w), 1226 (m), 1205 (w), 1188 (m), 1172 (s), 1150 (m), 1120 (m), 1092 (m), 1035 (w), 1020 (w), 993 (w), 970 (w), 944 (w), 908 (w), 812 (w), 764 (w), 745 (m), 705 (w), 669 (s), 634 (w), 602 (w), 577 (s), 542 (s), 505 (w), 426 (w) cm^{-1}.

HRMS (ESI): calcd for $C_{26}H_{32}NO_2S^+$ $[M+H]^+$: 422.2148; found: 422.2138.

Pure tetracycle **40a** could be obtained after two consecutive recrystallizations: To a solution of tetracycle **40a**/**b** (34.5 mg, 81.8 µmol, **40a**/**b** = 1.82:1.00) in dichloromethane (1.00 mL) was added a solution of methanol (1.00 mL) at 23 °C. After cooling to –30 °C for 48 h, the supernatant was removed to obtain white needles of tetracycle **40a**/**b** (14.1 mg, 34.6 µmol, **40a**/**b** = 6.05:1.00). To a solution of tetracycle **40a**/**b** (14.1 mg, 34.6 µmol, **40a**/**b** = 6.05:1.00) in dichloromethane (700 µL) was added a solution of methanol (700 µL) at 23 °C. After cooling to –30 °C for 120 h, the supernatant was removed to obtain white needles of tetracycle **40a** (9.2 mg, 22 µmol, 41% recovery), which were suitable for single crystal X-ray analysis.

<u>Analytical data of tetracycle **40a**:</u>

TLC (40% dichloromethane in *n*-pentane): R_f = 0.48 (UV, CAM).

mp: 193–194 °C.

^{1}H NMR (400 MHz, $CDCl_3$): δ 8.02 – 7.98 (m, 1H), 7.69 (d, *J* = 8.4 Hz, 2H), 7.34 – 7.30 (m, 1H), 7.23 – 7.13 (m, 4H), 3.08 (dd, *J* = 15.5, 6.3 Hz, 1H), 2.87 (dd, *J* = 15.5, 11.8 Hz, 1H), 2.34 (s, 3H), 2.21 – 2.11 (m, 2H), 1.73 (ddd, *J* = 12.2, 9.7, 7.5 Hz, 1H), 1.61 – 1.54 (m, 2H), 1.36 (dt, *J* = 9.5, 6.8 Hz, 1H), 1.04 (s, 3H), 1.02 (s, 3H), 0.94 (s, 3H), 0.90 (d, *J* = 6.7 Hz, 3H).

^{13}**C NMR** (101 MHz, $CDCl_3$): δ 144.7, 142.1, 139.3, 136.3, 136.0, 130.0 (2C), 126.6 (2C), 126.2, 123.3, 123.2, 118.7, 114.7, 64.3, 43.3, 43.3, 36.3, 36.1, 30.0, 28.9, 27.4, 21.7, 20.0, 16.8, 15.9.

IR (ATR, neat): $\tilde{\nu}$ = 2997 (w), 2957 (m), 2921 (m), 2853 (w), 1598 (w), 1476 (w), 1444 (m), 1394 (w), 1369 (m), 1337 (w), 1312 (w), 1279 (w), 1226 (w), 1204 (w), 1172 (s), 1150 (w), 1120 (w), 1093 (w), 1034 (w), 1019 (w), 993 (w), 907 (w), 813 (w), 745 (m), 706 (w), 670 (m), 633 (w), 602 (w), 578 (s), 542 (m), 427 (w), cm^{-1}.

HRMS (ESI): calcd for $C_{26}H_{31}NO_2SNa^+$ $[M+Na]^+$: 444.1968; found: 444.1957.

2.2.33 Cyclohexenes **41**

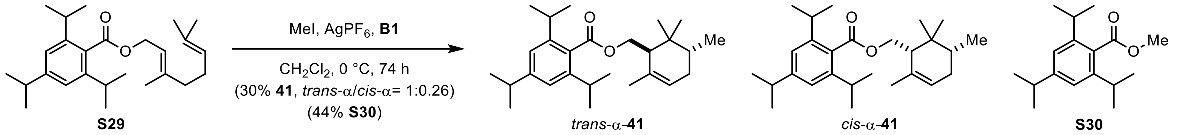

S29 *trans*-α-**41** *cis*-α-**41** **S30**

According to GP1, 1,3,5-triisopropylbenzoyl (TIB)-protected geraniol **S29** (24.5 mg, 63.7 µmol, 1 equiv) was converted at 0 °C over 74 h to cyclohexene *trans*-α-**41** and *cis*-α-**41**. Purification by flash column chromatography on silica gel (2% ethyl acetate in cyclohexane) followed by semipreparative normal-phase high performance liquid chromatography (HPLC) (0.2% to 1.5% ethyl acetate in *n*-hexane over 40 min) afforded an inseparable mixture of cyclohexane *trans*-α-**41** and *cis*-α-**41** (5.9 mg, 19 µmol, 30%, *trans*-α/*cis*-α = 1.00:0.26) as a colorless oil together with methyl ester **S30** (7.3 mg, 28 µmol, 44%) as a white solid.

The obtained analytical data for methyl ester **S30** were in accordance with reported literature values.[5]

<u>Analytical data of cyclohexene *trans*-α-**41** and *cis*-α-**41**:</u>

TLC (1.5% ethyl acetate in cyclohexane): R_f = 0.18 (UV, CAM).

^{1}**H NMR** (600 MHz, $CDCl_3$): δ 6.99 (s, 2H, *cis*-α-**41**), 6.99 (s, 2H, *trans*-α-**41**), 5.44 – 5.42 (m, 1H, *cis*-α-**41**), 5.42 – 5.40 (m, 1H, *trans*-α-**41**), 4.59 (dd, *J* = 11.9, 3.2 Hz, 1H, *cis*-α-**41**), 4.47 (dd, *J* = 12.0, 5.3 Hz, 1H, *trans*-α-**41**), 4.30 (dd, *J* = 11.9, 5.6 Hz, 1H, *cis*-α-**41**), 4.26 (dd, *J* = 12.0, 2.7 Hz, 1H, *trans*-α-**41**), 2.92 – 2.79 (m, 3H, *cis*-α-**41**, 3H, *trans*-α-**41**), 2.13 – 2.10 (m, 1H, *cis*-α-**41**), 1.94 (dq, *J* = 17.1, 4.8, 1.8 Hz, 1H, *trans*-α-**41**), 1.90 – 1.85 (m, 1H, *cis*-α-**41**), 1.85 – 1.82 (m, 1H, *trans*-α-**41**), 1.75 (q, *J* = 1.9 Hz, 3H, *trans*-α-**41**), 1.73 – 1.71 (m, 3H, *cis*-α-**41**), 1.72 – 1.63 (m, 1H, *cis*-α-**41**, 1H, *trans*-α-**41**), 1.63 – 1.59 (m, 1H, *trans*-α-**41**), 1.53 – 1.47 (m, 1H, *cis*-α-**41**), 1.26 – 1.21 (m, 18H, *cis*-α-**41**, 18H, *trans*-α-**41**), 1.04 (s, 3H, *cis*-α-**41**),

0.98 (s, 3H, *trans*-α-**41**), 0.86 (d, *J* = 6.8 Hz, 3H, *cis*-α-**41**), 0.81 (d, *J* = 6.6 Hz, 3H, *trans*-α-**41**), 0.78 (s, 3H, *trans*-α-**41**), 0.72 (s, 3H, *cis*-α-**41**).

^{13}C NMR (151 MHz, $CDCl_3$): δ 171.5 (*cis*-α-**41**), 171.4 (*trans*-α-**41**), 150.1 (*cis*-α-**41**), 150.0 (*trans*-α-**41**), 144.9 (2C, *cis*-α-**41**), 144.8 (2C, *trans*-α-**41**), 133.3 (*cis*-α-**41**), 132.4 (*trans*-α-**41**), 131.0 (*trans*-α-**41**), 130.9 (*cis*-α-**41**), 123.7 (*trans*-α-**41**), 123.6 (*cis*-α-**41**), 121.0 (2C, *cis*-α-**41**), 120.9 (2C, *trans*-α-**41**), 64.8 (*trans*-α-**41**), 64.7 (*cis*-α-**41**), 51.4 (*trans*-α-**41**), 50.0 (*cis*-α-**41**), 38.6 (*cis*-α-**41**), 35.2 (*cis*-α-**41**), 34.7 (*trans*-α-**41**), 34.5 (1C, *trans*-α-**41**, 1C, *cis*-α-**41**), 32.5 (*trans*-α-**41**), 32.4 (*trans*-α-**41**), 31.8 (2C, *cis*-α-**41**), 31.7 (2C, *trans*-α-**41**, 1C, *cis*-α-**41**), 26.7 (*cis*-α-**41**), 25.5 (*trans*-α-**41**), 24.6 (2C, *cis*-α-**41**), 24.4 (2C, *trans*-α-**41**), 24.2 (4C, *cis*-α-**41**), 24.1 (4C, *trans*-α-**41**), 22.9 (*trans*-α-**41**), 22.1 (*cis*-α-**41**), 21.3 (*trans*-α-**41**), 15.6 (*cis*-α-**41**), 15.3 (*trans*-α-**41**), 15.0 (*cis*-α-**41**).

IR (ATR, neat): $\tilde{\nu}$ = 2960 (s), 2928 (m), 2872 (m), 1723 (s), 1607 (w), 1575 (w), 1461 (m), 1383 (m), 1364 (m), 1283 (m), 1248 (s), 1188 (w), 1172 (w), 1136 (m), 1103 (m), 1075 (s), 1052 (m), 961 (w), 908 (w), 876 (m), 847 (w), 804 (w), 770 (w), 654 (w), 591 (w), 546 (w) cm^{-1}.

HRMS (ESI): calcd for $C_{27}H_{42}O_2Na^+$ $[M+Na]^+$: 421.3077; found: 421.3067.

2.2.34 Cyclohexenes **42**

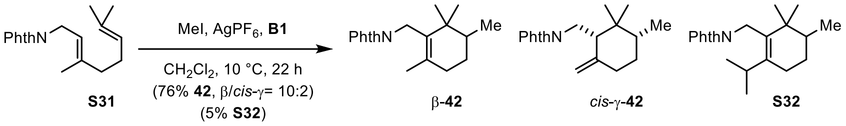

According to GP1, alkene **S31** (35.4 mg, 125 µmol, 1 equiv) was converted at 10 °C over 23 h to phthalimide β-**42**, *cis*-γ-**42** and **S32**. Purification by flash column chromatography on silica gel (5% to 100% diethyl ether in *n*-pentane) followed by semipreparative normal-phase high performance liquid chromatography (HPLC) (1.0% to 5.0% ethyl acetate in *n*-hexane over 40 min) afforded phthalimide β-**42** (24.3 mg, 81.7 µmol, 65%) as a colorless oil, phthalimide *cis*-γ-**42** (4.2 mg, 14 µmol, 11%) as a colorless oil, and phthalimide **S32** (2.0 mg, 6.1 µmol, 5%) as a colorless oil.

<u>Analytical data of phthalimide β-**42**:</u>

TLC (7% ethyl acetate in cyclohexane): R_f = 0.36 (UV, CAM).

^{1}H NMR (400 MHz, $CDCl_3$): δ 7.80 (dd, *J* = 5.4, 3.1 Hz, 2H), 7.68 (dd, *J* = 5.5, 3.0 Hz, 2H), 4.47 – 4.31 (m, 2H), 2.03 (t, *J* = 6.2 Hz, 2H), 1.79 (s, 3H), 1.59 (dtd, *J* = 14.3, 6.1, 2.7 Hz, 1H), 1.50 – 1.33 (m, 2H), 1.05 (s, 3H), 0.91 (s, 3H), 0.86 (d, *J* = 6.6 Hz, 3H).

^{13}C NMR (101 MHz, $CDCl_3$): δ 168.6 (2C), 134.0, 133.9 (2C), 132.2 (2C), 130.2, 123.2 (2C), 39.9, 37.7, 37.4, 32.2, 27.3, 26.8, 22.4, 20.8, 16.3.

IR (ATR, neat): $\tilde{\nu}$ = 2970 (w), 2876 (w), 1771 (w), 1707 (s), 1613 (w), 1467 (w), 1432 (w), 1396 (m), 1362 (m), 1329 (m), 1312 (m), 1188 (w), 1110 (w), 1088 (w), 1048 (w), 921 (w), 729 (m), 713 (m), 531 (w), 525 (w) cm^{-1}.

HRMS (ESI): calcd for $C_{19}H_{24}NO_2^+$ $[M+H]^+$: 298.1802; found: 298.1798.

Analytical data of phthalimide *cis*-γ-**42**:

TLC (7% ethyl acetate in cyclohexane): R_f = 0.36 (UV, CAM).

^{1}H NMR (400 MHz, $CDCl_3$): δ 7.83 – 7.77 (m, 2H), 7.71 – 7.66 (m, 2H), 4.80 (s, 1H), 4.76 (s, 1H), 4.07 (dd, *J* = 13.8, 11.0 Hz, 1H), 3.75 (dd, *J* = 13.8, 3.3 Hz, 1H), 2.74 – 2.62 (m, 1H), 2.26 (dt, *J* = 12.8, 4.2 Hz, 1H), 1.97 (td, *J* = 12.3, 4.5 Hz, 1H), 1.61 (dq, *J* = 12.8, 4.2 Hz, 1H), 1.54 – 1.47 (m, 1H), 1.34 – 1.26 (m, 1H), 1.18 (s, 3H), 0.90 (d, *J* = 6.8 Hz, 3H), 0.73 (s, 3H).

^{13}C NMR (101 MHz, $CDCl_3$): δ 168.9 (2C), 147.1, 133.9 (2C), 132.3 (2C), 123.2 (2C), 107.9, 51.2, 41.7, 38.9, 36.7, 36.3, 33.1, 27.1, 16.4, 15.5.

IR (ATR, neat): $\tilde{\nu}$ = 2972 (w), 2855 (w), 1771 (w), 1712 (s), 1648 (w), 1468 (w), 1392 (m), 1191 (w), 1123 (w), 1085 (w), 1008 (w), 902 (w), 717 (m), 614 (w), 531 (w) cm^{-1}.

HRMS (ESI): calcd for $C_{19}H_{24}NO_2^+$ $[M+H]^+$: 298.1802; found: 298.1801.

Analytical data of phthalimide **S32**:

TLC (7% ethyl acetate in cyclohexane): R_f = 0.36 (UV, CAM).

^{1}H NMR (400 MHz, $CDCl_3$): δ 7.84 – 7.77 (m, 2H), 7.71 – 7.65 (m, 2H), 4.45 (dt, *J* = 15.1, 1.3 Hz, 1H), 4.31 (d, *J* = 15.2 Hz, 1H), 3.15 (hept, *J* = 6.9 Hz, 1H), 2.09 – 1.94 (m, 2H), 1.63 (dtd, *J* = 12.4, 5.8, 2.6 Hz, 1H), 1.50 – 1.42 (m, 1H), 1.40 – 1.32 (m, 1H), 1.02 (s, 3H), 0.99 (d, *J* = 6.8 Hz, 3H), 0.95 (s, 3H), 0.93 (d, *J* = 6.8 Hz, 3H), 0.87 (d, *J* = 6.7 Hz, 3H).

^{13}C NMR (101 MHz, $CDCl_3$): δ 168.8 (2C), 142.9, 133.9 (2C), 132.3 (2C), 128.5, 123.1 (2C), 39.8, 38.1, 36.7, 30.5, 27.6, 26.7, 22.7, 22.7, 20.9, 20.8, 16.3.

IR (ATR, neat): $\tilde{\nu}$ = 2964 (w), 2927 (w), 1772 (w), 1714 (s), 1467 (w), 1397 (w), 1362 (w), 1109 (w), 728 (w), 713 (w) cm^{-1}.

HRMS (ESI): calcd for $C_{21}H_{28}NO_2^+$ $[M+H]^+$: 326.2115; found: 326.2113.

2.2.35 Cyclohexenes **43**

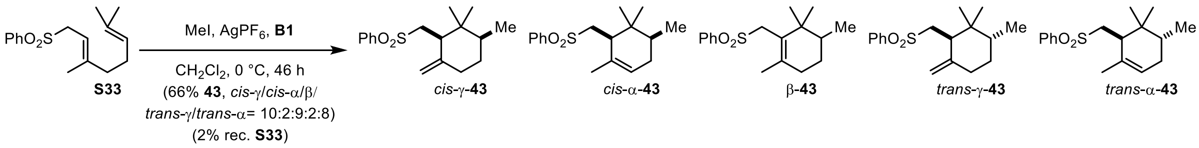

According to GP1, geranyl phenyl sulfone (**S33**) (34.8 mg, 125 µmol, 1 equiv) was converted at 0 °C over 46 h to cyclohexene *cis*-γ-**43**, *cis*-α-**43**, β-**43**, *trans*-γ-**43**, and *trans*-α-**43**. Purification by flash column chromatography on silica gel (5% to 15% ethyl acetate in *n*-pentane) followed by semipreparative normal-phase high performance liquid chromatography (HPLC) (2% to 10% ethyl acetate in *n*-hexane over 40 min) afforded cyclohexenes *cis*-γ-**43** (7.9 mg, 27 µmol, 22%), as a colorless oil, *cis*-α-**43** (1.4 mg, 4.9 µmol, 4%) as a colorless oil, β-**43** (6.9 mg, 24 µmol, 19%) as a colorless oil, *trans*-γ-**43** (1.3 mg, 4.3 µmol, 3%) as a colorless oil, *trans*-α-**43** (6.5 mg, 22 µmol, 18%) as a yellowish oil, and recovered alkene **S33** (0.7 mg, 3 µmol, 2%) as a colorless oil.

<u>Analytical data of cyclohexene *cis*-γ-**43**:</u>

TLC (5% ethyl acetate in *n*-pentane): R_f = 0.21 (UV, CAM).

^{1}H NMR (400 MHz, $CDCl_3$): δ 7.92 – 7.86 (m, 2H), 7.63 (tt, *J* = 7.4, 1.2 Hz, 1H), 7.57 – 7.50 (m, 2H), 4.77 (t, *J* = 1.7 Hz, 1H), 4.49 (t, *J* = 1.2 Hz, 1H), 3.44 (dd, *J* = 15.0, 9.2 Hz, 1H), 3.26 (dd, *J* = 15.0, 1.5 Hz, 1H), 2.42 (d, *J* = 9.2 Hz, 1H), 2.26 (ddd, *J* = 13.0, 4.4, 2.6 Hz, 1H), 2.00 (td, *J* = 13.2, 4.9 Hz, 1H), 1.60 – 1.51 (m, 1H), 1.51 – 1.41 (m, 1H), 1.19 (tdd, *J* = 13.1, 12.0, 4.4 Hz, 1H), 0.89 (s, 3H), 0.82 (d, *J* = 6.8 Hz, 3H), 0.47 (s, 3H).

^{13}C NMR (101 MHz, $CDCl_3$): δ 145.9, 140.1, 133.6, 129.2 (2C), 128.4 (2C), 108.1, 53.5, 48.1, 42.2, 39.7, 37.1, 32.8, 26.4, 16.7, 14.6.

IR (ATR, neat): $\tilde{\nu}$ = 3079 (w), 2964 (m), 2928 (m), 2857 (w), 1649 (w), 1447 (m), 1396 (w), 1370 (w), 1306 (s), 1261 (w), 1195 (w), 1144 (s), 1086 (m), 1071 (w), 1030 (w), 999 (w), 887 (w), 861 (w), 797 (w), 780 (w), 753 (m), 732 (m), 690 (m), 592 (m), 569 (m), 518 (w) cm^{-1}.

HRMS (ESI): calcd for $C_{17}H_{24}O_2SNa^+$ $[M+Na]^+$: 315.1389; found: 315.1381.

<u>Analytical data of cyclohexene *cis*-α-**43**:</u>

TLC (5% ethyl acetate in *n*-pentane): R_f = 0.34 (UV, CAM).

^{1}H NMR (400 MHz, $CDCl_3$): δ 7.96 – 7.93 (m, 2H), 7.66 (tt, *J* = 7.5, 1.2 Hz, 1H), 7.60 – 7.55 (m, 2H), 5.46 – 5.42 (m, 1H), 3.25 (dd, *J* = 15.3, 2.7 Hz, 1H), 3.15 (dd, *J* = 15.2, 5.7 Hz, 1H), 2.62 – 2.51 (m, 1H), 2.02 – 1.91 (m, 1H), 1.76 – 1.72 (m, 3H), 1.71 – 1.60 (m, 1H), 1.57 – 1.49 (m, 1H), 0.82 (d, *J* = 6.9 Hz, 3H), 0.79 (s, 3H), 0.61 (s, 3H).

^{13}C NMR (101 MHz, $CDCl_3$): δ 140.5, 133.7, 132.8, 129.4 (2C), 128.4 (2C), 123.8, 55.9, 44.4, 38.0, 36.1, 31.8, 26.4, 22.6, 16.4, 15.7.

IR (ATR, neat): $\tilde{\nu}$ = 2959 (s), 2924 (s), 2881 (m), 2854 (m), 1447 (m), 1391 (w), 1371 (w), 1305 (s), 1216 (w), 1151 (s), 1086 (m), 1066 (w), 1014 (w), 812 (w), 779 (w), 747 (m), 726 (m), 711 (w), 689 (m), 593 (s), 582 (m), 541 (m), 515 (w) cm^{-1}.

HRMS (ESI): calcd for $C_{17}H_{24}O_2SNa^+$ $[M+Na]^+$: 315.1389; found: 315.1381.

Analytical data of cyclohexene β-**43**:

TLC (5% ethyl acetate in *n*-pentane): R_f = 0.39 (UV, CAM).

^{1}H NMR (400 MHz, $CDCl_3$): δ 7.96 – 7.89 (m, 2H), 7.64 (tt, *J* = 7.3, 1.4 Hz, 1H), 7.59 – 7.52 (m, 2H), 3.99 (s, 2H), 2.15 – 1.99 (m, 2H), 1.68 (s, 3H), 1.67 – 1.60 (m, 1H), 1.56 – 1.49 (m, 1H), 1.47 – 1.36 (m, 1H), 1.06 (s, 3H), 0.91 (s, 3H), 0.90 (d, *J* = 6.8 Hz, 3H).

^{13}C NMR (101 MHz, $CDCl_3$): δ 141.9, 139.0, 133.4, 129.3 (2C), 128.0 (2C), 126.2, 58.1, 39.2, 37.7, 31.9, 27.9, 26.7, 22.8, 22.1, 16.7.

IR (ATR, neat): $\tilde{\nu}$ = 3064 (w), 2963 (m), 2925 (m), 2876 (m), 1447 (m), 1429 (w), 1412 (w), 1390 (w), 1376 (w), 1365 (w), 1317 (s), 1307 (s), 1243 (w), 1211 (w), 1149 (s), 1086 (m), 1040 (w), 1025 (w), 999 (w), 887 (w), 822 (w), 766 (w), 725 (m), 689 (m), 634 (w), 614 (w), 592 (m), 561 (m), 522 (w), 494 (w), 442 (w) cm^{-1}.

HRMS (ESI): calcd for $C_{17}H_{24}O_2SNa^+$ $[M+Na]^+$: 315.1389; found: 315.1381.

Analytical data of cyclohexene *trans*-γ-**43**:

TLC (5% ethyl acetate in *n*-pentane): R_f = 0.17 (UV, CAM).

^{1}H NMR (600 MHz, $CDCl_3$): δ 7.89 – 7.86 (m, 2H), 7.62 (tt, *J* = 7.5, 1.3 Hz, 1H), 7.55 – 7.51 (m, 2H), 4.67 (t, *J* = 2.0 Hz, 1H), 4.57 (t, *J* = 1.7 Hz, 1H), 3.34 (dd, *J* = 14.6, 9.5 Hz, 1H), 3.28 (dd, *J* = 14.5, 2.9 Hz, 1H), 2.44 (dd, *J* = 9.4, 2.9 Hz, 1H), 2.04 (ddd, *J* = 14.0, 5.2, 2.6 Hz, 1H), 2.01 – 1.94 (m, 1H), 1.48 – 1.43 (m, 1H), 1.38 – 1.32 (m, 1H), 1.23 – 1.15 (m, 1H), 0.88 (s, 3H), 0.77 (d, *J* = 6.7 Hz, 3H), 0.76 (s, 3H).

^{13}C NMR (151 MHz, $CDCl_3$): δ 145.5, 140.3, 133.6, 129.1 (2C), 128.4 (2C), 112.7, 55.1, 49.9, 37.5, 36.0, 31.6, 30.8, 26.6, 21.0, 15.7.

IR (ATR, neat): $\tilde{\nu}$ = 3070 (w), 2958 (s), 2925 (s), 2855 (m), 1733 (w), 1651 (w), 1447 (m), 1391 (w), 1374 (w), 1306 (s), 1260 (w), 1145 (s), 1087 (m), 1071 (w), 1052 (w), 1024 (w), 1000 (w), 979 (w), 945 (w), 896 (w), 866 (w), 798 (w), 749 (w), 717 (m), 689 (m), 655 (w), 595 (m), 580 (m), 528 (m), 457 (w), 442 w), 419 (w) cm^{-1}.

HRMS (ESI): calcd for $C_{17}H_{24}O_2SNa^+$ $[M+Na]^+$: 315.1389; found: 315.1381.

Analytical data of cyclohexene *trans*-α-**43**:

TLC (5% ethyl acetate in *n*-pentane): R_f = 0.30 (UV, CAM).

^{1}H NMR (400 MHz, $CDCl_3$): δ 7.96 – 7.89 (m, 2H), 7.64 (tt, *J* = 7.5, 1.4 Hz, 1H), 7.59 – 7.53 (m, 2H), 5.32 – 5.24 (m, 1H), 3.32 (dd, *J* = 15.1, 4.0 Hz, 1H), 2.88 (dd, *J* = 15.1, 4.0 Hz, 1H), 2.26 (t, *J* = 4.1 Hz, 1H), 1.98 – 1.87 (m, 1H), 1.63 (s, 3H), 1.62 – 1.56 (m, 1H), 1.34 (dq, *J* = 11.0, 6.6 Hz, 1H), 0.92 (s, 3H), 0.81 (d, *J* = 6.6 Hz, 3H), 0.80 (s, 3H).

^{13}C NMR (101 MHz, $CDCl_3$): δ 140.6, 135.7, 133.7, 129.4 (2C), 128.2 (2C), 122.3, 59.1, 45.1, 35.2, 32.4, 32.3, 25.3, 22.7, 20.5, 15.2.

IR (ATR, neat): $\tilde{\nu}$ = 3064 (w), 3033 (w), 2961 (m), 2878 (m), 2831 (w), 1447 (m), 1407 (w), 1390 (w), 1368 (w), 1307 (s), 1291 (m), 1241 (w), 1218 (w), 1147 (s), 1086 (m), 1071 (w), 1043 (w), 1014 (w), 998 (w), 959 (w), 911 (w), 878 (w), 803 (w), 777 (w), 745 (m), 721 (w), 689 (m), 594 (s), 556 (m), 538 (w), 521 (w), 508 (w), 438 (w) cm^{-1}.

HRMS (ESI): calcd for $C_{17}H_{24}O_2SNa^+$ $[M+Na]^+$: 315.1389; found: 315.1381.

2.3 Limitations

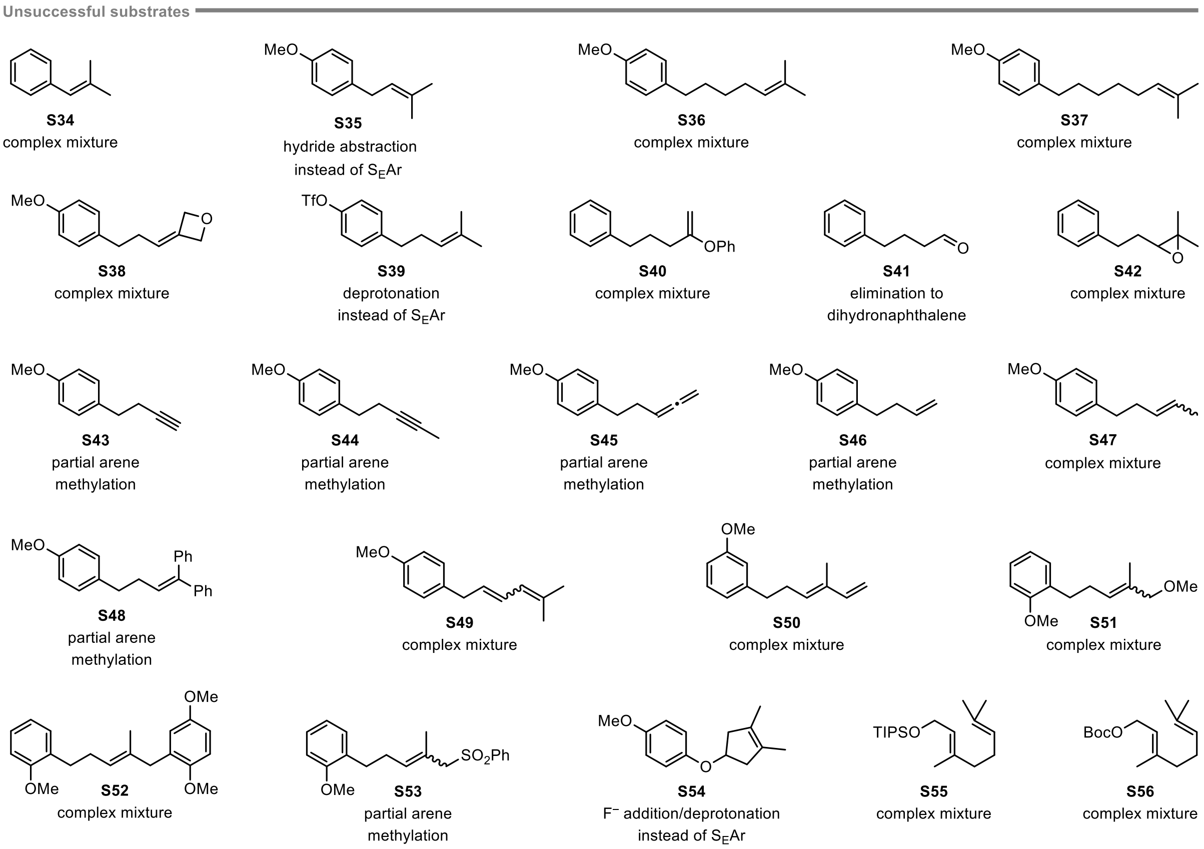

List of unsuccessful substrates along with the observed outcome based on NMR analysis. For substrates with partial arene methylation, most of the mass balance was accounted for by recovered starting material. For unsuccessful substrates with isolated side products, representative procedures are listed below.

2.3.1 Attempted cyclization of alkene **S35**

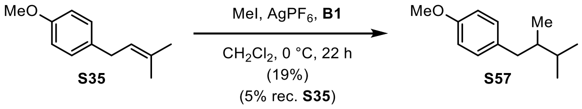

According to GP1, alkene **S35** (22.0 mg, 125 µmol, 1 equiv) was converted at 0 °C over 22 h to arene **S57**. Purification by flash column chromatography on silica gel (5% to 100% dichloromethane in *n*-pentane) followed by semipreparative normal-phase high performance liquid chromatography (HPLC) (0.1% to 1.8% ethyl acetate in *n*-hexane over 40 min) afforded recovered alkene **S35** (1.1 mg, 6.4 µmol, 5%) as a colorless oil and arene **S57** (4.6 mg, 24 µmol, 19%) as a colorless oil.

For GP2 (24 h at 10 °C; analysis via NMR standard):
Arene **S57** (4.7 mg, 25 µmol, 20% NMR yield)

<u>Analytical data of arene **S57**:</u>

TLC (5% dichloromethane in *n*-pentane): R_f = 0.25 (UV, CAM).

^{1}H NMR (400 MHz, $CDCl_3$): δ 7.06 (d, *J* = 8.6 Hz, 2H), 6.82 (d, *J* = 8.6 Hz, 2H), 3.79 (s, 3H), 2.63 (dd, *J* = 13.5, 5.2 Hz, 1H), 2.24 (dd, *J* = 13.5, 8.9 Hz, 1H), 1.66 – 1.56 (m, 2H), 0.91 (d, *J* = 6.6 Hz, 3H), 0.87 (d, *J* = 6.6 Hz, 3H), 0.75 (d, *J* = 6.6 Hz, 3H).

^{13}C NMR (101 MHz, $CDCl_3$): δ 157.7, 134.3, 130.1 (2C), 113.7 (2C), 55.4, 41.2, 40.0, 31.6, 20.6, 17.9, 14.9.

IR (ATR, neat): $\tilde{\nu}$ = 2957 (s), 2925 (s), 2854 (m), 1462 (m), 1256 (w), 1044 (w), 414 (w) cm^{-1}.

HRMS (ESI): calcd for $C_{13}H_{21}O^+$ [M+H]$^+$: 193.1587; found: 193.1583.

2.3.2 Attempted cyclization of alkyne **43**

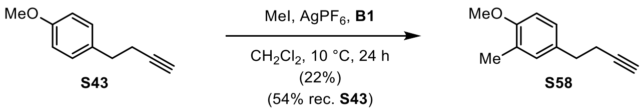

According to GP1, alkyne **S43** (20.1 mg, 125 µmol, 1 equiv) was converted at 10 °C over 24 h to alkyne **S58**. Purification by flash column chromatography on silica gel (0% to 30% diethyl ether in *n*-pentane) followed by semipreparative normal-phase high performance liquid chromatography (HPLC) (0.1% to 1.0% ethyl acetate in *n*-hexane over 40 min) afforded alkyne **S58** (4.7 mg, 27 µmol, 22%) as a colorless oil and recovered alkyne **S43** (10.8 mg, 67.4 µmol, 54%) as a colorless oil.

<u>Analytical data of alkyne **S58**:</u>

TLC (20% dichloromethane in *n*-pentane): R_f = 0.49 (UV, CAM).

^{1}H NMR (400 MHz, $CDCl_3$): δ 7.04 – 6.96 (m, 2H), 6.76 (d, *J* = 8.1 Hz, 1H), 3.81 (s, 3H), 2.76 (t, *J* = 7.6 Hz, 2H), 2.45 (td, *J* = 7.5, 2.6 Hz, 2H), 2.21 (s, 3H), 1.98 (t, *J* = 2.6 Hz, 1H).

^{13}C NMR (101 MHz, $CDCl_3$): δ 156.5, 132.3, 130.9, 126.6, 126.6, 110.0, 84.2, 68.9, 55.5, 34.2, 21.0, 16.4.

IR (ATR, neat): $\tilde{\nu}$ = 3289 (w), 2997 (w), 2951 (w), 2926 (m), 2857 (w), 2834 (w), 1613 (w), 1505 (s), 1466 (w), 1441 (w), 1309 (w), 1252 (s), 1226 (m), 1184 (w), 1134 (m), 1037 (m), 879 (w), 807 (w), 753 (w), 630 (m), 503 (w), 444 (w) cm^{-1}.

HRMS (ESI): calcd for $C_{12}H_{25}O^+$ [M+H]$^+$: 175.1117; found: 175.1116.

2.3.3 Attempted cyclization of cyclopentene ether **S54**

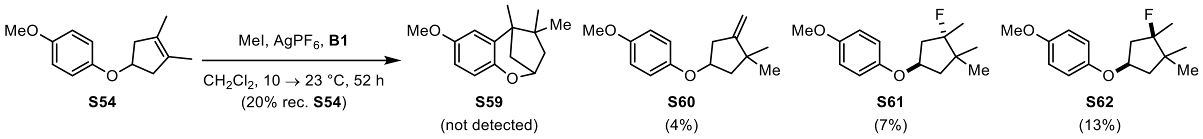

According to GP1, cyclopentene ether **S54** (27.3 mg, 125 µmol, 1 equiv) was converted at 10 °C over 44 h followed by 8 h at 23 °C to cyclopentene **S60** and fluoride **S61** and **S62**. Purification by flash column chromatography on silica gel (20% to 100% dichloromethane in *n*-pentane) followed by semipreparative normal-phase high performance liquid chromatography (HPLC) (0.5% to 2.0% ethyl acetate in *n*-hexane over 40 min) afforded cyclopentene **S60** (1.2 mg, 5.5 µmol, 4%) as a colorless oil, fluoride **S61** (2.1 mg, 8.3 µmol, 7%) as a colorless oil, fluoride **S62** (4.1 mg, 16 µmol, 13%) as a colorless oil, and recovered cyclopentene ether **S54** (5.5 mg, 25 µmol, 20%) as a colorless oil.

<u>Analytical data of cyclopentene **S60**:</u>

TLC (30% dichloromethane in *n*-pentane): R_f = 0.20 (UV, CAM).

^{1}H NMR (400 MHz, $CDCl_3$): δ 6.82 (s, 4H), 4.86 (t, *J* = 2.1 Hz, 1H), 4.83 (t, *J* = 2.4 Hz, 1H), 4.70 (tt, *J* = 6.3, 4.6 Hz, 1H), 3.77 (s, 3H), 2.89 (ddtd, *J* = 16.8, 6.6, 2.4, 1.9, 0.7 Hz, 1H), 2.69 – 2.61 (m, 1H), 1.96 (dd, *J* = 13.4, 6.0 Hz, 1H), 1.88 (ddd, *J* = 13.3, 4.7, 1.3 Hz, 1H), 1.22 (s, 3H), 1.11 (s, 3H).

^{13}C NMR (101 MHz, $CDCl_3$): δ 159.1, 153.9, 152.2, 116.9 (2C), 114.8 (2C), 104.8, 76.9, 55.9, 47.6, 41.3, 40.2, 30.1, 29.9.

IR (ATR, neat): $\tilde{\nu}$ = 3071 (w), 2956 (w), 2928 (w), 2865 (w), 2833 (w), 1654 (w), 1507 (s), 1464 (w), 1441 (w), 1362 (w), 1288 (w), 1230 (s), 1181 (w), 1106 (w), 1040 (m), 999 (w), 938 (w), 884 (w), 825 (w), 749 (w), 518 (w), 411 (w) cm^{-1}.

HRMS (ESI): calcd for $C_{15}H_{21}O_2^+$ [M+H]$^+$: 233.1536; found: 233.1534.

<u>Analytical data of fluoride **S61**:</u>

TLC (30% dichloromethane in *n*-pentane): R_f = 0.18 (UV, CAM).

^{1}H NMR (400 MHz, $CDCl_3$): δ 6.85 – 6.80 (m, 2H), 6.79 – 6.73 (m, 2H), 4.78 (tdd, *J* = 7.8, 4.8, 1.7 Hz, 1H), 3.76 (s, 3H), 2.59 (ddd, *J* = 21.0, 15.3, 7.8 Hz, 1H), 2.28 – 2.04 (m, 2H), 1.79 (d, *J* = 14.3 Hz, 1H), 1.34 (d, *J* = 22.4 Hz, 3H), 1.06 (s, 3H), 1.03 (d, *J* = 1.8 Hz, 3H).

^{13}C NMR (101 MHz, $CDCl_3$): δ 153.8, 152.1, 116.4 (2C), 114.8 (2C), 107.3 (d, *J* = 171.2 Hz), 75.7, 55.9, 45.7 (d, *J* = 23.1 Hz), 45.8, 44.5 (d, *J* = 19.7 Hz), 26.3 (d, *J* = 6.0 Hz), 21.3 (d, *J* = 8.0 Hz), 18.5 (d, *J* = 26.0 Hz).

^{19}F NMR (376 MHz, $CDCl_3$) δ –144.6.

IR (ATR, neat): $\tilde{\nu}$ = 2963 (w), 2834 (w), 1507 (s), 1469 (w), 1441 (w), 1390 (w), 1379 (w), 1366 (w), 1320 (w), 1287 (w), 1230 (s), 1187 (w), 1135 (m), 1106 (w) 1039 (m), 1005 (w), 910 (w), 825 (m), 738 (w), 525 (w) cm^{-1}.

HRMS (ESI): calcd for $C_{15}H_{22}FO_2^+$ $[M+H]^+$: 253.1598; found: 253.1594.

<u>Analytical data of fluoride **S62**:</u>

TLC (30% dichloromethane in *n*-pentane): R_f = 0.08 (UV, CAM).

^{1}H NMR (400 MHz, $CDCl_3$): δ 6.91 – 6.70 (m, 4H), 4.72 (qd, *J* = 7.3, 2.8 Hz, 1H), 3.76 (s, 3H), 2.40 (ddd, *J* = 38.2, 15.8, 8.6 Hz, 1H), 2.21 (ddd, *J* = 24.0, 15.8, 2.8 Hz, 1H), 2.10 (dd, *J* = 7.2, 2.0 Hz, 2H), 1.31 (d, *J* = 22.0 Hz, 3H), 1.09 (d, *J* = 1.7 Hz, 3H), 0.90 (s, 3H).

^{13}C NMR (101 MHz, $CDCl_3$): δ 153.8, 152.4, 116.6 (2C), 114.7 (2C), 105.5 (d, *J* = 175.3 Hz), 75.6, 55.9, 46.7, 45.1 (d, *J* = 21.1 Hz), 44.6 (d, *J* = 23.3 Hz), 25.8 (d, *J* = 6.0 Hz), 21.0 (d, *J* = 7.0 Hz), 18.7 (d, *J* = 26.3 Hz).

^{19}F NMR (376 MHz, $CDCl_3$) δ –142.9.

IR (ATR, neat): $\tilde{\nu}$ =2964 (w), 2939 (w), 2834 (w), 1507 (s),1468 (w), 1443 (w), 1379 (w), 1368 (w) 1288 (w), 1230 (s), 1187 (w), 1148 (w), 1126 (w), 1108 (w), 1081 (w), 1038 (m), 899 (w), 825 (m), 806 (w), 741 (w), 524 (w) cm^{-1}.

HRMS (ESI): calcd for $C_{15}H_{21}FO_2Na^+$ $[M+Na]^+$: 275.1418; found: 275.1412.

2.4 Substrate synthesis

2.4.1 Alkene **1**

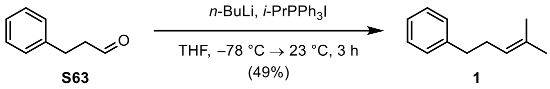

Alkene **1** was prepared according to a known literature procedure[6]: To a suspension of isopropyltriphenylphosphonium iodide (12.0 g, 27.8 mmol, 1.85 equiv) in tetrahydrofuran (75.1 mL) was added a solution of *n*-butyllithium (2.50 M in hexanes, 10.5 mL, 26.3 mmol, 1.75 equiv) at –78 °C, upon which the suspension turned from yellow to orange-red. After stirring for 30 min at –78 °C, the reaction mixture was warmed to 23 °C through exchange of the cooling bath with a water bath. Stirring was continued for 30 min at 23 °C, during which the suspension turned red-brownish. The reaction mixture was cooled to –78 °C and a solution of 3-phenylpropanal (**S63**) (2.02 g, 15.0 mmol, 1 equiv) in tetrahydrofuran (11.7 mL) was added resulting in slight decolorization and immediate formation of an off-white solid. Stirring was continued for 1 h at –78 °C, followed by exchange of the cooling bath with a water bath and stirring for 45 min at 23 °C. Next, a saturated aqueous solution of ammonium chloride (60 mL) was added, which resulted in decolorization of the reaction mixture. The organic layer was separated and the aqueous layer was extracted with diethyl ether (2 × 50 mL). The combined organic layers were dried over sodium sulfate, the dried organic layer was filtered, and the filtrate was concentrated under reduced pressure. The residue was purified by flash column chromatography on silica gel (*n*-pentane) to yield alkene **1** (1.19 g, 7.42 mmol, 49%) as a colorless oil. The obtained analytical data for alkene **1** were in accordance with reported literature values.[6]

2.4.2 Alkene **S1**

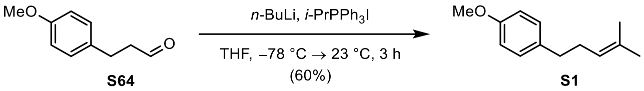

Alkene **S1** was prepared according to a known literature procedure[6]: To a suspension of isopropyltriphenylphosphonium iodide (5.72 g, 13.2 mmol, 1.85 equiv) in tetrahydrofuran (36.0 mL) was added a solution of *n*-butyllithium (2.50 M in hexanes, 5.00 mL, 12.5 mmol, 1.75 equiv) at –78 °C, upon which the suspension turned from yellow to orange-red. After stirring for 30 min at –78 °C, the reaction mixture was warmed to 23 °C through exchange of the cooling bath with a water bath. Stirring was continued for 30 min at 23 °C, during which the suspension turned red-brownish. The reaction mixture was cooled to –78 °C and a solution of 3-(4-methoxyphenyl)propanal (**S64**) (1.17 g, 7.15 mmol, 1 equiv) in tetrahydrofuran

(5.60 mL) was added resulting in slight decolorization and immediate formation of an off-white solid. Stirring was continued for 30 min at –78 °C, followed by exchange of the cooling bath with a water bath and stirring for 1 h 30 min at 23 °C. Next, a saturated aqueous solution of ammonium chloride (60 mL) was added, which resulted in decolorization of the reaction mixture. The organic layer was separated and the aqueous layer was extracted with diethyl ether (2 × 50 mL). The combined organic layers were dried over sodium sulfate, the dried organic layer was filtered, and the filtrate was concentrated under reduced pressure. The residue was purified by flash column chromatography on silica gel (2% to 15% dichloromethane in *n*-pentane) to yield alkene **S1** (810 mg, 4.26 mmol, 60%) as a colorless oil. The obtained analytical data for alkene **S1** were in accordance with reported literature values.[6]

2.4.3 Alkene **S2**

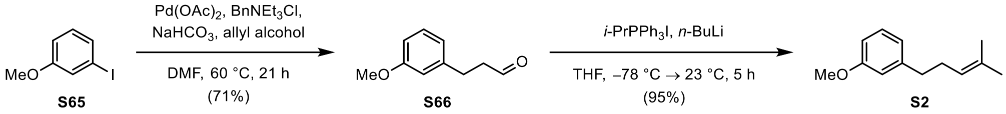

Aldehyde **S66** was prepared according to a modified literature procedure[7]: To a suspension of sodium bicarbonate (1.32 g, 15.7 mmol. 2.50 equiv), benzyltriethylammonium chloride (1.43 g, 6.28 mmol, 1.00 equiv), palladium diacetate (28.2 mg, 126 µmol, 2.00 mol%), 3-iodoanisole (**S65**) (1.47 g, 6.28 mmol, 1 equiv) in degassed dimethylformamide (21.0 mL) was added allyl alcohol (547 mg, 9.42 mmol, 1.50 equiv) at 23 °C under argon atmosphere. The reaction mixture was heated to 60 °C for 21 h, after which the resulting black suspension was cooled down to 23 °C, diluted with ethyl acetate (120 mL), and washed with a saturated aqueous solution of sodium chloride (3 × 30 mL). The washed organic layer was dried over sodium sulfate, the dried organic layer was filtered, and the filtrate was concentrated under reduced pressure. The residue was purified by flash column chromatography on silica gel (7% ethyl acetate in cyclohexane) to yield aldehyde **S66** (733 mg, 4.46 mmol, 71%) as a slightly yellowish oil. The obtained analytical data for aldehyde **S66** were in accordance with reported literature values.[7]

Alkene **S2** was prepared according to a modified literature procedure[8]: To a suspension of isopropyltriphenylphosphonium iodide (2.72 g, 6.29 mmol, 1.45 equiv) in tetrahydrofuran (21.7 mL) was added a solution of *n*-butyllithium (1.60 M in hexanes, 3.66 mL, 5.86 mmol, 1.35 equiv) at –78 °C, upon which the suspension turned from yellowish to orange. After stirring for 30 min at –78 °C, the reaction mixture was warmed to 23 °C through exchange of the cooling bath with a water bath. After stirring for 30 min, the red reaction mixture was cooled

to –78 °C and a solution of aldehyde **S66** (713 mg, 4.34 mmol, 1 equiv) in tetrahydrofuran (3.40 mL) was added, which resulted in a color change from red to yellow and immediate formation of an off-white solid. After stirring for 30 min at –78 °C, the reaction mixture was warmed to 23 °C through exchange of the cooling bath with a water bath. After stirring for 3 h 30 min at 23 °C, the reaction mixture was diluted with a saturated aqueous solution of ammonium chloride (50 mL) and diethyl ether (80 mL), which resulted in decolorization of the reaction mixture. The resulting suspension was filtered through a glass sinter filter and the remaining solid was washed with diethyl ether (40 mL) before being discharged. The organic layer was separated and the aqueous layer was extracted with diethyl ether (2 × 30 mL). The combined organic layers were washed successively with a saturated aqueous solution of sodium chloride (2 × 20 mL), an aqueous hydrogen peroxide solution (10 wt%, 2 × 20 mL) and a saturated aqueous solution of sodium chloride (2 × 20 mL). The washed organic layer was dried over sodium sulfate, the dried organic layer was filtered, and the filtrate was concentrated under reduced pressure. The residue was purified by flash column chromatography on silica gel (12% to 15% dichloromethane in *n*-pentane) to yield alkene **S2** (781 mg, 4.10 mmol, 95%) as a colorless oil. The obtained analytical data for alkene **S2** were in accordance with reported literature values.[8]

2.4.4 Alkene **S3**

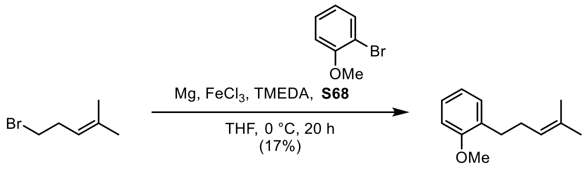

Alkene **S3** was prepared according to a modified literature procedure[9]: To a flask filled with magnesium chips (71.6 mg, 2.94 mmol, 1.20 equiv) was added in succession a solution of iron(III) chloride (59.7 mg, 368 µmol, 0.150 equiv) in dry tetrahydrofuran (5.00 mL) and *N*,*N*,*N'*,*N'*-tetramethylethylenediamine (441 µL, 2.94 mmol, 1.20 equiv) and the resulting suspension was stirred for 20 minutes. Subsequently, the reaction mixture was cooled to 0 °C and 2-bromoanisole (**S68**) (918 mg, 4.91 mmol, 2.00 equiv) and homoprenyl bromide (**S67**) (400 mg, 2.45 mmol, 1 equiv) were added in succession. After stirring for 20 h at 0 °C, a saturated aqueous solution of ammonium chloride (10 mL), a 1 M aqueous solution of hydrochloric acid (4 mL), and diethyl ether (5 mL) were added. The aqueous layer was separated and extracted with diethyl ether (3 × 10 mL). The combined organic layers were dried over sodium sulfate, the dried organic layer was filtered, and the filtrate was concentrated under reduced pressure. The residue was purified by flash column chromatography on silica

gel (0% to 2% diethyl ether in *n*-pentane) to afford the alkene **S3** (78.3 mg, 411 µmol, 17%) as a colorless oil. The obtained analytical data for alkene **S3** were in accordance with reported literature values.[10]

2.4.5 Alkene **S4**

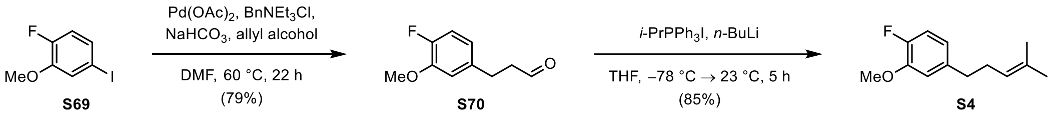

Preparation of aldehyde **S70**: To a suspension of sodium bicarbonate (1.30 g, 15.5 mmol, 2.50 equiv), benzyltriethylammonium chloride (1.41 g, 6.19 mmol, 1.00 equiv), palladium diacetate (27.7 mg, 123 µmol, 2.00 mol%), 1-fluoro-4-iodo-2-methoxybenzene (**S69**) (1.56 g, 6.19 mmol, 1 equiv) in degassed dimethylformamide (21.0 mL) was added allyl alcohol (539 mg, 9.29 mmol, 1.50 equiv) at 23 °C under argon atmosphere. The reaction mixture was heated to 60 °C for 22 h, after which the resulting black suspension was cooled down to 23 °C, diluted with ethyl acetate (120 mL), and washed with a saturated aqueous solution of sodium chloride (3 × 20 mL). The washed organic layer was dried over sodium sulfate, the dried organic layer was filtered, and the filtrate was concentrated under reduced pressure. The residue was purified by flash column chromatography on silica gel (8% to 14% ethyl acetate in cyclohexane) to yield aldehyde **S70** (894 mg, 4.91 mmol, 79%) as a yellowish oil.

Analytical data of aldehyde **S70**:

TLC (20% diethyl ether in *n*–pentane): R_f = 0.27 (UV, CAM).

^{1}H NMR (400 MHz, $CDCl_3$): δ 9.81 (t, *J* = 1.3 Hz, 1H), 6.97 (dd, *J* = 11.3, 8.2 Hz, 1H), 6.79 (dd, *J* = 8.1, 2.1 Hz, 1H), 6.69 (ddd, *J* = 8.1, 4.1, 2.1 Hz, 1H), 3.87 (s, 3H), 2.91 (t, *J* = 7.4 Hz, 2H), 2.77 (tt, *J* = 7.3, 1.1 Hz, 2H).

^{13}C NMR (101 MHz, $CDCl_3$): δ 201.3, 151.2 (d, *J* = 243.8 Hz), 147.6 (d, *J* = 10.8 Hz), 136.8 (d, *J* = 3.9 Hz), 120.4 (d, *J* = 6.7 Hz), 116.0 (d, *J* = 18.3 Hz), 113.8 (d, *J* = 1.9 Hz), 56.3, 45.5, 27.9.

^{19}F NMR (376 MHz, $CDCl_3$) δ –138.9.

IR (ATR, neat): $\tilde{\nu}$ = 2941 (w), 2984 (w), 2726 (w), 1721 (s), 1610 (m), 1515 (s), 1465 (m), 1452 (m), 1419 (m), 1389 (w), 1350 (w), 1319 (w), 1280 (m), 1266 (s), 1215 (s), 1189 (w), 1152 (s), 1120 (w), 1158 (w), 1030 (m), 935 (m), 934 (w), 866 (m), 812 (m), 783 (m), 755 (w) 623 (w), 557 (w), 542 (w), 578 (w), 554 (w) cm^{-1}.

HRMS (ESI): calcd for $C_{10}H_{11}FO_2Na^+$ $[M+Na]^+$: 205.0635; found: 205.0634.

Preparation of alkene **S4**: To a suspension of isopropyltriphenylphosphonium iodide (2.94 g, 6.80 mmol, 1.40 equiv) in tetrahydrofuran (24.3 mL) was added a solution of *n*-butyllithium (1.60 M in hexanes, 4.10 mL, 6.56 mmol, 1.35 equiv) at –78 °C, upon which the suspension turned from white to yellow. After stirring for 30 min at –78 °C, the reaction mixture was warmed to 23 °C through exchange of the cooling bath with a water bath. After stirring for 30 min, the red reaction mixture was cooled to –78 °C and a solution of aldehyde **S70** (885 mg, 4.86 mmol, 1 equiv) in tetrahydrofuran (3.80 mL) was added, which resulted in a color change from red to yellow. After stirring for 30 min at –78 °C, the reaction mixture was warmed to 23 °C through exchange of the cooling bath with a water bath. After stirring for 3 h at 23 °C, the reaction mixture was diluted with a saturated aqueous solution of ammonium chloride (25 mL) and *n*-pentane (25 mL), which resulted in decolorization of the reaction mixture. The resulting suspension was filtered through a glass sinter filter and the remaining solid was washed with diethyl ether (30 mL) before being discharged. The organic layer was separated and the aqueous layer was extracted with diethyl ether (3 × 30 mL). The combined organic layers were washed successively with a saturated aqueous solution of sodium chloride (2 × 50 mL), an aqueous hydrogen peroxide solution (10 wt%, 3 × 30 mL) and a saturated aqueous solution of sodium chloride (2 × 50 mL). The washed organic layer was dried over sodium sulfate, the dried organic layer was filtered, and the filtrate was concentrated under reduced pressure. The residue was purified by flash column chromatography on silica gel (30% dichloromethane in *n*-pentane) to yield alkene **S4** (865 mg, 4.15 mmol, 85%) as a yellow oil.

Analytical data of alkene **S4**:

TLC (10% dichloromethane in pentane): R_f = 0.32 (UV, CAM).

^{1}H NMR (400 MHz, $CDCl_3$): δ 6.96 (dd, *J* = 11.4, 8.2 Hz, 1H), 6.78 (dd, *J* = 8.3, 2.1 Hz, 1H), 6.69 (ddd, *J* = 8.2, 4.3, 2.1 Hz, 1H), 5.14 (thept, *J* = 6.9, 1.4 Hz, 1H), 3.88 (s, 3H), 2.59 (dd, *J* = 8.7, 6.8 Hz, 2H), 2.27 (q, *J* = 7.6 Hz, 2H), 1.69 (q, *J* = 1.3 Hz, 3H), 1.56 (s, 3H).

^{13}C NMR (101 MHz, $CDCl_3$): δ 151.0 (d, *J* = 242.7 Hz), 147.3 (d, *J* = 10.7 Hz), 138.8 (d, *J* = 3.7 Hz), 132.5, 123.6, 120.6 (d, *J* = 6.6 Hz), 115.7 (d, *J* = 18.1 Hz), 113.9 (d, *J* = 1.8 Hz), 56.4, 35.9, 30.2, 25.8, 17.8.

^{19}F NMR (376 MHz, $CDCl_3$) δ –140.0.

IR (ATR, neat): $\tilde{\nu}$ = 2965 (w), 2924 (w), 2957 (w), 1609 (m), 1517 (s), 1464 (m), 1452 (m), 1417 (w), 1317 (w), 1281 (s), 1266 (w), 1217 (m), 1189 (m), 1152 (m), 1119 (m), 1036 (m), 850 (w), 813 (w), 781 (w) cm^{-1}.

HRMS (ESI): calcd for $C_{13}H_{18}FO^+$ $[M+H]^+$: 209.1336; found: 209.1335.

2.4.6 Alkene **S5**

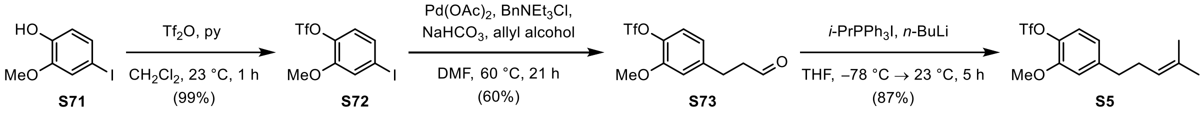

Triflate **S72** was prepared according to a modified literature procedure[11]: To a solution of 4-iodo-2-methoxyphenol (**S71**) (2.02 g, 8.08 mmol, 1 equiv) and pyridine (1.44 mL, 17.8 mmol, 2.20 equiv) in dichloromethane (51.0 mL) was added trifluoromethanesulfonic anhydride (1.62 mL, 9.69 mmol, 1.20 equiv) dropwise at 23 °C. After stirring for 1 h at 23 °C, the reaction mixture was diluted with diethyl ether (100 mL) and washed in succession with a saturated aqueous solution of sodium bicarbonate (2 × 50 mL) and a saturated aqueous solution of sodium chloride (50 mL). The washed organic layer was dried over sodium sulfate, the dried organic layer was filtered, and the filtrate was concentrated under reduced pressure. The residue was purified by flash column chromatography on silica gel (10% diethyl ether in *n*-pentane) to yield triflate **S72** (3.04 g, 7.96 mmol, 99%) as a colorless oil. The obtained analytical data for trilfate **S72** were in accordance with reported literature values.[11]

Preparation of aldehyde **S73**: To a vial charged with palladium(II) acetate (89.4 mg, 398 µmol, 5.00 mol%), sodium bicarbonate (1.67 g, 19.9 mmol, 2.50 equiv), and benzyl(triethyl)ammonium chloride (1.81 g, 7.96 mmol, 1.00 equiv) was added in succession a solution of triflate **S72** (3.04 g, 7.96 mmol, 1 equiv) in degassed dimethylformamide (27.0 mL) and allyl alcohol (624 µL, 9.16 mmol, 1.15 equiv) at 23 °C under an argon atmosphere. The resulting orange suspension was heated to 60 °C for 21 h, after which the reaction mixture was allowed to cool to 23 °C, diluted with ethyl acetate (100 mL), and washed with a saturated aqueous solution of sodium chloride (3 × 40 mL). The washed organic layer was dried over sodium sulfate, the dried organic layer was filtered, and the filtrate was concentrated under reduced pressure. The residue was purified by flash column chromatography on silica gel (40% to 50% diethyl ether in *n*-pentane) to yield aldehyde **S73** (1.49 g, 4.76 mmol, 60%) as a slightly yellowish oil.

<u>Analytical data of aldehyde **S73**:</u>

TLC (60% diethyl ether in *n*-pentane): R_f = 0.36 (UV, $KMnO_4$).

^{1}**H NMR** (400 MHz, $CDCl_3$): δ 9.82 (t, *J* = 1.1 Hz, 1H), 7.12 (d, *J* = 8.3 Hz, 1H), 6.87 (d, *J* = 2.0 Hz, 1H), 6.79 (dd, *J* = 8.3, 2.0 Hz, 1H), 3.89 (s, 3H), 2.96 (t, *J* = 7.3 Hz, 2H), 2.81 (tt, *J* = 7.4, 1.0 Hz, 2H).

^{13}**C NMR** (101 MHz, $CDCl_3$): δ 200.8, 151.4, 142.4, 137.3, 122.5, 120.6, 118.9 (q, *J* = 320.4 Hz), 113.5, 56.3, 45.1, 28.0.

^{19}**F NMR** (376 MHz, $CDCl_3$) δ –73.9.

IR (ATR, neat): $\tilde{\nu}$ = 2945 (w), 2835 (w), 2729 (w), 1725 (m), 1607 (m), 1506 (m), 1466 (w), 1418 (s), 1351 (w), 1291 (w), 1269 (w), 1247 (m), 1204 (s), 1181 (m), 1138 (s), 1106 (s), 1031 (m), 878 (s), 817 (w), 775 (w), 744 (w), 713 (w), 615 (s), 576 (w), 545 (w), 502 (m), 457 (w) cm^{-1}.

HRMS (ESI): calcd for $C_{11}H_{11}F_3O_5SNa^+$ $[M+Na]^+$: 335.0171; found: 335.0169.

Preparation of alkene **S5**: To a suspension of isopropyltriphenylphosphonium iodide (1.05 g, 2.44 mmol, 1.45 equiv) in tetrahydrofuran (6.50 mL) was added a solution of *n*-butyllithium (1.60 M in hexanes, 1.42 mL, 2.27 mmol, 1.35 equiv) at –78 °C, upon which the yellow suspension turned orange-red. After stirring for 30 min at –78 °C, the reaction mixture was warmed to 23 °C through exchange of the cooling bath with a water bath. Stirring was continued for 30 min at 23 °C, during which the suspension turned red-brownish. The reaction mixture was cooled to –78 °C and a solution of aldehyde **S73** (525 mg, 1.68 mmol, 1 equiv) in tetrahydrofuran (3.20 mL) was added, which resulted in slight decolorization and immediate formation of an off-white solid. Stirring was continued for 60 min at –78 °C, followed by exchange of the cooling bath with a water bath and stirring for 3 h at 23 °C. Diethyl ether (25 mL) and a saturated aqueous solution of ammonium chloride (25 mL) were added, which resulted in decolorization of the reaction mixture. The biphasic mixture was filtered through celite and the filter cake was washed with diethyl ether (40 mL). The aqueous layer was separated and the organic layer was washed in succession with a saturated aqueous solution of sodium chloride (2 × 20 mL), an aqueous hydrogen peroxide solution (10 wt%, 2 × 20 mL), and a saturated aqueous solution of sodium chloride (2 × 20 mL). The washed organic layer was dried over sodium sulfate, the dried organic layer was filtered, and the filtrate was concentrated under reduced pressure. The residue was purified by flash column chromatography on silica gel (10% dichloromethane in *n*-pentane) to yield alkene **S5** (493 mg, 1.46 mmol, 87%) as a colorless oil.

<u>Analytical data of alkene **S5**:</u>

TLC (15% dichloromethane in *n*-pentane): R_f = 0.30 (UV, CAM).

^{1}H NMR (400 MHz, $CDCl_3$): δ 7.10 (d, *J* = 8.2 Hz, 1H), 6.84 (d, *J* = 2.0 Hz, 1H), 6.78 (dd, *J* = 8.3, 2.0 Hz, 1H), 5.14 (thept, *J* = 7.2, 1.4 Hz, 1H), 3.90 (s, 3H), 2.64 (dd, *J* = 8.6, 6.8 Hz, 2H), 2.30 (q, *J* = 7.5 Hz, 2H), 1.69 (q, *J* = 1.5 Hz, 3H), 1.54 (s, 3H).

^{13}C NMR (101 MHz, $CDCl_3$): δ 151.1, 144.3, 137.0, 133.0, 123.2, 122.1, 120.8, 118.9 (q, *J* = 320.4 Hz), 113.5, 56.2, 36.1, 29.9, 25.8, 17.8.

^{19}F NMR (376 MHz, $CDCl_3$) δ –73.9.

IR (ATR, neat): $\tilde{\nu}$ = 2969 (w), 2926 (w), 2860 (w), 1606 (w), 1505 (m), 1454 (w), 1419 (s), 1378 (w), 1333 (w), 1286 (m), 1248 (m), 1204 (s), 1178 (m), 1140 (s), 1106 (s), 1033 (m), 876 (s), 818 (m), 773 (w), 744 (w), 704 (w), 616 (s), 546 (w), 504 (m), 455 (w) cm^{-1}.

HRMS (ESI): calcd for $C_{14}H_{18}F_3O_4S^+$ $[M+H]^+$: 339.0872; found: 339.0871.

2.4.7 Alkene **S6**

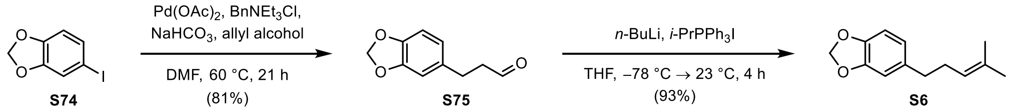

Aldehyde **S75** was prepared according to a modified literature procedure[12]: To a vial charged with palladium(II) acetate (26.7 mg, 119 µmol, 2.00 mol%), sodium bicarbonate (1.25 g, 14.9 mmol, 2.50 equiv), and benzyltriethylammonium chloride (1.36 g, 5.96 mmol, 1.00 equiv) was added successively a solution of 5-iodobenzo[d][1,3]dioxole (**S74**) (1.48 g, 5.96 mmol, 1 equiv) in degassed dimethylformamide (19.9 mL) and allyl alcohol (519 mg, 8.93 mmol, 1.50 equiv) at 23 °C under an argon atmosphere. The resulting orange suspension was heated to 60 °C. After stirring for 21 h at 60 °C, the reaction mixture was cooled to 23 °C, diluted with ethyl acetate (120 mL), and washed with a saturated aqueous solution of sodium chloride (3 × 30 mL). The washed organic layer was dried over sodium sulfate, the dried organic layer was filtered, and the filtrate was concentrated under reduced pressure. The residue was purified by flash column chromatography on silica gel (7% to 10% ethyl acetate in cyclohexane) to yield aldehyde **S75** (855 mg, 4.80 mmol, 81%) as a slightly yellowish oil. The obtained analytical data for aldehyde **S75** were in accordance with reported literature values.[12]

Preparation of alkene **S6**: To a suspension of isopropyltriphenylphosphonium iodide (2.90 g, 6.71 mmol, 1.45 equiv) in tetrahydrofuran (23.0 mL) was added a solution of *n*-butyllithium (1.60 M in hexanes, 3.91 mL, 6.25 mmol, 1.35 equiv) at –78 °C, upon which the suspension

turned from yellow to orange-red. After stirring for 30 min at –78 °C, the reaction mixture was warmed to 23 °C through exchange of the cooling bath with a water bath. Stirring was continued for 30 min at 23 °C, during which the suspension turned red-brownish. The reaction mixture was cooled to –78 °C and a solution of aldehyde **S75** (825 mg, 4.63 mmol, 1 equiv) in tetrahydrofuran (3.60 mL) was added resulting in slight decolorization and immediate formation of an off-white solid. Stirring was continued for 30 min at –78 °C, followed by exchange of the cooling bath with a water bath and stirring for 2.5 h at 23 °C. Next, diethyl ether (50 mL) and a saturated aqueous solution of ammonium chloride (60 mL) were added, which resulted in decolorization of the reaction mixture. The biphasic mixture was filtered through celite and the filter cake was washed with diethyl ether (50 mL). The aqueous layer was separated and the organic layer was successively washed with a saturated aqueous solution of sodium chloride (2 × 20 mL), an aqueous hydrogen peroxide solution (10 wt%, 2 × 20 mL), and a saturated aqueous solution of sodium chloride (2 × 20 mL). The washed organic layer was dried over sodium sulfate, the dried organic layer was filtered, and the filtrate was concentrated under reduced pressure. The residue was purified by flash column chromatography on silica gel (12% to 15% dichloromethane in *n*-pentane) to yield alkene **S6** (875 mg, 4.28 mmol, 93%) as a colorless oil. The obtained analytical data for alkene **S6** were in accordance with reported literature values.[13]

2.4.8 Alkene **S7**

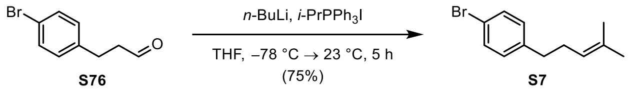

To a suspension of isopropyltriphenylphosphonium iodide (4.28 g, 9.90 mmol, 1.85 equiv) in tetrahydrofuran (26.7 mL) was added a solution of *n*-butyllithium (1.60 M in hexanes, 5.85 mL, 9.36 mmol, 1.75 equiv) at –78 °C, upon which the suspension turned from yellow to orange-red. After stirring for 30 min at –78 °C, the reaction mixture was warmed to 23 °C through exchange of the cooling bath with a water bath. Stirring was continued for 30 min at 23 °C, during which the suspension turned red-brownish. The reaction mixture was cooled to –78 °C and a solution of aldehyde **S76** (1.14 g, 5.35 mmol, 1 equiv) in tetrahydrofuran (4.20 mL) was added resulting in slight decolorization and immediate formation of an off-white solid. Stirring was continued for 45 min at –78 °C, followed by exchange of the cooling bath with a water bath and stirring for 3 h at 23 °C. Next, diethyl ether (50 mL) and a saturated aqueous solution of ammonium chloride (60 mL) were added, which resulted in decolorization of the reaction mixture. The biphasic mixture was filtered through celite and the filter cake was washed with diethyl ether (50 mL). The organic layer was separated and the aqueous layer was extracted

with diethyl ether (3 × 20 mL). The combined organic layers were dried over sodium sulfate, the dried organic layer was filtered, and the filtrate was concentrated under reduced pressure. The residue was purified by flash column chromatography on silica gel (1% dichloromethane in *n*-pentane) to yield alkene **S7** (992 mg, 4.15 mmol, 75%) as a colorless oil. The obtained analytical data for alkene **S7** were in accordance with reported literature values.[14]

2.4.9 Alkene **S8**

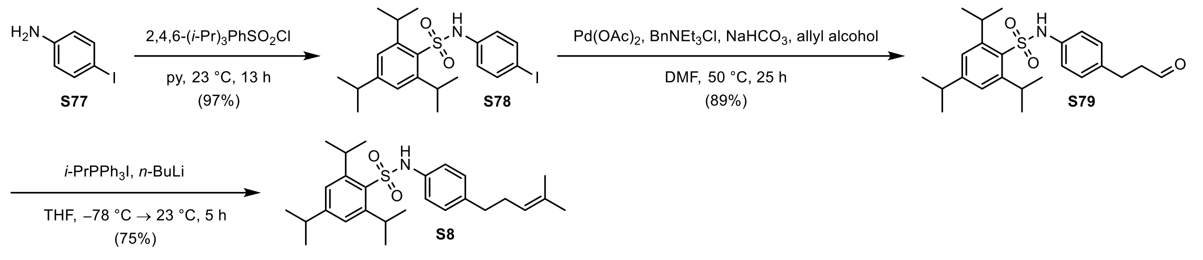

Preparation of sulfonamide **S78**: To a solution of 4-iodoaniline (**S77**) (2.00 g, 9.13 mmol, 1 equiv) in pyridine (46.0 mL) was added 2,4,6-triisopropylbenzenesulfonyl chloride (3.32 g, 11.0 mmol, 1.20 equiv) at 23 °C, whereupon the deep violet solution turned deep green. After stirring for 13 h at 23 °C, the reaction mixture was diluted with ethyl acetate (150 mL) and washed in succession with a 1 M aqueous solution of hydrochloric acid (4 × 50 mL) and a saturated aqueous solution of sodium chloride (50 mL). The washed organic layer was dried over sodium sulfate, the dried organic layer was filtered, silica (16 g) was added to the filtrate and the filtrate was concentrated under reduced pressure. The on silica adsorbed residue was purified by flash column chromatography on silica gel (10% ethyl acetate in cyclohexane) to yield sulfonamide **S78** (4.28 g, 8.81 mmol, 97%) as a white-orange solid.

Analytical data of sulfonamide **S78**:

TLC (10% ethyl acetate in cyclohexane): R_f = 0.32 (UV, CAM).

mp: 163–164 °C.

^{1}H NMR (400 MHz, $CDCl_3$): δ 7.55 – 7.48 (m, 2H), 7.18 (s, 1H), 7.15 (s, 2H), 6.80 – 6.73 (m, 2H), 4.12 (hept, *J* = 6.7 Hz, 2H), 2.89 (hept, *J* = 6.9 Hz, 1H), 1.24 (d, *J* = 6.9 Hz, 6H), 1.21 (d, *J* = 6.8 Hz, 12H).

^{13}C NMR (101 MHz, $CDCl_3$): δ 153.5, 150.7 (2C), 138.3 (2C), 136.8, 132.0, 124.2 (2C), 123.6 (2C), 89.0, 34.2, 30.0 (2C), 24.9 (4C), 23.6 (2C).

IR (ATR, neat): $\tilde{\nu}$ = 3248 (br), 2961 (w), 1590 (w), 1486 (w), 1461 (w), 1423 (w), 1385 (w), 1364 (w), 1323 (w), 1294 (w), 1279 (w), 1231 (w), 1148 (w), 1103 (w), 1059 (w), 1037 (w), 1005 (w), 904 (s), 816 (w), 804 (w), 727 (s), 650 (m), 569 (m), 551 (m), 499 (w), 429 (w) cm^{-1}.

HRMS (ESI): calcd for $C_{21}H_{28}INO_2SNa^+$ $[M+Na]^+$: 508.0778; found: 508.0772.

Preparation of aldehyde **S79**: To a vial charged with palladium(II) acetate (4.4 mg, 19 µmol, 1.0 mol%), sodium bicarbonate (0.40 g, 4.9 mmol, 2.5 equiv), and benzyltriethylammonium chloride (0.44 g, 2.0 mmol, 1.00 equiv) was added in succession a solution of sulfonamide **S78** (0.95 g, 2.0 mmol, 1 equiv) in degassed dimethylformamide (7.8 mL) and allyl alcohol (0.17 g, 2.9 mmol, 1.5 equiv) at 23 °C under an argon atmosphere. The resulting orange suspension was heated to 50 °C. After stirring for 25 h at 50 °C, the reaction mixture was diluted with ethyl acetate (80 mL) and washed with a saturated aqueous solution of sodium chloride (3 × 20 mL). The washed organic layer was dried over sodium sulfate, the dried organic layer was filtered, and the filtrate was concentrated under reduced pressure. The residue was purified by flash column chromatography on silica gel (10% to 20% ethyl acetate in cyclohexane) to yield aldehyde **S79** (0.72 g, 1.7 mmol, 89%) as a colorless oil.

Analytical data of aldehyde **S79**:

TLC (20% ethyl acetate in cyclohexane): R_f = 0.30 (UV, CAM).

^{1}H NMR (400 MHz, $CDCl_3$): δ 9.76 (t, *J* = 1.4 Hz, 1H), 7.12 (s, 2H), 7.05 (d, *J* = 8.4 Hz, 2H), 6.93 (d, *J* = 8.4 Hz, 2H), 6.79 (s, 1H), 4.04 (hept, *J* = 6.8 Hz, 2H), 2.93 – 2.84 (m, 3H), 2.70 (tt, *J* = 7.5, 1.1 Hz, 2H), 1.23 (d, *J* = 7.0 Hz, 6H), 1.16 (d, *J* = 6.8 Hz, 12H).

^{13}C NMR (101 MHz, $CDCl_3$): δ 201.5, 153.1, 150.6 (2C), 138.1, 134.7, 132.4, 129.2 (2C), 124.0 (2C), 123.8 (2C), 45.2, 34.2, 30.0 (2C), 27.5, 24.8 (4C), 23.6 (2C).

IR (ATR, neat): $\tilde{\nu}$ = 3274 (br), 2959 (m), 2929 (w), 2869 (w), 2727 (w), 1718 (m), 1600 (w), 1563 (w), 1512 (m), 1461 (m), 1424 (w), 1385 (m), 1362 (m), 1326 (m), 1256 (w), 1225 (w), 1194 (w), 1149 (s), 1106 (w), 1070 (w), 1059 (w), 1038 (w), 1020 (w), 940 (w), 907 (s), 883 (m), 845 (w), 729 (s), 659 (s), 623 (w), 581 (s), 556 (s) cm^{-1}.

HRMS (ESI): calcd for $C_{24}H_{33}NO_3SNa^+$ $[M+Na]^+$: 438.2073; found: 438.2063.

Preparation of alkene **S8**: To a suspension of isopropyltriphenylphosphonium iodide (1.69 g, 3.91 mmol, 2.60 equiv) in tetrahydrofuran (7.50 mL) was added a solution of *n*-butyllithium (1.60 M in hexanes, 2.35 mL, 3.76 mmol, 2.50 equiv) at –78 °C, upon which the suspension

turned from yellow to orange-red. After stirring for 60 min at –78 °C, the reaction mixture was warmed to 23 °C through exchange of the cooling bath with a water bath. Stirring was continued for 45 min at 23 °C, during which the suspension turned red-brownish. The reaction mixture was cooled to –78 °C and a solution of aldehyde **S79** (624 mg, 1.50 mmol, 1 equiv) in tetrahydrofuran (1.20 mL) was added resulting in slight decolorization and immediate formation of an off-white solid. Stirring was continued for 2 h at –78 °C, followed by exchange of the cooling bath with a water bath and stirring for 1.5 h at 23 °C. Next, a saturated aqueous solution of ammonium chloride (30 mL) was added, which resulted in decolorization of the reaction mixture. The organic layer was separated and the aqueous layer was extracted with diethyl ether (3 × 20 mL). The combined organic layers were dried over sodium sulfate, the dried organic layer was filtered, and the filtrate was concentrated under reduced pressure. The residue was purified by flash column chromatography on silica gel (20% diethyl ether in *n*-pentane) to yield alkene **S8** (494 mg, 1.12 mmol, 75%) as a white solid.

Analytical data of alkene **S8**:

TLC (20% diethyl ether in *n*-pentane): R_f = 0.47 (UV, CAM).

mp: 136–137 °C.

^{1}H NMR (400 MHz, $CDCl_3$): δ 7.11 (s, 2H), 7.05 (d, *J* = 8.3 Hz, 2H), 6.91 (d, *J* = 8.4 Hz, 2H), 6.25 (s, 1H), 5.08 (t, *J* = 7.1 Hz, 1H), 3.98 (hept, *J* = 6.8 Hz, 2H), 2.88 (hept, *J* = 6.9 Hz, 1H), 2.54 (dd, *J* = 8.7, 6.8 Hz, 2H), 2.20 (q, *J* = 7.5 Hz, 2H), 1.65 (s, 3H), 1.51 (s, 3H), 1.24 (d, *J* = 6.9 Hz, 6H), 1.16 (d, *J* = 6.8 Hz, 12H).

^{13}C NMR (101 MHz, $CDCl_3$): δ 153.1, 150.7 (2C), 140.7, 133.8, 132.5, 132.4, 129.4 (2C), 124.3 (2C), 124.0 (2C), 123.5, 35.6, 34.2, 30.1 (2C), 30.0, 25.8, 24.9 (4C), 23.7 (2C), 17.8.

IR (ATR, neat): $\tilde{\nu}$ = 3270 (br), 2960 (s), 2926 (m), 2868 (m), 1600 (w), 1511 (m), 1461 (m), 1425 (w), 1384 (m), 1363 (w), 1326 (m), 1226 (w), 1150 (s), 1106 (w), 1039 (w), 941 (w), 912 (w), 883 (w), 825 (w), 660 (m), 559 (m) cm^{-1}.

HRMS (ESI): calcd for $C_{27}H_{39}NO_2SNa^+$ $[M+Na]^+$: 464.2594; found: 464.2588.

2.4.10 Alkene **S9**

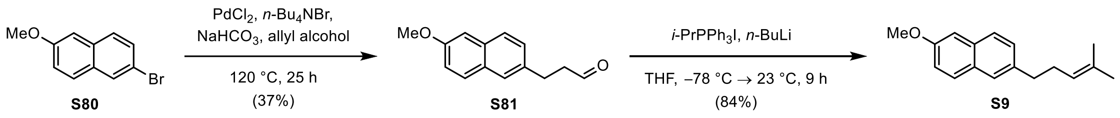

Aldehyde **S81** was prepared according to a modified literature procedure[15]: Palladium chloride (93.5 mg, 527 μmol, 0.104 equiv) and tetrabutylammonium bromide (7.68 g, 23.8 mmol, 4.70 equiv) were heated at 120 °C for 50 min under an argon atmosphere to afford a homogenous red liquid. The homogenous mixture was cooled down to 80 °C and allyl alcohol (324 mg, 5.57 mmol, 1.10 equiv), bromide **S80** (1.20 g, 5.07 mmol, 1 equiv), and sodium bicarbonate (511 mg, 6.08 mmol, 1.20 equiv) were added in succession. Stirring was continued for 25 h at 120 °C, after which the resulting deep red suspension was cooled to 23 °C and diluted with dichloromethane (100 mL) and diethyl ether (100 mL). The diluted reaction mixture was washed with a saturated aqueous solution of sodium chloride (2 × 150 mL), the washed organic layer was dried over sodium sulfate, the dried organic layer was filtered, and the filtrate was concentrated under reduced pressure. The residue was purified by flash column chromatography on silica gel (10% to 15% diethyl ether in *n*- pentane) to yield aldehyde **S81** (403 mg, 1.88 mmol, 37%) as a white solid. The obtained analytical data for aldehyde **S81** were in accordance with reported literature.[15]

Preparation of alkene **S9**: To a suspension of isopropyltriphenylphosphonium iodide (1.49 g, 3.44 mmol, 1.85 equiv) in tetrahydrofuran (9.30 mL) was added a solution of *n*-butyllithium (1.60 M in hexanes, 2.03 mL, 3.25 mmol, 1.75 equiv) at –78 °C, upon which the yellow suspension turned orange-red. After stirring for 30 min at –78 °C, the reaction mixture was warmed to 23 °C through exchange of the cooling bath with a water bath. After stirring for 30 min at 23 °C, the reaction mixture was cooled to –78 °C and a solution of aldehyde **S81** (398 mg, 1.86 mmol, 1 equiv) in tetrahydrofuran (1.50 mL) was added at –78 °C. After stirring for 30 min at –78 °C, the reaction mixture was warmed to 23 °C through exchange of the cooling bath with a water bath. After stirring for 7 h at 23 °C, the suspension was diluted with a saturated aqueous solution of ammonium chloride (40 mL) and diethyl ether (40 mL), which resulted in decolorization of the reaction mixture. The resulting suspension was filtered through a glass sinter filter and the remaining solid was washed with diethyl ether (30 mL) before being discharged. The organic layer was separated and the aqueous layer was extracted with diethyl ether (2 × 30 mL). The combined organic layers were dried over sodium sulfate, the dried organic layer was filtered, and the filtrate was concentrated under reduced pressure. The residue was purified by flash column chromatography on silica gel (10% to 20% dichloromethane in *n*-pentane) to yield alkene **S9** (375 mg, 1.56 mmol, 84%) as a white solid.

<u>Analytical data of alkene **S9**:</u>

TLC (20% dichloromethane in *n*-pentane): R_f = 0.43 (UV, CAM).

mp: 58–59 °C.

^{1}H NMR (400 MHz, $CDCl_3$): δ 7.70 – 7.65 (m, 2H), 7.56 (s, 1H), 7.32 (dd, *J* = 8.4, 1.8 Hz, 1H), 7.15 – 7.11 (m, 2H), 5.22 (thept, *J* = 7.2, 1.4 Hz, 1H), 3.92 (s, 3H), 2.77 (dd, *J* = 9.5, 7.4 Hz, 2H), 2.38 (q, *J* = 7.6 Hz, 2H), 1.70 (q, *J* = 1.4 Hz, 3H), 1.59 (s, 3H).

^{13}C NMR (101 MHz, $CDCl_3$): δ 157.2, 137.8, 133.1, 132.3, 129.2, 129.1, 128.1, 126.7, 126.4, 123.9, 118.7, 105.8, 55.4, 36.2, 30.2, 25.8, 17.8.

IR (ATR, neat): $\tilde{\nu}$ = 3054 (w), 2963 (w), 2913 (s), 2853 (w), 1634 (w), 1605 (s), 1505 (w), 1483 (s), 1452 (m), 1439 (m), 1415 (m), 1390 (w), 1376 (s), 1325 (s), 1265 (w), 1228 (w), 1196 (w), 1173 (s), 1160 (s), 1119 (w), 1104 (m), 1033 (m), 984 (m), 957 (w), 930 (s), 918 (w), 885 (w), 849 (w),809 (w), 757 (s), 687(m), 675 (w), 661 (w), 615 (w), 571 (w), 542 (w), 475 (w), 441 (s) cm^{-1}.

HRMS (ESI): calcd for $C_{17}H_{21}O^+$ $[M+H]^+$: 241.1587; found: 241.1583.

2.4.11 Phosphonium salt **S83**

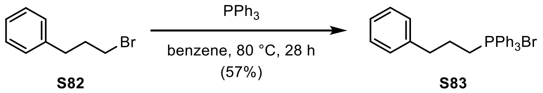

Phosphonium salt **S83** was prepared according to a modified literature procedure[16]: To a solution of 3-bromopropyl benzene (**S82**) (2.50 g, 12.6 mmol, 1 equiv) in benzene (13.0 mL) was added triphenylphosphine (3.29 g, 12.6 mmol, 1.00 equiv) at 23 °C. The reaction mixture was heated to 80 °Cand stirred for 28 h. The resulting suspension was cooled to 23 °C and diluted with diethyl ether (30 mL). The white solid material was filtered off and washed successively with diethyl ether (2 × 30 mL) and *n*-pentane (2 × 100 mL). The remaining solid was dried under reduced pressure to afford phosphonium salt **S83** (3.30 g, 7.16 mmol, 57%) as a white solid. The obtained analytical data for phosphonium salt **S83** were in accordance with reported literature values.[16]

2.4.12 Alkene **S10**

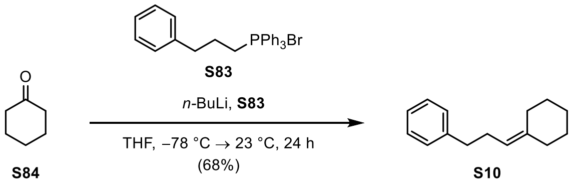

Alkene **S10** was prepared according to a modified literature procedure[17]: To a suspension of phosphonium salt **S83** (739 mg, 1.60 mmol, 1.85 equiv) in tetrahydrofuran (4.30 mL) was

added a solution of *n*-butyllithium (1.60 M in hexanes, 974 µL, 1.52 mmol, 1.75 equiv) at –78 °C, upon which the yellow suspension turned orange. After stirring for 3 h at –78 °C, the reaction mixture was warmed to 23 °C through exchange of the cooling bath with a water bath. After stirring for 20 min at 23 °C, the reaction mixture was cooled to –78 °C and a solution of cyclohexanone (**S84**) (85.0 mg, 866 µmol, 1 equiv) in tetrahydrofuran (700 µL) was added. After stirring for 30 min at –78 °C, the reaction mixture was warmed to 23 °C through exchange of the cooling bath with a water bath. After stirring for 20 h at 23 °C, a saturated aqueous solution of ammonium chloride (10 mL) was added, which resulted in decolorization of the reaction mixture. The resulting suspension was filtered through a glass sinter filter and the remaining solid was washed with diethyl ether (10 mL) before being discharged. The organic layer was separated and the aqueous layer was extracted with diethyl ether (4 × 10 mL). The combined organic layers were dried over sodium sulfate, the dried organic layer was filtered, and the filtrate was concentrated under reduced pressure. The residue was purified by flash column chromatography on silica gel (100% *n*-pentane) to yield alkene **S10** (119 mg, 592 µmol, 68%) as a colorless oil. The obtained analytical data for alkene **S10** were in accordance with reported literature values.[17]

2.4.13 Phosphonium salt **S87**

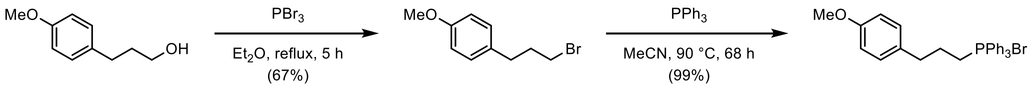

S85 **S86** **S87**

Alkyl bromide **S86** was prepared according to a modified literature procedure[18]: To a solution of 3-(4-methoxyphenyl)propan-1-ol (**S85**) (8.08 g, 48.6 mmol, 1 equiv) in diethyl ether (185 mL) was added phosphorus tribromide (4.87 g, 18.0 mmol, 0.370 equiv) at 0 °C. The resulting reaction mixture was heated at reflux for 5 h (oil bath temperature of 40 °C), after which the reaction mixture was allowed to cool to 23 °C and water (150 mL) was added. The organic layer was separated and the aqueous layer was extracted with diethyl ether (3 × 100 mL). The combined organic layers were washed in succession with a saturated aqueous solution of sodium bicarbonate (100 mL) and a saturated aqueous solution of sodium chloride (100 mL). The washed organic layer was dried over sodium sulfate, the dried organic layer was filtered, and the filtrate was concentrated under reduced pressure. The residue was purified by flash column chromatography on silica gel (10% diethyl ether in *n*-pentane) to yield alkyl bromide **S86** (7.42 g, 32.4 mmol, 67%) as a colorless oil. The obtained analytical data for alkyl bromide **S86** were in accordance with reported literature values.[18]

Wittig salt **S87** was prepared according to a modified literature procedure[19]: A solution of triphenylphosphine (12.9 g, 49.2 mmol, 1.50 equiv) and bromide **S86** (7.52 g, 32.8 mmol, 1 equiv) in acetonitrile (32.8 ml) was heated to 90 °C in a pressure tube. After stirring for 68 h at 90 °C, the reaction mixture was cooled to 23 °C and the solvent was removed under reduced pressure. The remaining white solid was washed successively with diethyl ether (2 × 30 mL), *n*-pentane (30 mL), and dichloromethane (3 × 20 mL). The washed solid was dried under reduced pressure to afford phosphonium salt **S87** (16.0 g, 32.6 mmol, 99%) as a withe solid. The obtained analytical data for phosphonium salt **S87** were in accordance with reported literature values.[19]

2.4.14 Alkene **S11**

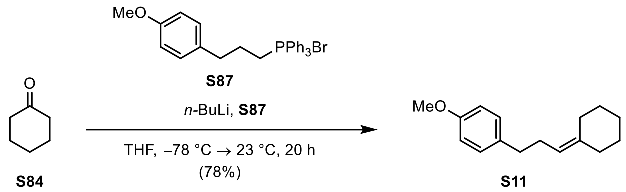

Preparation of alkene **S11**: To a suspension of phosphonium salt **S87** (3.01 g, 6.12 mmol, 1.35 equiv) in tetrahydrofuran (23.0 mL) was added a solution of *n*-butyllithium (1.60 M in hexanes, 3.68 mL, 5.89 mmol, 1.30 equiv) at –78 °C, upon which the white suspension turned pumpkin orange. After stirring for 30 min at –78 °C, the reaction mixture was warmed to 23 °C through exchange of the cooling bath with a water bath. After stirring for 40 min at 23 °C, the reaction mixture was cooled to –78 °C and a solution of cyclohexanone (**S84**) (445 mg, 4.53 mmol, 1 equiv) in tetrahydrofuran (3.50 mL) was added, which resulted in a slight color change from orange to bright orange. After stirring for 1 h at –78 °C, the reaction mixture was warmed to 23 °C by exchange of the cooling bath with a water bath. After stirring for 17 h at 23 °C, a saturated aqueous solution of ammonium chloride (25 mL) and diethyl ether (25 mL) were added, which resulted in decolorization of the reaction mixture. The resulting suspension was filtered through a glass sinter filter and the remaining solid was washed with diethyl ether (30 mL) before being discharged. The organic layer was separated and the aqueous layer was extracted with diethyl ether (3 × 30 mL). The combined organic layers were washed successively with a saturated aqueous solution of sodium chloride (2 × 30 mL), an aqueous hydrogen peroxide solution (10 wt%, 3 × 30 mL), and a saturated aqueous solution of sodium chloride (3 × 30 mL). The washed organic layer was dried over sodium sulfate, the dried organic layer was filtered, and the filtrate was concentrated under reduced pressure. The residue was purified by flash column chromatography on silica gel (15% dichloromethane in *n*-pentane) to yield alkene **S11** (811 mg, 3.52 mmol, 78%) as a colorless oil.

Analytical data of alkene **S11**:

TLC (10% dichloromethane in pentane): R_f = 0.29 (UV, CAM).

^{1}H NMR (400 MHz, $CDCl_3$): δ 7.10 (d, *J* = 8.6 Hz, 2H), 6.82 (d, *J* = 8.6 Hz, 2H), 5.11 (tt, *J* = 7.3, 1.3 Hz, 1H), 3.79 (s, 3H), 2.57 (dd, *J* = 9.0, 7.4 Hz, 2H), 2.27 (q, *J* = 7.5 Hz, 2H), 2.09 – 2.02 (m, 4H), 1.53 – 1.46 (m, 4H), 1.45 – 1.36 (m, 2H).

^{13}C NMR (101 MHz, $CDCl_3$): δ 157.8, 140.4, 134.7 (2C), 129.5, 120.5, 113.8 (2C), 55.4, 37.3, 35.7, 29.4, 28.8, 28.8, 27.8, 27.1.

IR (ATR, neat): $\tilde{\nu}$ = 2995 (s), 2923 (m), 2852 (m), 2834 (m), 1612 (w), 1584 (w), 1511 (s), 1445 (m), 1300 (w), 1244 (s), 1176 (m), 1105 (m), 1039 (m), 819 (m), 664 (w), 523 (w) cm^{-1}.

HRMS (ESI): calcd for $C_{16}H_{23}O^+$ $[M+H]^+$: 231.1743; found: 231.1741.

2.4.15 Alkene **S12**

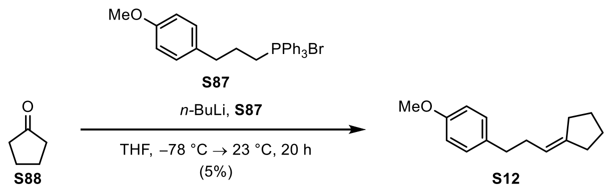

Preparation of alkene **S12**: To a suspension of phosphonium salt **S87** (2.35 g, 4.78 mmol, 1.40 equiv) in tetrahydrofuran (17.0 mL) was added a solution of *n*-butyllithium (1.60 M in hexanes, 2.88 mL, 4.61 mmol, 1.35 equiv) at –78 °C, upon which the white suspension turned yellow. After stirring for 1.5 h at –78 °C, the reaction mixture was warmed to 23 °C through exchange of the cooling bath with a water bath. After stirring for 30 min at 23 °C, the red reaction mixture was cooled to –78 °C and a solution of cyclopentanone (**S88**) (287 mg, 3.41 mmol, 1 equiv) in tetrahydrofuran (2.70 mL) was added, which resulted in a color change to orange and immediate formation of an off-white solid. After stirring for 1 h at –78 °C, the reaction mixture was warmed to 23 °C through exchange of the cooling bath with a water bath. After stirring for 17 h at 23 °C, a saturated aqueous solution of ammonium chloride (25 mL) was added, which resulted in decolorization of the reaction mixture. The resulting suspension was filtered through a glass sinter filter and the remaining solid was washed with diethyl ether (30 mL) before being discharged. The organic layer was separated and the aqueous layer was extracted with diethyl ether (3 × 20 mL). The combined organic layers were washed successively with a saturated aqueous solution of sodium chloride (2 × 30 mL), an aqueous hydrogen peroxide solution (10 wt%, 3 × 30 mL) and a saturated aqueous solution of sodium chloride (3 × 30 mL). The washed organic layer was dried over sodium sulfate, the dried

organic layer was filtered, and the filtrate was concentrated under reduced pressure. The residue was purified by flash column chromatography on silica gel (20% dichloromethane in *n*-pentane) to yield alkene **S12** (34.6 mg, 160 µmol, 5%) as a colorless oil. The obtained analytical data for alkene **S12** were in accordance with reported literature values.[20]

2.4.16 Piperidine **S13**

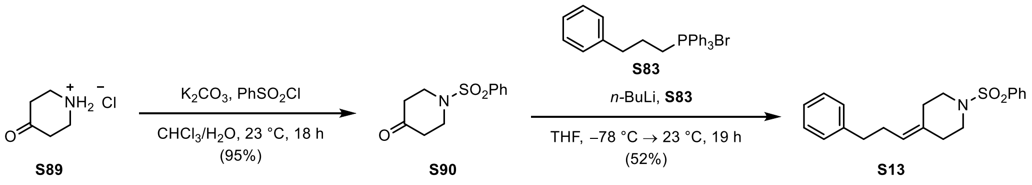

Sulfonamide **S90** was prepared according to a modified literature procedure[21]: To a suspension of 4-piperidone hydrochloride (**S89**) (1.00 g, 7.38 mmol, 1 equiv) and potassium carbonate (2.61 g, 18.9 mmol, 2.56 equiv) in chloroform (8.80 mL) and water (8.80 mL) was added benzenesulfonyl chloride (2.03 g, 11.5 mmol, 1.56 equiv) at 23 °C. After stirring for 18 h at 23 °C, the reaction mixture was diluted with ethyl acetate (60 mL). The aqueous layer was separated and the organic layer was washed successively with a 1 M aqueous solution of sodium hydroxide (30 mL), a saturated aqueous solution of sodium bicarbonate (3 × 20 mL), and a saturated aqueous solution of sodium chloride (20 mL). The washed organic layer was dried over sodium sulfate, the dried organic layer was filtered, and the filtrate was concentrated under reduced pressure. The residue was purified by flash column chromatography on silica gel (40% ethyl acetate in cyclohexane) to yield sulfonamide **S90** (1.67 g, 6.98 mmol, 95%) as a white solid. The obtained analytical data for sulfonamide **S90** were in accordance with reported literature values.[21]

Preparation of piperidine **S13**: To a suspension of phosphonium salt **S83** (2.14 g, 4.64 mmol, 1.85 equiv) in tetrahydrofuran (12.5 mL) was added a solution of *n*-butyllithium (1.60 M in hexanes, 2.74 mL, 4.39 mmol, 1.75 equiv) at –78 °C, upon which the white suspension turned orange. After stirring for 1 h at –78 °C, the reaction mixture was warmed to 23 °C through exchange of the cooling bath with a water bath. After stirring for 20 min at 23 °C, the red reaction mixture was cooled to –78 °C and a solution of sulfonamide **S90** (600 mg, 2.51 mmol, 1 equiv) in tetrahydrofuran (2.00 mL) was added. Stirring was continued for 30 min at –78 °C, after which the reaction mixture was warmed to 23 °C by exchange of the cooling bath with a water bath. After stirring for 17 h at 23 °C, the pink suspension was diluted with a saturated aqueous solution of ammonium chloride (15 mL), which resulted in decolorization of the reaction mixture. The organic layer was separated and the aqueous layer was extracted with diethyl ether (3 × 20 mL). The combined organic layers were dried over sodium sulfate, the

dried organic layer was filtered, and the filtrate was concentrated under reduced pressure. The residue was purified by flash column chromatography on silica gel (15% diethyl ether in *n*-pentane) to yield piperidine **S13** (446 mg, 1.31 mmol, 52%) as a colorless oil.

Analytical data of piperidine **S13**:

TLC (40% diethyl ether in *n*-pentane): R_f = 0.69 (UV, CAM).

^{1}H NMR (400 MHz, $CDCl_3$): δ 7.75 – 7.70 (m, 2H), 7.65 – 7.59 (m, 1H), 7.58 – 7.51 (m, 2H), 7.15 – 7.09 (m, 2H), 7.09 – 7.00 (m, 3H), 5.18 (t, *J* = 7.5 Hz, 1H), 2.96 (t, *J* = 5.7 Hz, 2H), 2.67 (t, *J* = 5.8 Hz, 2H), 2.58 (t, *J* = 7.3 Hz, 2H), 2.29 – 2.20 (m, 4H), 2.13 (t, *J* = 5.7 Hz, 2H).

^{13}C NMR (101 MHz, $CDCl_3$): δ 141.6, 136.4, 134.1, 132.7, 129.1 (2C), 128.7 (2C), 128.1 (2C), 127.7 (2C), 125.8, 123.7, 48.1, 46.9, 35.9, 35.4, 29.1, 27.7.

IR (ATR, neat): $\tilde{\nu}$ = 3061 (w), 3025 (w), 2908 (w), 2852 (w), 1602 (w), 1495 (w), 1478 (w), 1465 (w), 1445 (m), 1352 (m), 1336 (s), 1248 (w), 1234 (w), 1166 (s), 1103 (m), 1091 (w), 1073 (w), 1039 (w), 1010 (w), 981 (w), 930 (m), 877 (w), 849 (w), 738 (s), 691 (s), 653 (w), 574 (s) cm^{-1}.

HRMS (ESI): calcd for $C_{20}H_{23}NO_2SNa^+$ $[M+Na]^+$: 364.1342; found: 364.1333.

2.4.17 Alkene **18**

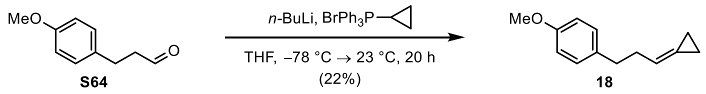

Preparation of cyclopropane **18**: To a suspension of cyclopropyltriphenylphosphonium bromide (2.91 g, 7.60 mmol, 1 equiv) in tetrahydrofuran (27.0 mL) was added a solution of *n*-butyllithium (1.60 M in hexanes, 4.58 mL, 7.33 mmol, 1.35 equiv) at –78 °C, upon which the white suspension turned orange. After stirring for 30 min at –78 °C, the reaction mixture was warmed to 23 °C through exchange of the cooling bath with a water bath. After stirring for 30 min at 23 °C, the reaction mixture was cooled to –78 °C and a solution of 3-(4-methoxyphenyl)propanal (**S64**) (891 mg, 5.43 mmol, 1 equiv) in tetrahydrofuran (4.20 mL) was added, which resulted in slight decolorization and immediate formation of an off-white solid. After stirring for 30 min at –78 °C, the reaction mixture was warmed to 23 °C through exchange of the cooling bath with a water bath. After stirring for 19 h at 23 °C, a saturated aqueous solution of ammonium chloride (25 mL) was added, which resulted in decolorization of the reaction mixture. The resulting suspension was filtered through a glass sinter filter and the remaining solid was washed with diethyl ether (10 mL) before being discharged. The

organic layer was separated, and the aqueous layer was extracted with diethyl ether (3 × 30 mL). The combined organic layers were dried over sodium sulfate, the dried organic layer was filtered, and the filtrate was concentrated under reduced pressure. The residue was purified by flash column chromatography on silica gel (15% dichloromethane in *n*-pentane) to yield alkene **18** (227 mg, 1.20 mmol, 22%) as a colorless oil.

Analytical data of alkene **18**:

TLC (10% dichloromethane in pentane): R_f = 0.29 (UV, CAM).

^{1}H NMR (400 MHz, $CDCl_3$): δ 7.12 (d, *J* = 8.6 Hz, 2H), 6.82 (d, *J* = 8.6 Hz, 2H), 5.80 (tp, *J* = 6.2, 1.8 Hz, 1H), 3.79 (s, 3H), 2.71 (t, *J* = 8.0 Hz, 2H), 2.51 – 2.42 (m, 2H), 1.05 – 0.92 (m, 4H).

^{13}C NMR (101 MHz, $CDCl_3$): δ 157.8, 134.5 (2C), 129.5 (2C), 122.0, 117.6, 113.8, 55.4, 35.0, 34.0, 2.3, 2.0.

IR (ATR, neat): $\tilde{\nu}$ = 3050 (w), 2978 (w), 2931 (w), 2834 (w), 1611 (w), 1584 (w), 1511 (s), 1464 (w), 1454 (w), 1441 (w), 1300 (w), 1244 (s), 1177 (m), 1109 (w), 1038 (m) , 958 (w), 931 (w), 822 (m), 782 (w), 747 (w), 694 (w), 563 (w), 519 (w) cm^{-1}.

HRMS (ESI): calcd for $C_{13}H_{17}O^+$ [M+H]$^+$: 189.1274; found: 189.1273.

2.4.18 Alkene **S14**

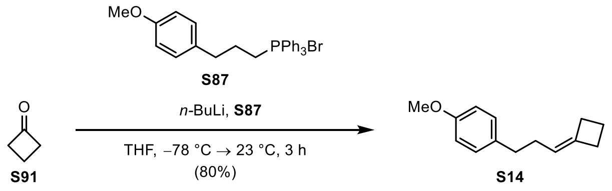

Preparation of alkene **S14**: To a suspension of phosphonium salt **S87** (2.92 g, 5.93 mmol, 1.40 equiv) in tetrahydrofuran (21.0 mL) was added a solution of *n*-butyllithium (1.60 M in hexanes, 3.58 mL, 5.72 mmol, 1.35 equiv) at –78 °C, upon which the white suspension turned yellow. After stirring for 30 min at –78 °C, the reaction mixture was warmed to 23 °C through exchange of the cooling bath with a water bath. After stirring for 30 min at 23 °C, the red reaction mixture was cooled to –78 °C and a solution of cyclobutanone (**S91**) (297 mg, 4.24 mmol, 1 equiv) in tetrahydrofuran (3.30 mL) was added, which resulted in a color change to orange and immediate formation of an off-white solid. After stirring for 1 h at –78 °C, the reaction mixture was warmed to 23 °C through exchange of the cooling bath with a water bath. After stirring for 1 h at 23 °C, a saturated aqueous solution of ammonium chloride (25 mL) was

added, which resulted in decolorization of the reaction mixture. The resulting suspension was filtered through a glass sinter filter and the remaining solid was washed with diethyl ether (30 mL) before being discharged. The organic layer was separated, and the aqueous layer was extracted with diethyl ether (3 × 20 mL). The combined organic layers were washed successively with a saturated aqueous solution of sodium chloride (2 × 30 mL), an aqueous hydrogen peroxide solution (10 wt%, 3 × 30 mL) and a saturated aqueous solution of sodium chloride (3 × 30 mL). The washed organic layer was dried over sodium sulfate, the dried organic layer was filtered, and the filtrate was concentrated under reduced pressure. The residue was purified by flash column chromatography on silica gel (10% dichloromethane in *n*-pentane) to yield alkene **S14** (682 mg, 3.37 mmol, 80%) as a colorless oil.

Analytical data of alkene **S14**:

TLC (10% dichloromethane in pentane): R_f = 0.29 (UV, CAM).

^{1}H NMR (400 MHz, $CDCl_3$): δ 7.10 (d, *J* = 8.6 Hz, 2H), 6.82 (d, *J* = 8.6 Hz, 2H), 5.08 (tp, *J* = 7.0, 2.2 Hz, 1H), 3.79 (s, 3H), 2.65 – 2.59 (m, 2H), 2.59 – 2.52 (m, 4H), 2.15 (qt, *J* = 7.5, 1.4 Hz, 2H), 1.90 (p, *J* = 7.9 Hz, 2H).

^{13}C NMR (101 MHz, $CDCl_3$): δ 157.8, 140.8, 134.6, 129.5 (2C), 119.6, 113.8 (2C), 55.4, 35.3, 31.0, 30.3, 29.4, 17.2.

IR (ATR, neat): $\tilde{\nu}$ = 2916 (m), 2834 (w), 1612 (w), 1584 (w), 1511 (s), 1464 (w), 1441 (s), 1300 (w), 1245 (s), 1176 (m), 1112 (w), 1039 (m), 821 (w) cm^{-1}.

HRMS (ESI): calcd for $C_{14}H_{19}O^+$ [M+H]$^+$: 203.1430; found: 203.1427.

2.4.19 Alkene **24** and aldehyde **S41**

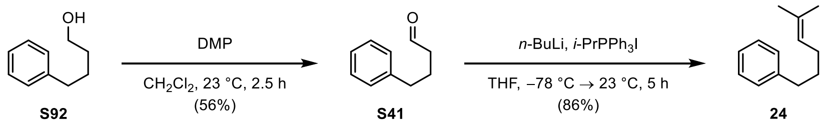

4-Phenylbutanal (**S41**) was prepared according to a modified literature procedure[22]: To a suspension of Dess–Martin periodinane (2.94 g, 6.92 mmol, 1.30 equiv) in dichloromethane (21.3 mL) was added 4-phenylbutan-1-ol (**S92**) (800 mg, 5.33 mmol, 1 equiv) at 23 °C. After stirring for 2 h 30 min at 23 °C, diethyl ether (50 mL) and a saturated aqueous solution of sodium hydrogencarbonate (30 mL) were added. The organic layer was separated and the aqueous layer was extracted with diethyl ether (3 × 30 mL). The combined organic layers were washed in succession with a saturated aqueous solution of sodium hydrogencarbonate (40 mL), water (40 mL), and a saturated aqueous solution of sodium chloride (40 mL). The

washed organic layer was dried over sodium sulfate, the dried organic layer was filtered, and the filtrate was concentrated under reduced pressure. The residue was purified by flash column chromatography on silica gel (10% diethyl ether in *n*-pentane) to yield 4-phenylbutanal (**S41**) (442 mg, 2.98 µmol, 56%) as a colorless oil. The obtained analytical data for 4-phenylbutanal (**S41**) were in accordance with reported literature values.[22]

Alkene **24** was prepared according to a modified literature procedure[23]: To a suspension of isopropyltriphenylphosphonium iodide (2.40 g, 5.55 mmol, 1.85 equiv) in tetrahydrofuran (15.0 mL) was added a solution of *n*-butyllithium (2.50 M in hexanes, 2.10 mL, 5.25 mmol, 1.75 equiv) at –78 °C, upon which the suspension turned from yellow to orange-red. After stirring for 30 min at –78 °C, the reaction mixture was warmed to 23 °C through exchange of the cooling bath with a water bath. Stirring was continued for 30 min at 23 °C, during which the suspension turned red-brownish. The reaction mixture was cooled to –78 °C and a solution of 4-phenylbutanal (**S41**) (445 mg, 3.00 mmol, 1 equiv) in tetrahydrofuran (2.40 mL) was added resulting in slight decolorization and immediate formation of an off-white solid. Stirring was continued for 30 min at –78 °C, followed by exchange of the cooling bath with a water bath and stirring for 2 h at 23 °C. Next, a saturated aqueous solution of ammonium chloride (60 mL) was added, which resulted in decolorization of the reaction mixture. The organic layer was separated and the aqueous layer was extracted with ethyl acetate (2 × 50 mL). The combined organic layers were dried over sodium sulfate, the dried organic layer was filtered, and the filtrate was concentrated under reduced pressure. The residue was purified by flash column chromatography on silica gel (*n*-pentane) to yield alkene **24** (449 mg, 2.58 µmol, 86%) as a colorless oil. The obtained analytical data for alkene **24** were in accordance with reported literature values.[23]

2.4.20 Alkene **S15**

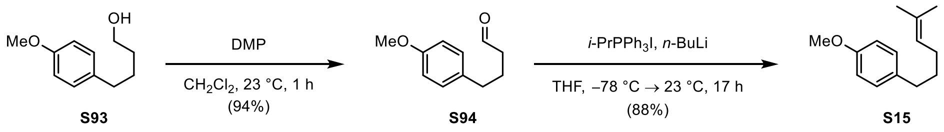

Aldehyde **S94** was prepared according to a modified literature procedure[24]: To a solution of 4-(4-methoxyphenyl)butan-1-ol (**S93**) (1.18 g, 6.56 mmol, 1 equiv) in dichloromethane (22.0 mL) was added Dess–Martin periodinane (3.30 g, 7.78 mmol, 1.19 equiv) at 23 °C. After stirring for 1 h at 23 °C, a saturated aqueous solution of sodium thiosulfate (25 mL) was added, the organic layer was separated, and the aqueous layer was extracted with dichloromethane (3 × 15 mL). The combined organic layers were washed in succession with a saturated aqueous solution of sodium thiosulfate (2 × 20 mL), a saturated aqueous solution of sodium

bicarbonate (2 × 20 mL), and a saturated aqueous solution of sodium chloride (20 mL). The washed organic layer was dried over sodium sulfate, the dried organic layer was filtered, and the filtrate was concentrated under reduced pressure. The residue was purified by flash column chromatography on silica gel (15% diethyl ether in *n*-pentane) to yield aldehyde **S94** (1.10 g, 6.17 mmol, 94%) as a colorless oil. The obtained analytical data for aldehyde **S94** were in accordance with reported literature values.[24]

Alkene **S15** was prepared according to a modified literature procedure[23]: To a suspension of isopropyltriphenylphosphonium iodide (3.76 g, 8.70 mmol, 1.45 equiv) in tetrahydrofuran (30.0 mL) was added a solution of *n*-butyllithium (1.60 M in hexanes, 5.06 mL, 8.10 mmol, 1.35 equiv) at –78 °C, upon which the yellow suspension turned red. After stirring for 30 min at –78 °C, the reaction mixture was warmed to 23 °C through exchange of the cooling bath with a water bath. After stirring for 30 min at 23 °C, the red solution was cooled to –78 °C and a solution of aldehyde **S94** (1.07 g, 6.00 mmol, 1 equiv) in tetrahydrofuran (4.70 mL) was added. Stirring was continued for 30 min at –78 °C, after which the reaction mixture was warmed to 23 °C by exchange of the cooling bath with a water bath. After stirring for 15 h at 23 °C, the reaction mixture was diluted with a saturated aqueous solution of ammonium chloride (50 mL) and diethyl ether (40 mL), which resulted in decolorization of the reaction mixture. The resulting suspension was filtered through a glass sinter filter and the remaining solid was washed with diethyl ether (30 mL) before being discharged. The organic layer was separated and the aqueous layer was extracted with diethyl ether (2 × 40 mL). The combined organic layers were washed successively with a saturated aqueous solution of sodium chloride (2 × 50 mL), an aqueous hydrogen peroxide solution (10 wt%, 2 × 40 mL), and a saturated aqueous solution of sodium chloride (2 × 50 mL). The washed organic layer was dried over sodium sulfate, the dried organic layer was filtered, and the filtrate was concentrated under reduced pressure. The residue was purified by flash column chromatography on silica gel (15% dichloromethane in *n*-pentane) to yield alkene **S15** (1.07 g, 5.26 mmol, 88%) as a colorless oil. The obtained analytical data for alkene **S15** were in accordance with reported literature values.[23]

2.4.21 1,1-Disubstituted alkene **S16**

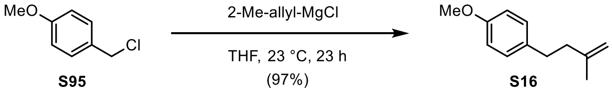

Preparation of 1,1-disubstituted alkene **S16**: To a solution of 4-methoxybenzyl chloride (**S95**) (1.04 g, 6.62 mmol, 1 equiv) in tetrahydrofuran (3.30 mL) was added dropwise a solution of 2-

methylallylmagnesium chloride (0.500 M in tetrahydrofuran, 26.5 mL, 13.2 mmol, 2.00 equiv) at 23 °C. After stirring for 23 h at 23 °C, a saturated aqueous solution of ammonium chloride (60 mL) was added cautiously to the reaction mixture. The organic layer was separated and the aqueous layer was extracted with dichloromethane (3 × 60 mL). The combined organic layers were dried over sodium sulfate, the dried solution was filtered, and the filtrate was concentrated under reduced pressure. The residue was purified by flash column chromatography on silica gel (15% dichloromethane in *n*-pentane) to yield 1,1-disubstituted alkene **S16** (1.13 g, 6.43 mmol, 97%) as a colorless oil. The obtained analytical data for 1,1-disubstituted alkene **S16** were in accordance with reported literature values.[25]

2.4.22 Tetrasubstituted alkene **S18**

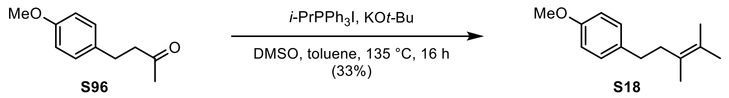

Preparation of tetrasubstituted alkene **S18**: To a suspension of potassium *tert*-butoxide (2.52 g, 22.4 mmol, 4.00 equiv) in dimethyl sulfoxide (20.0 mL) was added isopropyltriphenylphosphonium iodide (9.70 g, 22.4 mmol, 4.00 equiv) at 23 °C. After stirring for 10 min at 23 °C, a solution of ketone **S96** (1.00 g, 5.61 mmol, 1 equiv) in toluene (18.0 mL) was added and the resulting solution was heated to 135 °C for 16 h. After cooling to 23 °C, the reaction mixture was diluted by addition of hexanes (100 mL) and water (100 mL). The organic phase was separated and washed in succession with water (2 × 100 mL) and a saturated aqueous solution of sodium chloride (100 mL). The washed organic layer was dried over sodium sulfate, the dried solution was filtered, and the filtrate was concentrated under reduced pressure. The residue was purified by flash column chromatography on silica gel (2% diethyl ether in *n*-pentane) followed by semipreparative normal-phase high performance liquid chromatography (HPLC) (0.1% to 3.0% ethyl acetate in *n*-hexane) to afford tetrasubstituted alkene **S18** (376 mg, 1.84 mmol, 33%) as a colorless oil. The obtained analytical data for tetrasubstituted alkene **S18** were in accordance with reported literature values.[26]

2.4.23 Phenyl prenyl ether **S19**

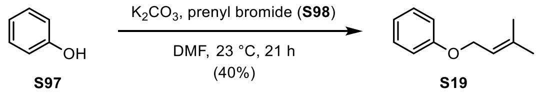

Phenyl prenyl ether **S19** was prepared according to a modified literature procedure[27,28]: To a solution of phenol (**S97**) (600 mg, 6.38 mmol, 1 equiv) in dimethylformamide (97.0 mL) was

added potassium carbonate (2.64 g, 19.1 mmol, 3.00 equiv) at 23 °C, after which the reaction mixture was heated to 60 °C for 1 h. After cooling of the reaction mixture to 23 °C, prenyl bromide (**S98**) (1.43 g, 9.56 mmol, 1.50 equiv) was added and stirring was continued at 23 °C for 21 h. Next, the reaction mixture was diluted with dichloromethane (50 mL) and water (100 mL). The organic layer was separated and the aqueous layer was extracted with dichloromethane (4 × 50 mL). The combined organic layers were washed in succession with water (50 mL) and a saturated aqueous solution of sodium chloride (50 mL). The washed organic layer was dried over sodium sulfate, the dried organic layer was filtered, and the filtrate was concentrated under reduced pressure. The residue was purified by flash column chromatography on silica gel (7% dichloromethane in *n*-pentane) to yield phenyl prenyl ether **S19** (412 mg, 2.54 mmol, 40%) as a colorless oil. The obtained analytical data for phenyl prenyl ether **S19** were in accordance with reported literature values.[27]

2.4.24 Alkene **S20**

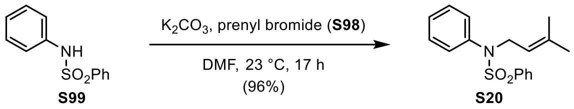

Preparation of alkene **S20**: To a suspension of sulfonamide **S99** (700 mg, 3.00 mmol, 1 equiv) and potassium carbonate (829 mg, 6.00 mmol, 2.00 equiv) in dimethylformamide (9.70 mL) was added prenyl bromide (**S98**) (671 mg, 4.50 mmol, 1.50 equiv) at 23 °C. After stirring for 17 h at 23 °C, triethylamine (455 mg, 4.50 mmol, 1.50 equiv) was added to the reaction mixture and stirring was continued for 10 min. To the reaction mixture were added diethyl ether (15 mL) and water (15 mL). The organic layer was separated and the aqueous layer was extracted with diethyl ether (3 × 15 mL). The combined organic layers were washed in succession with water (2 × 100 mL) and a saturated aqueous solution of sodium chloride (2 × 100 mL). The washed organic layer was dried over sodium sulfate, the dried organic layer was filtered, and the filtrate was concentrated under reduced pressure. The residue was purified by flash column chromatography on silica gel (10% diethyl ether in *n*-pentane) to yield alkene **S20** (864 mg, 2.87 mmol, 96%) as a yellowish oil.

<u>Analytical data of alkene **S20**:</u>

TLC (20% diethyl ether in pentane): R_f = 0.50 (UV, CAM).

^{1}H NMR (400 MHz, $CDCl_3$): δ 7.65 – 7.60 (m, 2H), 7.56 (tt, *J* = 7.3, 1.2 Hz, 1H), 7.49 – 7.42 (m, 2H), 7.31 – 7.25 (m, 3H), 7.06 – 7.00 (m, 2H), 5.08 (thept, *J* = 7.1, 1.4 Hz, 1H), 4.17 (d, *J* = 7.0 Hz, 2H), 1.58 (s, 3H), 1.47 (s, 3H).

^{13}C NMR (101 MHz, $CDCl_3$): δ 139.4, 138.9, 137.2, 132.6, 129.0 (2C), 128.9 (2C), 128.9 (2C), 127.8, 127.8 (2C), 118.8, 48.9, 25.8, 17.9.

IR (ATR, neat): $\tilde{\nu}$ = 3064 (w), 2970 (w), 2916 (w), 1673 (w), 1595 (w), 1492 (m), 1447 (m), 1377 (w), 1348 (s), 1310 (w), 1292 (w), 1223 (w), 1196 (w), 1162 (s), 1092 (m), 1060 (w), 1025 (w), 999 (w), 917 (w), 878 (m), 841 (m), 760 (m), 732 (s) cm^{-1}.

HRMS (ESI): calcd for $C_{17}H_{19}NO_2SNa^+$ $[M+Na]^+$: 324.1029; found: 324.1021.

2.4.25 Alkene **S21**

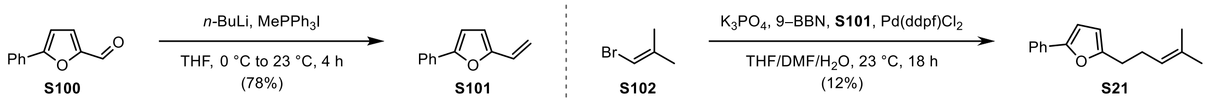

2-Phenyl-5-vinylfuran **S101** was prepared according to a modified literature procedure[29,30]: To a solution of methyltriphenylphosphonium iodide (2.82 g, 6.97 mmol, 1.20 equiv) in tetrahydrofuran (20.0 mL) was added dropwise a solution of *n*-butyllithium (3.99 mL, 1.60 M in hexanes,, 6.39 mmol, 1.10 equiv) 0 °C. After stirring for 30 min at 0 °C, a solution of 5-phenylfuran-2-carbaldehyde (**S100**) (1.00 g, 5.81 mmol, 1 equiv) in tetrahydrofuran (6.00 mL) was added. The resulting solution was kept a 0 °C for 20 min before being warmed to 23 °C. After stirring for 4 h, a saturated aqueous solution of ammonium chloride (50 mL) was added. The organic layer was separated and the aqueous layer was extracted with diethyl ether (3 × 50 mL). The combined organic layers were dried over sodium sulfate, the dried organic layer was filtered, and the filtrate was concentrated under reduced pressure. The residue was purified by flash column chromatography on silica gel (1% dichloromethane in *n*-pentane) to afford 2-phenyl-5-vinylfuran (**S101**) (770 mg, 4.52 mmol, 78%) as a colorless oil.

Analytical data of 2-phenyl-5-vinylfuran (**S101**):

TLC (*n*-pentane): R_f = 0.50 (UV, CAM).

^{1}H NMR (400 MHz, $CDCl_3$): δ 7.74 – 7.67 (m, 2H), 7.43 – 7.34 (m, 2H), 7.30 – 7.23 (m, 1H), 6.65 (d, J = 3.4 Hz, 1H), 6.54 (dd, J = 17.5, 11.3 Hz, 1H), 6.35 (d, J = 3.4 Hz, 1H), 5.78 (d, J = 17.5 Hz, 1H), 5.20 (dd, J = 11.2, 1.4 Hz, 1H).

^{13}C NMR (101 MHz, $CDCl_3$): δ 153.4, 152.8, 130.8, 128.8 (2C), 127.6, 125.1, 124.0 (2C), 112.3, 110.5, 106.9.

IR (ATR, neat): $\tilde{\nu}$ = 3011 (w), 1522 (w), 1483 (m), 1449 (m), 1008 (s), 978 (m), 897 (m), 787 (m), 756 (s), 688 (m) cm^{-1}.

HRMS (ESI): calcd for $C_{12}H_{11}O^+$ $[M+H]^+$: 171.0804; found: 171.0800.

Preparation of alkene **S21**: To a solution of 2-phenyl-5-vinylfuran (**S101**) (760 mg, 4.47 mmol, 2.00 equiv) in tetrahydrofuran (15.0 mL) was added 9-borabicyclo[3.3.1]nonane (11.0 mL, 0.500 M in tetrahydrofuran, 5.58 mmol, 2.50 equiv) at 23 °C. After stirring for 3 h at 23 °C, a mixture of *N*,*N*-dimethylformamide/water (9:1, 22.0 mL), potassium phosphate (1.18 g, 5.58 mmol, 2.50 equiv), [1,1′-bis(diphenylphosphino)ferrocene]dichloropalladium(II) (161 mg, 223 µmol, 0.100 equiv), and vinyl bromide **S102** (301 mg, 2.23 mmol, 1 equiv) were added in sequence. The resulting suspension was stirred for 12 h at 23 °C before being diluted by addition of a saturated aqueous solution of ammonium chloride (20 mL) and diethyl ether (20 mL). The organic layer was separated and the aqueous layer was extracted with diethyl ether (3 × 20 mL). The combined organic layers were washed with a saturated aqueous solution of sodium chloride (3 × 50 mL). The washed organic layer was dried over sodium sulfate, the dried organic layer was filtered, and the filtrate was concentrated under reduced pressure. The residue was purified by flash column chromatography on silica gel (0.5% dichloromethane in *n*-pentane) to afford alkene **S21** (62.7 mg, 277 µmol, 12%) as a colorless oil.

<u>Analytical data of alkene **S21**:</u>

TLC (*n*-pentane): R_f = 0.36 (UV, CAM).

^{1}H NMR (400 MHz, $CDCl_3$): δ 7.67 – 7.60 (m, 2H), 7.36 (t, *J* = 7.8 Hz, 2H), 7.24 – 7.18 (m, 1H), 6.55 (d, *J* = 3.2 Hz, 1H), 6.07 (dt, *J* = 3.2, 1.0 Hz, 1H), 5.20 (thept, *J* = 7.0, 1.4 Hz, 1H), 2.71 (t, *J* = 7.6 Hz, 2H), 2.39 (q, *J* = 7.5 Hz, 3H), 1.71 (s, 3H), 1.62 (s, 3H).

^{13}C NMR (101 MHz, $CDCl_3$): δ 156.1, 152.3, 132.7, 131.4, 128.7 (2C), 126.9, 123.5 (2C), 123.4, 107.2, 105.8, 28.6, 26.8, 25.8, 17.8.

IR (ATR, neat): $\tilde{\nu}$ = 1610 (w), 1594 (w), 1579 (w), 1547 (m), 1487 (w), 1447 (w), 1377 (w), 1329 (w), 1286 (w), 1205 (w), 1104 (w), 1064 (w), 1022 (m), 965 (w), 921 (w), 908 (w), 833 (w), 783 (m), 756 (s), 690 (m), 661 (w), 497 (w), 456 (w) cm^{-1}.

HRMS (ESI): calcd for $C_{16}H_{19}O^+$ $[M+H]^+$: 227.1430; found: 227.1428.

2.4.26 Prenyl pyrrole **S22**

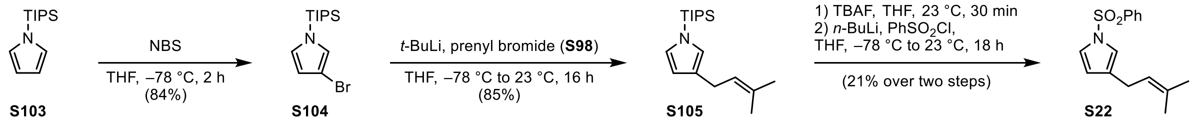

3-Bromo-*N*-TIPS pyrrole **S104** was prepared according to a modified literature procedure[31]: To a –78 °C solution of *N*-TIPS-pyrrole (**S103**) (2.50 g, 11.2 mmol, 1 equiv) in tetrahydrofuran (50.0 mL) was slowly added a solution of *N*-bromosuccinimide (2.09 g, 11.7 mmol, 1.05 equiv) in tetrahydrofuran (20.0 mL) over 30 min using a syringe pump. After stirring for 2 h at –78 °C, the reaction mixture was diluted by addition of a saturated aqueous sodium bicarbonate solution (100 mL). The organic layer was separated and the aqueous layer was extracted with diethyl ether (3 × 50 mL). The combined organic layers were dried over sodium sulfate, the dried organic layer was filtered, and the filtrate was concentrated under reduced pressure. The residue was purified by flash column chromatography on silica gel (*n*-pentane) to afford 3-bromo-*N*-TIPS pyrrole (**S104**) (2.83 g, 9.36 mmol, 84%) as a colorless oil. The obtained analytical data for 3-Bromo-*N*-TIPS pyrrole (**S104**) were in accordance with reported literature values.[31]

3-Prenyl-*N*-TIPS pyrrole **S105** was prepared according to a modified literature procedure[29]: To a solution of 3-bromo-*N*-TIPS pyrrole (**S104**) (2.83 g, 9.36 mmol, 1 equiv) in tetrahydrofuran (50.0 mL) was added dropwise a solution of *t*-butyllithium (11.0 mL, 1.70 M in *n*-pentane, 18.7 mmol, 2.00 equiv) at –78 °C. After complete addition, the resulting solution was stirred for 5 min at –78 °C. Then, a solution of prenyl bromide (1.31 mL, 11.2 mmol, 1.20 equiv) in tetrahydrofuran (6.00 mL) was added dropwise at –78 °C and the resulting solution was allowed to slowly warm up in the cooling bath to 23 °C over 16 h. Triethylamine (1.00 mL, 7.17 mmol, 0.766 equiv) was added and stirring was continued for 10 min, after which a saturated aqueous sodium chloride solution (20 mL) was added. The organic layer was separated and the aqueous layer was extracted with diethyl ether (3 × 30 mL). The combined organic layers were dried over sodium sulfate, the dried organic layer was filtered, and the filtrate was concentrated under reduced pressure. The residue was purified by flash column chromatography on silica gel (*n*-pentane) to afford 3-prenyl-*N*-TIPS pyrrole (**S105**) (2.31 g, 7.92 mmol, 85%) as a colorless oil.

Analytical data of 3-prenyl-*N*-TIPS pyrrole (**S105**):

TLC (*n*-pentane): R_f = 0.23 (UV, CAM).

^{1}H NMR (400 MHz, $CDCl_3$): δ 6.70 (t, *J* = 2.4 Hz, 1H), 6.51 (s, 1H), 6.17 – 6.13 (m, 1H), 5.37 (tp, *J* = 7.2, 1.5 Hz, 1H), 3.21 (d, *J* = 7.1 Hz, 2H), 1.74 (s, 3H), 1.70 (s, 3H), 1.43 (hept, *J* = 7.4 Hz, 3H), 1.10 (d, *J* = 7.6 Hz, 18H).

^{13}C NMR (101 MHz, $CDCl_3$): δ 131.1, 125.5, 124.6, 124.3, 121.2, 110.8, 26.0, 25.9, 18.0 (6C), 17.9, 11.9 (3C).

IR (ATR, neat): $\tilde{\nu}$ = 2946 (m), 2926 (m), 2893 (w), 2867 (m), 1535 (w), 1464 (m), 1384 (w), 1303 (w), 1259 (w), 1186 (w), 1091 (s), 1070 (m), 1016 (m), 995 (w), 963 (w), 921 (w), 883 (m), 847 (w), 769 (m), 689 (s), 656 (s), 631 (m), 610 (w), 577 (m), 524 (m), 489 (w), 456 (w), 435 (w) cm^{-1}.

HRMS (ESI): calcd for $C_{18}H_{34}NSi^+$ $[M+H]^+$: 292.2455; found: 292.2454.

Preparation of prenyl pyrrole **S22**: To a solution of 3-prenyl-*N*-TIPS pyrrole (**S105**) (300 mg, 1.03 mmol, 1 equiv) in tetrahydrofuran (10.0 mL) was added dropwise a solution of tetrabutylammonium fluoride (1.18 mL, 1.00 M in tetrahydrofuran, 1.18 mmol, 1.15 equiv) at 23 °C. After stirring for 30 min at 23 °C, the reaction mixture was concentrated under reduced pressure. The residue was purified by flash column chromatography on silica gel (10% ethyl acetate and 5% triethylamine in hexanes). The intermediate 3-prenyl pyrrole was immediately used for the following step. To a solution of intermediate 3-prenyl pyrrole (in theory: 1.03 mmol, 1 equiv) in tetrahydrofuran (10.0 mL) was added dropwise *n*-butyllithium (675 µL, 1.60 M in hexanes, 1.08 mmol, 1.05 equiv) at –78 °C. After stirring for 20 min at –78 °C, the reaction mixture was allowed to warm to 23 °C over 1 h. The solution was cooled to –78 °C and a solution of benzenesulfonyl chloride (146 µL, 1.13 mmol, 1.10 equiv) in tetrahydrofuran (1.00 mL) was added dropwise –78 °C. The resulting mixture was allowed to slowly warm up in the cooling bath to 23 °C over 16 h, after which water (2 mL) and a saturated aqueous sodium chloride solution (20 mL) were added. The organic layer was separated and the aqueous layer was extracted with diethyl ether (3 × 50 mL). The combined organic layers were washed in succession with water (10 mL) and a half-saturated aqueous solution of sodium chloride (10.0 mL). The washed organic layer was dried over sodium sulfate, the dried organic layer was filtered, and the filtrate was concentrated under reduced pressure. The residue was purified by flash column chromatography on silica gel (5% diethyl ether in *n*-pentane) to afford the prenyl pyrrole **S22** (60.0 mg, 220 µmol, 21% over two steps) as a slightly yellow oil.

<u>Analytical data of prenyl pyrrole **S22**:</u>

TLC (5% diethyl ether in *n*-pentane): R_f = 0.42 (UV, CAM).

^{1}H NMR (400 MHz, $CDCl_3$): δ 7.86 – 7.81 (m, 2H), 7.61 – 7.56 (m, 1H), 7.52 – 7.46 (m, 2H), 7.08 (dd, *J* = 3.2, 2.3 Hz, 1H), 6.90 – 6.87 (m, 1H), 6.15 (dd, *J* = 3.2, 1.6 Hz, 1H), 5.27 – 5.19 (m, 1H), 3.09 (d, *J* = 7.2 Hz, 2H), 1.72 (q, *J* = 1.3 Hz, 3H), 1.63 (s, 3H).

^{13}C NMR (101 MHz, $CDCl_3$): δ 139.4, 133.7, 133.1, 129.4 (2C), 129.4 126.8 (2C), 122.0, 121.2, 117.4, 115.0, 25.8, 25.7, 17.8.

IR (ATR, neat): $\tilde{\nu}$ = 3139 (w), 2969 (w), 2914 (w), 1584 (w), 1473 (w), 1448 (m), 1368 (s), 1311 (w), 1283 (w), 1251 (m), 1174 (s), 1095 (s), 1058 (s), 1024 (w), 999 (w), 956 (w), 846 (w), 782 (w), 755 (m), 728 (s), 685 (m), 610 (s), 588 (s), 558 (m) cm^{-1}.

HRMS (ESI): calcd for $C_{15}H_{18}NO_2S^+$ [M+H]$^+$: 276.1053; found: 276.1049.

2.4.27 2-Prenyl indole **S23**

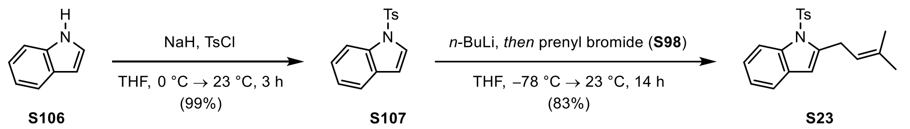

N-Tosylindole **S107** was prepared according to a modified literature procedure[32]: To a solution of indole (**S106**) (2.01 g, 17.1 mmol, 1 equiv) in tetrahydrofuran (60.3 mL) was added sodium hydride (1.03 g, 60.0% wt, 25.7 mmol, 1.50 equiv) at 0 °C, which resulted in the formation of a grayish suspension. After stirring for 30 min at 0 °C, a solution of *p*-toluenesulfonyl chloride (3.58 g, 18.8 mmol, 1.10 equiv) in tetrahydrofuran (25.0 mL) was added. After stirring for 5 min at 0 °C, the reaction mixture was warmed to 23 °C through exchange of the cooling bath with a water bath. After stirring for 3 h at 23 °C, the reaction mixture was diluted with diethyl ether (300 mL) and water (100 mL). The aqueous layer was separated and the organic layer was washed in succession with a 2 M aqueous solution of sodium hydroxide (2 × 50 mL) and a saturated aqueous solution of sodium chloride (50 mL). The washed organic layer was dried over sodium sulfate, the dried organic layer was filtered, and the filtrate was concentrated under reduced pressure. Purification by flash column chromatography on silica gel (5% diethyl ether in *n*-pentane) afforded *N*-tosylindole (**S107**) (4.61 g, 17.0 mmol, 99%) as a white solid. The obtained analytical data for *N*-tosylindole (**S107**) were in accordance with reported literature values.[32]

2-Prenyl indole **S23** was prepared according to a modified literature procedure[33]: To a solution of 1-(*p*-toluenesulfonyl)indole (**S107**) (985 mg, 3.63 mmol, 1 equiv) in tetrahydrofuran (9.00 mL) was added a solution of *n*-butyllithium (1.60 M in hexanes, 2.50 mL, 3.99 mmol, 1.10 equiv) at –78 °C, upon which the solution turned from colorless to orange-yellow. After stirring for 30 min at –78 °C, prenyl bromide (**S98**) (649 mg, 4.35 mmol, 1.20 equiv) was

added at –78 °C and the reaction mixture was allowed to slowly warm up in the cooling bath to 23 °C. After stirring for 13 h 30 min, triethylamine (759 µL, 5.44 mmol, 1.50 equiv) was added to remove an excess of prenyl bromide and stirring was continued for 15 min at 23 °C. Next, a saturated aqueous solution of ammonium chloride (20 mL) was added and the biphasic mixture was extracted with dichloromethane (4 × 20 mL). The combined organic layers were dried over sodium sulfate, the dried organic layer was filtered, and the filtrate was concentrated under reduced pressure. The residue was purified by flash column chromatography on silica gel (4% diethyl ether in *n*-pentane) to yield 2-prenyl indole **S23** (1.02 g, 3.01 mmol, 83%) as a white solid. The obtained analytical data for 2-prenyl indole **S23** were in accordance with reported literature values.[33]

2.4.28 3-Prenyl indole **S24**

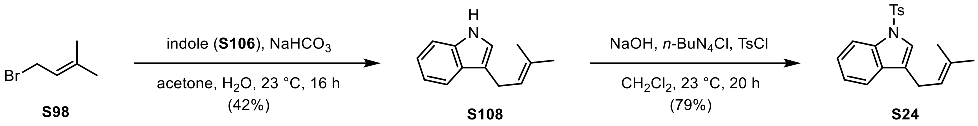

Indole **S108** was prepared according to a modified literature procedure[34]: To a solution of indole (**S106**) (7.17 g, 61.2 mmol, 5.00 equiv) in acetone (19.6 mL) and water (4.90 mL) was added in succession sodium hydrogencarbonate (2.06 g, 24.5 mmol, 2.00 equiv) and prenyl bromide (**S98**) (1.82 g, 12.2 mmol, 1 equiv) at 23 °C. After stirring for 16 h at 23 °C, diethyl ether (40 mL) and water (40 mL) were added. The organic layer was separated and the aqueous layer was extracted with diethyl ether (3 × 40 mL). The combined organic layers were dried over sodium sulfate, the dried organic layer was filtered, and the filtrate was concentrated under reduced pressure. The residue was purified by flash column chromatography on silica gel (3.5% diethyl ether in *n*-pentane) to yield indole **S108** (960 mg, 5.18 mmol, 42%) as a yellowish oil. The obtained analytical data for indole **S108** were in accordance with reported literature values.[34]

3-Prenyl indole **S24** was prepared according to a modified literature procedure[33]: To a solution of indole **S108** (960 mg, 10.4 mmol, 1 equiv) in dichloromethane (13.0 mL) was added in succession tetra-*n*-butylammonium chloride (144 mg, 518 µmol, 10.0 mol%) and finely powdered sodium hydroxide (461 mg, 90.0 wt%, 10.4 mmol, 2.00 equiv) at 23 °C, after which the slightly yellowish solution turned green. After stirring for 15 min at 23 °C, a solution of *p*-toluenesulfonyl chloride (1.19 g, 6.22 mmol, 1.20 equiv) in dichloromethane (8.88 mL) was added dropwise resulting in a color change to orange. Stirring was continued for 20 h at 23 °C, after which the reaction mixture was diluted with diethyl ether (100 mL) and washed in succession with a 2 M aqueous solution of sodium hydroxide (3 × 30 mL) and a saturated

aqueous solution of sodium chloride (30 mL).The washed organic layer was dried over sodium sulfate, the dried organic layer was filtered, and the filtrate was concentrated under reduced pressure. The residue was purified by flash column chromatography on silica gel (5% diethyl ether in *n*-pentane) to yield 3-prenyl indole **S24** (1.38 g, 4.07 mmol, 79%) as a colorless oil. The obtained analytical data for 3-prenyl indole **S24** were in accordance with reported literature values.[33]

2.4.29 Alkene **S25**

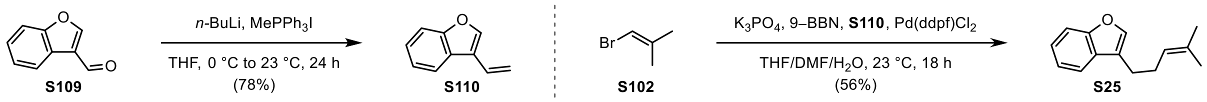

3-Vinyl-benzo[b]furane **S110** was prepared according to a modified literature procedure[30]: To a solution of methyltriphenylphosphonium iodide (3.32 g, 8.21 mmol, 1.20 equiv) in tetrahydrofuran (22.0 mL) was added dropwise a solution of *n*-butyllithium (4.70 mL, 1.60 M in hexanes, 7.52 mmol, 1.10 equiv) at 0 °C. After stirring for 30 min at 0 °C, a solution of benzo[b]furan-3-carbaldehyde (**S109**) (1.00 g, 6.84 mmol, 1 equiv) in tetrahydrofuran (7.00 mL) was added and the resulting solution was kept at 0 °C for 20 min before being allowed to warm to 23 °C. After stirring for 24 h, a saturated aqueous solution of ammonium chloride (50 mL) was added. The organic layer was separated and the aqueous layer was extracted with diethyl ether (3 × 50 mL). The combined organic layers were dried over sodium sulfate, the dried organic layer was filtered, and the filtrate was concentrated under reduced pressure. The residue was purified by flash column chromatography on silica gel (1% dichloromethane in *n*-pentane) to afford 3-vinyl-benzo[b]furane (**S110**) (771 mg, 5.35 mmol, 78%) as a colorless oil. The obtained analytical data for 3-vinyl-benzo[b]furane (**S110**) were in accordance with reported literature values.[30]

Preparation of alkene **S25**: To a solution of 3-vinyl-benzo[b]furane (**S110**) (771 mg, 5.35 mmol, 2.00 equiv) in tetrahydrofuran (15.0 mL) was added 9-borabicyclo[3.3.1]nonane (13.4 mL, 0.500 M in tetrahydrofuran, 6.68 mmol, 2.50 equiv) at 23 °C. After stirring for 3 h at 23 °C, the reaction mixture was added to a suspension of potassium phosphate (1.42 mg, 6.68 mmol, 2.50 equiv), [1,1′-bis(diphenylphosphino)ferrocene]dichloropalladium(II) (196 mg, 267 µmol, 0.100 equiv), and vinyl bromide **S102** (361 mg, 2.67 mmol, 1 equiv) in a mixture of *N*,*N*-dimethylformamide/water (9:1, 22.0 mL) at 23 °C. The resulting deep-red suspension was stirred for 18 h at 23 °C, after which a saturated aqueous solution of ammonium chloride (20 mL) and diethyl ether (20 mL) were added. The organic layer was separated and the aqueous layer was extracted with diethyl ether (3 × 20 mL). The combined organic layers were

washed with a saturated aqueous solution of sodium chloride (3 × 50 mL). The washed organic layer was dried over sodium sulfate, the dried organic layer was filtered, and the filtrate was concentrated under reduced pressure. The residue was purified by flash column chromatography on silica gel (*n*-pentane) to afford alkene **S25** (298 mg, 1.49 mmol, 56%) as a colorless oil.

<u>Analytical data of alkene **S25**:</u>

TLC (*n*-pentane): R_f = 0.37 (UV, CAM).

^{1}H NMR (400 MHz, $CDCl_3$): δ 7.56 (d, *J* = 7.6 Hz, 1H), 7.46 (d, *J* = 7.2 Hz, 1H), 7.41 (s, 1H), 7.31 – 7.26 (m, 1H), 7.23 (td, *J* = 7.4, 1.2 Hz, 1H), 5.22 (thept, *J* = 7.2, 1.4 Hz, 1H), 2.70 (t, *J* = 7.6 Hz, 2H), 2.39 (q, *J* = 7.4 Hz, 2H), 1.70 (s, 3H), 1.60 (s, 3H).

^{13}C NMR (101 MHz, $CDCl_3$): δ 155.5, 141.2, 132.7, 128.5, 124.1, 123.9, 122.3, 120.5, 119.8, 111.5, 27.8, 25.8, 24.0, 17.9.

IR (ATR, neat): $\tilde{\nu}$ = 2967 (w), 2915 (w), 2855 (w), 1583 (w), 1452 (s), 1377 (w), 1280 (w), 1184 (m), 1091 (m), 1009 (w), 929 (w), 857 (w), 743 (s), 588 (w), 422 (w) cm^{-1}.

HRMS (ESI): calcd for $C_{14}H_{17}O^+$ $[M+H]^+$: 201.1274; found: 201.1273.

2.4.30 Alkene **S26**

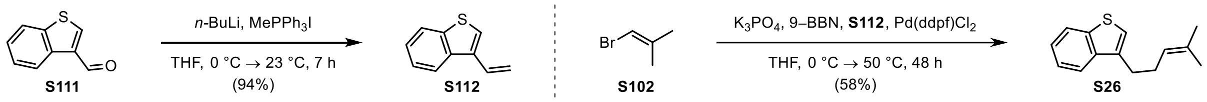

Alkene **S112** was prepared according to a modified literature procedure[35]: To a solution of methyltriphenylphosphonium iodide (3.01 g, 7.45 mmol, 1.20 equiv) in tetrahydrofuran (24.8 mL) was added a solution of *n*-butyllithium (1.60 M in hexanes, 4.27 mL, 6.83 mmol, 1.10 equiv) at 0 °C, upon which the colorless solution turned to a yellow suspension. After stirring for 30 min at 0 °C, a solution of aldehyde **S111** (1.01 g, 6.21 mmol, 1 equiv) in tetrahydrofuran (6.20 mL) was added at 0 °C. Stirring was continued for 30 min at 0 °C, after which the reaction mixture was warmed to 23 °C through exchange of the cooling bath with a water bath. After stirring for 6 h at 23 °C, a saturated aqueous solution of ammonium chloride (50 mL), diethyl ether (50 mL) and *n*-pentane (50 mL) were added. The aqueous layer was separated and the organic layer was washed with a saturated aqueous solution of sodium chloride (50 mL). The washed organic layer was dried over sodium sulfate, the dried organic layer was filtered, and the filtrate was concentrated under reduced pressure. The residue was purified by flash column chromatography on silica gel (100% *n*-pentane) to yield alkene **S112**

(933 mg, 5.82 mmol, 94%) as a colorless oil. The obtained analytical data for alkene **S112** were in accordance with reported literature values.[35]

Preparation of alkene **S26**: To a solution of alkene **S112** (893 mg, 5.57 mmol, 2.00 equiv) in degassed tetrahydrofuran (7.00 mL) was added a solution of 9-borabicyclo[3.3.1]nonane, (0.500 M in tetrahydrofuran, 13.9 mL, 6.96 mmol, 2.50 equiv) at 0 °C. The reaction mixture was warmed to 23 °C through exchange of the cooling bath with a water bath. After stirring for 3 h at 23 °C, to the reaction mixture were added in succession degassed dimethylformamide (28.0 mL), a degassed aqueous solution of potassium phosphate (3.00 M, 2.32 mL, 6.96 mmol, 2.50equiv), 1,1'-bis(diphenylphosphino)ferrocene-palladium(II) dichloride (204 mg, 279 µmol, 0.100 equiv), and vinyl bromide **S102** (376 mg, 2.79 mmol, 1 equiv) at 23 °C. The reaction mixture was stirred for 45 h at 50 °C, after which the reaction mixture was allowed to cool to 23 °C and *n*-pentane (30 mL) and a saturated aqueous solution of ammonium chloride (30 mL) were added. The organic layer was separated and the aqueous layer was extracted with *n*-pentane (3 × 30 mL). The combined organic layers were dried over magnesium sulfate, the dried organic layer was filtered, and the filtrate was concentrated under reduced pressure. The residue was purified by flash column chromatography on silica gel (100% *n*-pentane) to yield alkene **S26** (348 mg, 1.61 mmol, 58%) as a colorless oil.

<u>Analytical data of alkene **S26**:</u>

TLC (100% pentane): R_f = 0.54 (UV, CAM).

^{1}H NMR (400 MHz, $CDCl_3$): δ 7.90 – 7.84 (m, 1H), 7.77 (dd, *J* = 7.3, 1.4 Hz, 1H), 7.40 (td, *J* = 7.5, 1.5 Hz, 1H), 7.35 (td, *J* = 7.4, 1.5 Hz, 1H), 7.10 (s, 1H), 5.26 (thept, *J* = 7.1, 1.4 Hz, 1H), 2.88 (td, *J* = 7.7, 1.1 Hz, 2H), 2.46 (q, *J* = 7.5 Hz, 2H), 1.73 (q, *J* = 1.2 Hz, 3H), 1.61 (s, 3H).

^{13}C NMR (101 MHz, $CDCl_3$): δ 140.6, 139.3, 136.9, 132.6, 124.2, 123.9, 123.9, 123.0, 121.8, 121.1, 28.9, 27.8, 25.9, 17.9.

IR (ATR, neat): $\tilde{\nu}$ = 3060 (w), 2965 (w), 2913 (m), 2853 (w), 1427 (m), 1375 (w), 1316 (w), 1258 (w), 1240 (w), 1157 (w), 1106 (w), 1082 (w), 1051 (m), 1020 (w), 984 (m), 849 (s), 816 (s) cm^{-1}.

HRMS (ESI): calcd for $C_{14}H_{17}S^+$ [M+H]$^+$: 217.1045; found: 217.1045.

2.4.31 3-Geranyl indole **S27**

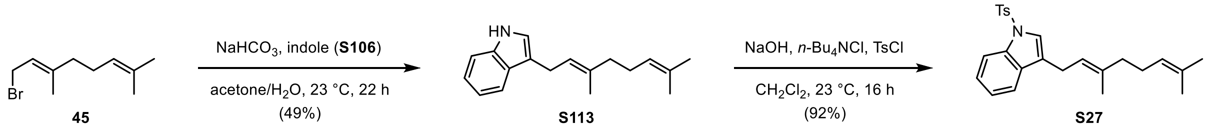

Indole **S113** was prepared according to a modified literature procedure[34]: To a solution of indole (**S106**) (5.49 g, 46.9 mmol, 5.00 equiv) in acetone (15.0 mL) and water (3.80 mL) was added in succession sodium bicarbonate (1.58 g, 18.7 mmol, 2.00 equiv) and geranyl bromide (**45**) (2.04 g, 9.37 mmol, 1 equiv) at 23 °C. After stirring for 22 h at 23 °C diethyl ether (40 mL) and water (40 mL) were added to the reaction mixture. The organic layer was separated and the aqueous layer was extracted with diethyl ether (3 × 40 mL). The combined organic layers were dried over sodium sulfate, the dried solution was filtered, and the filtrate was concentrated under reduced pressure. The residue was purified by automated flash column chromatography on silica gel (0% to 3% diethyl ether in *n*-pentane) to afford indole **S113** (1.16 g, 4.59 mmol, 49%) as a slightly yellowish oil. The obtained analytical data for indole **S113** were in accordance with reported literature values.[34]

3-Geranyl indole **S27** was prepared according to a modified literature procedure[33]: To a solution of indole **S113** (968 mg, 3.82 mmol, 1 equiv) in dichloromethane (9.60 mL) was added in succession tetrabutylammonium chloride (106 mg, 382 µmol, 0.100 equiv) and sodium hydroxide (340 mg, 90.0 wt%, 7.64 mmol, 2.00 equiv) at 23 °C, whereupon the slightly yellowish reaction mixture turned green. After stirring for 15 min at 23 °C, the reaction mixture was cooled to 0 °C and tosyl chloride (874 mg, 4.59 mmol, 1.20 equiv) was added, which resulted in a color change to orange. After stirring for 5 min at 0 °C, the cooling bath was removed and stirring was continued for 15.5 h at 23 °C. The reaction mixture was diluted with diethyl ether (100 mL) and washed in succession with a 2 M aqueous solution of sodium hydroxide (6 × 30 mL) and a saturated aqueous solution of sodium chloride (3 × 30 mL). The washed organic layer was dried over anhydrous sodium sulfate, the dried solution was filtered, and the filtrate was concentrated under reduced pressure. The residue was purified by flash column chromatography on silica gel (5% diethyl ether in *n*-pentane) to afford 3-geranyl indole **S27** (1.43 g, 3.51 mmol, 92%) as a colorless oil. The obtained analytical data for 3-geranyl indole **S27** were in accordance with reported literature values.[33]

2.4.32 2-Geranyl indole **S28**

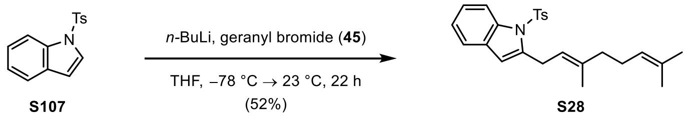

2-Geranyl indole **S28** was prepared according to a modified literature procedure[33]: To a solution of *N*-tosylindole (**S107**) (686 mg, 2.53 mmol, 1 equiv) in tetrahydrofuran (6.30 mL), was added a solution of *n*-butyllithium (1.60 M in hexanes, 1.74 mL, 2.78 mmol, 1.10 equiv) at –78 °C. After stirring for 30 min at –78 °C, geranyl bromide (**45**) (658 mg, 3.03 mmol,

1.20 equiv) was added to the yellow reaction mixture. The reaction mixture was allowed to slowly warm up to 23 °C in the cooling bath and stirring was continued for 22 h. Triethylamine (768 mg, 7.58 mmol, 3.00 equiv) and water (10 mL) were added to the reaction mixture. The organic layer was separated and the aqueous layer was extracted with diethyl ether (3 × 20 mL). The combined organic layers were dried over sodium sulfate, the dried organic layer was filtered, and the filtrate was concentrated under reduced pressure. Purification by flash column chromatography on silica gel (2% diethyl ether in *n*-pentane) afforded 2-geranyl indole **S28** (534 mg, 1.31 mmol, 52%) as a colorless oil. The obtained analytical data for 2-geranyl indole **S28** were in accordance with reported literature values.[33]

2.4.33 TIB-protected geraniol **S29**

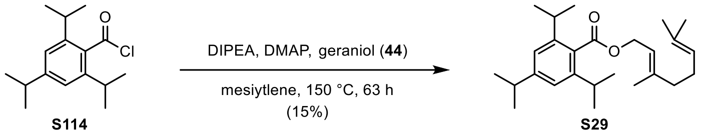

To a solution of 2,4,6-triisopropylbenzoyl chloride (**S114**) (116 mg, 435 µmol, 1 equiv) in mesitylene (750 µL) was added in succession 4-dimethylaminopyridine (9.9 mg, 81 µmol, 0.19 equiv), diisopropylethylamine (230 µL, 1.32 mmol, 3.04 equiv), and geraniol (**44**) (214 mg, 1.35 mmol, 3.10 equiv) at 23 °C. The reaction mixture was stirred for 63 h at 150 °C, after which the reaction mixture was allowed to cool to 60 °C and concentrated under reduced pressure (60 °C, down to 5 mbar). The residue was purified by flash column chromatography on silica gel (2% diethyl ether in *n*-pentane) to yield TIB-protected geraniol **S29** (25.2 mg, 65.5 µmol, 15%) as a slightly yellowish oil.

Analytical data of alkene **S29**:

TLC (2% diethyl ether in *n*-pentane): R_f = 0.28 (UV, CAM).

^{1}H NMR (400 MHz, $CDCl_3$): δ 7.00 (s, 2H), 5.49 (tq, *J* = 7.2, 1.3 Hz, 1H), 5.11 (thept, *J* = 6.8, 1.4 Hz, 1H), 4.83 (d, *J* = 7.3 Hz, 2H), 2.88 (hept, *J* = 6.9 Hz, 3H), 2.15 – 2.03 (m, 4H), 1.77 (d, *J* = 1.3 Hz, 3H), 1.69 (q, *J* = 1.1 Hz, 3H), 1.61 (s, 3H), 1.24 (d, *J* = 6.8 Hz, 12H), 1.24 (d, *J* = 6.9 Hz, 6H).

^{13}C NMR (101 MHz, $CDCl_3$): δ 171.0, 150.2, 144.9 (2C), 143.3, 132.0, 130.7, 123.9, 120.9 (2C), 118.1, 61.7, 39.7, 34.6, 31.5 (2C), 26.6, 25.8, 24.3 (4C), 24.1 (2C), 17.8, 16.7.

IR (ATR, neat): $\tilde{\nu}$ = 2960 (m), 2928 (m), 2870 (w), 1723 (s), 1670 (w), 1606 (w), 1575 (w), 1460 (m), 1383 (m), 1363 (w), 1281 (m), 1270 (m), 1247 (s), 1188 (w), 1136 (m), 1103 (m), 1067 (s), 922 (m), 876 (m), 835 (w), 769 (w), 655 (w) cm^{-1}.

HRMS (ESI): calcd for $C_{26}H_{40}O_2Na^+$ $[M+Na]^+$: 407.2921; found: 407.2903.

2.4.34 Alkene **S31**

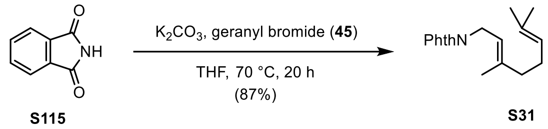

Alkene **S31** was prepared according to a modified literature procedure[36]: To a solution of phthalimide **S115** (500 mg, 3.40 mmol, 1 equiv) in tetrahydrofuran (14.2 mL) was added in succession potassium carbonate (1.41 g, 10.2 mmol, 3.00 equiv) and geranyl bromide (**45**) (1.11 g, 5.10 mmol, 1.50 equiv) at 23 °C. The reaction mixture was heated to 70 °C for 20 h, after which the reaction mixture was allowed to cool to 23 °C and triethylamine (344 mg, 3.40 mmol, 1.00 equiv), diethyl ether (15 mL) and water (15 mL) were added. The organic layer was separated and the aqueous layer was extracted with diethyl ether (3 × 15 mL). The combined organic layers were washed with a saturated aqueous solution of sodium chloride (50 mL), the washed organic layer was dried over sodium sulfate, the dried organic layer was filtered, and the filtrate was concentrated under reduced pressure. The residue was purified by flash column chromatography on silica gel (7% ethyl acetate in cyclohexane) to yield alkene **S31** (842 mg, 2.97 mmol, 87%) as a white solid. The obtained analytical data for alkene **S31** were in accordance with reported literature values.[36]

2.4.35 Geranyl phenyl sulfone (**S33**)

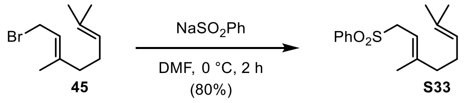

Geranyl phenyl sulfone **S33** was prepared according to a modified literature procedure[37]: To a solution of geranyl bromide (**45**) (1.00 g, 4.61 mmol, 1 equiv) in dimethylformamide (9.20 mL) was added sodium benzenesulfinate (1.13 g, 6.91 mmol, 1.50 equiv) at 0 °C. Stirring was continued for 2 h at 0 °C, after which the reaction mixture was diluted with ethyl acetate (70 mL) and washed in succession with water (40 mL) and a saturated aqueous solution of sodium chloride (40 mL). The washed organic layer was dried over sodium sulfate, the dried organic layer was filtered, and the filtrate was concentrated under reduced pressure. The residue was purified by flash column chromatography on silica gel (15% diethyl ether in *n*-pentane) to yield geranyl phenyl sulfone (**S33**) (1.03 g, 3.68 mmol, 80%) as a colorless oil.

The obtained analytical data for geranyl phenyl sulfone (**S33**) were in accordance with reported literature values.[37]

2.4.36 Alkene **S35**

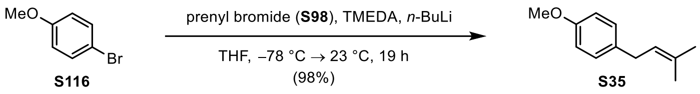

Alkene **S35** was prepared according to a modified literature procedure[38]: To a solution of bromide **S116** (1.10 g, 5.88 mmol, 1 equiv) in tetrahydrofuran (7.80 mL) was added *n*-butyllithium (1.60 M in hexanes, 5.15 mL, 8.23 mmol, 1.40 equiv) over a period of 10 min at –78 °C. After stirring for 10 min at –78 °C *N*,*N*,*N′*,*N′*-tetramethylethylenediamine (1.03 g, 8.82 mmol, 1.50 equiv) was added at –78 °C. After stirring for 30 min at –78 °C, prenyl bromide (**S98**) (1.31 g, 8.82 mmol, 1.50 equiv) was added at –78 °C, after which the reaction mixture was allowed to slowly warm up in the cooling bath to 23 °C. After stirring for 18 , water (15 ml) was added, the organic layer was separated, and the aqueous layer was extracted with diethyl ether (3 × 20 mL). The combined organic layers washed with a saturated aqueous solution of sodium chloride (50 mL), the washed organic layer was dried over sodium sulfate, the dried organic layer was filtered, and the filtrate was concentrated under reduced pressure. The residue was purified by flash column chromatography on silica gel (3% to 50% dichloromethane in *n*-pentane) to yield alkene **S35** (1.02 g, 5.78 mmol, 98%) as a colorless oil. The obtained analytical data for alkene **S35** were in accordance with reported literature values.[38]

2.4.37 Alkene **S36**

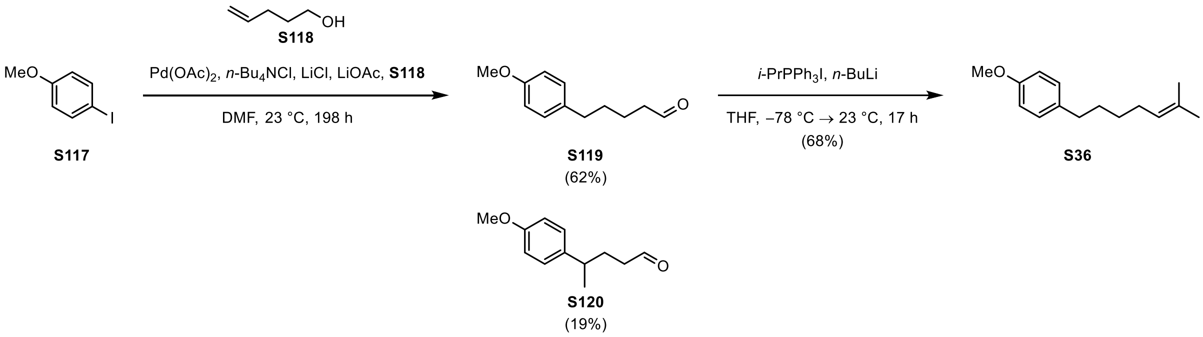

Aldehyde **S119** was prepared according to a modified literature procedure[39]: To a suspension of tetrabutylammonium chloride (3.56 g, 12.8 mmol, 2.00 equiv), palladium diacetate (43.2 mg, 192 µmol, 3.00 mol%), lithium chloride (272 mg, 6.41 mmol, 1.00 equiv), lithium acetate (1.06 g, 16.0 mmol, 2.50 equiv), and 1-iodo-4-methoxybenzene (**S117**) (1.50 g,

6.41 mmol, 1 equiv) in degassed dimethylformamide (12.8 mL) was added pent-4-en-1-ol (**S118**) (574 mg, 6.67 mmol, 1.04 equiv) at 23 °C under argon atmosphere. After stirring for 8 d at 23 °C under light protection, the reaction mixture was diluted with diethyl ether (60 mL) and washed with a saturated aqueous solution of sodium chloride (3 × 20 mL). The washed organic layer was dried over sodium sulfate, the dried organic layer was filtered, and the filtrate was concentrated under reduced pressure. Purification by flash column chromatography on silica gel (7% ethyl acetate in cyclohexane) followed by preparative normal-phase high performance liquid chromatography (HPLC) (5.0% to 15.0% ethyl acetate in *n*-hexane over 60 min) afforded aldehyde **S119** (767 mg, 3.99 mmol, 62%) as a colorless oil and aldehyde **S120** (238 mg, 1.24 mmol, 19%) as a colorless oil. The obtained analytical data for aldehyde **S119** were in accordance with reported literature values.[40] The obtained analytical data for aldehyde **S120** were in accordance with reported literature values.[41]

Preparation of alkene **S36**: To a suspension of isopropyltriphenylphosphonium iodide (1.41 g, 3.26 mmol, 1.45 equiv) in tetrahydrofuran (10.4 mL) was added a solution of *n*-butyllithium (1.60 M in hexanes, 1.90 mL, 3.04 mmol, 1.35 equiv) at –78 °C, upon which the yellow suspension turned orange. After stirring for 30 min at –78 °C, the reaction mixture was warmed to 23 °C through exchange of the cooling bath with a water bath. After stirring for 30 min at 23 °C, the wine-red reaction mixture was cooled to –78 °C and a solution of aldehyde **S119** (432 mg, 2.25 mmol, 1 equiv) in tetrahydrofuran (2.60 mL) was added, which led to immediate formation of an orange solid. Stirring was continued for 30 min at –78 °C, followed by exchange of the cooling bath with a water bath at 23 °C. After stirring for 15 h at 23 °C, the suspension was diluted with a saturated aqueous solution of ammonium chloride (25 mL) and diethyl ether (30 mL). The resulting biphasic mixture was filtered through a glass sinter filter and the remaining solid was washed with diethyl ether (30 mL) before being discharged. The organic layer was separated and the aqueous layer was extracted with diethyl ether (2 × 30 mL). The combined organic layers were washed successively with a saturated aqueous solution of sodium chloride (2 × 40 mL), an aqueous hydrogen peroxide solution (10 wt%, 2 × 40 mL), and a saturated aqueous solution of sodium chloride (2 × 40 mL). The washed organic layer was dried over sodium sulfate, the dried organic layer was filtered, and the filtrate was concentrated under reduced pressure. The residue was purified by flash column chromatography on silica gel (10% dichloromethane in *n*-pentane) to yield alkene **S36** (335 mg, 1.53 mmol, 68%) as a colorless oil.

Analytical data of alkene **S36**:

TLC (15% dichloromethane in *n*-pentane): R_f = 0.31 (UV, CAM).

^{1}H NMR (400 MHz, $CDCl_3$): δ 7.10 (d, *J* = 8.5 Hz, 2H), 6.83 (d, *J* = 8.7 Hz, 2H), 5.12 (thept, *J* = 7.1, 1.4 Hz, 1H), 3.79 (s, 3H), 2.56 (t, *J* = 7.7 Hz, 2H), 2.00 (q, *J* = 7.4 Hz, 2H), 1.69 (q, *J* = 1.1 Hz, 3H), 1.64 – 1.55 (m, 5H), 1.37 (h, *J* = 7.0 Hz, 2H).

^{13}C NMR (101 MHz, $CDCl_3$): δ 157.8, 135.1, 131.5, 129.4 (2C), 124.8, 113.8 (2C), 55.4, 35.1, 31.5, 29.6, 28.0, 25.9, 17.8.

IR (ATR, neat): $\tilde{\nu}$ = 2926 (m), 2854 (w), 2834 (w), 1612 (w), 1511 (s), 1454 (m), 1441 (m), 1376 (w), 1299 (m), 1242 (s), 1176 (m), 1111 (w), 1037 (m), 825 (m), 806 (m), 559 (w), 517 (w) cm^{-1}.

HRMS (ESI): calcd for $C_{15}H_{23}O^+$ $[M+H]^+$: 219.1743; found: 219.1743.

2.4.38 Alkene **S37**

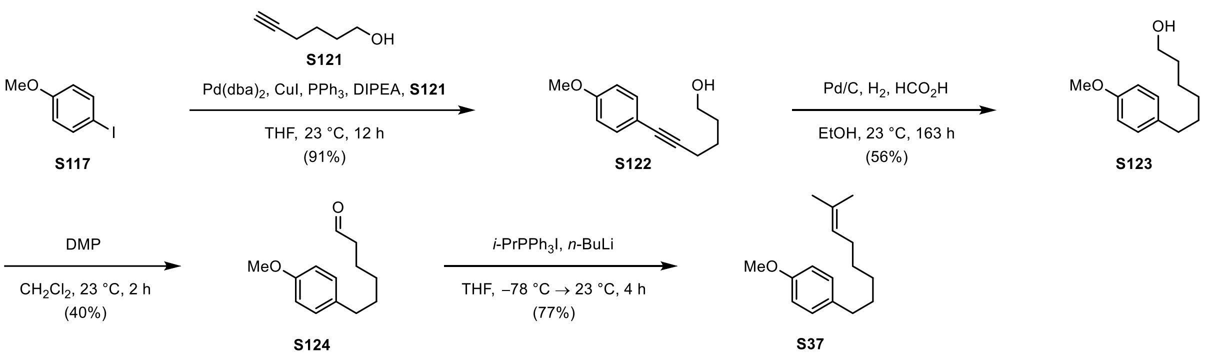

Alkyne **S122** was prepared according to a modified literature procedure[24]: A solution of 4-iodoanisole (**S117**) (1.00 g, 4.27 mmol, 1 equiv), diisopropylethylamine (2.21 g, 17.1 mmol, 4.00 equiv), and hex-5-yn-1-ol (**S121**) (461 mg, 4.70 mmol, 1.10 equiv) in degassed tetrahydrofuran (28.5 mL) was added to a mixture of triphenylphosphine (112 mg, 427 µmol, 0.100 equiv), CuI (81.4 mg, 427 µmol, 0.100 equiv) and bis(dibenzylideneacetone)palladium (246 mg, 427 µmol, 0.100 equiv) at 23 °C under argon atmosphere. After stirring for 12 h at 23 °C, the reaction mixture was diluted with a saturated aqueous solution of ammonium chloride (30 mL), the organic layer was separated, and the aqueous layer was extracted with ethyl acetate (3 × 30 mL). The combined organic layers were washed with a saturated aqueous solution of sodium chloride (2 × 60 mL), the washed organic layer was dried over sodium sulfate, the dried organic layer was filtered, and the filtrate was concentrated under reduced pressure. The residue was purified by flash column chromatography on silica gel (60% diethyl ether in *n*-pentane) to yield alkyne **S122** (797 mg, 3.90 mmol, 91%) as a brown oil. The obtained analytical data for alkyne **S122** were in accordance with reported literature values.[42]

Alcohol **S123** was prepared according to a modified literature procedure[24]: To a suspension of palladium on carbon (10.0 wt%, 83.0 mg, 78.0 µmol, 2.00 mol%) and alkyne **S122** (797 mg, 3.90 mmol, 1 equiv) in ethanol (15.6 mL) was added formic acid (898 mg, 19.5 mmol, 5.00 equiv) under argon atmosphere at 23 °C. The resulting black suspension was flushed with hydrogen for 5 min and stirred at 23 °C for 7 d under a hydrogen atmosphere. The reaction mixture was filtered through a celite plug and the filtrate was concentrated under reduced pressure. The residue was purified by flash column chromatography on silica gel (40% diethyl ether in *n*-pentane) to yield alcohol **S123** (453 mg, 2.17 mmol, 56%) as a colorless oil. The obtained analytical data for alcohol **S123** were in accordance with reported literature values.[24]

Preparation of aldehyde **S124**: To a solution of alcohol **S123** (453 mg, 2.17 mmol, 1 equiv) in dichloromethane (7.25 mL) was added Dess–Martin periodinane (1.11 g, 2.61 mmol, 1.20 equiv) at 23 °C. After stirring for 2 h at 23 °C, a saturated aqueous solution of sodium thiosulfate (10 mL) was added, the organic layer was separated, and the aqueous layer was extracted with dichloromethane (3 × 10 mL). The combined organic layers were washed in succession with a saturated aqueous solution of sodium thiosulfate (2 × 20 mL), a saturated aqueous solution of sodium bicarbonate (2 × 20 mL) and a saturated aqueous solution of sodium chloride (20 mL). The washed organic layer was dried over sodium sulfate, the dried organic layer was filtered, and the filtrate was concentrated under reduced pressure. The residue was purified by flash column chromatography on silica gel (40% dichloromethane in *n*-pentane) to yield aldehyde **S124** (178 mg, 863 µmol, 40%) as a colorless oil. The obtained analytical data for aldehyde **S124** were in accordance with reported literature values.[24]

Preparation of alkene **S37**: To a suspension of isopropyltriphenylphosphonium iodide (411 mg, 950 µmol, 1.80 equiv) in tetrahydrofuran (2.60 mL) was added a solution of *n*-butyllithium (1.60 M in hexanes, 561 µL, 897 µmol, 1.70 equiv) at –78 °C, upon which the yellow suspension turned red. After stirring for 30 min at –78 °C, the reaction mixture was warmed to 23 °C through exchange of the cooling bath with a water bath. After stirring for 30 min at 23 °C, the red reaction mixture was cooled to –78 °C and a solution of aldehyde **S124** (109 mg, 528 µmol, 1 equiv) in tetrahydrofuran (412 µL) was added, which led to a color change from red to orange. Stirring was continued for 30 min at –78 °C, followed by exchange of the cooling bath with a water bath at 23 °C. After stirring for 2 h at 23 °C, the reaction mixture was diluted with a saturated aqueous solution of ammonium chloride (10 mL) and diethyl ether (10 mL), which resulted in decolorization of the organic layer. The resulting biphasic mixture was filtered through a glass sinter filter and the remaining solid was washed with diethyl ether (10 mL) before being discharged. The organic layer was separated and the aqueous layer was extracted with diethyl ether (2 × 10 mL). The combined organic layers were washed

successively with a saturated aqueous solution of sodium chloride (2 × 20 mL), an aqueous hydrogen peroxide solution (10 wt%, 2 × 20 mL), and a saturated aqueous solution of sodium chloride (2 × 20 mL). The washed organic layer was dried over sodium sulfate, the dried organic layer was filtered, and the filtrate was concentrated under reduced pressure. The residue was purified by flash column chromatography on silica gel (10% diethyl ether in *n*-pentane) to yield alkene **S37** (94.5 mg, 407 µmol, 77%) as a colorless oil.

Analytical data of alkene **S37**:

TLC (10% dichloromethane in *n*-pentane): R_f = 0.27 (UV, CAM).

^{1}H NMR (400 MHz, $CDCl_3$): δ 7.09 (d, *J* = 8.6 Hz, 2H), 6.82 (d, *J* = 8.9 Hz, 2H), 5.11 (thept, *J* = 7.1, 1.4 Hz, 1H), 3.79 (s, 3H), 2.54 (dd, *J* = 7.8, 7.0 Hz, 2H), 1.96 (q, *J* = 6.9 Hz, 2H), 1.69 (s, 3H), 1.62 – 1.55 (m, 5H), 1.37 – 1.29 (m, 4H).

^{13}C NMR (101 MHz, $CDCl_3$): δ 157.7, 135.2, 131.4, 129.4 (2C), 125.0, 113.8 (2C), 55.4, 35.2, 31.9, 29.9, 29.1, 28.1, 25.9, 17.8.

IR (ATR, neat): $\tilde{\nu}$ = 2926 (m), 2854 (m), 1613 (w), 1584 (w), 1512 (s), 1510 (s), 1464 (w), 1441 (w), 1376 (w), 1300 (w), 1246 (s), 1177 (w), 1112 (w), 1039 (m), 828 (w) cm^{-1}.

HRMS (ESI): calcd for $C_{16}H_{25}O^+$ [M+H]$^+$: 233.1900; found: 233.1894.

2.4.39 Alkene **S38**

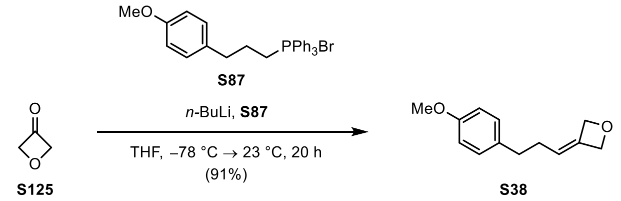

Preparation of alkene **S38**: To a suspension of phosphonium salt **S87** (3.06 g, 6.22 mmol, 1.40 equiv) in tetrahydrofuran (22.2 mL) was added a solution of *n*-butyllithium (1.60 M in hexanes, 3.75 mL, 6.00 mmol, 1.35 equiv) at –78 °C, upon which the white suspension turned pumpkin orange. After stirring for 30 min at –78 °C, the reaction mixture was warmed to 23 °C through exchange of the cooling bath with a water bath. After stirring for 30 min at 23 °C, the reaction mixture was cooled to –78 °C and a solution of oxetane-3-one (**S125**) (320 mg, 4.44 mmol, 1 equiv) in tetrahydrofuran (3.50 mL) was added. After stirring for 30 min at – 78 °C, the reaction mixture was warmed to 23 °C through exchange of the cooling bath with a water bath. After stirring for 18 h at 23 °C, a saturated aqueous solution of ammonium chloride (25 mL) was added, which resulted in decolorization of the reaction mixture. The

resulting suspension was filtered through a glass sinter filter and the remaining solid was washed with diethyl ether (30 mL) before being discharged. The organic layer was separated and the aqueous layer was extracted with diethyl ether (3 × 30 mL). The combined organic layers were washed with a saturated aqueous solution of sodium chloride (2 × 50 mL), the washed organic layer was dried over sodium sulfate, the dried organic layer was filtered, and the filtrate was concentrated under reduced pressure. The residue was purified by flash column chromatography on silica gel (20% diethyl ether in *n*-pentane) to yield alkene **S38** (828 mg, 4.05 mmol, 91%) as a colorless oil.

Analytical data of alkene **S38**:

TLC (20% diethyl ether in *n*-pentane): R_f = 0.40 (UV, CAM).

^{1}H NMR (400 MHz, $CDCl_3$): δ 7.11 – 7.05 (m, 2H), 6.87 – 6.80 (m, 2H), 5.18 – 5.13 (m, 3H), 5.11 – 5.08 (m, 2H), 3.79 (s, 3H), 2.61 (t, *J* = 7.5 Hz, 2H), 2.17 – 2.09 (m, 2H).

^{13}C NMR (101 MHz, $CDCl_3$): δ 158.0, 134.8, 133.7 (2C), 129.4, 118.8, 113.9 (2C), 79.5, 78.9, 55.4, 34.6, 30.4.

IR (ATR, neat): $\tilde{\nu}$ = 2995 (w), 2919 (w), 2853 (m), 1611 (w), 1584 (w), 1510 (s), 1463 (w), 1454 (w), 1442 (w), 1299 (m), 1242 (s), 1177 (m), 1115 (w), 1035 (m), 957 (s), 854 (m), 824 (m), 807 (m), 753 (w), 692 (w), 562 (w), 521 (w) cm^{-1}.

HRMS (ESI): calcd for $C_{13}H_{16}O_2Na^+$ $[M+Na]^+$: 227.1043; found: 227.1042.

2.4.40 Alkene **S39**

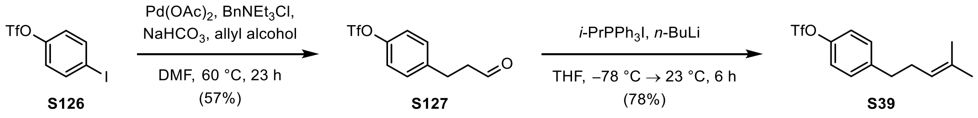

Aldehyde **S127** was prepared according to a modified literature procedure[43]: To a suspension of sodium bicarbonate (895 mg, 10.7 mmol, 2.50 equiv), benzyltriethylammonium chloride (971 mg, 4.26 mmol, 1.00 equiv) and palladium diacetate (47.8 mg, 213 µmol, 5.00 mol%) in dimethylformamide (14.2 mL) was added successively 4-iodophenyl triflate **S126** (1.50 g, 4.26 mmol, 1 equiv) and allyl alcohol (285 mg, 4.90 mmol, 1.15 equiv) at 23 °C under argon atmosphere and light protection. The reaction mixture was heated to 60 °C for 22 h, after which the resulting orange suspension was allowed to cool to 23 °C, diluted with ethyl acetate (80 mL), and washed with a saturated aqueous solution of sodium chloride (2 × 80 mL). The washed organic layer was dried over sodium sulfate, the dried organic layer was filtered, and the filtrate was concentrated under reduced pressure. The residue was purified by flash

column chromatography on silica gel (15% to 25% diethyl ether in *n*-pentane) to yield aldehyde **S127** (685 mg, 2.42 mmol, 57%) as a yellowish oil. The obtained analytical data for aldehyde **S127** were in accordance with reported literature values.[43]

Preparation of alkene **S39**: To a suspension of isopropyltriphenylphosphonium iodide (1.93 g, 4.46 mmol, 1.85 equiv) in tetrahydrofuran (12.0 mL) was added a solution of *n*-butyllithium (1.60 M in hexanes, 2.64 mL, 4.22 mmol, 1.75 equiv) at –78 °C, upon which the white suspension turned yellow. After stirring for 30 min at –78 °C, the reaction mixture was warmed to 23 °C through exchange of the cooling bath with a water bath. After stirring for 30 min at 23 °C, the resulting red solution was cooled to –78 °C and a solution of aldehyde **S127** (680 mg, 2.41 mmol, 1 equiv) in tetrahydrofuran (1.90 mL) was added, which led to immediate formation of an orange solid. After stirring for 30 min at –78 °C, the reaction mixture was warmed to 23 °C through exchange of the cooling bath with a water bath. After stirring for 4 h at 23 °C, the reaction mixture was diluted with a saturated aqueous solution of ammonium chloride (25 mL) and diethyl ether (25 mL), which resulted in decolorization of the reaction mixture. The resulting mixture was filtered through a glass sinter filter and the remaining solid was washed with diethyl ether (20 mL) before being discharged. The organic layer was separated and the aqueous layer was extracted with diethyl ether (2 × 40 mL). The combined organic layers were dried over sodium sulfate, the dried organic layer was filtered, and the filtrate was concentrated under reduced pressure. The residue was purified by flash column chromatography on silica gel (2% to 10% dichloromethane in *n*-pentane) to yield alkene **S39** (578 mg, 1.88 mmol, 78%) as a colorless oil.

<u>Analytical data of alkene **S39**:</u>

TLC (6% dichloromethane in *n*-pentane): R_f = 0.37 (UV, CAM).

^{1}H NMR (400 MHz, $CDCl_3$): δ 7.24 (d, *J* = 8.7 Hz, 2H), 7.17 (d, *J* = 8.7 Hz, 2H), 5.13 (t, *J* = 7.2 Hz, 1H), 2.66 (t, *J* = 7.7 Hz, 2H), 2.29 (q, *J* = 7.6 Hz, 2H), 1.68 (s, 3H), 1.52 (s, 3H).

^{13}C NMR (101 MHz, $CDCl_3$): δ 147.9, 143.1, 133.0, 130.3 (2C), 123.0, 121.1 (2C), 118.9 (q, *J* = 320.8 Hz), 35.6, 29.9, 25.8, 17.7.

^{19}F NMR (376 MHz, $CDCl_3$) δ –72.9.

IR (ATR, neat): $\tilde{\nu}$ = 2970 (w), 2926 (w), 2861 (w), 1500 (m), 1422 (s), 1379 (w), 1249 (m), 1205 (s), 1179 (m), 1135 (s), 1018 (w), 939 (w), 884 (s), 833 (m), 812 (w), 761 (w), 716 (w), 693 (w), 639 (m), 608 (m), 573 (w), 544 (w), 512 (w) cm^{-1}.

HRMS (ESI): calcd for $C_{13}H_{16}F_3O_3S^+$ $[M+H]^+$: 309.0767; found: 309.0767.

2.4.41 Enol ether **S40**

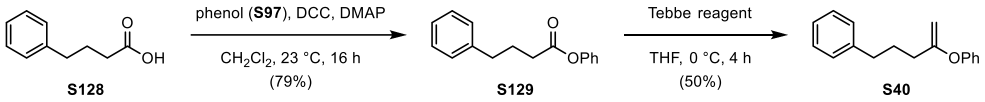

Phenyl ester **S129** was prepared according to a modified literature procedure[44]: To a solution of phenol (**S97**) (1.15 g, 12.2 mmol, 1.00 equiv), 4-dimethylaminopyridine (149 mg, 1.22 mmol, 0.100 equiv), and acid **S128** (2.00 g, 12.2 mmol, 1 equiv) in dichloromethane (122 mL) was added *N*,*N'*-dicyclohexylcarbodiimide (2.76 g, 13.4 mmol, 1.10 equiv) at 23 °C under argon atmosphere. The resulting yellow suspension was stirred for 16 h at 23 °C, after which the reaction mixture was concentrated under reduced pressure. The residue was purified by flash column chromatography on silica gel (7% to 10% diethyl ether in *n*-pentane) to yield phenyl ester **S129** (2.30 g, 9.56 mmol, 79%) as a colorless oil. The obtained analytical data for phenyl ester **S129** were in accordance with reported literature values.[45]

Preparation of enol ether **S40**: To a solution of phenyl ester **S129** (950 mg, 3.95 mmol, 1 equiv) in tetrahydrofuran (3.95 mL) was added a solution of Tebbe reagent (0.500 M in toluene, 9.88 mL, 4.94 mmol, 1.25 equiv) at 0 °C, which resulted in a deep red reaction mixture. After stirring for 4 h at 0 °C, a saturated aqueous solution of ammonium chloride (20 mL) was added at 0 °C. The organic layer was separated and the aqueous layer was extracted with ethyl acetate (3 × 20 mL). The combined organic layers were dried over sodium sulfate, the dried organic layer was filtered, and the filtrate was concentrated under reduced pressure. The residue was purified by flash column chromatography on deactivated silica gel (1% triethylamine and 5% dichloromethane in *n*-pentane) to yield enol ether **S40** (468 mg, 1.96 mmol, 50%) as a colorless oil.

Analytical data of enol ether **S40**:

TLC (5% dichloromethane in *n*-pentane): R_f = 0.40 (UV, CAM).

^{1}H NMR (400 MHz, C_6D_6): δ 7.20 – 7.15 (m, 2H), 7.11 – 7.06 (m, 5H), 7.05 – 7.01 (m, 2H), 6.92 – 6.86 (m, 1H), 4.15 – 4.05 (m, 2H), 2.54 (t, *J* = 7.5 Hz, 2H), 2.23 (t, *J* = 7.7, 7.1 Hz, 2H), 1.94 – 1.85 (m, 2H).

^{13}C NMR (101 MHz, C_6D_6): δ 163.5, 156.1, 142.4, 129.9 (2C), 128.8 (2C), 128.7 (2C), 126.2, 124.2, 121.3 (2C), 89.1, 35.5, 33.9, 29.3.

IR (ATR, neat): $\tilde{\nu}$ = 3062 (w), 3027 (w), 2936 (w), 1611 (w), 2861 (w), 1656 (w), 1635 (w), 1593 (m), 1454 (w), 1434 (w), 1330 (w), 1301 (w), 1220 (s), 1161 (w), 1070 (w), 1024 (w), 958 (m), 899 (w), 817 (w), 748 (m), 693 (m), 505 (w) cm^{-1}.

HRMS (ESI): calcd for $C_{17}H_{19}O^+$ $[M+H]^+$: 239.1430; found: 239.1428.

2.4.42 Epoxide **S42**

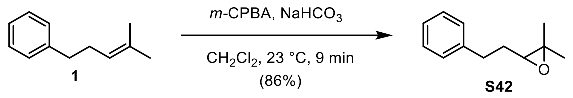

Epoxide **S42** was prepared according to a modified literature procedure[46]: To a solution of alkene **1** (120 mg, 749 µmol, 1 equiv) in dichloromethane (5.00 mL) was added in succession sodium bicarbonate (189 mg, 2.25 mmol, 3.00 equiv) and *meta*-chloroperoxybenzoic acid (218 mg, 77.0% wt, 973 µmol, 1.30 equiv) at 23 °C. After stirring for 9 min at 23 °C, the reaction mixture was diluted with diethyl ether (10 mL) and washed in succession with a saturated aqueous solution of sodium sulfite (5 mL), a saturated aqueous solution of sodium bicarbonate (2 × 5 mL), and a saturated aqueous solution of sodium chloride (5 mL). The washed organic layer was dried over sodium sulfate, the dried organic layer was filtered, and the filtrate was concentrated under reduced pressure. Purification by flash column chromatography on silica gel (10% to 20% diethyl ether in *n*-pentane) afforded epoxide **S42** (114 mg, 644 µmol, 86%) as a colorless liquid. The obtained analytical data for epoxide **S42** were in accordance with reported literature values.[46]

2.4.43 Alkyne **S43**

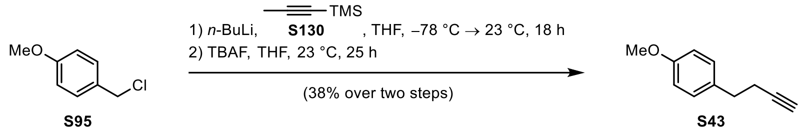

Alkyne **S43** was prepared according to a modified literature procedure[47]: To a solution of 1-(trimethylsilyl)propyne (**S130**) (860 mg, 7.66 mmol, 1.20 equiv) in tetrahydrofuran (14.5 mL) was added a solution of *n*-butyllithium (1.60 M in hexanes, 4.39 mL, 7.02 mmol, 1.10 equiv) at –78 °C. After stirring for 1 h 30 min at –78 °C, 4-methoxybenzyl chloride (**S95**) (1.00 g, 6.39 mmol, 1 equiv) was added at –78 °C and the reaction mixture was allowed to slowly warm up in the cooling bath to 23 °C. After stirring for 16 h 30 min, a saturated aqueous solution of sodium chloride (30 mL) was added, the organic layer was separated, and the aqueous layer was extracted with diethyl ether (2 × 30 mL). The combined organic layers were washed with a saturated aqueous solution of sodium chloride (30 mL), the washed organic layer was dried over sodium sulfate, the dried organic layer was filtered, and the filtrate was concentrated under reduced pressure. The residue was directly subjected to the next step without further purification. To a solution of the intermediate crude TMS-protected alkyne (in theory: 6.39 mmol, 1 equiv) in tetrahydrofuran (10.0 mL) was added a solution of tetrabutylammonium

fluoride (1.00 M in tetrahydrofuran, 7.66 mL, 7.66 mmol, 1.20 equiv) at 23 °C. After stirring for 25 h at 23 °C, a 2 M aqueous solution of hydrochloric acid (15 mL) was added and the biphasic mixture was extracted with ethyl acetate (4 × 15 mL). The combined organic layers were dried over sodium sulfate, the dried solution was filtered, and the filtrate was concentrated under reduced pressure. The residue was purified by flash column chromatography on silica gel (10% dichloromethane in *n*-pentane) to yield alkyne **S43** (384 mg, 2.40 mmol, 38%) as a colorless oil. The obtained analytical data for alkyne **S43** were in accordance with reported literature values.[48]

2.4.44 Alkyne **S44**

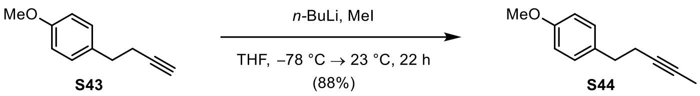

Preparation of alkyne **S44**: To a solution of alkyne **S43** (83.5 mg, 521 µmol, 1 equiv) in tetrahydrofuran (3.50 mL) was added a solution of *n*-butyllithium (1.60 M in hexanes, 423 µL, 678 µmol, 1.30 equiv) at –78 °C. After stirring for 2 h at –78 °C, methyl iodide (50.6 µL, 782 µmol, 1.50 equiv) was added at –78 °C and the reaction mixture was allowed to slowly warm up in the cooling bath to 23 °C. After stirring for 22 h, a saturated aqueous solution of ammonium chloride (20 mL) and diethyl ether (20 mL) were added. The organic layer was separated, and the aqueous layer was extracted with diethyl ether (2 × 20 mL). The combined organic layers were washed with a saturated aqueous solution of sodium chloride (20 mL), the washed organic layer was dried over sodium sulfate, the dried organic layer was filtered, and the filtrate was concentrated under reduced pressure. The residue was purified by flash column chromatography on silica gel (15% to 25% dichloromethane in *n*-pentane) to yield alkyne **S44** (80.3 mg, 461 µmol, 88%) as a yellowish oil.

<u>Analytical data of alkyne **S44**:</u>

TLC (30% dichloromethane in *n*-pentane): R_f = 0.46 (UV, CAM).

^{1}H NMR (400 MHz, $CDCl_3$): δ 7.14 (d, *J* = 8.6 Hz, 2H), 6.84 (d, *J* = 8.6 Hz, 2H), 3.79 (s, 3H), 2.75 (t, *J* = 7.6 Hz, 2H), 2.39 (tq, *J* = 7.6, 2.5 Hz, 2H), 1.79 (t, *J* = 2.6 Hz, 3H).

^{13}C NMR (101 MHz, $CDCl_3$): δ 158.1, 133.3, 129.5 (2C), 113.9 (2C), 78.8, 76.2, 55.4, 34.8, 21.4, 3.6.

IR (ATR, neat): $\tilde{\nu}$ = 2996 (w), 2918 (w), 2858 (w), 2834 (w), 1612 (w), 1584 (w), 1511 (s), 1466 (w), 1440 (w), 1341 (w), 1300 (m), 1243 (s), 1177 (m), 1107 (w), 1036 (m), 853 (w), 822 (m), 704 (w), 565 (w), 524 (w) cm^{-1}.

HRMS (ESI): calcd for $C_{12}H_{15}O^+$ $[M+H]^+$: 175.1117; found: 175.1116.

2.4.45 Allene **S45**

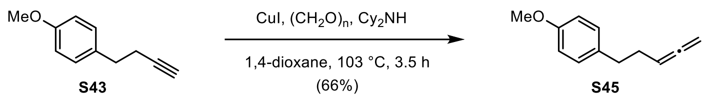

Allene **S45** was prepared according to a modified literature procedure[49]: To a pressure tube charged with copper(I) iodide (57.0 mg, 299 µmol, 0.500 equiv) and paraformaldehyde (44.9 mg, 1.50 mmol, 2.50 equiv) was added in succession a solution of alkyne **S43** (95.8 mg, 598 µmol, 1 equiv) in 1,4-dioxane (3.00 mL) and dicyclohexylamine (195 mg, 1.08 mmol, 1.80 equiv) at 23 °C. The yellow reaction mixture was stirred for 3.5 h at 103 °C, after which the reaction mixture was cooled to 23 °C and water (5 mL) was added. The biphasic mixture was extracted with diethyl ether (3 × 5 mL), the combined organic layers were dried over sodium sulfate, the dried organic layer was filtered, and the filtrate was concentrated under reduced pressure. The residue was purified by flash column chromatography on silica gel (15% to 25% dichloromethane in *n*-pentane) to yield allene **S45** (69.1 mg, 397 µmol, 66%) as a colorless oil. The obtained analytical data for allene **S45** were in accordance with reported literature values.[50]

2.4.46 Monosubstituted alkene **S46**

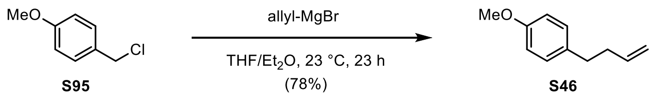

Monosubstituted alkene **S46** was prepared according to a modified literature procedure[51]: To a solution of 4-methoxybenzyl chloride (**S95**) (501 mg, 3.20 mmol, 1 equiv) in tetrahydrofuran (5.30 mL) was added dropwise a solution of allylmagnesium bromide (1.00 M in diethyl ether, 6.39 mL, 6.39 mmol, 2.00 equiv) at 23 °C. After stirring for 23 h at 23 °C, a saturated aqueous solution of ammonium chloride (40 mL) was added cautiously to the reaction mixture. The organic layer was separated and the aqueous layer was extracted with dichloromethane (3 × 40 mL). The combined organic layers were dried over sodium sulfate, the dried solution was filtered, and the filtrate was concentrated under reduced pressure. The residue was purified by flash column chromatography on silica gel (15% dichloromethane in *n*-pentane) to yield monosubstituted alkene **S46** (403 mg, 2.48 mmol, 78%) as a colorless oil. The obtained analytical data for monosubstituted alkene **S46** were in accordance with reported literature values.[51]

2.4.47 Alkene **S47**

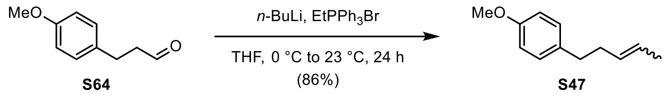

Preparation of alkene **S47**: To a solution of ethyltriphenylphosphonium bromide (1.36 g, 3.65 mmol, 1.20 equiv) in tetrahydrofuran (25.0 mL) was added dropwise a solution of *n*-butyllithium (2.09 mL, 1.60 M in hexanes, 3.35 mmol, 1.10 equiv) at 0 °C. After stirring for 30 min at 0 °C, a solution of 3-(4-methoxyphenyl)propanal (**S64**) (500 mg, 3.05 mmol, 1 equiv) in tetrahydrofuran (5.00 mL) was added and the resulting solution was kept a 0 °C for 20 min before being warmed to 23 °C. After stirring for 23 h at 23 °C, a saturated aqueous solution of ammonium chloride (40 mL) was added. The organic layer was separated and the aqueous layer was extracted with diethyl ether (3 × 40 mL). The combined organic layers were dried over sodium sulfate, the dried organic layer was filtered, and the filtrate was concentrated under reduced pressure. The residue was purified by flash column chromatography on silica gel (*n*-pentane) to afford alkene **S47** (460 mg, 2.61 mmol, 86%) as a colorless oil. The obtained analytical data for alkene **S47** were in accordance with reported literature values.[52]

2.4.48 Alkene **S48**

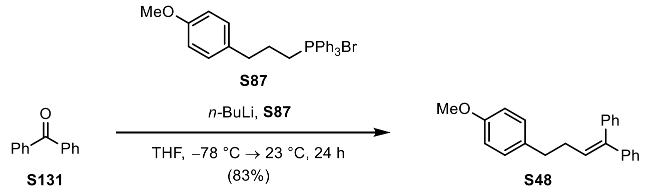

Preparation of Alkene **S48**: To a suspension of phosphonium salt **S87** (2.66 g, 5.41 mmol, 1.40 equiv) in tetrahydrofuran (19.3 mL) was added a solution of *n*-butyllithium (1.60 M in hexanes, 3.26 mL, 5.22 mmol, 1.35 equiv) at –78 °C, upon which the white suspension turned orange. After stirring for 30 min at –78 °C, the reaction mixture was warmed to 23 °C through exchange of the cooling bath with a water bath. After stirring for 30 min at 23 °C, the red reaction mixture was cooled to –78 °C and a solution of benzophenone (**S131**) (705 mg, 3.87 mmol, 1 equiv) in tetrahydrofuran (3.02 mL) was added, which resulted in a color change to orange and immediate formation of an off-white solid. After stirring for 45 min at –78 °C, the reaction mixture was warmed to 23 °C through exchange of the cooling bath with a water bath. After stirring for 22 h at 23 °C, diethyl ether (40 mL) and a saturated aqueous solution of ammonium chloride (40 mL) were added, which resulted in decolorization of the reaction mixture. The resulting suspension was filtered through a glass sinter filter and the remaining

solid was washed with diethyl ether (50 mL) before being discharged. The organic layer was separated and the aqueous layer was extracted with diethyl ether (2 × 50 mL). The combined organic layers were dried over sodium sulfate, the dried organic layer was filtered, and the filtrate was concentrated under reduced pressure. The residue was purified by flash column chromatography on silica gel (15% to 25% dichloromethane in *n*-pentane) to yield alkene **S48** (1.00 g, 3.19 mmol, 83%) as a white solid. The obtained analytical data for alkene **S48** were in accordance with reported literature values.[52]

2.4.49 Diene **S49**

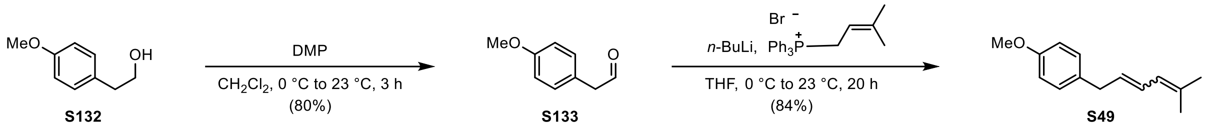

Preparation of 2-(4-methoxyphenyl)acetaldehyde **S133**: To a solution of 2-(4-methoxyphenyl)ethanol (**S132**) (3.10 g, 20.4 mmol, 1 equiv) in dichloromethane (40.0 mL) was added Dess–Martin periodinane (9.07 g, 21.4 mmol, 1.05 equiv) over a duration of 5 min at 0 °C. The reaction mixture was allowed to warm to 23 °C and stirring was continued for 3 h, after which a saturated aqueous solution of sodium thiosulfate (50 mL) was added. The organic layer was separated and the aqueous layer was extracted with dichloromethane (3 × 20 mL). The combined organic layers were washed in succession with a saturated aqueous solution of sodium bicarbonate (20 mL) and a saturated aqueous solution of sodium chloride (20 mL). The washed organic layer was dried over sodium sulfate, the dried organic layer was filtered, and the filtrate was concentrated under reduced pressure. The residue was purified by flash column chromatography on silica gel (10% to 20% diethyl ether in *n*-pentane) to afford 2-(4-methoxyphenyl)acetaldehyde (**S133**) (2.45 g, 16.3 mmol, 80%) as a colorless oil. The obtained analytical data for 2-(4-methoxyphenyl)acetaldehyde (**S133**) were in accordance with reported literature values.[53]

Preparation of diene **S49**: To a solution of (3-methyl-2-butenyl)triphenylphosphonium iodide (3.46 g, 7.99 mmol, 1.20 equiv) in tetrahydrofuran (30.0 mL) was added dropwise a solution of *n*-butyllithium (4.58 mL, 1.60 M in hexanes, 7.32 mmol, 1.10 equiv) at 0 °C. After stirring for 30 min at 0 °C, a solution of 2-(4-methoxyphenyl)acetaldehyde (**S133**) (1.00 g, 6.66 mmol, 1 equiv) in tetrahydrofuran (5.00 mL) was added and the resulting solution was kept at 0 °C for 20 min before being warmed to 23 °C. Stirring was continued for 16 h, after which a saturated aqueous solution of ammonium chloride (50 mL) was added. The organic layer was separated and the aqueous layer was extracted with diethyl ether (3 × 50 mL). The combined organic layers were dried over sodium sulfate, the dried organic layer was filtered, and the

filtrate was concentrated under reduced pressure. The residue was purified by flash column chromatography on silica gel (5% to 10% diethyl ether in *n*-pentane) to afford diene **S49** (1.13 g, 5.59 mmol, *E*:*Z* = 1:1, 84%) as a colorless oil.

Analytical data of diene **S49**:

TLC (*n*-pentane): R_f = 0.27 (UV, CAM).

^{1}H NMR (400 MHz, $CDCl_3$): δ 7.17 – 7.11 (m, 2H, (*Z*)-**S49**, 2H, (*E*)-**S49**), 6.86 (d, *J* = 8.6 Hz, 2H, (*Z*)-**S49**, 2H, (*E*)-**S49**), 6.39 – 6.26 (m, 1H, (*Z*)-**S49**, 1H, (*E*)-**S49**), 6.24 (d, *J* = 12.5 Hz, 1H, (*Z*)-**S49**), 5.85 (d, *J* = 10.8 Hz, 1H, (*E*)-**S49**), 5.70 (dt, *J* = 14.5, 7.0 Hz, 1H, (*E*)-**S49**), 5.56 – 5.45 (m, 1H, (*Z*)-**S49**), 3.81 (s, 3H, (*Z*)-**S49**, 3H, (*E*)-**S49**), 3.50 (d, *J* = 7.0 Hz, 2H, (*Z*)-**S49**), 3.40 (d, *J* = 7.0 Hz, 2H, (*E*)-**S49**), 1.87 (s, 3H, (*Z*)-**S49**), 1.82 (s, 3H, (*Z*)-**S49**), 1.79 (s, 3H, (*E*)-**S49**), 1.78 (s, 3H, (*E*)-**S49**).

^{13}C NMR (101 MHz, $CDCl_3$): δ 158.0 ((*Z*)-**S49**), 158.0 ((*E*)-**S49**), 136.2 ((*Z*)-**S49**), 133.8 ((*E*)-**S49**), 133.2 ((*Z*)-**S49**), 132.9 ((*E*)-**S49**), 130.5 ((*E*)-**S49**), 129.5 (2C, (*Z*)-**S49**), 129.4 (2C, (*E*)-**S49**), 127.8 ((*Z*)-**S49**), 127.8 ((*E*)-**S49**), 125.4 ((*Z*)-**S49**), 125.0 ((*E*)-**S49**), 120.2 ((*Z*)-**S49**), 114.0 (2C, (*Z*)-**S49**), 113.9 (2C, (*E*)-**S49**), 55.4 (1C, (*Z*)-**S49**, 1C, (*E*)-**S49**), 38.5 ((*E*)-**S49**), 32.9 ((*Z*)-**S49**), 26.5 ((*Z*)-**S49**), 26.0 ((*E*)-**S49**), 18.4 ((*E*)-**S49**), 18.3 ((*Z*)-**S49**).

IR (ATR, neat): $\tilde{\nu}$ = 3021 (w), 2996 (w), 2963 (w), 2909 (w), 2834 (w), 1651 (w), 1610 (w), 1584 (w), 1509 (s), 1463 (w), 1440 (m), 1377 (w), 1358 (w), 1322 (w), 1300 (w), 1243 (s), 1175 (m), 1106 (w), 1037 (m), 986 (w), 959 (w), 911 (w), 846 (w), 827 (m), 815 (m), 777 (w), 715 (w), 571 (w), 538 (w), 520 (w), 461 (w), 444 (w) cm^{-1}.

HRMS (ESI): calcd for $C_{14}H_{19}O^+$ $[M+H]^+$: 203.1430; found: 203.1430.

2.4.50 Diene **S50**

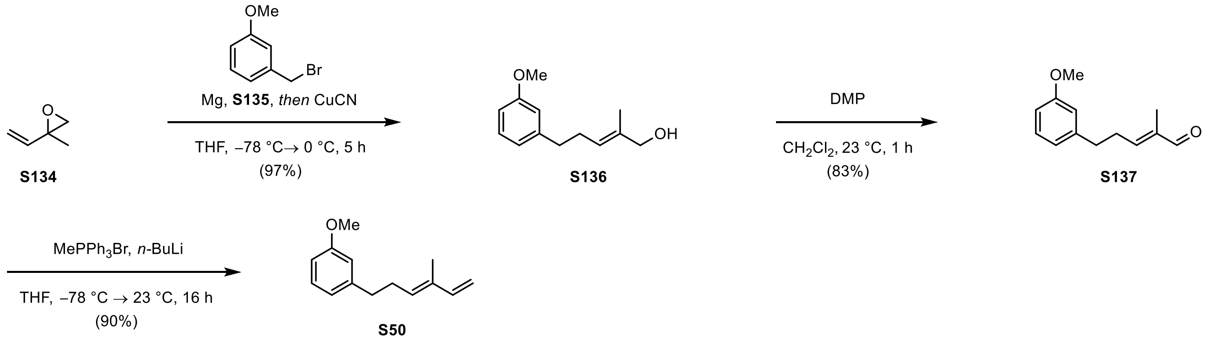

Alcohol **S136** was prepared according to a modified literature procedure[54,55]: To a suspension of activated magnesium turnings[2] (4.84 g, 199 mmol, 10.0 equiv) in tetrahydrofuran (47.0 mL) was added a solution of 3-methoxybenzyl bromide (**S135**) (4.00 g, 19.9 mmol, 1 equiv) in tetrahydrofuran (20.0 mL) slowly at 0 °C. The reaction mixture was stirred at 0 °C for 2 h. Titration of the resulting yellowish Grignard solution using iodide dissolved in a saturated solution of lithium chloride in tetrahydrofuran revealed a Grignard concentration of 0.156 M. The Grignard solution (0.156 M, 67.0 mL, 10.4 mmol, 1.98 equiv) was transferred to a solution of cuprous cyanide (389 mg, 4.34 mmol, 0.830 equiv) in tetrahydrofuran (8.00 mL) at –78 °C. The resulting yellow solution was allowed to warm up to –50 °C. Stirring was continued at – 50 °C for 30 min, after which a solution of 2-methyl-2-vinyloxirane (**S134**) (440 mg, 5.23 mmol, 1 equiv) was added at –50 °C, which resulted in a color change from yellow to orange. Stirring was continued at –50 °C for 3 h, after which the reaction mixture was diluted with a saturated aqueous solution of ammonium chloride (50 mL) and an aqueous solution of ammonia (25 wt%, 50 ml). The organic layer was separated and the aqueous layer was extracted with diethyl ether (3 × 30 mL). The combined organic layers were dried over sodium sulfate, the dried organic layer was filtered, and the filtrate was concentrated under reduced pressure. Purification by automated flash column chromatography on silica gel (5.0% to 30.0% ethyl acetate in *n*-hexane) afforded alcohol **S136** (1.05 g, 5.09 mmol, 97%) as a yellow oil. The obtained analytical data for alcohol **S136** were in accordance with reported literature values.[56]

Preparation of aldehyde **S137**: To a solution of alcohol **S136** (763 mg, 3.70 mmol, 1 equiv) in dichloromethane (12.3 mL) was added Dess–Martin periodinane (1.88 g, 4.44 mmol, 1.20 equiv) at 23 °C. The reaction mixture was stirred at 23 °C for 1 h, after which a saturated aqueous solution of sodium thiosulfate (20 mL) was added. The organic layer was separated and the aqueous layer was extracted with dichloromethane (3 × 10 mL). The combined organic layers were washed in succession with a saturated aqueous solution of sodium thiosulfate (2 × 20 mL), a saturated aqueous solution of sodium bicarbonate (2 × 20 mL), and a saturated aqueous solution of sodium chloride (20 mL). The washed organic layer was dried over sodium sulfate, the dried organic layer was filtered, and the filtrate was concentrated under reduced pressure. The residue was purified by flash column chromatography on silica gel (20% diethyl ether in *n*-pentane) to yield aldehyde **S137** (630 mg, 3.08 mmol, 83%) as a

---

[2] Magnesium turnings were washed in succession with an aqueous solution of hydrochloric acid (1.0 M, 3 × 20 mL), water (3 × 20 mL), acetone (3 × 20 mL), diethyl ether (3 × 20 mL) and dried under reduced pressure. Directly before use, a single iodine crystal was added to the magnesium turnings, which were heated under argon atmosphere in the reaction flask until violet gas formation was observed.

colorless oil. The obtained analytical data for aldehyde **S137** were in accordance with reported literature values.[57]

Preparation of diene **S50**: To a suspension of methyltriphenylphosphonium bromide (1.41 g, 3.95 mmol, 1.45 equiv) in tetrahydrofuran (14.0 mL) was added a solution of *n*-butyllithium (1.60 M in hexanes, 2.38 mL, 3.81 mmol, 1.35 equiv) at –78 °C, upon which the white suspension turned yellow. After stirring for 30 min at –78 °C, the reaction mixture was warmed to 23 °C through exchange of the cooling bath with a water bath. After stirring for 30 min at 23 °C, the red reaction mixture was cooled to –78 °C and a solution of aldehyde **S137** (576 mg, 2.82 mmol, 1 equiv) in tetrahydrofuran (2.20 mL) was added, which led to immediate formation of a pink solid. Stirring was continued for 30 min at –78 °C, followed by exchange of the cooling bath with a water bath at 23 °C. After stirring for 14 h at 23 °C, the reaction mixture was diluted with a saturated aqueous solution of ammonium chloride (20 mL) and diethyl ether (20 mL). The resulting biphasic mixture was filtered through a glass sinter filter and the remaining solid was washed with diethyl ether (20 mL) before being discharged. The organic layer was separated and the aqueous layer was extracted with diethyl ether (2 × 30 mL). The combined organic layers were washed with a saturated aqueous solution of sodium chloride (2 × 40 mL), the washed organic layer was dried over sodium sulfate, the dried solution was filtered, and the filtrate was concentrated under reduced pressure. The residue was purified by flash column chromatography on silica gel (10% dichloromethane in *n*-pentane) to yield diene **S50** (511 mg, 2.53 mmol, 90%) as a colorless oil.

Analytical data of diene **S50**:

TLC (10% dichloromethane in *n*-pentane): R_f = 0.28 (UV, CAM).

^{1}H NMR (400 MHz, $CDCl_3$): δ 7.23 – 7.17 (m, 1H), 6.80 (dt, *J* = 7.5, 1.2 Hz, 1H), 6.77 – 6.71 (m, 2H), 6.37 (dd, *J* = 17.4, 10.7 Hz, 1H), 5.53 (t, *J* = 7.3 Hz, 1H), 5.09 (d, *J* = 17.3 Hz, 1H), 4.94 (d, *J* = 10.7 Hz, 1H), 3.80 (s, 3H), 2.68 (dd, *J* = 9.0, 6.7 Hz, 2H), 2.46 (q, *J* = 7.6 Hz, 2H), 1.71 (s, 3H).

^{13}C NMR (101 MHz, $CDCl_3$): δ 159.8, 143.7, 141.6, 134.7, 132.1, 129.4, 121.0, 114.3, 111.3, 110.9, 55.3, 35.9, 30.2, 11.8.

IR (ATR, neat): $\tilde{\nu}$ = 2939 (w), 2834 (w), 1603 (s), 1583 (m), 1488 (m), 1453 (m), 1436 (w), 1261 (s), 1151 (s), 1083 (w), 1045 (m), 991 (w), 893 (m), 851 (w), 778 (m), 695 (m) cm^{-1}.

HRMS (ESI): calcd for $C_{14}H_{19}O^+$ $[M+H]^+$: 203.1430; found: 203.1430.

2.4.51 Alkene **S51**

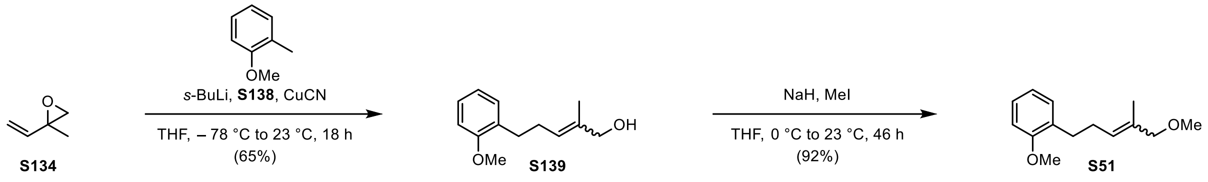

Allylic alcohol **S139** was prepared according to a modified literature procedure[58]: To a solution of 2-methylanisol (**S138**) (4.36 g, 35.7 mmol, 1.50 equiv) in tetrahydrofuran (119 mL) was added dropwise a solution of *s*-butyllithium (34.0 mL, 1.40 M in cyclohexane, 47.7 mmol 2.00 equiv) at –78 °C. The resulting solution was allowed to warm to –20 °C over 3 h. This solution was cooled to –78 °C and cannulated to a suspension of copper(I) cyanide (1.92 g, 21.4 mmol, 0.900 equiv) in tetrahydrofuran (40.0 mL) over a duration of 10 min at –50 °C, which resulted in a color change of the suspension from greenish to red. After stirring for 45 min at –50 °C, 2-methyl-2-vinyloxirane (**S134**) (2.00 g, 23.8 mmol, 1 equiv) was added, which resulted in slight decolorization to orange within 1–2 min. The mixture was allowed to slowly warm up in the cooling bath to 23 °C and stirring was continued for 15 h, after which a saturated aqueous solution of ammonium chloride (150 mL) was added. The resulting biphasic suspension was filtered through celite, the filter cake was washed with diethyl ether (200 mL), and the filtrate was further diluted by addition of a concentrated aqueous solution of ammonia (40 mL). The organic layer was separated and the aqueous layer was extracted with diethyl ether (3 × 150 mL). The combined organic layers were dried over sodium sulfate, the dried organic layer was filtered, and the filtrate was concentrated under reduced pressure. The residue was purified by flash column chromatography on silica gel (20% to 40% diethyl ether in *n*-pentane) to afford allylic alcohol **S139** (3.18 g, 15.5 mmol, *E*:*Z* = 1.0:0.37, 65%) as a yellow oil. For characterization, a small amount of allylic alcohol **S139** (*E*:*Z* = 1.0:0.37) was subjected to preparative normal-phase high performance liquid chromatography (HPLC) (15% to 25% ethyl acetate in *n*-hexane over 60 min) to yield allylic alcohol (*E*)-**S139** as a colorless oil and allylic alcohol (*Z*)-**S139** as a colorless oil.

Analytical data of allylic alcohol (*E*)-**S139**:

TLC (20% ethyl acetate in cyclohexane): R_f = 0.32 (UV, CAM).

^{1}H NMR (400 MHz, $CDCl_3$): δ 7.19 (td, *J* = 7.8, 1.8 Hz, 1H), 7.13 (dd, *J* = 7.4, 1.8 Hz, 1H), 6.89 (td, *J* = 7.6, 1.4 Hz, 1H), 6.85 (dd, *J* = 8.1, 1.1 Hz, 1H), 5.50 (tp, *J* = 7.2, 1.4 Hz, 1H), 3.99 (s, 2H), 3.83 (s, 3H), 2.67 (dd, *J* = 9.0, 6.6 Hz, 2H), 2.34 (q, *J* = 7.4 Hz, 2H), 1.63 (s, 3H).

^{13}C NMR (101 MHz, $CDCl_3$): δ 157.6, 135.3, 130.5, 129.9, 127.2, 126.1, 120.4, 110.3, 69.1, 55.3, 30.3, 27.9, 13.7.

IR (ATR, neat): $\tilde{\nu}$ = 3337 (br), 2918 (w), 2857 (w), 1600 (w), 1587 (w), 1493 (s), 1463 (m), 1438 (m), 1289 (w), 1240 (s), 1176 (w), 1112 (m), 1051 (m), 1031 (m), 1002 (m), 850 (w), 751 (s), 593 (w) cm^{-1}.

HRMS (ESI): calcd for $C_{13}H_{18}O_2Na^+$ $[M+Na]^+$: 229.1199; found: 229.1194.

Analytical data of allylic alcohol (*Z*)-**S139**:

TLC (20% ethyl acetate in cyclohexane): R_f = 0.32 (UV, CAM).

^{1}H NMR (400 MHz, $CDCl_3$): δ 7.19 (td, *J* = 7.8, 1.8 Hz, 1H), 7.09 (dd, *J* = 7.4, 1.8 Hz, 1H), 6.91 – 6.84 (m, 2H), 5.37 (t, *J* = 7.7 Hz, 1H), 3.94 (s, 2H), 3.84 (s, 3H), 2.66 (t, *J* = 7.4 Hz, 2H), 2.33 (qd, *J* = 7.3, 1.2 Hz, 2H), 1.76 (q, *J* = 1.3 Hz, 3H).

^{13}C NMR (101 MHz, $CDCl_3$): δ 157.6, 135.3, 130.3, 130.2, 127.9, 127.4, 120.3, 110.4, 61.6, 55.4, 30.8, 28.0, 21.4.

IR (ATR, neat): $\tilde{\nu}$ = 3344 (br), 2937 (w), 1600 (w), 1587 (w), 1493 (s), 1463 (m), 1438 (m), 1289 (w), 1240 (s), 1177 (w), 1110 (m), 1051 (m), 1030 (m), 1001 (s), 946 (w), 751 (s), 572 (w) cm^{-1}.

HRMS (ESI): calcd for $C_{13}H_{18}O_2Na^+$ $[M+Na]^+$: 229.1199; found: 229.1195.

Preparation of alkene **S51**: To a suspension of sodium hydride (82.0 mg, 60.0 wt%, 2.06 mmol, 1.41 equiv) in tetrahydrofuran (10.0 mL) was added a solution of allylic alcohol **S139** (300 mg, 1.45 mmol, *E*:*Z* = 1.0:0.37, 1 equiv) in tetrahydrofuran (5.00 mL) at 0 °C. Subsequently, the mixture was allowed to warm to 23 °C and stirred for 16 h. Then, methyl iodide (182 µL, 2.91 mmol, 2.00 equiv) was added and the reaction mixture was stirred for 25 h at 23 °C. Then, additional sodium hydride (50.0 mg, 60.0 wt%, 1.25 mmol, 0.860 equiv) was added and stirring was continued for 5 h at 23 °C. Next, triethylamine (223 µL, 1.60 mmol, 1.10 equiv) was added and stirring was continued for 10 min at 23 °C, after which a saturated aqueous solution of ammonium chloride (20 mL) was added. The organic layer was separated and the aqueous layer was extracted with diethyl ether (3 × 15 mL). The combined organic layers were dried over sodium sulfate, the dried organic layer was filtered, and the filtrate was concentrated under reduced pressure. The residue was purified by flash column chromatography on silica gel (10% diethyl ether in *n*-pentane) to afford alkene **S51** (295 mg, 1.45 mmol, *E*:*Z* = 1.0:0.26, 92%) as a colorless oil.

Analytical data of alkene **S51**:

TLC (4% diethyl ether in *n*-pentane): R_f = 0.34 (UV, CAM).

^{1}H NMR (400 MHz, $CDCl_3$): δ 7.21 – 7.10 (m, 2H, (*E*)-**S51**, 2H, (*Z*)-**S51**), 6.92 – 6.82 (m, 2H, (*E*)-**S51**, 2H, (*Z*)-**S51**), 5.52 – 5.44 (m, 1H, (*E*)-**S51**, 1H, (*Z*)-**S51**), 3.88 (s, 2H, (*Z*)-**S51**) 3.83 (s, 3H, (*E*)-**S51**, 3H (*Z*)-**S51**), 3.79 (d, *J* = 1.2 Hz, 2H, (*E*)-**S51**), 3.26 (s, 3H, (*E*)-**S51**), 3.24 (s, 3H, (*Z*)-**S51**), 2.71 – 2.62 (m, 2H, (*E*)-**S51**, 2H, (*Z*)-**S51**), 2.38 – 2.29 (m, 2H, (*E*)-**S51**, 2H, (*Z*)-**S51**), 1.74 (s, 3H, (*Z*)-**S51**), 1.61 (s, 3H, (*E*)-**S51**).

^{13}C NMR (101 MHz, $CDCl_3$): δ 157.6 ((*E*)-**S51**), 157.6 ((*Z*)-**S51**), 132.5 ((*E*)-**S51**), 132.4 ((*Z*)-**S51**), 130.5 ((*E*)-**S51**), 130.4 ((*Z*)-**S51**), 130.0 ((*Z*)-**S51**), 130.0 ((*E*)-**S51**), 129.3 ((*Z*)-**S51**), 128.2 ((*E*)-**S51**), 127.2 ((*Z*)-**S51**), 127.2 ((*E*)-**S51**), 120.5 ((*Z*)-**S51**), 120.4 ((*E*)-**S51**), 110.3 ((*Z*)-**S51**), 110.3 ((*E*)-**S51**), 78.8 ((*E*)-**S51**), 70.8 ((*Z*)-**S51**), 57.5 ((*Z*)-**S51**), 57.4 ((*E*)-**S51**), 55.3 ((*E*)-**S51**, (*Z*)-**S51**), 31.0 ((*Z*)-**S51**), 30.4 ((*E*)-**S51**), 28.1 ((*Z*)-**S51**), 28.1 ((*E*)-**S51**), 21.5 ((*Z*)-**S51**), 13.8 ((*E*)-**S51**).

IR (ATR, neat): $\tilde{\nu}$ = 2920 (w), 2835 (w), 2816 (w), 1601 (w), 1588 (w), 1493 (s), 1463 (m), 1439 (m), 1376 (w), 1354 (w), 1326 (w), 1289 (w), 1240 (s), 1189 (w), 1177 (m), 1160 (w), 1112 (m), 1090 (s), 1051 (m), 1032 (m), 963 (w), 914 (w), 848 (w), 812 (w), 750 (s), 732 (m) cm^{-1}.

HRMS (ESI): calcd for $C_{14}H_{20}O_2Na^+$ $[M+Na]^+$: 243.1356; found: 243.1356.

2.4.52 Alkene **S52**

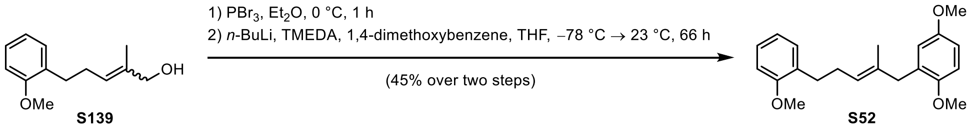

To a solution of allylic alcohol **S139**[3] (1.01 g, 4.91 mmol, 1 equiv) in diethyl ether (12.3 mL) was added dropwise a solution of phosphorus tribromide (664 mg, 2.45 mmol, 0.500 equiv) in diethyl ether (700 µL) at 0 °C. After stirring for 1 h at 0 °C, the reaction mixture was carefully poured onto ice water (50 mL). The biphasic mixture was extracted with diethyl ether (50 mL). The organic layer was sequentially washed with a saturated aqueous solution of sodium chloride (50 mL), a saturated aqueous solution of sodium bicarbonate (50 mL), and a saturated aqueous solution of sodium chloride (50 mL). The washed organic phases was dried over sodium sulfate, the dried solution was filtered, and the filtrate was concentrated under reduced pressure. The residue was used in the next step without further purification.

To a solution of 1,4-dimethoxybenzene (1.36 g, 9.81 mmol, 2.00 equiv) and *N*,*N*,*N′*,*N′*-tetramethylethylenediamine (1.14 g, 9.81 mmol, 2.00 equiv) in tetrahydrofuran

[3]For preparation see chapter 2.4.51.

(32.7 mL) was a solution of *n*-butyllithium (1.60 M in hexanes, 6.13 mL, 9.81 mmol, 2.00 equiv) at –78 °C. The reaction mixture was allowed to warm up from –78 °C to 5 °C over 5 h, during which the solution turned from colorless to yellow. The reaction mixture was cooled to –78 °C and a solution of crude allylic bromide (in theory: 4.91 mmol, 1 equiv) in tetrahydrofuran (8.18 mL) was added. The reaction mixture was allowed to warm up from –78 °C to 23 °C in the cooling bath and stirring was continued for 61 h. A saturated aqueous solution of ammonium chloride (50 mL) was added and the biphasic mixture was extracted with diethyl ether (3 × 50 mL). The combined organic layers were dried over sodium sulfate, the dried solution was filtered, and the filtrate was concentrated under reduced pressure. The residue was purified by flash column chromatography on silica gel (20% to 35% dichloromethane in *n*-pentane) to yield alkene **S52** (722 mg, 2.21 mmol, 45%) as a colorless oil.

Analytical data of alkene **S52**:

TLC (40% dichloromethane in *n*-pentane): R_f = 0.39 (UV, CAM).

^{1}H NMR (400 MHz, $CDCl_3$): δ 7.18 (td, *J* = 7.8, 1.8 Hz, 1H), 7.14 (dd, *J* = 7.4, 1.7 Hz, 1H), 6.91 – 6.83 (m, 2H), 6.82 – 6.77 (m, 1H), 6.74 – 6.69 (m, 2H), 5.29 (t, *J* = 7.1 Hz, 1H), 3.83 (s, 3H), 3.78 (s, 3H), 3.77 (s, 3H), 3.29 (s, 2H), 2.67 (dd, *J* = 9.1, 6.6 Hz, 2H), 2.32 (q, *J* = 7.6 Hz, 2H), 1.57 (s, 3H).

^{13}C NMR (101 MHz, $CDCl_3$): δ 157.6, 153.7, 152.1, 134.3, 130.8, 130.4, 130.0, 127.0, 126.3, 120.4, 116.5, 111.7, 111.1, 110.3, 56.3, 55.8, 55.3, 39.2, 30.6, 28.5, 16.1.

IR (ATR, neat): $\tilde{\nu}$ = 2937 (w), 2832 (w), 1588 (w), 1493 (s), 1463 (m), 1438 (m), 1277 (m), 1240 (s), 1221 (s), 1176 (m), 1156 (w), 1111 (m), 1049 (s), 1031 (m), 930 (w), 858 (w), 799 (w), 752 (s), 710 (w), 451 (w) cm^{-1}.

HRMS (ESI): calcd for $C_{21}H_{26}O_3Na^+$ $[M+Na]^+$: 349.1774; found: 349.1763.

2.4.53 Alkene **S53**

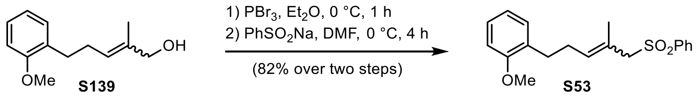

To a solution of allylic alcohol **S139**[4] (300 mg, 1.45 mmol, *E*:*Z* = 1.0:0.37, 1 equiv) in diethyl ether (2.00 mL) was added dropwise a solution of phosphorus tribromide (197 mg, 727 µmol, 0.500 equiv) in diethyl ether (700 µL) at 0 °C. After stirring for 75 min at 0 °C, the reaction mixture was carefully poured onto ice water (10 mL). The organic layer was separated and

[4]See preparation in chapter 2.4.51.

the aqueous layer was extracted with diethyl ether (3 × 10 mL). The combined organic layers were sequentially washed with a saturated aqueous solution of sodium bicarbonate (20 mL) and a saturated aqueous solution of sodium chloride (20 mL). The washed layer was dried over sodium sulfate and filtered. The filtrate was concentrated under reduced pressure and the residue was used in the next step without further purification.

To a solution of sodium benzenesulfinate (335 mg, 2.04 mmol, 1.50 equiv) in *N*,*N*-dimethylformamide (4.00 mL) was added the above-described, crude allylic bromide (in theory: 1.45 mmol, 1 equiv) as a solution in *N*,*N*-dimethylformamide (1.00 mL) at 0 °C. After stirring for 4 h at 0 °C, diethyl ether (30 mL) and water (10 mL) were added. The organic layer was separated and the aqueous layer was extracted with diethyl ether (3 × 10 mL). The combined organic layers were washed with a saturated aqueous solution of sodium chloride (30 mL). The washed organic layer was dried over sodium sulfate and filtered. The filtrate was concentrated under reduced pressure and the residue was purified by flash column chromatography on silica gel (20% ethyl acetate in hexanes) to afford alkene **S53** (392 mg, 1.19 mmol, *E*:*Z* = 1.0:0.40, 82%) as a yellow oil. For characterization, a small amount of alkene **S53** (*E*:*Z* = 1.0:0.40) was subjected to preparative normal-phase high performance liquid chromatography (HPLC) (5% to 15% ethyl acetate in *n*-hexane over 60 min) to yield alkene (*E*)-**S53** as a colorless oil and alkene (*Z*)-**S53** as a colorless oil.

Analytical data of alkene (*Z*)-**S53**:

TLC (20% ethyl acetate in hexanes): R_f = 0.40 (UV, CAM).

^{1}H NMR (400 MHz, $CDCl_3$): δ 7.88 (d, *J* = 6.8 Hz, 2H), 7.62 (t, *J* = 7.4 Hz, 1H), 7.52 (t, *J* = 7.7 Hz, 2H), 7.17 (td, *J* = 7.8, 1.8 Hz, 1H), 6.95 (dd, *J* = 7.4, 1.8 Hz, 1H), 6.83 (dd, *J* = 17.5, 7.9 Hz, 2H), 5.53 (t, *J* = 8.2 Hz, 1H), 3.80 (s, 3H), 3.78 (s, 2H), 2.41 (t, 2H), 1.87 (q, *J* = 7.5 Hz, 2H), 1.83 (s, 3H).

^{13}C NMR (101 MHz, $CDCl_3$): δ 157.5, 139.2, 134.8, 133.7, 129.9, 129.7, 129.2 (2C), 128.6 (2C), 127.4, 123.3, 120.4, 110.3, 59.5, 55.3, 30.0, 28.2, 24.0.

IR (ATR, neat): $\tilde{\nu}$ = 3065 (w), 2923 (m), 2854 (w), 2836 (w), 1600 (w), 1586 (w), 1493 (m), 1464 (m), 1447 (m), 1405 (w), 1382 (w), 1307 (s), 1292 (m), 1242 (s), 1177 (m), 1148 (s), 1132 (s), 1110 (w), 1086 (m), 1071 (w), 1051 (w), 1031 (m), 999 (w), 851 (w), 819 (w), 752 (s), 725 (m), 689 (m), 608 (m), 586 (m), 531 (m) cm^{-1}.

HRMS (ESI): calcd for $C_{19}H_{23}O_3S^+$ $[M+H]^+$: 331.1362; found: 331.1358.

Analytical data of alkene (*E*)-**S53**:

TLC (20% ethyl acetate in hexanes): R_f = 0.35 (UV, CAM).

^{1}H NMR (400 MHz, $CDCl_3$): δ 7.87 – 7.81 (m, 2H), 7.65 – 7.59 (m, 1H), 7.56 – 7.50 (m, 2H), 7.18 (td, *J* = 7.8, 1.8 Hz, 1H), 7.00 (dd, *J* = 7.4, 1.8 Hz, 1H), 6.90 – 6.79 (m, 2H), 5.15 (t, *J* = 7.2 Hz, 1H), 3.81 (s, 3H), 3.72 (s, 2H), 2.46 (dd, *J* = 9.4, 7.2 Hz, 2H), 2.22 (q, *J* = 7.6 Hz, 2H), 1.73 (s, 3H).

^{13}C NMR (101 MHz, $CDCl_3$): δ 157.5, 138.5, 136.1, 133.6, 129.9, 129.8, 129.0 (2C), 128.7 (2C), 127.3, 123.7, 120.5, 110.3, 66.4, 55.3, 29.8, 28.6, 16.7.

IR (ATR, neat): $\tilde{\nu}$ =3064 (w), 2922 (w), 2837 (w), 2363 (w), 1600 (w), 1586 (w), 1493 (s), 1464 (m), 1447 (m), 1402 (w), 1306 (s), 1242 (s), 1162 (m), 1149 (m), 1133 (s), 1112 (m), 1085 (m), 1051 (w), 1030 (m), 999 (w), 930 (w), 884 (w), 812 (w), 753 (s), 726 (m), 689 (m), 618 (w), 595 (w), 570 (w), 544 (m), 525 (m), 469 (w), 443 (w) cm^{-1}.

HRMS (ESI): calcd for $C_{19}H_{23}O_3S^+$ $[M+H]^+$: 331.1362; found: 331.1358.

2.4.54 Cyclopentene ether **S54**

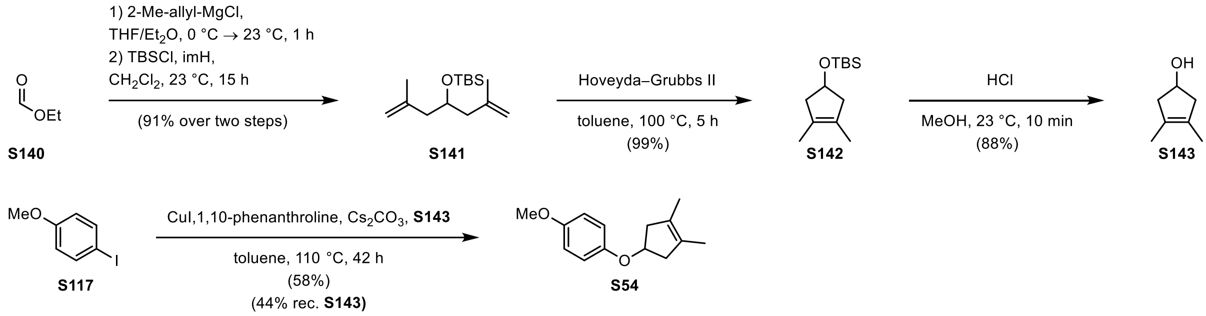

Silyl ether **S141** was prepared according to a modified literature procedure[59]: To a solution of ethyl formate (**S140**) (880 mg, 11.9 mmol, 1 equiv) in diethyl ether (10.0 mL) was added dropwise a solution of 2-methylallylmagnesium chloride (0.500 M in tetrahydrofuran, 49.9 mL, 25.0 mmol, 2.10 equiv) at 0 °C. After stirring for 5 min at 0 °C, the cooling bath was removed and stirring was continued for 1 h at 23 °C. Diethyl ether (40 mL) and a saturated aqueous solution of ammonium chloride (40 mL) were added to the reaction mixture. The organic layer was separated and the aqueous layer was extracted with diethyl ether (2 × 40 mL). The combined organic layers were dried over sodium sulfate, the dried solution was filtered, and the filtrate was concentrated under reduced pressure (40 °C, down to 500 mbar). The residue was directly subjected to the next step without further purification. To a solution of the intermediate crude secondary alcohol (in theory: 11.9 mmol, 1 equiv) in dichloromethane (29.7 mL) was added in succession imidazole (2.43 g, 35.6 mmol, 3.00 equiv) and *tert*-butyldimethylsilyl chloride (2.69 g, 17.8 mmol, 1.50 equiv) at 23 °C. After stirring for 15 h at 23 °C, a saturated aqueous solution of ammonium chloride (40 mL) was added to the reaction

mixture, the organic layer was separated, and the aqueous layer was extracted with dichloromethane (3 × 40 mL). The combined organic layers were dried over sodium sulfate, the dried solution was filtered, and the filtrate was concentrated under reduced pressure. The residue was purified by flash column chromatography on silica gel (1% dichloromethane in *n*-pentane) to silyl ether **S141** (2.74 g, 10.8 mmol, 91% over two steps) as a colorless oil. The obtained analytical data for silyl ether **S141** were in accordance with reported literature values.[60]

Cyclopentene silyl ether **S142** was prepared according to a modified literature procedure[61]: To a solution of silyl ether **S141** (2.61 g, 10.3 mmol, 1 equiv) in degassed toluene (205 mL) was added Grubbs–Hoveyda 2nd generation catalyst (96.4 mg, 154 µmol, 1.50 mol%) at 23 °C. The resulting green solution was stirred for 5 h at 100 °C, during which the reaction mixture turned brown. The reaction mixture was cooled to 23 °C and subjected to flash column chromatography on silica gel (10% dichloromethane in *n*-pentane) to afford cyclopentene silyl ether **S142** (2.30 g, 10.2 mmol, 99%) as a colorless oil. The obtained analytical data for cyclopentene silyl ether **S142** were in accordance with reported literature values.[61]

Secondary alcohol **S143** was prepared according to a modified literature procedure[61]: To a solution of cyclopentene silyl ether **S142** (2.30 g, 10.2 mmol, 1 equiv) in methanol (80.7 mL) was added concentrated hydrochloric acid (110 mg, 37.0 wt% in water, 1.12 mmol, 0.110 equiv) at 23 °C. After stirring for 10 min at 23 °C, a saturated aqueous solution of sodium chloride (250 mL) and diethyl ether (125 mL) were added to the reaction mixture. The organic layer was separated and the aqueous layer was extracted with diethyl ether (2 × 125 mL). The combined organic layers were washed with a saturated aqueous solution of sodium chloride (3 × 50 mL). The washed organic layer was dried over sodium sulfate, the dried solution was filtered, and the filtrate was concentrated under reduced pressure. The residue was purified by flash column chromatography on silica gel (30% diethyl ether in *n*-pentane) to afford secondary alcohol **S143** (1.01 g, 8.99 mmol, 88%) as a colorless oil. The obtained analytical data for secondary alcohol **S143** were in accordance with reported literature values.[61]

Cyclopentene ether **S54** was prepared according to a modified literature procedure[62]: To a vial charged with 1-iodo-4-methoxybenzene (**S117**) (375 mg, 1.60 mmol, 1 equiv), copper(I) iodide (30.5 mg, 160 µmol, 0.100 equiv), 1,10-phenantroline (57.8 mg, 321 µmol, 0.200 equiv), and cesium carbonate (1.04 g, 3.21 mmol, 2.00 equiv) was added a solution of secondary alcohol **S143** (357 mg, 3.18 mmol, 1.98 equiv) in toluene (800 µL) at 23 °C. The reaction mixture was stirred for 42 h at 110 °C, after which the reaction mixture was allowed to cool to 23 °C and subjected to flash column chromatography on silica gel (15% to 50% dichloromethane followed by 2% to 40% diethyl ether in *n*-pentane) to afford cyclopentene

ether **S54** (201 mg, 922 µmol, 58%) as a colorless oil and secondary alcohol **S143** (157 mg, 1.40 mmol, 44% recovery) as a colorless oil.

<u>Analytical data of cyclopentene ether **S54**:</u>

TLC (2% diethyl ether in *n*-pentane): R_f = 0.30 (UV, CAM).

^{1}H NMR (400 MHz, $CDCl_3$): δ 6.86 – 6.78 (m, 4H), 4.81 (tt, *J* = 7.0, 2.5 Hz, 1H), 3.77 (s, 3H), 2.75 (dd, *J* = 16.1, 6.3 Hz, 2H), 2.48 (d, *J* = 16.8 Hz, 2H), 1.65 (s, 6H).

^{13}C NMR (101 MHz, $CDCl_3$): δ 153.7, 152.1, 128.9 (2C), 116.3 (2C), 114.8 (2C), 75.7, 55.8, 45.6 (2C), 13.7 (2C).

IR (ATR, neat): $\tilde{\nu}$ = 2911 (w), 2855 (w), 2832 (w), 1504 (s), 1464 (w), 1440 (m), 1357 (w), 1283 (w), 1226 (s), 1197 (s), 1180 (m), 1106 (w), 1066 (m), 1037 (s), 988 (m), 870 (w), 822 (s), 795 (w), 759 (w), 730 (m), 522 (w) cm^{-1}.

HRMS (ESI): calcd for $C_{14}H_{19}O_2^+$ $[M+H]^+$: 219.1380; found: 219.1379.

2.4.55 TIPS-protected geraniol **S55**

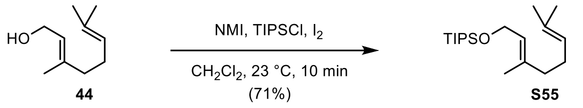

TIPS-protected geraniol **S55** was prepared according to a known literature procedure[63]: To a solution of iodine (3.23 g, 12.7 mmol, 2.00 equiv) in dichloromethane (19.1 mL) was added *N*-methylimidazole (NMI) (1.57 g, 19.1 mmol, 3.00 equiv) at 23 °C, whereupon the deep violet solution turned olive/brown. After 4 min at 23 °C, geraniol (**44**) (982 mg, 6.36 mmol, 1 equiv) was added at 23 °C. Stirring was continued for 10 min at 23 °C, after which a saturated aqueous solution of sodium thiosulfate (50 mL) and diethyl ether (20 mL) were added. The aqueous layer was separated and the organic layer was washed with a saturated aqueous solution of sodium thiosulfate (2 × 50 mL). The washed organic layer was dried over sodium sulfate, the dried organic layer was filtered, and the filtrate was concentrated under reduced pressure. The residue was purified by flash column chromatography on silica gel (10% dichloromethane in *n*-pentane) to yield TIPS-protected geraniol **S55** (1.40 g, 4.51 mmol, 71%) as a colorless oil. The obtained analytical data for TIPS-protected geraniol **S55** were in accordance with reported literature values.[63]

2.4.56 Boc-protected geraniol **S56**

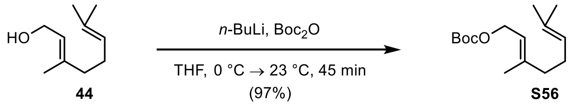

Boc-protected geraniol **S56** was prepared according to a known literature procedure[64]: To a solution of geraniol (**44**) (971 mg, 6.29 mmol, 1 equiv) in tetrahydrofuran (24.3 mL) was added a solution of *n*-butyllithium (1.60 M in hexanes, 3.93 mL, 6.29 mmol, 1.00 equiv) at 0 °C. After stirring for 15 min at 0 °C, di-*tert*-butyl dicarbonate (1.37 g, 6.29 mmol, 1.00 equiv) was added at 0 °C and the reaction mixture was allowed to warm up to 23 °C through removal of the cooling bath. Stirring was continued for 45 min, after which a saturated aqueous solution of sodium hydrogencarbonate (30 mL) and diethyl ether (30 mL) were added. The organic layer was separated and the aqueous layer was extracted with diethyl ether (2 × 30 mL). The combined organic layers were dried over sodium sulfate, the dried organic layer was filtered, and the filtrate was concentrated under reduced pressure. The residue was purified by flash column chromatography on silica gel (3% diethyl ether in *n*-pentane) to yield Boc-protected geraniol **S56** (1.56 g, 6.13 mmol, 97%) as a colorless oil. The obtained analytical data for Boc-protected geraniol **S56** were in accordance with reported literature values.[64]

2.5 Miscellaneous

2.5.1 Influence of base additive on arene methylation

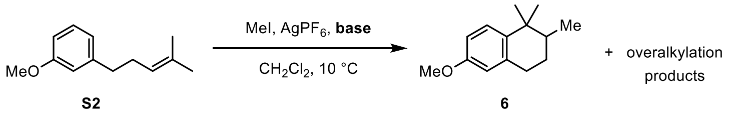

entry	base	time	yield 6	yield S2
1	**B1**	47 h	38%	29%
2	**B2**	22 h	47%	0%
3	**B7**	22 h	47%	0%
4	**B8**	22 h	46%	0%

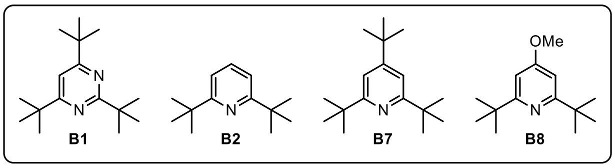

Conclusion: Base **B2** significantly suppresses arene methylation compared to **B1** (entries 1 and 2). Increased electron-density through para-substituents (i.e., **B7** and **B8**) does not result in further improvement compared to **B2**.

General procedure: To a vial charged with silver hexafluorophosphate ($AgPF_6$, 63.2 mg, 250 µmol, 2.00 equiv) in the glovebox was added in succession a solution of the indicated

base (100 mM in dichloromethane, 2.50 mL, 250 µmol, 2.00 equiv), a solution of alkene **S2** (50.0 mM in dichloromethane, 2.50 mL, 125 µmol, 1 equiv), and a solution of methyl iodide (625 mM in dichloromethane, 600 µL, 375 µmol, 3.00 equiv) at 23 °C. The reaction was stirred for the indicated time at 10 °C and stopped by addition of triethylamine (100 µL, 717 µmol, 5.74 equiv). After stirring for 10 min at 23 °C, the solvent was removed under reduced pressure. The residue was purified by flash column chromatography on silica gel (5% to 17% dichloromethane in *n*-pentane) to afford tetralin **6** (yields as indicated) as a colorless oil and recovered alkene **S2** (entry 1, yields as indicated) as a colorless oil.

2.5.2 Screening of electrophiles

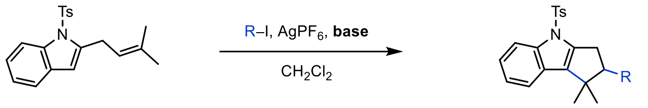

S23 **35**

(**a** = Me, **b** = CH_2F, **c** = Et)

entry	R–I	base	temp.	time	yield 35	yield S23
1	MeI	**B1**	0 °C	25 h	75%	5%
2	FCH_2I	**B1**	23 °C	23 h	36%	—
3	EtI	**B1**	0 °C → 23 °C	45 h	15%[a]	16%[a]
4	EtI	**B2**	23 °C	23 h	23%[a]	29%[a]
5	*i*-PrI	**B1**	0 °C	4 h	complex mixture	
6	allyl iodide	**B1**	0 °C	17 h	complex mixture	
7	CF_3CH_2I	**B1**	40 °C	96 h	—	82%[a]

[a]NMR yield

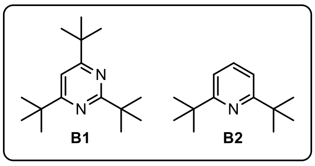

Summary: Fluoroiodomethane (entry 2) and ethyl iodide (entry 3–4) are affording the desired alkylated, cyclized products **35**, albeit in lower yield than for methyl iodide (entry 1). In contrast, isopropyl iodide (entry 5) and ally iodide (entry 6) give complex reaction mixtures, for which NMR analysis indicates at least some degree of unspecific Friedel–Crafts arene alkylation. For 2,2,2-trifluoroethyl iodide (entry 7) no conversion was observed even with elevated temperature and prolonged reaction times. Based on our mechanistic investigations, methylation of the alkene proceeds via a S_N2-type mechanism, in which the methyl group represents a rather soft electrophile. Putatively, allyl iodide and isopropyl iodide directly form the corresponding alkyl cations, which resemble hard electrophiles and preferentially react with the arene over the alkene.

General procedure: A vial was charged with silver hexafluorophosphate ($AgPF_6$, 63.2 mg, 250 µmol, 2.00 equiv) in the glovebox and sealed under Argon atmosphere using a rubber septum. To this vial were added in succession a solution of 2,4,6-tri-*tert*-butylpyrimidine (**B1**)

(entry 4: 2,6-di-*tert*-butylpyridine (**B2**) instead) (100 mM in dichloromethane, 2.50 mL, 250 µmol, 2.00 equiv), a solution of 2-prenyl indole **S23** (50.0 mM in dichloromethane, 2.50 mL, 125 µmol, 1 equiv), and a solution of the indicated iodide (625 mM in dichloromethane, 600 µL, 375 µmol, 3.00 equiv) at the indicated temperature. The reaction was stirred at the indicated temperature for the indicated time (entry 3: 22 h at 0 °C, then 23 h at 23 °C) until no further conversion was observed (NMR or TLC reaction control). The reaction was stopped through the addition of triethylamine (100 µL, 717 µmol, 5.74 equiv). The reaction mixture was allowed to warm to 23 °C and stirred for 10 min, after which the solvent was removed under reduced pressure.

Entry 1: Purification by flash column chromatography on silica gel (3% diethyl ether in *n*-pentane) afforded indole **35**(**a**) (33.1 mg, 93.6 µmol, 75%) as a colorless oil along with recovered 2-prenyl indole **S23** (1.9 mg, 5.6 µmol, 5%) as a colorless oil.

Entry 2: Purification by flash column chromatography on silica gel (5% to 10% diethyl ether in *n*-pentane) afforded indole **35b** (16.5 mg, 44.4 µmol, 36%) as a colorless oil.

<u>Analytical data of indole **35b**:</u>

TLC (10% diethyl ether in *n*-pentane): R_f = 0.37 (UV, CAM).

^{1}H NMR (400 MHz, $CDCl_3$): δ 8.01 (dd, *J* = 8.1, 1.8 Hz, 1H), 7.72 (d, *J* = 8.4 Hz, 2H), 7.41 (dd, *J* = 6.9, 1.6 Hz, 1H), 7.26 – 7.17 (m, 4H), 4.79 – 4.52 (m, 2H), 3.38 – 3.28 (m, 1H), 2.98 – 2.83 (m, 2H), 2.35 (s, 3H), 1.48 (s, 3H), 1.23 (s, 3H).

^{13}C NMR (101 MHz, $CDCl_3$): δ 144.9, 140.1, 139.1, 135.9, 133.7, 130.1 (2C), 126.7 (2C), 126.0, 123.6, 123.4, 118.6, 114.7, 83.9 (d, *J* = 167.5 Hz), 53.8 (d, *J* = 17.3 Hz), 41.4 (d, *J* = 4.2 Hz), 29.9 (d, *J* = 8.7 Hz), 28.7, 22.6, 21.7.

^{19}F NMR (376 MHz, $CDCl_3$) δ –220.4.

IR (ATR, neat): $\tilde{\nu}$ = 2960 (w), 2925 (w), 2868 (w), 1597 (w), 1494 (w), 1445 (m), 1399 (w), 1367 (m), 1345 (w), 1217 (w), 1189 (m), 1172 (s), 1150 (m), 1122 (m), 1093 (m), 1067 (w), 1021 (w), 986 (m), 928 (w), 912 (w), 813 (w), 785 (w), 764 (w), 746 (m), 703 (w), 668 (s), 619 (w), 587 (m), 571 (m), 542 (m), 485 (w), 431 (w) cm^{-1}.

HRMS (ESI): calcd for $C_{21}H_{23}FNO_2S^+$ $[M+H]^+$: 372.1428; found: 372.1424.

Entry 3–7: The residue was filtered through a short silica plug, which was eluted with four column volumes of 30% diethyl ether in *n*-pentane. The filtrate was concentrated under

reduced pressure. To the residue was added 1,1,2,2-tetrachloroethane as an NMR standard and the yield was determined through quantitative NMR analysis.

For characterization, indole **35c** was purified by semipreparative normal-phase high performance liquid chromatography (HPLC) (0.5% to 1.5% ethyl acetate in *n*-hexane over 40 min) to afford indole **35c** as a colorless oil.

Analytical data of indole **35c**:

TLC (5% diethyl ether in *n*-pentane): R_f = 0.40 (UV, CAM).

^{1}H NMR (400 MHz, $CDCl_3$): δ 8.00 (dd, *J* = 7.2, 1.9 Hz, 1H), 7.71 (d, *J* = 8.4 Hz, 2H), 7.41 (dd, *J* = 6.8, 2.0 Hz, 1H), 7.23 – 7.15 (m, 4H), 3.34 (dd, *J* = 16.4, 7.9 Hz, 1H), 2.68 (dd, *J* = 16.5, 9.4 Hz, 1H), 2.34 (s, 3H), 2.37 – 2.29 (m, 1H), 1.69 – 1.60 (m, 1H), 1.49 – 1.42 (m, 1H), 1.41 (s, 3H), 1.06 (t, *J* = 7.4 Hz, 3H), 1.05 (s, 3H).

^{13}C NMR (101 MHz, $CDCl_3$): δ 144.7, 140.3, 139.9, 136.0, 134.6, 130.0 (2C), 126.7 (2C), 126.6, 123.2, 123.2, 118.5, 114.7, 56.9, 41.7, 33.3, 27.5, 22.8, 22.4, 21.7, 13.5.

IR (ATR, neat): $\tilde{\nu}$ = 2958 (m), 2928 (w), 2873 (w), 1598 (w), 1494 (w), 1463 (w), 1445 (m), 1397 (w), 1368 (m), 1342 (w), 1324 (w), 1287 (w), 1218 (w), 1189 (m), 1172 (s), 1151 (w), 1121 (w), 1093 (m), 1045 (w), 1021 (w), 984 (w), 911 (w), 813 (w), 783 (w), 764 (w), 746 (m), 703 (w), 668 (m), 611 (w), 574 (m), 542 (m), 427 (w) cm^{-1}.

HRMS (ESI): calcd for $C_{22}H_{26}NO_2S^+$ $[M+H]^+$: 368.1679; found: 368.1676.

2.5.3 Synthesis of 2,6-(*t*-Bu)$_2$-4-MeO-pyridine **B8**

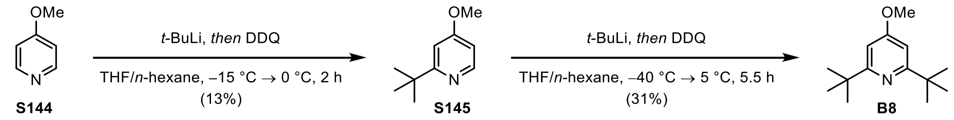

2-(*tert*-butyl)-4-methoxypyridine **S145** was prepared according to a modified literature procedure[65]: To a solution of 4-methoxypyridine (**S144**) (3.00 g, 27.5 mmol, 1 equiv) in dry *n*-hexane (27.5 mL) was added dropwise a solution of *tert*-butyl lithium (1.90 M in *n*-pentane, 15.9 mL, 30.2 mmol, 1.10 equiv) at –15 °C. The resulting deep brown suspension was stirred for 30 min at –15 °C followed by 60 min at 0 °C, after which a solution of 2,3-dichloro-5,6-dicyano-1,4-benzoquinone (DDQ) (6.86 g, 30.2 mmol, 1.10 equiv) in tetrahydrofuran (27.5 mL) was added dropwise at 0 °C over 10 min. Stirring was continued at 0 °C for 10 min, after which the reaction mixture was diluted with diethyl ether (200 mL) and washed in succession with a 2 M aqueous solution of sodium hydroxide (3 × 200 mL) and a saturated

aqueous solution of sodium chloride (2 × 100 mL). The washed organic layer was dried over sodium sulfate, the dried organic layer was filtered, and the filtrate was concentrated under reduced pressure. The residue was purified by flash column chromatography on silica gel (10% ethyl acetate in cyclohexane) to yield 2-(*tert*-butyl)-4-methoxypyridine (**S145**) (600 mg, 3.63 mmol, 13%) as a slightly orange oil. The obtained analytical data for 2-(*tert*-butyl)-4-methoxypyridine (**S145**) were in accordance with reported literature values.[66]

2,6-(*t*-Bu)$_2$-4-MeO-pyridine **B8** was prepared according to a modified literature procedure[65]: To a solution of 2-(*tert*-butyl)-4-methoxypyridine (**S145**) (600 mg, 3.63 mmol, 1 equiv) in dry *n*-hexane (18.2 mL) was added dropwise a solution of *tert*-butyl lithium (1.90 M in *n*-pentane, 2.10 mL, 3.99 mmol, 1.10 equiv) at –40 °C. The resulting orange suspension was allowed to warm up to 5 °C over 5 h, after which the reaction mixture was cooled to –5 °C and a solution of 2,3-dichloro-5,6-dicyano-1,4-benzoquinone (DDQ) (907 mg, 3.99 mmol, 1.10 equiv) in tetrahydrofuran (18.2 mL) was added dropwise at –5 °C over 10 min. Stirring was continued at –5 °C for 10 min, after which the reaction mixture was diluted with diethyl ether (100 mL) and washed in succession with a 2 M aqueous solution of sodium hydroxide (3 × 100 mL) and a saturated aqueous solution of sodium chloride (2 × 100 mL). The washed organic layer was dried over sodium sulfate, the dried organic layer was filtered, and the filtrate was concentrated under reduced pressure. The residue was purified by flash column chromatography on silica gel (15% dichloromethane in *n*-pentane) to yield 2,6-(*t*-Bu)$_2$-4-MeO-pyridine **B8** (251 mg, 1.13 mmol, 31%) as a colorless oil.

Analytical data of 2,6-(*t*-Bu)$_2$-4-MeO-pyridine **B8**:

TLC (25% dichloromethane in *n*-pentane): R_f = 0.27 (UV, CAM).

^{1}H NMR (400 MHz, $CDCl_3$): δ 6.63 (s, 2H), 3.83 (s, 3H), 1.33 (s, 18H).

^{13}C NMR (101 MHz, $CDCl_3$): δ 169.5 (2C), 166.4, 101.6 (2C), 54.9, 37.8 (2C), 30.2 (6C).

IR (ATR, neat): $\tilde{\nu}$ = 2955 (m), 2866 (w), 1589 (s), 1576 (s), 1479 (w), 1456 (m), 1427 (m), 1409 (w), 1358 (w), 1330 (s), 1254 (w), 1229 (m), 1162 (w), 1118 (m), 1062 (s), 1023 (w), 918 (m), 859 (m), 840 (w), 767 (w), 606 (w) cm^{-1}.

HRMS (ESI): calcd for $C_{14}H_{24}NO^+$ $[M+H]^+$: 222.1852; found: 222.1847.

2.5.4 Tetralin **4**

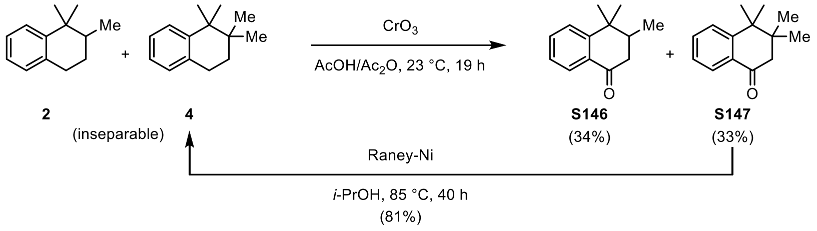

For purification and characterization of tetralin **4** an oxidation/reduction sequence was employed:

To a solution of an inseparable mixture of tetralin **2** (49.2 mg, 282 µmol, 1 equiv) and tetralin **4** (19.3 mg, 102 µmol, 0.363 equiv) in a mixture of acetic acid (2.50 mL) and acetic anhydride (1.25 mL) was added chromium trioxide (88.2 mg, 882 µmol, 3.12 equiv) at 23 °C. After stirring for 19 h at 23 °C, the yellow reaction mixture was diluted with water (5 mL) and extracted with diethyl ether (3 × 5 mL). The combined organic layers were washed in succession with a saturated aqueous solution of sodium chloride (5 mL), a 1 M aqueous solution of sodium hydroxide (2 × 5 mL), and a saturated aqueous solution of sodium chloride (5 mL). The washed organic layer was dried over sodium sulfate, the dried organic layer was filtered, and the filtrate was concentrated under reduced pressure. The residue was purified by flash column chromatography on silica gel (10% to 20% diethyl ether in *n*-pentane) followed by semipreparative normal-phase high performance liquid chromatography (HPLC) (1.0% to 5.0% ethyl acetate in *n*-hexane over 40 min) to afford ketone **S146** (17.8 mg, 94.5 µmol, 34%) as a colorless liquid and ketone **S147** (6.9 mg, 34 µmol, 33%) as a colorless liquid.

<u>Analytical data of ketone **S146**:</u>

TLC (5% ethyl acetate in cyclohexane): R_f = 0.29 (UV).

^{1}H NMR (400 MHz, $CDCl_3$): δ 8.01 (ddd, J = 7.8, 1.6, 0.5 Hz, 1H), 7.53 (ddd, J = 8.0, 7.1, 1.6 Hz, 1H), 7.46 (ddd, J = 8.0, 1.3, 0.5 Hz, 1H), 7.29 (ddd, J = 7.8, 7.1, 1.3 Hz, 1H), 2.76 (dd, J = 17.4, 4.5 Hz, 1H), 2.52 (dd, J = 17.5, 9.5 Hz, 1H), 2.19 (dqd, J = 9.6, 6.9, 4.5 Hz, 1H), 1.42 (s, 3H), 1.27 (s, 3H), 1.04 (d, J = 6.9 Hz, 3H).

^{13}C NMR (101 MHz, $CDCl_3$): δ 198.5, 152.4, 134.1, 131.3, 127.1, 126.3, 126.3, 43.3, 39.0, 37.6, 29.0, 24.9, 16.5.

IR (ATR, neat): $\tilde{\nu}$ = 3065 (w), 2967 (m), 2937 (w), 1682 (s), 1598 (m), 1477 (w), 1448 (m), 1415 (w), 1389 (w), 1377 (w), 1365 (w), 1342 (w), 1299 (m), 1257 (m), 1194 (w), 1157 (w), 1128 (w), 1090 (w), 1054 (w), 1034 (w), 990 (w), 962 (w), 766 (s), 698 (w), 660 (w), 604 (w), 585 (w), 566 (w), 532 (w), 471 (w), 448 (w) cm^{-1}.

HRMS (ESI): calcd for $C_{13}H_{17}O^+$ [M+H]$^+$: 189.1274; found: 189.1270.

The obtained analytical data for ketone **S146** were in accordance with reported literature values.[67]

Analytical data of ketone **S147**:

TLC (5% ethyl acetate in cyclohexane): R_f = 0.30 (UV).

^{1}**H NMR** (400 MHz, $CDCl_3$): δ 8.01 (dd, J = 7.8, 1.6 Hz, 1H), 7.54 (ddd, J = 8.0, 7.1, 1.6 Hz, 1H), 7.46 (dd, J = 8.0, 1.3 Hz, 1H), 7.29 (ddd, J = 8.3, 7.1, 1.3 Hz, 1H), 2.60 (s, 2H), 1.35 (s, 6H), 1.03 (s, 6H).

^{13}**C NMR** (101 MHz, $CDCl_3$): δ 198.5, 152.6, 134.3, 131.2, 126.8, 126.4, 126.2, 50.1, 40.9, 38.3, 24.7 (4C).

IR (ATR, neat): $\tilde{\nu}$ = 3065 (w), 2972 (m), 1684 (s), 1598 (m), 1467 (w), 1449 (m), 1416 (w), 1395 (w), 1380 (w), 1369 (w), 1294 (m), 1268 (w), 1248 (w), 1157 (w), 1113 (w), 1088 (w), 1037 (w), 953 (w), 861 (w), 766 (m), 682 (w), 646 (w), 596 (w), 562 (w), 528 (w), 471 (w) cm^{-1}.

HRMS (ESI): calcd for $C_{14}H_{19}O^+$ [M+H]$^+$: 203.1430; found: 203.1426.

The obtained analytical data for ketone **S147** were in accordance with reported literature values.[67]

Tetralin **4** was prepared according to a modified literature procedure[68]: To a solution of ketone **S147** (6.9 mg, 34 µmol, 1 equiv) in isopropanol (1.00 mL) was added Raney-Nickel (W.R. Grace and Co. Raney® 2800, slurry in water, 203 mg) at 23 °C. The resulting suspension was stirred at 85 °C for 40 h, after which the reaction mixture was allowed to cool to 23 °C and filtered through a silica plug, which was eluted with four column volumes of dichloromethane. The filtrate was concentrated under reduced pressure to afford tetralin **4** (5.2 mg, 28 µmol, 81%) as a colorless liquid.

Analytical data of tetralin **4**:

TLC (*n*-pentane): R_f = 0.74 (UV, CAM).

^{1}**H NMR** (400 MHz, $CDCl_3$): δ 7.36 (dd, *J* = 7.9, 1.3 Hz, 1H), 7.14 (tdt, *J* = 7.8, 1.6, 0.8 Hz, 1H), 7.07 (td, *J* = 7.2, 1.4 Hz, 1H), 7.03 (dd, *J* = 7.5, 1.3 Hz, 1H), 2.81 (t, *J* = 6.9 Hz, 2H), 1.67 (t, *J* = 6.9 Hz, 2H), 1.23 (s, 6H), 0.94 (s, 6H).

^{13}**C NMR** (101 MHz, $CDCl_3$): δ 146.7, 135.1, 128.9, 127.1, 126.0, 125.1, 39.9, 34.6, 33.3, 26.6, 26.5 (2C), 24.6 (2C).

IR (ATR, neat): $\tilde{\nu}$ = 3061 (w), 3014 (w), 2969 (s), 2923 (s), 2873 (m), 1490 (m), 1459 (m), 1448 (m), 1393 (w), 1377 (m), 1364 (m), 1291 (w), 1252 (w), 1222 (w), 1159 (w), 1120 (w), 1094 (w), 1047 (m), 1015 (w), 942 (w), 812 (w), 758 (s), 722 (m), 571 (w), 555 (w), 461 (w), 448 (w) cm^{-1}.

HRMS (ESI): Mass could not be found due to insufficient ionization with electrospray ionization (ESI).

2.5.5 Ketone **S148**

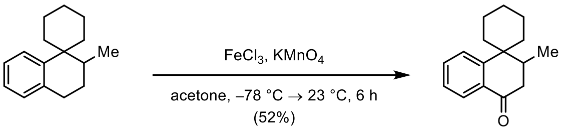

14 **S148**

Ketone **S148** was prepared according to a modified literature procedure[69]: To a solution of spirocycle **14** (7.0 mg, 33 µmol, 1 equiv) in dry acetone (0.44 mL) was added in succession potassium permanganate (52 mg, 0.33 mmol, 10 equiv) and anhydrous iron(III) chloride (13 mg, 82 µmol, 2.5 equiv) at –78 °C under an argon atmosphere. The resulting deep purple suspension was stirred for 1 h 30 min at –78 °C and then allowed to slowly warm up to 23 °C over 4 h 30 min, after which water (1.5 mL) was added to the reaction mixture. The diluted reaction mixture was extracted with diethyl ether (5 × 1.5 mL), the combined organic layers were dried over sodium sulfate, the dried organic layer was filtered, and the filtrate was concentrated under reduced pressure. The residue was purified by flash column chromatography on silica gel (5% diethyl ether in *n*-pentane) to yield ketone **S148** (3.9 mg, 17 µmol, 52%) as a colorless oil.

Analytical data of ketone **S148**:

TLC (10% diethyl ether in *n*-pentane): R_f = 0.51 (UV, CAM).

^{1}H NMR (400 MHz, $CDCl_3$): δ 8.01 (dd, *J* = 7.8, 1.3 Hz, 1H), 7.55 (ddd, *J* = 8.0, 7.1, 1.6 Hz, 1H), 7.48 (dd, *J* = 8.0, 1.3 Hz, 1H), 7.29 (ddd, *J* = 7.8, 7.1, 1.3 Hz, 1H), 3.00 (ddd, *J* = 17.7, 5.4, 0.9 Hz, 1H), 2.83 – 2.74 (m, 1H), 2.42 (ddd, *J* = 17.7, 2.3, 0.9 Hz, 1H), 2.07 – 1.96 (m, 2H), 1.88 – 1.76 (m, 2H), 1.75 – 1.68 (m, 1H), 1.67 – 1.53 (m, 3H), 1.43 – 1.32 (m, 2H), 0.88 (d, *J* = 7.0 Hz, 3H).

^{13}C NMR (101 MHz, $CDCl_3$): δ 198.7, 151.0, 134.3, 131.6, 127.1, 126.2, 125.8, 42.0, 40.3, 39.3, 32.5, 29.9, 26.0, 22.5, 21.0, 15.5.

IR (ATR, neat): $\tilde{\nu}$ = 3064 (w), 2939 (m), 2920 (m), 2866 (m), 1682 (s), 1597 (m), 1477 (w), 1451 (m), 1413 (w), 1380 (w), 1348 (w), 1290 (m), 1278 (w), 1247 (w), 1181 (w), 1142 (w), 1123 (w), 1010 (w), 993 (w), 885 (w), 758 (m), 582 (w), 563 (w), 547 (w) cm^{-1}.

HRMS (ESI): calcd for $C_{16}H_{21}O^+$ [M+H]$^+$: 229.1587; found: 229.1584.

2.5.6 Ketone **S149**

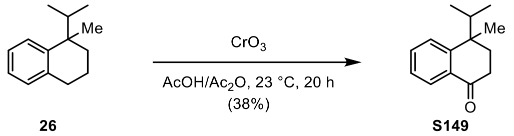

To a solution of tetralin **26** (10.3 mg, 54.5 µmol, 1 equiv) in a mixture of acetic acid (1.33 mL) and acetic anhydride (666 µL) was added chromium trioxide (30.0 mg, 300 µmol, 5.50 equiv) at 23 °C. The resulting yellow solution was stirred at 23 °C for 20 h, after which the reaction mixture was diluted with water (5 mL) and diethyl ether (5 mL). The organic layer was separated and the aqueous layer was extracted with diethyl ether (2 × 5 mL). The combined organic layers were washed in succession with an aqueous solution of sodium hydroxide (2.0 M, 1 × 10 mL) and a saturated aqueous solution of sodium chloride (2 × 10 mL). The washed organic layer was dried over anhydrous sodium sulfate, the dried organic layer was filtered, and the filtrate was concentrated under reduced pressure. Purification via semipreparative normal-phase high performance liquid chromatography (HPLC) (1.0% to 5.0% ethyl acetate in *n*-hexane over 40 min) afforded ketone **S149** (4.2 mg, 21 µmol, 38%) as a colorless oil.

Analytical data of ketone **S149**:

TLC (10% diethyl ether in *n*-pentane): R_f = 0.50 (UV, CAM).

^{1}H NMR (400 MHz, $CDCl_3$): δ 8.01 (dd, *J* = 7.8, 1.5 Hz, 1H), 7.50 (ddd, *J* = 7.9, 7.2, 1.6 Hz, 1H), 7.35 (dd, *J* = 8.0, 1.2 Hz, 1H), 7.29 (ddd, *J* = 8.3, 7.2, 1.2 Hz, 1H), 2.75 (ddd, *J* = 18.1, 9.8, 5.2 Hz, 1H), 2.64 (ddd, *J* = 18.1, 6.9, 5.6 Hz, 1H), 2.15 (ddd, *J* = 13.8, 6.9, 5.2 Hz, 1H), 2.06 (hept, *J* = 6.9 Hz, 1H), 1.91 (ddd, *J* = 13.8, 9.8, 5.6 Hz, 1H), 1.31 (s, 3H), 0.87 (d, *J* = 3.8 Hz, 3H), 0.85 (d, *J* = 3.8 Hz, 3H).

^{13}C NMR (101 MHz, $CDCl_3$): δ 199.0, 152.0, 133.2, 132.1, 127.7, 126.9, 126.3, 39.6, 34.9, 34.4, 31.1, 22.8, 18.1, 17.3.

IR (ATR, neat): $\tilde{\nu}$ = 3064 (w), 2960 (m), 2924 (m), 2873 (w), 1682 (s), 1599 (m), 1459 (m), 1449 (m), 1418 (w), 1390 (w), 1376 (m), 1330 (m), 1286 (m), 1271 (m), 1238 (w), 1196 (w),

1164 (w) 1121 (w), 1101 (w), 1083 (w), 1029 (w), 1017 (w), 983 (w), 962 (w), 925 (w), 904 (w), 856 (w), 793 (w), 760 (s), 736 (w), 681 (w), 659 (w), 581 (w), 567 (m), 551 (m) cm^{-1}.

HRMS (ESI): calcd for $C_{14}H_{18}ONa^+$ $[M+Na]^+$: 225.1250; found: 225.1244.

2.5.7 Standard conditions without methyl iodide

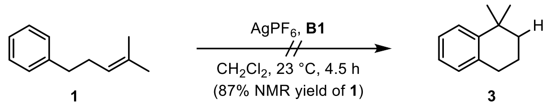

Conclusion: Alkene **1** does not undergo any detectable transformation under standard conditions in the absence of methyl iodide.

Alkene **1** (20.0 mg, 125 µmol, 1 equiv) was subjected to GP1 without the addition of methyl iodide. After stirring for 4.5 h at 23 °C, triethylamine (100 µL, 717 µmol, 5.74 equiv) was added. After stirring for 10 min at 23 °C, the solvent was removed under reduced pressure (40 °C, down to 100 mbar) and the residue was filtered through a silica plug, which was eluted with four column volumes of 10% dichloromethane in *n*-pentane. The filtrate was concentrated under reduced pressure (40 °C, down to 100 mbar). To the residue was added 1,1,2,2-tetrachloroethane as an NMR standard and the yield was determined through quantitative NMR analysis.

2.5.8 Complexation of the silver(I)-ion by **B1**

To investigate complexation between the silver(I)-ion and the base **B1**, three NMR experiments under strict air and moisture exclusion (Argon atmosphere) were conducted:
Sample I consisted of a solution of 2,4,6-tri-*tert*-butylpyrimidine (**B1**) (44.6 mM in dichloromethane).
Sample II consisted of a solution of 2,4,6-tri-*tert*-butylpyrimidine (**B1**) and $AgPF_6$ in the ratio 1:1 (44.6 mM of **B1** and $AgPF_6$ in dichloromethane).
Sample III consisted of a solution of 2,4,6-tri-*tert*-butylpyrimidine (**B1**) and $AgPF_6$ in the ratio 2:1 (89.2 mM of **B1** and 44.6 mM of $AgPF_6$ in dichloromethane).

^1H-NMR, ^{13}C-NMR and ^1H-^{15}N-HSQC were recorded at 25 °C. The ^1H-NMR signal of CH_2Cl_2 was referenced to 5.32 ppm and the ^{13}C-NMR signal of CH_2Cl_2 was referenced to 54.0 ppm. The ^{15}N-NMR was externally referenced to liquid ammonia. Comparison of the NMR data for sample I (only **B1**) with sample II ($AgPF_6$:**B1** stoichiometry of 1:1) indicates quantitative complex formation to either [Ag(**B1**)]$^+$ or to 50% solvated Ag^+ and 50% [Ag(**B1**)$_2$]$^+$. Comparison of the NMR data for sample II ($AgPF_6$:**B1** stoichiometry of 1:1) with sample III ($AgPF_6$:**B1**

stoichiometry of 1:2) indicates different species proving the presence of complex [Ag(**B1**)]$^+$ in sample II, which represents the stoichiometry found in the methylcyclization reaction conditions.

^{1}H-NMR (700 MHz, CH_2Cl_2) δ_H [ppm]

sample I (only **B1**)	sample II ($AgPF_6$:**B1** = 1:1)	sample III ($AgPF_6$:**B1** = 1:2)
1.31 (s, 18H)	1.42 (s, 18H)	1.39 (s, 18H)
1.37 (s, 9H)	1.55 (s, 9H)	1.49 (s, 9H)
7.06 (s, 1H)	7.30 (s, 1H)	7.23 (s, 1H)

^{13}C-NMR (176 MHz, CH_2Cl_2) δ_C [ppm]

sample I (only **B1**)	sample II ($AgPF_6$:**B1** = 1:1)	sample III ($AgPF_6$:**B1** = 1:2)
29.4 (6C)	29.7 (6C)	29.6 (6C)
29.6 (3C)	30.4 (3C)	30.1 (3C)
37.7 (2C)	37.8 (2C)	37.8 (2C)
39.5	39.7	39.6
107.6	110.0	109.3
175.0	175.2	175.2
176.8 (2C)	178.0 (2C)	177.6 (2C)

^{1}H-^{15}N-HSQC (700 MHz, CH_2Cl_2) δ_N [ppm]

sample I (only **B1**)	sample II ($AgPF_6$:**B1** = 1:1)	sample III ($AgPF_6$:**B1** = 1:2)
279.1	266.4	270.6

3. Computational studies

3.1 Computational Methodology

For all minimum structures and transition states conformational sampling using CREST[70] (Version 2.1.12, xTB version 6.4.0) at the GFN2-xTB[71] level with implicit solvation using dichloromethane (ALPB solvation model[72]) was carried out if not noted otherwise. For conformational sampling of transition states, bonds involved in the reaction mode were subjected to constraints. Selected low energy conformers were further refined with wB97XD[73] using the def2-TZVP basis set[74,75] in implicit solvent (dichloromethane) employing the Gaussian 16 package.[76] Core electrons of silver were calculated with effective core potentials to account for scalar relativistic effects. Bulk solvent effects were implicitly modelled by Integral Equation Formalism Polarizable Continuum Model (IEFPCM) of Tomasi and Pascual-Ahuir[77–79] as implemented in Gaussian 16. The internally stored parameters for dichloromethane (ε = 8.9300) were used. Frequency analyses were carried out at the same level as the geometry optimizations and the nature of the stationary points was determined by analyses of the Hessian matrix. (Local) energy minima were confirmed to show only real eigenvalues, whereas transition states were confirmed to have one imaginary eigenvalue, while the corresponding eigenvector coincided with the change in the reaction coordinate. Zero-point energy and thermal corrections were calculated using the standard rigid-rotator/harmonic oscillator model to obtain Gibbs free energies at 298.15 K, no scaling of the frequencies was applied. Structures were visualized using CylView.[80]

3.2 Reaction pathway

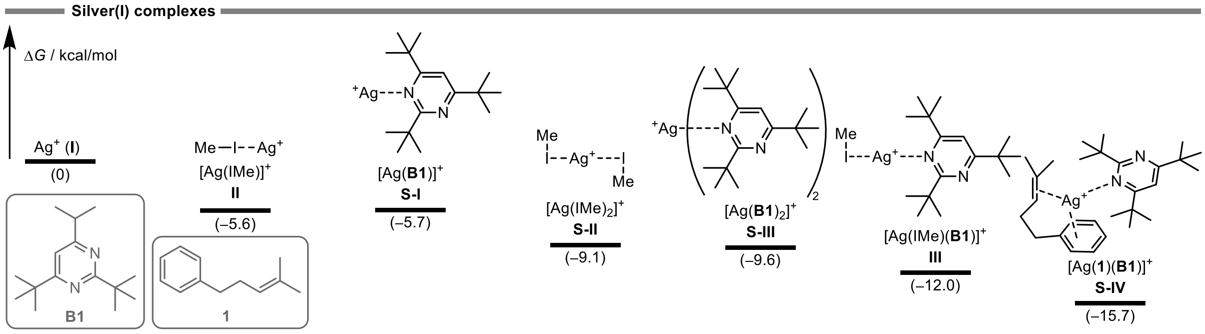

Figure 1. Overview of silver(I) complexes. Complexes **II**, **S-II**, and **III** containing a methyl iodide ligand were considered as reactive methyltransfer species, while **S-IV** (the lowest lying complex) is assumed to be a resting state of the reaction. All calculations were carried out with wB97XD/def2-TZVP in dichloromethane treated as implicit solvent (IEFPCM).

Comparison of various silver(I) complexes with coordination numbers ranging from 1 to 2 at the wB97XD/def2-TZVP (IEFPCM(CH_2Cl_2)) level of theory displayed both methyl iodide and 2,4,6-*tert*-butyl-pyrimidine **B1** as competent ligands (Figure 1). In this context, mixed complex **III** ([Ag(IMe)(**B1**)]$^+$, ΔG = –12.0 kcal/mol) was more stable than silver(I) complexes **II**

($[Ag(IMe)]^+$, ΔG = –5.6 kcal/mol), **S-I** ($[Ag(\mathbf{B1})]^+$, ΔG = –5.7 kcal/mol), **S-II** ($[Ag(IMe)_2]^+$, ΔG = –9.1 kcal/mol), and **S-III** ($[Ag(\mathbf{B1})_2]^+$, ΔG = –9.6 kcal/mol). Of note, coordination of **B1** to silver(I)-ions could be experimentally validated by NMR (see section 2.5.8). While alkene **1** was also an excellent ligand leading to complex **S-IV** ($[Ag(\mathbf{1})(\mathbf{B1})]^+$, ΔG = –15.7 kcal/mol), the lack of a transferable methyl group led us to propose this complex to be a resting state in the reaction pathway.

Silver(I) complexes **II**, **III**, and **S-II** bearing a methyl iodide ligand were investigated with regards to their methyltransfer capability (Figure 2). Assuming fast interconversion (compared to methyltransfer) between the silver(I) complexes **II**, **III**, and **S-II**, the difference in reaction rate is only determine by ΔΔG‡ between the respective transition states (Curtin–Hammett-type scenario).[81,82] Under this assumption, methyltransfer between complex **III** and alkene **1** via **TS-III** (ΔG‡ = 26.6 kcal/mol, Figure 2, blue) should give the highest contribution to the reaction rate. Additionally, **TS-II** (ΔΔG‡ = 0.5 kcal/mol, Figure 2, black) is expected to also contribute with approximately a 0.45-fold slower reaction rate assuming transition state theory ($k_{\text{TS-II}}/k_{\text{TS-III}} = e^{\frac{-\Delta\Delta G^\ddagger}{RT}}$, with R being the molar gas constant and T being the temperature) to the formation of tertiary cation **IV**. Alternative alignment of alkene **1**, which was assumed to stabilize the building positive charge of the tertiary carbocation through the π-system of the arene, resulted in identification of less favorable transition state **S-TS-IIb** (ΔΔG‡ = 5.3 kcal/mol, Figure 2, grey). Of note, **S-TS-IIb** also led to tertiary cation **IV** and no concerted methylation/ring closure was observed. Alternative methylation from complex **S-II** via **S-TS-II** (ΔΔG‡ = 2.2 kcal/mol, Figure S2, red) is predicted to have a negligible contribution to the overall methyltransfer rate.

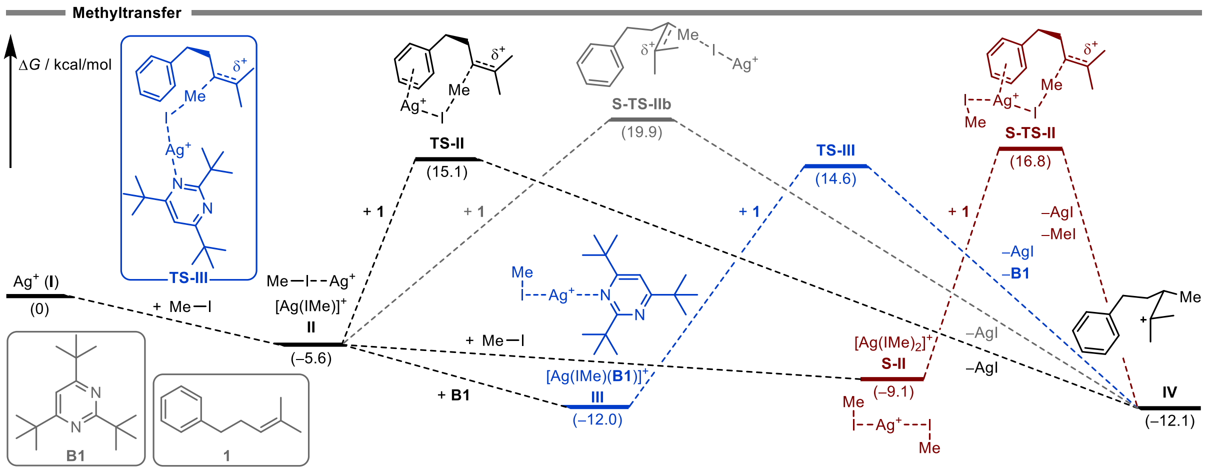

Figure 2. Overview of methyltransfer pathways. **TS-II** and **TS-III** resemble the lowest-lying transition states (assuming comparatively fast interconversion of the silver(I) complexes **II**, **III**, and **S-II**). All calculations were carried out with wB97XD/def2-TZVP in dichloromethane treated as implicit solvent (IEFPCM).

Nucleophilic attack of the arene in **IV** to the tertiary cation leads to slightly favored Wheland intermediate **V** via **TS-IV** (ΔG‡ = 5.8 kcal/mol, Figure 3, black). Various deprotonation

pathways were investigated for **V**: Deprotonation by 2,4,6-*tert*-butyl-pyrimidine **B1** has a high activation barrier (**TS-V**[5], $\Delta G^{\ddagger}$ = 18.1 kcal/mol, Figure 3, black), which can be attributed to unfavorable steric clashes. Alternatively, formation of contact ion pair **VII** (the adduct of Wheland intermediate **V** and PF_6^-, Figure 3, blue), which is lower in energy due to favorable electrostatic interactions, and deprotonation by PF_6^- via **TS-VII**[6] ($\Delta G^{\ddagger}$ = 7.4 kcal/mol) leads to formation of tetralin **2**, PF_5, and HF (**VIII**). Exergonic proton transfer of HF to the terminal base **B1** regenerates PF_6^- leading to tetralin **2** and [H**B1**]$^+$ (**VI**).

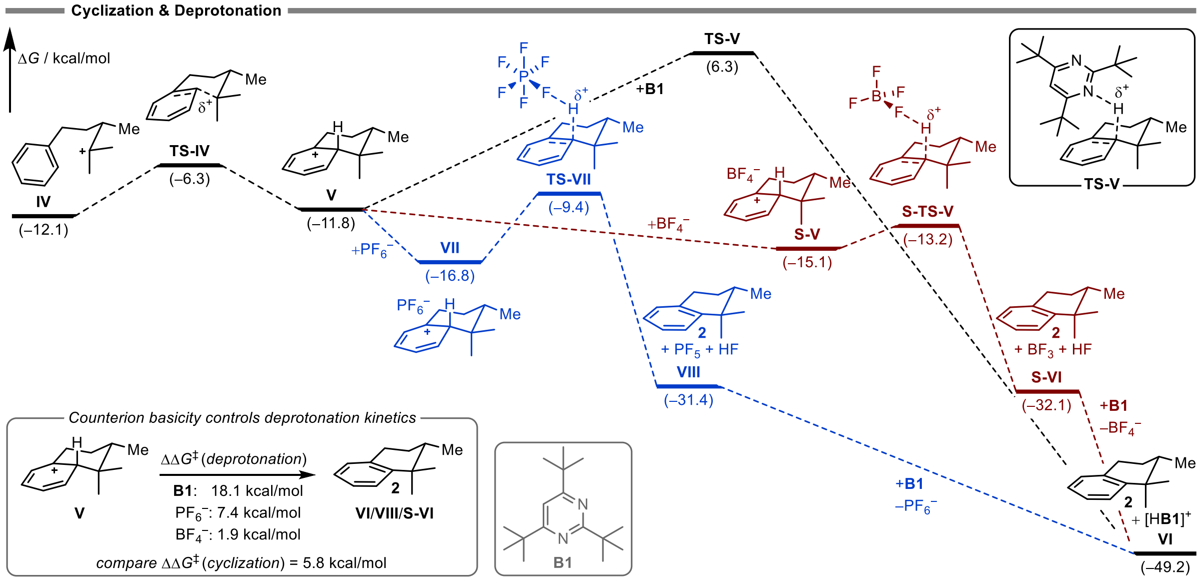

Figure 3. Investigation of cyclization and different deprotonation pathways. Deprotonation is performed by the respective silver(I) counterion (PF_6^- of BF_4^-) and not by the terminal base 2,4,6-*tert*-butyl-pyrimidine **B1**. All calculations were carried out with wB97XD/def2-TZVP in dichloromethane treated as implicit solvent (IEFPCM).

During reaction development, we observed formation of dimethylcyclized side product **4** when silver(I) tetrafluoroborate was used. Formation of **4** can be attributed to premature deprotonation of tertiary cation **IV** followed by a second methylation and subsequent cyclization. To investigate the influence of the silver(I) counterion further, contact ion pair **S-V** (the adduct of Wheland intermediate **V** and BF_4^-) as well as deprotonation with BF_4^- (**S-TS-V**, $\Delta G^{\ddagger}$ = 1.9 kcal/mol) leading initially to tetralin **2**, BF_3, and HF (**S-VI**) were calculated (Figure 3, red). Strikingly, BF_4^- has a significantly lower deprotonation barrier (for Wheland intermediate **V**) compared to PF_6^- and **B1**. While premature deprotonation of tertiary cation **IV** will have different absolute activation barriers, the qualitative deprotonation trend (BF_4^- > PF_6^- > **B1**) should be the same and it is conceivable that a more basic counterion leads to premature deprotonation. Of note, the activation barriers for cyclization (**TS-IV**, $\Delta G^{\ddagger}$ = 5.8 kcal/mol) and

[5] For **TS-V** conformational sampling was unsuccessful. Weak constraints only afforded the educts (**V** and **B1**), whereas higher constraints led to a failure of the meta-dynamics (MTD) simulations in CREST.
[6] For **TS-VII** and **S-TS-V** no conformational sampling was performed, instead the transition states were determined from the lowest-lying contact ion pair conformers **VII** and **S-V**, respectively.

deprotonation with PF_6^- (**TS-VII**, $\Delta G^{\ddagger}$ = 7.4 kcal/mol) are very close to each other. As a consequence, variations in the substrate might lead to preferential deprotonation over cyclization. This would rationalize some of the substrate limitations, for which complex mixtures of (poly)methylated products (both with and without cyclization) have been observed.

3.3 Cartesian Coordinates

Ag^+ (**I**)

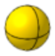

IEFPCM(CH_2Cl_2) wB97xd/def2-TZVP Electronic Energy = –146.878401

IEFPCM(CH_2Cl_2) wB97xd/def2-TZVP Free Energy = –146.895007 (T = 298.15 K)

Number of imaginary frequencies = 0

Ag	1.03254075	0.80725906	0.00000000

MeI

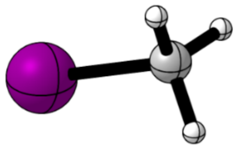

IEFPCM(CH_2Cl_2) wB97xd/def2-TZVP Electronic Energy = –337.702596

IEFPCM(CH_2Cl_2) wB97xd/def2-TZVP Free Energy = –337.690494 (T = 298.15 K)

Number of imaginary frequencies = 0

C	0.00000000	0.00000000	-1.80917400
H	0.00000000	1.03331200	-2.13846100
H	-0.89487400	-0.51665600	-2.13846100
H	0.89487400	-0.51665600	-2.13846100
I	0.00000000	0.00000000	0.32585700

$[Ag(IMe)]^+$ (**II**)

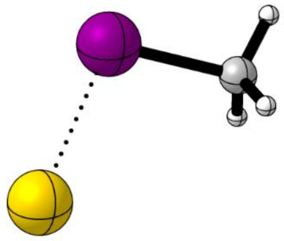

IEFPCM(CH_2Cl_2) wB97xd/def2-TZVP Electronic Energy = –484.599119

IEFPCM(CH_2Cl_2) wB97xd/def2-TZVP Free Energy = –484.594403 (T = 298.15 K)

Number of imaginary frequencies = 0

C	-1.88453100	-1.61020900	0.00000000
H	-2.96306600	-1.49654200	0.00026900

H	-1.53312700	-2.10128600	0.89956300
H	-1.53355900	-2.10113500	-0.89982100
I	-1.09638500	0.37532900	0.00000000
Ag	1.60522000	-0.09643100	0.00000000

2,4,6-*tert*-butyl-pyrimidine (B1)

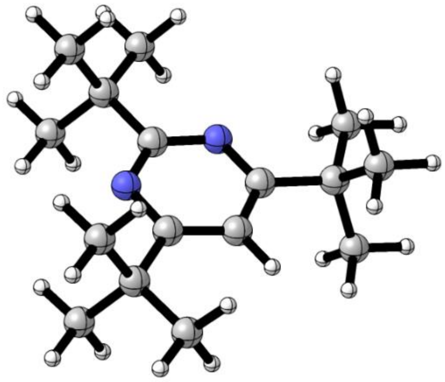

IEFPCM(CH_2Cl_2) wB97xd/def2-TZVP Electronic Energy = –736.152703

IEFPCM(CH_2Cl_2) wB97xd/def2-TZVP Free Energy = –735.787104 (T = 298.15 K)

Number of imaginary frequencies = 0

C	0.25465300	-1.37854000	-0.00019000
C	1.31342500	-0.47743000	-0.00012700
N	1.08552300	0.83190200	-0.00010800
C	-0.18032100	1.24276200	-0.00005700
N	-1.23799600	0.44439500	-0.00000800
C	-1.03362000	-0.87369900	-0.00009600
C	-0.46191200	2.74205500	-0.00001200
C	2.75851400	-0.96504200	-0.00001000
C	0.82917600	3.55761800	-0.00035900
C	2.99681900	-1.81971800	-1.25428700
C	3.73930000	0.20650900	-0.00084300
C	2.99691500	-1.81806000	1.25539000
C	-1.28092000	3.08364400	-1.25240700
C	-1.28027900	3.08368200	1.25279500
C	-2.28443300	-1.74395500	-0.00000800
C	-1.95391700	-3.23622000	-0.00034100
C	-3.10438700	-1.40989200	1.25394300
C	-3.10495700	-1.40947000	-1.25346800
H	0.43772300	-2.44149600	-0.00028800
H	1.43594400	3.34412400	0.88014400
H	1.43542000	3.34421400	-0.88124900
H	0.58318000	4.62181000	-0.00024600
H	2.81598700	-1.23867600	-2.16087400

H	4.03179700	-2.16714300	-1.27314000
H	2.34860600	-2.69705300	-1.27690100
H	3.60734700	0.83661700	0.87890500
H	3.60712400	0.83552500	-0.88134700
H	4.76076300	-0.17965700	-0.00073500
H	2.34855800	-2.69525900	1.27923500
H	4.03185400	-2.16558700	1.27457900
H	2.81626100	-1.23577700	2.16121600
H	-0.72297900	2.84436600	-2.16068000
H	-1.51013700	4.15138800	-1.26631400
H	-2.21775200	2.52664800	-1.26706400
H	-2.21707100	2.52662900	1.26799300
H	-0.72183900	2.84452000	2.16079100
H	-1.50958100	4.15141000	1.26675600
H	-1.38463000	-3.52522600	0.88558200
H	-2.88106800	-3.81170300	-0.00034400
H	-1.38483900	-3.52487000	-0.88651800
H	-4.02486200	-1.99731700	1.26272500
H	-3.36554400	-0.35215700	1.27414300
H	-2.54237300	-1.64348400	2.16107100
H	-4.02541400	-1.99692300	-1.26204500
H	-3.36615300	-0.35173800	-1.27318500
H	-2.54334300	-1.64272500	-2.16093100

[Ag(B1)]+ (S-I)

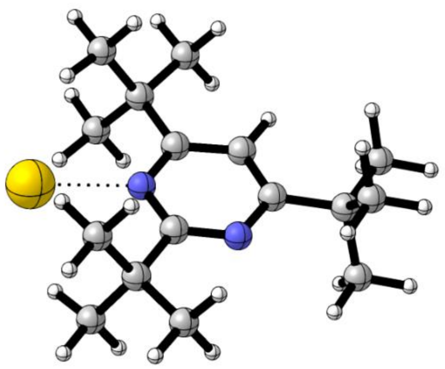

IEFPCM(CH_2Cl_2) wB97xd/def2-TZVP Electronic Energy = –883.056281

IEFPCM(CH_2Cl_2) wB97xd/def2-TZVP Free Energy = –882.691271 (T = 298.15 K)

Number of imaginary frequencies = 0

C	1.53114300	1.33173300	0.00702100
C	2.27675500	0.16695500	-0.00789600
N	1.66304200	-1.01503400	0.00569100
C	0.34744400	-1.05852700	0.02210300
N	-0.43699300	0.03318900	0.01954700

C	0.14947200	1.24136200	0.02174200
C	-0.29602500	-2.44803000	0.05431900
C	3.79567700	0.13129700	-0.03687800
C	0.78036600	-3.53740800	0.07839500
C	4.22945600	-0.61236600	-1.30895000
C	4.40793800	1.53137000	-0.03400100
C	4.28042100	-0.64021900	1.19948200
C	-1.14073800	-2.59926400	1.32744200
C	-1.14255500	-2.66197300	-1.20733700
C	-0.72973800	2.49438100	0.04937700
C	0.10929400	3.77695400	0.08312200
C	-1.59415400	2.55128600	-1.21862200
C	-1.59472400	2.48146100	1.31773100
H	2.01557300	2.29148300	0.00708400
H	1.41366700	-3.49196200	-0.80653500
H	1.42125800	-3.44544300	0.95419100
H	0.29242500	-4.51322800	0.10650300
H	5.31831100	-0.67988200	-1.33962200
H	3.81769400	-1.62113200	-1.32796200
H	3.89576500	-0.08384000	-2.20452500
H	5.49520100	1.44817100	-0.05585600
H	4.13722900	2.09060600	0.86392500
H	4.10326600	2.10944200	-0.90884900
H	5.36979700	-0.70508500	1.18616600
H	3.98066900	-0.13357900	2.11936300
H	3.87157000	-1.65016900	1.21147000
H	-1.94654900	-1.86248400	1.40990200
H	-0.51856200	-2.49199500	2.21715700
H	-1.60302600	-3.58657200	1.34737000
H	-1.95887500	-1.93906800	-1.31269300
H	-0.52808200	-2.58175600	-2.10505300
H	-1.59566200	-3.65338500	-1.18518900
H	-0.56235400	4.63513400	0.11906700
H	0.74870600	3.82214900	0.96570400
H	0.73000700	3.88196800	-0.80769000
H	-0.96920100	2.57816400	-2.11226500
H	-2.21213500	3.44930500	-1.20111100
H	-2.27291400	1.69894100	-1.32636000
H	-0.96921300	2.51701800	2.21086000
H	-2.25615200	3.34795600	1.32335600
H	-2.22168700	1.58966300	1.40288300
Ag	-2.72423900	-0.19237400	-0.06404800

$[Ag(IMe)_2]^+$ (S-II)

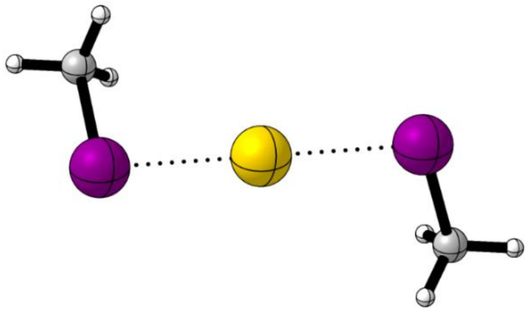

IEFPCM(CH_2Cl_2) wB97xd/def2-TZVP Electronic Energy = –822.323188

IEFPCM(CH_2Cl_2) wB97xd/def2-TZVP Free Energy = –822.290424 (T = 298.15 K)

Number of imaginary frequencies = 0

C	3.21250600	1.38523000	1.08268900
H	2.83153600	2.27033000	0.58756500
H	4.29521600	1.39323300	1.14310900
H	2.75706700	1.23153500	2.05353100
I	2.70096100	-0.29704000	-0.13428000
Ag	-0.00002800	0.00023300	-0.13467200
I	-2.70101300	0.29694600	-0.13415700
C	-3.21207200	-1.38586300	1.08227800
H	-2.83128000	-2.27075300	0.58663500
H	-2.75628400	-1.23257600	2.05302200
H	-4.29476100	-1.39392700	1.14310900

$[Ag(B1)_2]^+$ (S-III)

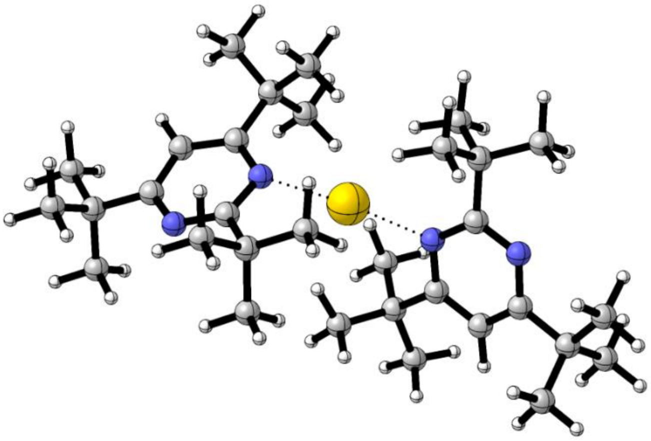

IEFPCM(CH_2Cl_2) wB97xd/def2-TZVP Electronic Energy = –1619.241915

IEFPCM(CH_2Cl_2) wB97xd/def2-TZVP Free Energy = –1618.484510 (T = 298.15 K)

Number of imaginary frequencies = 0

N	4.22568700	0.50470100	0.47958800
N	2.14149100	0.28151500	-0.57963000

N	-2.15668800	0.49208900	0.55010100
N	-3.75891400	-1.19168900	0.87322900
C	3.82153000	-1.33723000	-0.91764000
C	4.61677400	-0.66922500	0.00493500
C	3.03046400	0.96790800	0.15641400
C	2.56620000	-0.84364800	-1.19993700
C	2.69905600	2.38057600	0.65004100
C	5.95963500	-1.22637100	0.44756200
C	4.00059000	3.13598200	0.94769200
C	5.76672400	-2.66120200	0.96057300
C	6.90134400	-1.23376200	-0.76794400
C	6.57185700	-0.37233600	1.55668100
C	1.93452000	3.17103000	-0.41816800
C	1.89092500	2.29956300	1.95232300
C	1.66178200	-1.52139400	-2.23477000
C	2.45460100	-2.52774300	-3.07891300
C	0.53175600	-2.29436300	-1.54248000
C	1.08756500	-0.46560000	-3.18939600
C	-4.27155500	0.64855100	-0.48975600
C	-3.02806100	1.18331900	-0.21677200
C	-2.60318600	-0.62155100	1.15660100
C	-4.58398500	-0.60563300	0.01354700
C	-1.82389700	-1.29000100	2.29269700
C	-2.62211900	2.55807000	-0.75498500
C	-0.59828200	-0.50821000	2.75961100
C	-1.85434400	3.34299400	0.31693800
C	-1.77547000	2.38782500	-2.02430000
C	-3.85946700	3.38528600	-1.12980000
C	-2.79185300	-1.39542600	3.48584100
C	-1.40466200	-2.69821500	1.85373200
C	-5.84935700	-1.36997600	-0.33570200
C	-6.73939700	-0.60207700	-1.31214900
C	-5.42730100	-2.70366300	-0.97074700
C	-6.62818100	-1.63932600	0.95976900
Ag	-0.00736800	0.49415500	-0.03267600
H	4.17819700	-2.23441400	-1.39451300
H	3.75349900	4.15615100	1.24573800
H	4.56419200	2.66489900	1.74994300
H	4.64128200	3.18250300	0.06611200
H	5.07769600	-2.68432500	1.80703800
H	5.38050900	-3.32486200	0.18601800
H	6.72627700	-3.06083800	1.29244900
H	6.51842600	-1.86319700	-1.57279100
H	7.87658600	-1.62331800	-0.47095800
H	7.04145800	-0.22397000	-1.15805700

H	6.74601900	0.65021700	1.22321600
H	5.92182800	-0.33259900	2.43171400
H	7.52656500	-0.80722800	1.85752200
H	2.50723100	3.22795500	-1.34511800
H	1.75976700	4.18600800	-0.05933500
H	0.96075500	2.74582700	-0.65879800
H	2.42630700	1.72463900	2.70921000
H	1.71720200	3.30470600	2.33919200
H	0.91174900	1.83619000	1.81128200
H	2.79234200	-3.38265400	-2.49167400
H	1.80516200	-2.91209100	-3.86568300
H	3.31956100	-2.06409700	-3.55573700
H	0.92688900	-3.00059500	-0.81098800
H	-0.03885300	-2.85164300	-2.28649600
H	-0.17837400	-1.63699200	-1.03476700
H	0.48442100	0.28172300	-2.67632800
H	1.88786200	0.05682600	-3.71596900
H	0.45189700	-0.95327300	-3.92933100
H	-4.97449800	1.18702600	-1.10029000
H	-0.23757600	-0.94125200	3.69329800
H	0.24021900	-0.57711000	2.05975800
H	-0.82581100	0.54254600	2.94143500
H	-2.48485400	3.51901800	1.18995500
H	-0.95560100	2.83381000	0.66032700
H	-1.55093200	4.30877900	-0.08844700
H	-2.32447200	1.83996400	-2.79141800
H	-0.84232500	1.85084100	-1.83878900
H	-1.50604900	3.36731500	-2.42154700
H	-3.53929400	4.39267400	-1.39722700
H	-4.38731500	2.97579000	-1.99140200
H	-4.55774200	3.46421000	-0.29508400
H	-3.10197900	-0.40475300	3.82535800
H	-2.28828900	-1.89643900	4.31384200
H	-3.68042500	-1.96469100	3.22098300
H	-0.90147100	-3.20471100	2.67914800
H	-2.27375000	-3.28711100	1.56196000
H	-0.71308400	-2.65874300	1.01058200
H	-7.08330700	0.34406800	-0.88929400
H	-6.22680900	-0.39663500	-2.25410800
H	-7.62205400	-1.20091900	-1.54003500
H	-6.31483300	-3.29159300	-1.21057300
H	-4.80373500	-3.27935300	-0.28688300
H	-4.86839500	-2.53813300	-1.89451000
H	-6.93218700	-0.70397900	1.43434700
H	-7.52780400	-2.21398100	0.73267200

H	-6.02205200	-2.20468000	1.66683600

[Ag(B1)(IMe)]$^{+}$ (**III**)

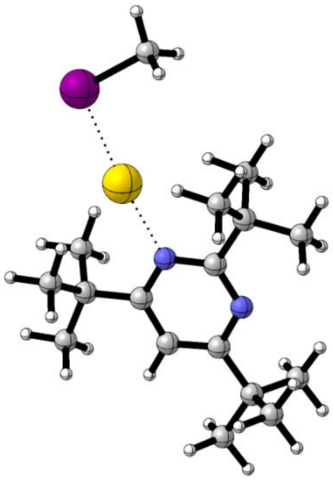

IEFPCM(CH_2Cl_2) wB97xd/def2-TZVP Electronic Energy = –1220.784892

IEFPCM(CH_2Cl_2) wB97xd/def2-TZVP Free Energy = –1220.391726 (T = 298.15 K)

Number of imaginary frequencies = 0

C	-3.04391600	1.16933400	-0.00575900
C	-3.62629400	-0.08444400	0.00062600
N	-2.85874000	-1.17231400	0.00588800
C	-1.54884800	-1.04277900	0.00527600
N	-0.91709800	0.14684400	0.00299000
C	-1.66316500	1.26598900	-0.00362800
C	-0.73570600	-2.34271300	0.00074500
C	-5.12662200	-0.32467200	0.00148600
C	0.10981100	-2.42584700	-1.27883700
C	-5.47997500	-1.12589200	1.26330700
C	-5.92280300	0.97959600	-0.01138500
C	-5.47850300	-1.14930200	-1.24552000
C	0.14104600	-2.42694800	1.25871000
C	-1.67210500	-3.55527400	0.01385500
C	-0.96577300	2.62896700	-0.00926300
C	-0.11136300	2.76690300	-1.27955500
C	-1.97447200	3.78361700	-0.02206500
C	-0.12386000	2.78254900	1.26746100
H	-3.65225000	2.05551200	-0.01210300
H	0.69125000	-3.34872100	-1.27328400
H	-0.53009600	-2.42855400	-2.16253500
H	0.81564200	-1.59873400	-1.39190400
H	-6.55011400	-1.33982300	1.27267200
H	-4.93583100	-2.06970800	1.28799700

H	-5.23769700	-0.56140200	2.16626400
H	-6.98899500	0.74961100	-0.01109400
H	-5.71337900	1.57422400	-0.90295000
H	-5.71650800	1.59017100	0.87003600
H	-4.93522000	-2.09387100	-1.25154800
H	-6.54883200	-1.36233000	-1.25278700
H	-5.23403200	-0.60210400	-2.15847400
H	0.69730900	-3.36517000	1.25237500
H	-0.47347400	-2.39808300	2.15969900
H	0.87443600	-1.62016300	1.33827800
H	-1.06762700	-4.46393700	0.00649800
H	-2.30078500	-3.56491000	0.90335900
H	-2.32493600	-3.56613400	-0.85775300
H	-0.74163600	2.72711300	-2.16915800
H	0.40886800	3.72500600	-1.26831000
H	0.64822500	1.98775900	-1.39106400
H	-1.42644300	4.72617900	-0.02582400
H	-2.61303900	3.77822300	0.86226600
H	-2.60403900	3.76573800	-0.91271200
H	0.40328500	3.73671800	1.24598100
H	-0.76378800	2.76184900	2.15079400
H	0.62822700	1.99936000	1.40003000
Ag	1.33761100	0.26654800	0.01060100
I	4.03877100	0.13826200	0.00695000
C	4.21554400	-1.99158500	-0.05645900
H	3.73735600	-2.38158500	0.83444800
H	3.72603300	-2.32864900	-0.96255100
H	5.27839500	-2.20591000	-0.06937600

Alkene 1

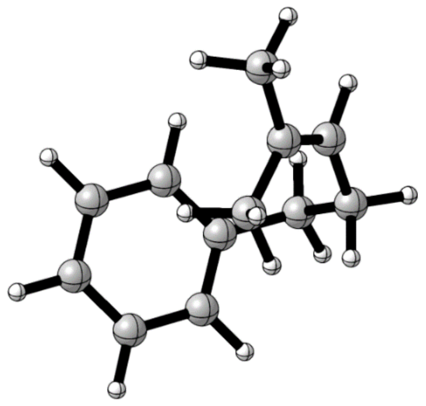

IEFPCM(CH_2Cl_2) wB97xd/def2-TZVP Electronic Energy = –466.921383

IEFPCM(CH_2Cl_2) wB97xd/def2-TZVP Free Energy = –466.711717 (T = 298.15 K)

Number of imaginary frequencies = 0

C	2.64741700	-1.29620200	0.01573400
C	2.66194200	-0.23668900	0.91233700
C	1.82971400	0.85396500	0.71209400
C	0.97001000	0.90973100	-0.38174800
C	0.97304800	-0.15400300	-1.27856700
C	1.80199700	-1.24929400	-1.08345200
C	-2.09943900	-0.54502300	0.26184700
C	-2.10432300	0.70835500	-0.18944500
C	-1.27418600	1.85832400	0.29670500
C	0.01000200	2.05708900	-0.53676000
C	-2.94017400	-1.60653700	-0.38876100
C	-1.25140800	-1.03741000	1.39837600
H	3.29438200	-2.15056000	0.17040700
H	3.32283400	-0.26059700	1.77007600
H	1.84023200	1.67460800	1.42134700
H	0.30833300	-0.12802000	-2.13399000
H	1.78623300	-2.06924800	-1.79100300
H	-2.73498900	0.92845100	-1.04822500
H	-1.86263300	2.77729300	0.23750500
H	-0.99374000	1.73354900	1.34430700
H	-0.26132500	2.17588000	-1.58849300
H	0.49104600	2.98553500	-0.22054400
H	-3.52045400	-1.21204600	-1.22314100
H	-2.30756100	-2.41801900	-0.76175100
H	-3.63025300	-2.05370800	0.33270600
H	-0.76878500	-0.23565000	1.95289800
H	-1.84975900	-1.63141600	2.09437500
H	-0.46115900	-1.68988600	1.01336200

[Ag(1)(B1)]$^+$ (S-IV)

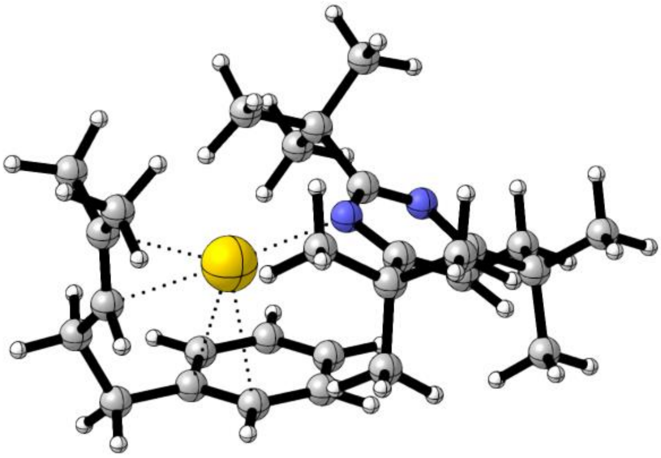

IEFPCM(CH_2Cl_2) wB97xd/def2-TZVP Electronic Energy = –1350.021688

IEFPCM(CH_2Cl_2) wB97xd/def2-TZVP Free Energy = –1349.418900 (T = 298.15 K)

Number of imaginary frequencies = 0

C	-0.88804800	-2.31063900	-1.43713800
C	0.00829700	-3.22374500	-0.89741800
C	1.36936100	-3.07016400	-1.10004800
C	1.86576100	-2.00895700	-1.85807000
C	0.95467800	-1.09651200	-2.39305900
C	-0.41450000	-1.24712300	-2.18508700
C	3.88386000	0.71060400	0.66613300
C	3.79423800	0.11052200	-0.55173400
C	4.10226800	-1.32763300	-0.86777200
C	3.34661600	-1.86500700	-2.08887700
C	3.92696100	2.20921800	0.79357200
C	4.20026600	-0.03123200	1.93284500
H	-1.94988500	-2.43442900	-1.27180900
H	-0.35820800	-4.06056100	-0.31696300
H	2.06009200	-3.79304400	-0.68125000
H	1.31680200	-0.28896400	-3.01957000
H	-1.10415400	-0.53369300	-2.61783500
H	3.75097600	0.76962500	-1.41820000
H	5.17554000	-1.38971600	-1.07384800
H	3.91738400	-1.97575200	-0.01037300
H	3.52419800	-1.20626500	-2.94162200
H	3.76367000	-2.83936200	-2.34839900
H	3.74057500	2.71022600	-0.15525500
H	3.20753300	2.56601400	1.53288100
H	4.91947700	2.50123500	1.14703000
H	3.93429200	-1.08530900	1.89456000
H	5.27618400	0.03868700	2.11607100
H	3.69800100	0.42672200	2.78507000
Ag	1.51027200	0.30683800	0.02504000
N	-0.61832000	0.86376500	0.67637900
C	-1.25463500	-0.10253000	1.35486200
C	-1.32900800	1.56163200	-0.23234800
N	-2.46833900	-0.53874000	1.05930000
C	-0.64551800	-0.69778500	2.62368000
C	-2.60352300	1.15110000	-0.56474300
C	-0.75063100	2.82846200	-0.86552300
C	-3.13221800	0.03161300	0.06268900
C	0.78141900	-0.23894500	2.90440400
C	-1.54622400	-0.22172600	3.77911200
C	-0.67022100	-2.22733300	2.54755300
H	-3.17427700	1.68677300	-1.30732200

C	0.58148300	3.25435200	-0.25074800
C	-0.56398100	2.61447300	-2.37424900
C	-1.75680300	3.97047400	-0.62984600
C	-4.49421200	-0.52717400	-0.31897400
H	0.88967900	0.84597100	2.87146800
H	1.07713000	-0.57822800	3.89812500
H	1.49330400	-0.69508900	2.20781000
H	-2.57381500	-0.55120600	3.63030100
H	-1.53752500	0.86759600	3.85875200
H	-1.18023700	-0.63543400	4.72014100
H	-0.30486100	-2.64626700	3.48689400
H	-1.67950400	-2.59467800	2.36896400
H	-0.02522400	-2.58187900	1.74160800
H	1.40333000	2.59823000	-0.54953000
H	0.84109500	4.24882600	-0.61501500
H	0.54196400	3.28456800	0.83797100
H	-0.20998300	3.53841000	-2.83445300
H	-1.49753200	2.33076700	-2.86169000
H	0.17689100	1.83755500	-2.57163000
H	-2.71936000	3.77781800	-1.10210400
H	-1.92392300	4.12823800	0.43709600
H	-1.35937700	4.89378900	-1.05358900
C	-4.74440300	-1.87486400	0.35679300
C	-5.56224000	0.47859300	0.14221400
C	-4.57744200	-0.70269200	-1.84209200
H	-5.71867300	-2.25618300	0.04665100
H	-3.98592700	-2.60842400	0.07837400
H	-4.73620600	-1.78498900	1.44217700
H	-5.43662800	1.44860700	-0.34183800
H	-5.51597700	0.62558400	1.22283300
H	-6.55474700	0.10049500	-0.10941400
H	-5.55358300	-1.11151100	-2.10762100
H	-4.45962300	0.24163400	-2.37446600
H	-3.81466200	-1.39484800	-2.20437300

TS-II

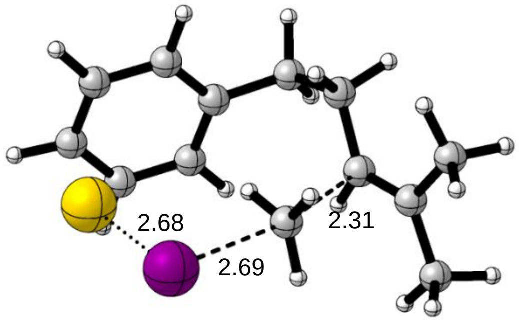

IEFPCM(CH_2Cl_2) wB97xd/def2-TZVP Electronic Energy = –951.512421

IEFPCM(CH_2Cl_2) wB97xd/def2-TZVP Free Energy = –951.273175 (T = 298.15 K)

Number of imaginary frequencies = 1 (508.83i cm^{-1})

C	2.01207400	2.54714800	0.37132200
C	1.09640400	2.55405100	1.43035700
C	-0.26389400	2.62563100	1.17411200
C	-0.74967500	2.68070800	-0.13235300
C	0.16146300	2.63182400	-1.18379900
C	1.52941500	2.57243000	-0.94494600
C	-3.82539100	-0.60462500	0.26372800
C	-3.09884600	0.53755100	0.31736100
C	-2.91807100	1.50599700	-0.82271600
C	-2.22408000	2.81237500	-0.41764700
C	-4.14186400	-1.38693800	1.49825300
C	-4.44189200	-1.10647300	-1.00258300
H	3.07512500	2.63442200	0.56736400
H	1.45632800	2.54652800	2.45059000
H	-0.95812300	2.66773000	2.00439600
H	-0.19698400	2.67504100	-2.20505400
H	2.22441400	2.60844700	-1.77402600
H	-2.77514600	0.87295600	1.29884200
H	-2.37153700	1.04253400	-1.64990200
H	-3.90378700	1.75577700	-1.22228500
H	-2.35596200	3.52820900	-1.23032700
H	-2.72954800	3.23021000	0.45511300
H	-5.22279000	-1.37831100	1.65851500
H	-3.85162900	-2.43387100	1.38251700
H	-3.65821100	-0.97557000	2.38323000
H	-4.31267200	-2.18653300	-1.09259200
H	-5.51872000	-0.91933600	-0.96809200
H	-4.04321500	-0.62462400	-1.89269600
C	-1.40046400	-1.01000300	0.09476300
H	-1.75028200	-1.49504700	-0.80034100
H	-0.88981500	-0.06864200	0.01584000
H	-1.52892700	-1.46836200	1.05854700

I	0.97504900	-2.24700500	-0.13825800
Ag	2.06857100	0.18982300	0.06410500

S-TS-IIb

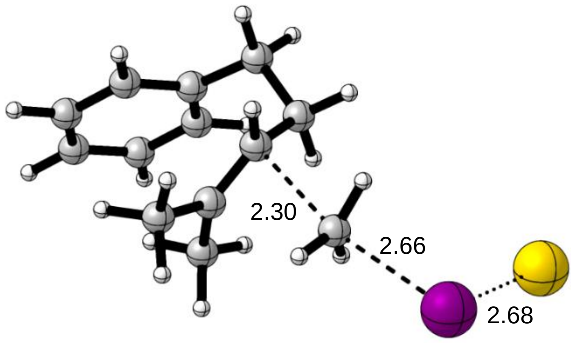

IEFPCM(CH_2Cl_2) wB97xd/def2-TZVP Electronic Energy = –951.499034

IEFPCM(CH_2Cl_2) wB97xd/def2-TZVP Free Energy = –951.265505 (T = 298.15 K)

Number of imaginary frequencies = 1 (516.38i cm^{-1})

C	6.18756500	-0.30723300	-1.22276200
C	5.42650900	-1.45570100	-1.39348400
C	4.47477500	-1.80519600	-0.44834400
C	4.26551100	-1.01784300	0.68056400
C	5.04438000	0.12285100	0.84794100
C	5.99715700	0.47904700	-0.09582600
C	2.07545800	1.43720700	0.03242100
C	1.75666500	0.65627100	1.09445100
C	1.79070500	-0.84072600	1.15720800
C	3.16165000	-1.35155600	1.64697000
C	2.24965400	2.91309000	0.19904800
C	2.34392400	0.91645200	-1.34132900
H	6.92960600	-0.03094800	-1.96094300
H	5.57513700	-2.08008100	-2.26560900
H	3.87819300	-2.69950700	-0.59203600
H	4.89686800	0.74488900	1.72335900
H	6.59163700	1.37234400	0.04962500
H	1.67763300	1.15457600	2.05686100
H	1.02309800	-1.19021600	1.85099300
H	1.57435500	-1.28833400	0.18594500
H	3.37677500	-0.91933900	2.62641400
H	3.09327500	-2.43274200	1.77965500
H	1.99160100	3.24957400	1.20201900
H	3.29514900	3.16788200	0.00522600

H	1.65382300	3.46425400	-0.53220800
H	2.07270400	-0.12820700	-1.47065100
H	1.82001900	1.51977900	-2.08541000
H	3.41483000	1.00727400	-1.54599100
C	-0.38215300	1.05540600	0.35986700
H	-0.33250500	2.11915400	0.20760700
H	-0.25945000	0.37943100	-0.46675600
H	-0.57680000	0.67426800	1.34648300
I	-2.99844100	1.11589800	-0.11433200
Ag	-3.38573200	-1.53274600	-0.10968800

TS-III

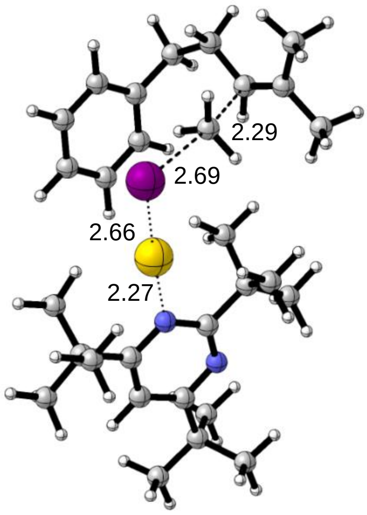

IEFPCM(CH_2Cl_2) wB97xd/def2-TZVP Electronic Energy = –1687.693298

IEFPCM(CH_2Cl_2) wB97xd/def2-TZVP Free Energy = –1687.061036 (T = 298.15 K)

Number of imaginary frequencies = 1 (500.79i cm^{-1})

C	1.54437700	-1.58995500	2.76877400
C	2.90925100	-1.84266100	2.72626000
C	3.81093100	-0.79214900	2.74310300
C	3.37080600	0.53001400	2.80295300
C	2.00168200	0.76933700	2.84346700
C	1.09263900	-0.28182200	2.82466400
C	4.31131700	2.69278900	-0.91270200
C	4.01500900	2.37341000	0.37068200
C	4.99810000	1.89421400	1.40516900
C	4.36634700	1.66144900	2.78264100
C	3.31814500	3.38291100	-1.79289700
C	5.66275200	2.46936000	-1.50999800
H	0.83991200	-2.41135400	2.75965800
H	3.27028200	-2.86213300	2.68024600

H	4.87508300	-0.99770400	2.71140800
H	1.63971600	1.79002900	2.89309500
H	0.02965700	-0.07496100	2.85292300
H	3.03260500	2.65816300	0.73704600
H	5.49635400	0.97894400	1.07356000
H	5.78711100	2.64444600	1.50491800
H	5.16866500	1.45015200	3.49182500
H	3.88397800	2.58289300	3.11555000
H	3.69793900	4.37412900	-2.05181400
H	3.18612500	2.84381400	-2.73399900
H	2.35002000	3.50263700	-1.30872000
H	6.30986900	1.85861000	-0.88465300
H	5.57533600	2.00530500	-2.49470600
H	6.14779100	3.43760400	-1.65973500
C	3.31981100	0.46764500	-0.70146200
H	2.99449900	0.26783400	0.30551300
H	2.63601500	0.90652800	-1.40543900
H	4.30407100	0.16033900	-1.01059400
I	2.34799700	-1.94226500	-1.39481300
Ag	-0.04920500	-1.02282500	-0.69587000
N	-2.10622500	-0.17824400	-0.22543300
C	-2.36215900	1.13998700	-0.34370800
C	-3.12115700	-0.99031500	0.12604300
N	-3.55029200	1.67578300	-0.15832700
C	-1.23371900	2.11585500	-0.69809600
C	-4.38272800	-0.46042700	0.34012800
C	-2.87152200	-2.49389100	0.27939900
C	-4.57606500	0.89776600	0.17887800
C	-0.13065200	2.03304400	0.36426000
C	-0.68659300	1.81534800	-2.10040300
C	-1.75666600	3.55582600	-0.70676400
H	-5.20000100	-1.09763100	0.62476200
C	-4.12615400	-3.23227300	0.76126200
C	-2.48864600	-3.09002600	-1.08403300
C	-1.77677700	-2.73821100	1.32741000
C	-5.91273500	1.59544100	0.36697400
H	-0.53205900	2.24548200	1.35680500
H	0.35521700	1.05703100	0.41424800
H	0.63971000	2.77444700	0.14789900
H	-0.27841600	0.80730100	-2.20268300
H	0.11703100	2.51478600	-2.33667400
H	-1.46989200	1.92941500	-2.85123400
H	-0.93156400	4.22485200	-0.95863900
H	-2.54822300	3.69050300	-1.44225200
H	-2.15146000	3.84416900	0.26682600

H	-4.46344300	-2.87245500	1.73461000
H	-4.94922600	-3.15110500	0.05013900
H	-3.88795300	-4.29109000	0.86553800
H	-1.59444400	-2.63731100	-1.51845000
H	-3.30186700	-2.96198900	-1.80026500
H	-2.29077500	-4.15673500	-0.97360200
H	-1.61381500	-3.81009900	1.44332100
H	-0.81523800	-2.29684900	1.05797300
H	-2.06716200	-2.32551600	2.29511500
C	-7.02412900	0.62251600	0.75862300
C	-5.74908200	2.65240300	1.46916600
C	-6.27987100	2.28457900	-0.95559800
H	-6.81041600	0.12299100	1.70576800
H	-7.95788400	1.17310200	0.87966700
H	-7.18625300	-0.13871800	-0.00717200
H	-4.97235600	3.36983400	1.20574900
H	-5.48367400	2.18706500	2.42096600
H	-6.68951500	3.18969100	1.60366500
H	-6.39930400	1.55333400	-1.75798000
H	-7.22373700	2.82017600	-0.83979900
H	-5.50900100	2.99707900	-1.24828500

S-TS-II

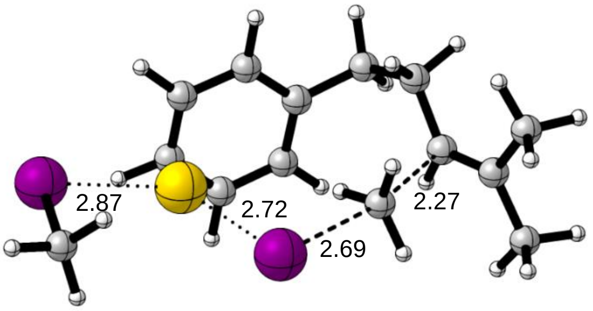

IEFPCM(CH_2Cl_2) wB97xd/def2-TZVP Electronic Energy = –1289.228028

IEFPCM(CH_2Cl_2) wB97xd/def2-TZVP Free Energy = –1288.960970 (T = 298.15 K)

Number of imaginary frequencies = 1 (514.33i cm^{-1})

C	-0.47214900	2.60497500	-1.02016700
C	0.64142700	2.47006300	-1.84755700
C	1.91576000	2.50279600	-1.30715100
C	2.11488800	2.63810200	0.06773100
C	0.99875400	2.72314100	0.89266900
C	-0.28752900	2.72752300	0.35948500
C	5.07722200	-0.74733100	0.18703400
C	4.43140100	0.43513000	0.03638000

C	4.06359300	1.38027400	1.15037000
C	3.50042300	2.71789300	0.65641400
C	5.60824400	-1.49599500	-0.99320700
C	5.37463900	-1.34339500	1.52409700
H	-1.46188000	2.69576200	-1.45096200
H	0.50925300	2.38128200	-2.91769000
H	2.77283600	2.44907100	-1.96794600
H	1.13051600	2.83788700	1.96191100
H	-1.13644400	2.90247500	1.00944400
H	4.35049500	0.82262400	-0.97473900
H	3.35146700	0.91953800	1.84129100
H	4.96117900	1.58550000	1.73891700
H	3.48146500	3.40761100	1.50118200
H	4.18587300	3.14179500	-0.08057500
H	6.69969600	-1.51103000	-0.94265600
H	5.28046700	-2.53799600	-0.97597500
H	5.31236100	-1.04038400	-1.93707700
H	4.89620500	-0.81542500	2.34582600
H	5.07221700	-2.39251700	1.54948900
H	6.45544800	-1.32497800	1.68738000
C	2.65847200	-0.98072500	-0.10820500
H	2.18988400	-0.05036400	-0.37961200
H	3.00044600	-1.64167500	-0.88428800
H	2.70703400	-1.27143400	0.92590700
I	0.24334300	-2.14852400	-0.23487900
Ag	-1.02792400	0.25038300	-0.11096200
I	-3.87395100	0.28774900	0.29241100
C	-4.07577900	-1.83856300	0.28148500
H	-5.11526800	-2.05729600	0.49900900
H	-3.79079800	-2.18306200	-0.70587300
H	-3.41400800	-2.22765200	1.04676100

AgI

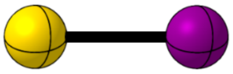

IEFPCM(CH_2Cl_2) wB97xd/def2-TZVP Electronic Energy = –444.895534

IEFPCM(CH_2Cl_2) wB97xd/def2-TZVP Free Energy = –444.921503 (T = 298.15 K)

Number of imaginary frequencies = 0

I	0.00000000	0.00000000	1.24097700
Ag	0.00000000	0.00000000	-1.39939900

Tertiary cation (**IV**)

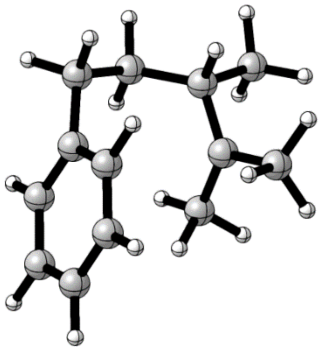

IEFPCM(CH_2Cl_2) wB97xd/def2-TZVP Electronic Energy = –506.643647

IEFPCM(CH_2Cl_2) wB97xd/def2-TZVP Free Energy = –506.395027 (T = 298.15 K)

Number of imaginary frequencies = 0

C	2.26384800	-0.96690000	-0.95789100
C	2.95867600	-0.84587900	0.23460000
C	-1.38097100	-2.15471600	-0.64903200
C	2.69851000	0.23097400	1.07653700
C	1.29585800	-0.02892100	-1.29542500
C	-0.65947200	-1.03971700	1.47640700
C	1.73241600	1.16104700	0.73656000
C	-1.29660500	-0.94042200	0.16808900
C	1.00410000	1.03424800	-0.44574900
C	-1.96968300	0.26706700	-0.30203000
C	-0.12465500	1.97800900	-0.74926100
C	-1.39082800	1.63636400	0.04487400
C	-3.37102700	0.07616500	0.38970000
H	2.47480100	-1.78967200	-1.62917700
H	3.71135600	-1.57573200	0.50285100
H	-0.48243800	-2.13465100	-1.28039900
H	-1.34070900	-3.06656300	-0.05771100
H	-2.23517800	-2.14001500	-1.32352200
H	3.25113600	0.34019100	2.00088600
H	0.76158200	-0.12119700	-2.23486800
H	-1.14685500	-1.84137400	2.03995500
H	0.36787600	-1.39532200	1.31398200
H	-0.64075200	-0.11708900	2.04423200
H	1.52691600	1.99176000	1.40197300
H	-2.13506600	0.18908600	-1.37792500
H	0.16397200	3.00081500	-0.50187200
H	-0.35320900	1.95687000	-1.81670000
H	-2.16180400	2.37428000	-0.18096600
H	-1.19715200	1.71447600	1.11645600

H	-4.01528300	0.84746000	-0.02826400
H	-3.81621300	-0.89498600	0.18055500
H	-3.29398600	0.21774500	1.46625100

TS-IV

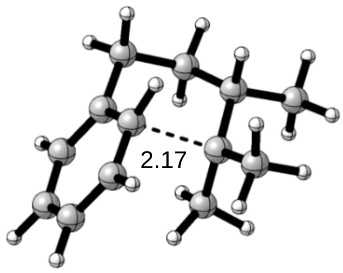

IEFPCM(CH_2Cl_2) wB97xd/def2-TZVP Electronic Energy = –506.639649

IEFPCM(CH_2Cl_2) wB97xd/def2-TZVP Free Energy = –506.385810 (T = 298.15 K)

Number of imaginary frequencies = 1 (249.86i cm^{-1})

C	2.76052000	0.34876500	0.79846300
C	2.78066500	-0.86345600	0.09352400
C	-0.39481200	-0.88990100	1.51198400
C	1.82808100	-1.10156300	-0.85972900
C	1.81089200	1.31653200	0.53532300
C	-1.02261200	-2.18489600	-0.54741500
C	0.80693700	-0.15341200	-1.09020200
C	-0.86935100	-0.84562600	0.10322300
C	0.83916600	1.09586200	-0.43413200
C	-1.89606200	0.19244600	-0.27921100
C	-0.24482800	2.07667600	-0.71365500
C	-1.51909700	1.63829300	0.02285200
C	-3.20288600	-0.15761000	0.46961000
H	3.51302000	0.53381000	1.55446300
H	3.55331400	-1.59219900	0.29579600
H	-0.40559600	0.07440900	2.00972100
H	-1.07023100	-1.56834900	2.04334800
H	0.59899800	-1.32978000	1.58877600
H	1.83855000	-2.01963000	-1.43157700
H	1.82459600	2.25351100	1.07687000
H	-0.20893300	-2.85899000	-0.29367800
H	-1.94196100	-2.61926400	-0.14208400
H	-1.13892300	-2.12029300	-1.62691600
H	0.20689600	-0.24979800	-1.98587000
H	-2.09219300	0.09185700	-1.34912200
H	0.04225900	3.07394100	-0.38244500
H	-0.44279500	2.11924400	-1.78586700

H	-2.34581100	2.28210400	-0.27743400
H	-1.38997000	1.77876400	1.09817000
H	-3.54054900	-1.17053200	0.25826300
H	-3.08123500	-0.04237000	1.54667900
H	-3.97911800	0.53090100	0.13911000

Wheland intermediate (V)

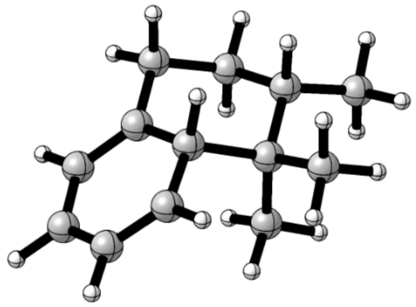

IEFPCM(CH_2Cl_2) wB97xd/def2-TZVP Electronic Energy = –506.648083

IEFPCM(CH_2Cl_2) wB97xd/def2-TZVP Free Energy = –506.394527 (T = 298.15 K)

Number of imaginary frequencies = 0

C	3.02294300	0.28935800	0.48525400
C	2.80013000	-1.02041300	0.02024300
C	-0.55737600	-0.92905400	1.51240100
C	1.61961800	-1.30834500	-0.57558700
C	2.10062000	1.31251900	0.31070500
C	-1.23912300	-2.11762400	-0.57937300
C	0.55902500	-0.31014600	-0.72568200
C	-0.81676300	-0.77410800	0.01321600
C	0.90656000	1.05795100	-0.31895100
C	-1.88215400	0.30955900	-0.30387500
C	-0.10387300	2.11137200	-0.56279900
C	-1.43687700	1.71541400	0.08515800
H	0.25723300	-0.29286800	-1.78223500
C	-3.24056200	0.02608600	0.33938000
H	3.95976100	0.51277300	0.98194800
H	3.56674000	-1.77198200	0.14041100
H	-0.26196900	0.00582000	1.98863800
H	-1.46116100	-1.28542900	2.00567400
H	0.22466500	-1.66490400	1.70026900
H	1.43243300	-2.30568400	-0.94875800
H	2.33318200	2.31127900	0.65401200
H	-0.52666300	-2.90898400	-0.35220900
H	-2.18936200	-2.42475600	-0.14576500
H	-1.36433000	-2.05832700	-1.66177700
H	-2.02210500	0.29275200	-1.39092100

H	0.24359800	3.07472300	-0.19392300
H	-0.25151300	2.19794700	-1.64370900
H	-1.35292900	1.79594300	1.17153800
H	-2.19598200	2.43458000	-0.22248900
H	-3.95545100	0.78209900	0.01305500
H	-3.64302500	-0.94527100	0.05781700
H	-3.18613200	0.07488200	1.42788800

TS-V

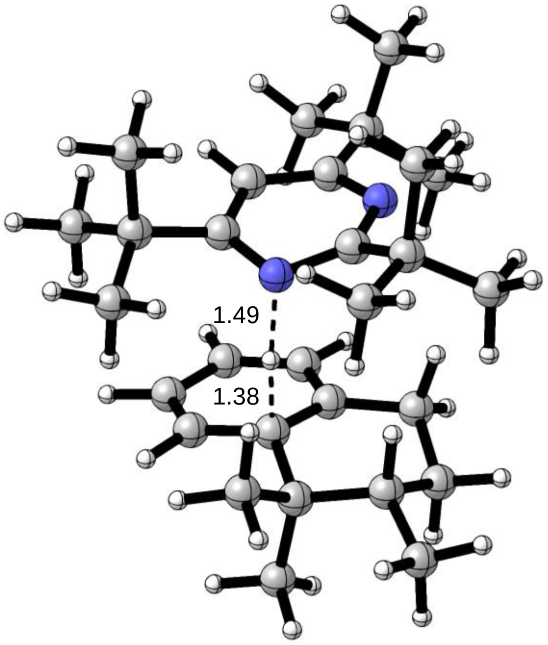

IEFPCM(CH_2Cl_2) wB97xd/def2-TZVP Electronic Energy = –1242.795741

IEFPCM(CH_2Cl_2) wB97xd/def2-TZVP Free Energy = –1242.152736 (T = 298.15 K)

Number of imaginary frequencies = 1 (1395.32i cm^{-1})

C	0.15538600	1.90513100	-2.53340100
C	-0.60917100	2.76184500	-1.74144900
C	-4.25390500	0.95901200	-1.27528300
C	-1.57457600	2.23497500	-0.93161800
C	-0.08248000	0.54598100	-2.53131500
C	-3.44855200	1.00412200	1.08541600
C	-1.80658000	0.82762800	-0.82380000
C	-3.20605000	0.39606300	-0.30096400
C	-1.08193700	-0.00648700	-1.74291500
C	-3.28261300	-1.14996500	-0.23015600
C	-1.36020700	-1.46901700	-1.83154500
C	-2.80463800	-1.81716300	-1.51624600
H	-0.91543400	0.58265600	0.20017400

C	-4.65385100	-1.69759400	0.15431800
H	0.92436500	2.31250200	-3.17736600
H	-0.46160000	3.83111400	-1.79377200
H	-4.19708900	2.04727100	-1.31878400
H	-4.11613700	0.57860100	-2.28804300
H	-5.25912600	0.69868800	-0.94427200
H	-2.19263700	2.90427300	-0.35158400
H	0.48131000	-0.09818000	-3.19251600
H	-2.69002200	0.69263500	1.80357200
H	-3.44899900	2.09370500	1.05485600
H	-4.42332900	0.70723200	1.46788000
H	-2.58667100	-1.44805100	0.55503200
H	-1.06882000	-1.82393600	-2.82076200
H	-0.69285200	-1.98070700	-1.13169500
H	-3.44972400	-1.53310900	-2.35106700
H	-2.89528500	-2.90022100	-1.41455400
H	-5.38347500	-1.54206600	-0.64215000
H	-4.57768200	-2.77299600	0.32211700
H	-5.04569900	-1.25189800	1.06761500
N	0.25959300	0.24602500	1.04572000
C	0.63965200	-1.04746600	0.93756600
C	1.22213200	1.17064400	0.80738000
N	1.66632900	-1.42937800	0.21132600
C	0.03069900	-2.09692500	1.86754600
C	2.34851100	0.81080200	0.09229500
C	1.13646300	2.49615000	1.55748900
C	2.46683500	-0.50869700	-0.32561400
C	1.22372600	-2.52438000	2.75452100
C	-1.03913400	-1.52319300	2.79347000
C	-0.47916500	-3.33432400	1.12417900
H	3.11088100	1.53819900	-0.13366500
C	2.06058900	2.25897900	2.77769800
C	1.67318200	3.68538900	0.76034000
C	-0.25945600	2.80699300	2.09432900
C	3.55124000	-1.00723900	-1.26162500
H	1.99817600	-3.00988500	2.16275700
H	1.66153900	-1.66269900	3.26308800
H	0.86818400	-3.22329600	3.51287300
H	-0.65349300	-0.67936500	3.36651600
H	-1.35078600	-2.29880600	3.49431700
H	-1.92645000	-1.18873400	2.26438500
H	-1.41126500	-3.14425200	0.59293700
H	0.26463700	-3.69127800	0.41145000
H	-0.67254600	-4.13135200	1.84360500
H	3.08837100	2.06707300	2.46808700

H	2.05061800	3.14913300	3.40865400
H	1.71313600	1.41173000	3.37209700
H	1.63473300	4.57876500	1.38504000
H	1.07689200	3.87278700	-0.13031800
H	2.71095700	3.54663800	0.45738500
H	-0.19257300	3.65954600	2.77092800
H	-0.95214200	3.07773300	1.30277000
H	-0.67554000	1.96561100	2.64653100
C	4.62670700	-1.68128400	-0.39203300
C	2.95744800	-2.04169000	-2.22517600
C	4.18049600	0.13358700	-2.06254700
H	4.20188600	-2.51123200	0.17342300
H	5.42366300	-2.06660000	-1.03049900
H	5.06534800	-0.97022800	0.31083600
H	2.20032500	-1.59320200	-2.87090500
H	2.50055500	-2.86842800	-1.68352600
H	3.74904100	-2.43859900	-2.86244900
H	4.72138300	0.83435100	-1.42485900
H	4.89801300	-0.27835600	-2.77316000
H	3.42940700	0.68944500	-2.62748600

2,4,6-*tert*-butyl-pyrimidinium$^+$ [(B1)H]$^+$

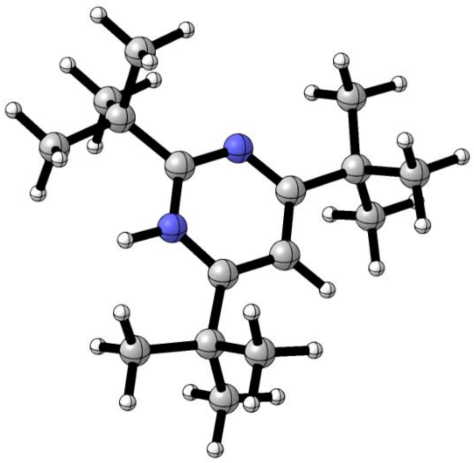

IEFPCM(CH_2Cl_2) wB97xd/def2-TZVP Electronic Energy = –736.598017

IEFPCM(CH_2Cl_2) wB97xd/def2-TZVP Free Energy = –736.218348 (T = 298.15 K)

Number of imaginary frequencies = 0

C	-0.15913500	-1.38728800	0.00884400
C	-1.26811600	-0.58128400	0.00451200
N	-1.03880500	0.74780800	0.00390000
C	0.19194500	1.28681400	0.00344800
N	1.25337500	0.51949500	0.00807200

C	1.11000700	-0.80661800	0.01122200
C	0.35709700	2.79219700	-0.00004600
C	-2.69445400	-1.09419800	-0.00001600
C	1.11943000	3.17550600	1.27826800
C	-3.71354800	0.04740900	-0.01666200
C	-2.88515100	-1.96458100	-1.25277400
C	-2.89950500	-1.94289500	1.26529800
C	1.19077400	3.16473800	-1.23573400
C	-0.98438900	3.52559900	-0.04264500
C	2.35955100	-1.66592000	0.00163100
C	2.31948400	-2.61945500	1.20702500
C	2.36787700	-2.47811600	-1.30535400
C	3.62205300	-0.80881600	0.07472500
H	-1.83397900	1.37241100	0.00279600
H	-0.27923500	-2.45908100	0.00868700
H	1.28805800	4.25278400	1.28181400
H	0.54914800	2.91565000	2.17172400
H	2.08421200	2.67164700	1.31970000
H	-4.71656400	-0.37656100	-0.01961800
H	-3.63968900	0.68105400	0.87055800
H	-3.62684400	0.66573900	-0.91350500
H	-2.20522300	-2.81645800	-1.25850300
H	-2.72296600	-1.38529300	-2.16275100
H	-3.90537600	-2.34888300	-1.26714200
H	-2.21948400	-2.79432900	1.29249800
H	-2.74655800	-1.34833500	2.16689000
H	-3.92006500	-2.32649800	1.27540700
H	1.36019900	4.24184500	-1.23881700
H	0.67061000	2.89773500	-2.15708500
H	2.15562900	2.65992500	-1.21957900
H	-1.55217500	3.29792500	-0.94859200
H	-1.60011600	3.31758000	0.83635100
H	-0.79720000	4.59854300	-0.04785000
H	3.22616200	-3.22557200	1.21195700
H	2.27623200	-2.06377200	2.14522800
H	1.46750000	-3.29906000	1.16823000
H	3.26681400	-3.09502300	-1.33655100
H	2.37593000	-1.81888900	-2.17503900
H	1.50400100	-3.13987900	-1.38135200
H	3.69296600	-0.12823500	-0.77335900
H	3.64781000	-0.21351200	0.98771900
H	4.49469100	-1.46312800	0.06592400

Tetralin 2

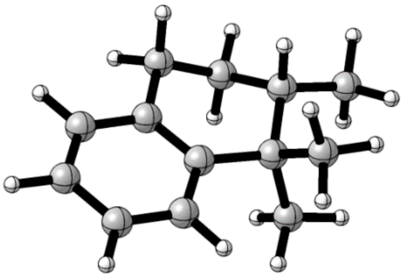

IEFPCM(CH_2Cl_2) wB97xd/def2-TZVP Electronic Energy = –506.265809

IEFPCM(CH_2Cl_2) wB97xd/def2-TZVP Free Energy = –506.022843 (T = 298.15 K)

Number of imaginary frequencies = 0

C	2.87958300	-1.10743900	0.07041200
C	3.31801700	0.20783500	0.00099700
C	2.38392300	1.22280300	-0.06963800
C	1.01344700	0.95945900	-0.08112100
C	0.56446700	-0.36273900	-0.02612900
C	1.52378900	-1.37644200	0.05891300
C	0.05515500	2.11976800	-0.15240900
C	-1.32869900	1.74270300	0.33745100
C	-1.81655100	0.47980600	-0.36244500
C	-0.92318700	-0.74663800	-0.02809600
C	-1.16590800	-1.82359900	-1.09815300
C	-1.27521900	-1.32957700	1.35054600
C	-3.30155100	0.26353100	-0.08041000
H	3.59137800	-1.92094100	0.13617100
H	4.37618800	0.43755600	0.01030700
H	2.71456500	2.25508800	-0.11051800
H	1.20491500	-2.40933000	0.12125500
H	0.45782200	2.95777600	0.42042700
H	-0.01321700	2.46245700	-1.19092300
H	-1.31468500	1.58575500	1.42096300
H	-2.02801100	2.56057400	0.14922400
H	-1.70737500	0.65238900	-1.44045400
H	-2.22661100	-2.06895600	-1.16476500
H	-0.83404900	-1.47608100	-2.07853800
H	-0.63703800	-2.74924500	-0.87086100
H	-1.19635000	-0.57630600	2.13663800
H	-2.29030600	-1.72909800	1.35901800
H	-0.59697700	-2.14489900	1.60522000
H	-3.68353400	-0.66278300	-0.51087200
H	-3.50074800	0.24280300	0.99306300
H	-3.87957500	1.08641100	-0.50486500

PF_6^-

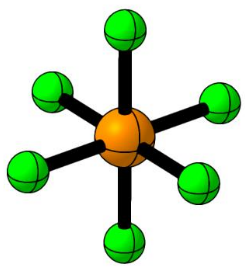

IEFPCM(CH_2Cl_2) wB97xd/def2-TZVP Electronic Energy = –940.913415

IEFPCM(CH_2Cl_2) wB97xd/def2-TZVP Free Energy = –940.921942 (T = 298.15 K)

Number of imaginary frequencies = 0

P	0.00000000	0.00000000	0.00000000
F	0.00000000	0.00000000	1.61835900
F	0.00000000	1.61835900	0.00000000
F	1.61835900	0.00000000	0.00000000
F	0.00000000	0.00000000	-1.61835900
F	0.00000000	-1.61835900	0.00000000
F	-1.61835900	0.00000000	0.00000000

Wheland PF_6 adduct (VII)

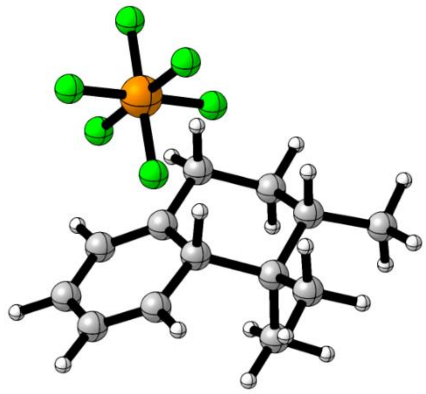

IEFPCM(CH_2Cl_2) wB97xd/def2-TZVP Electronic Energy = –1447.582367

IEFPCM(CH_2Cl_2) wB97xd/def2-TZVP Free Energy = –1447.324428 (T = 298.15 K)

Number of imaginary frequencies = 0

C	0.98762900	3.28222600	0.37738900
C	0.88283000	2.94359500	-0.98321300
C	3.62198100	0.21515000	-0.82168500
C	0.97626300	1.64095200	-1.33996900
C	1.14024500	2.32987300	1.37307400
C	1.92013800	-1.09104100	-2.11268800
C	1.15227800	0.57031400	-0.35875400
C	2.26989500	-0.49966800	-0.74628700

C	1.19947900	0.99594800	1.04581400
C	2.24423800	-1.62162000	0.32726900
C	1.27245300	-0.06225100	2.07804200
C	2.36053100	-1.08411500	1.75049500
H	0.21590100	-0.01750500	-0.42561300
C	3.31178300	-2.69405500	0.11331600
H	0.93175600	4.32693300	0.65974400
H	0.73358800	3.71781400	-1.72170000
H	3.59556600	1.01144200	-1.56715000
H	3.91004200	0.65967700	0.13199900
H	4.40353600	-0.48360800	-1.11939900
H	0.89706400	1.36406100	-2.38187600
H	1.18788900	2.63665700	2.40896100
H	0.93095200	-1.55241500	-2.10327500
H	1.94619500	-0.34324300	-2.90424100
H	2.64888100	-1.85228300	-2.38600300
H	1.26449700	-2.10310500	0.23760200
H	1.41217900	0.37455900	3.06571500
H	0.29916200	-0.56603200	2.07279400
H	3.34571100	-0.63604600	1.90477400
H	2.28303600	-1.91251000	2.45532100
H	4.31695700	-2.29112400	0.24977600
H	3.17548000	-3.48857800	0.84809000
H	3.25698800	-3.15114500	-0.87334500
P	-2.63423400	-0.36598000	-0.04007400
F	-1.85732000	0.83470800	0.72990000
F	-3.29185100	-0.83236900	1.35381000
F	-3.87778300	0.61447100	-0.32504100
F	-3.37672800	-1.56830500	-0.81069100
F	-1.93824800	0.09902700	-1.43113600
F	-1.35588200	-1.34261100	0.24228100

TS-VII

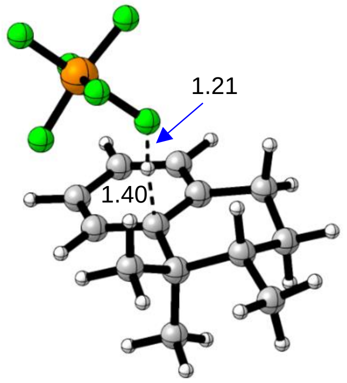

IEFPCM(CH_2Cl_2) wB97xd/def2-TZVP Electronic Energy = –1447.568917

IEFPCM(CH_2Cl_2) wB97xd/def2-TZVP Free Energy = –1447.312641 (T = 298.15 K)

Number of imaginary frequencies = 1 (895.22i cm^{-1})

C	-0.11277000	3.23100400	0.14352900
C	0.01022200	2.56565300	1.36112300
C	-3.26621800	-0.14968900	1.62067900
C	-0.56204400	1.32930500	1.51319300
C	-0.80159000	2.65264200	-0.90060600
C	-1.23426600	-1.59906700	1.55837900
C	-1.24593800	0.67778400	0.44956300
C	-2.07345500	-0.59357800	0.75833200
C	-1.39377700	1.39834700	-0.76883800
C	-2.53630200	-1.26456500	-0.56434100
C	-2.09297700	0.78803600	-1.94568800
C	-3.12074000	-0.25285500	-1.54513100
H	-0.05662500	0.22671900	-0.14651200
C	-3.52102300	-2.41388300	-0.36474500
H	0.33863600	4.20617300	0.01380100
H	0.55158600	3.01798500	2.18027500
H	-2.91617500	0.33869900	2.53137100
H	-3.91063900	0.55491100	1.09307200
H	-3.86665400	-1.00909300	1.91900500
H	-0.46246100	0.81939200	2.46042600
H	-0.89551600	3.17643500	-1.84333900
H	-0.38219400	-1.95885900	0.98089100
H	-0.85674600	-1.17446100	2.48781300
H	-1.84326700	-2.45859500	1.83442300
H	-1.63370200	-1.68174600	-1.02720100
H	-2.54094700	1.58447400	-2.54112500
H	-1.33006500	0.32476200	-2.58095500
H	-3.99554800	0.23302900	-1.10423100
H	-3.47307000	-0.77396700	-2.43681100

H	-4.49034400	-2.05065400	-0.01792700
H	-3.68388000	-2.92127700	-1.31683800
H	-3.16286000	-3.15839300	0.34536600
P	2.55095000	-0.52289900	-0.12769000
F	2.64019700	1.06720300	-0.19844200
F	2.93197100	-0.60954900	-1.66981900
F	4.03990700	-0.69011000	0.36658000
F	2.25613400	-2.08856700	-0.11583900
F	1.96020000	-0.41233800	1.35201600
F	0.84397800	-0.34073400	-0.71325700

HF

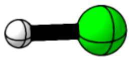

IEFPCM(CH_2Cl_2) wB97xd/def2-TZVP Electronic Energy = –100.468073

IEFPCM(CH_2Cl_2) wB97xd/def2-TZVP Free Energy = –100.475101 (T = 298.15 K)

Number of imaginary frequencies = 0

H	0.00000000	0.00000000	-0.82906400
F	0.00000000	0.00000000	0.09211800

PF_5

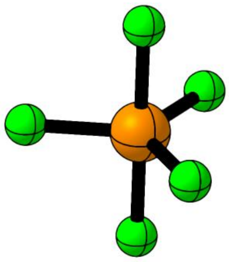

IEFPCM(CH_2Cl_2) wB97xd/def2-TZVP Electronic Energy = – -840.836648

IEFPCM(CH_2Cl_2) wB97xd/def2-TZVP Free Energy = – -840.849698 (T = 298.15 K)

Number of imaginary frequencies = 0

P	0.00002300	0.00000600	-0.00029800
F	-1.58374600	0.00027900	-0.00063900
F	0.00018400	1.33702600	-0.76675300
F	0.00001600	-0.00332900	1.54094600
F	1.58372800	-0.00006300	-0.00067400
F	-0.00022100	-1.33392200	-0.77238300

BF_4^-

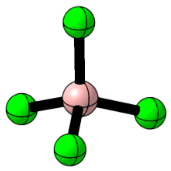

IEFPCM(CH_2Cl_2) wB97xd/def2-TZVP Electronic Energy = –424.691372

IEFPCM(CH_2Cl_2) wB97xd/def2-TZVP Free Energy = –424.704999 (T = 298.15 K)

Number of imaginary frequencies = 0

F	-0.03020500	0.32396500	1.36763200
B	-0.00011200	0.00006900	-0.00013000
F	-1.22574500	0.35196300	-0.59207100
F	1.04274200	0.70570900	-0.62573600
F	0.21327000	-1.38167500	-0.14975300

Wheland BF_4 adduct (S-V)

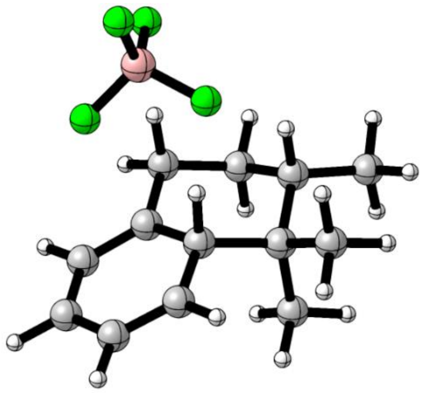

IEFPCM(CH_2Cl_2) wB97xd/def2-TZVP Electronic Energy = –931.361813

IEFPCM(CH_2Cl_2) wB97xd/def2-TZVP Free Energy = –931.104805 (T = 298.15 K)

Number of imaginary frequencies = 0

C	0.19011800	3.30326000	0.51318700
C	0.43706400	3.08550200	-0.85332600
C	3.11096500	0.20228200	-0.38711700
C	0.67497600	1.82205200	-1.28020400
C	0.15268100	2.27010700	1.43351100
C	1.64914800	-0.89916600	-2.09378000
C	0.63050400	0.66979000	-0.38247500
C	1.73938900	-0.43280800	-0.63960300
C	0.35870800	0.97021700	1.02720000
C	1.45172300	-1.63138600	0.30521900
C	0.24226900	-0.16150300	1.97588200
C	1.33094400	-1.20904600	1.76605700
H	-0.31477500	0.15500400	-0.69521400

C	2.47496100	-2.76013400	0.19302400
H	0.01625800	4.31582000	0.85757500
H	0.44311400	3.91909600	-1.54065700
H	3.27287100	1.04543200	-1.06088900
H	3.21587900	0.56816400	0.63533000
H	3.90587300	-0.52005700	-0.57208800
H	0.87152500	1.64059100	-2.32764200
H	-0.05316600	2.48301500	2.47369600
H	2.40505700	-1.65828200	-2.28792400
H	0.66896600	-1.32681000	-2.30970200
H	1.83943500	-0.09167400	-2.79990800
H	0.47876100	-2.03001100	-0.00246800
H	0.22379200	0.20661700	3.00103900
H	-0.73506400	-0.62096800	1.78210500
H	2.28950800	-0.82867400	2.12904500
H	1.09440200	-2.08257900	2.37452100
H	2.16262200	-3.59297200	0.82450900
H	2.57086800	-3.14088200	-0.82258700
H	3.46178300	-2.44252800	0.53521900
F	-1.78123800	-1.02307200	-1.14691300
B	-2.80997700	-0.66889900	-0.23095500
F	-4.04967100	-0.80775400	-0.84919400
F	-2.72261100	-1.50537400	0.89018800
F	-2.60520500	0.66633200	0.16096200

S-TS-V

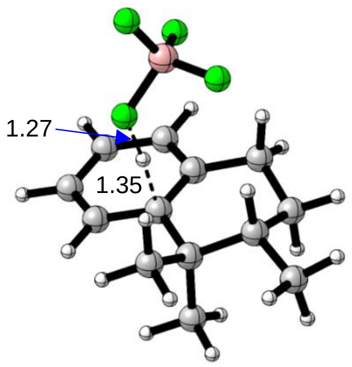

IEFPCM(CH_2Cl_2) wB97xd/def2-TZVP Electronic Energy = –931.356165

IEFPCM(CH_2Cl_2) wB97xd/def2-TZVP Free Energy = –931.101764 (T = 298.15 K)

Number of imaginary frequencies = 1 (801.11i cm^{-1})

C	-1.09401200	3.03235600	0.48328800
C	-0.54942300	3.01453100	-0.80070600
C	3.05885300	0.77671100	-0.57833100
C	0.29871600	1.99919300	-1.15413200

C	-0.78359000	2.04349800	1.39136200
C	1.56867300	-0.57672500	-2.05876000
C	0.60244800	0.92227400	-0.26623100
C	1.75849000	-0.04064200	-0.63332500
C	0.07769600	1.00153900	1.05809700
C	1.78745300	-1.23888200	0.35424500
C	0.35930900	-0.07188000	2.06112300
C	1.67267400	-0.78697300	1.80669800
H	-0.54950200	0.30247700	-0.59246100
C	3.00688200	-2.14330700	0.19391000
H	-1.76780600	3.82961300	0.76999500
H	-0.78949500	3.79558800	-1.50853700
H	3.00870100	1.62277700	-1.26541700
H	3.24907000	1.17162900	0.42075000
H	3.91054800	0.16419900	-0.87403900
H	0.71669400	1.98566600	-2.15095400
H	-1.20807500	2.07045000	2.38643100
H	2.41112000	-1.20753000	-2.33813700
H	0.65780200	-1.17133700	-2.14056500
H	1.52195100	0.22273000	-2.79798100
H	0.89779200	-1.83796300	0.13226400
H	0.32407500	0.36358400	3.06055200
H	-0.46581700	-0.78952300	2.01058200
H	2.51322500	-0.13714400	2.06630800
H	1.74013900	-1.65680100	2.46205500
H	2.88209300	-3.03216200	0.81423800
H	3.15214900	-2.47953300	-0.83221400
H	3.92036900	-1.64229400	0.52048800
F	-1.56140300	-0.30624200	-1.06399800
B	-2.40920000	-1.25326100	-0.22764600
F	-3.41384100	-1.66579400	-1.06339500
F	-1.56997400	-2.27687700	0.15593000
F	-2.86189700	-0.51756400	0.84384500

BF_3

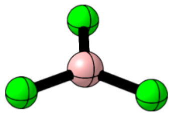

IEFPCM(CH_2Cl_2) wB97xd/def2-TZVP Electronic Energy = –324.619951

IEFPCM(CH_2Cl_2) wB97xd/def2-TZVP Free Energy = –324.633951 (T = 298.15 K)

Number of imaginary frequencies = 0

B	0.00010000	-0.00017900	-0.00006600
F	-0.84857000	-1.00275700	0.00001200
F	-0.44434400	1.23616000	0.00001200
F	1.29285800	-0.23330400	0.00001200

4. X–ray

4.1 Tetracycle **40a**

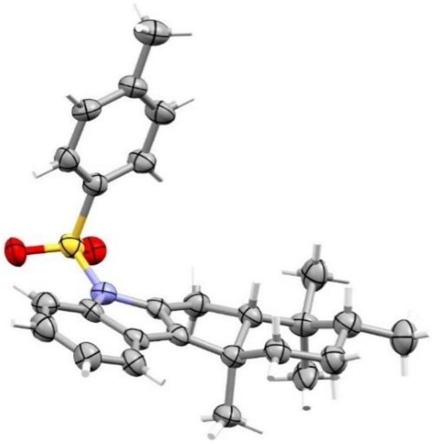

Thermal ellipsoids are shown at the 50% probability level.

Identification code	Tetracycle **40a**
Empirical formula	$C_{26}H_{31}NO_2S$
Formula weight	421.58
Temperature	173.00 K
Wavelength	0.71073 Å
Crystal system	Monoclinic
Space group	P 21/c (no. 14)
Unit cell dimensions	a = 24.618(4) Å α = 90°.<br>b = 9.8398(13) Å β = 111.625(4)°.<br>c = 19.728(3) Å γ = 90°.
Volume	4442.4(11) $Å^3$
Z	8
Density (calculated)	1.261 mg/m^3
Absorption coefficient	0.168 mm^{-1}
F(000)	1808
Crystal size	0.08 x 0.06 x 0.01 mm^3
Theta range for data collection	2.253 to 22.019°.
Index ranges	-25<=h<=25, -10<=k<=10, -20<=l<=20
Reflections collected	19483
Independent reflections	5450 [R(int) = 0.1097]
Completeness to theta = 22.019°	99.7 %
Absorption correction	Semi-empirical from equivalents
Max. and min. transmission	0.9566 and 0.8597
Refinement method	Full-matrix least-squares on F^2
Data / restraints / parameters	5450 / 0 / 551
Goodness-of-fit on F^2	1.002
Final R indices [I>2sigma(I)]	R1 = 0.0678, wR2 = 0.1633
R indices (all data)	R1 = 0.1236, wR2 = 0.2006
Extinction coefficient	n/a
Largest diff. peak and hole	0.474 and -0.474 e.$Å^{-3}$

Alert level A:

THETM01_ALERT_3_A The value of sine(theta_max)/wavelength is less than 0.550

Calculated sin(theta_max)/wavelength = 0.5275

Author Response: Thin plate with low diffraction: Crystal agglomerate with thin plate grown-ups. Only reflections until 44 degrees in 2Theta were used in the refinement. Visible reflections on frames could only be observed to a 2Theta angle of 40 degrees.

5. NMR Spectra

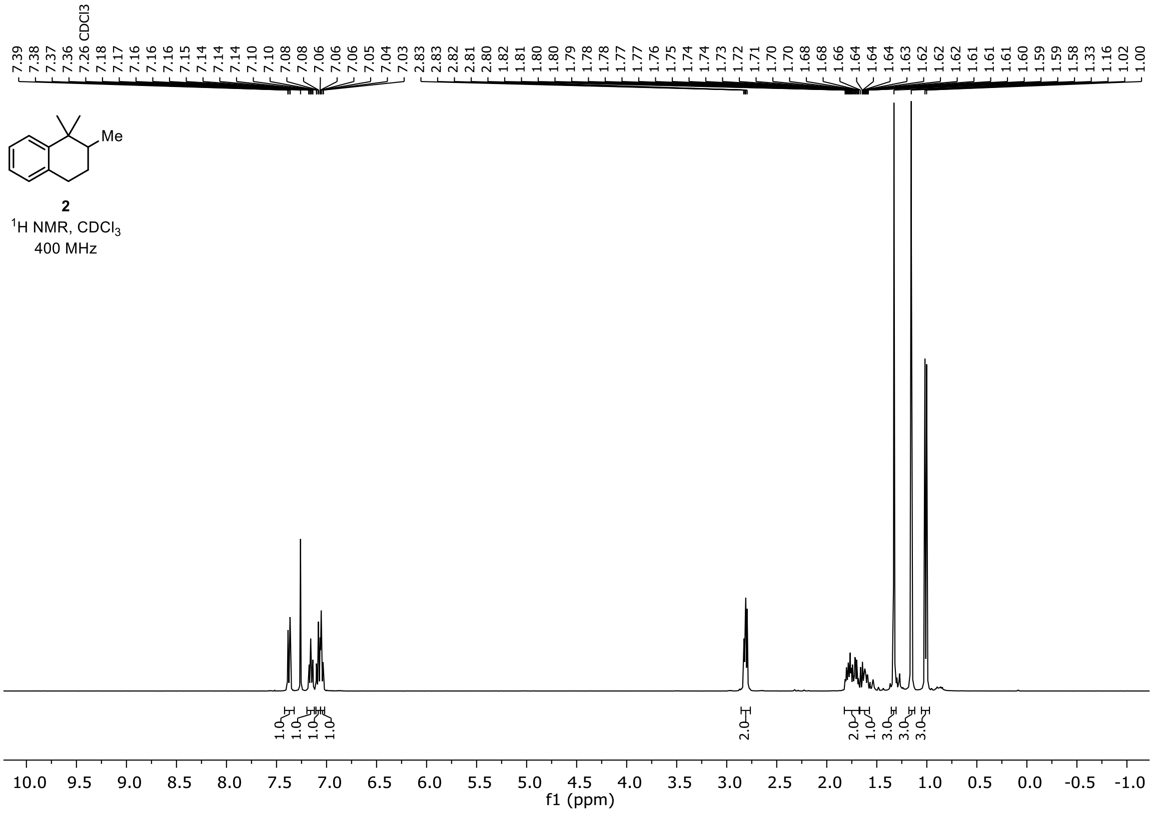

Figure 4. ^{1}H-NMR (400 MHz, $CDCl_3$) of tetralin **2**.

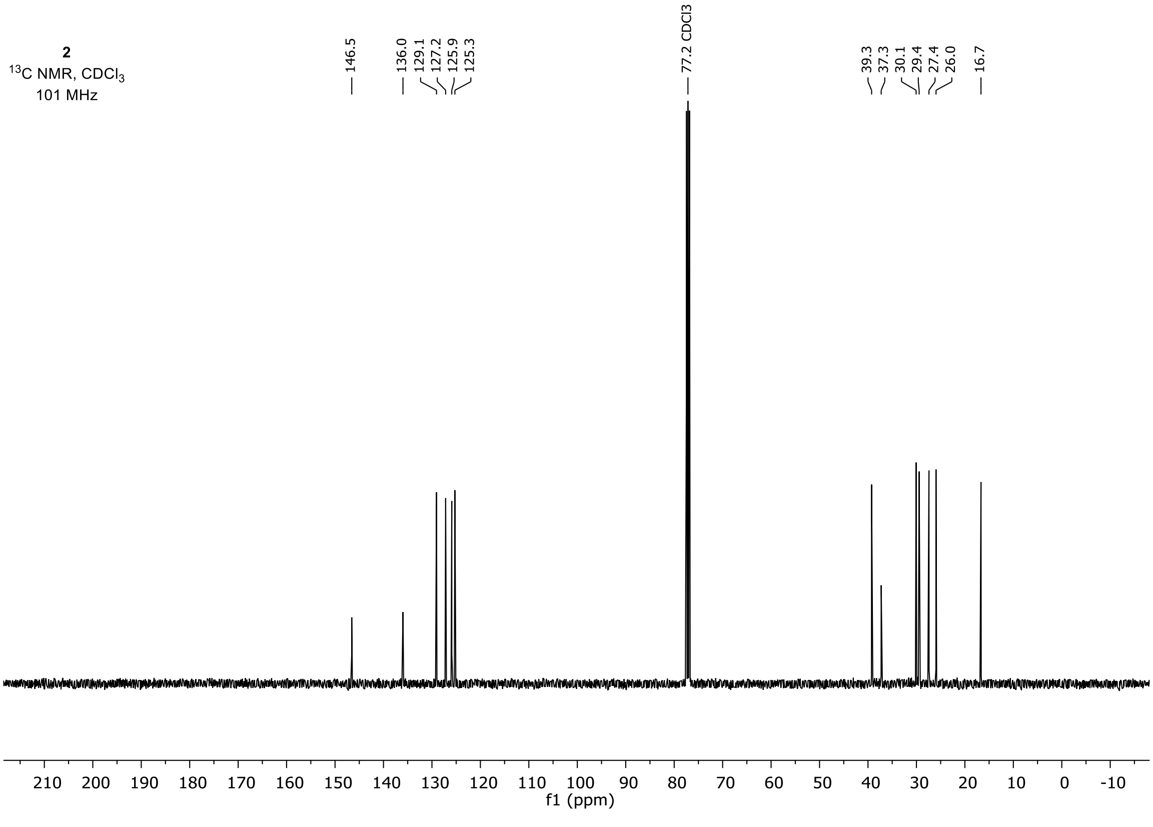

Figure 5. ^{13}C-NMR (101 MHz, $CDCl_3$) of tetralin **2**.

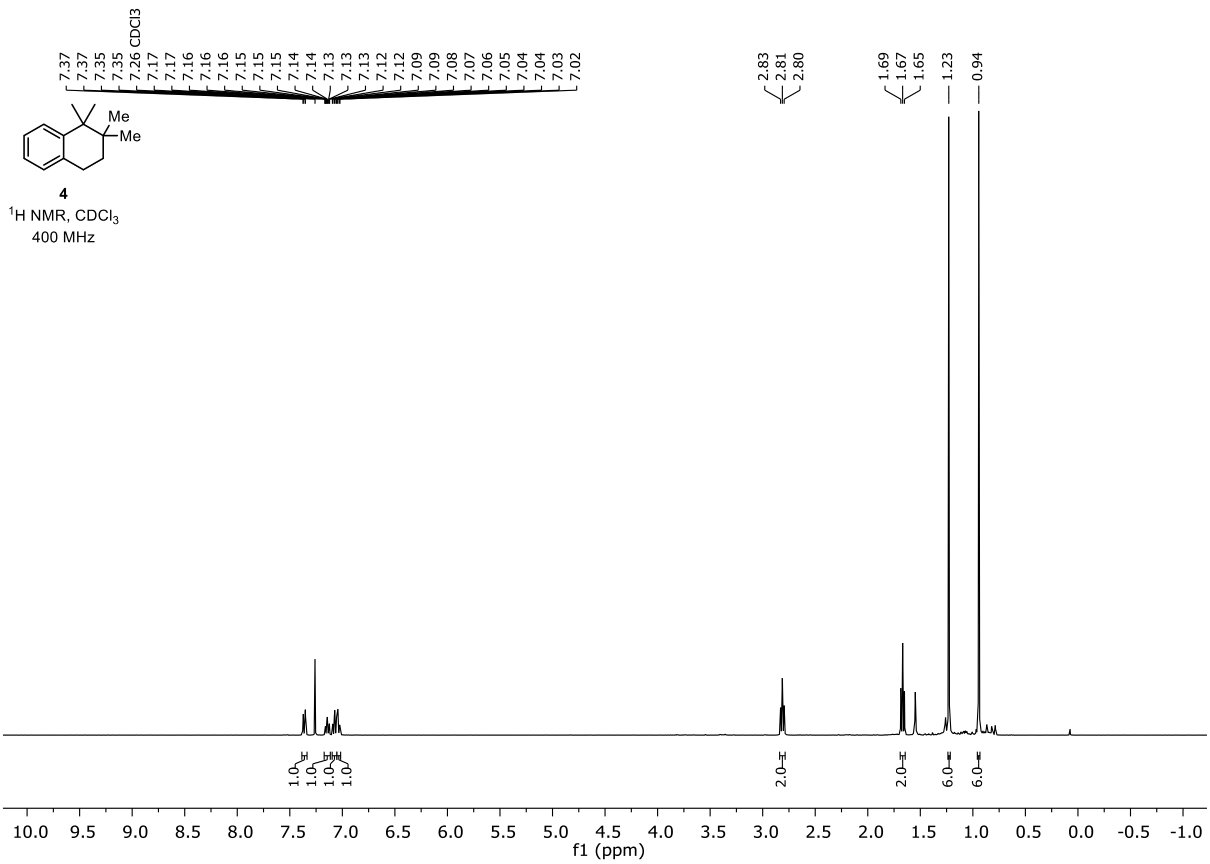

Figure 6. ^{1}H-NMR (400 MHz, $CDCl_3$) of tetralin **4**.

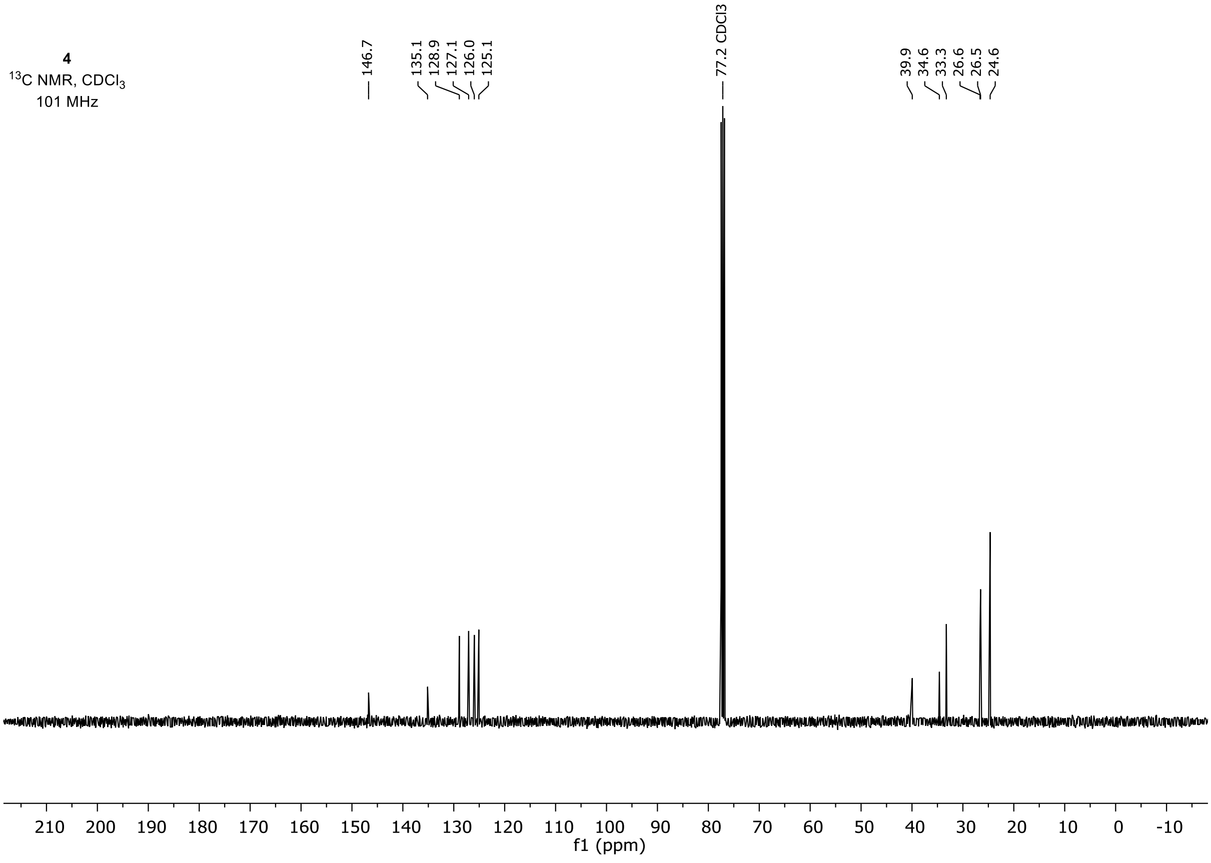

Figure 7. ^{13}C-NMR (101 MHz, $CDCl_3$) of tetralin **4**.

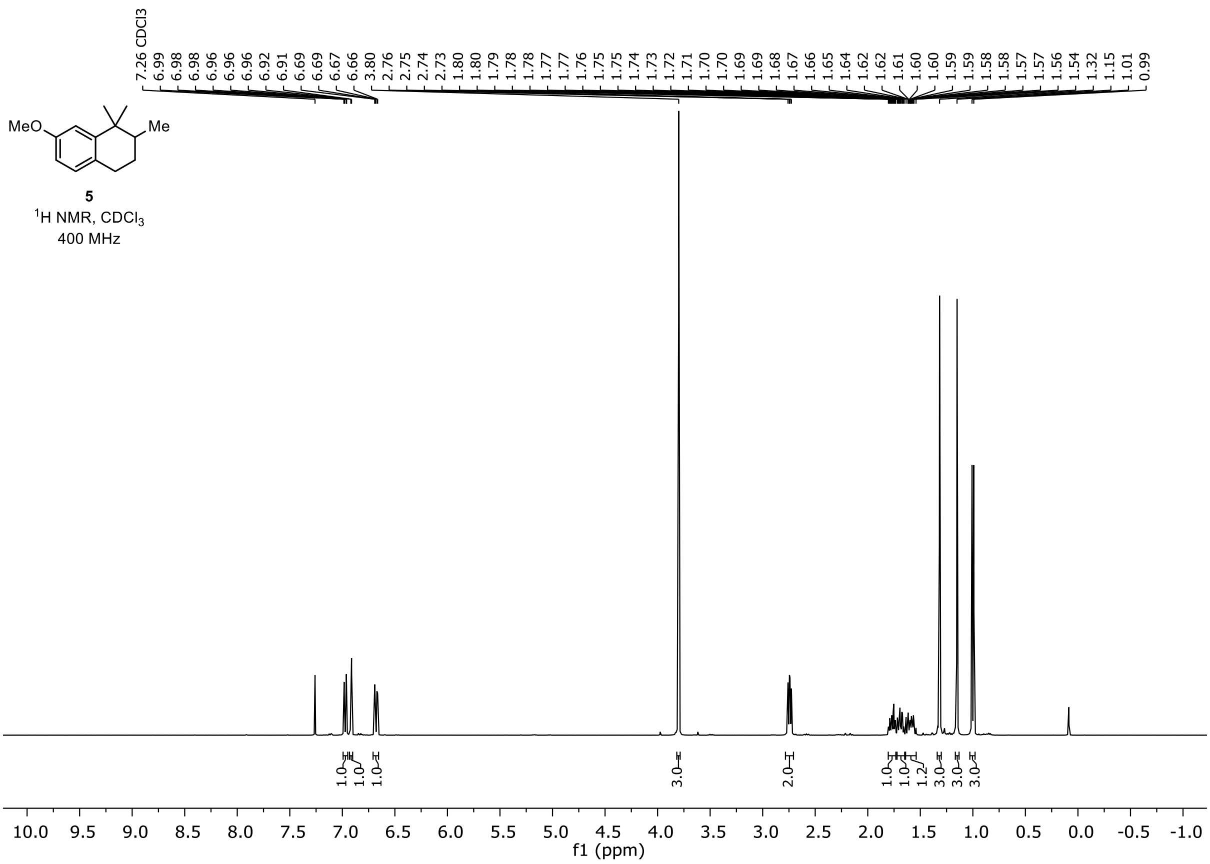

Figure 8. ^{1}H-NMR (400 MHz, $CDCl_3$) of tetralin **5**.

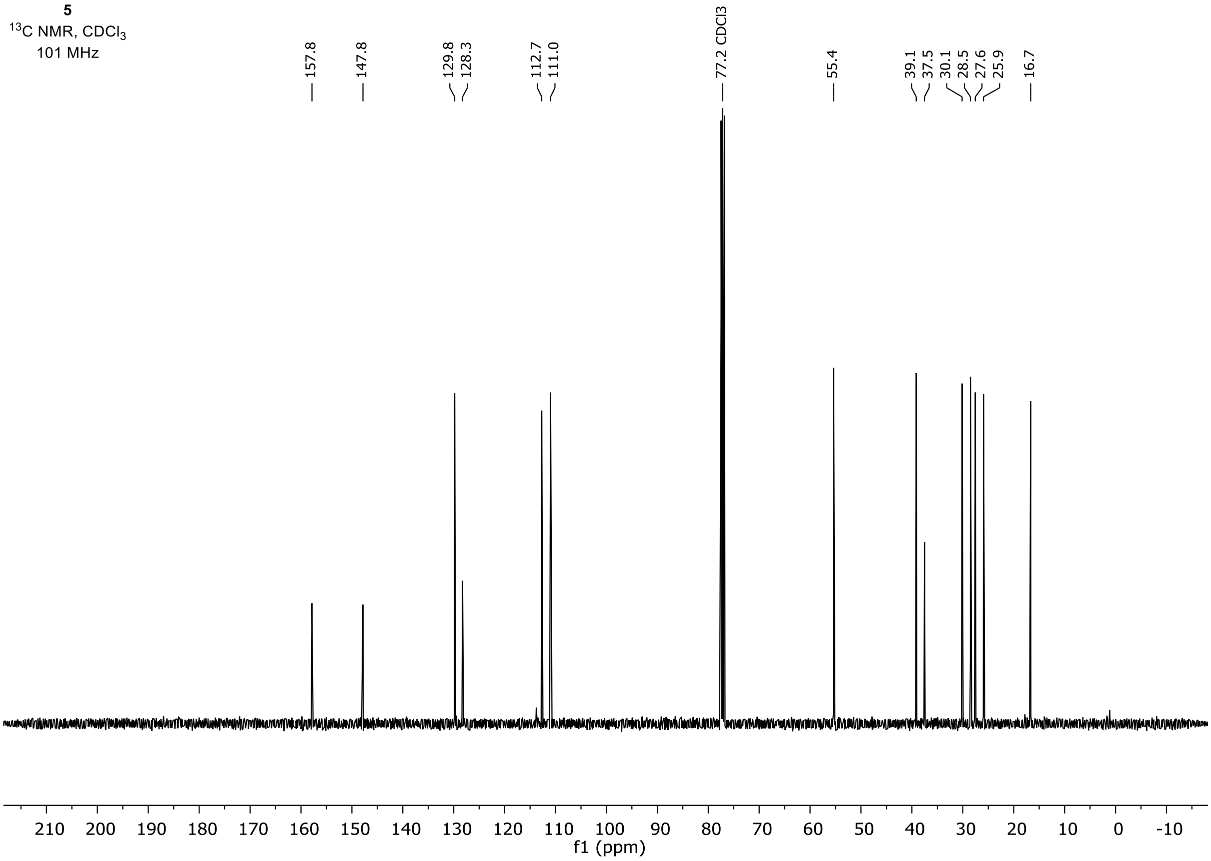

Figure 9. ^{13}C-NMR (101 MHz, $CDCl_3$) of tetralin **5**.

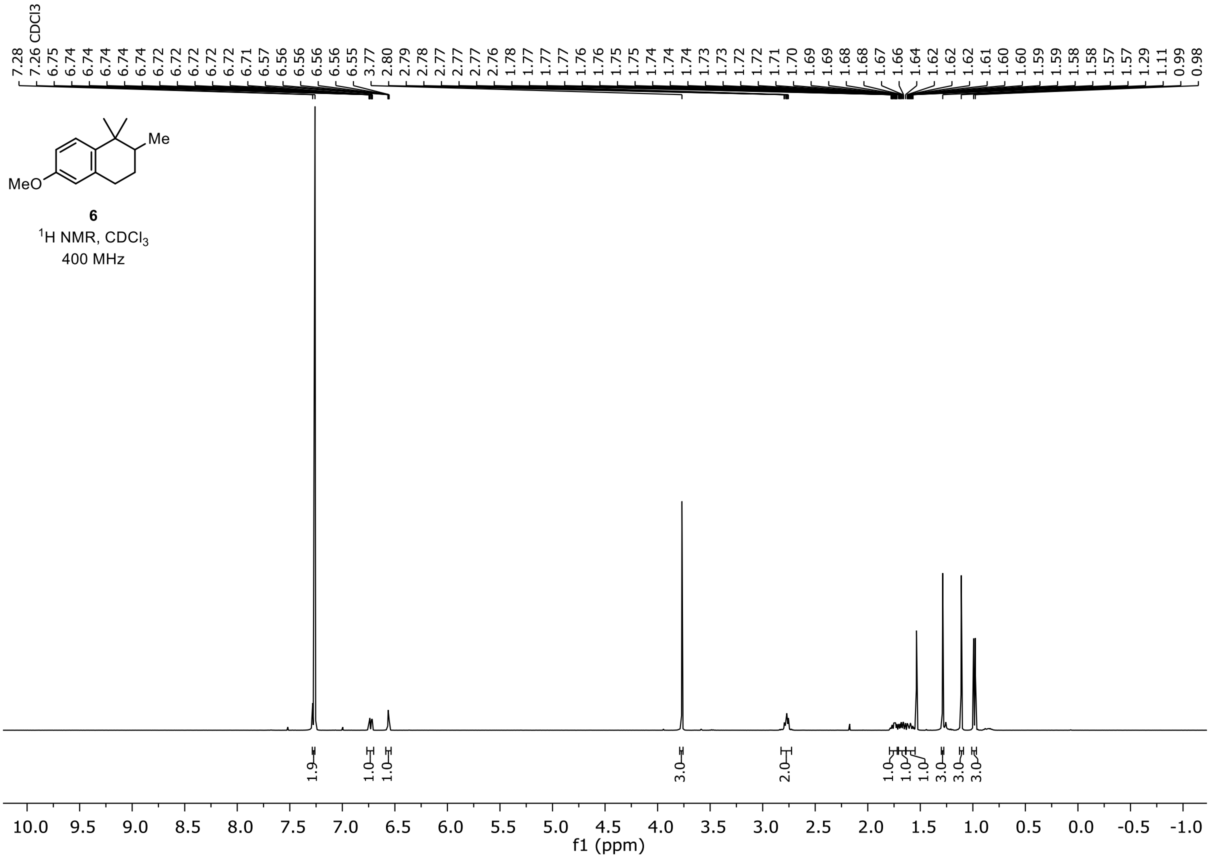

Figure 10. ^{1}H-NMR (400 MHz, $CDCl_3$) of tetralin **6**.

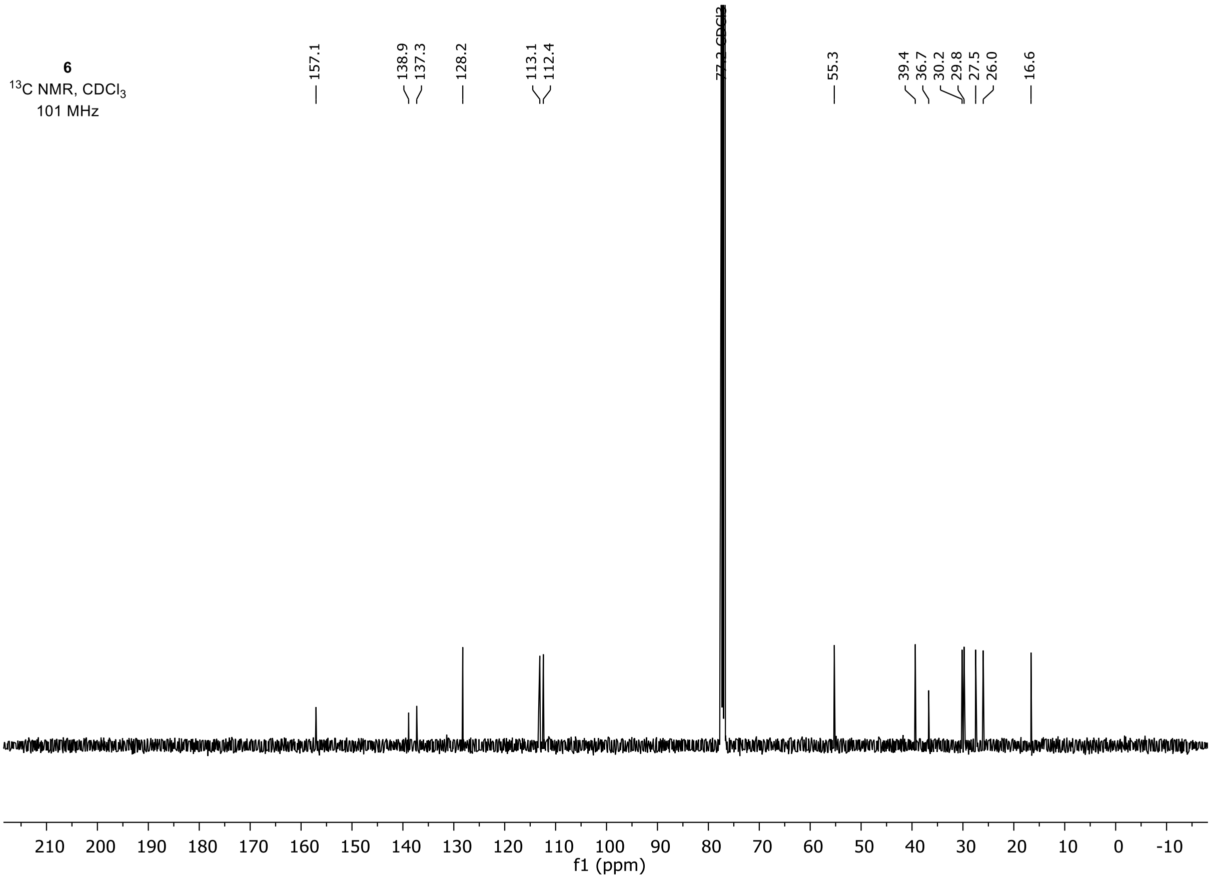

Figure 11. ^{13}C-NMR (101 MHz, $CDCl_3$) of tetralin **6**.

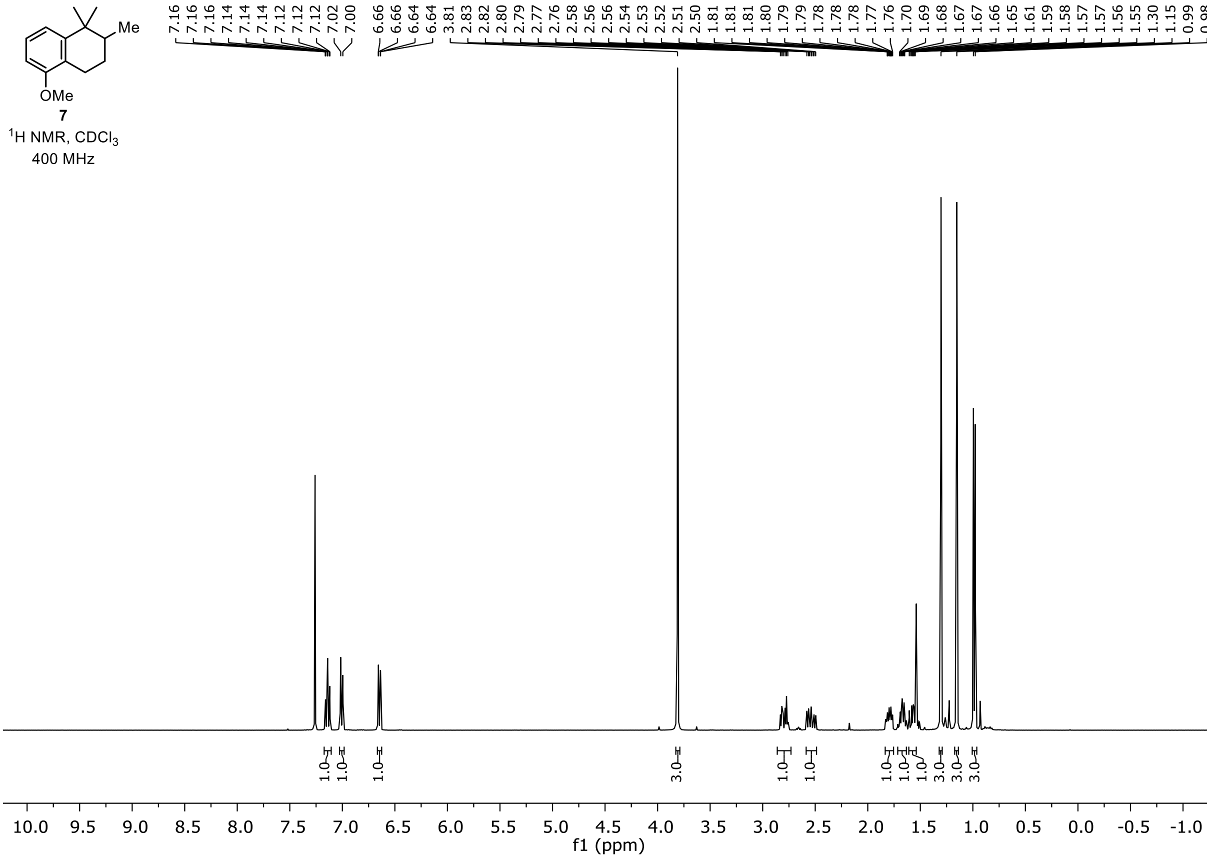

Figure 12. ^{1}H-NMR (400 MHz, $CDCl_3$) of tetralin **7**.

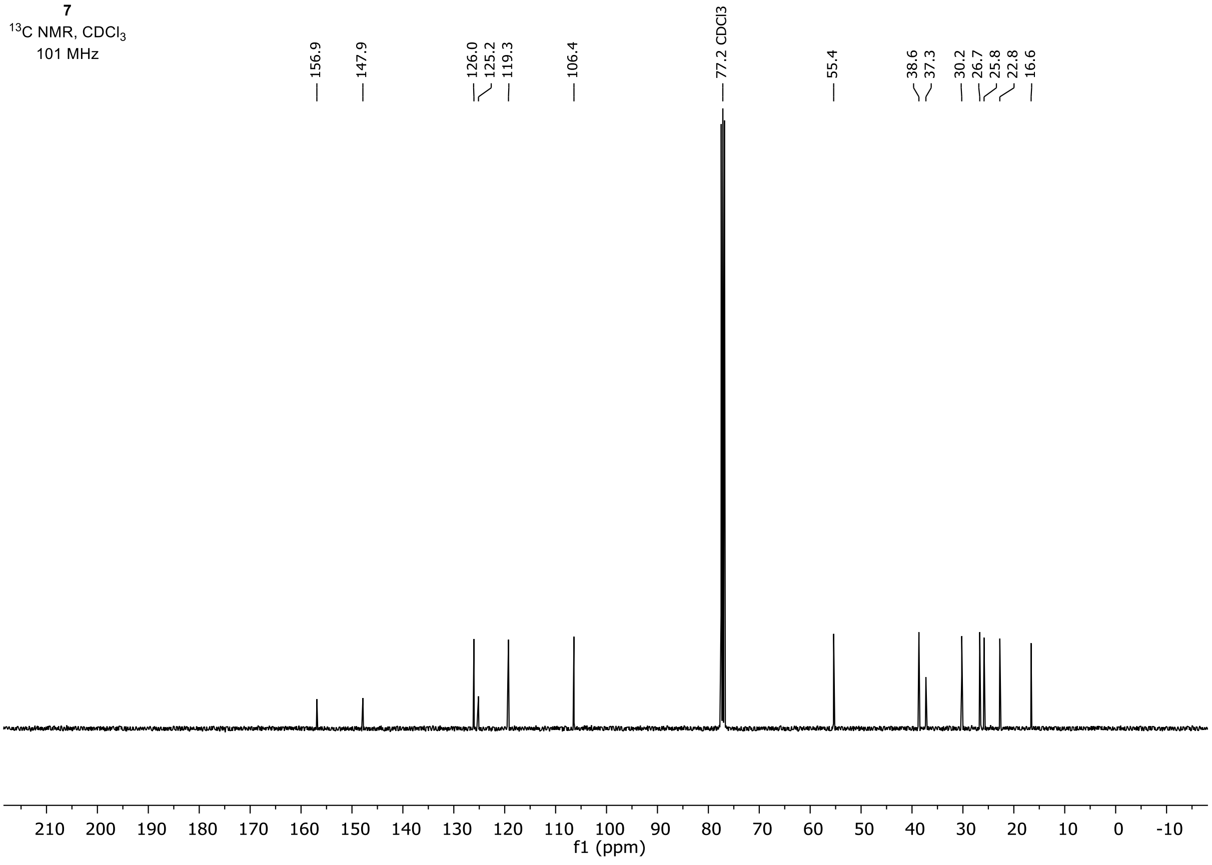

Figure 13. ^{13}C-NMR (101 MHz, $CDCl_3$) of tetralin **7**.

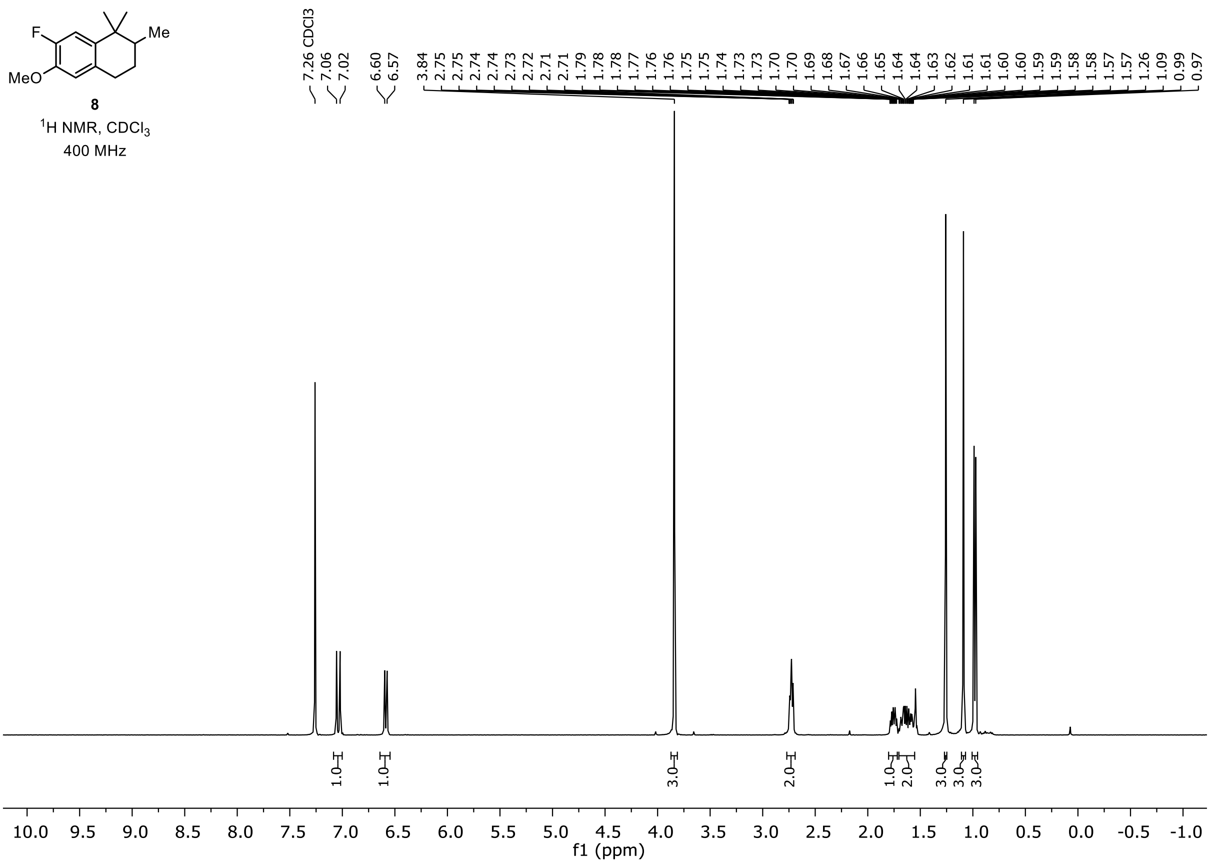

Figure 14. ^{1}H-NMR (400 MHz, $CDCl_3$) of fluorotetralin **8**.

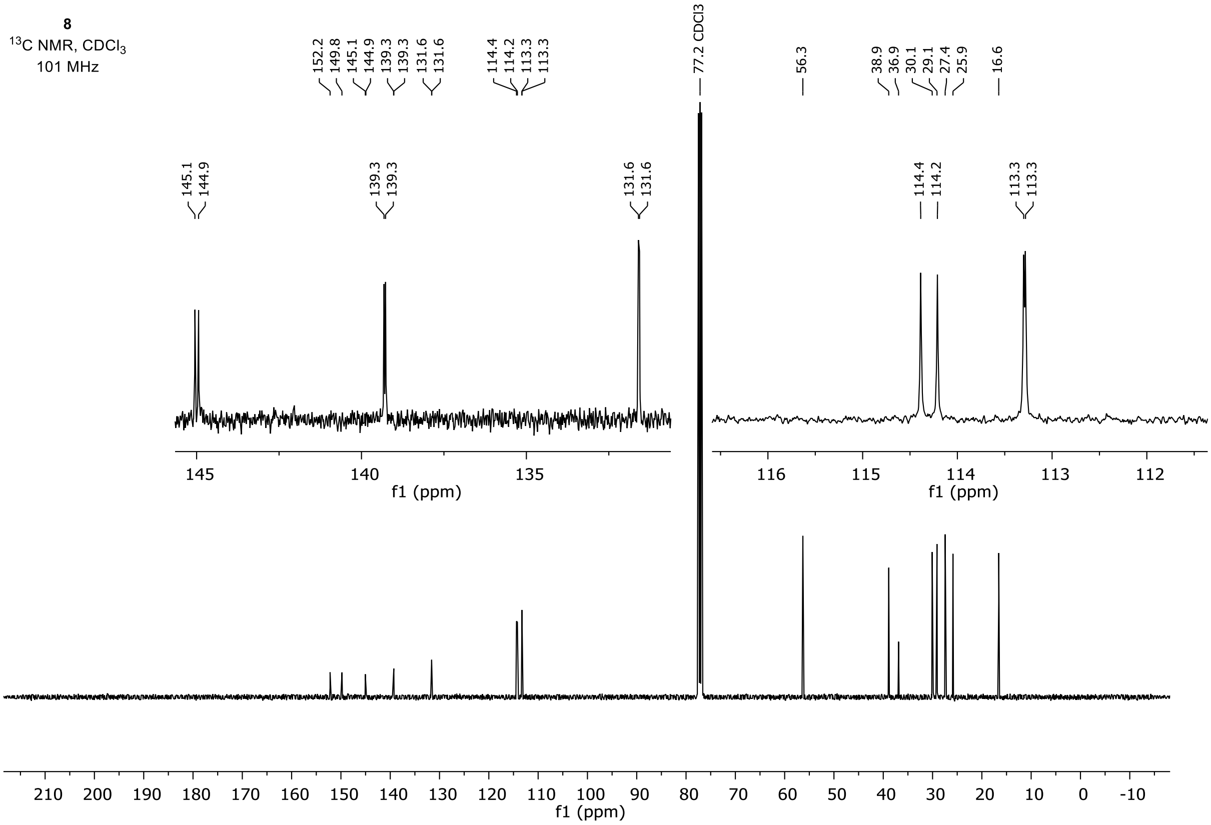

Figure 15. ^{13}C-NMR (101 MHz, $CDCl_3$) of fluorotetralin **8**.

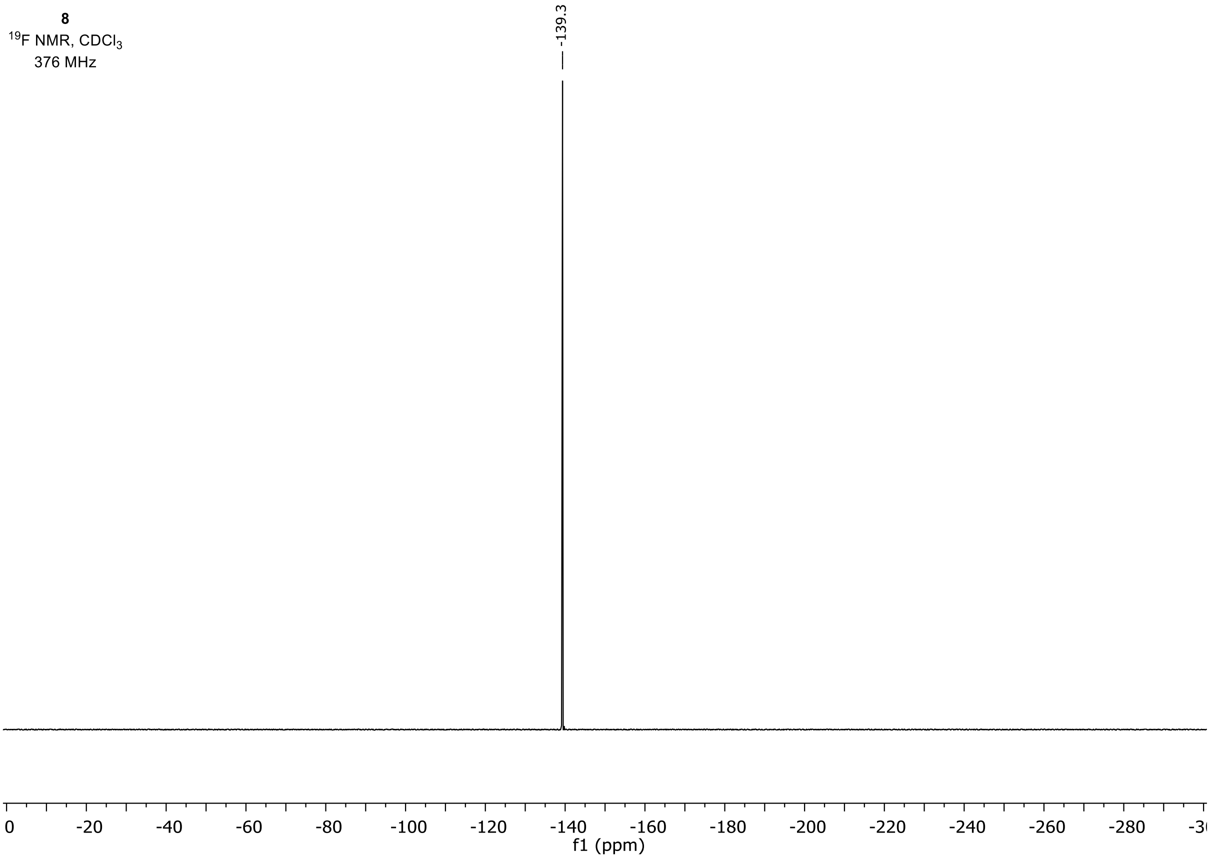

Figure 16. ^{19}F-NMR (376 MHz, $CDCl_3$) of fluorotetralin **8**.

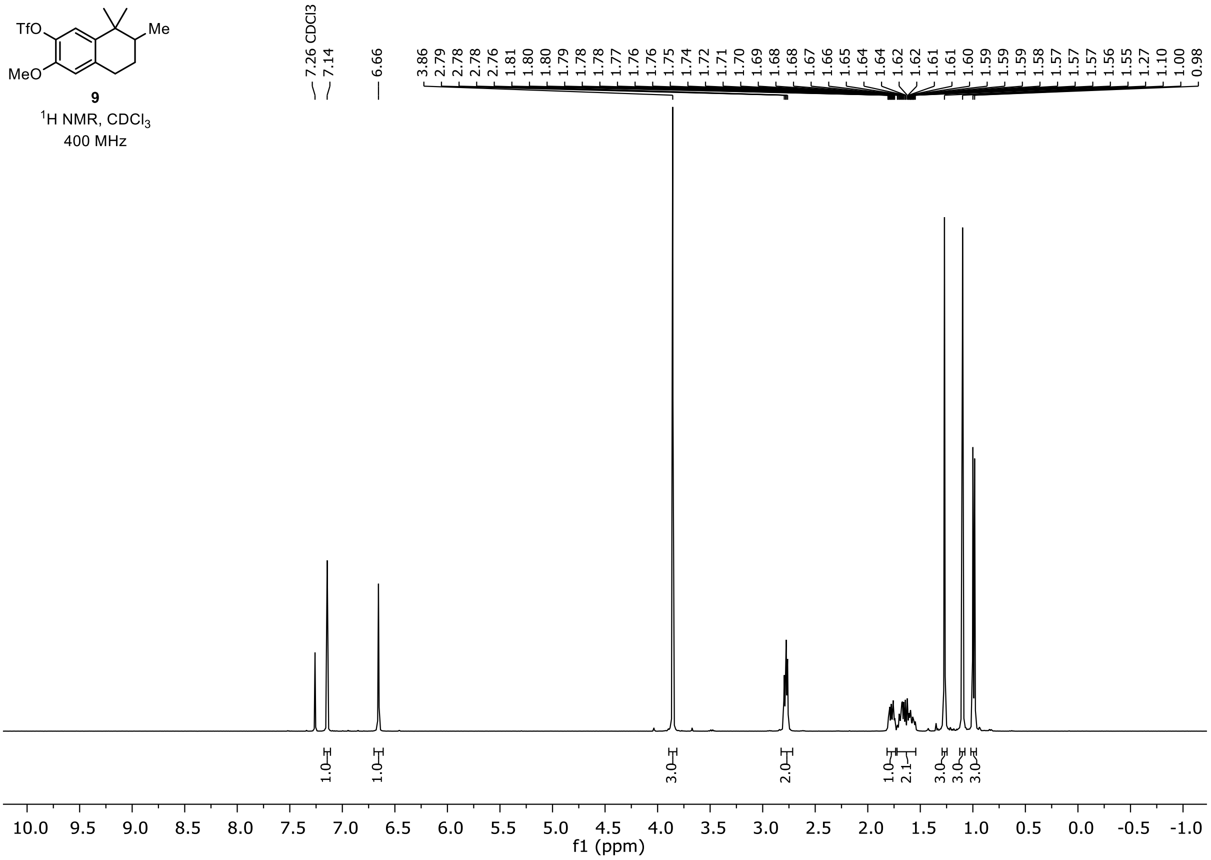

Figure 17. ^{1}H-NMR (400 MHz, $CDCl_3$) of tetralin **9**.

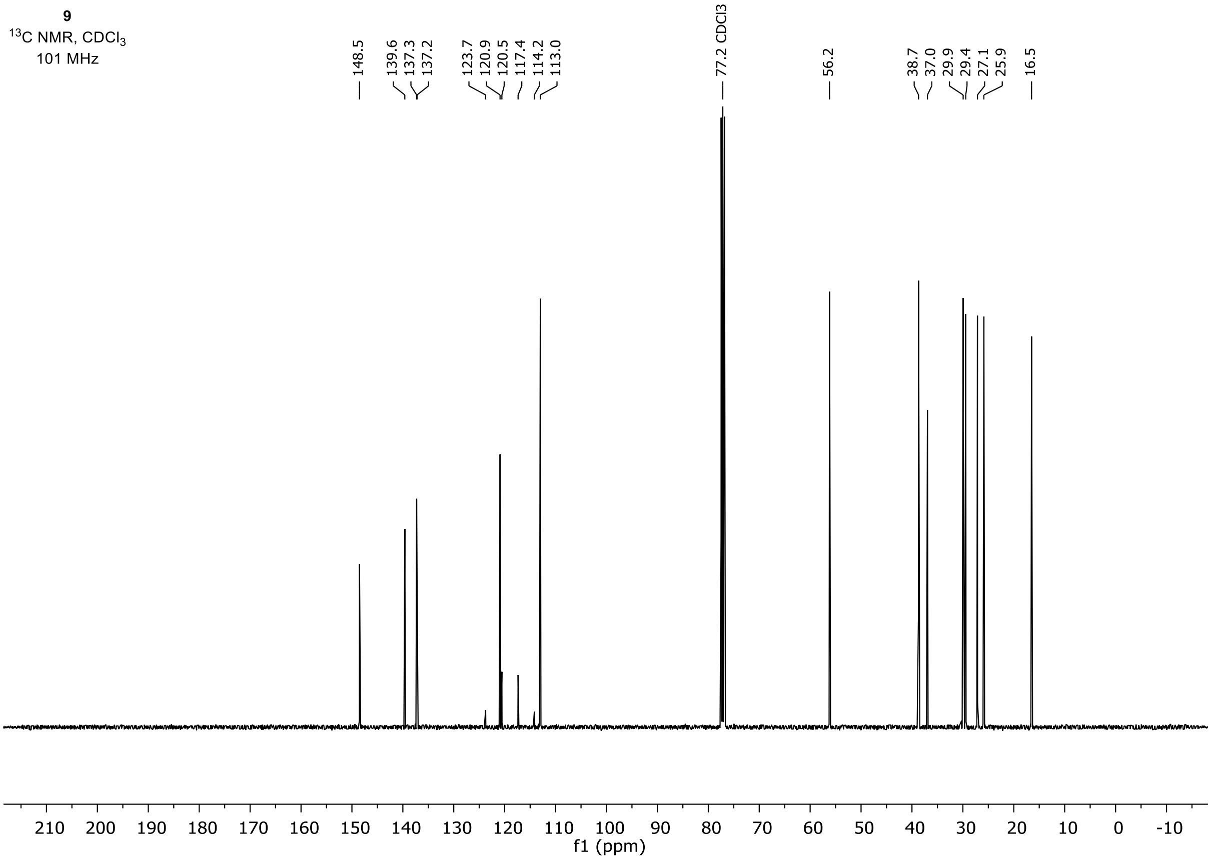

Figure 18. ^{13}C-NMR (101 MHz, $CDCl_3$) of tetralin **9**.

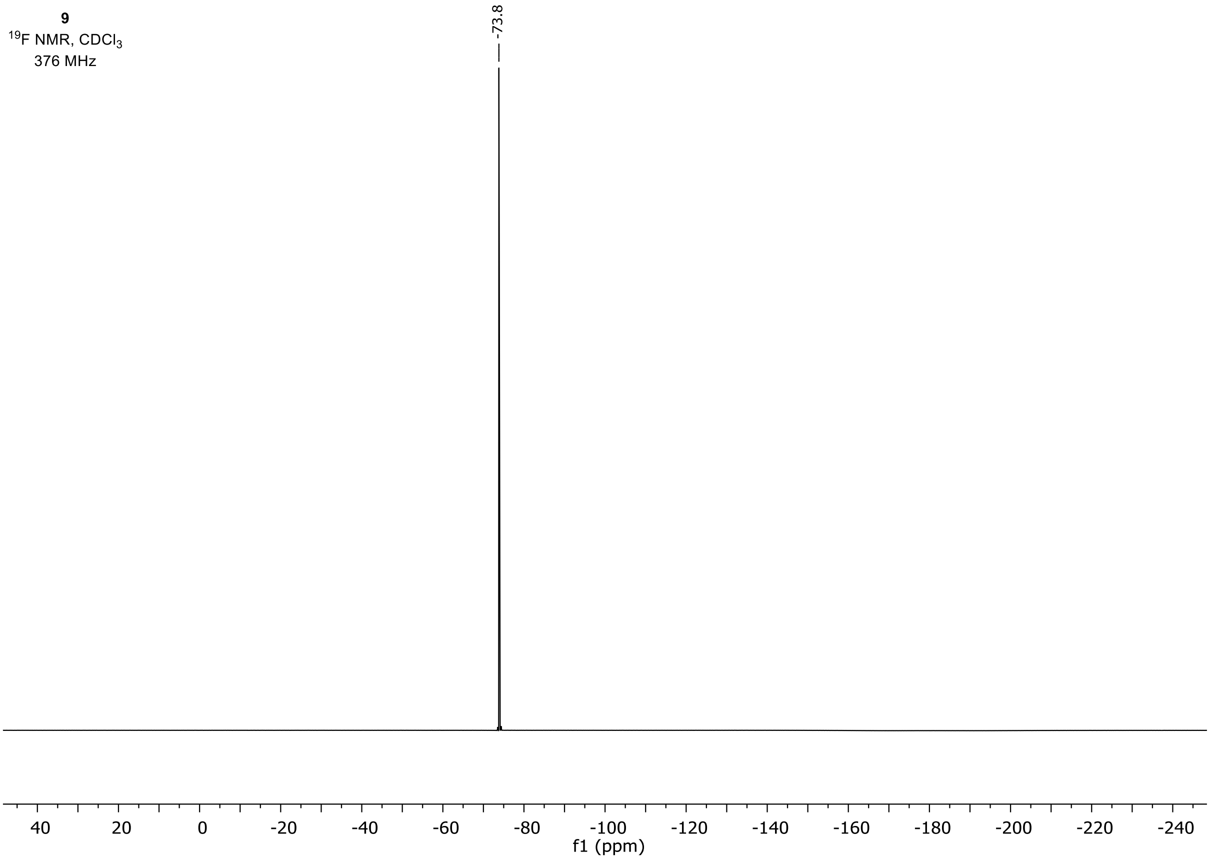

Figure 19. ^{19}F-NMR (376 MHz, $CDCl_3$) of tetralin **9**.

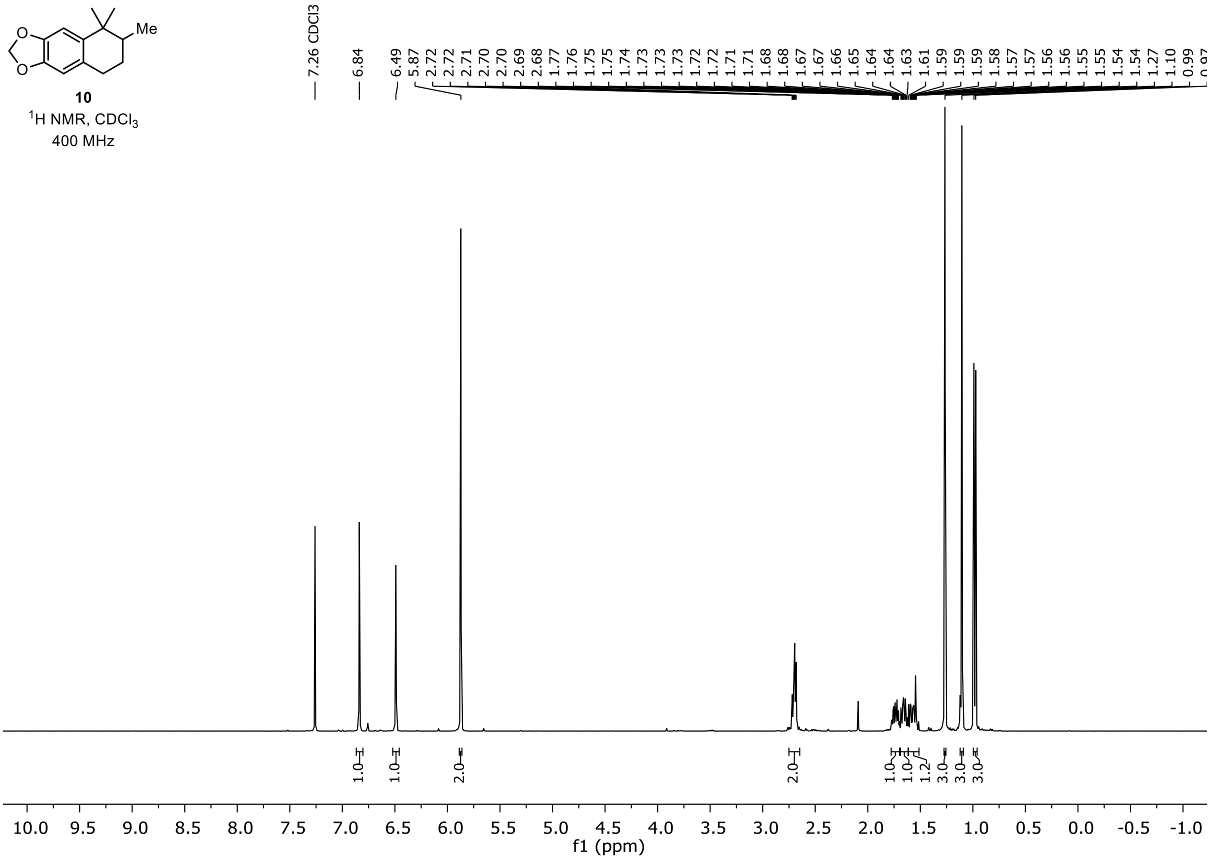

Figure 20. ^{1}H-NMR (400 MHz, $CDCl_3$) of 1,3-dioxole **10**.

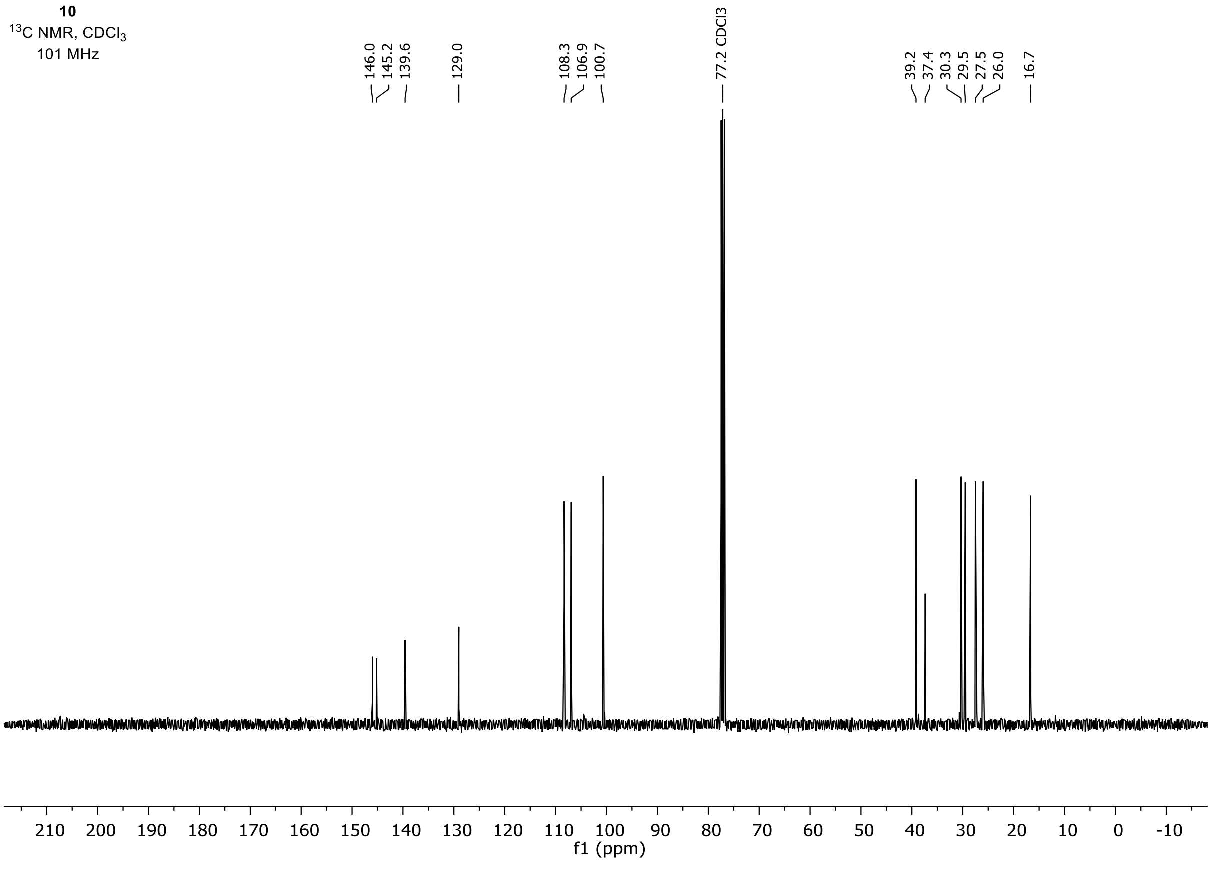

Figure 21. ^{13}C-NMR (101 MHz, $CDCl_3$) of 1,3-dioxole **10**.

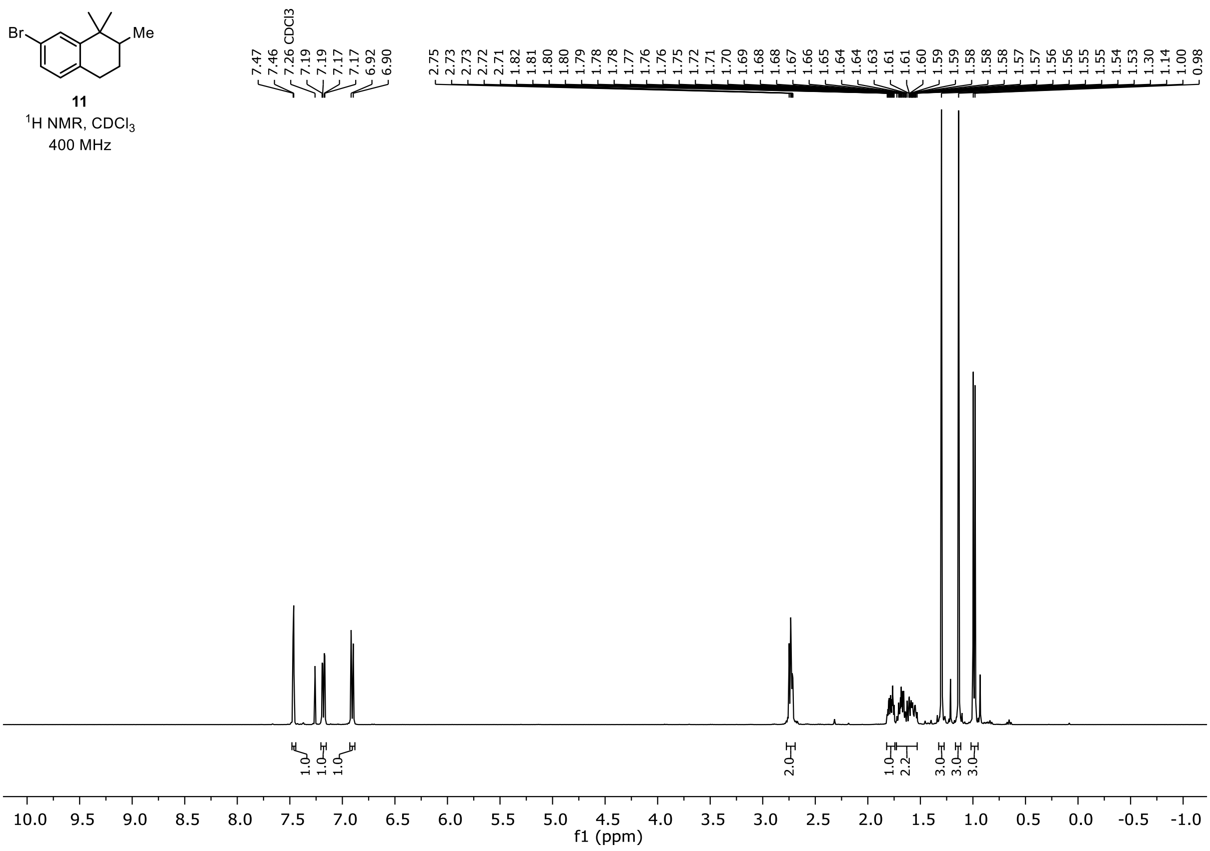

Figure 22. ^{1}H-NMR (400 MHz, $CDCl_3$) of tetralin **11**.

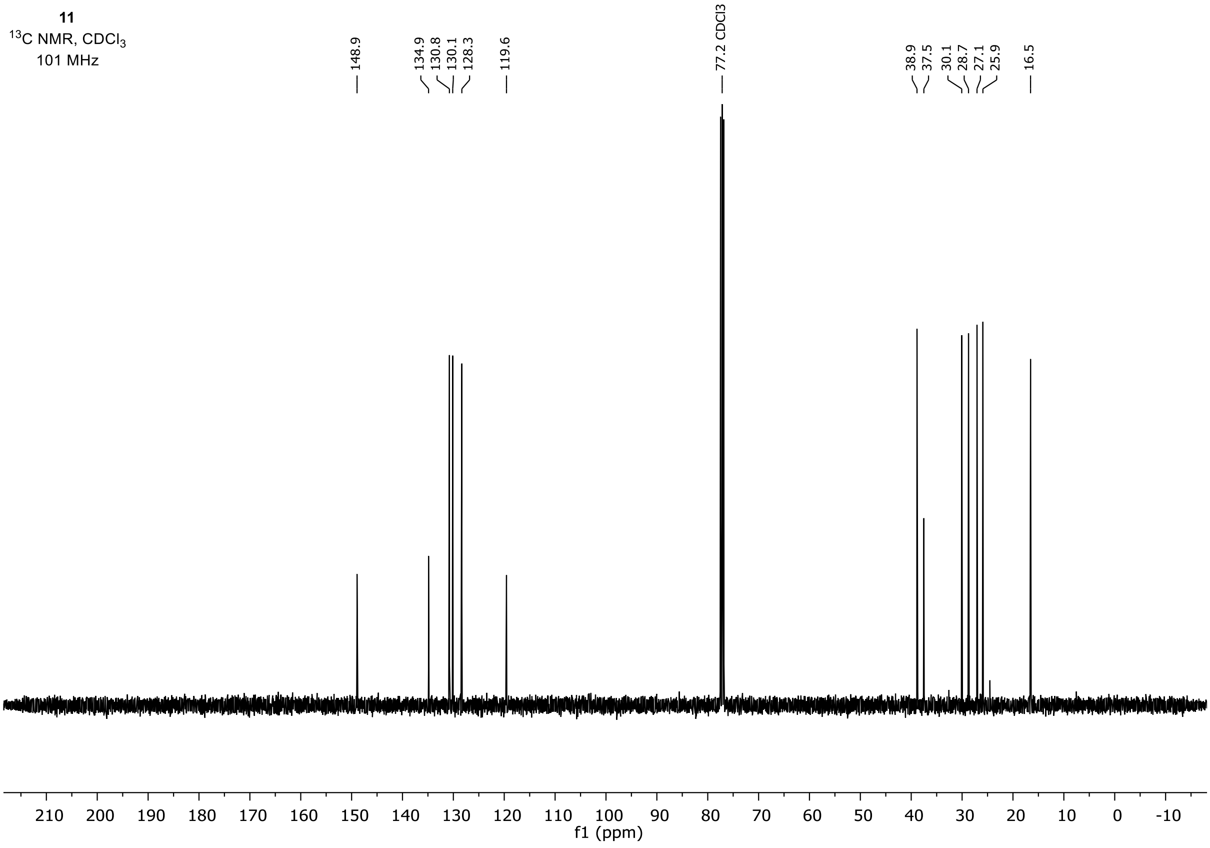

Figure 23. ^{13}C-NMR (101 MHz, $CDCl_3$) of tetralin **11**.

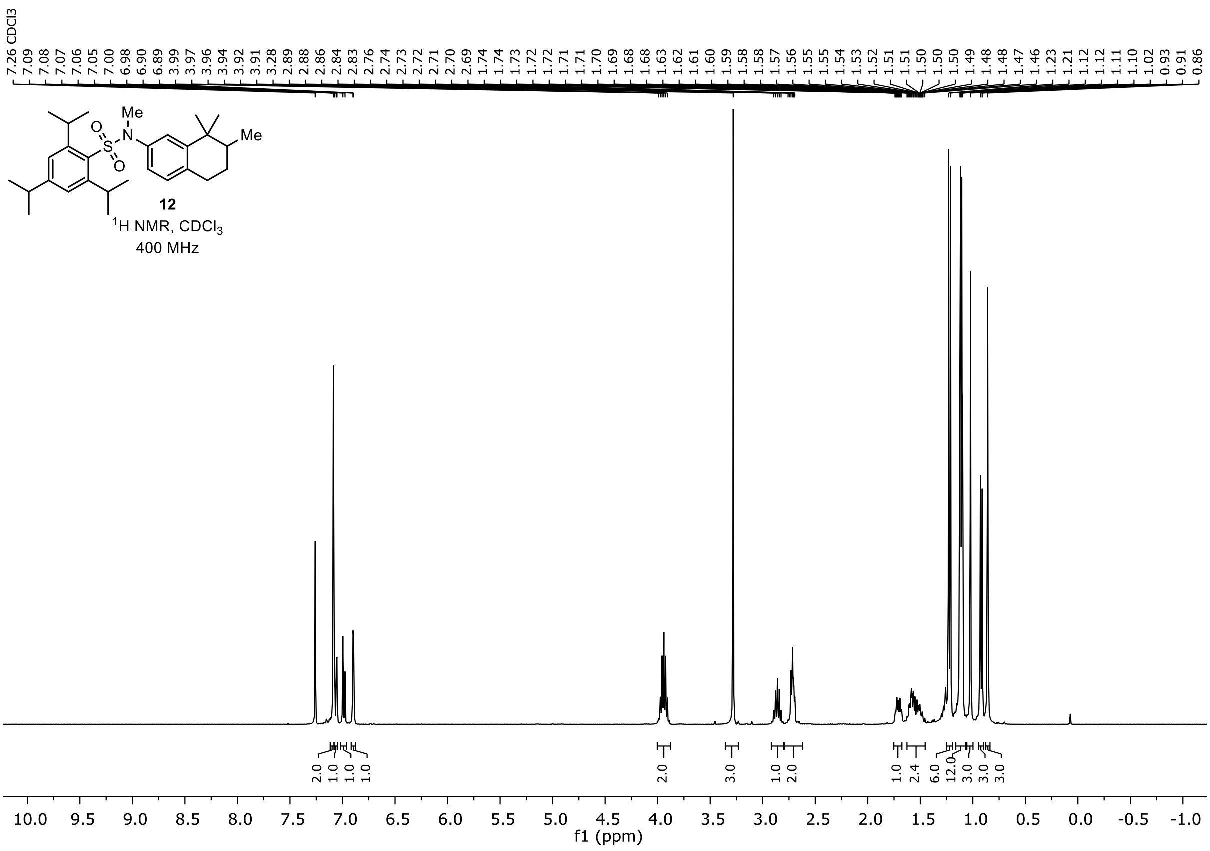

Figure 24. ^{1}H-NMR (400 MHz, $CDCl_3$) of tetralin **12**.

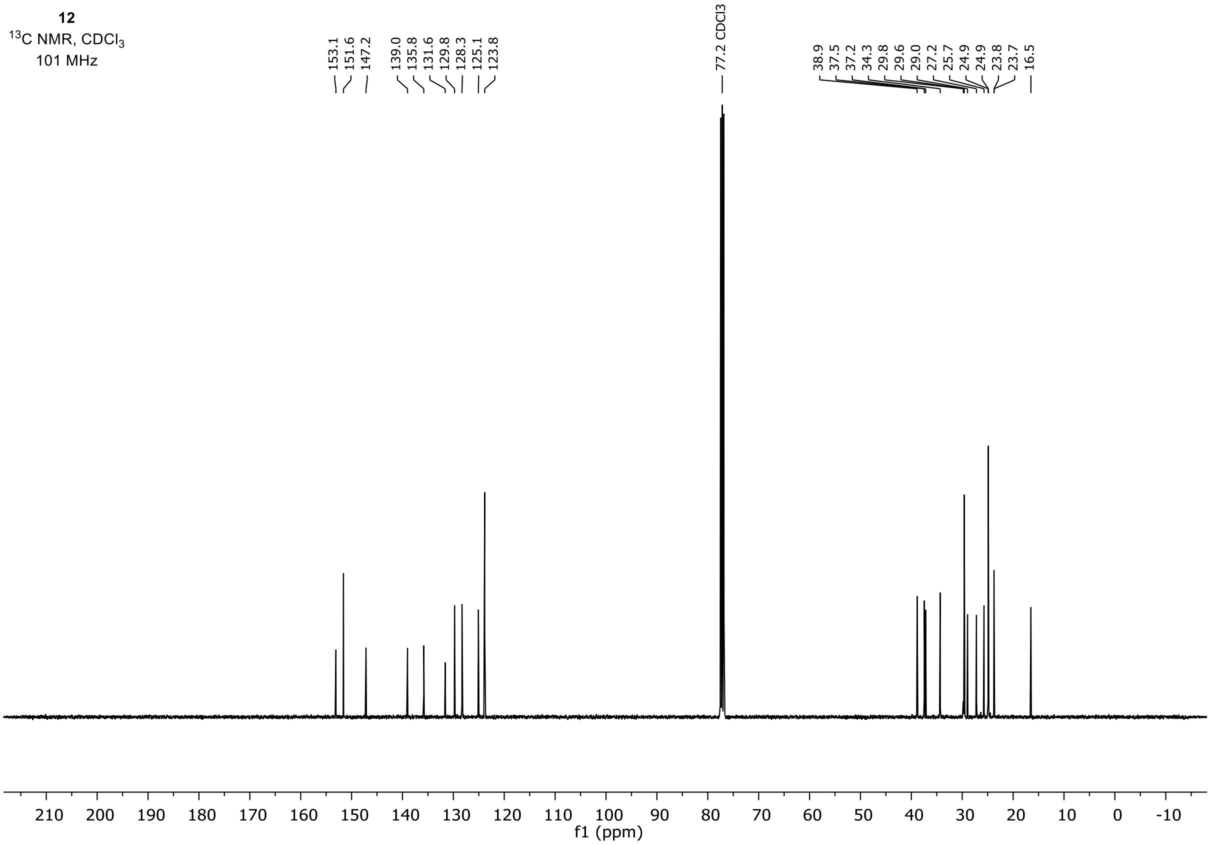

Figure 25. ^{13}C-NMR (101 MHz, $CDCl_3$) of tetralin **12**.

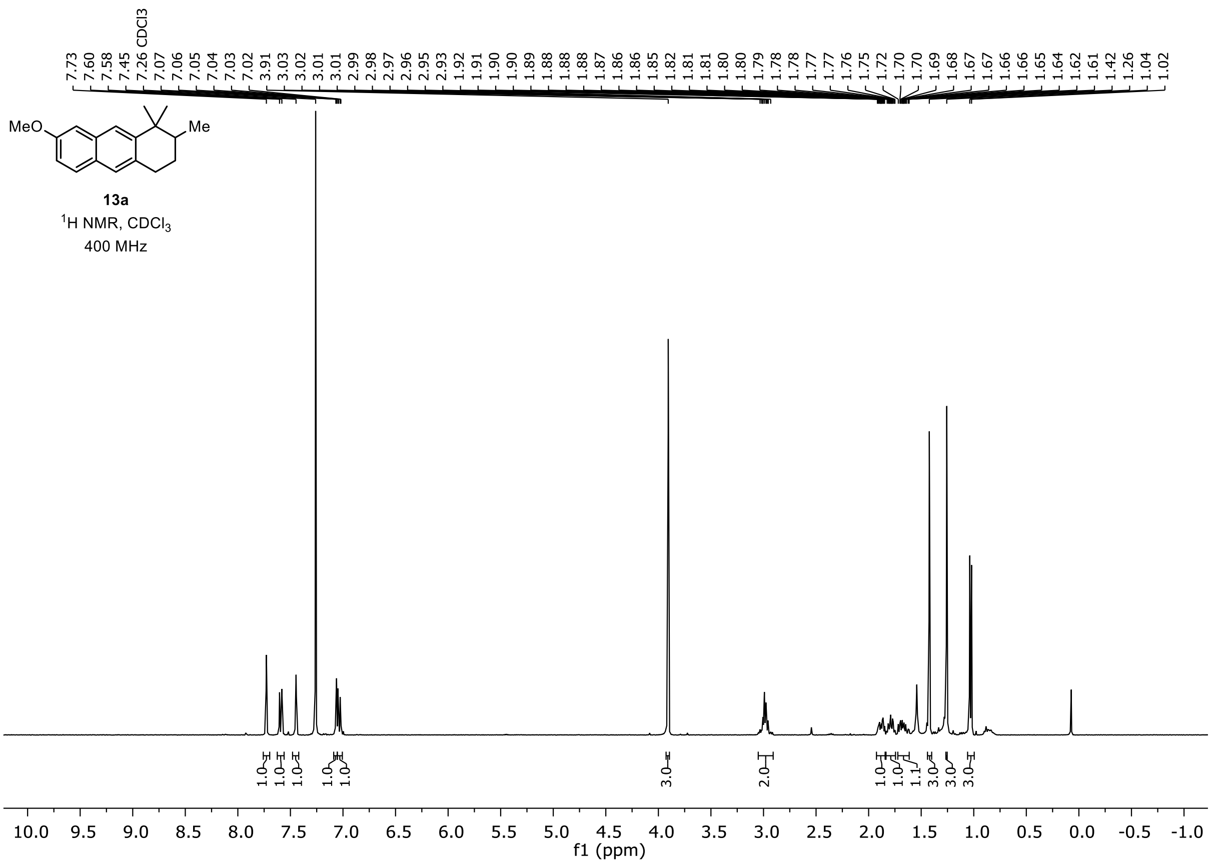

Figure 26. ^{1}H-NMR (400 MHz, $CDCl_3$) of tetrahydroanthracene **13a**.

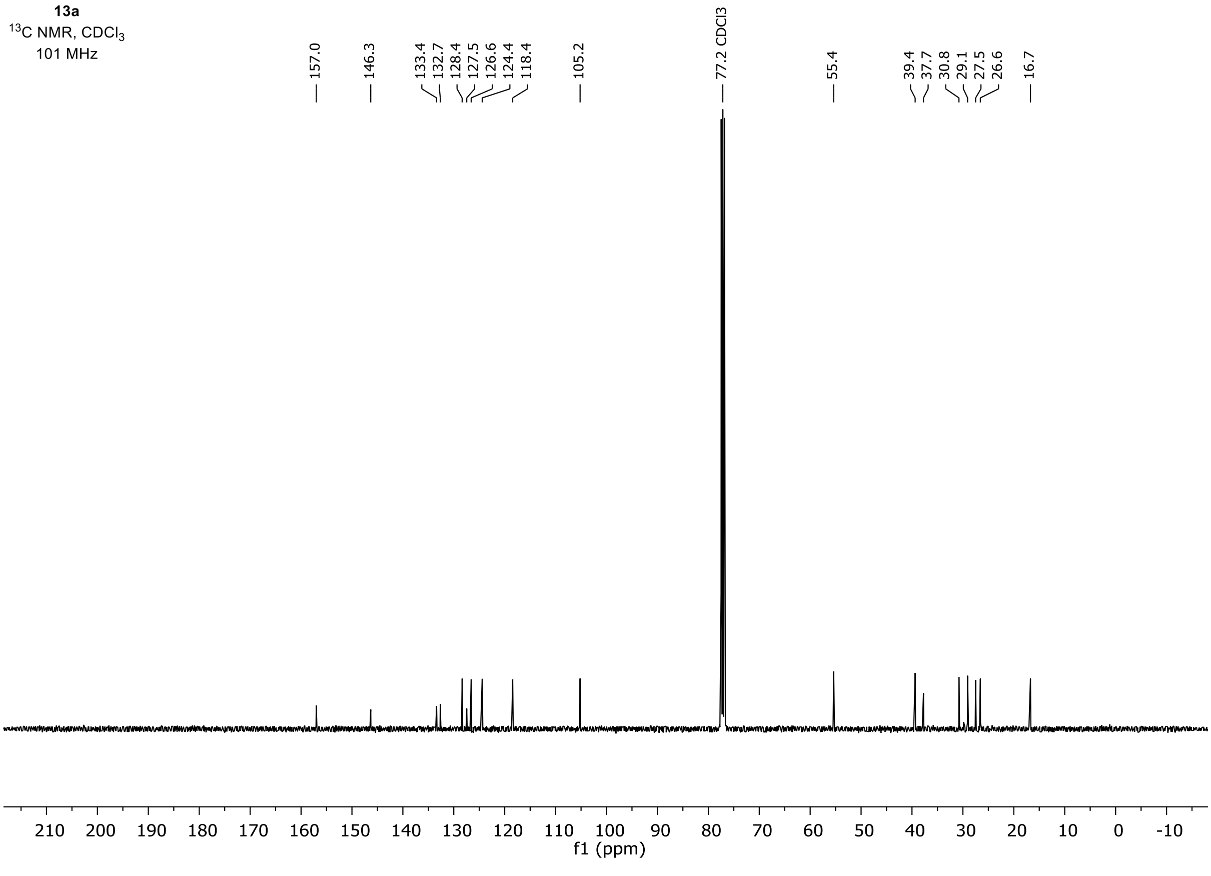

Figure 27. ^{13}C-NMR (101 MHz, $CDCl_3$) of tetrahydroanthracene **13a**.

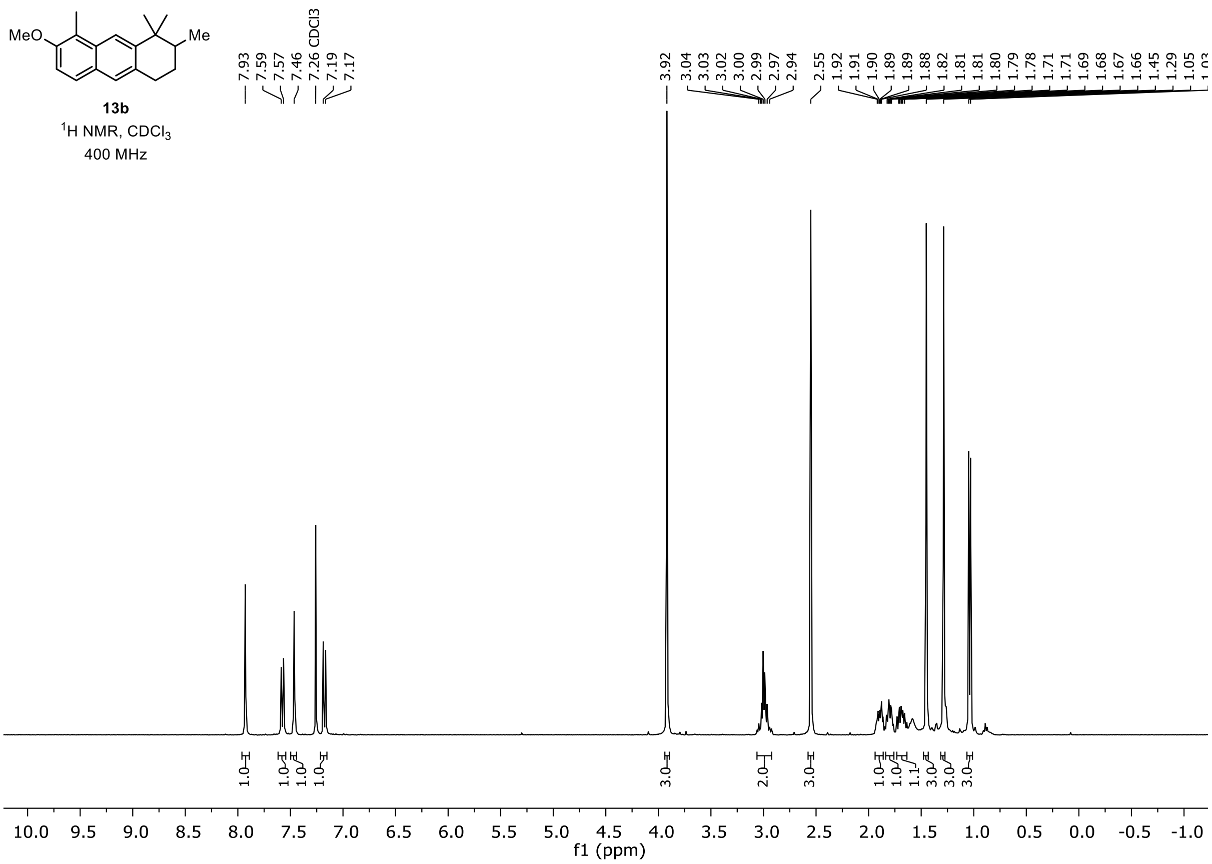

Figure 28. ^{1}H-NMR (400 MHz, $CDCl_3$) of tetrahydroanthracene **13b**.

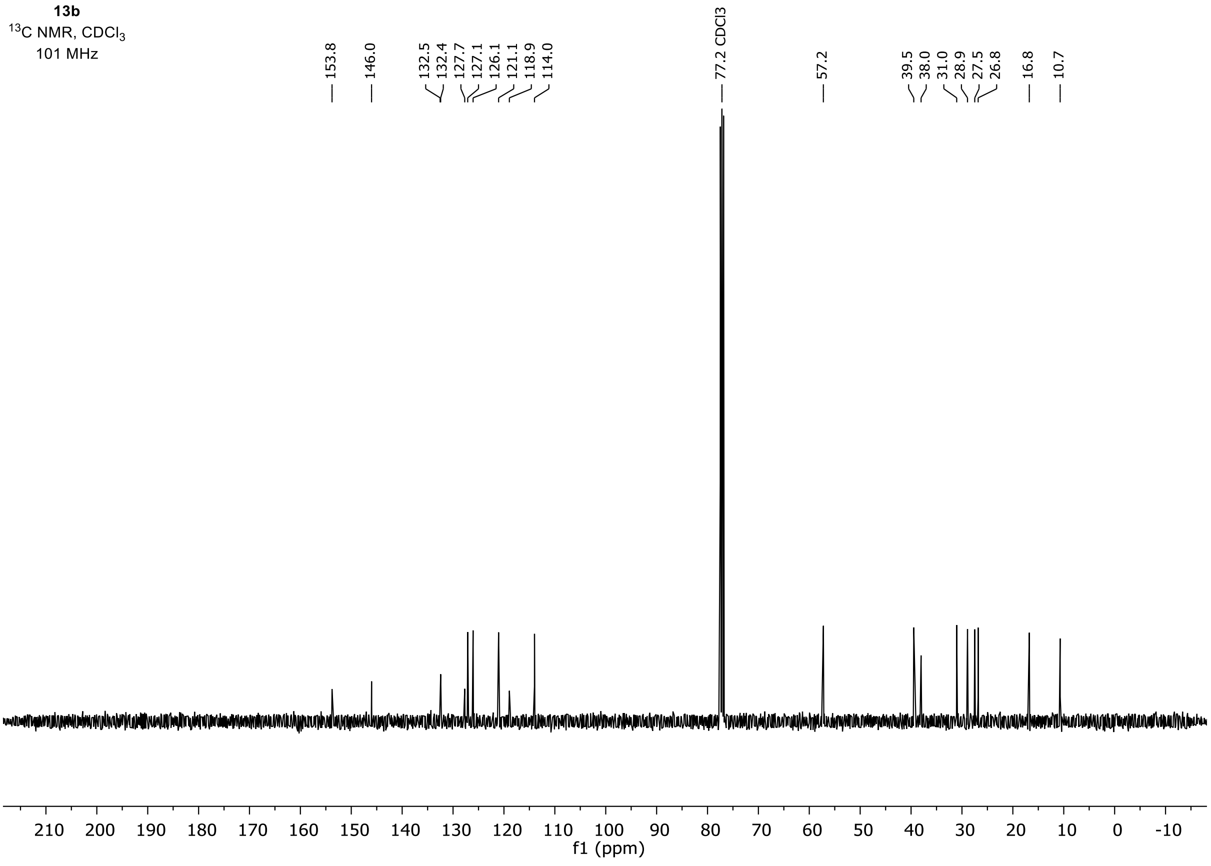

Figure 29. ^{13}C-NMR (101 MHz, $CDCl_3$) of tetrahydroanthracene **13b**.

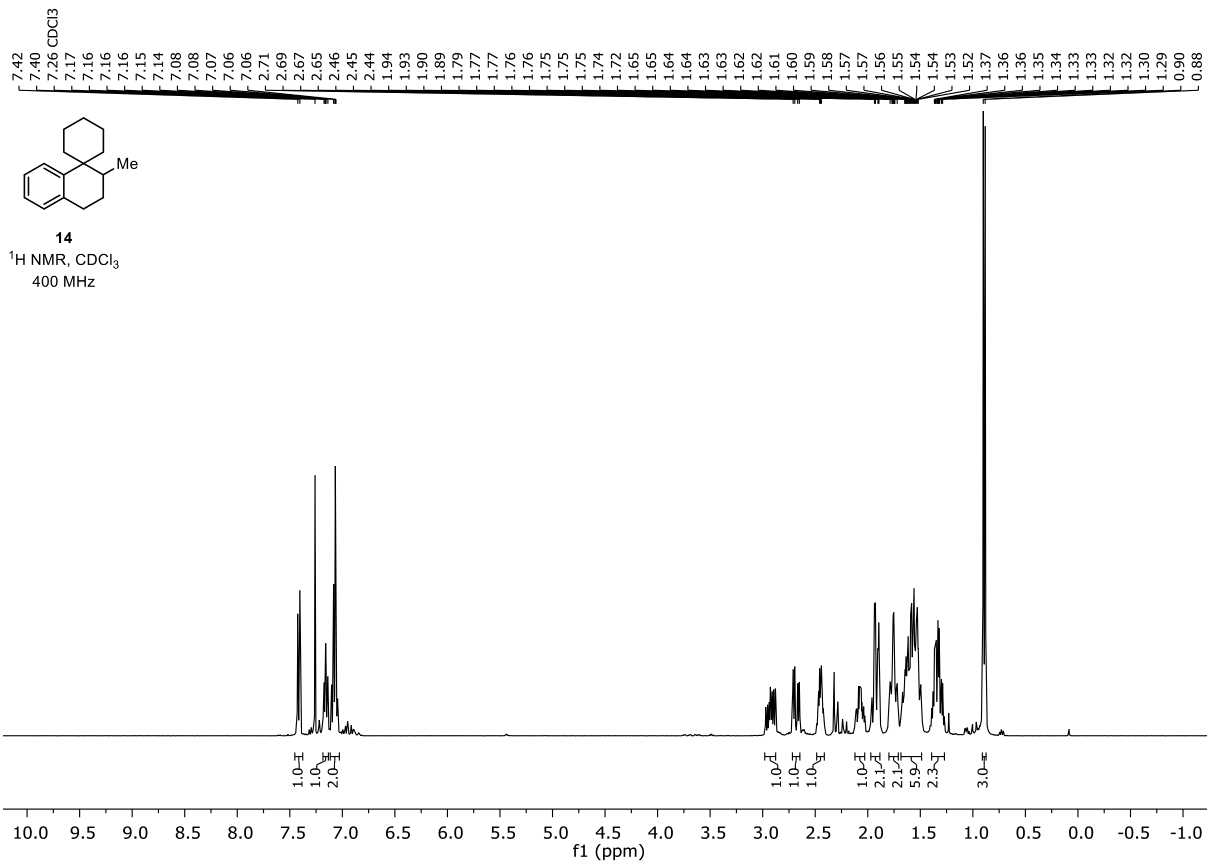

Figure 30. ^{1}H-NMR (400 MHz, $CDCl_3$) of spirocycle **14**.

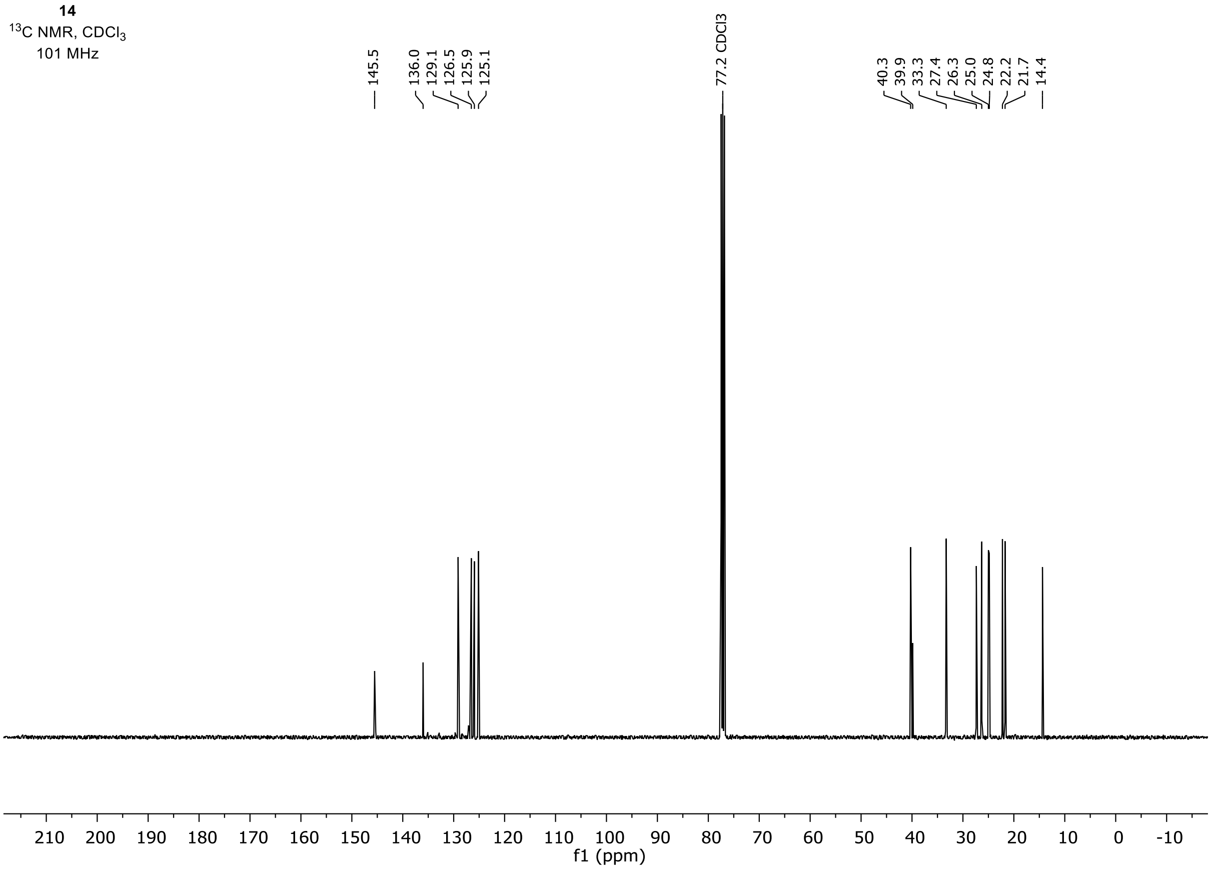

Figure 31. ^{13}C-NMR (101 MHz, $CDCl_3$) of spirocycle **14**.

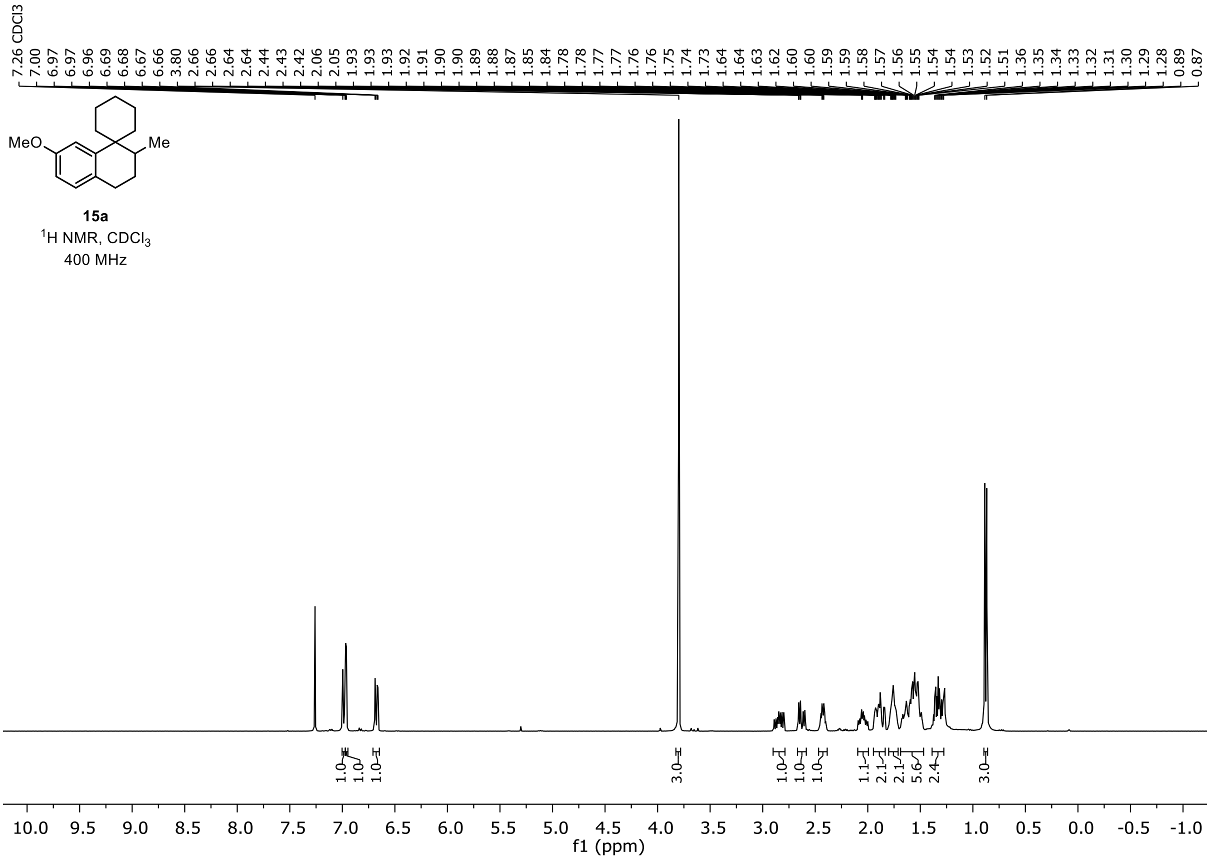

Figure 32. ^{1}H-NMR (400 MHz, $CDCl_3$) of spirocycle **15a**.

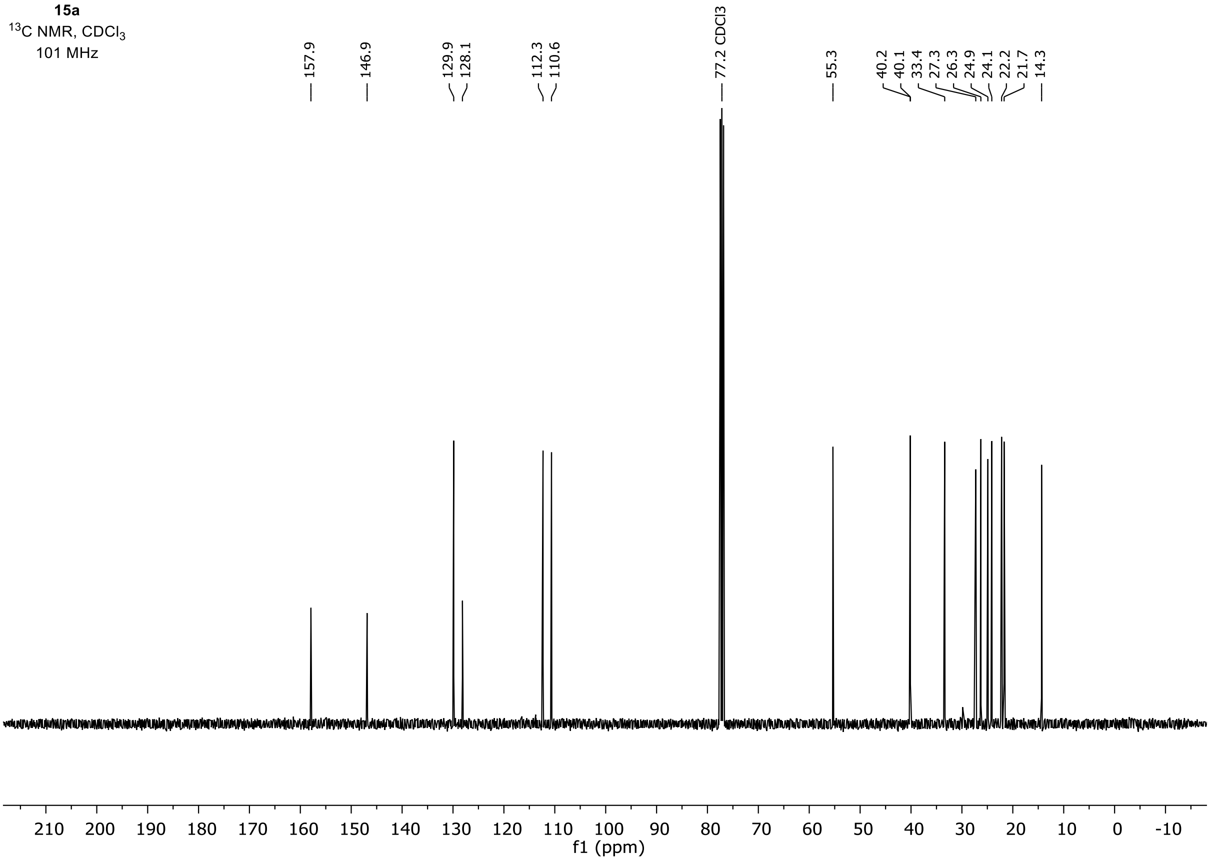

Figure 33. ^{13}C-NMR (101 MHz, $CDCl_3$) of spirocycle **15a**.

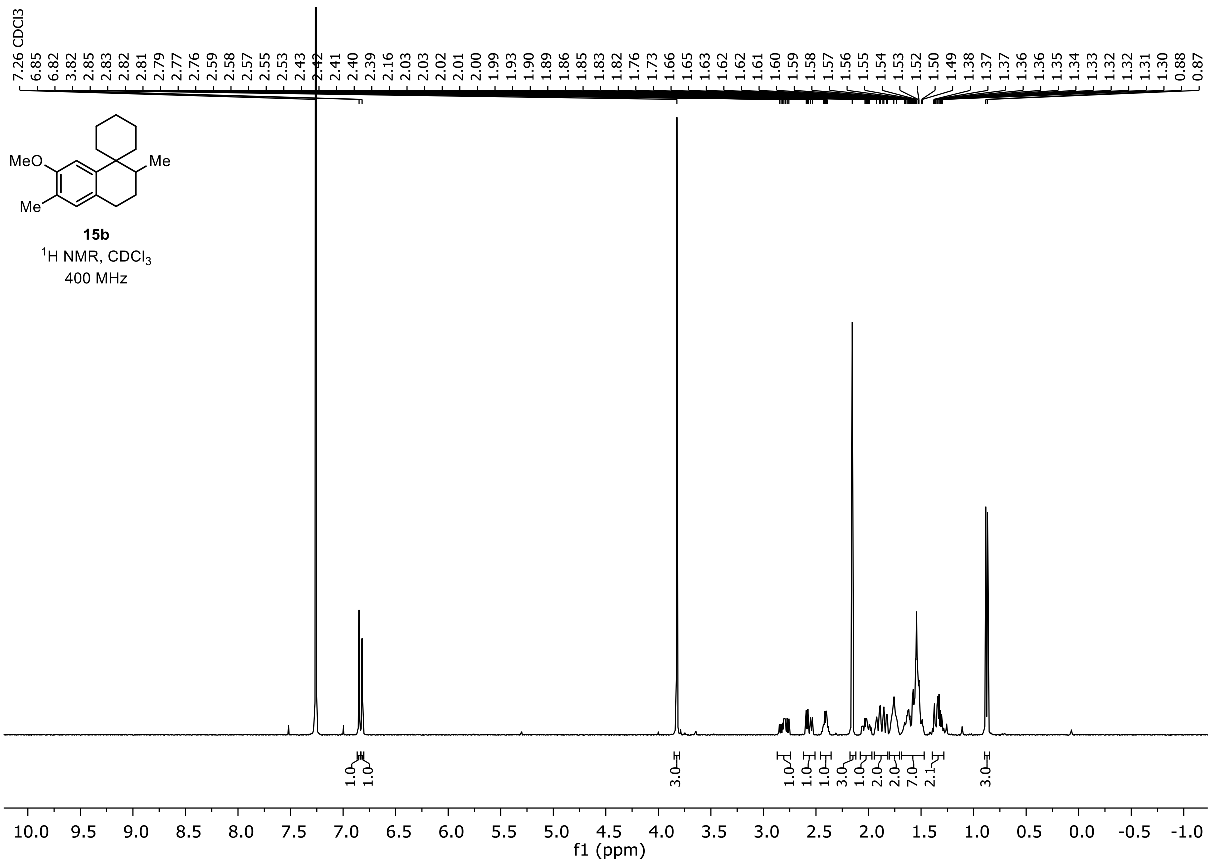

Figure 34. ^{1}H-NMR (400 MHz, $CDCl_3$) of spirocycle **15b**.

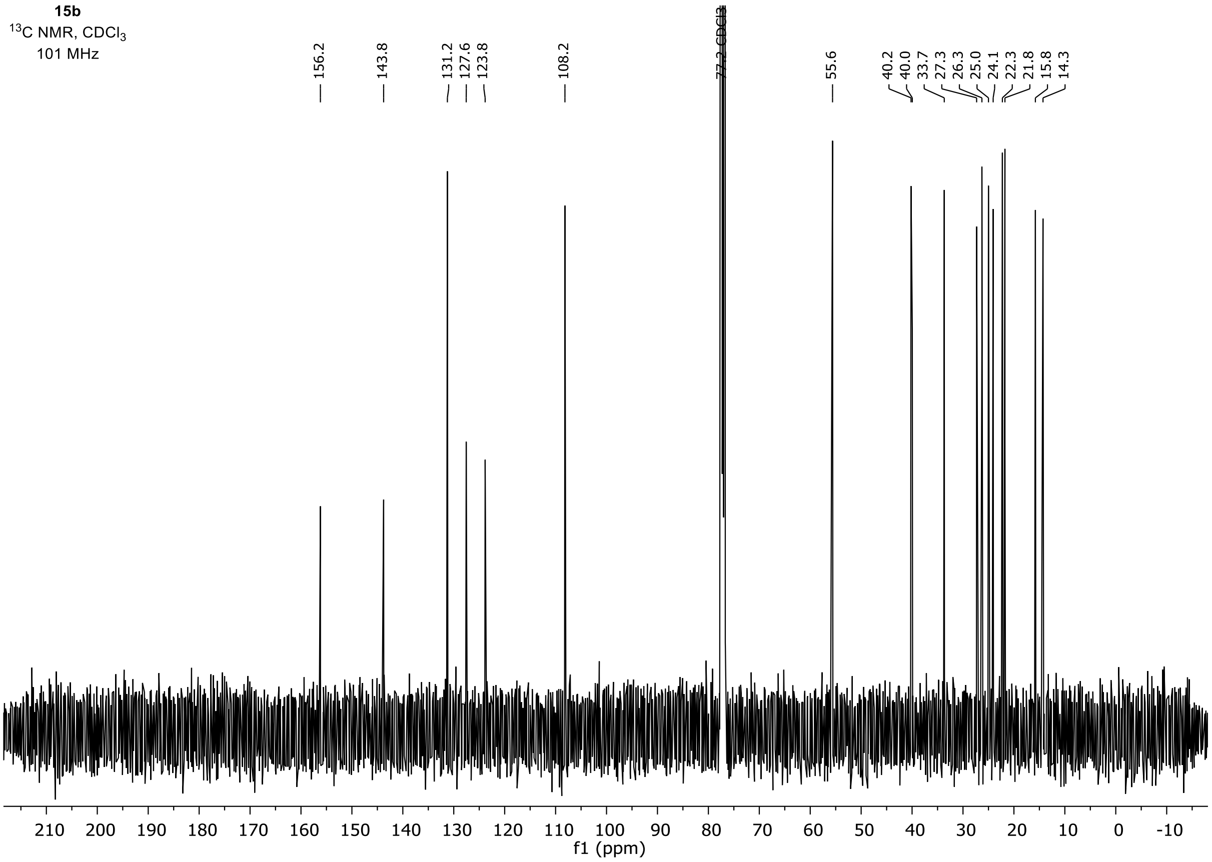

Figure 35. ^{13}C-NMR (101 MHz, $CDCl_3$) of spirocycle **15b**.

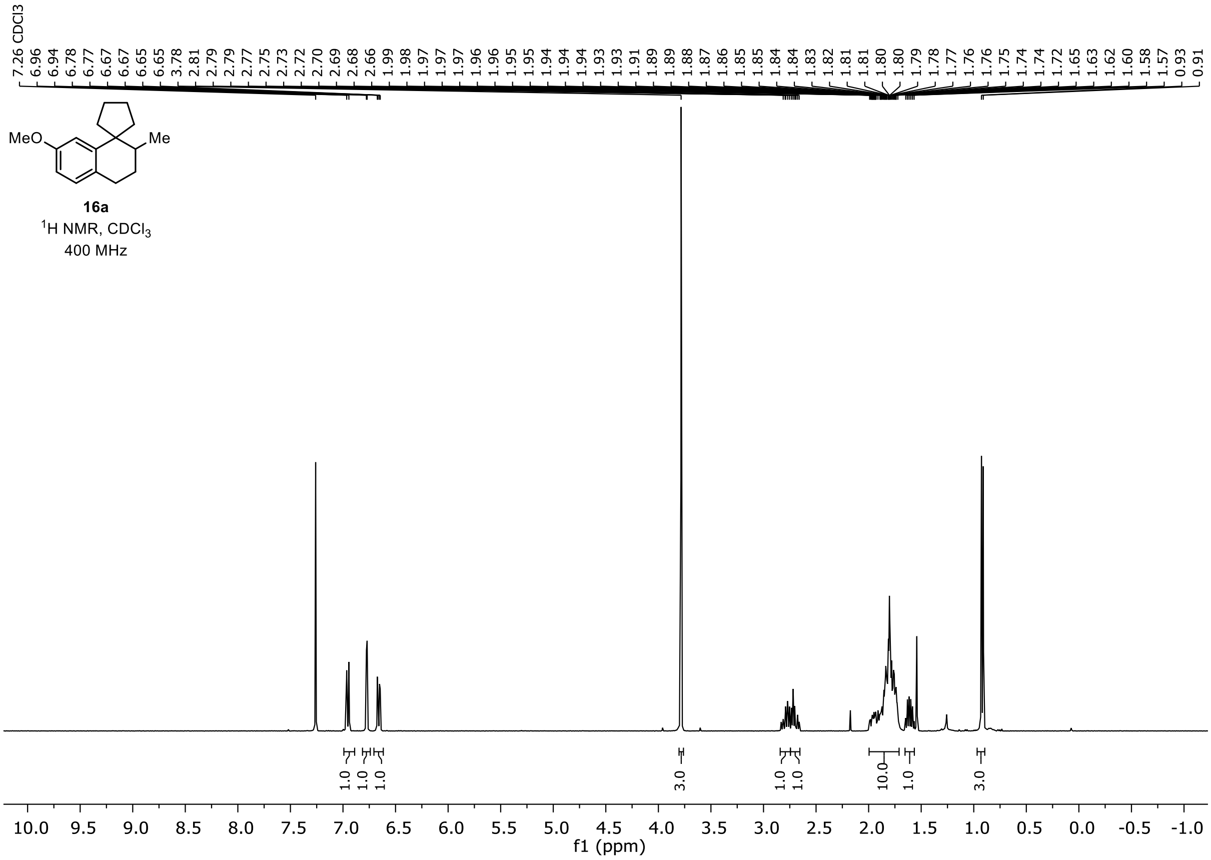

Figure 36. ^{1}H-NMR (400 MHz, $CDCl_3$) of spirocycle **16a**.

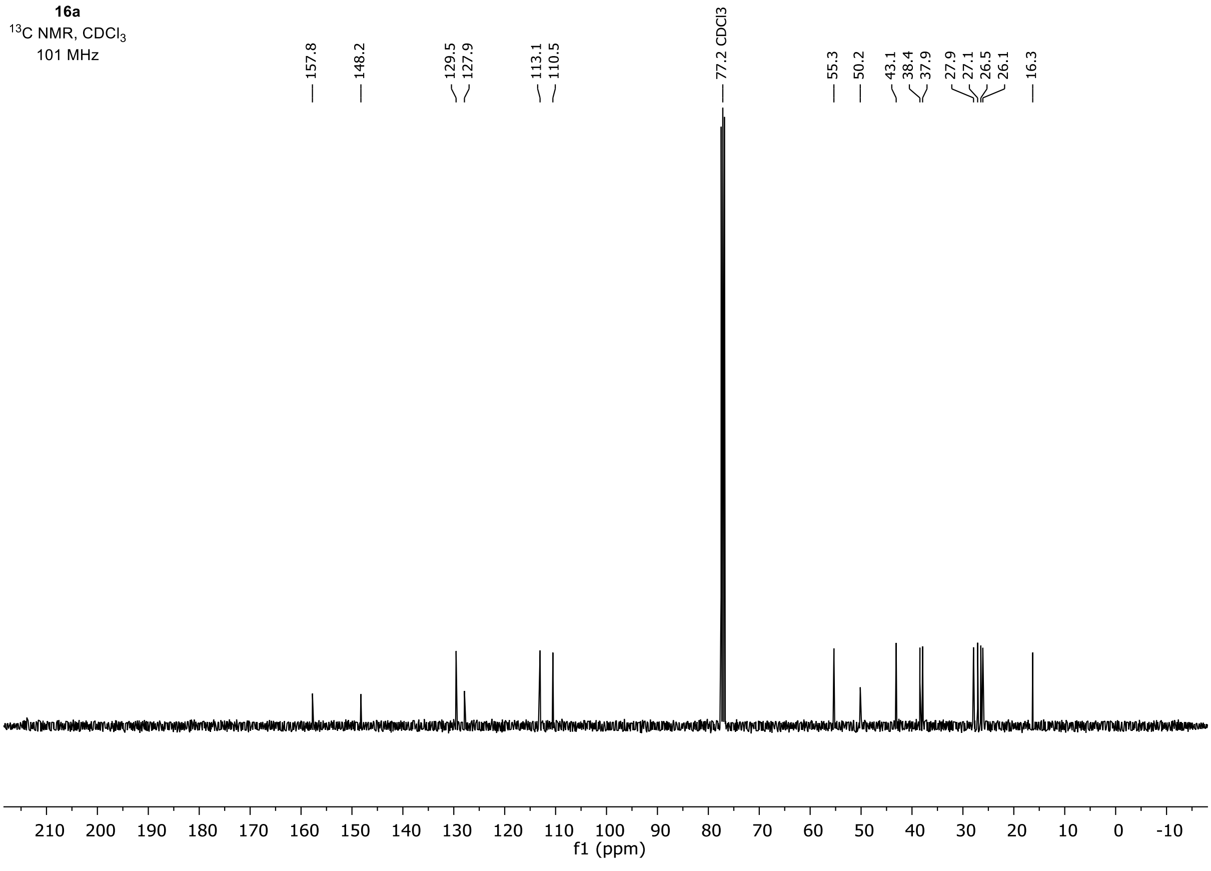

Figure 37. ^{13}C-NMR (101 MHz, $CDCl_3$) of spirocycle **16a**.

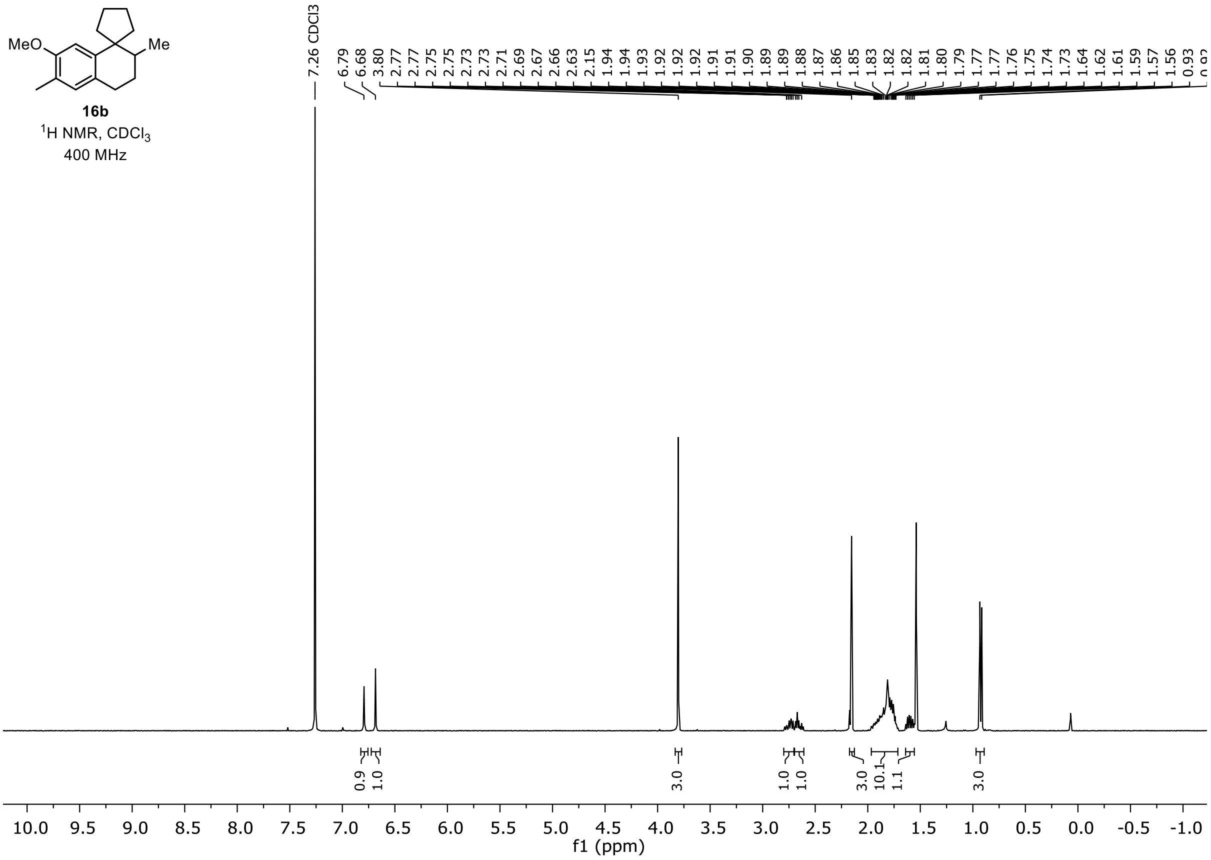

Figure 38. ^{1}H-NMR (400 MHz, $CDCl_3$) of spirocycle **16b**.

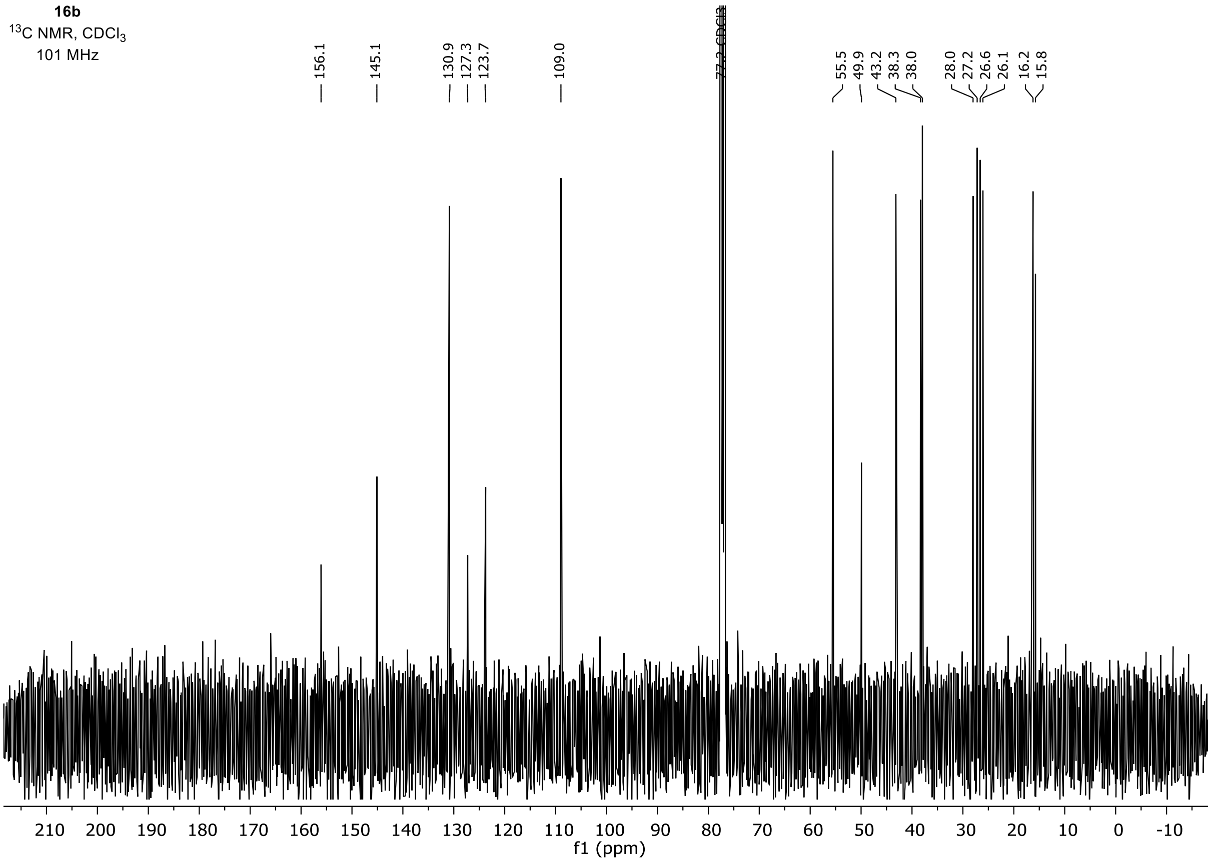

Figure 39. ^{13}C-NMR (101 MHz, $CDCl_3$) of spirocycle **16b**.

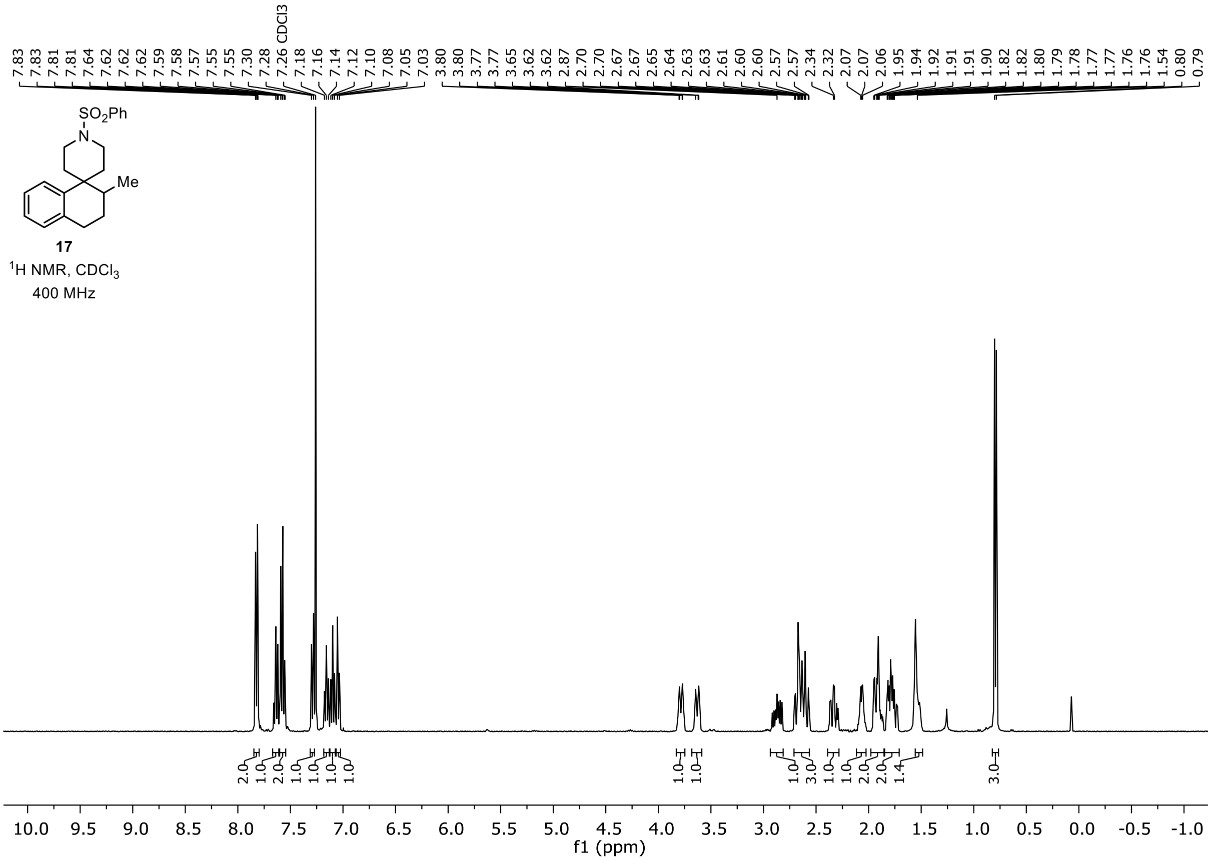

Figure 40. ^{1}H-NMR (400 MHz, $CDCl_3$) of piperidine **17**.

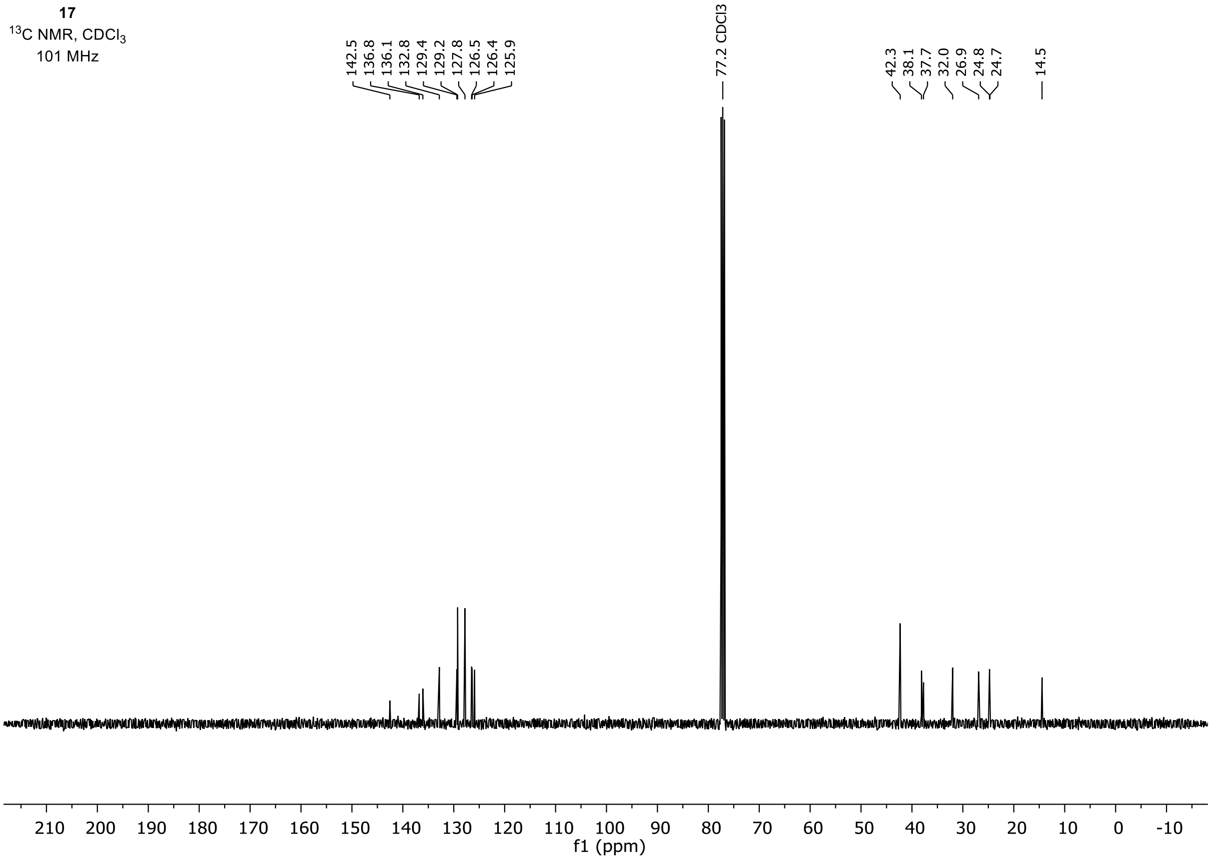

Figure 41. ^{13}C-NMR (101 MHz, $CDCl_3$) of piperidine **17**.

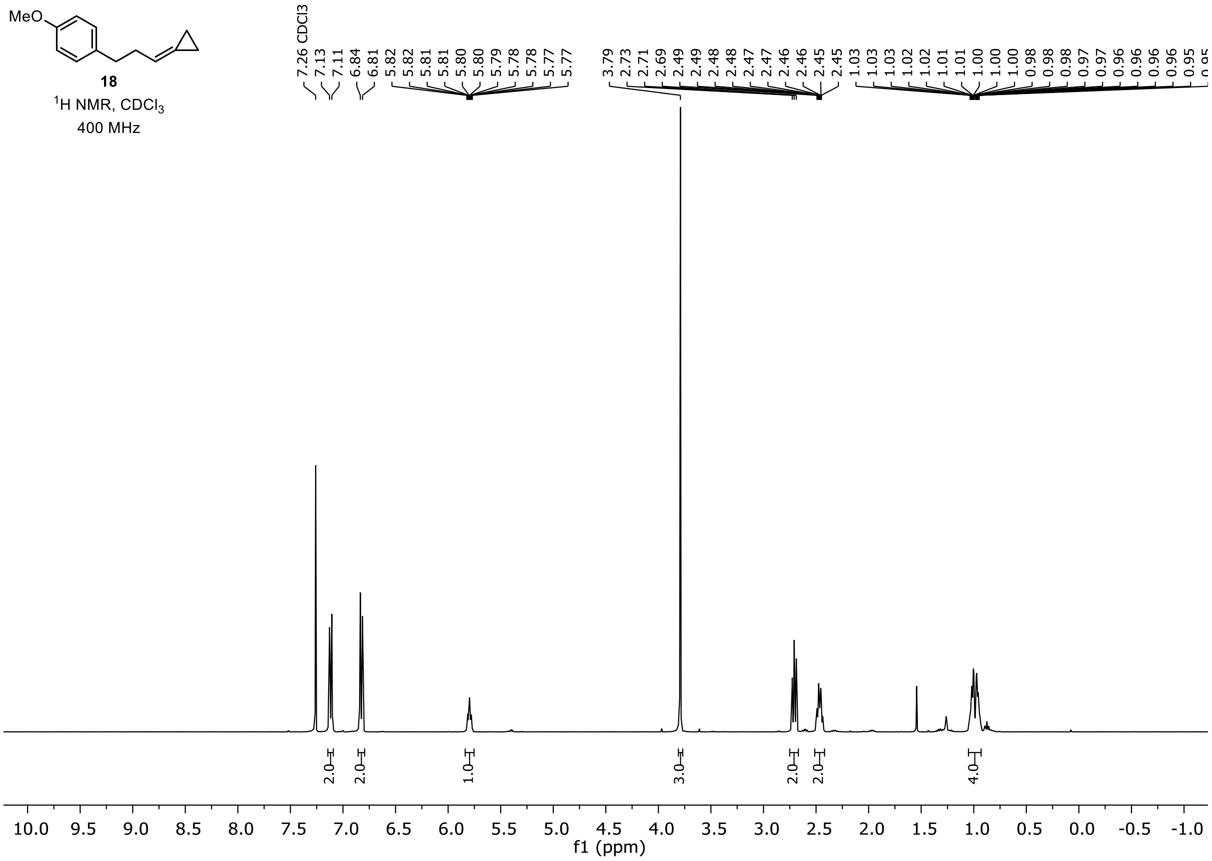

Figure 42. ^{1}H-NMR (400 MHz, $CDCl_3$) of alkene **18**.

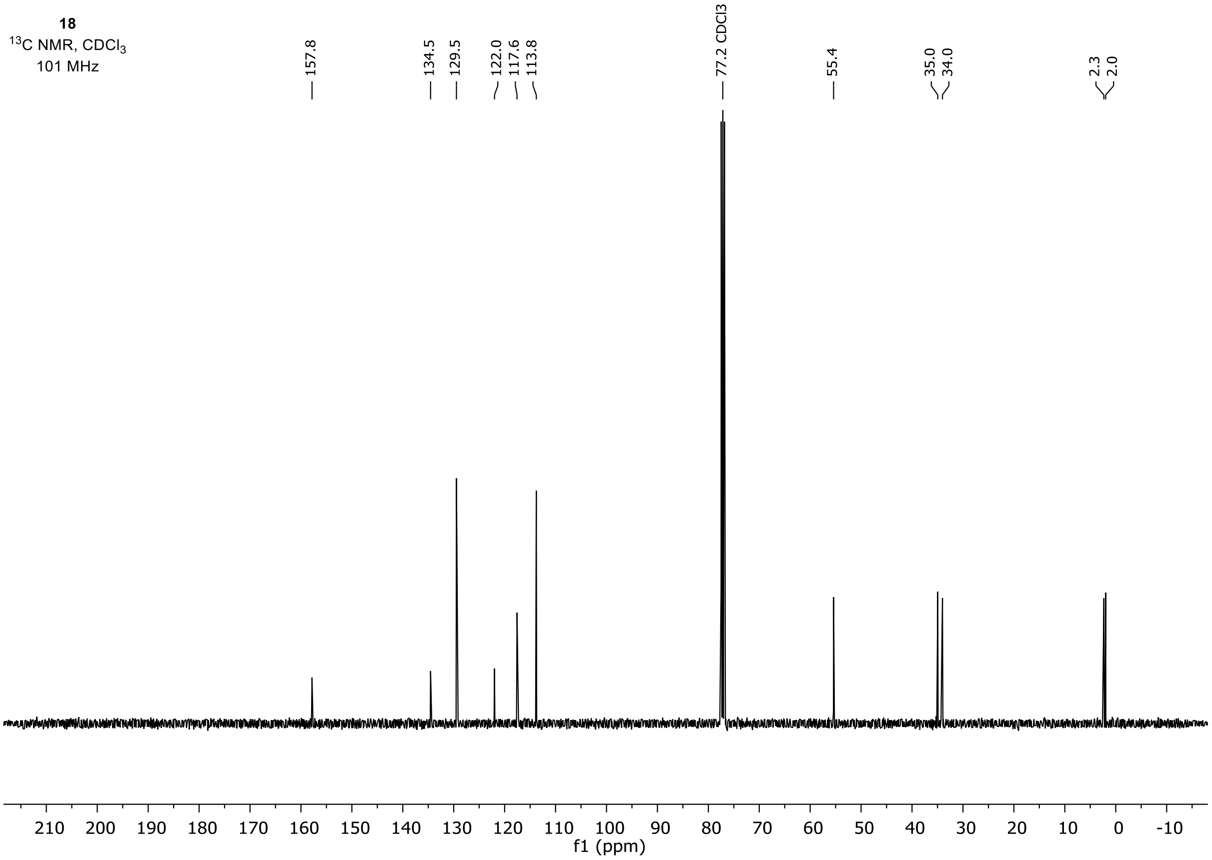

Figure 43. ^{13}C-NMR (101 MHz, $CDCl_3$) of alkene **18**.

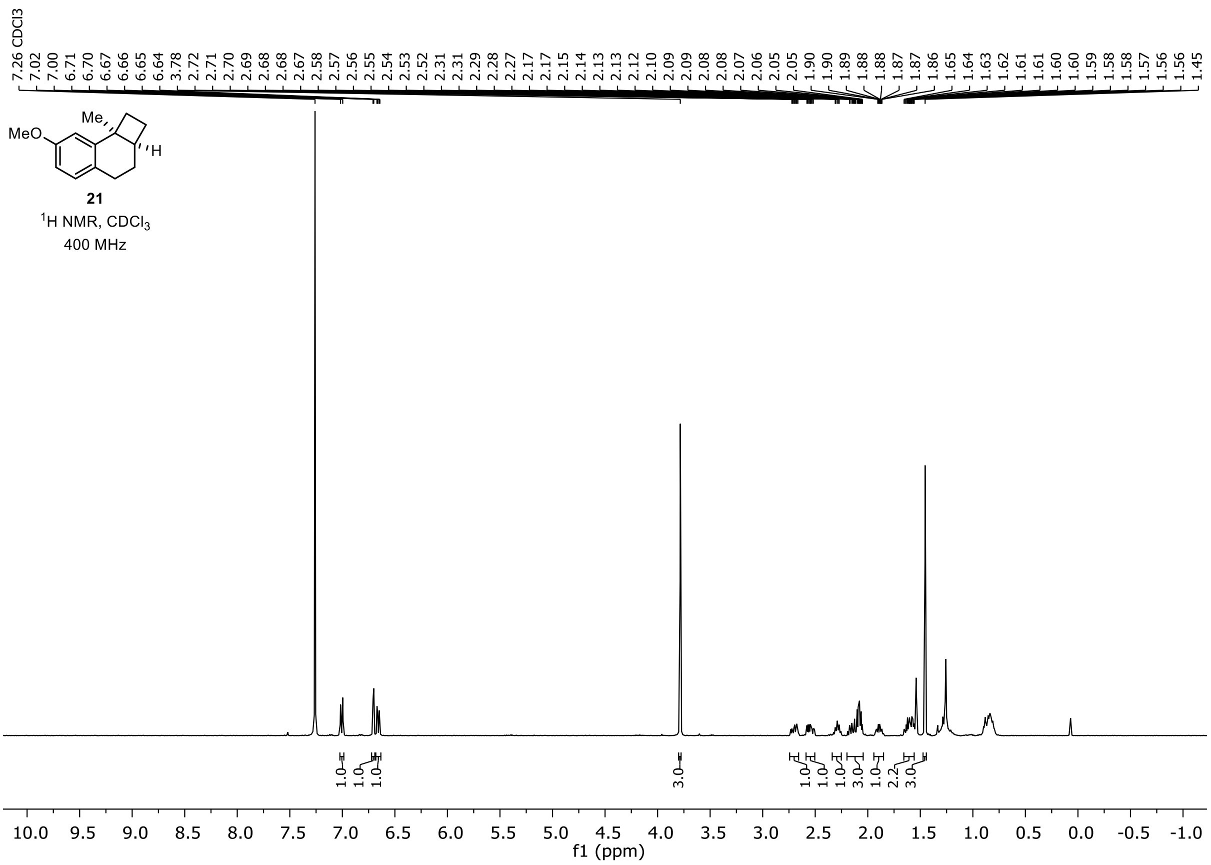

Figure 44. ^{1}H-NMR (400 MHz, $CDCl_3$) of cyclobutane **21**.

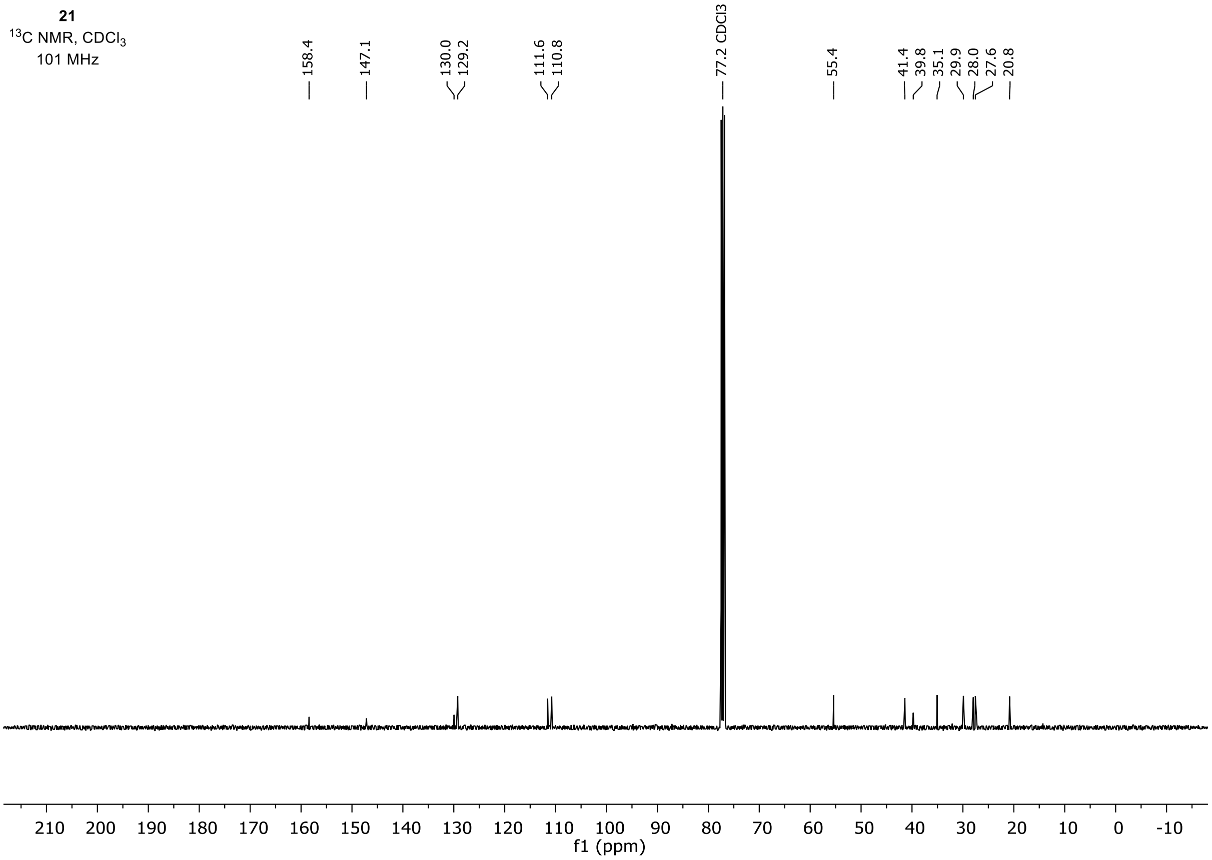

Figure 45. ^{13}C-NMR (101 MHz, $CDCl_3$) of cyclobutane **21**.

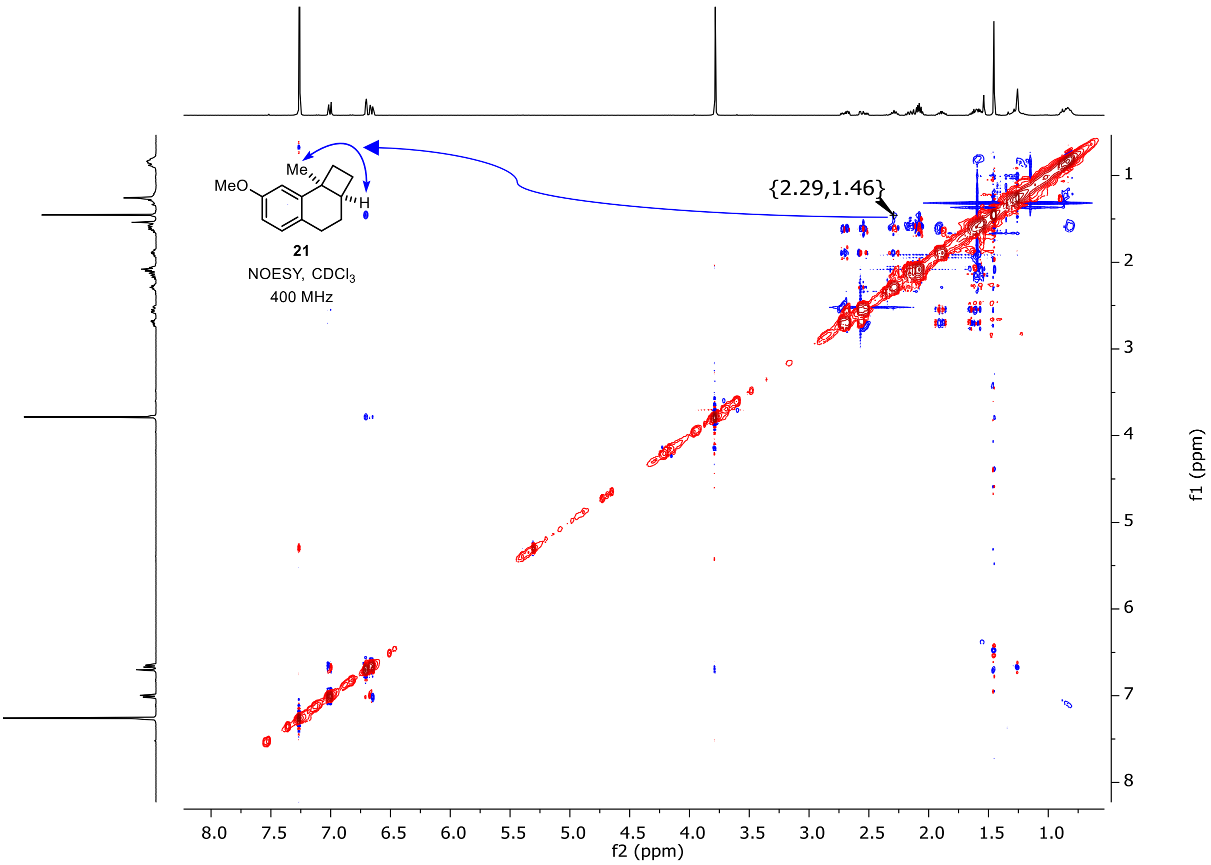

Figure 46. NOESY (400 MHz, $CDCl_3$) of cyclobutane **21**.

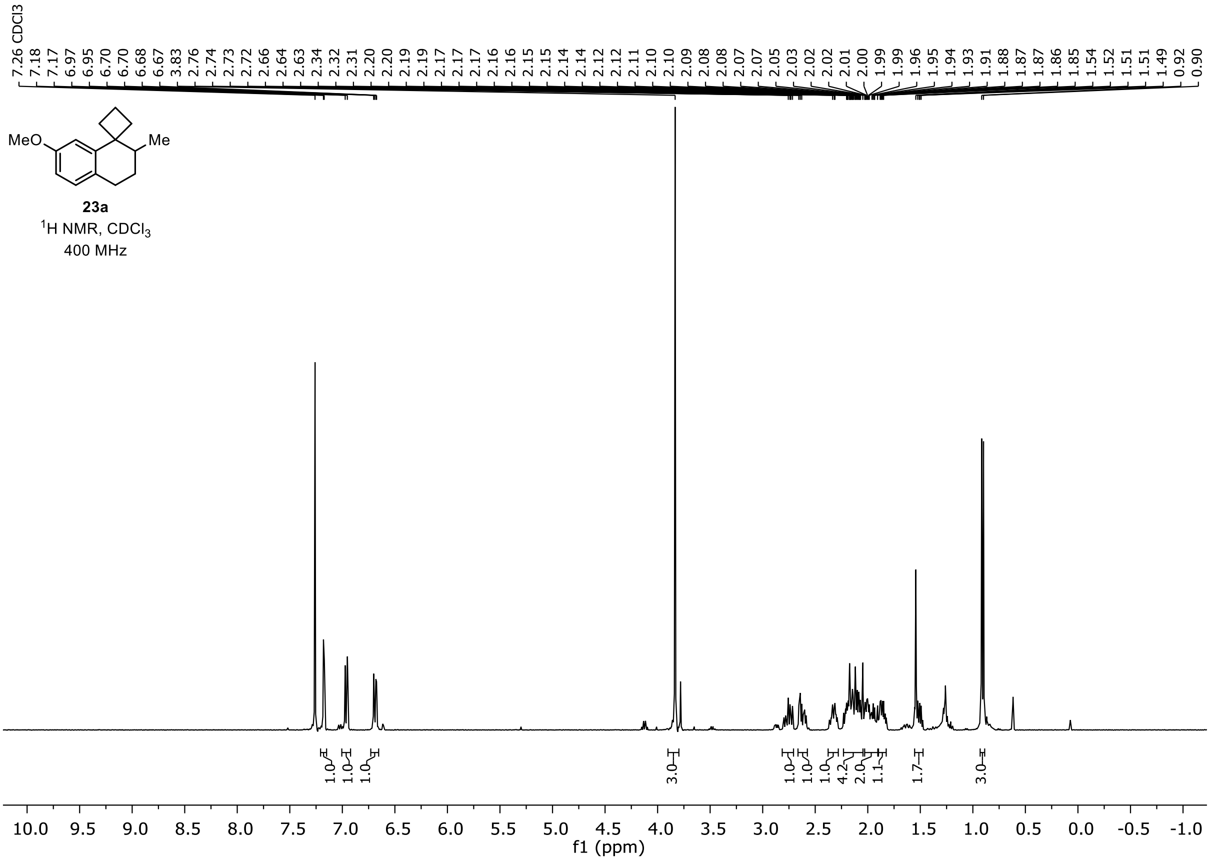

Figure 47. ^{1}H-NMR (400 MHz, $CDCl_3$) of spirocycle **23a**.

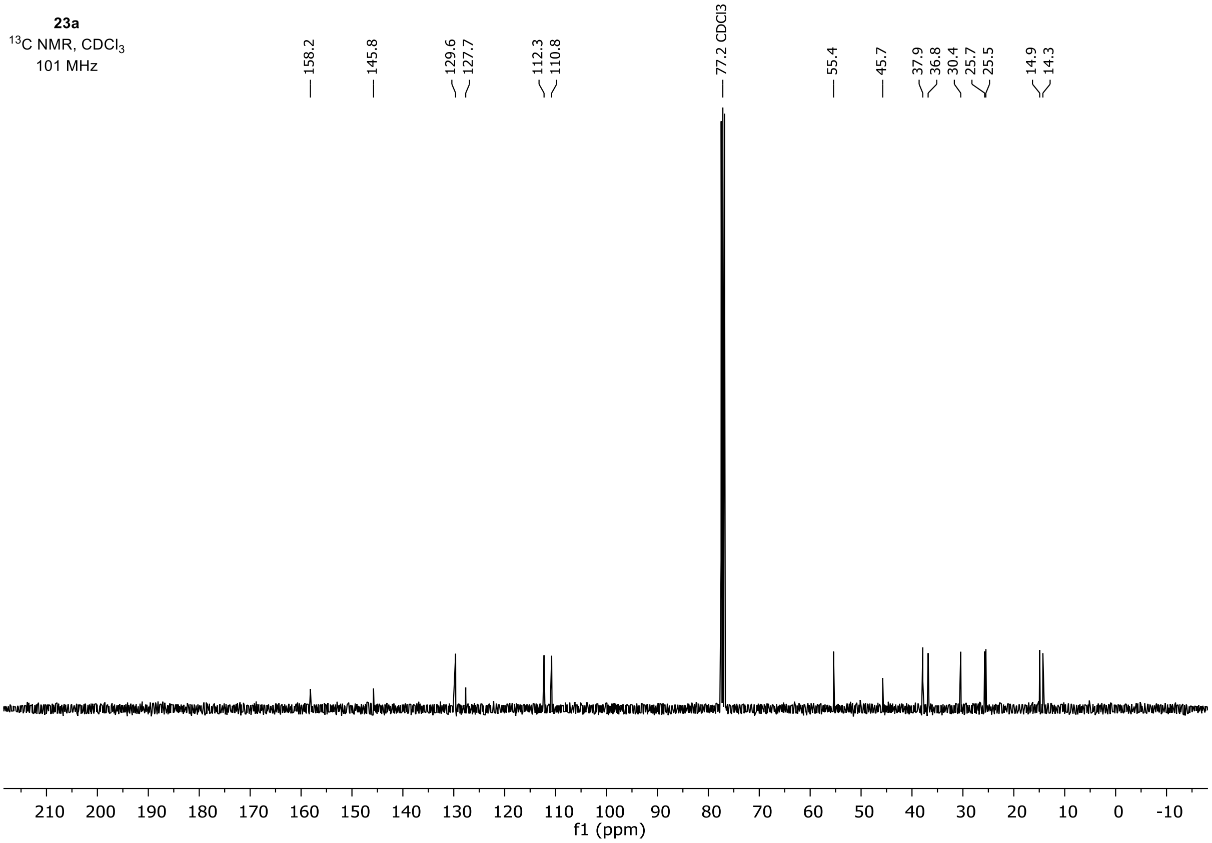

Figure 48. ^{13}C-NMR (101 MHz, $CDCl_3$) of spirocycle **23a**.

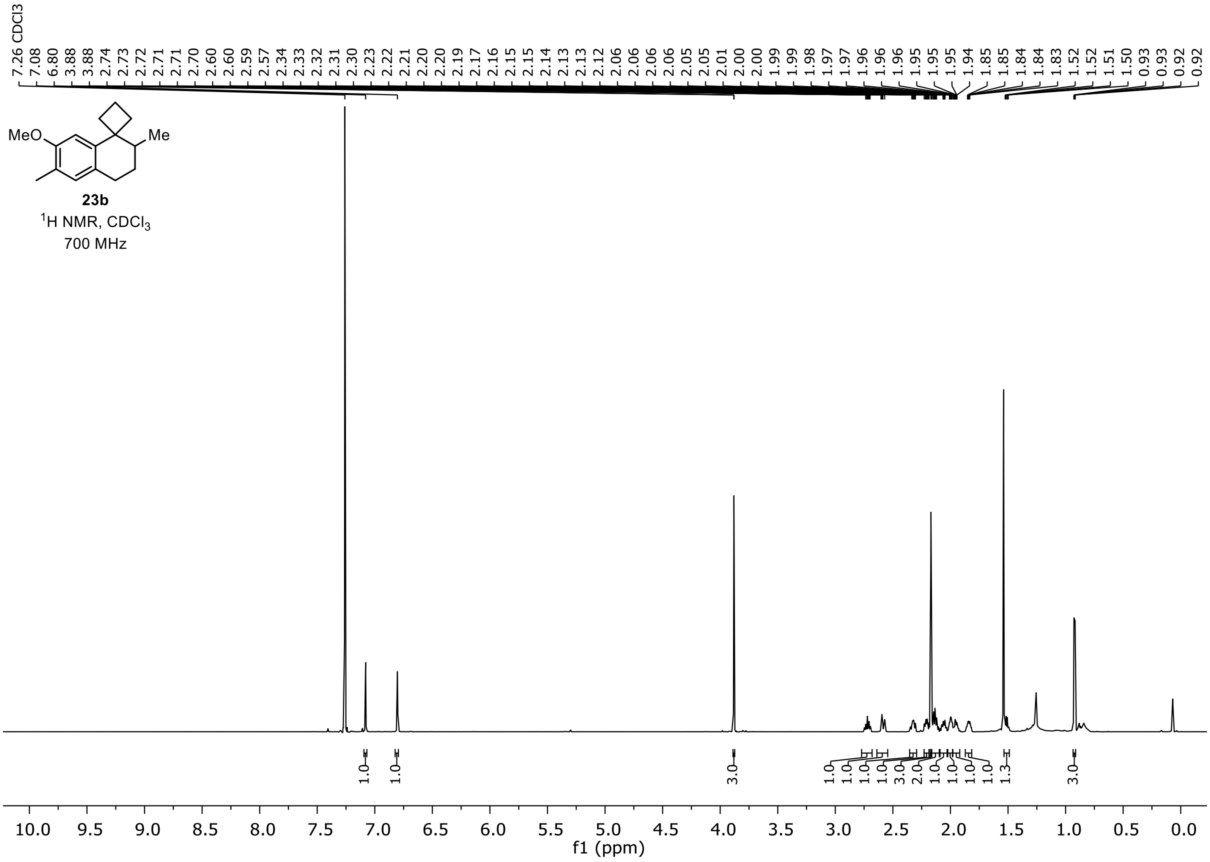

Figure 49. ^{1}H-NMR (700 MHz, $CDCl_3$) of spirocycle **23b**.

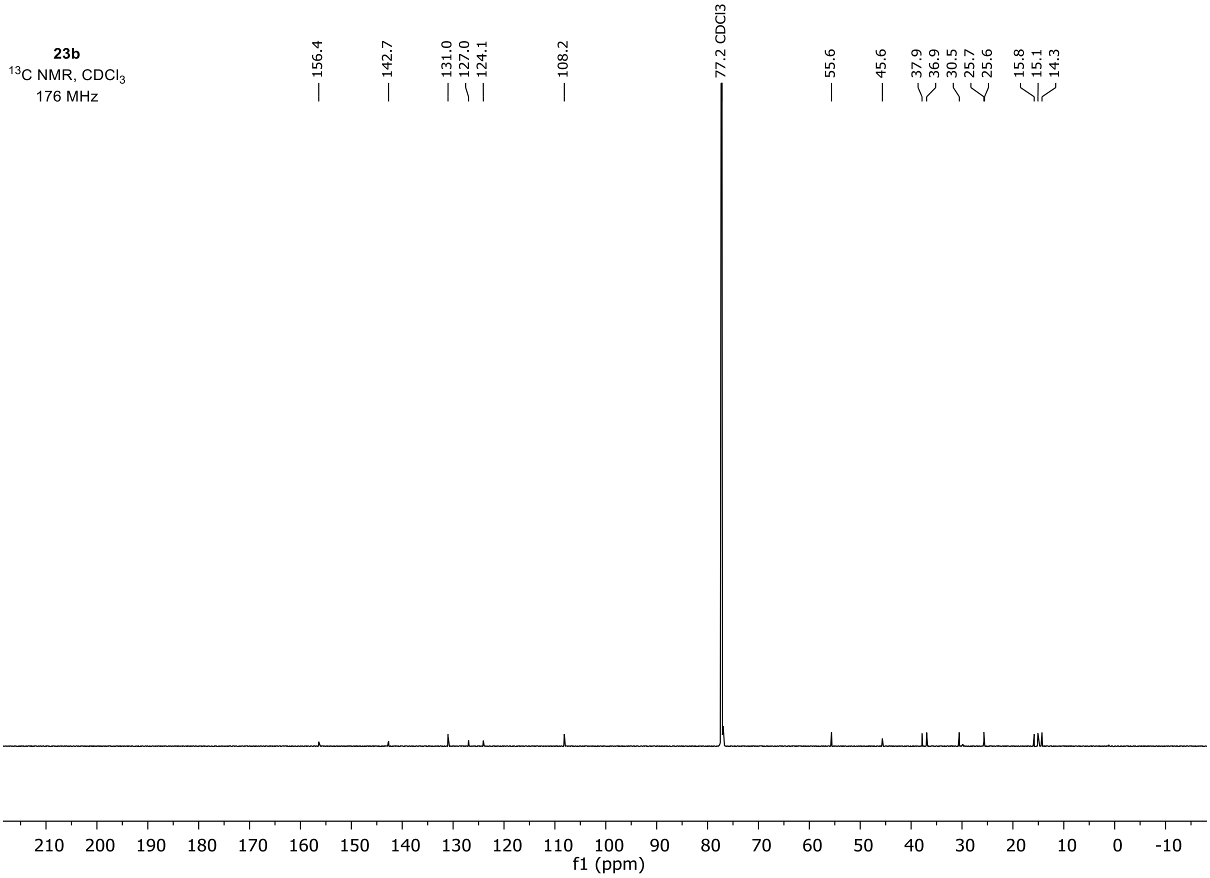

Figure 50. ^{13}C-NMR (176 MHz, $CDCl_3$) of spirocycle **23b**.

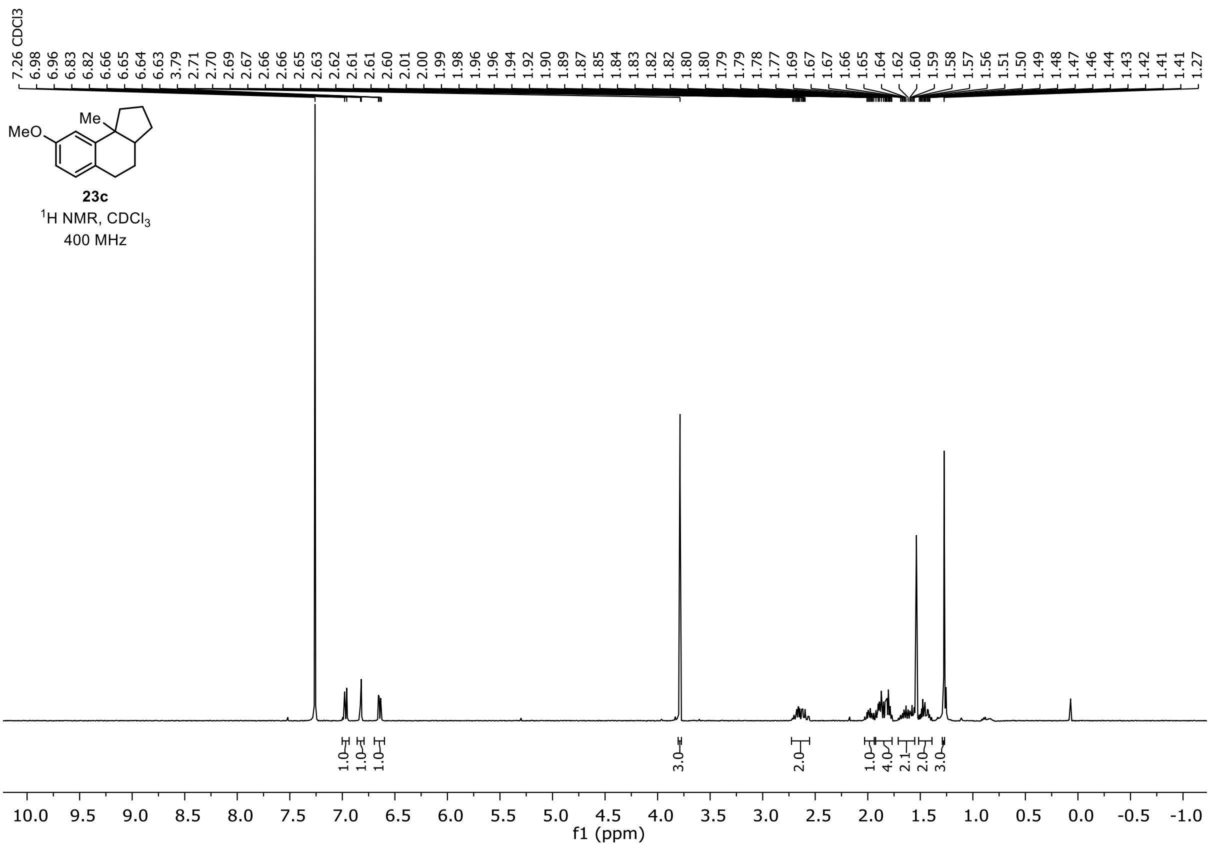

Figure 51. ^{1}H-NMR (400 MHz, $CDCl_3$) of cyclopentane **23c**.

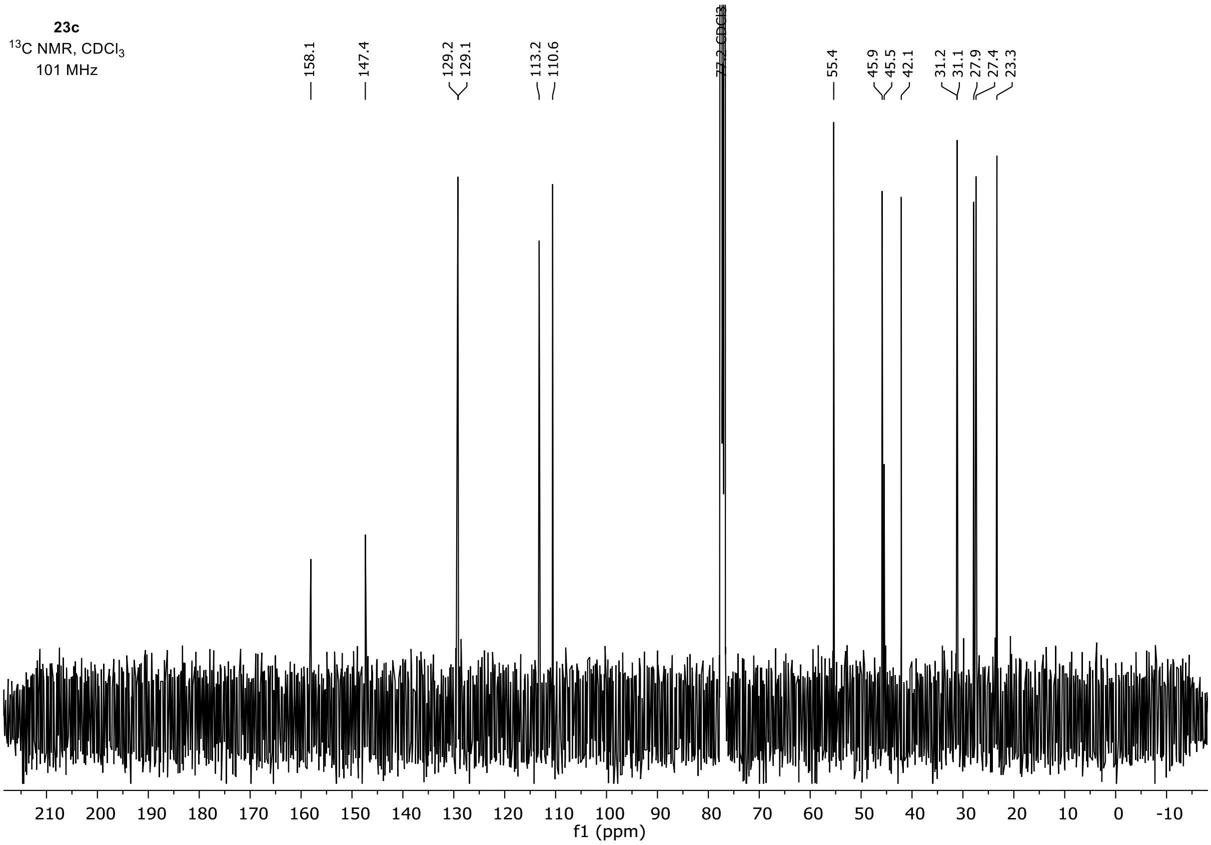

Figure 52. ^{13}C-NMR (101 MHz, $CDCl_3$) of cyclopentane **23c**.

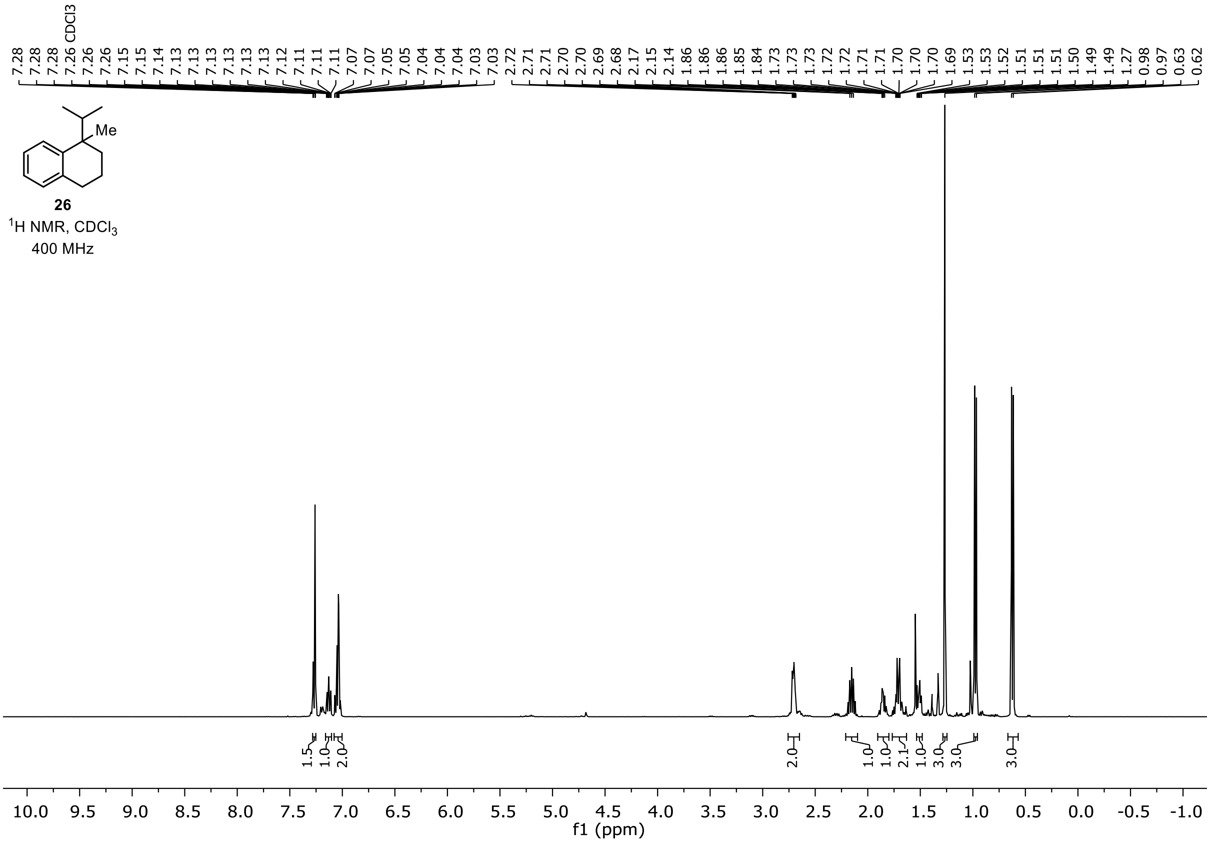

Figure 53. ^{1}H-NMR (400 MHz, $CDCl_3$) of tetralin **26**.

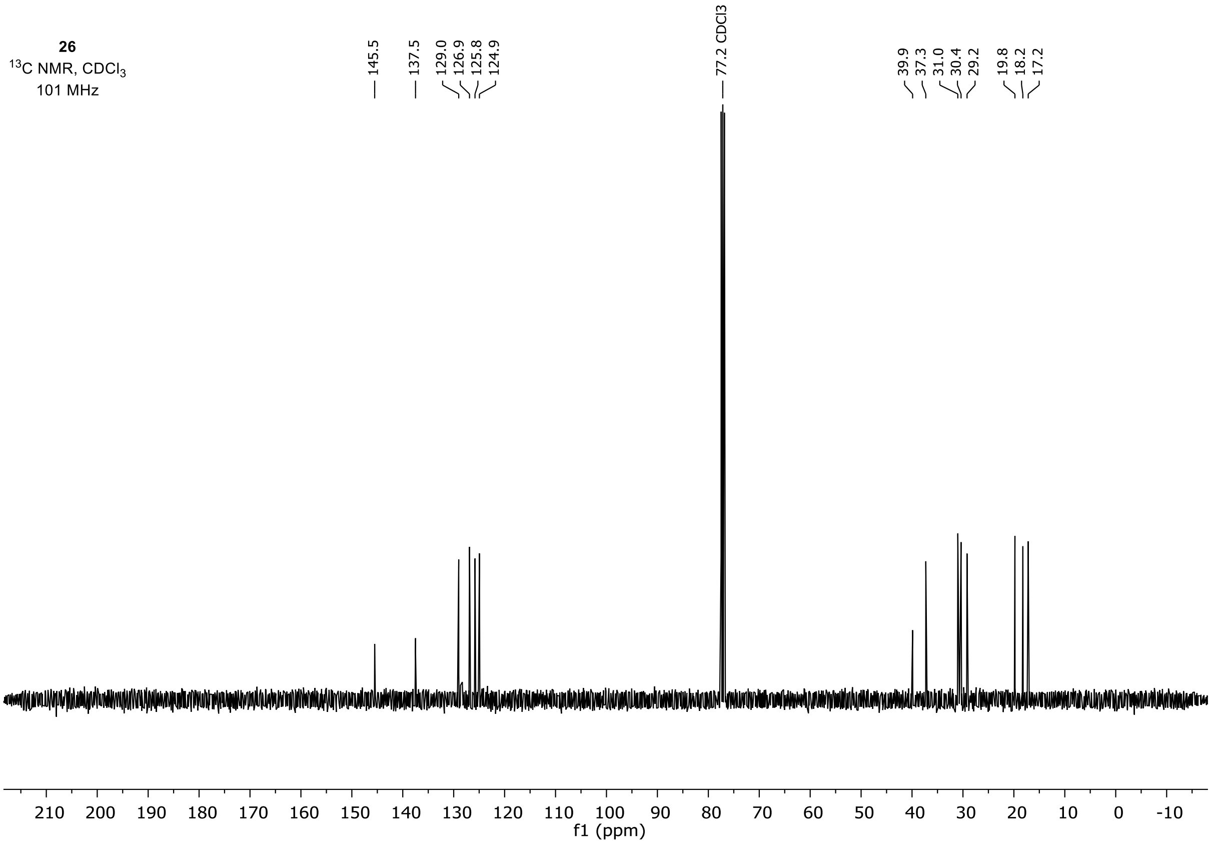

Figure 54. ^{13}C-NMR (101 MHz, $CDCl_3$) of tetralin **26**.

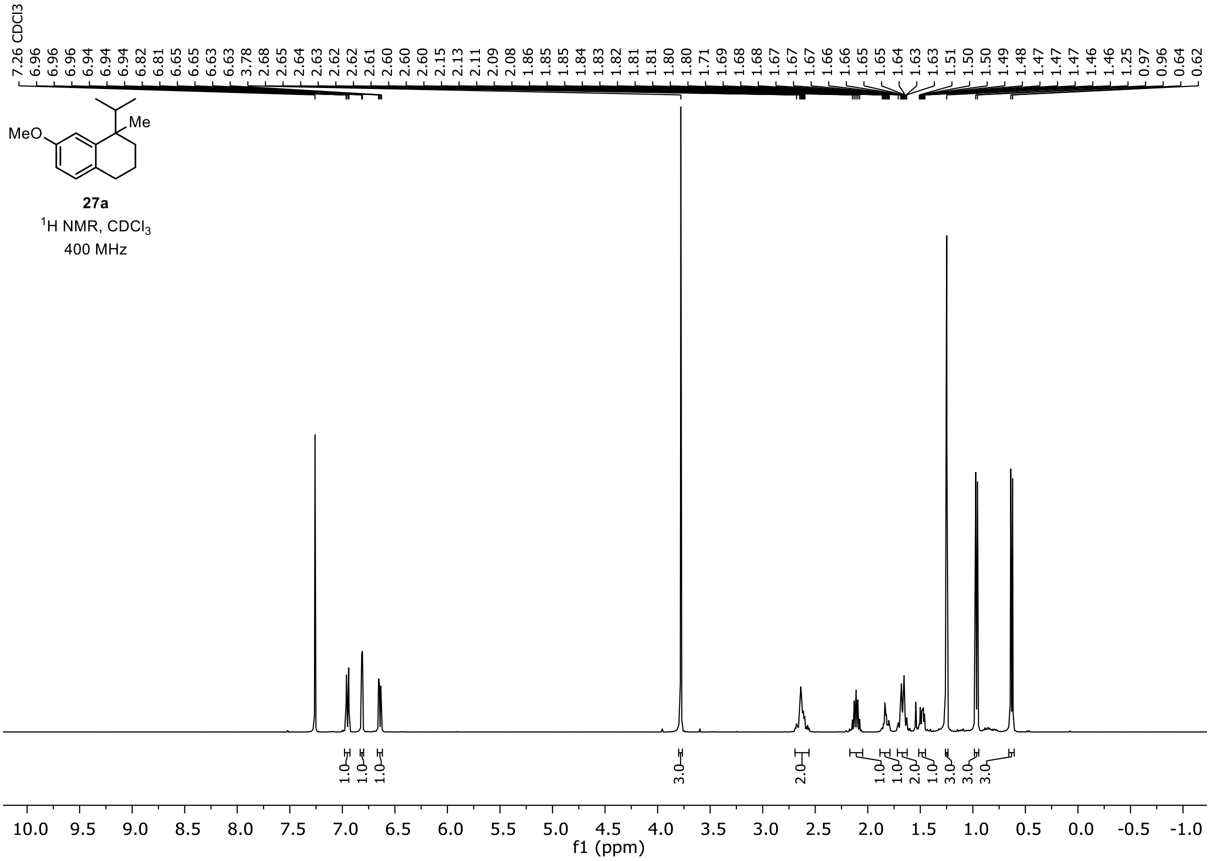

Figure 55. ^{1}H-NMR (400 MHz, $CDCl_3$) of tetralin **27a**.

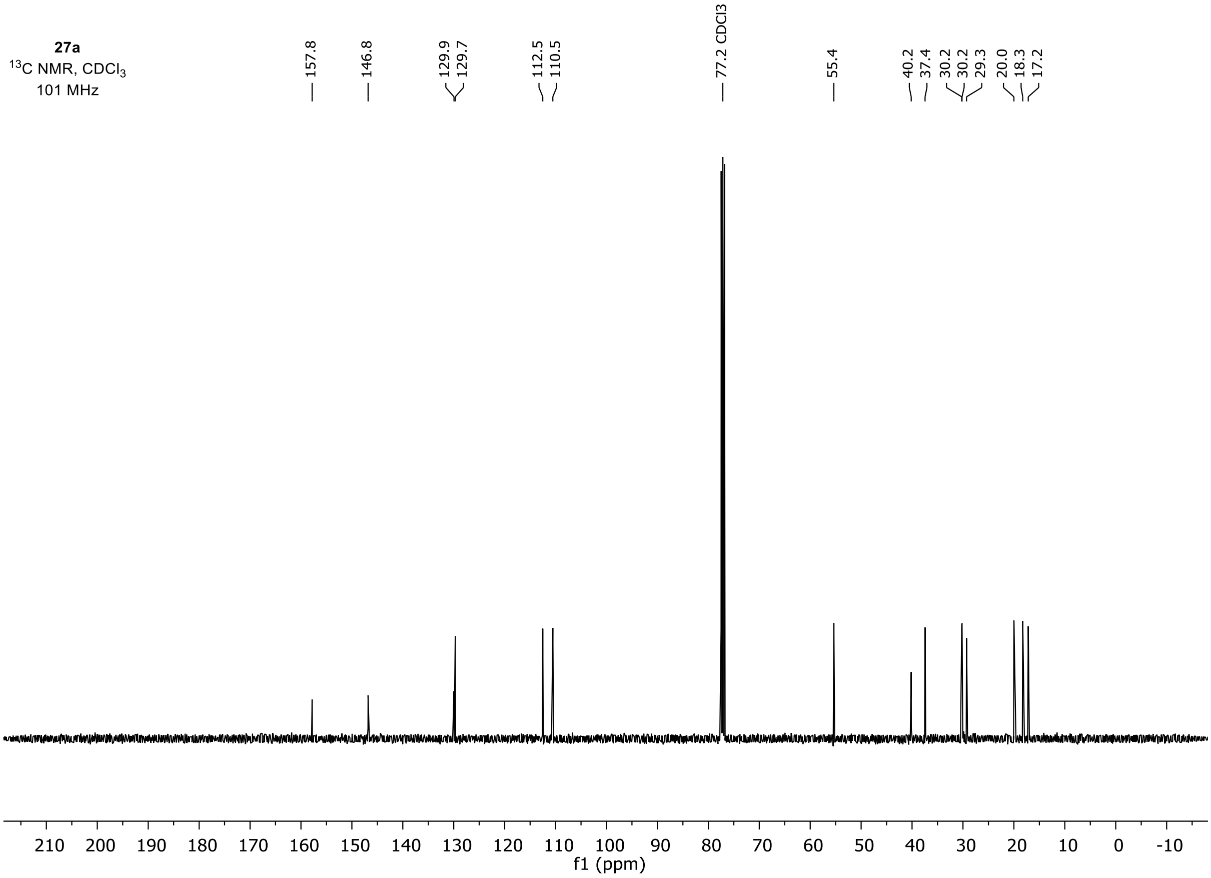

Figure 56. ^{13}C-NMR (101 MHz, $CDCl_3$) of tetralin **27a**.

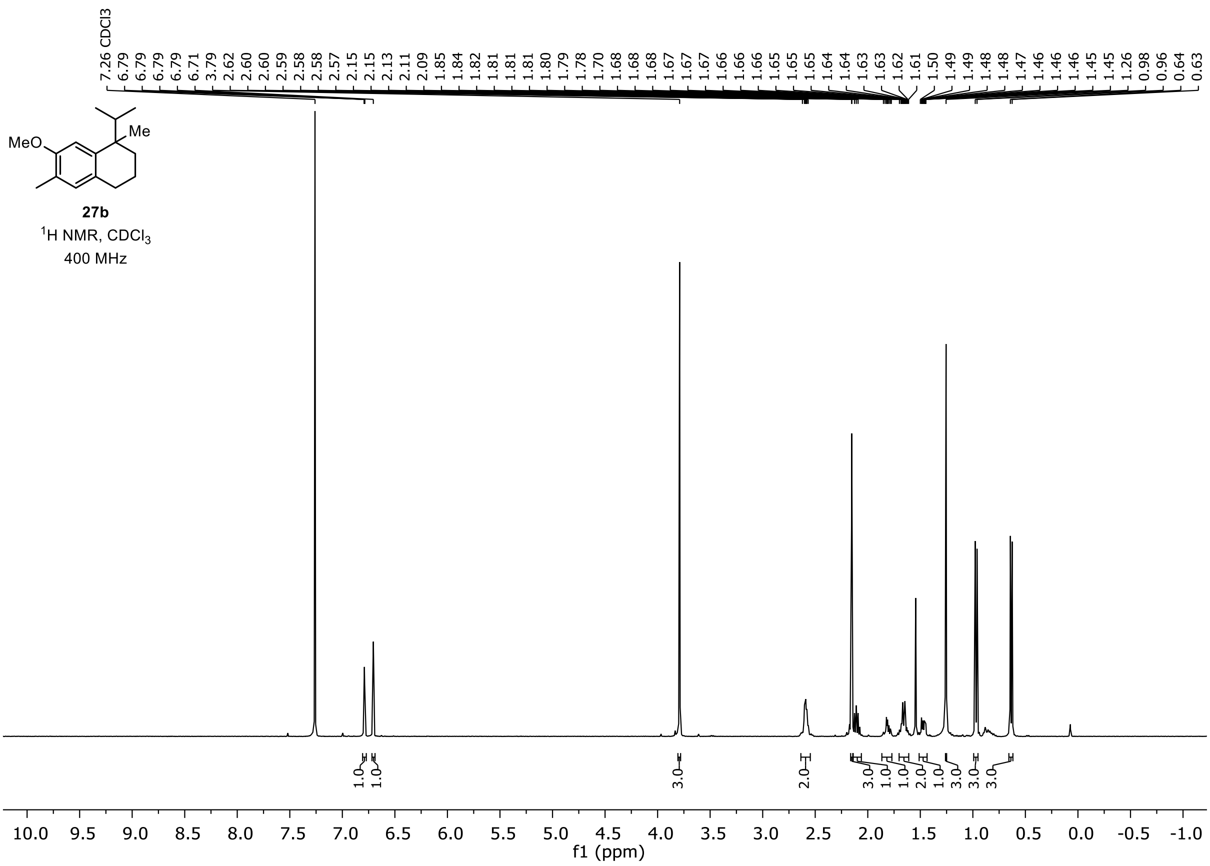

Figure 57. ^{1}H-NMR (400 MHz, $CDCl_3$) of tetralin **27b**.

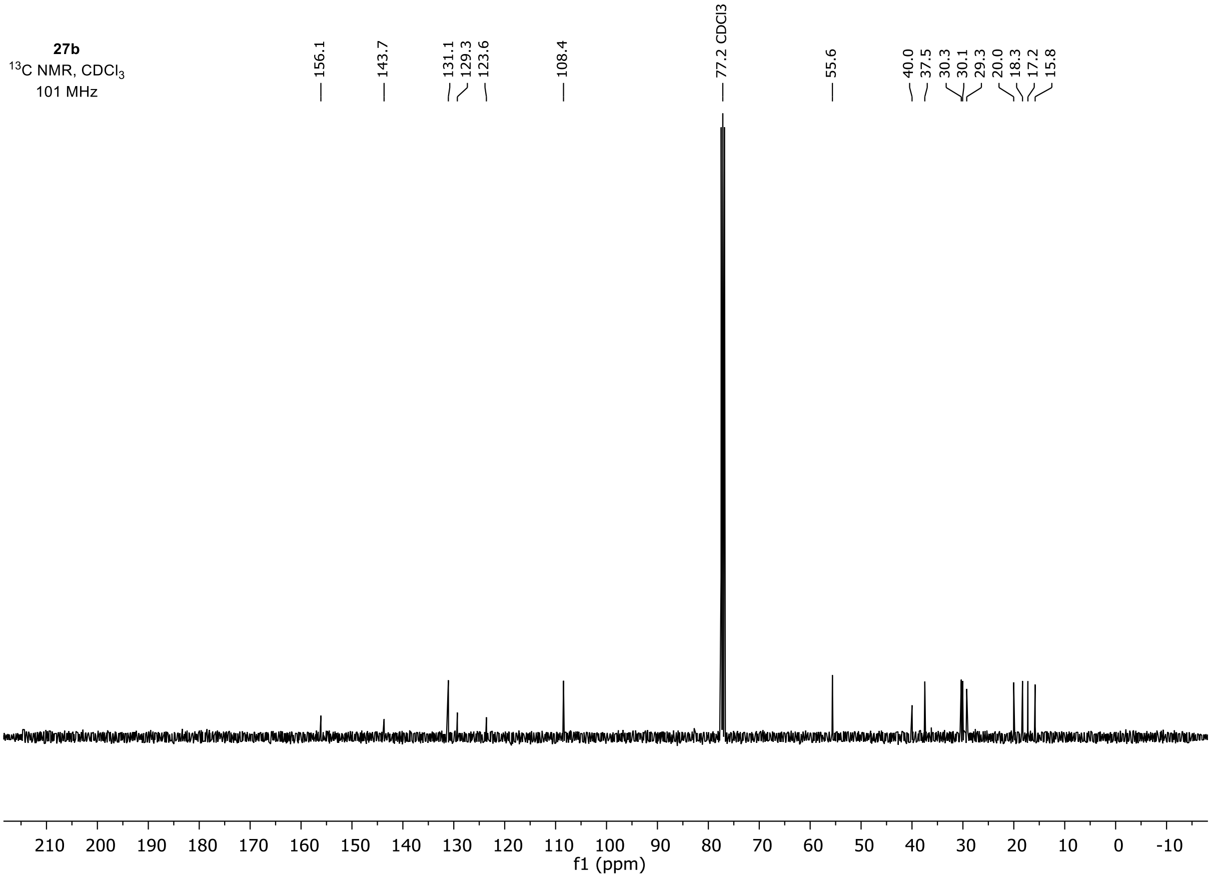

Figure 58. ^{13}C-NMR (101 MHz, $CDCl_3$) of tetralin **27b**.

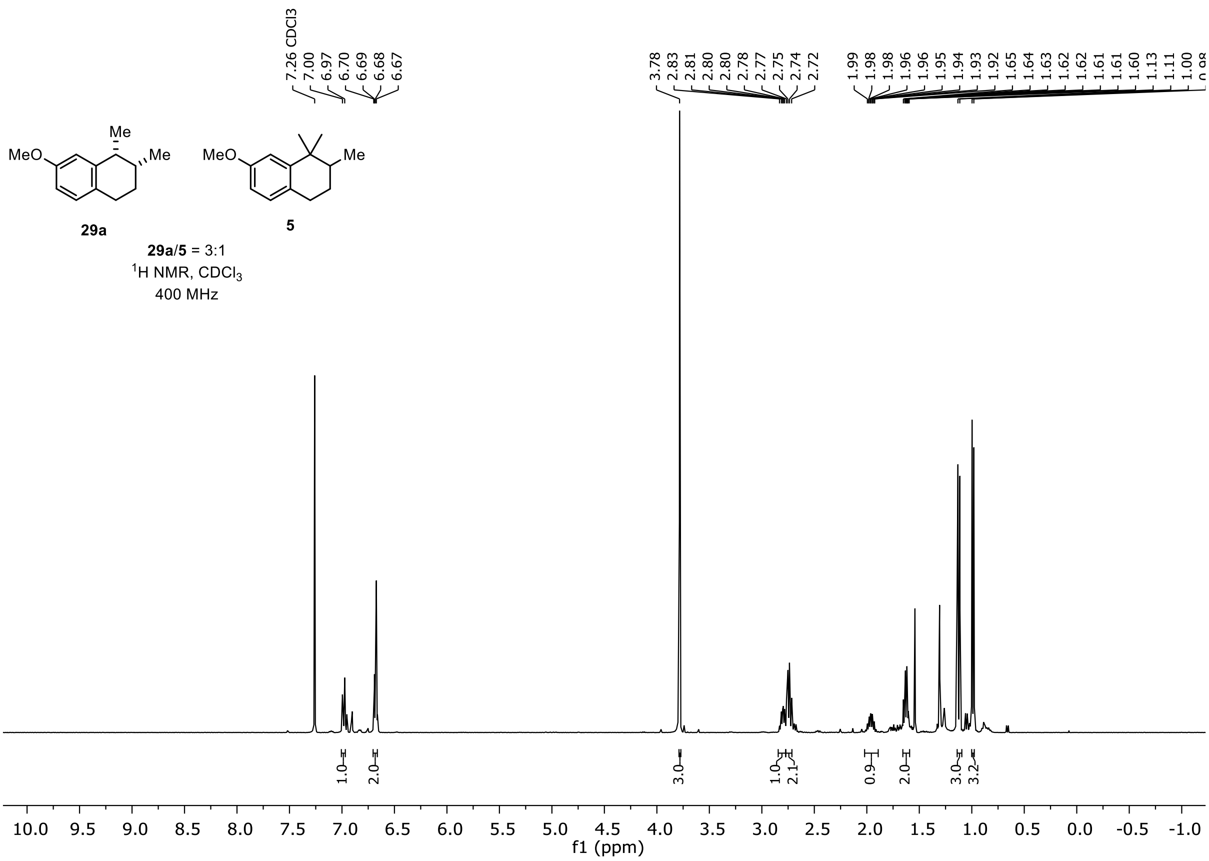

Figure 59. ^{1}H-NMR (400 MHz, $CDCl_3$) of tetralins **29a**/**5** (3:1). Only signals of **29a** are integrated.

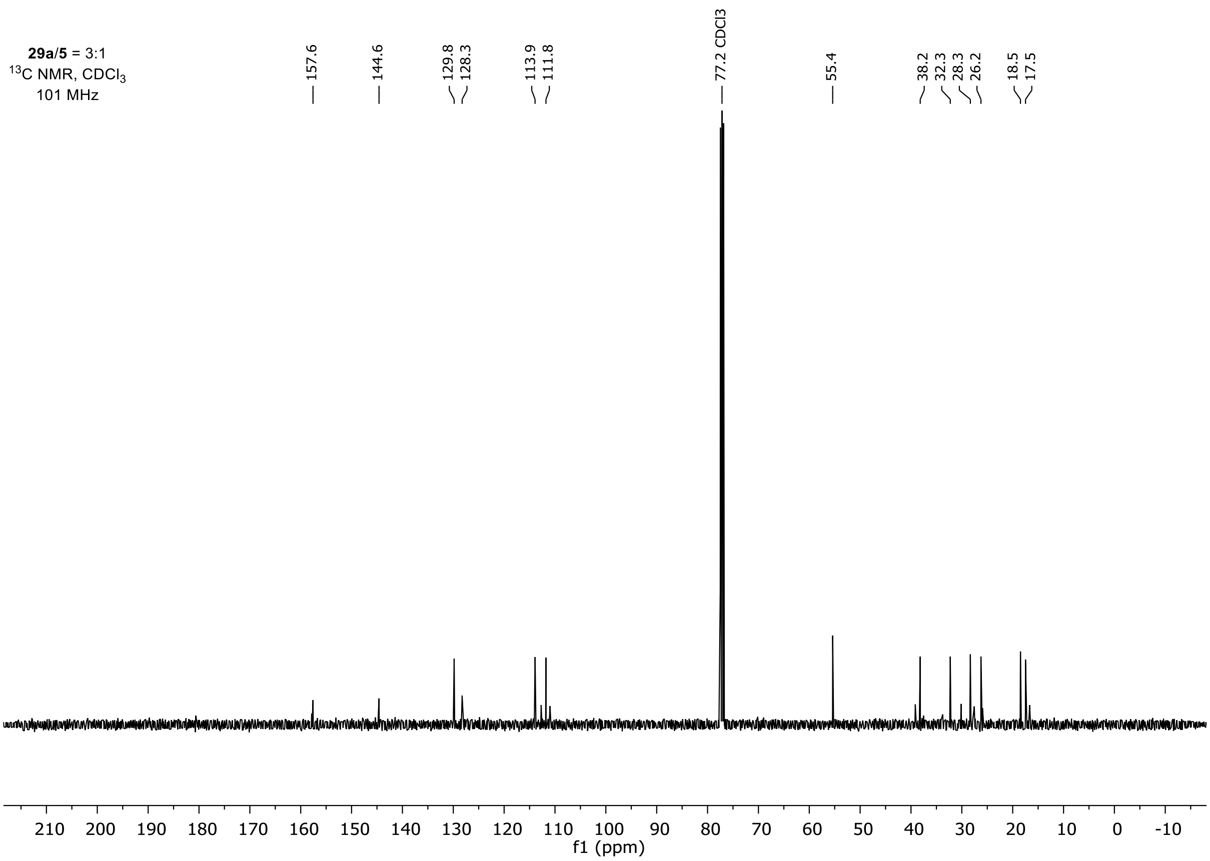

Figure 60. ^{13}C-NMR (101 MHz, $CDCl_3$) of tetralins **29a**/**5** (3:1). Only peaks of **29a** are picked.

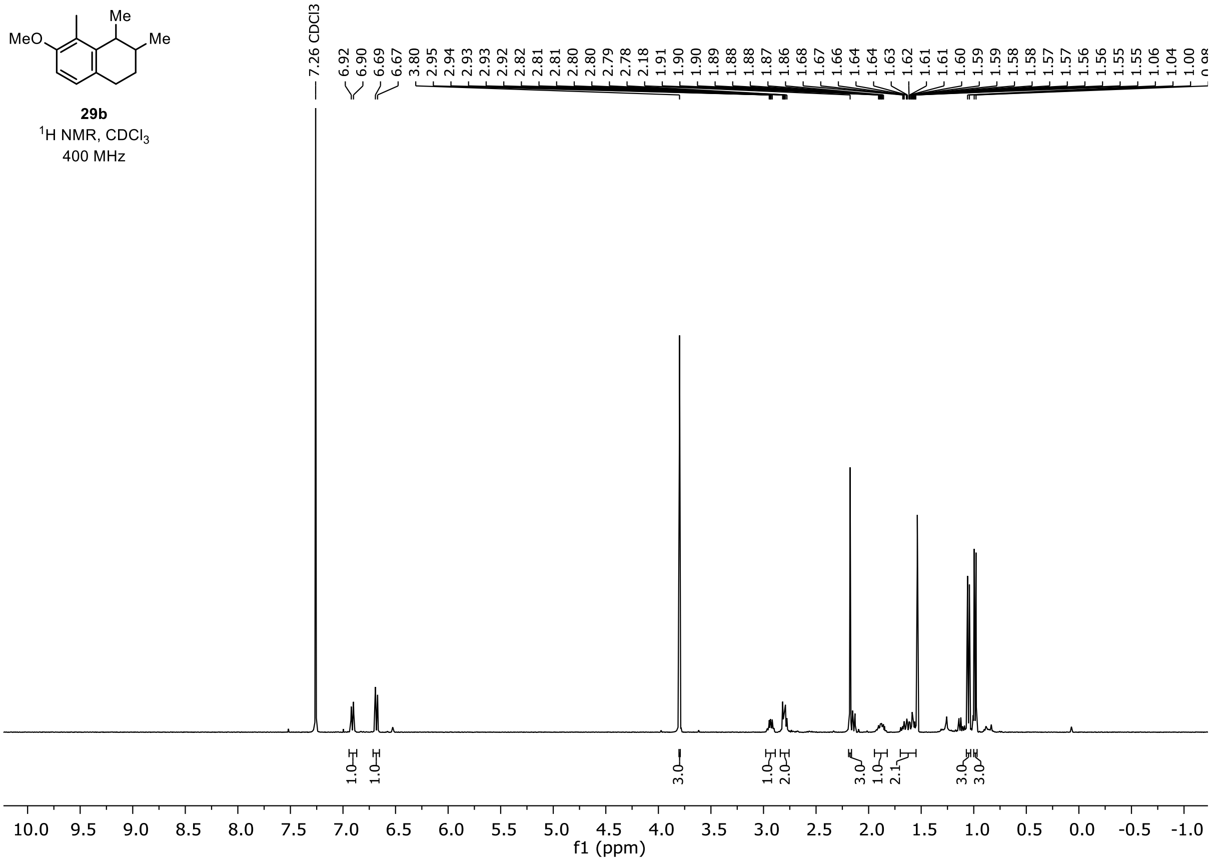

Figure 61. ^{1}H-NMR (400 MHz, $CDCl_3$) of tetralin **29b**.

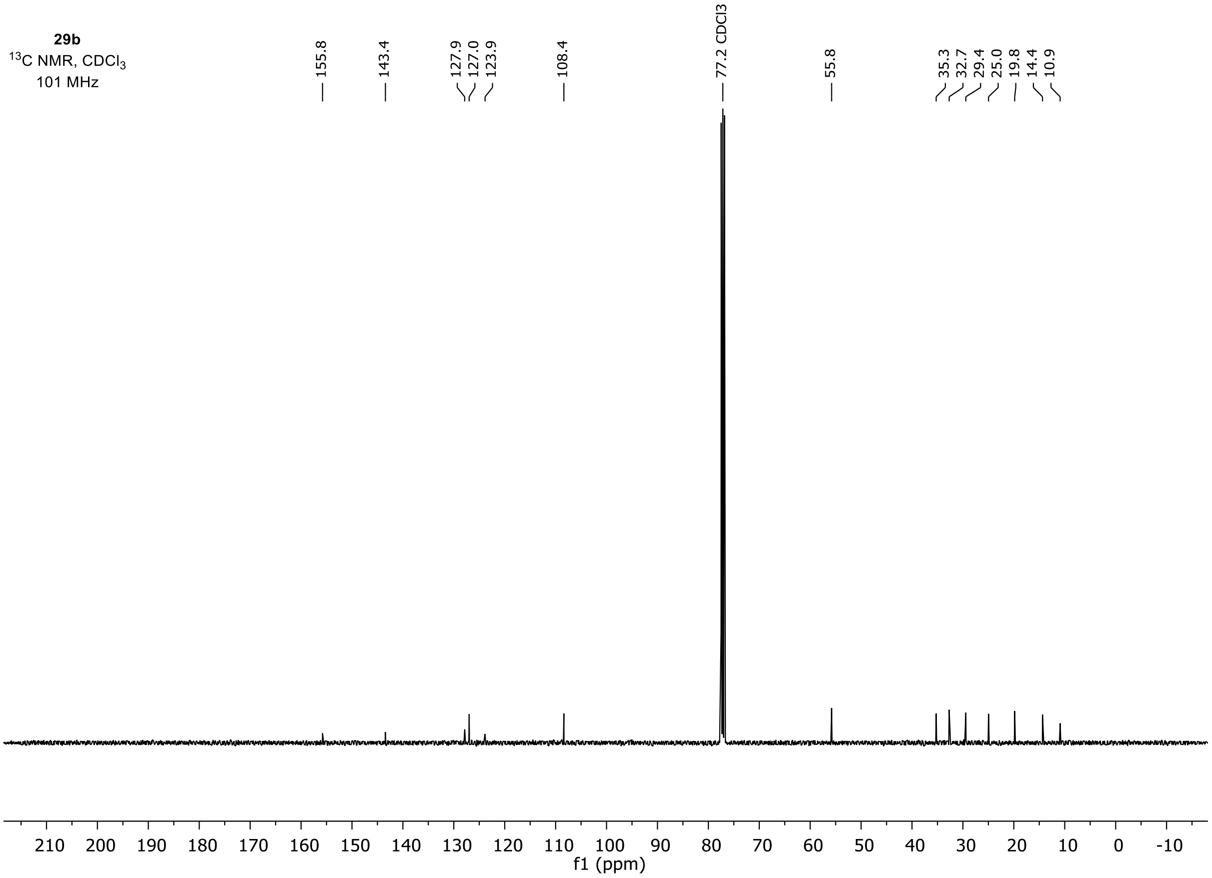

Figure 62. ^{13}C-NMR (101 MHz, $CDCl_3$) of tetralin **29b**.

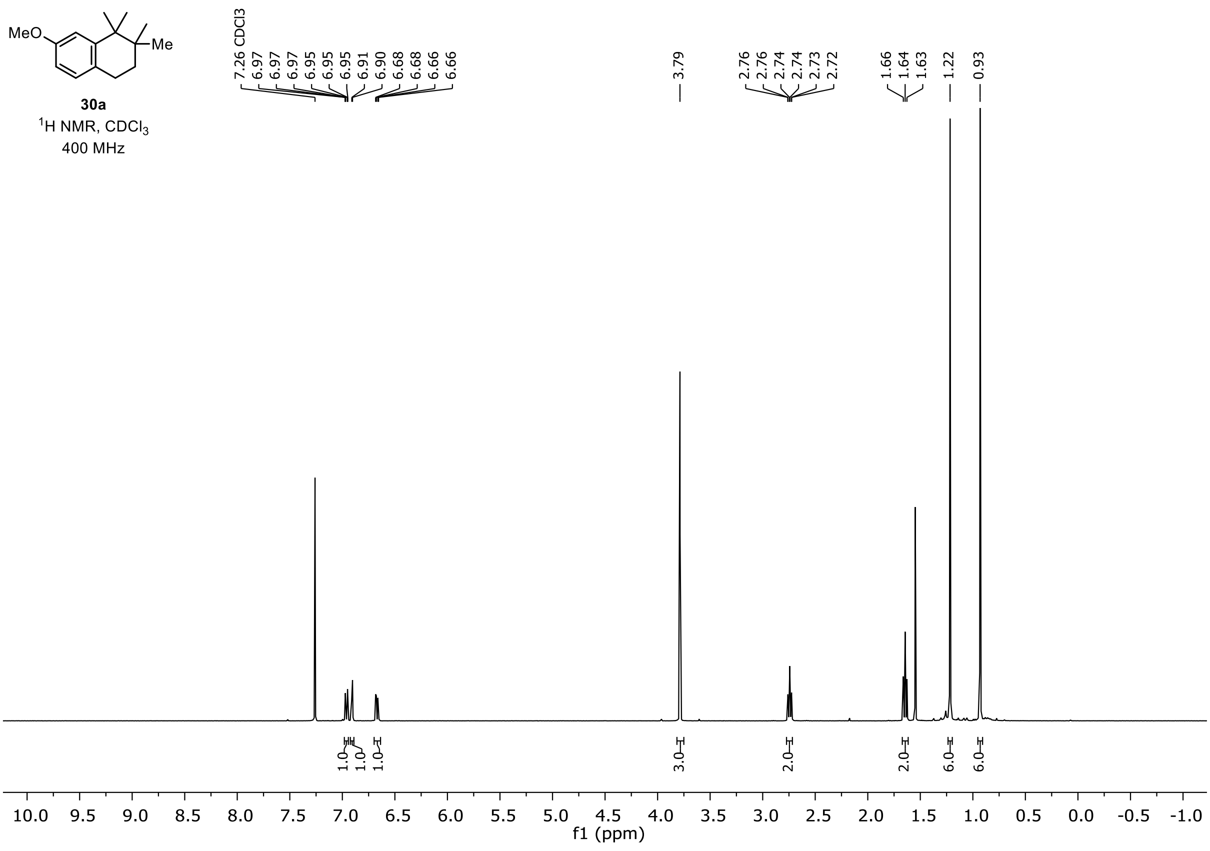

Figure 63. ^{1}H-NMR (400 MHz, $CDCl_3$) of tetralin **30a**.

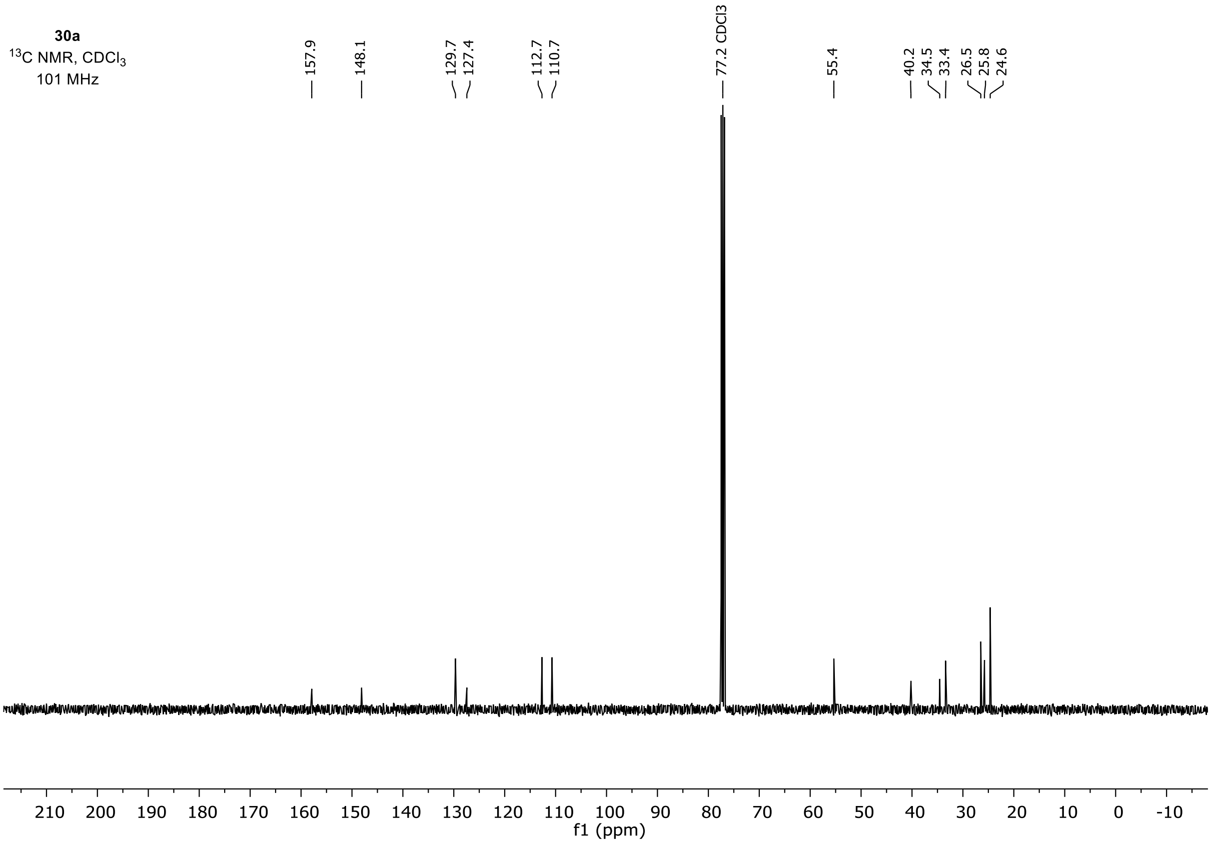

Figure 64. ^{13}C-NMR (101 MHz, $CDCl_3$) of tetralin **30a**.

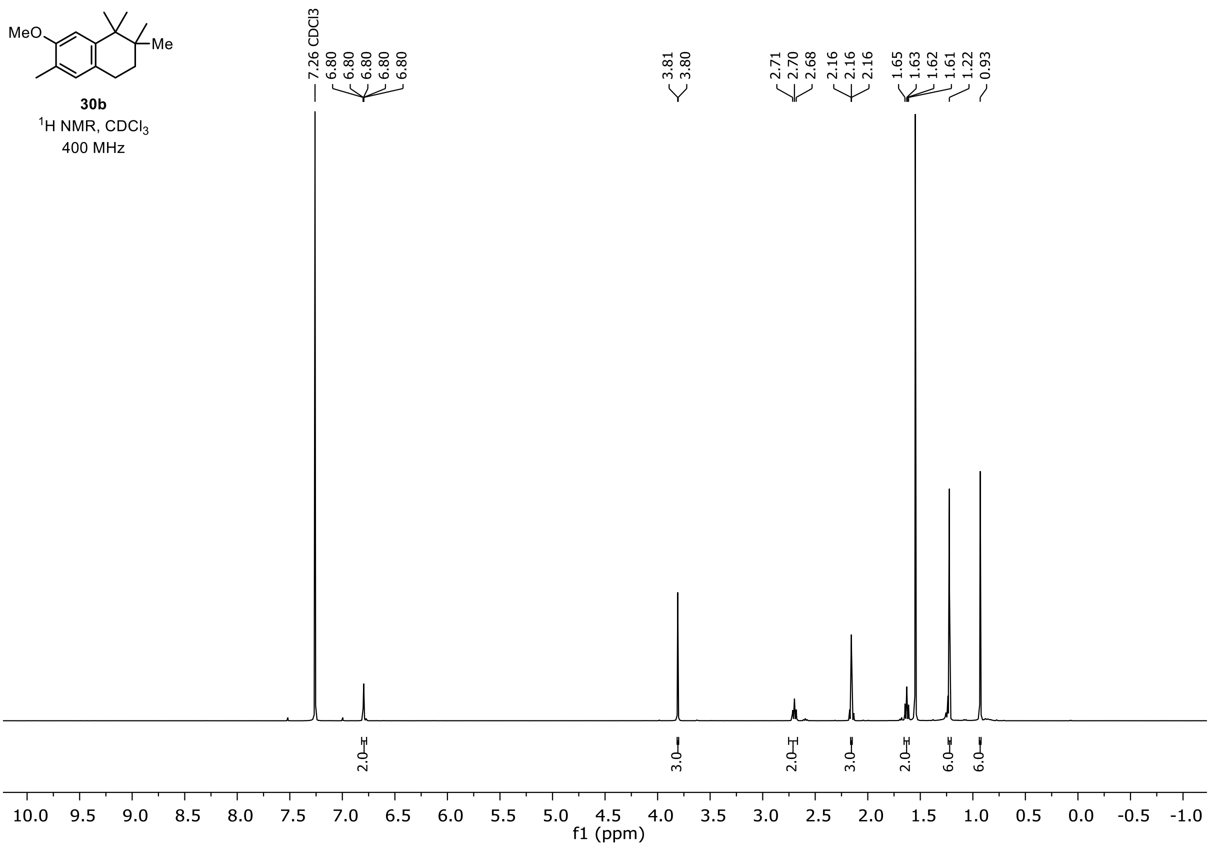

Figure 65. ^{1}H-NMR (400 MHz, $CDCl_3$) of tetralin **30b**.

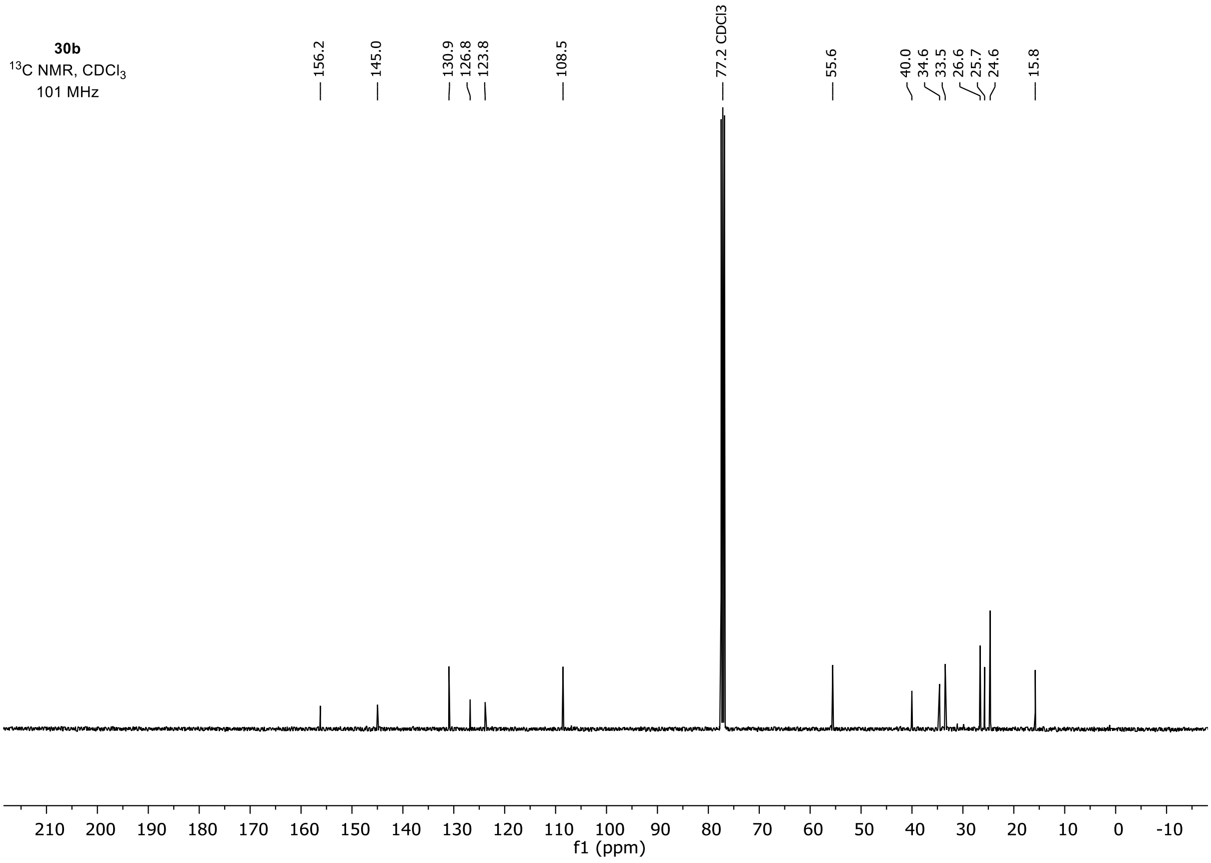

Figure 66. ^{13}C-NMR (101 MHz, $CDCl_3$) of tetralin **30b**.

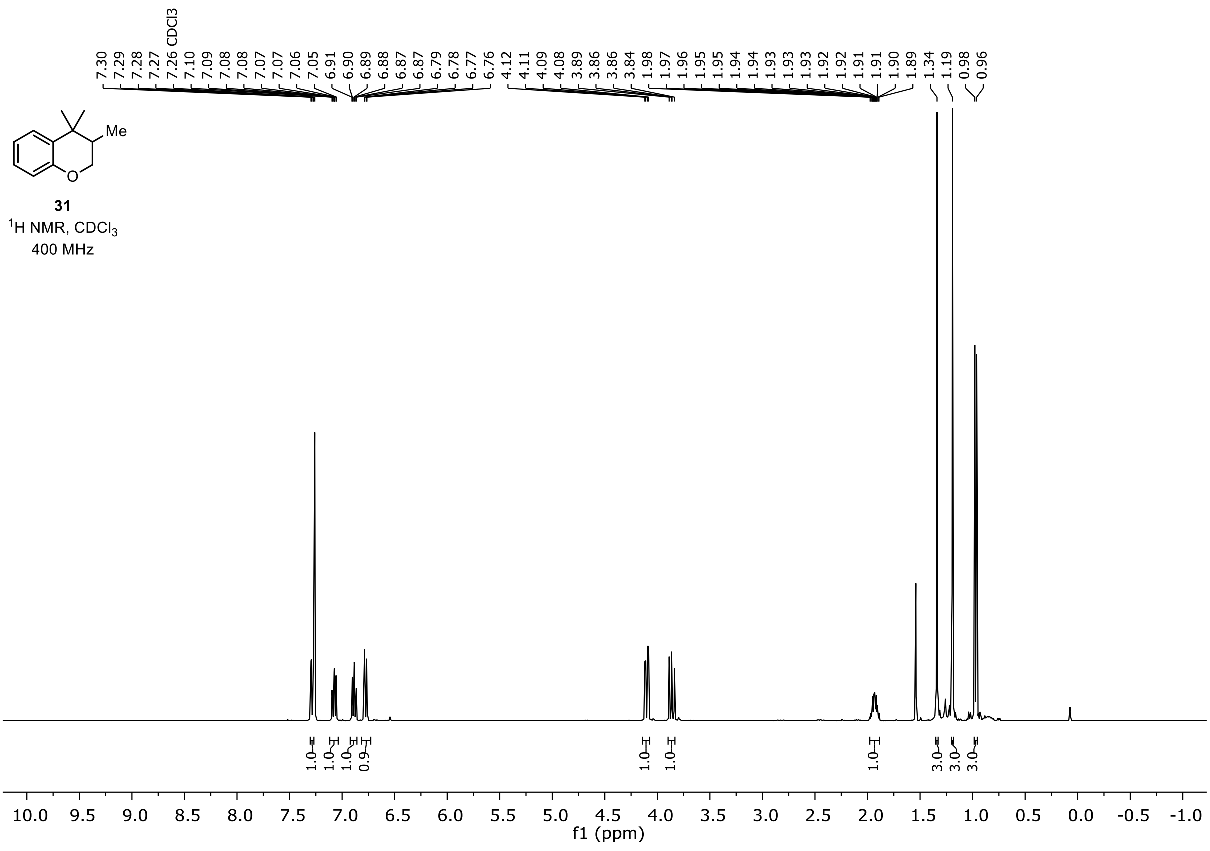

Figure 67. ^{1}H-NMR (400 MHz, $CDCl_3$) of chromane **31**.

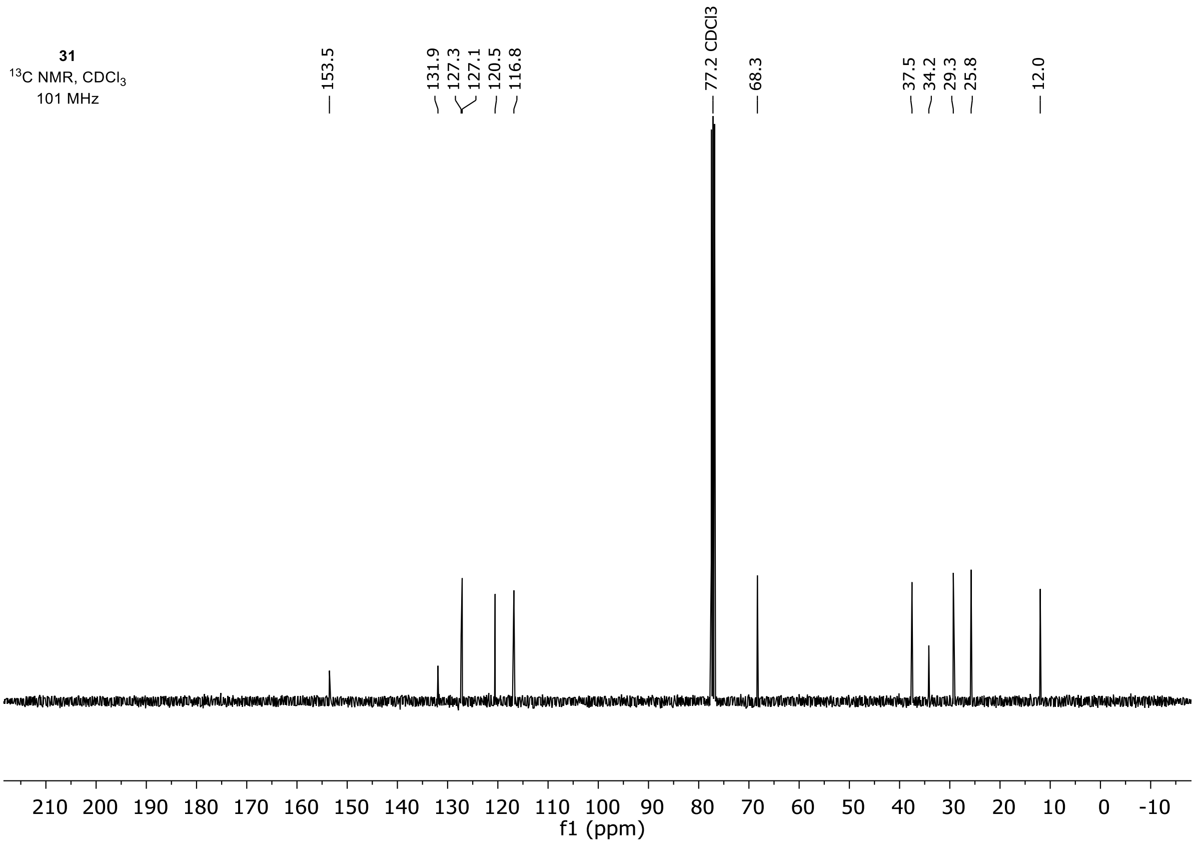

Figure 68. ^{13}C-NMR (101 MHz, $CDCl_3$) of chromane **31**.

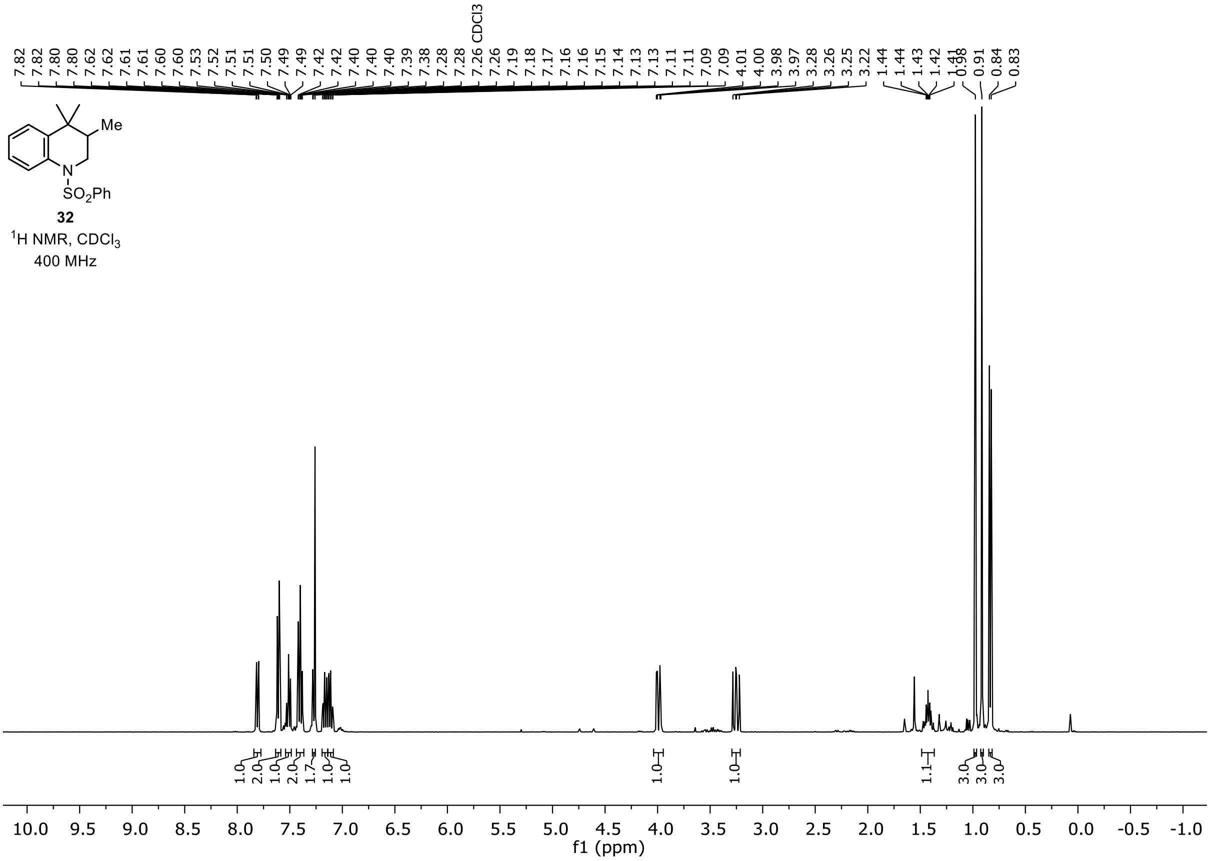

Figure 69. ^{1}H-NMR (400 MHz, $CDCl_3$) of tetrahydroquinoline **32**.

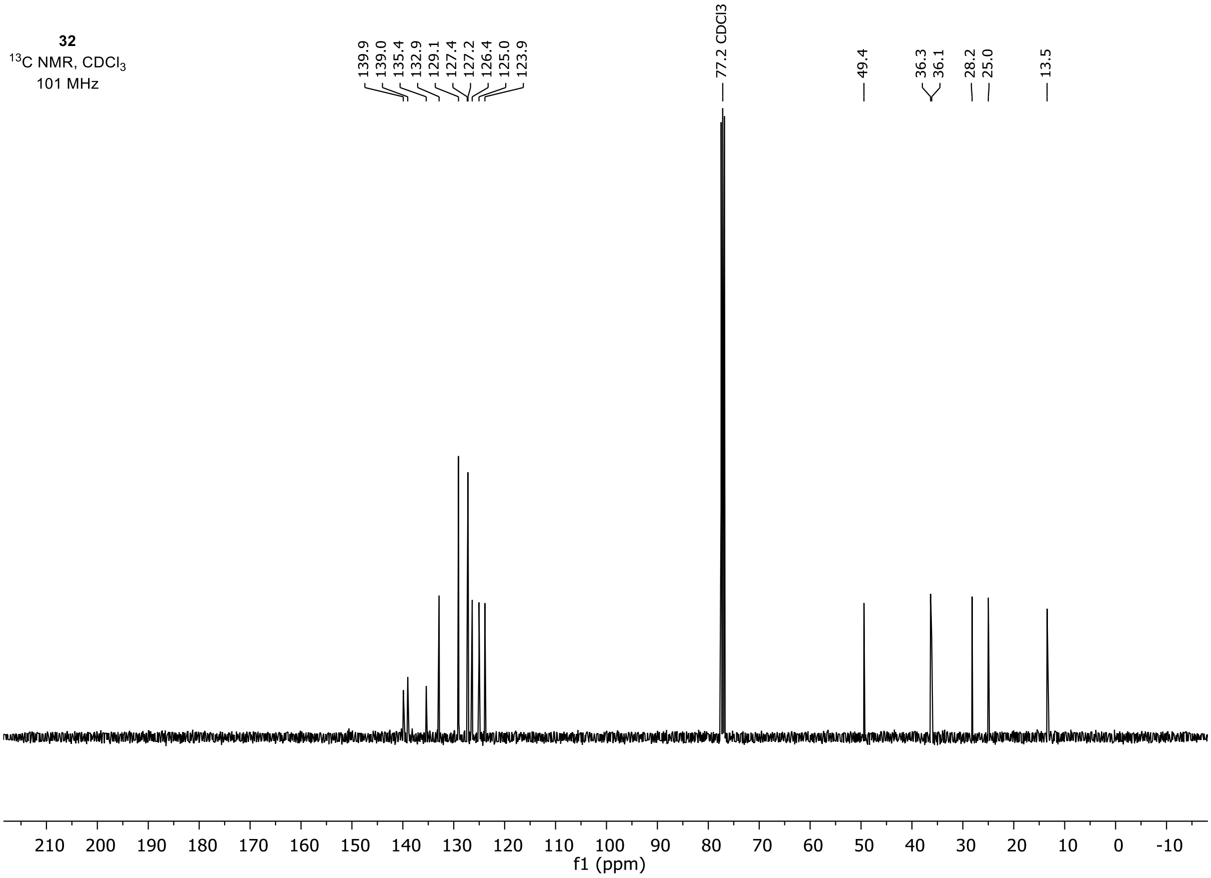

Figure 70. ^{13}C-NMR (101 MHz, $CDCl_3$) of tetrahydroquinoline **32**.

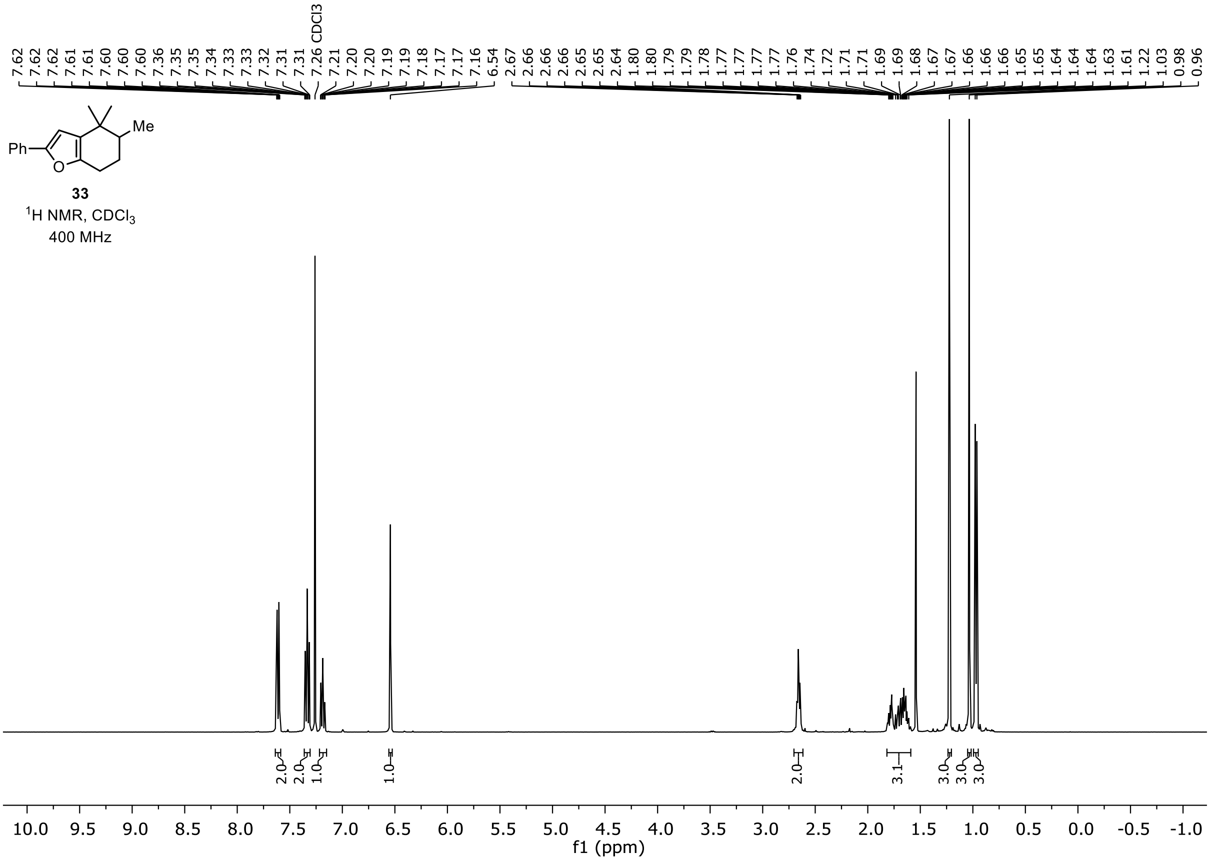

Figure 71. ^{1}H-NMR (400 MHz, $CDCl_3$) of furan **33**.

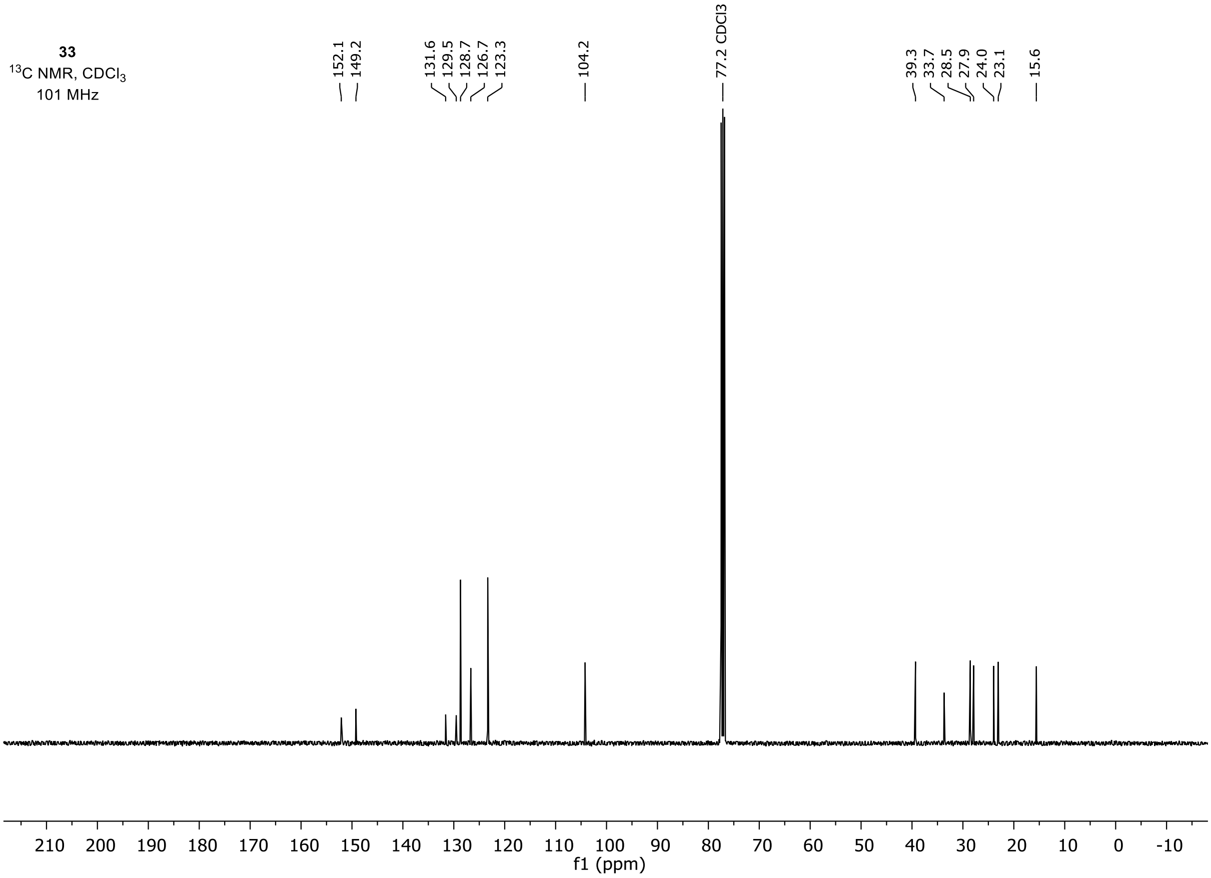

Figure 72. ^{13}C-NMR (101 MHz, $CDCl_3$) of furan **33**.

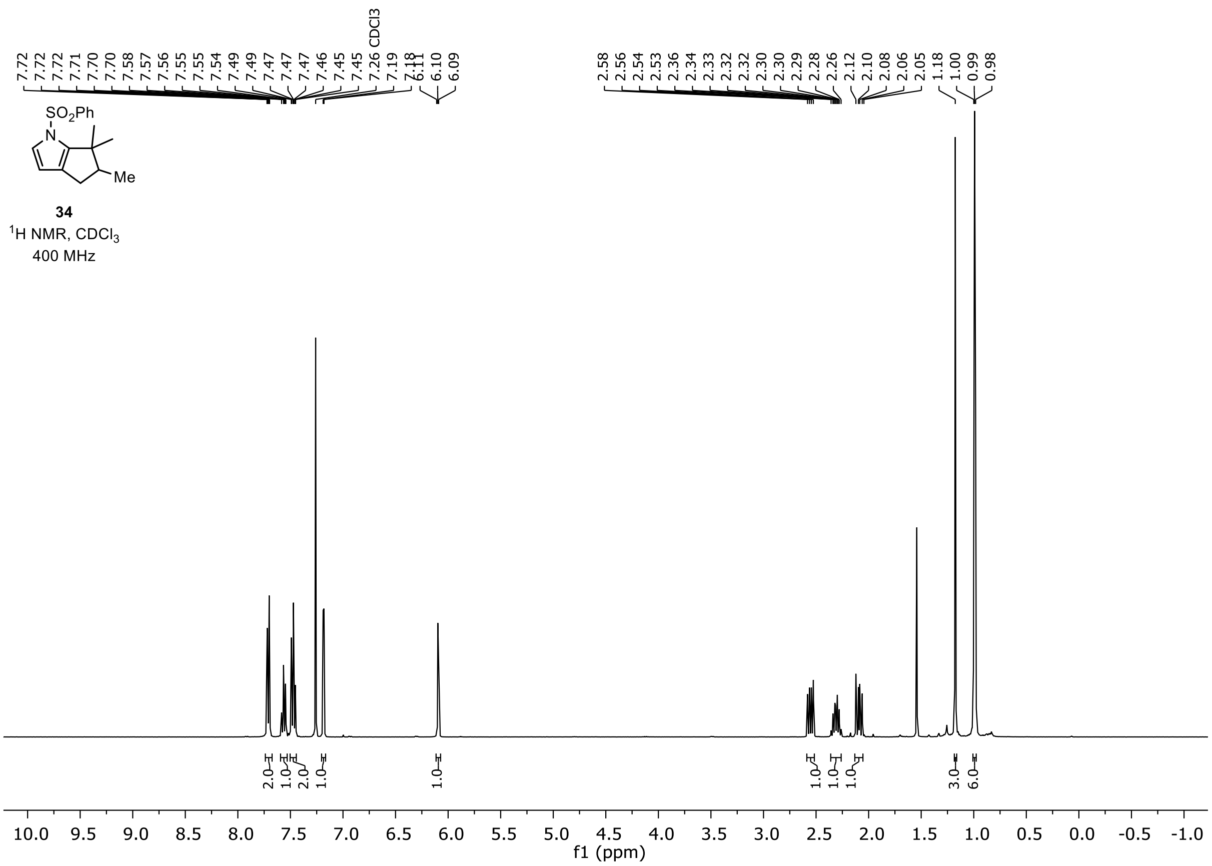

Figure 73. ^{1}H-NMR (400 MHz, $CDCl_3$) of pyrrole **34**.

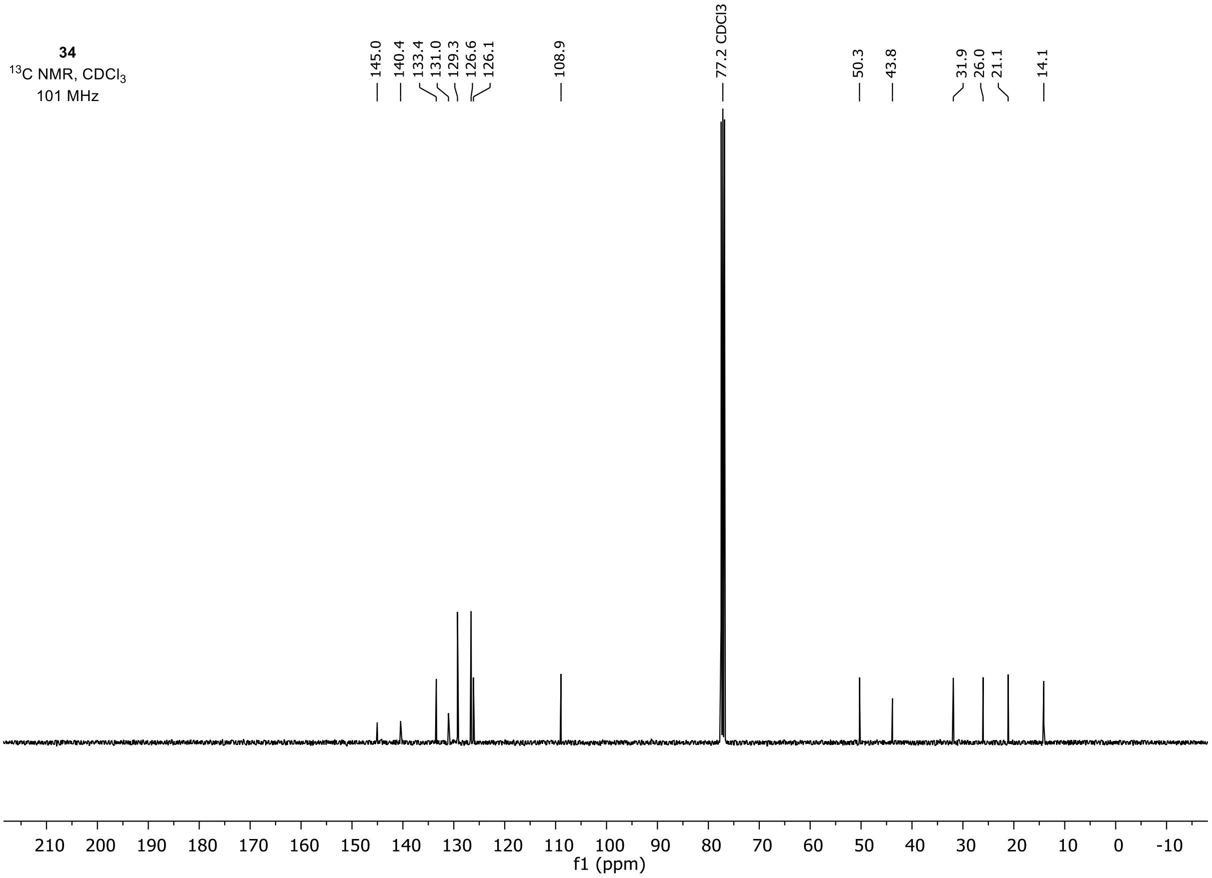

Figure 74. ^{13}C-NMR (101 MHz, $CDCl_3$) of pyrrole **34**.

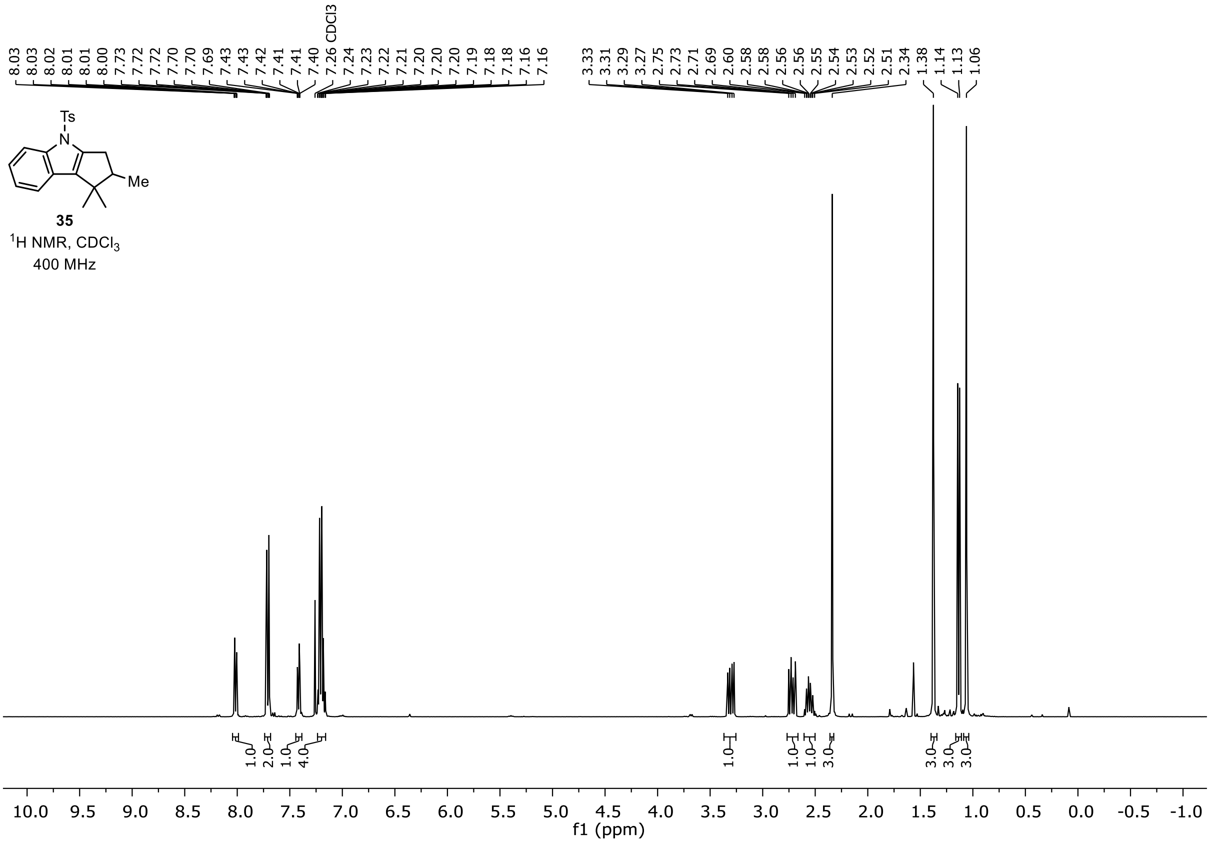

Figure 75. ^{1}H-NMR (400 MHz, $CDCl_3$) of indole **35**.

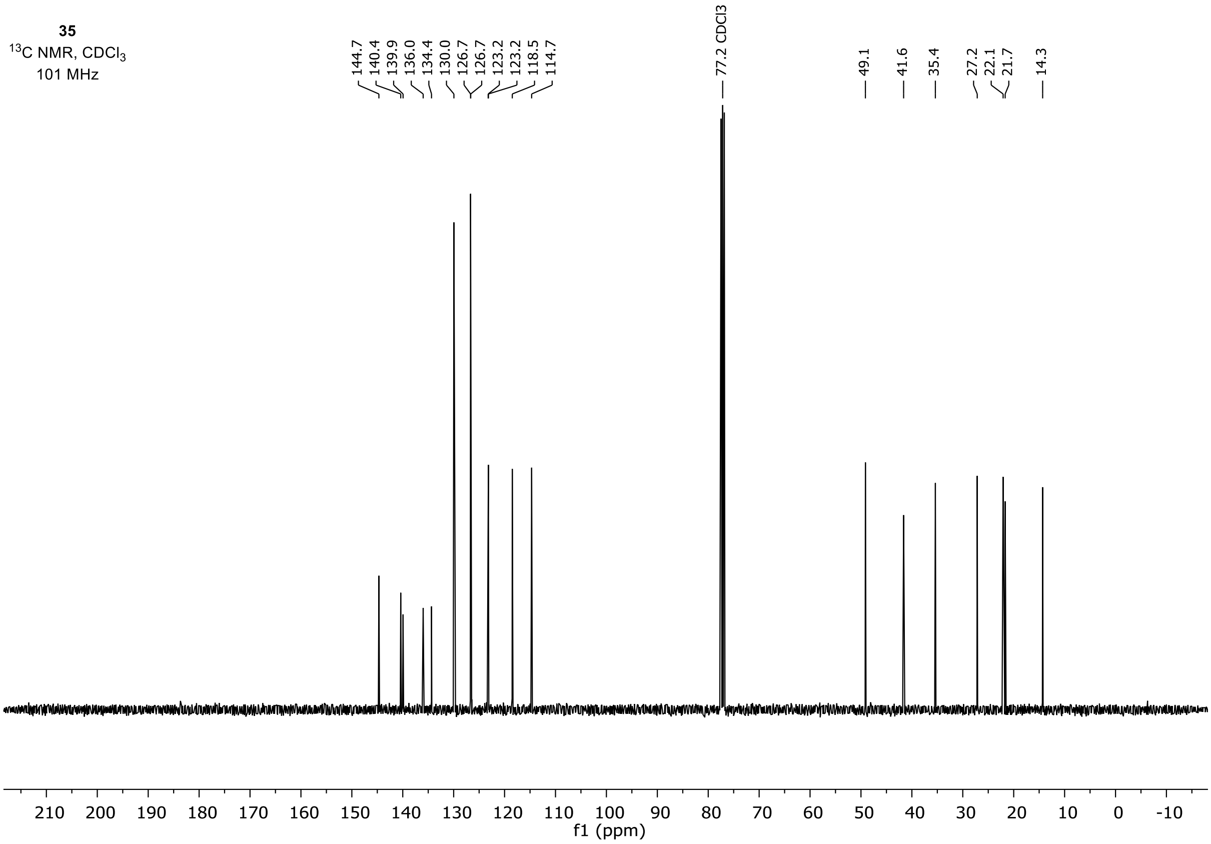

Figure 76. ^{13}C-NMR (101 MHz, $CDCl_3$) of indole **35**.

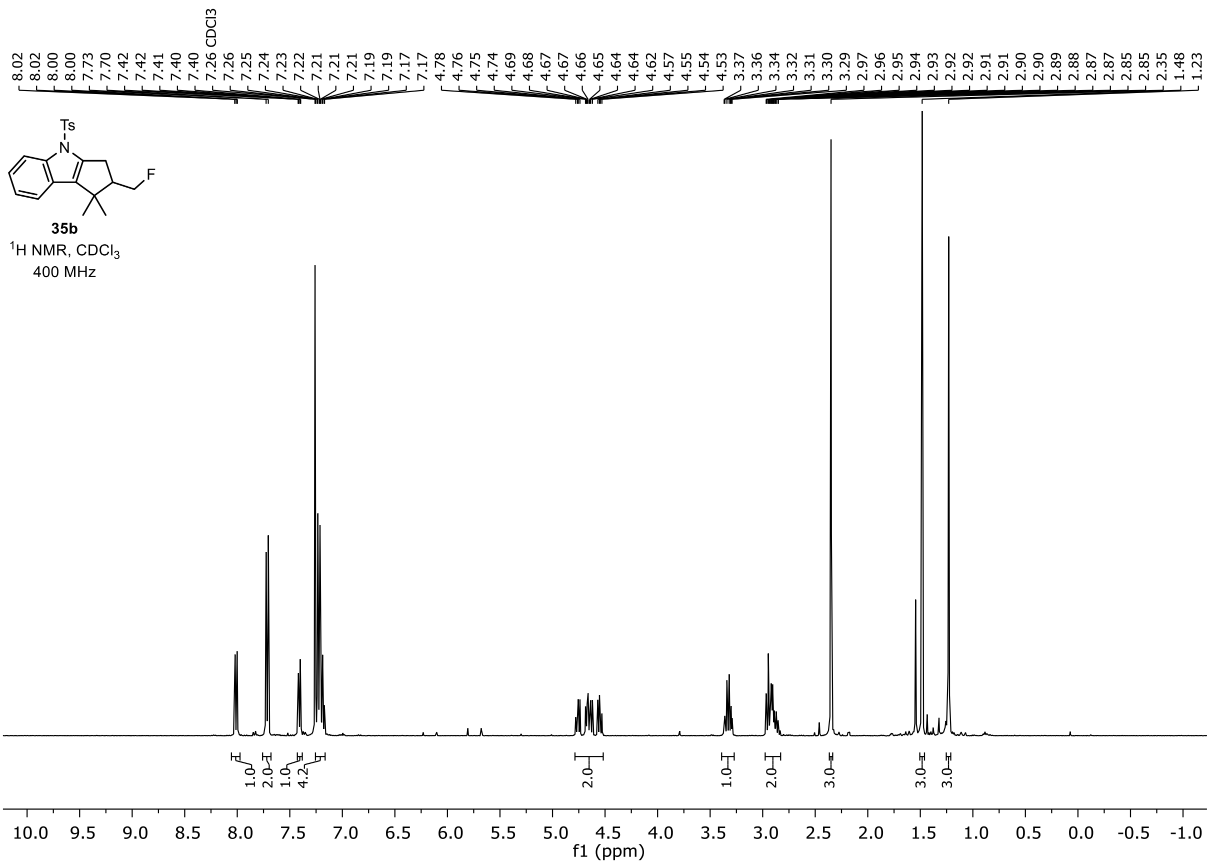

Figure 77. ^{1}H-NMR (400 MHz, $CDCl_3$) of indole **35b**.

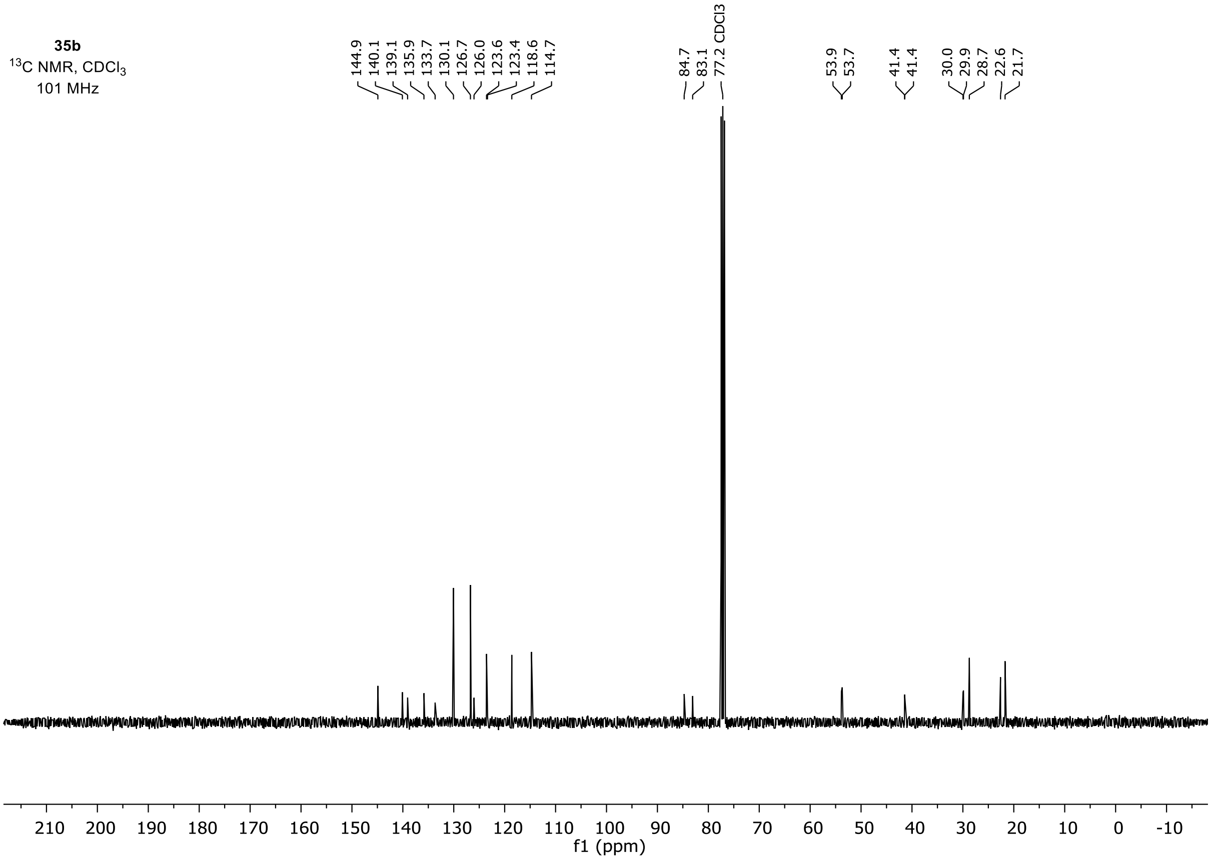

Figure 78. ^{13}C-NMR (101 MHz, $CDCl_3$) of indole **35b**.

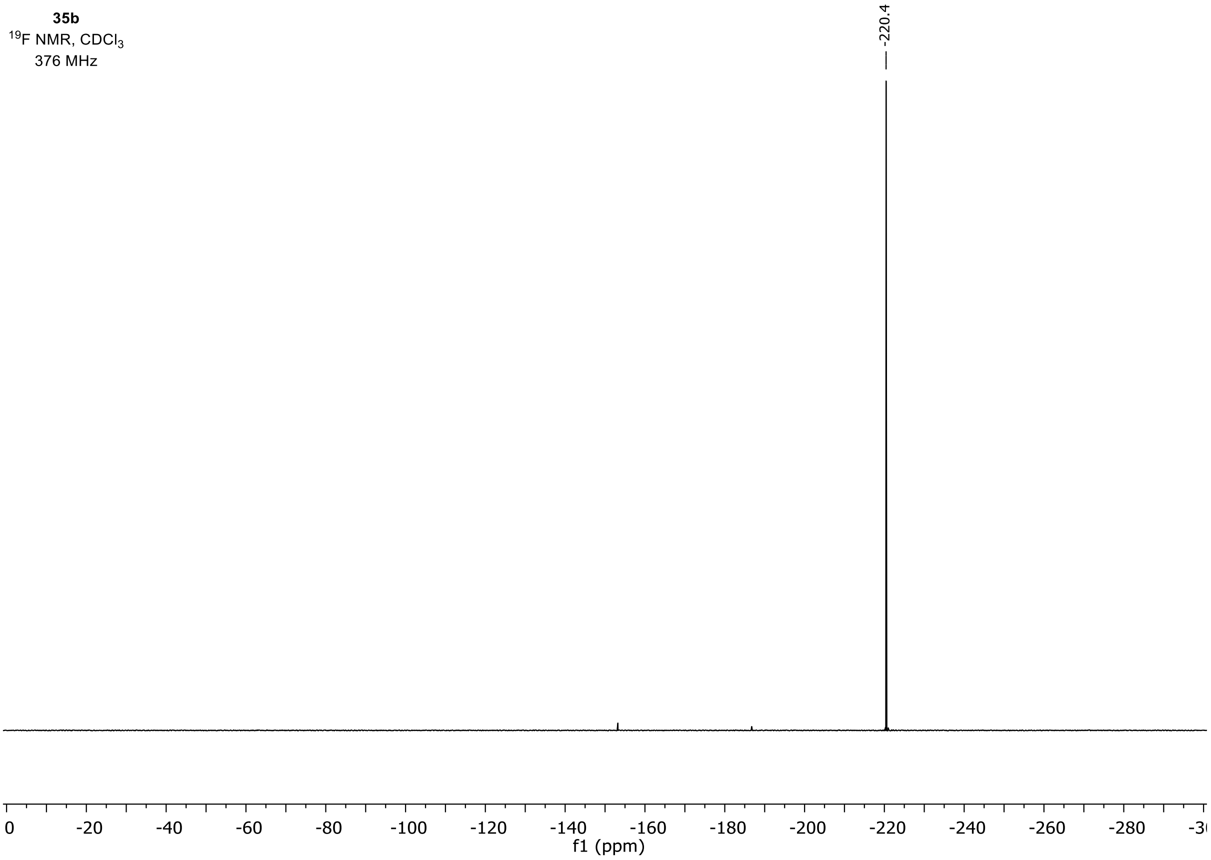

Figure 79. ^{19}F-NMR (376 MHz, $CDCl_3$) of indole **35b**.

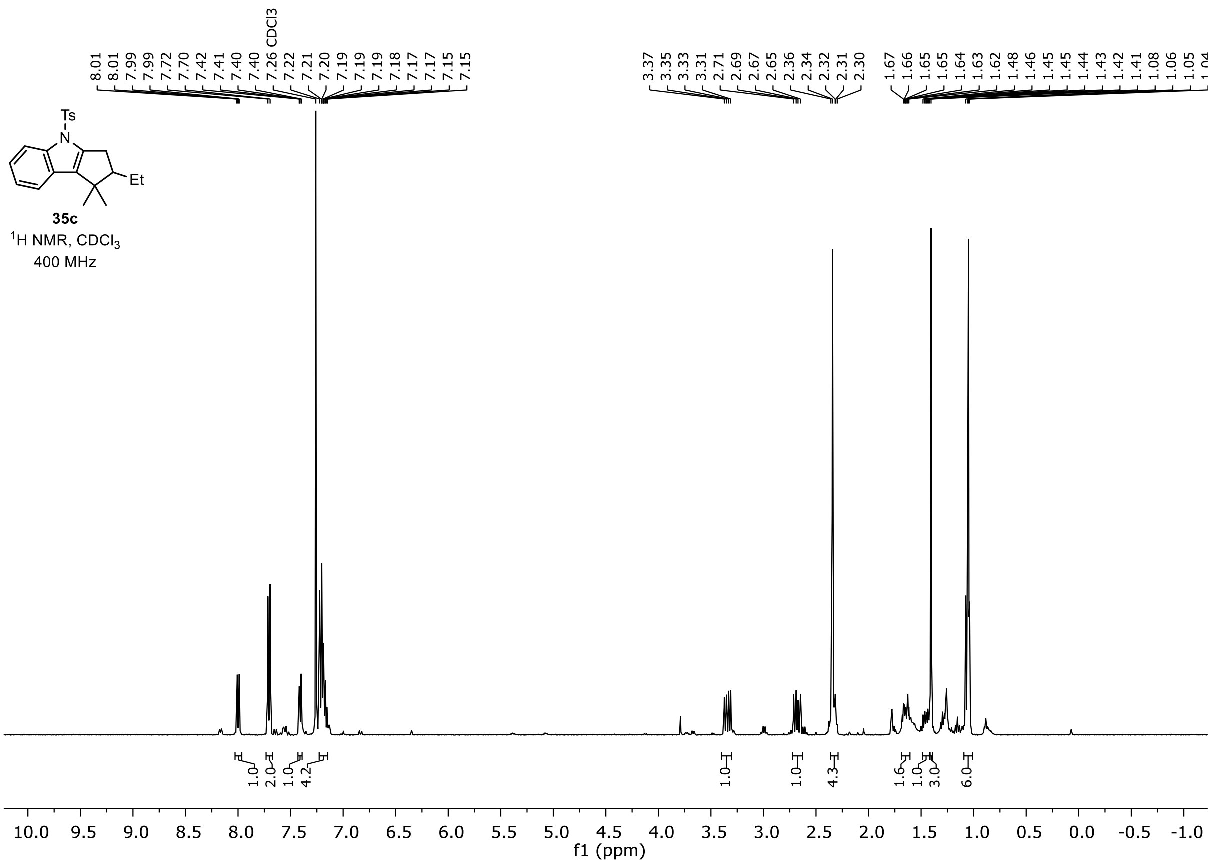

Figure 80. ^{1}H-NMR (400 MHz, $CDCl_3$) of indole **35c**.

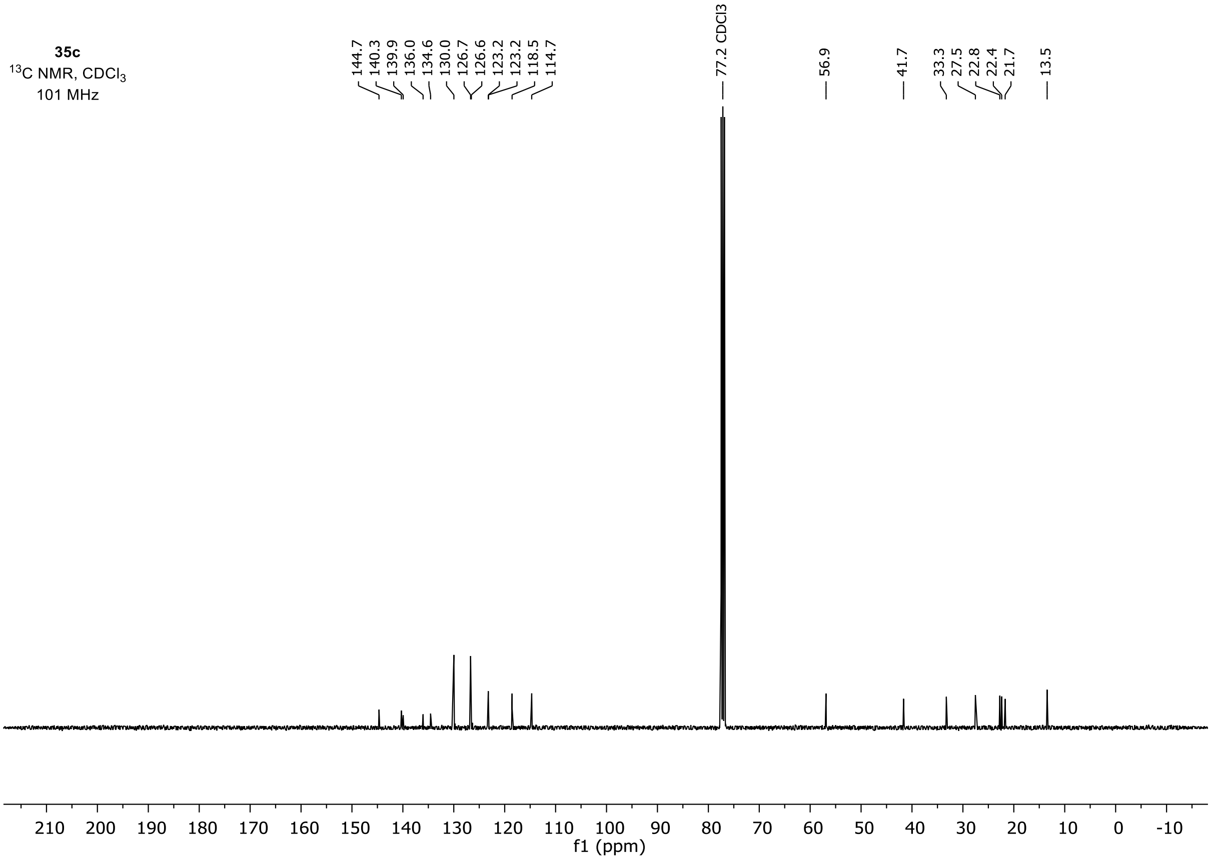

Figure 81. ^{13}C-NMR (101 MHz, $CDCl_3$) of indole **35c**.

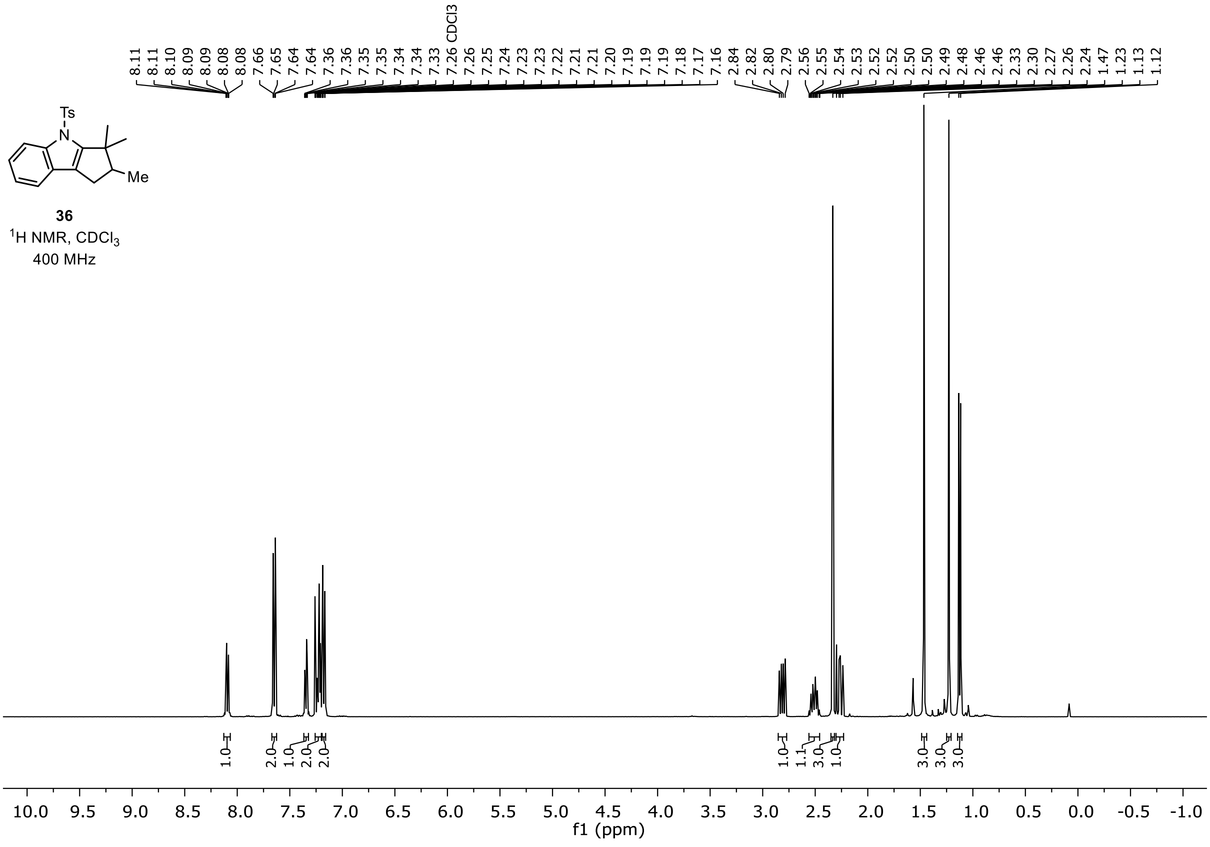

Figure 82. ^{1}H-NMR (400 MHz, $CDCl_3$) of indole **36**.

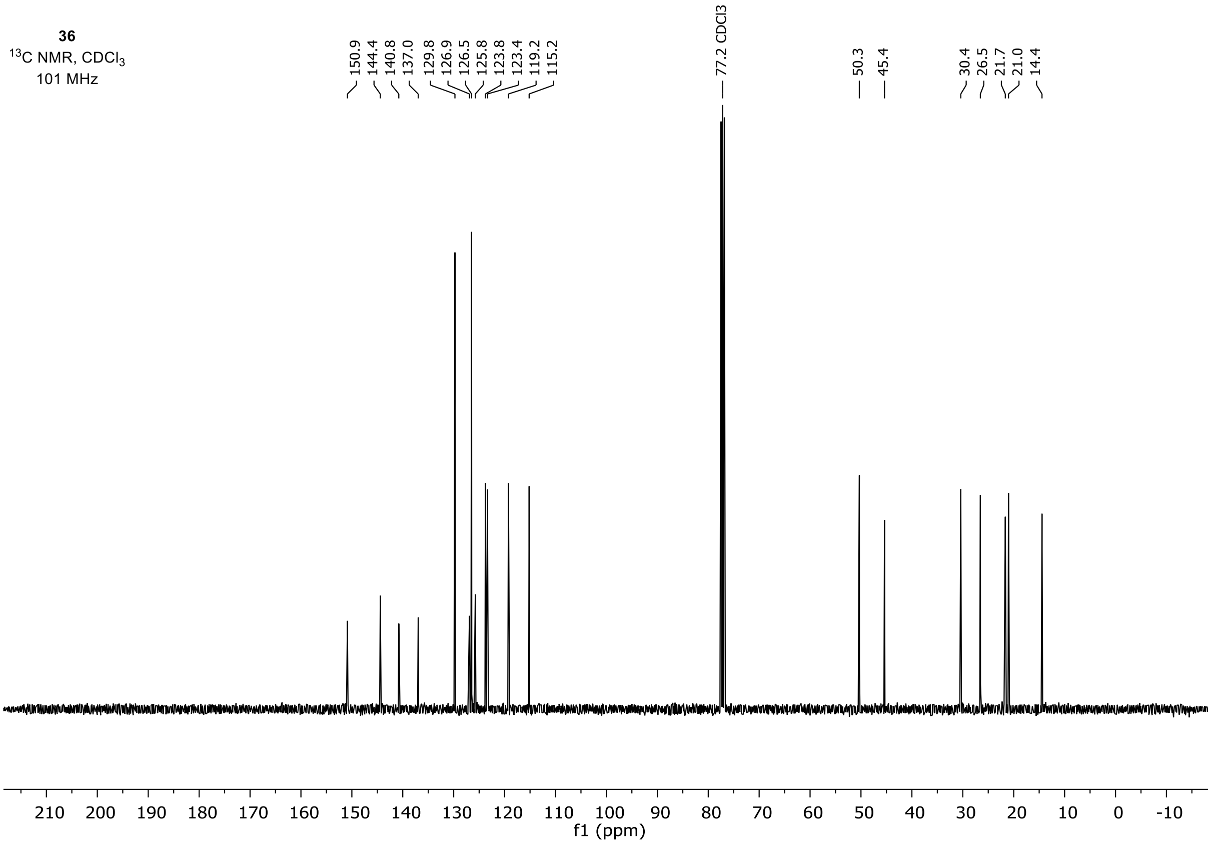

Figure 83. ^{13}C-NMR (101 MHz, $CDCl_3$) of indole **36**.

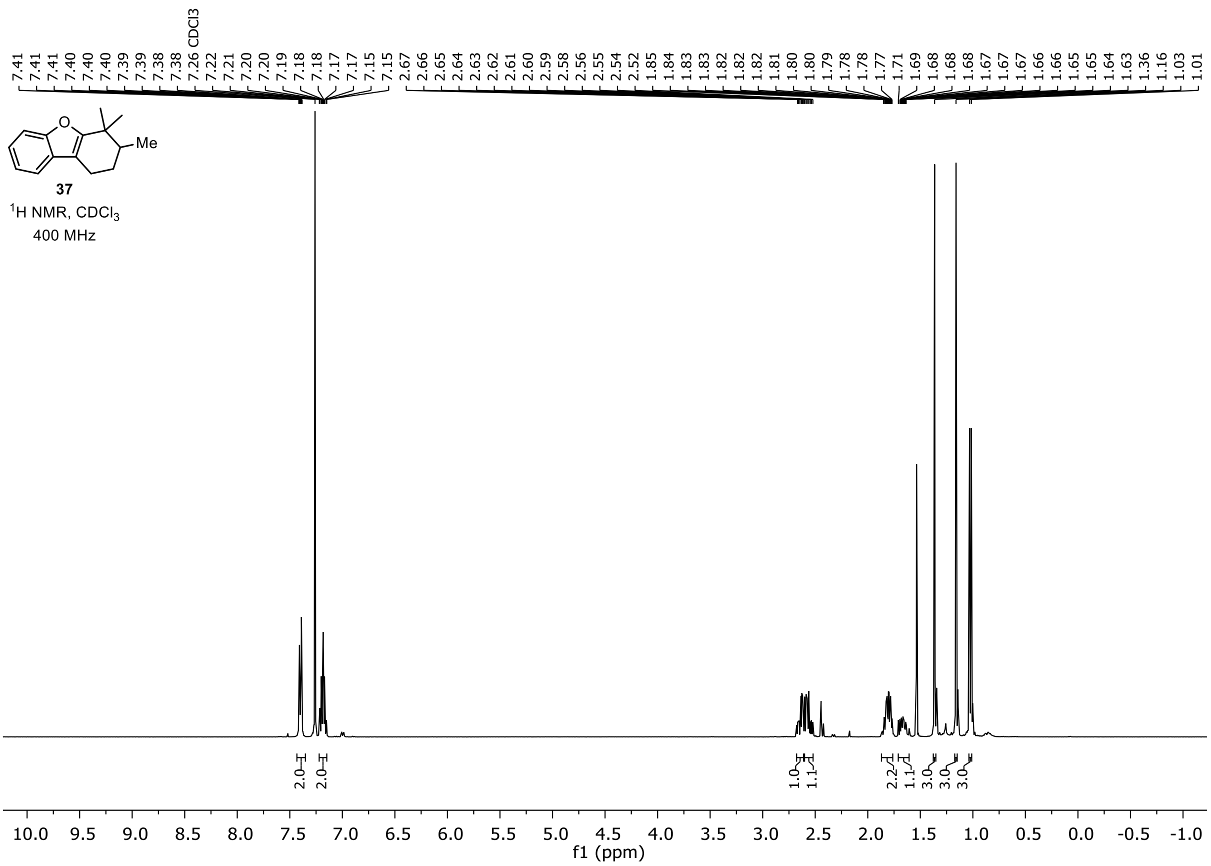

Figure 84. ^{1}H-NMR (400 MHz, $CDCl_3$) of benzofuran **37**.

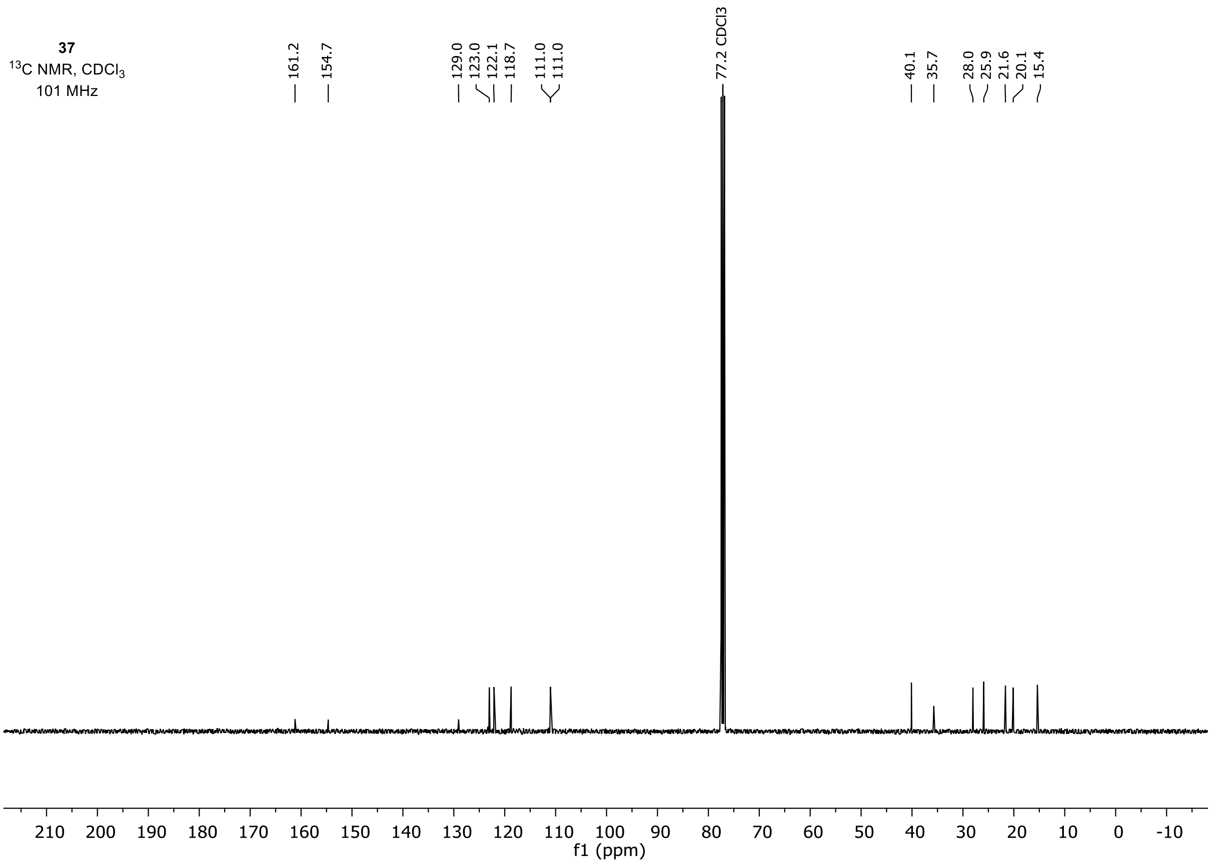

Figure 85. ^{13}C-NMR (101 MHz, $CDCl_3$) of benzofuran **37**.

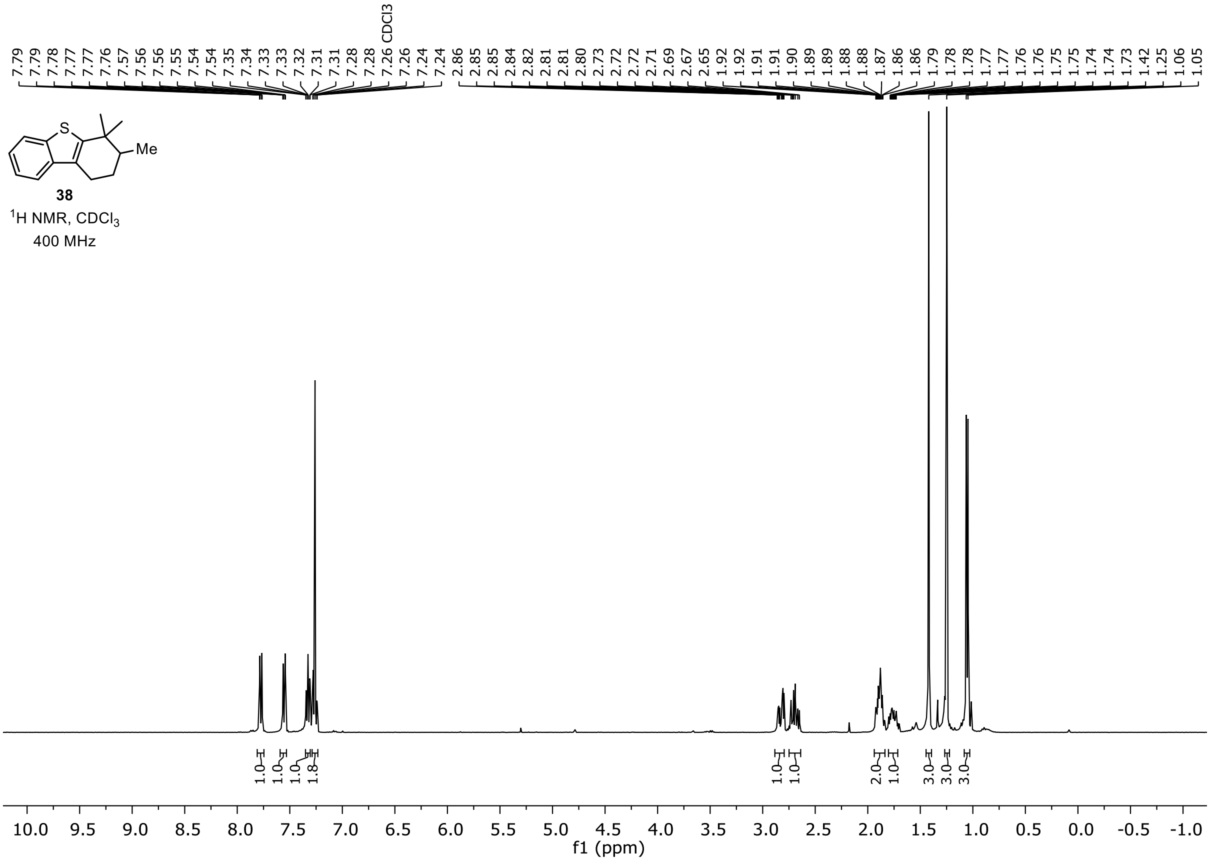

Figure 86. ^{1}H-NMR (400 MHz, $CDCl_3$) of benzothiophene **38**.

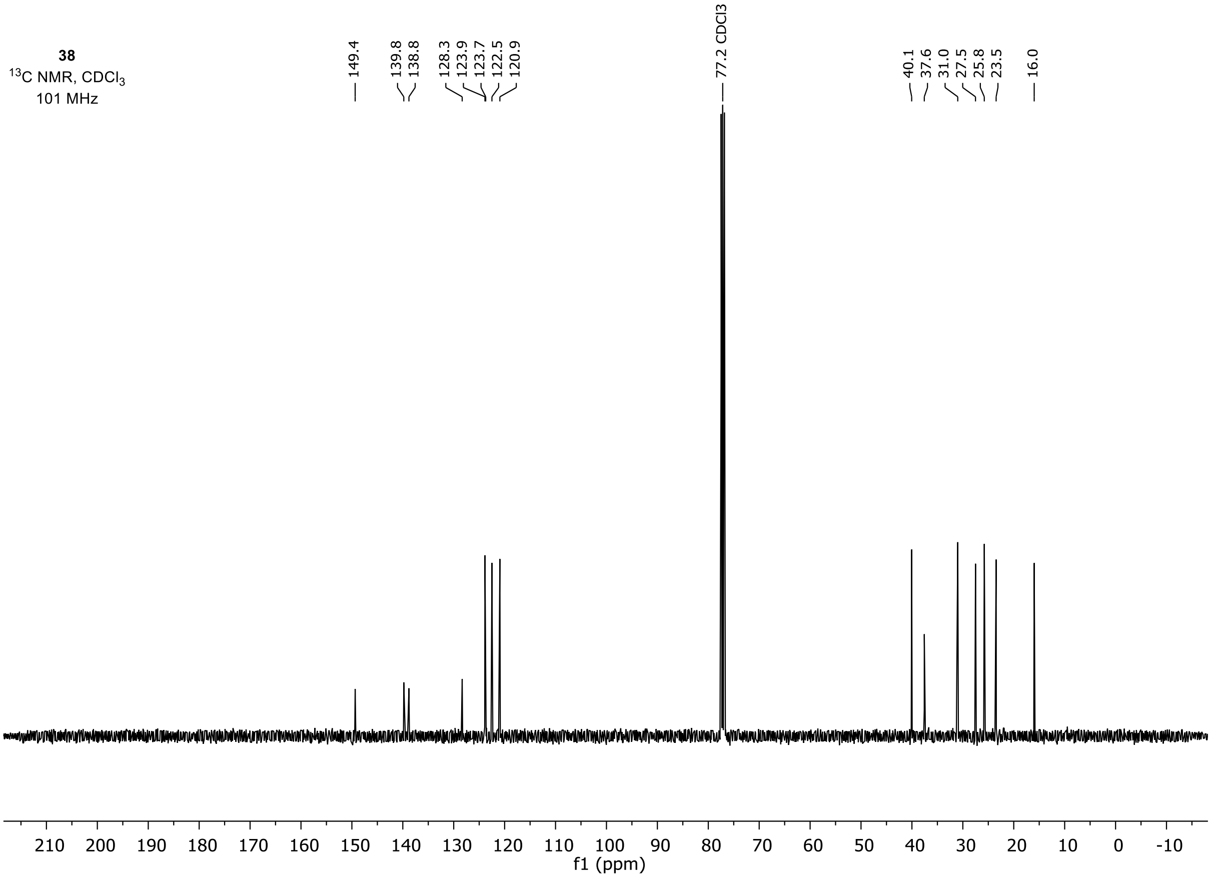

Figure 87. ^{13}C-NMR (101 MHz, $CDCl_3$) of benzothiophene **38**.

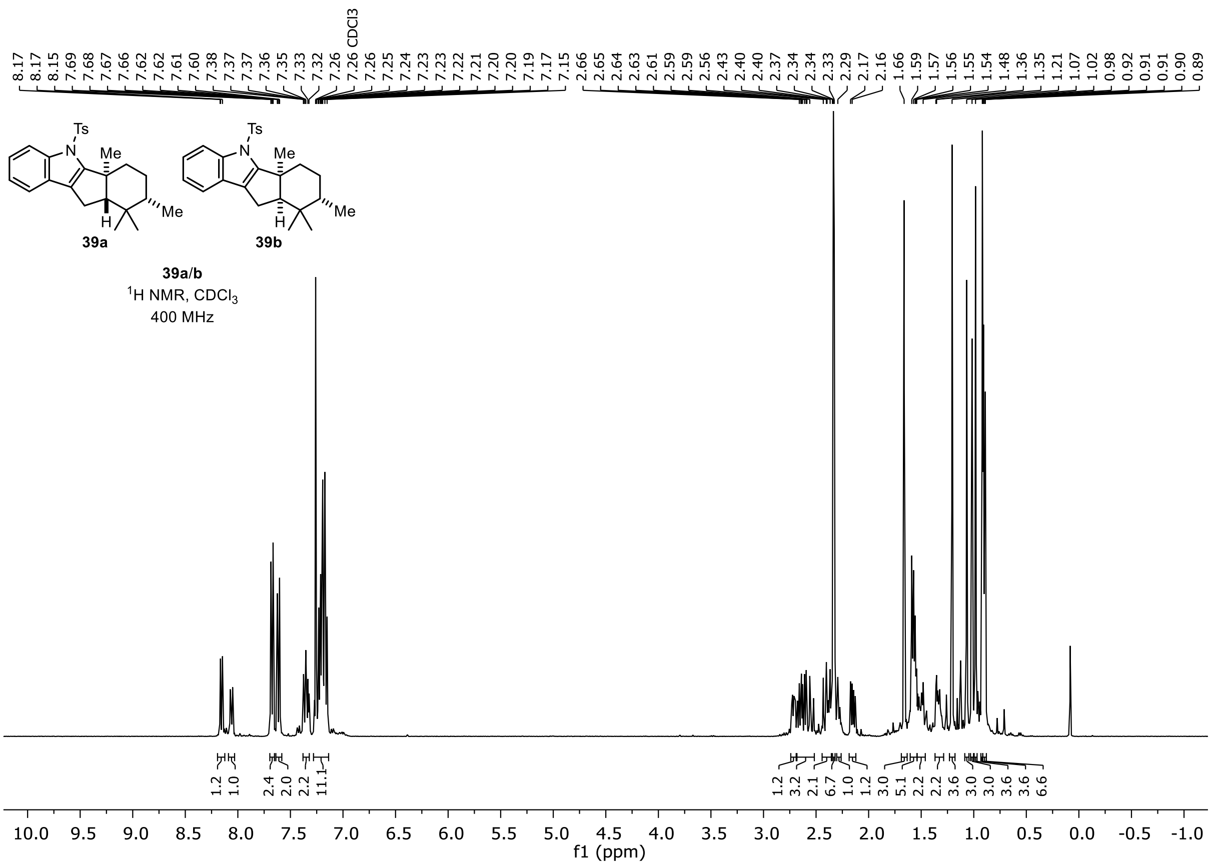

Figure 88. ^{1}H-NMR (400 MHz, $CDCl_3$) of tetracycle **39a/b** (1.2:1.0).

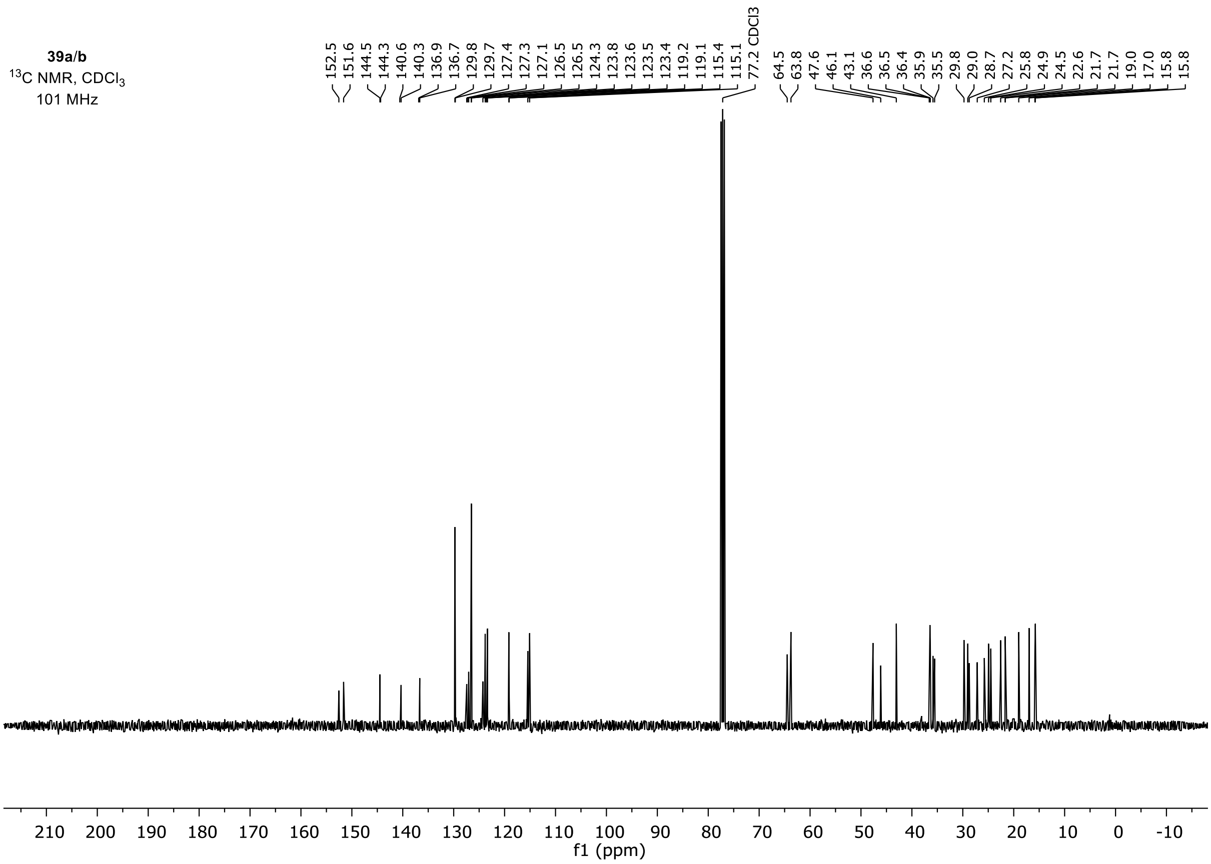

Figure 89. ^{13}C-NMR (101 MHz, $CDCl_3$) of tetracycle **39a/b** (1.2:1.0).

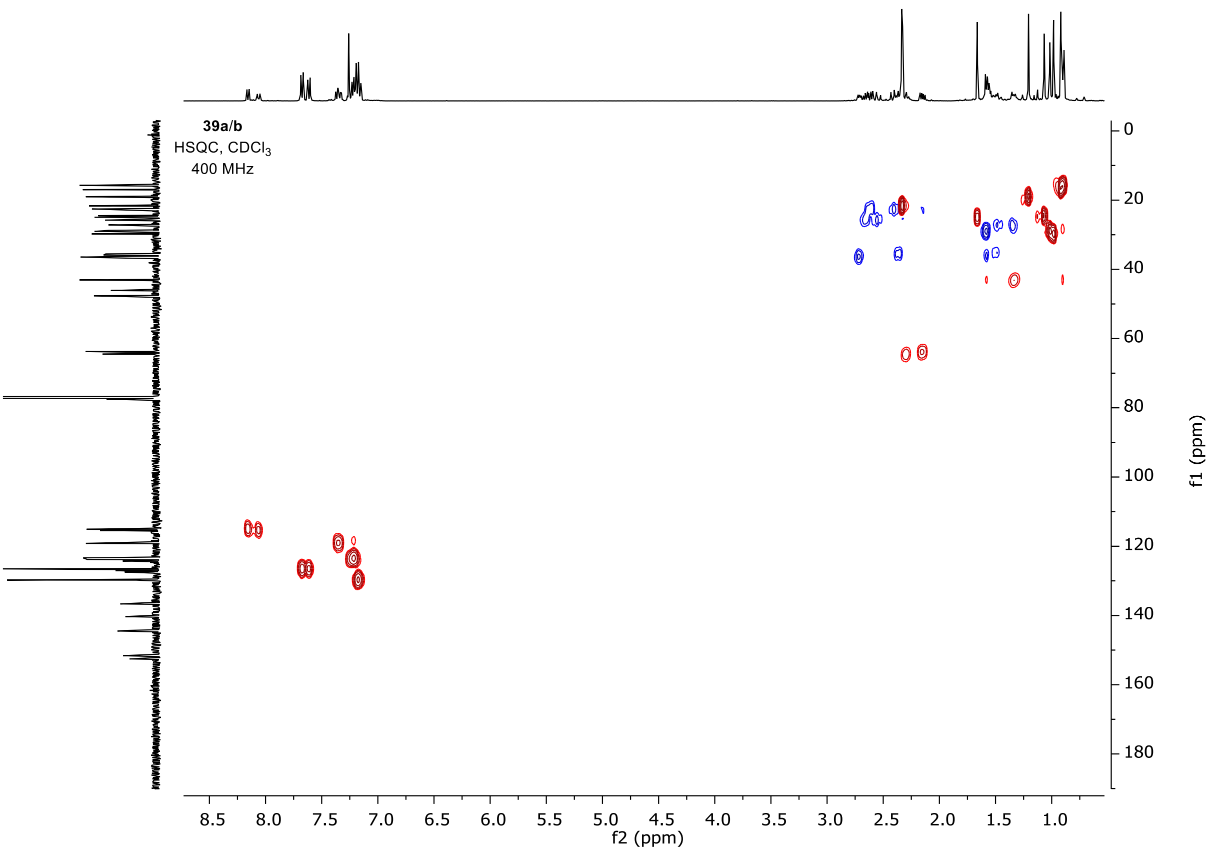

Figure 90. HSQC (400 MHz, $CDCl_3$) of tetracycle **39a/b** (1.2:1.0).

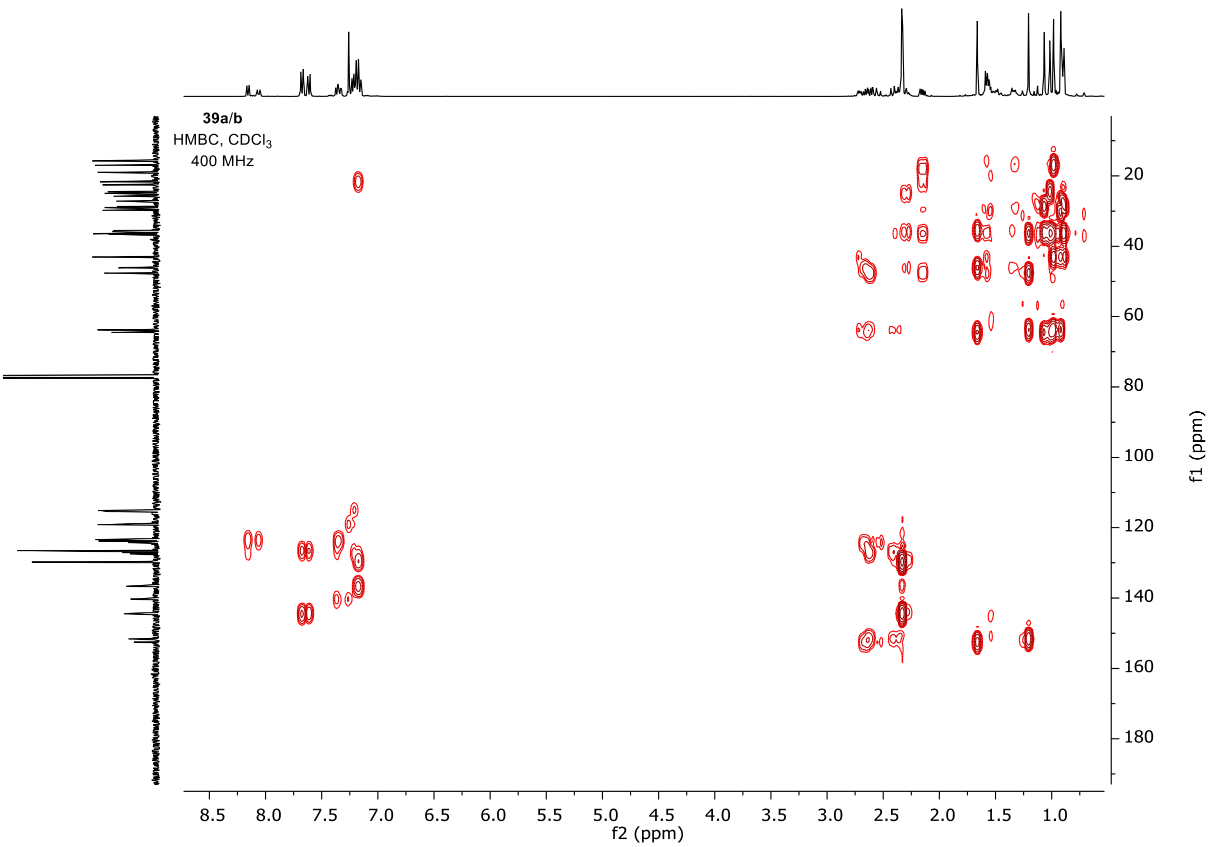

Figure 91. HMBC (400 MHz, $CDCl_3$) of tetracycle **39a/b** (1.2:1.0).

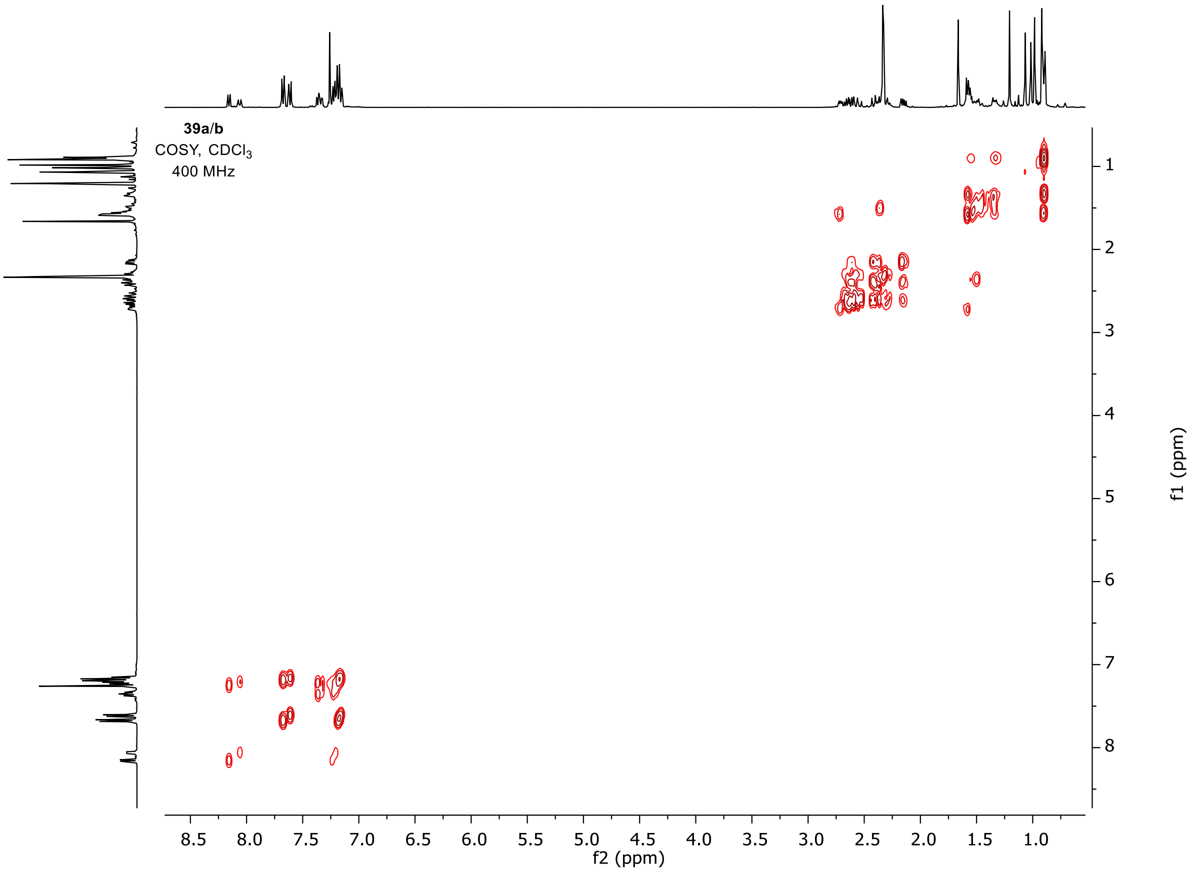

Figure 92. COSY (400 MHz, CDCl3) of tetracycle **39a/b** (1.2:1.0).

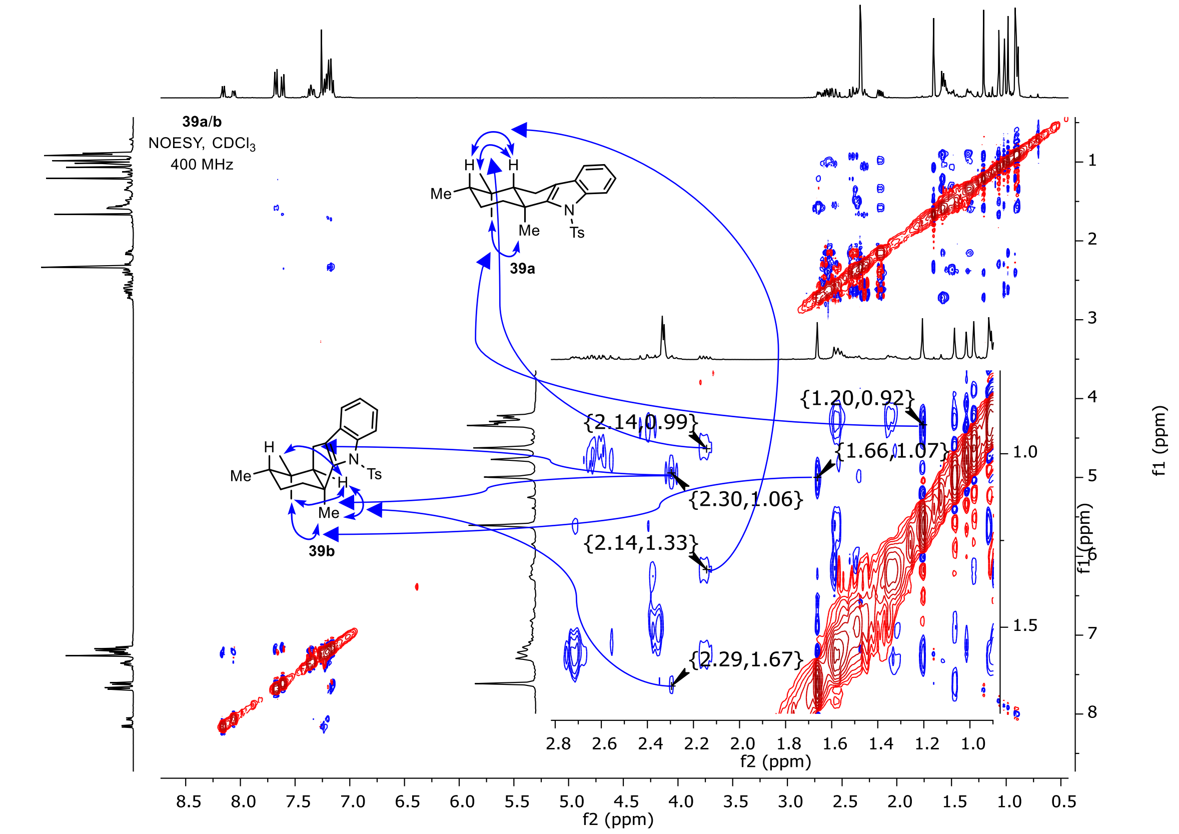

Figure 93. NOESY (400 MHz, CDCl3) of tetracycle **39a/b** (1.2:1.0).

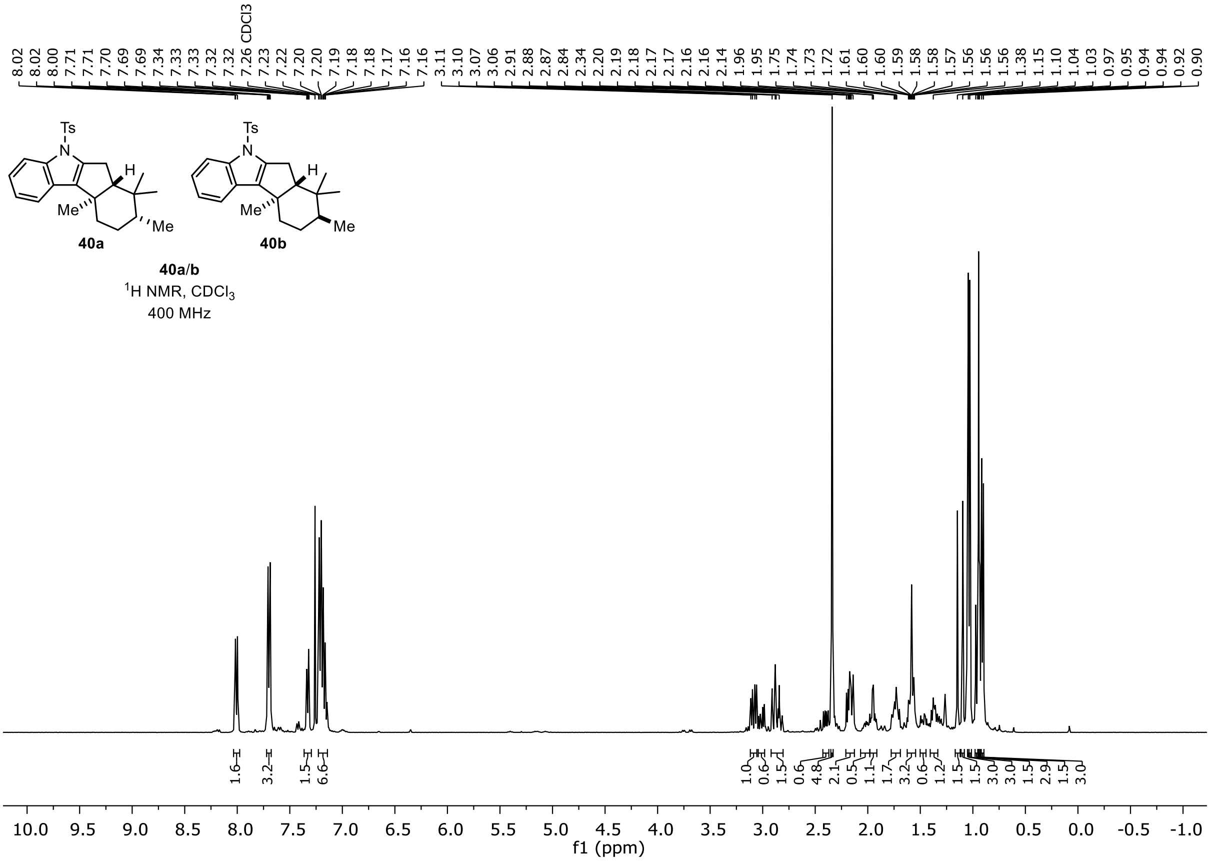

Figure 94. ^{1}H-NMR (400 MHz, $CDCl_3$) of tetracycle **40a/b** (1:0.6).

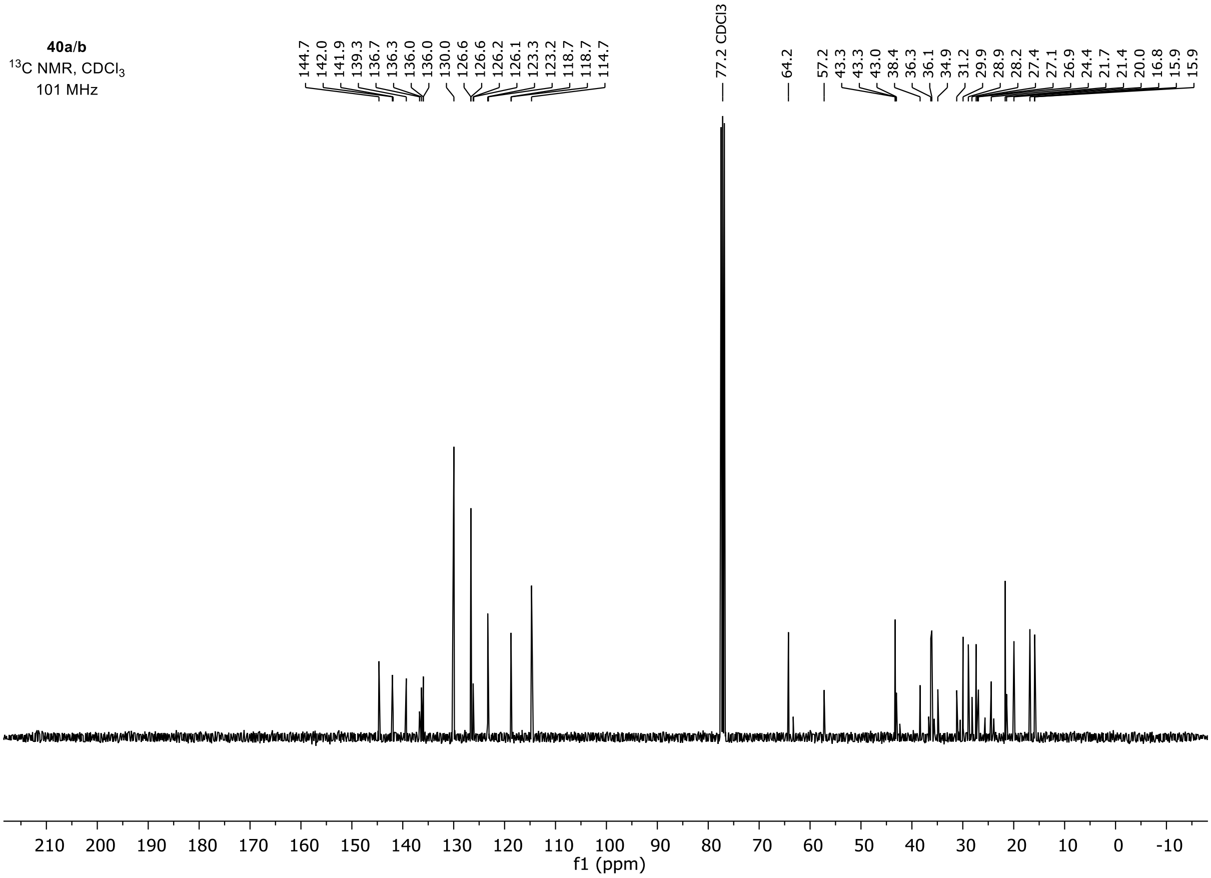

Figure 95. ^{13}C-NMR (101 MHz, $CDCl_3$) of tetracycle **40a/b** (1:0.6).

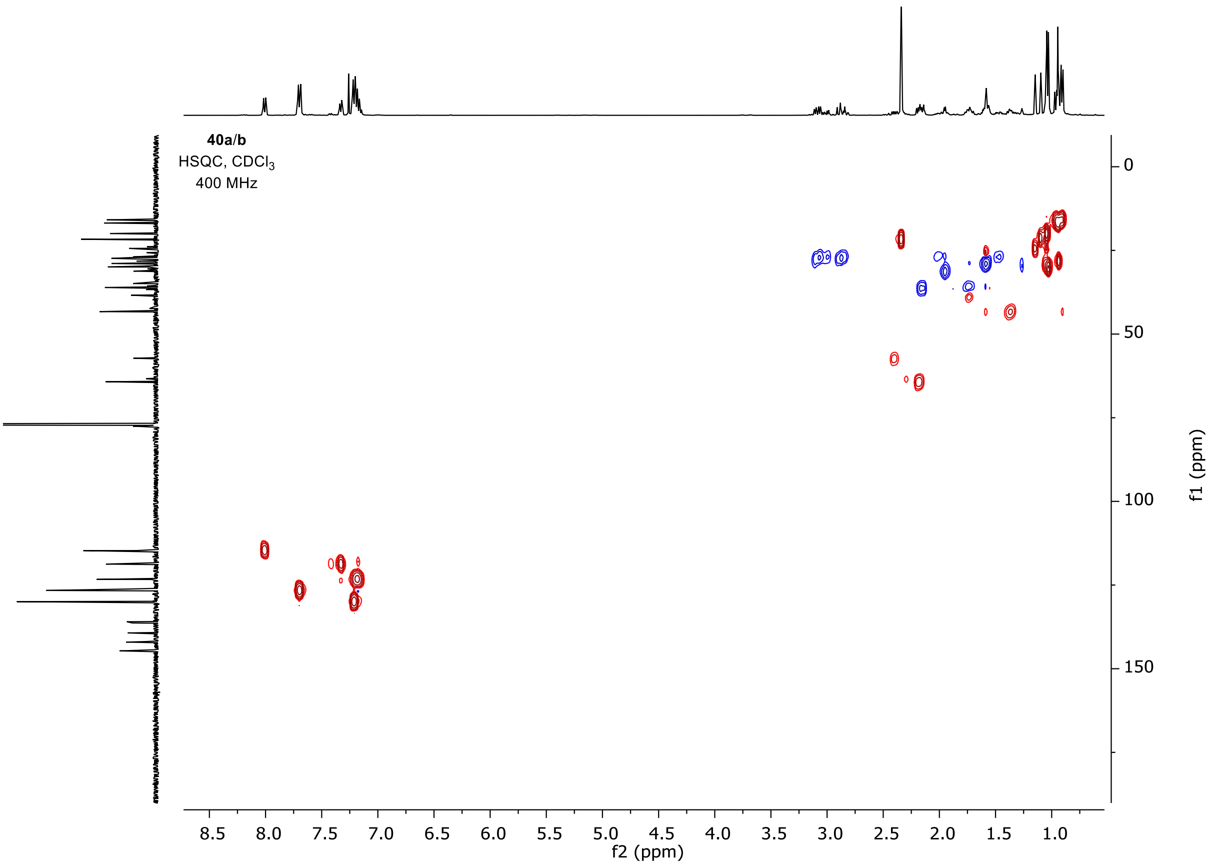

Figure 96. HSQC (400 MHz, $CDCl_3$) of tetracycle **40a/b** (1:0.6).

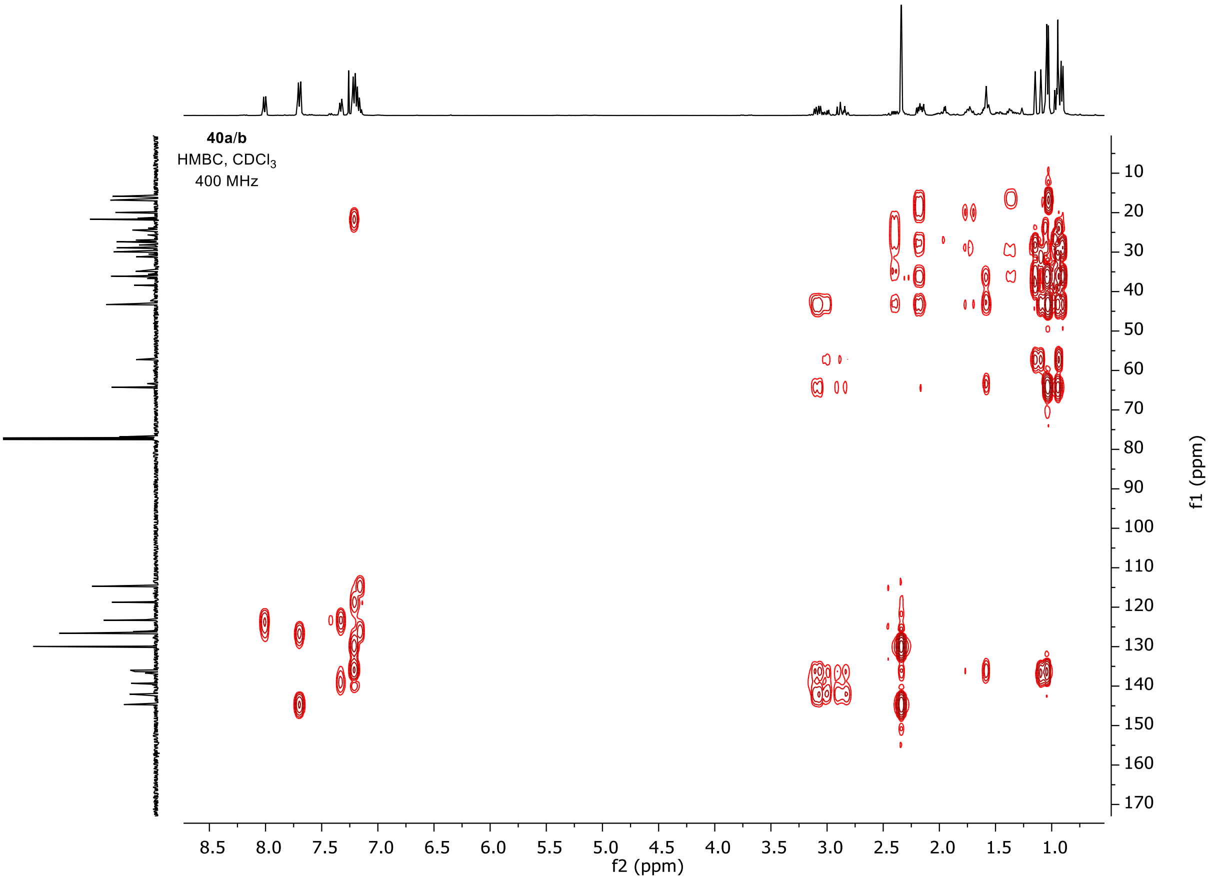

Figure 97. HMBC (400 MHz, $CDCl_3$) of tetracycle **40a/b** (1:0.6).

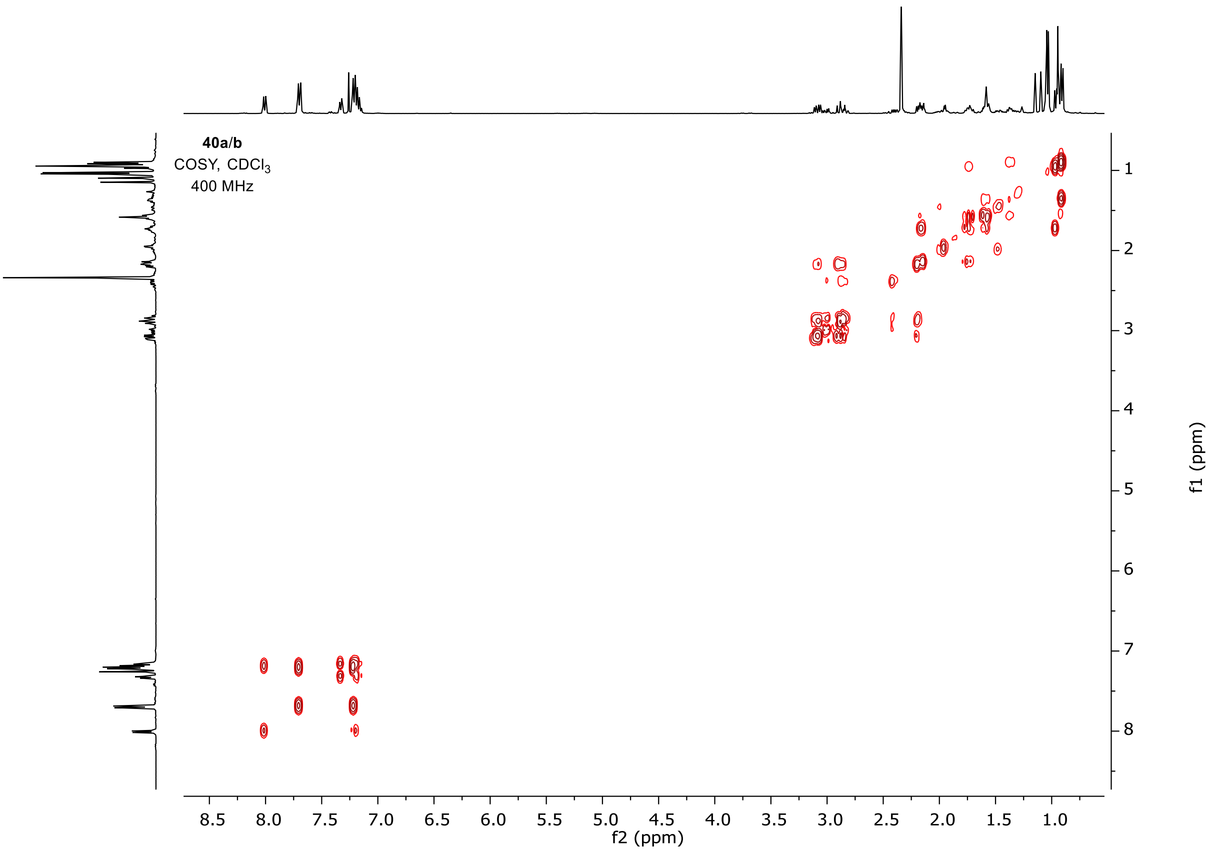

Figure 98. COSY (400 MHz, CDCl3) of tetracycle **40a/b** (1:0.6).

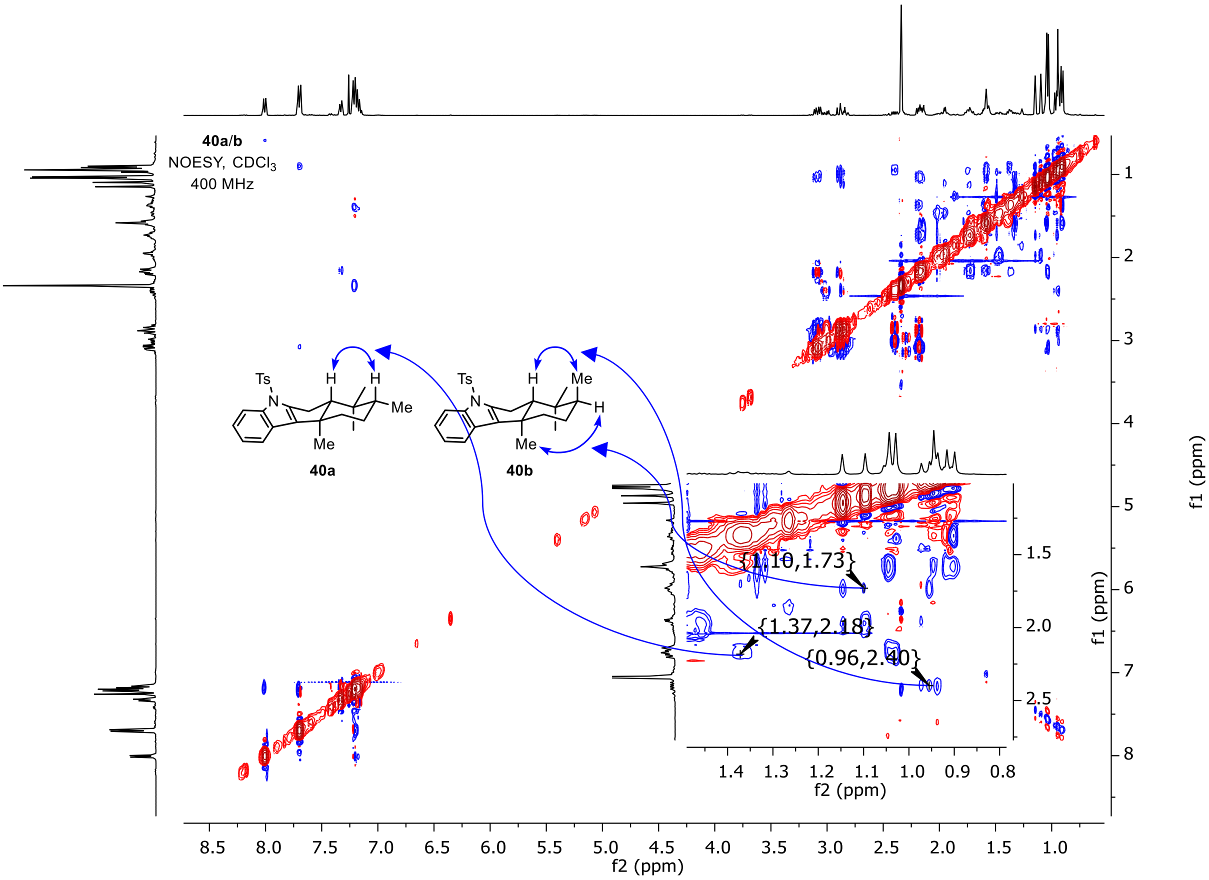

Figure 99. NOESY (400 MHz, CDCl3) of tetracycle **40a/b** (1:0.6).

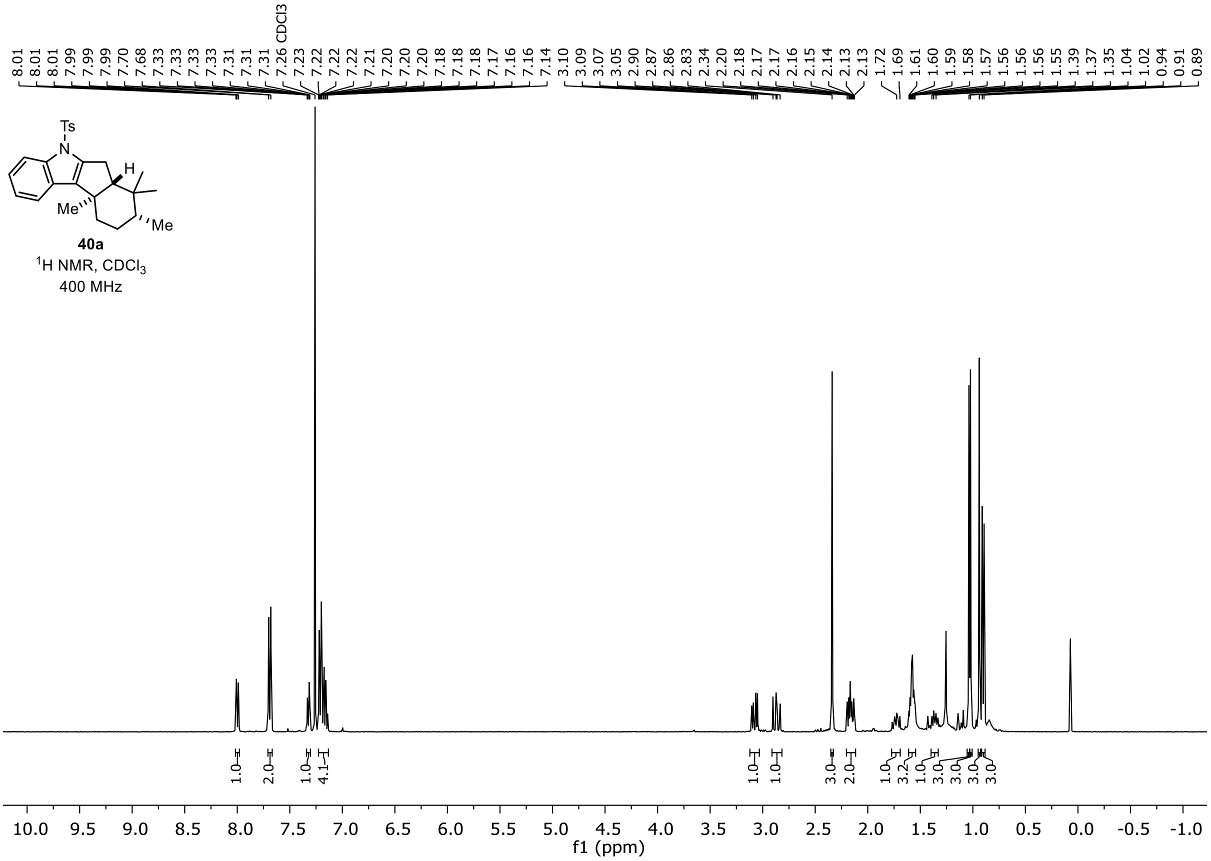

Figure 100. ^{1}H-NMR (400 MHz, $CDCl_3$) of tetracycle **40a**.

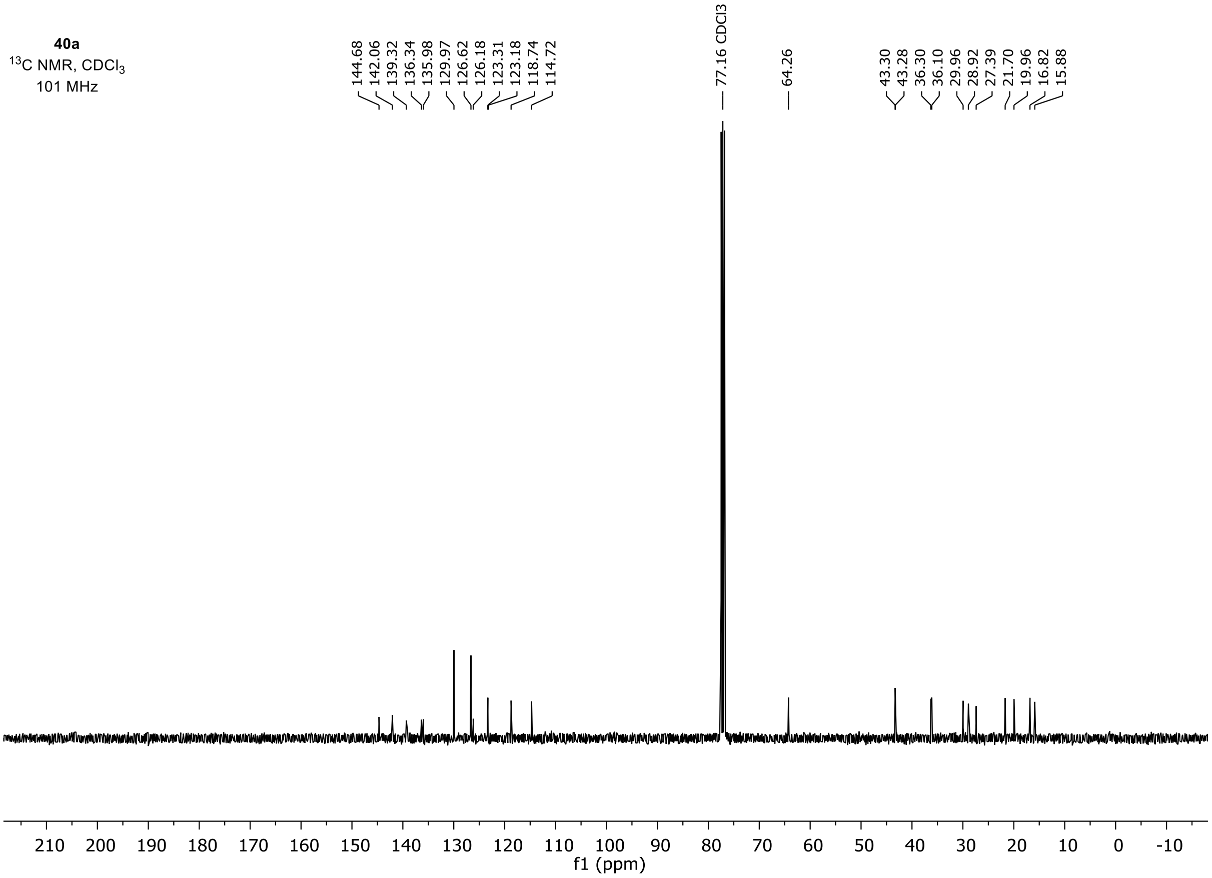

Figure 101. ^{13}C-NMR (101 MHz, $CDCl_3$) of tetracycle **40a**.

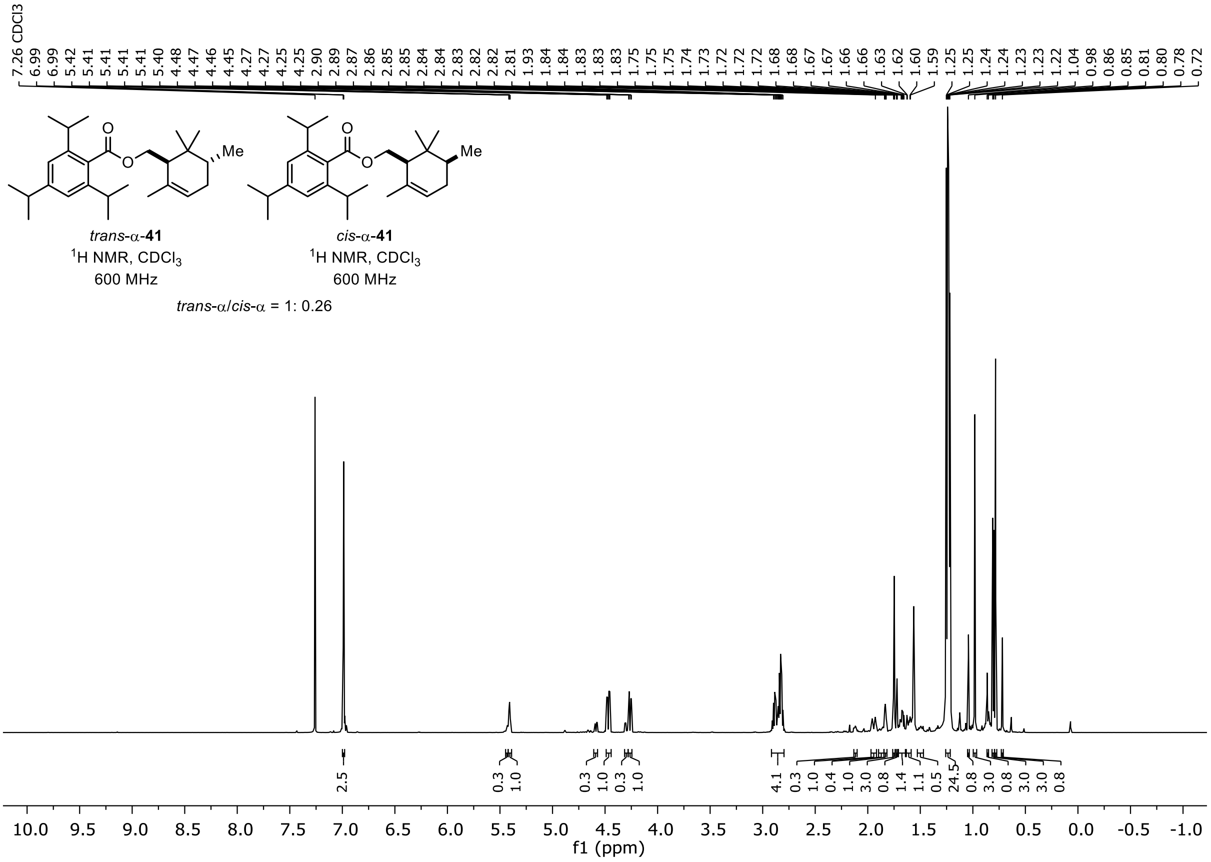

Figure 102. ^{1}H-NMR (600 MHz, $CDCl_3$) of cyclohexene *trans*-α-**41**/*cis*-α-**41** (1:0.26).

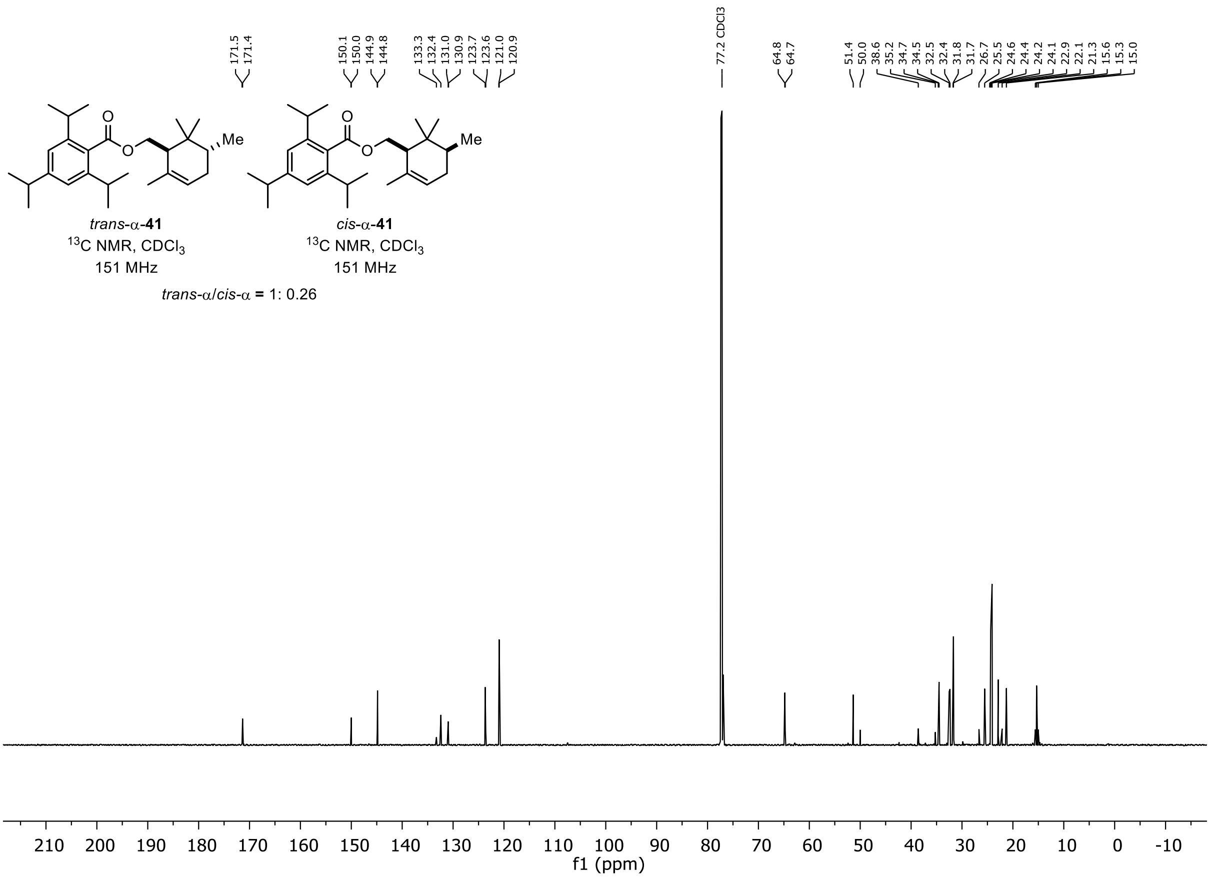

Figure 103. ^{13}C-NMR (151 MHz, $CDCl_3$) of cyclohexene *trans*-α-**41**/*cis*-α-**41** (1:0.26).

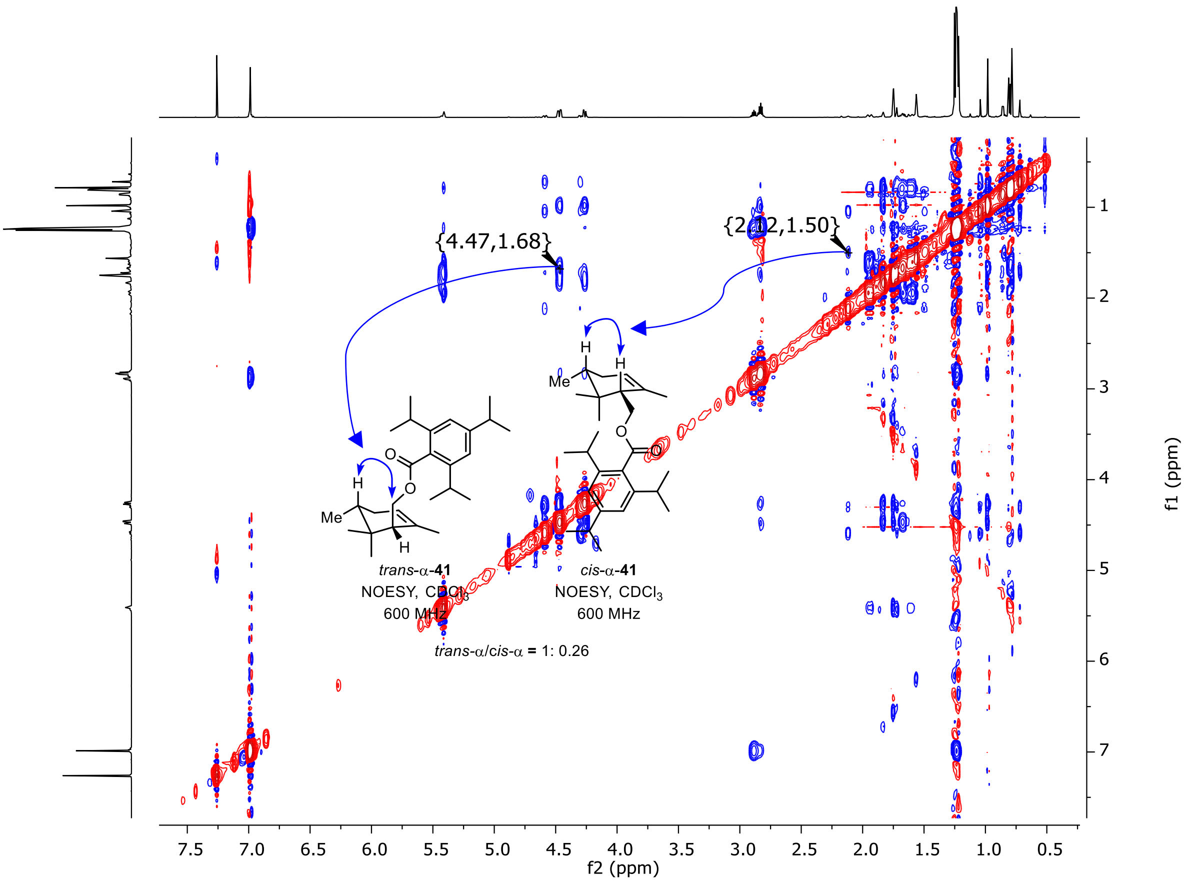

Figure 104. NOESY (600 MHz, $CDCl_3$) of cyclohexene *trans*-α-**41**/*cis*-α-**41** (1:0.26).

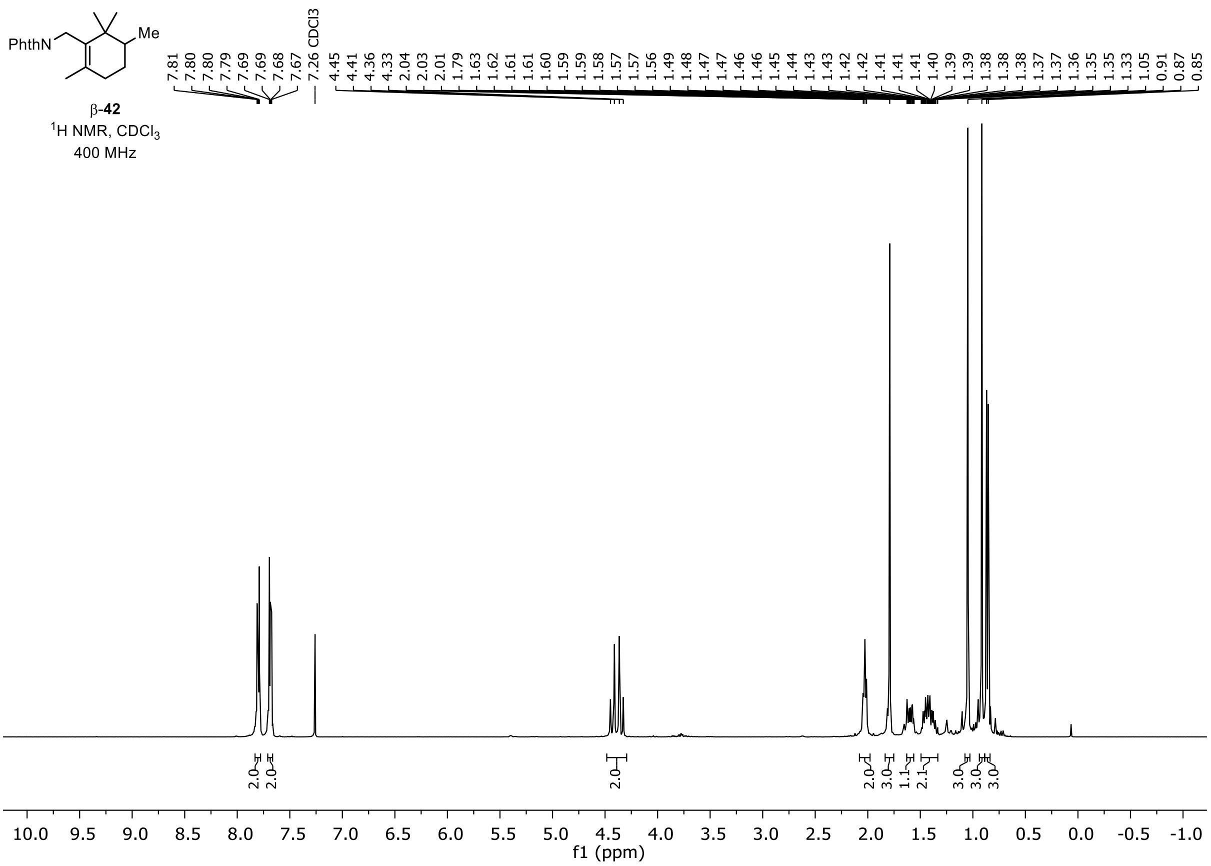

Figure 105. ^{1}H-NMR (400 MHz, $CDCl_3$) of cyclohexene β-**42**.

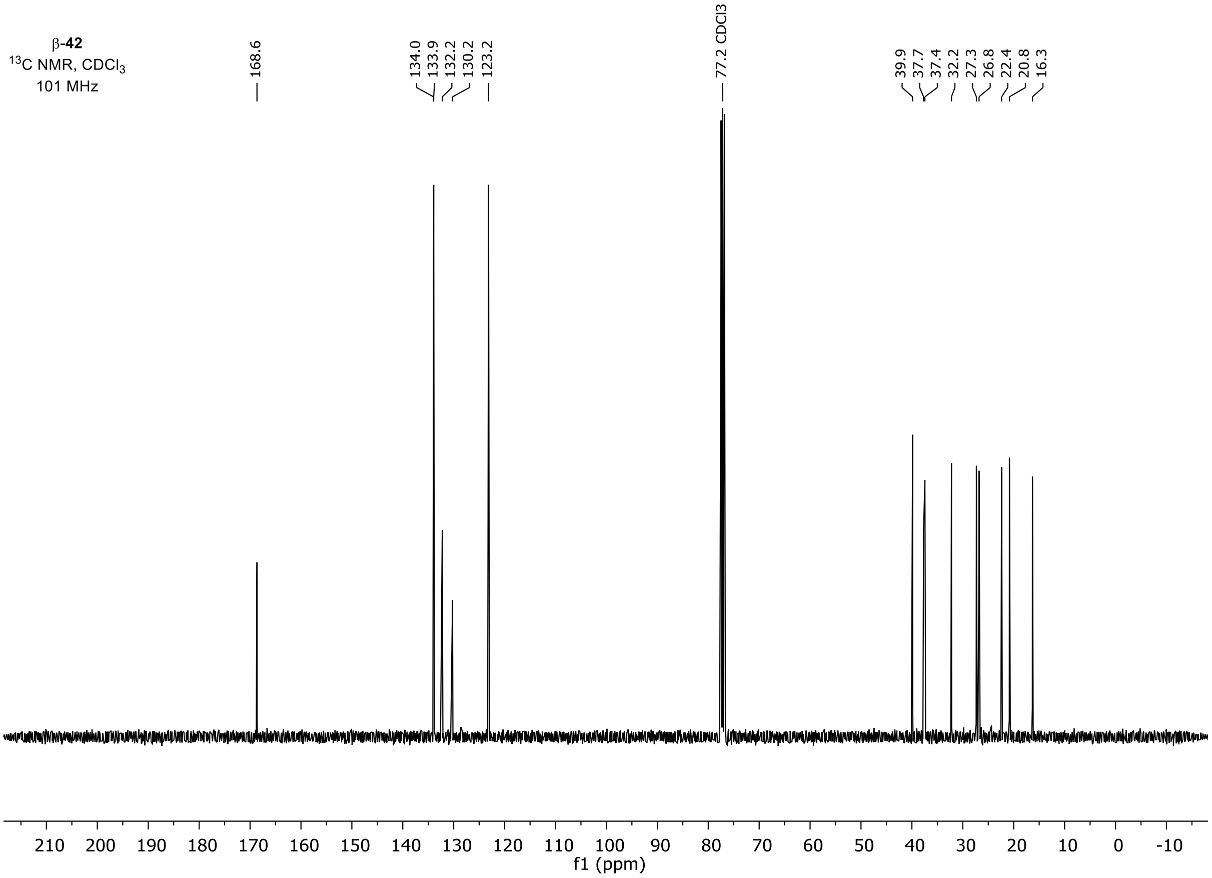

Figure 106. ^{13}C-NMR (101 MHz, $CDCl_3$) of cyclohexene β-**42**.

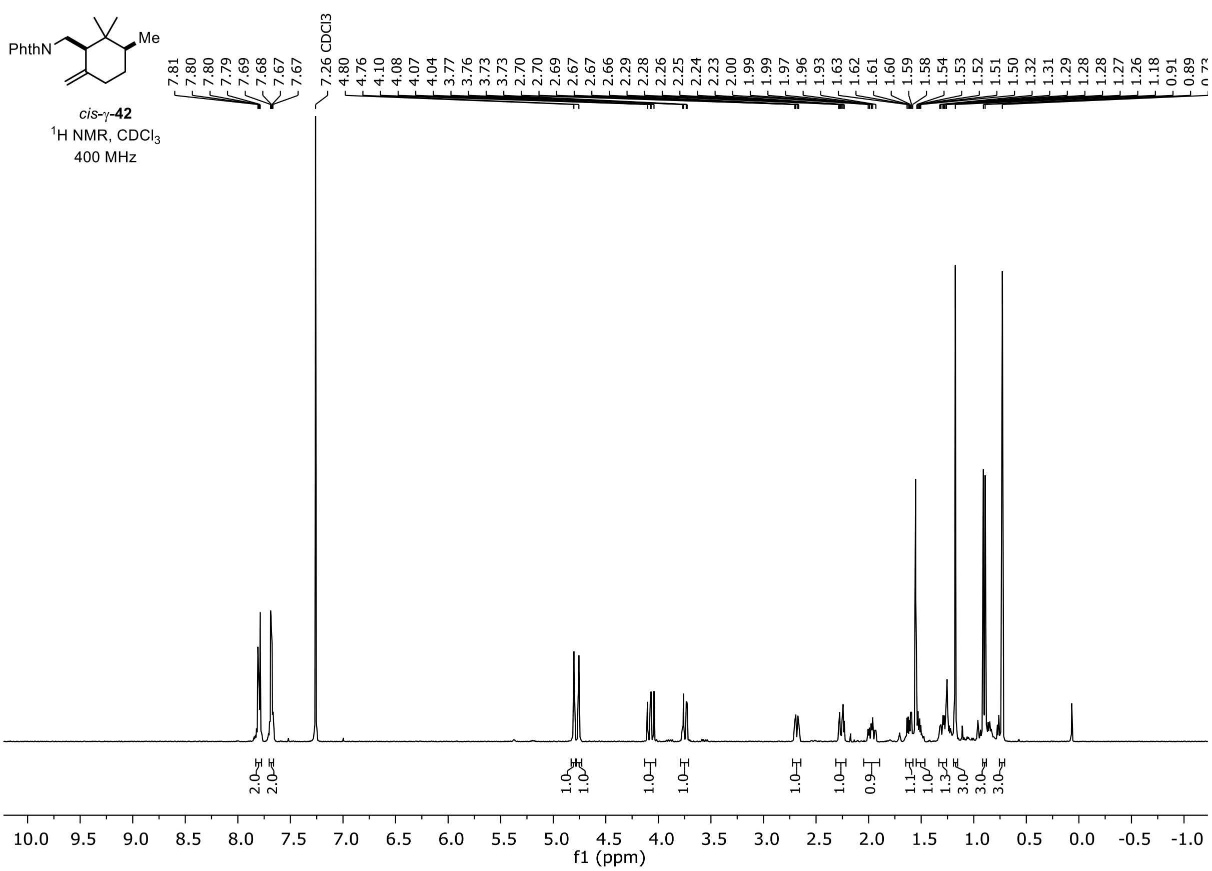

Figure 107. ^{1}H-NMR (400 MHz, $CDCl_3$) of cyclohexene *cis*-γ-**42**.

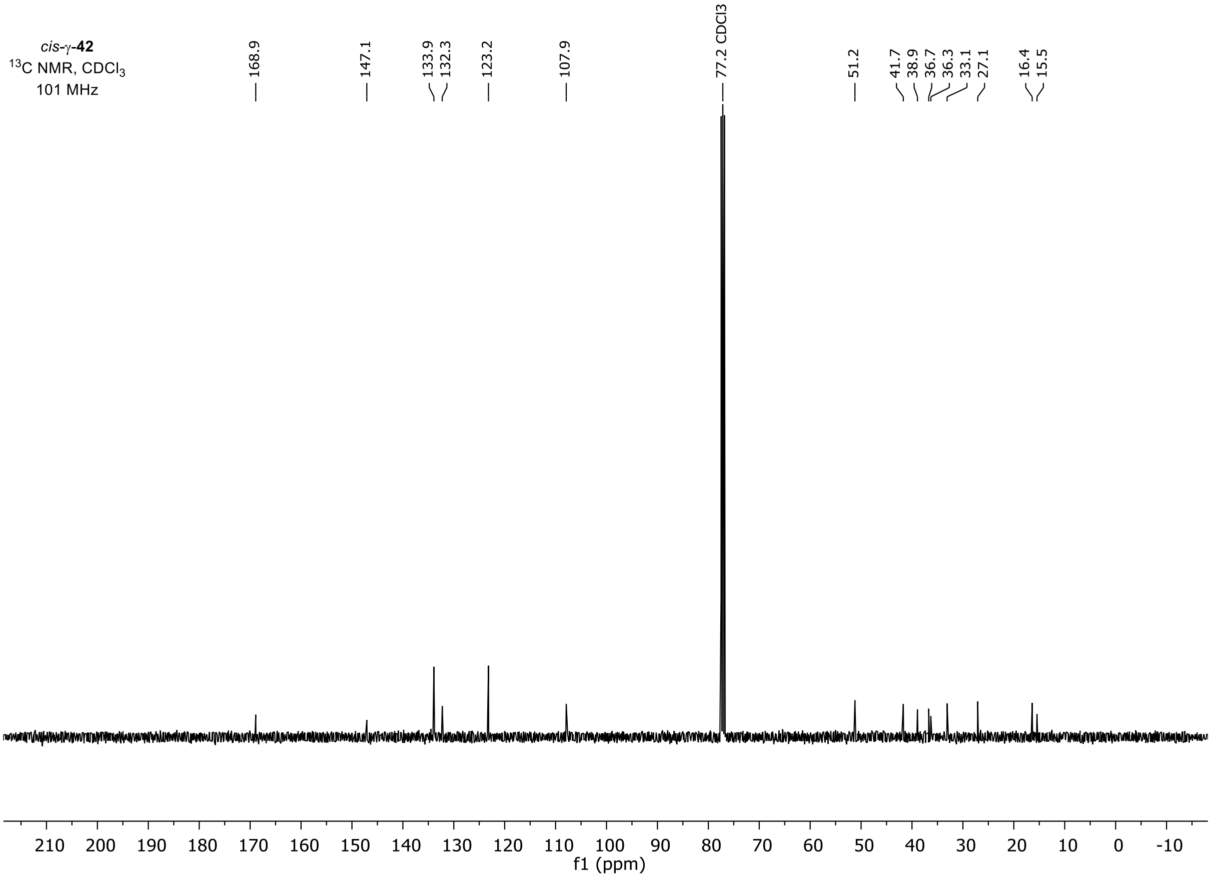

Figure 108. ^{13}C-NMR (101 MHz, $CDCl_3$) of cyclohexene *cis*-γ-**42**.

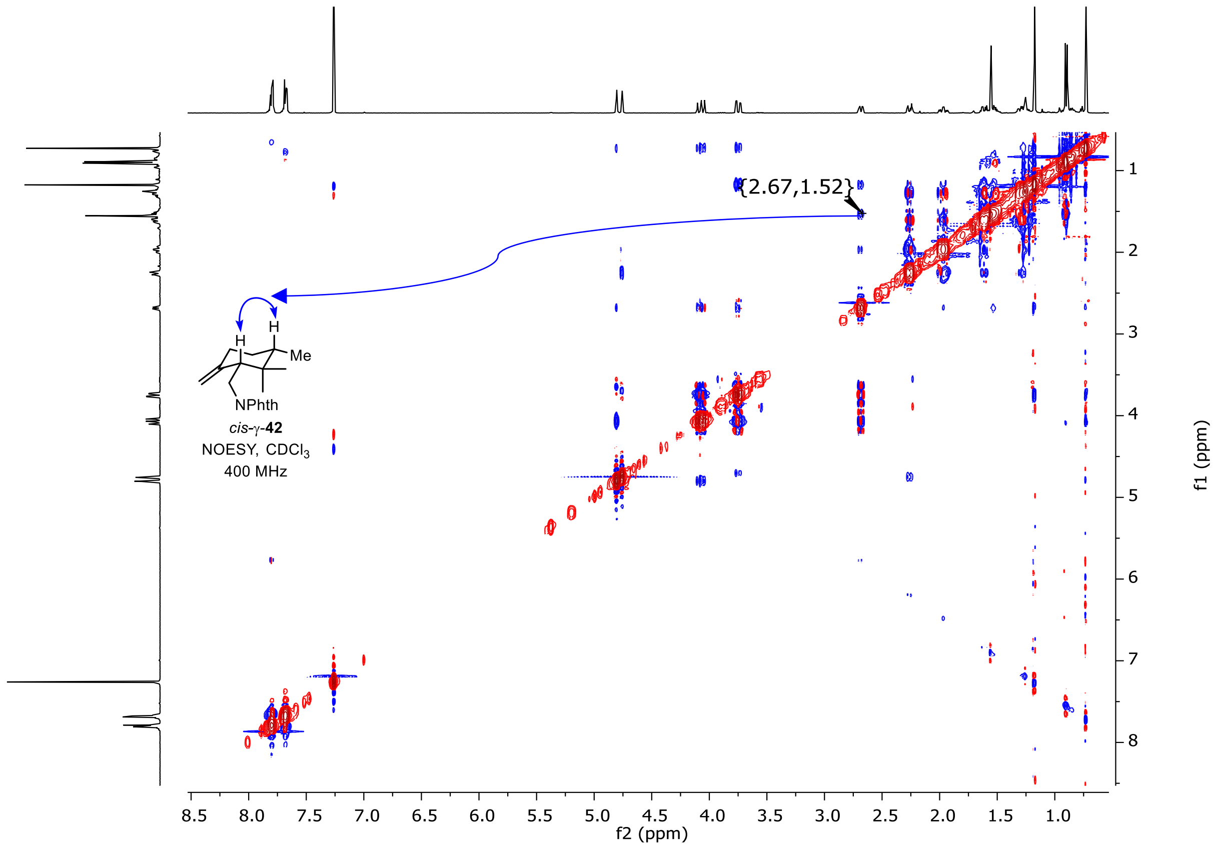

Figure 109. NOESY (400 MHz, $CDCl_3$) of cyclohexene *cis*-γ-**42**.

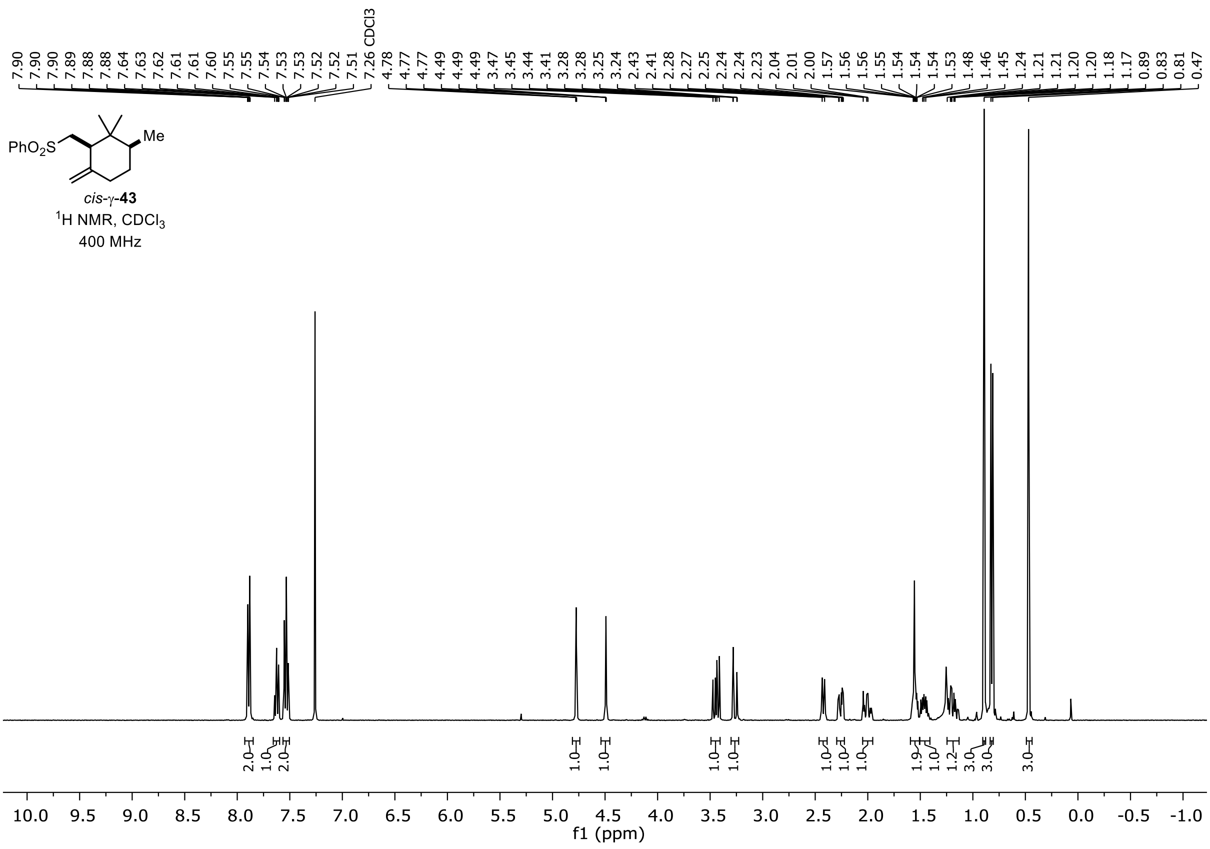

Figure 110. ^{1}H-NMR (400 MHz, $CDCl_3$) of cyclohexene *cis*-γ-**43**.

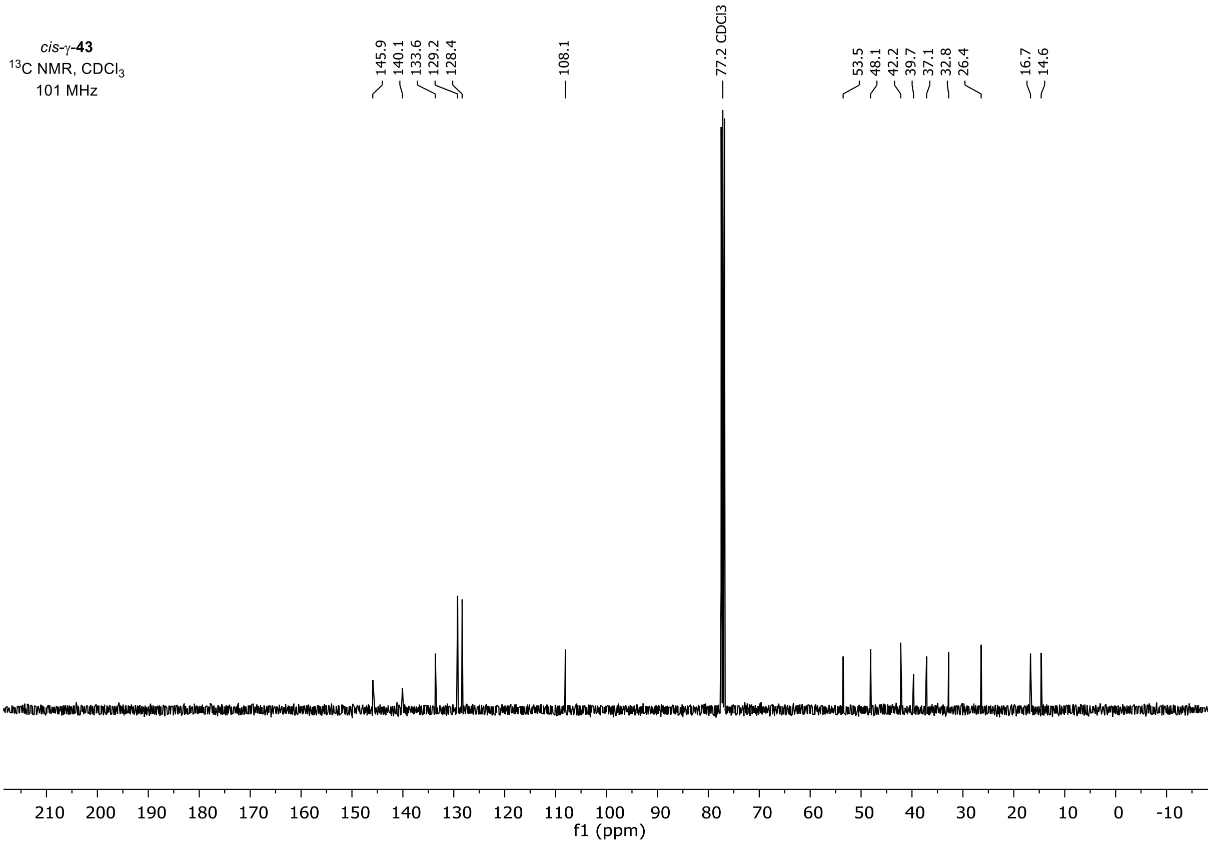

Figure 111. ^{13}C-NMR (101 MHz, $CDCl_3$) of cyclohexene *cis*-γ-**43**.

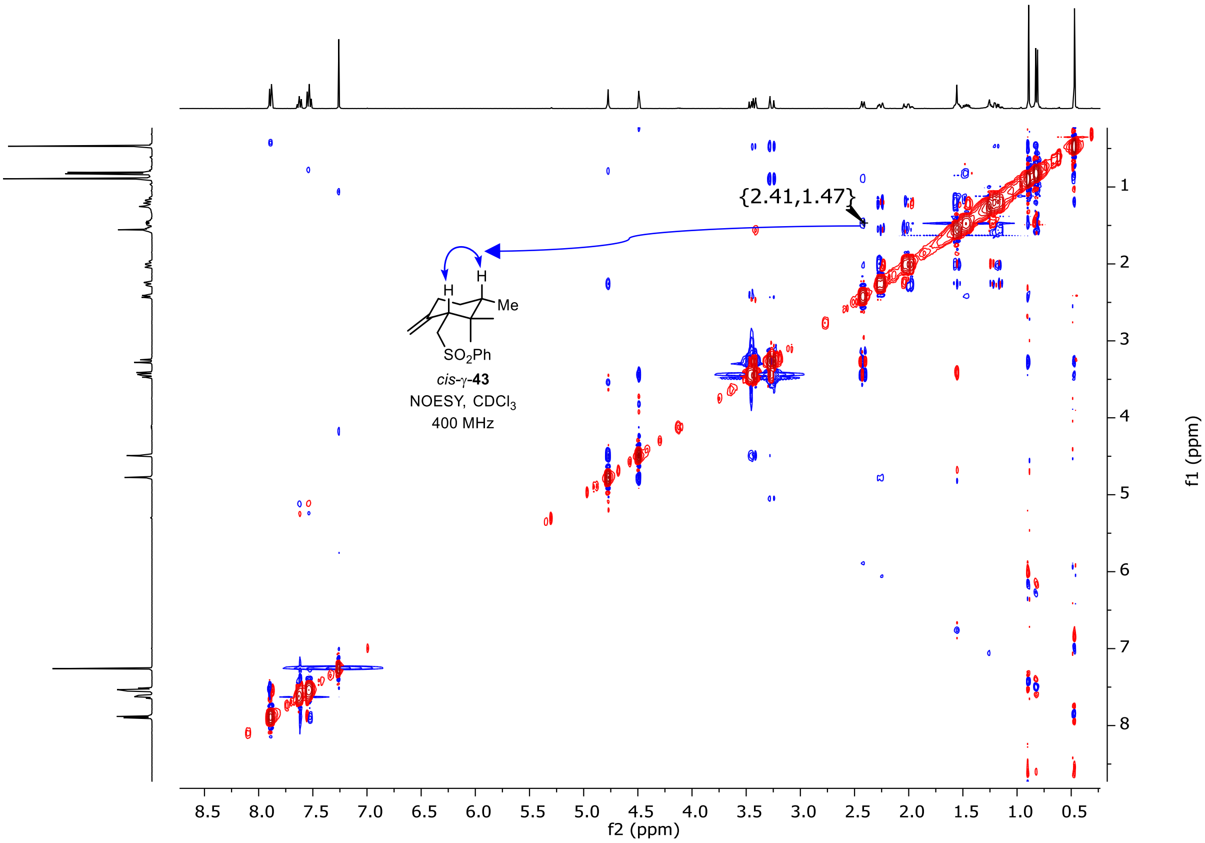

Figure 112. NOESY (400 MHz, $CDCl_3$) of cyclohexene *cis*-γ-**43**.

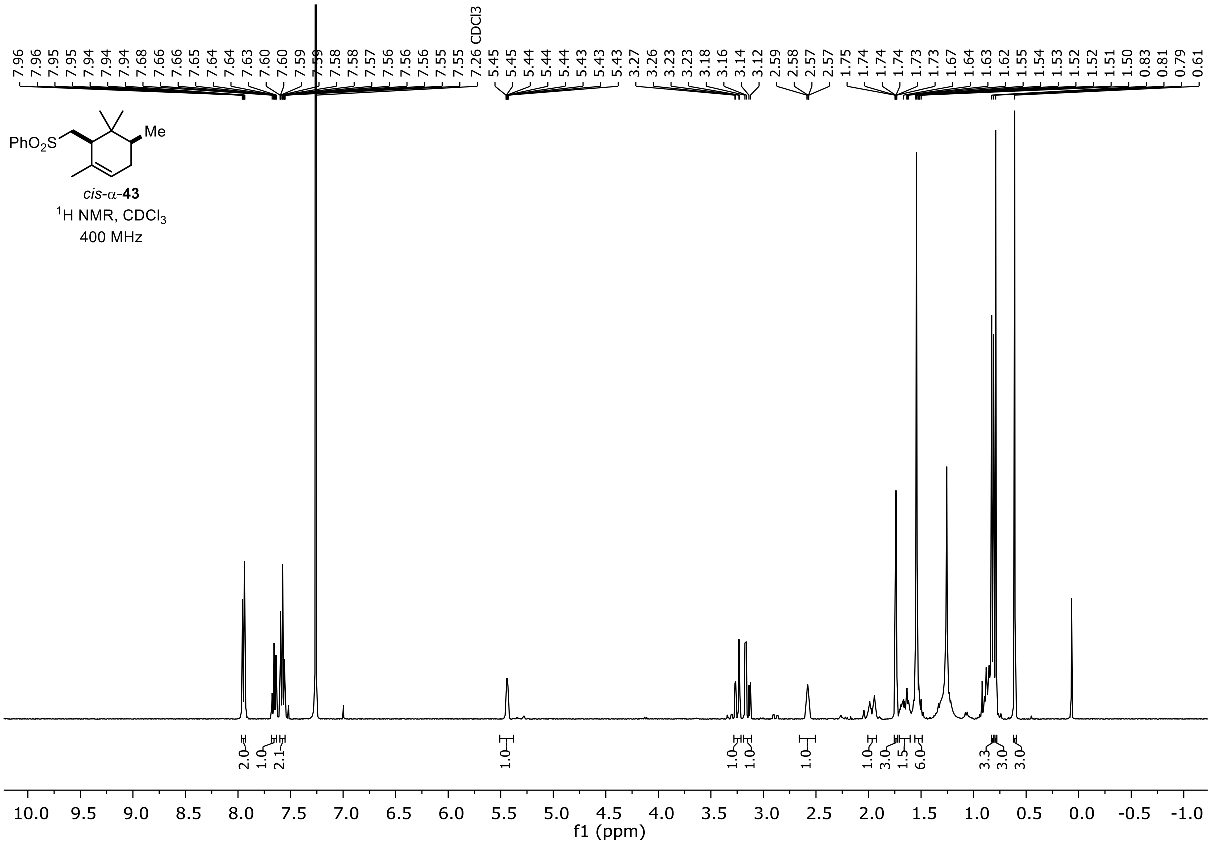

Figure 113. ^{1}H-NMR (400 MHz, $CDCl_3$) of cyclohexene *cis*-α-**43**.

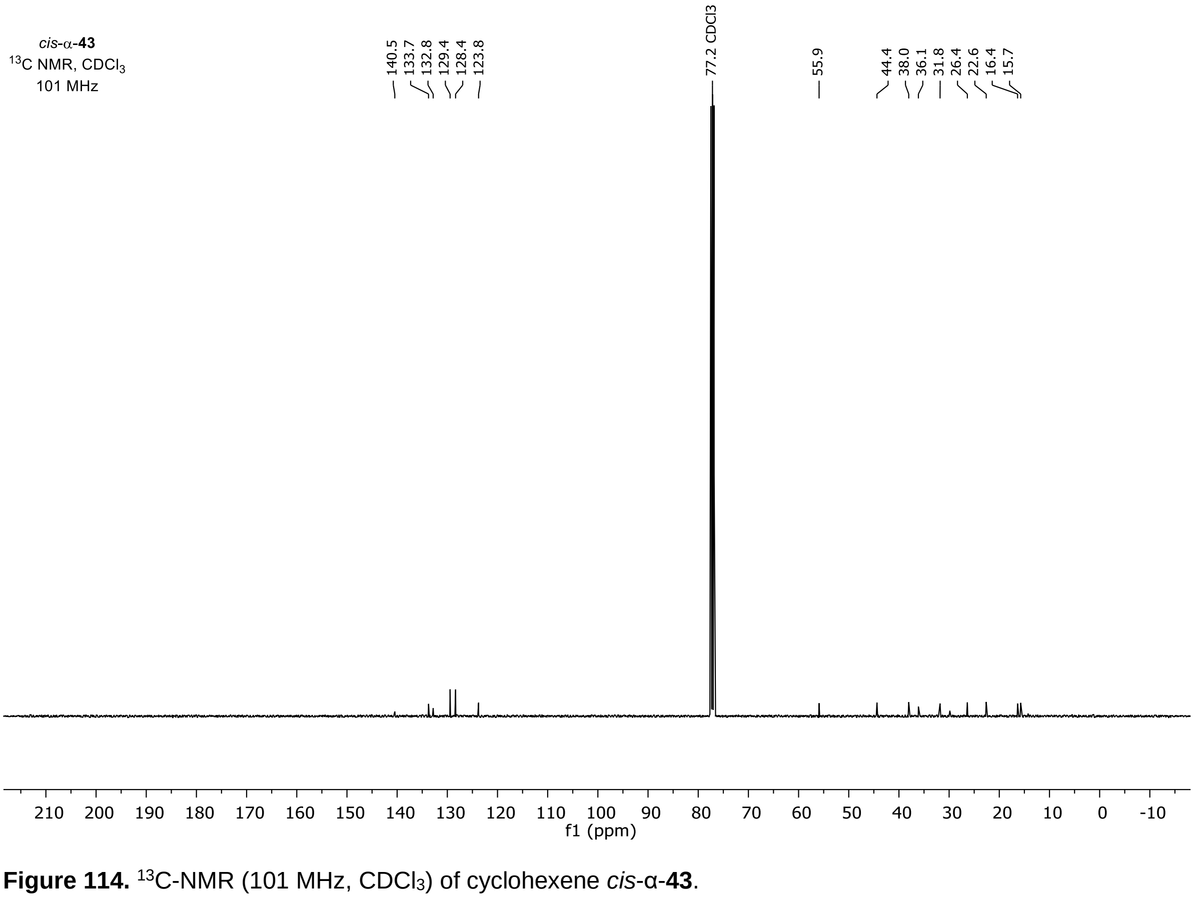

Figure 114. ^{13}C-NMR (101 MHz, $CDCl_3$) of cyclohexene *cis*-α-**43**.

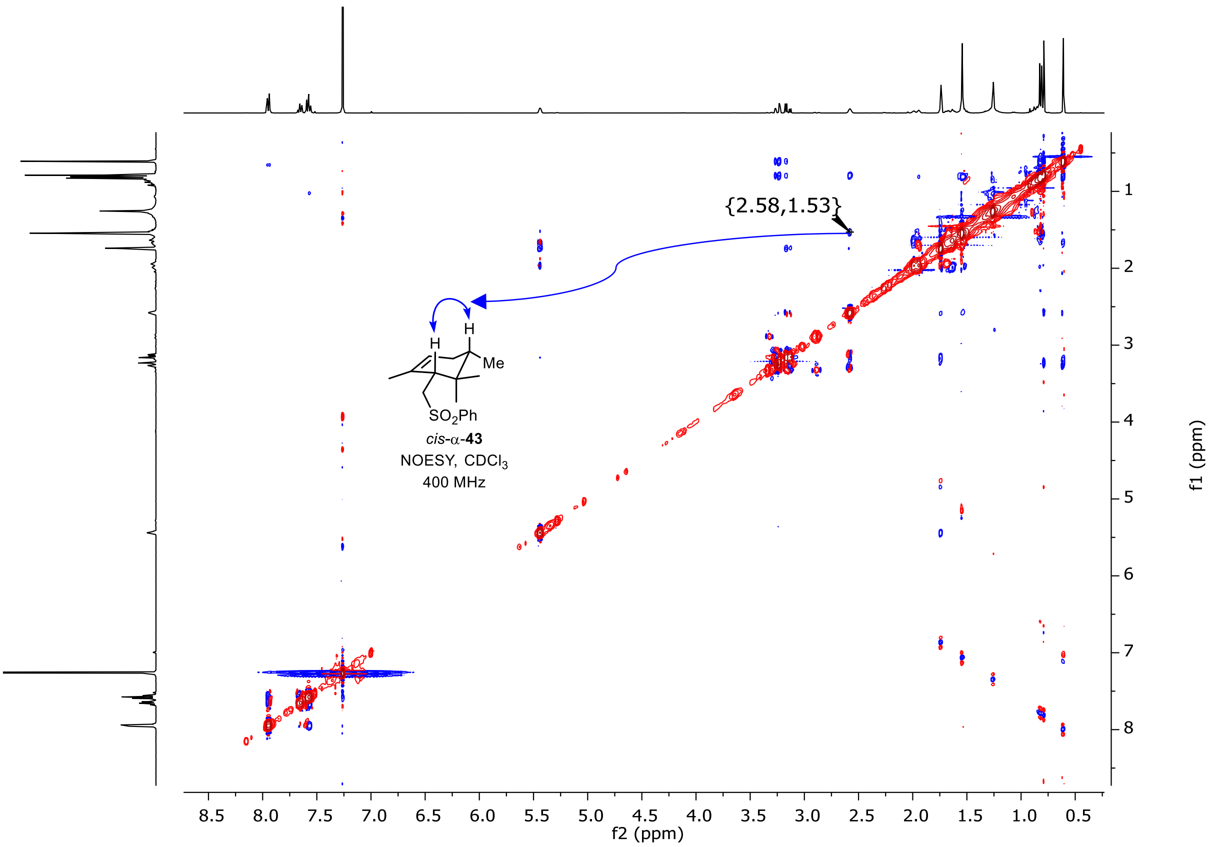

Figure 115. NOESY (400 MHz, $CDCl_3$) of cyclohexene *cis*-α-**43**.

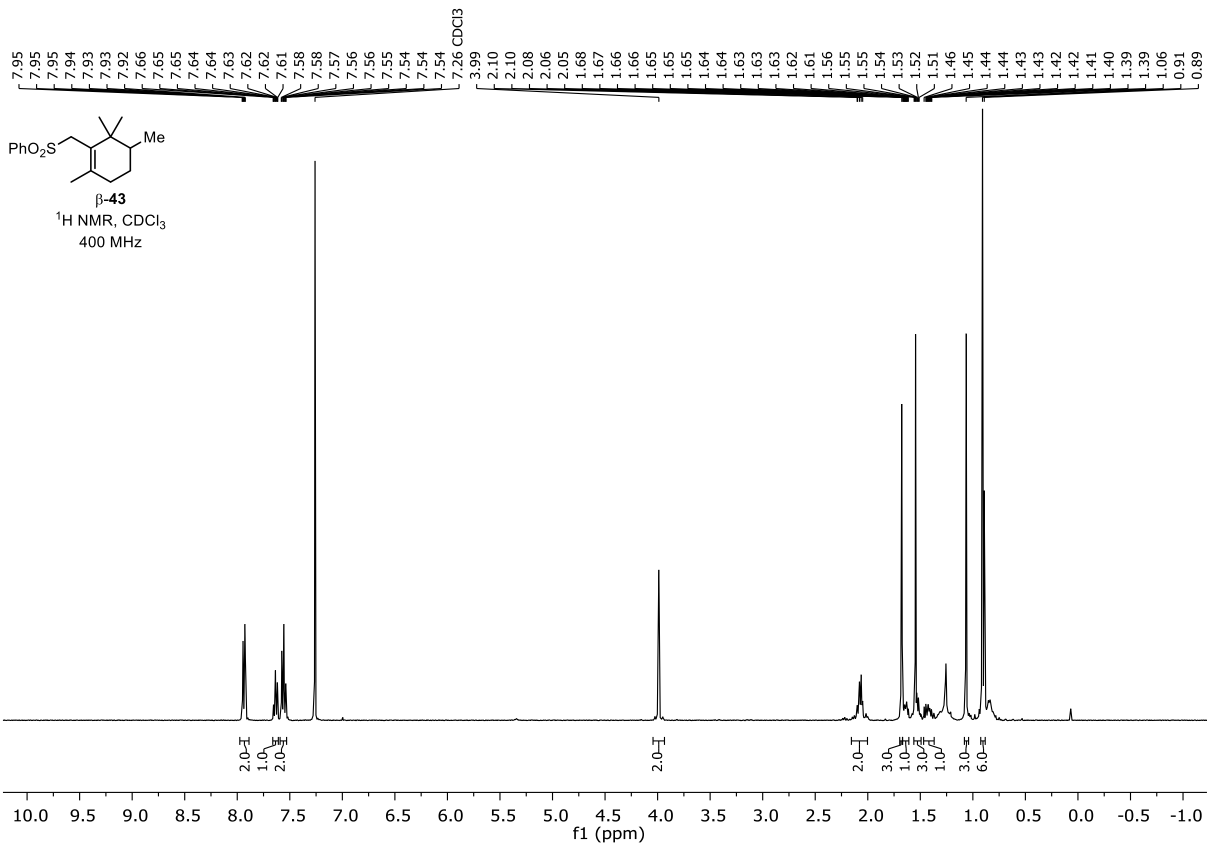

Figure 116. ^{1}H-NMR (400 MHz, $CDCl_3$) of cyclohexene β-**43**.

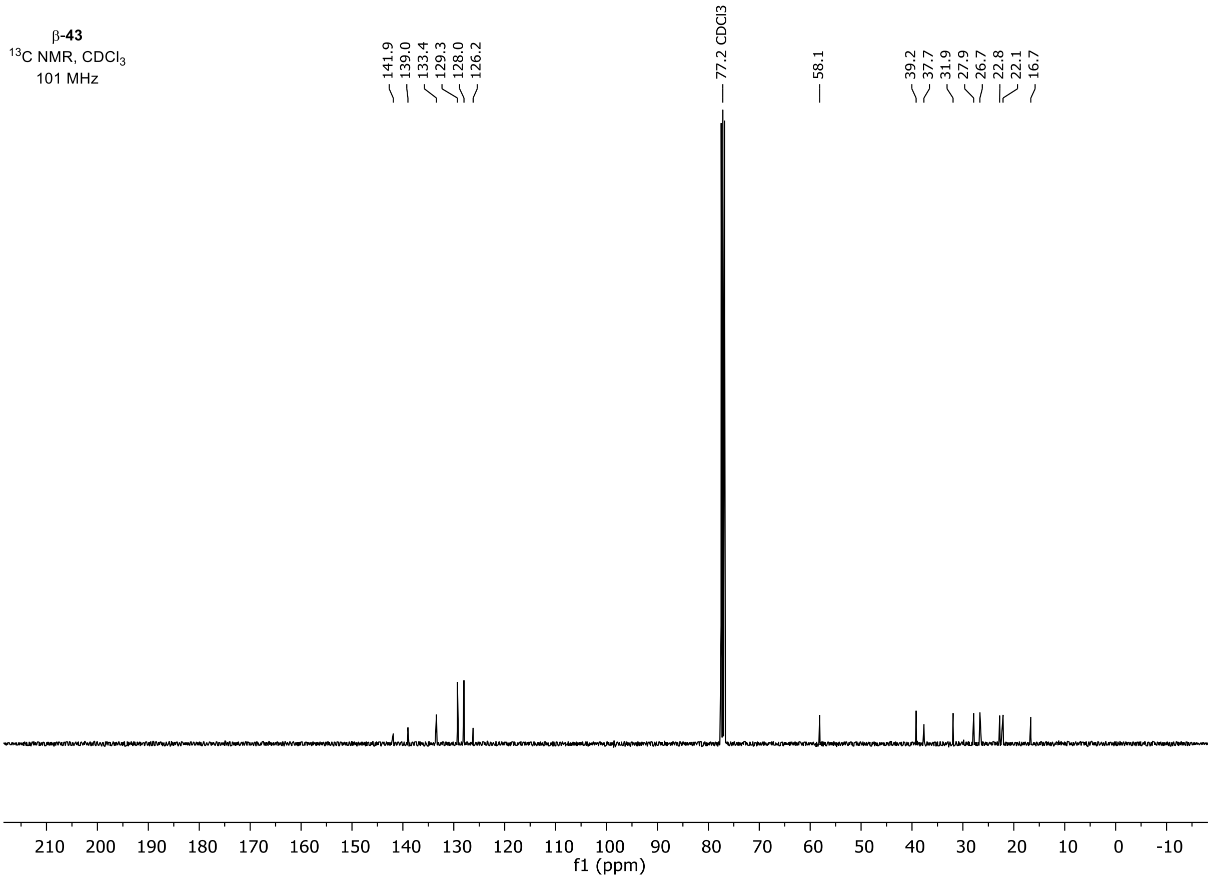

Figure 117. ^{13}C-NMR (101 MHz, $CDCl_3$) of cyclohexene β-**43**.

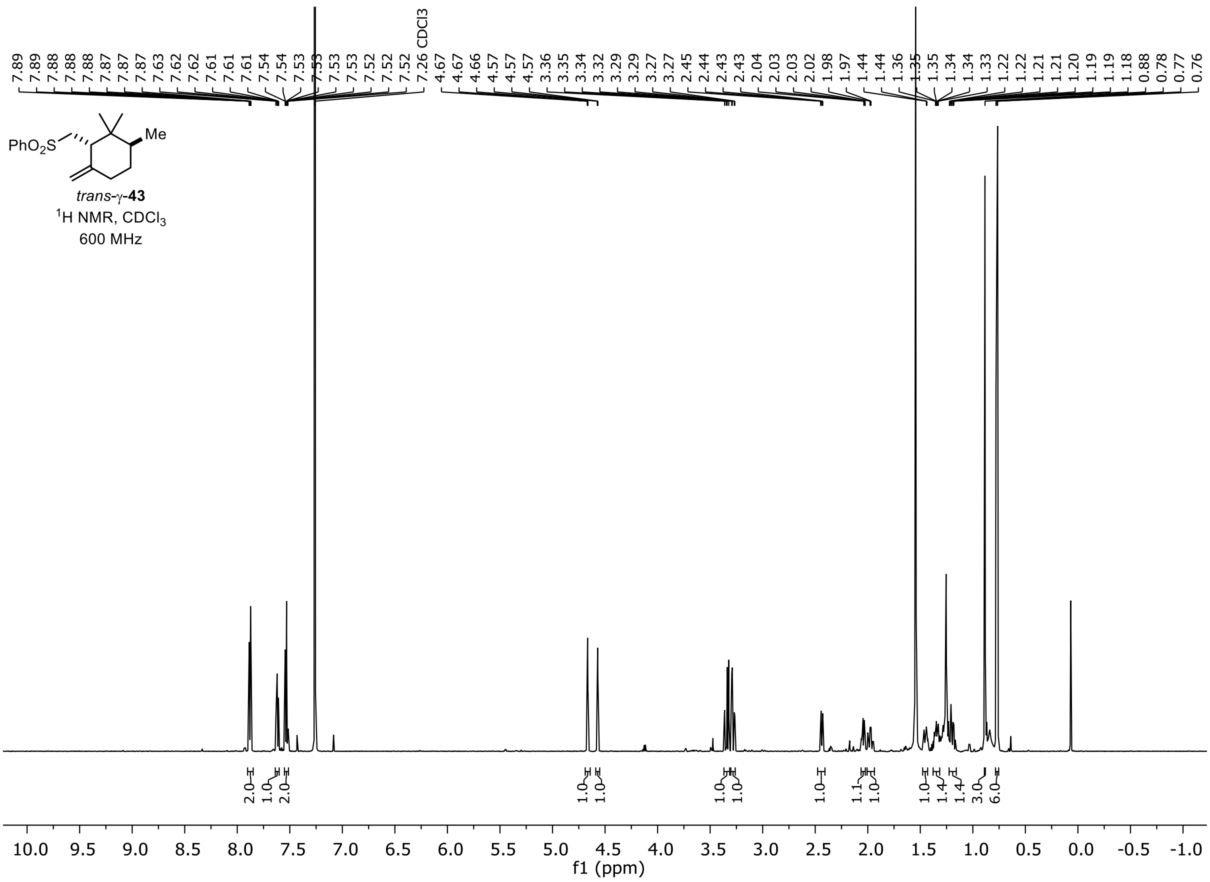

Figure 118. ^{1}H-NMR (600 MHz, $CDCl_3$) of cyclohexene *trans*-γ-**43**.

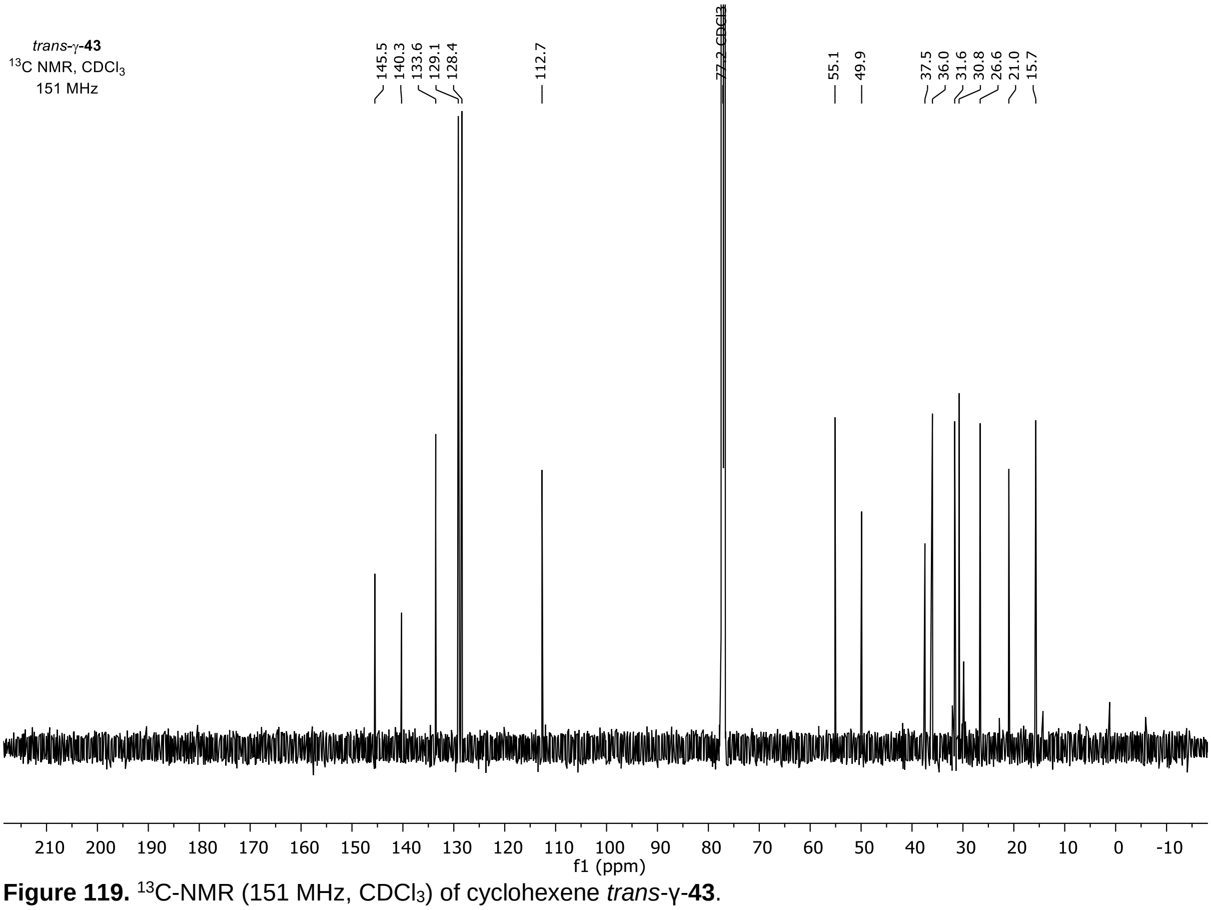

Figure 119. ^{13}C-NMR (151 MHz, $CDCl_3$) of cyclohexene *trans*-γ-**43**.

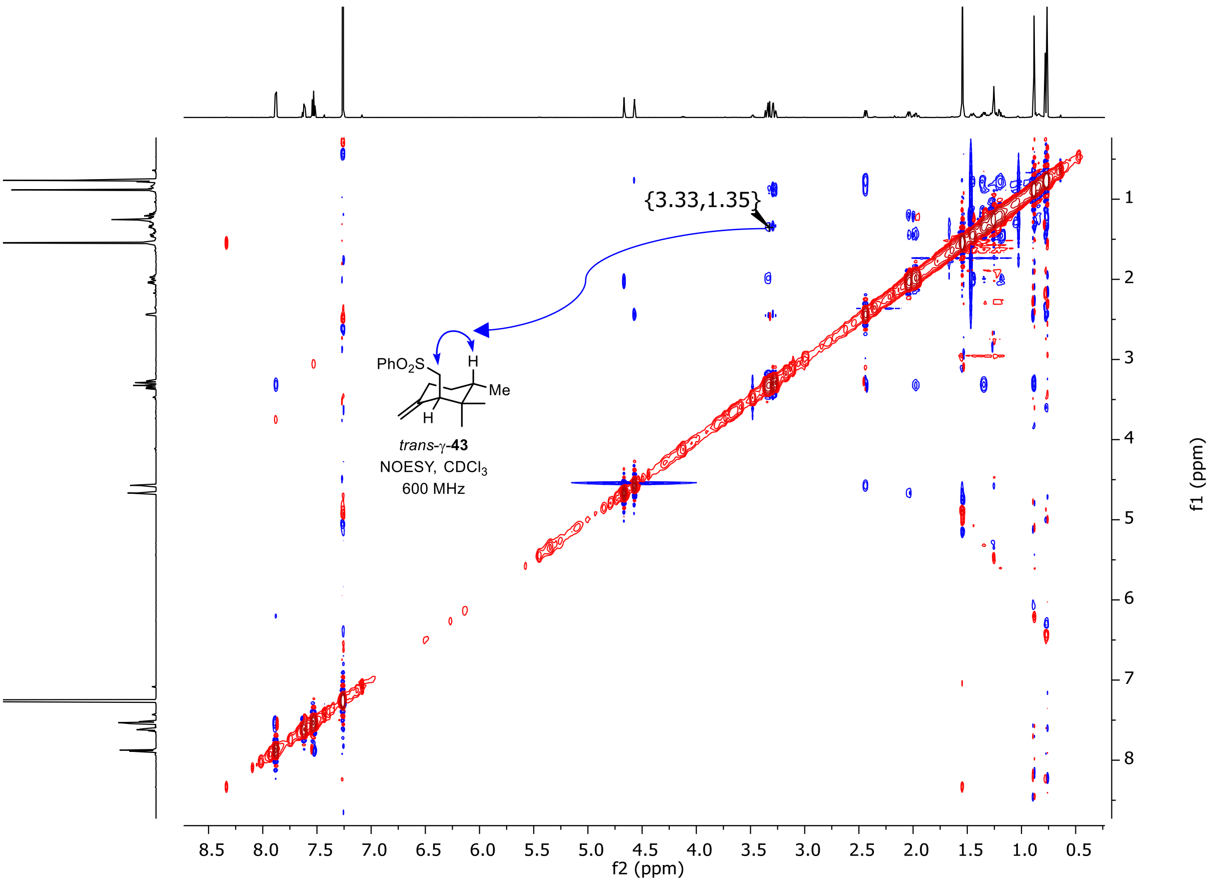

Figure 120. NOESY (600 MHz, $CDCl_3$) of cyclohexene *trans*-γ-**43**.

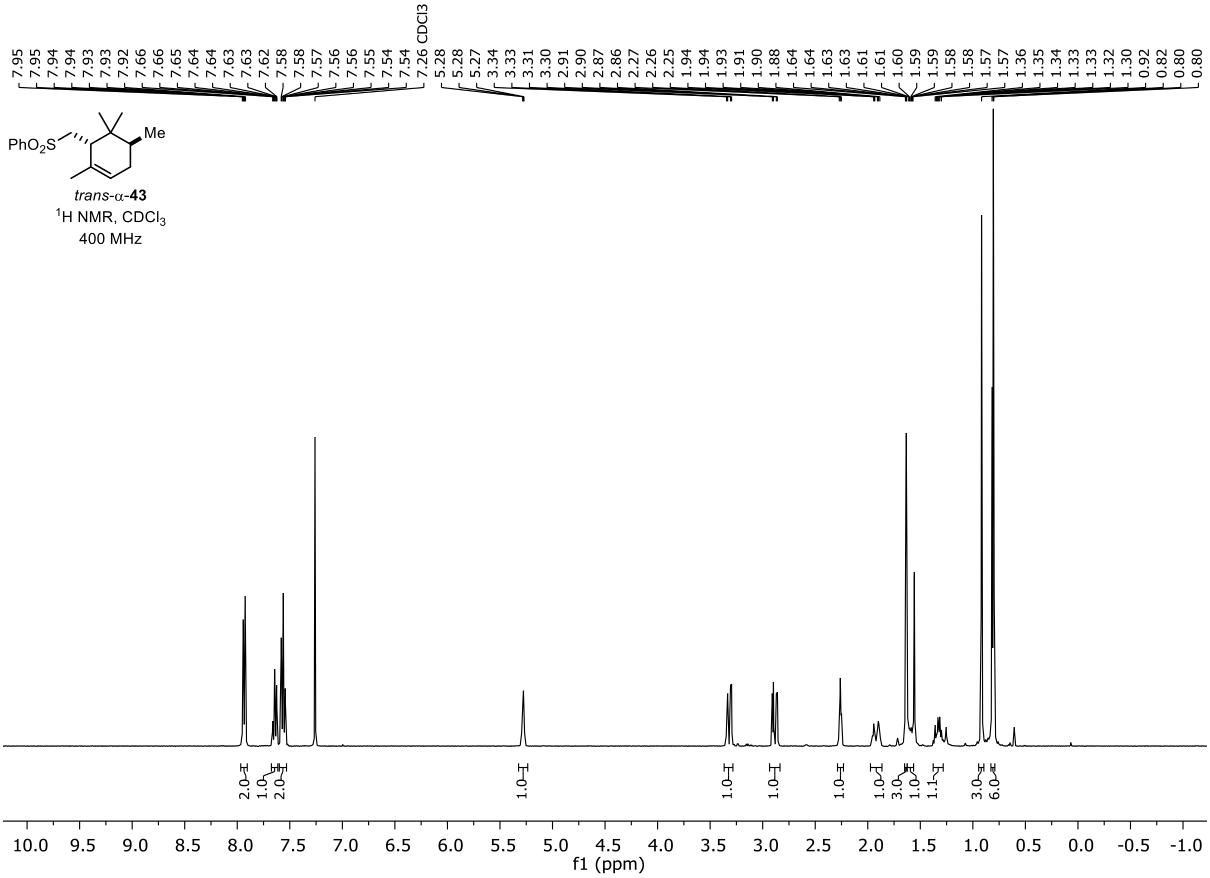

Figure 121. ^{1}H-NMR (400 MHz, $CDCl_3$) of cyclohexene *trans*-α-**43**.

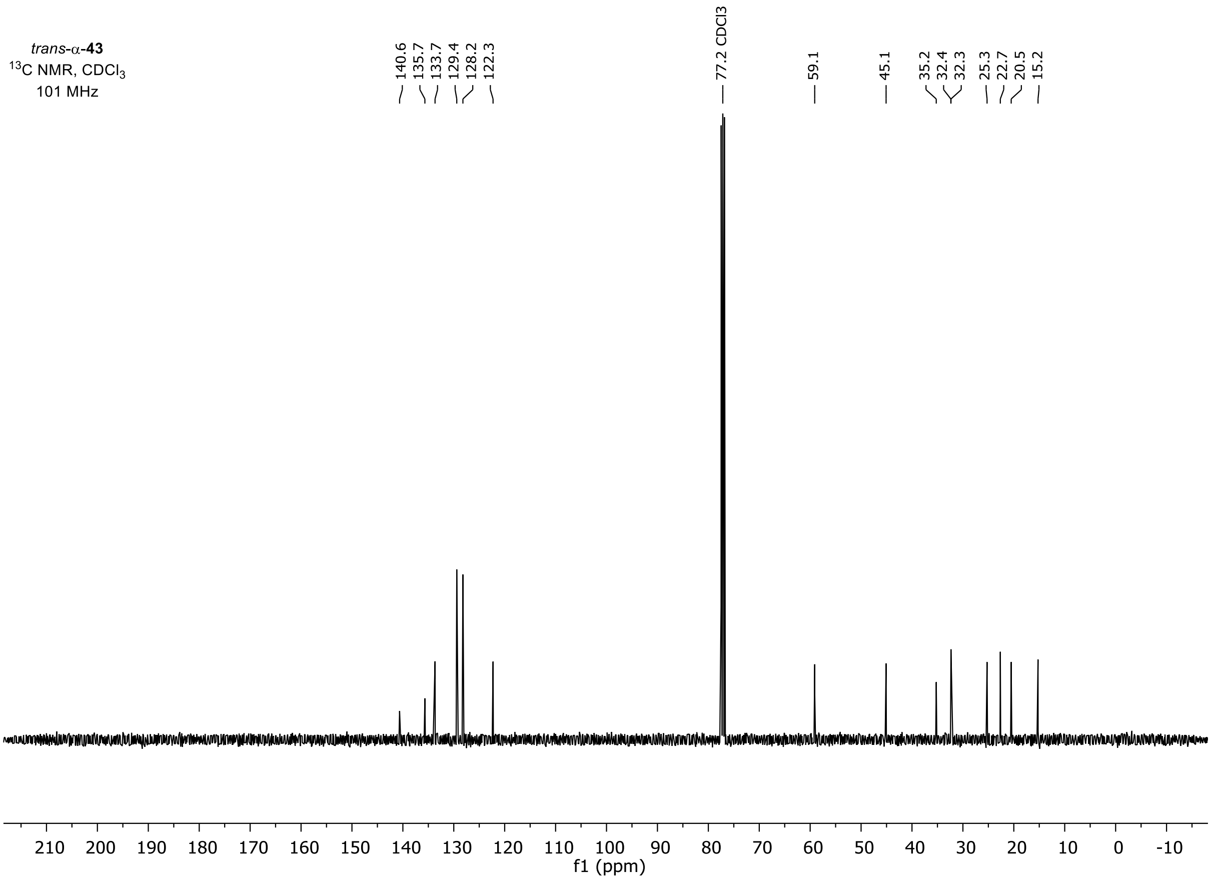

Figure 122. ^{13}C-NMR (101 MHz, $CDCl_3$) of cyclohexene *trans*-α-**43**.

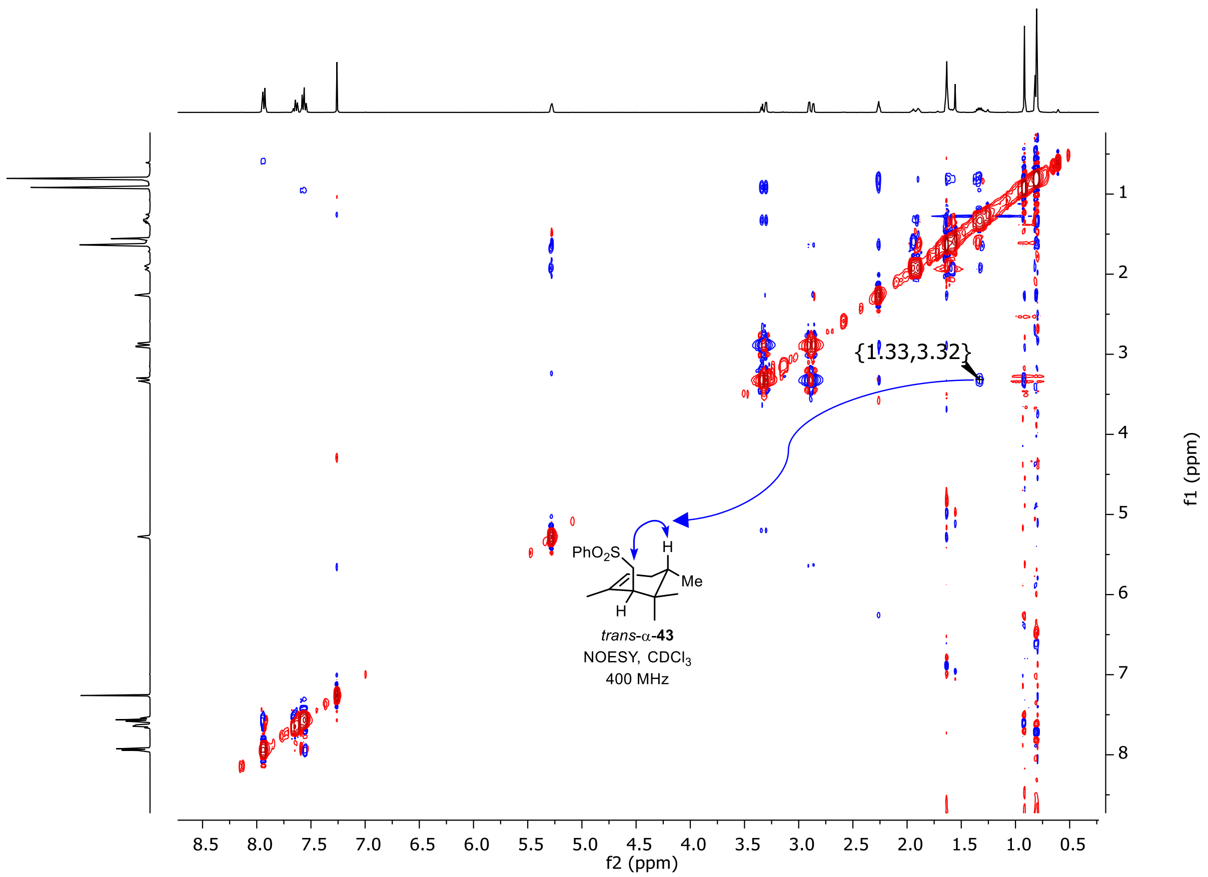

Figure 123. NOESY (400 MHz, $CDCl_3$) of cyclohexene *trans*-α-**43**.

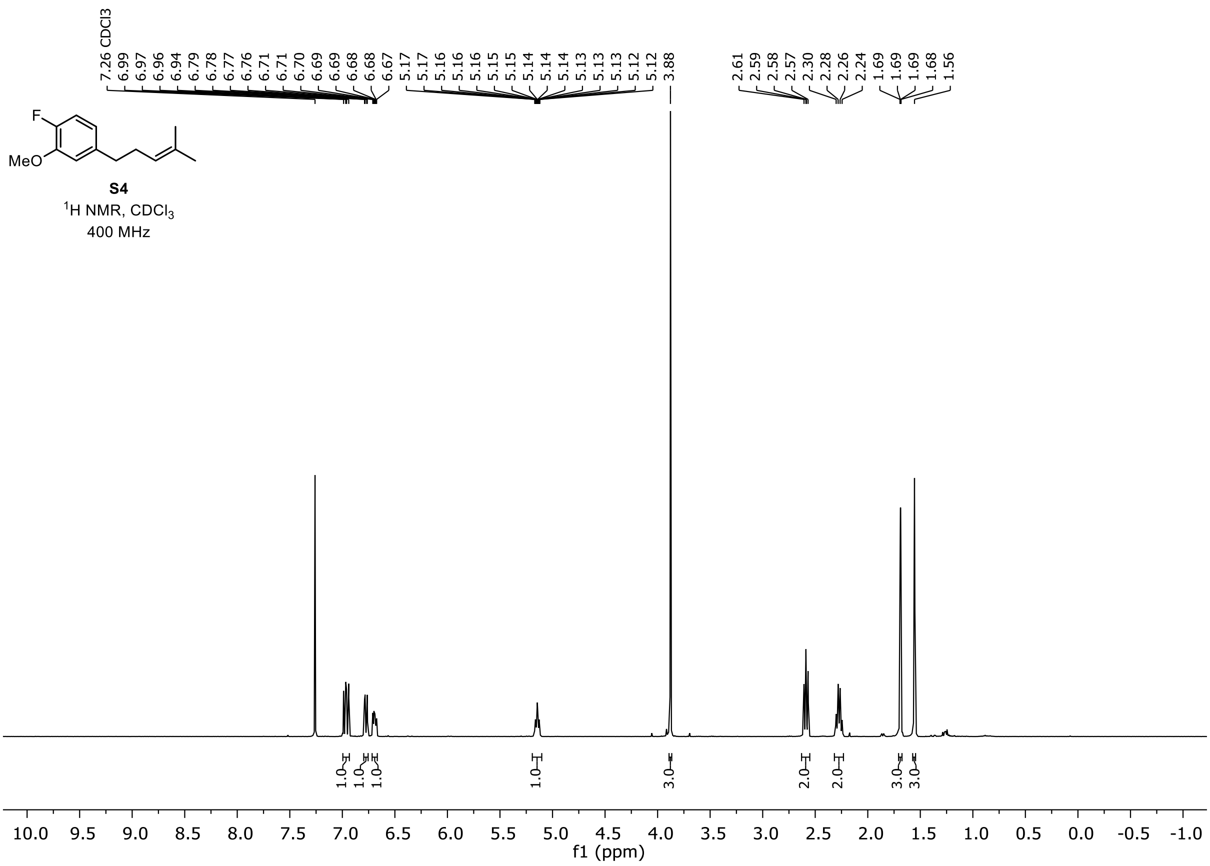

Figure 124. ^{1}H-NMR (400 MHz, $CDCl_3$) of alkene **S4**.

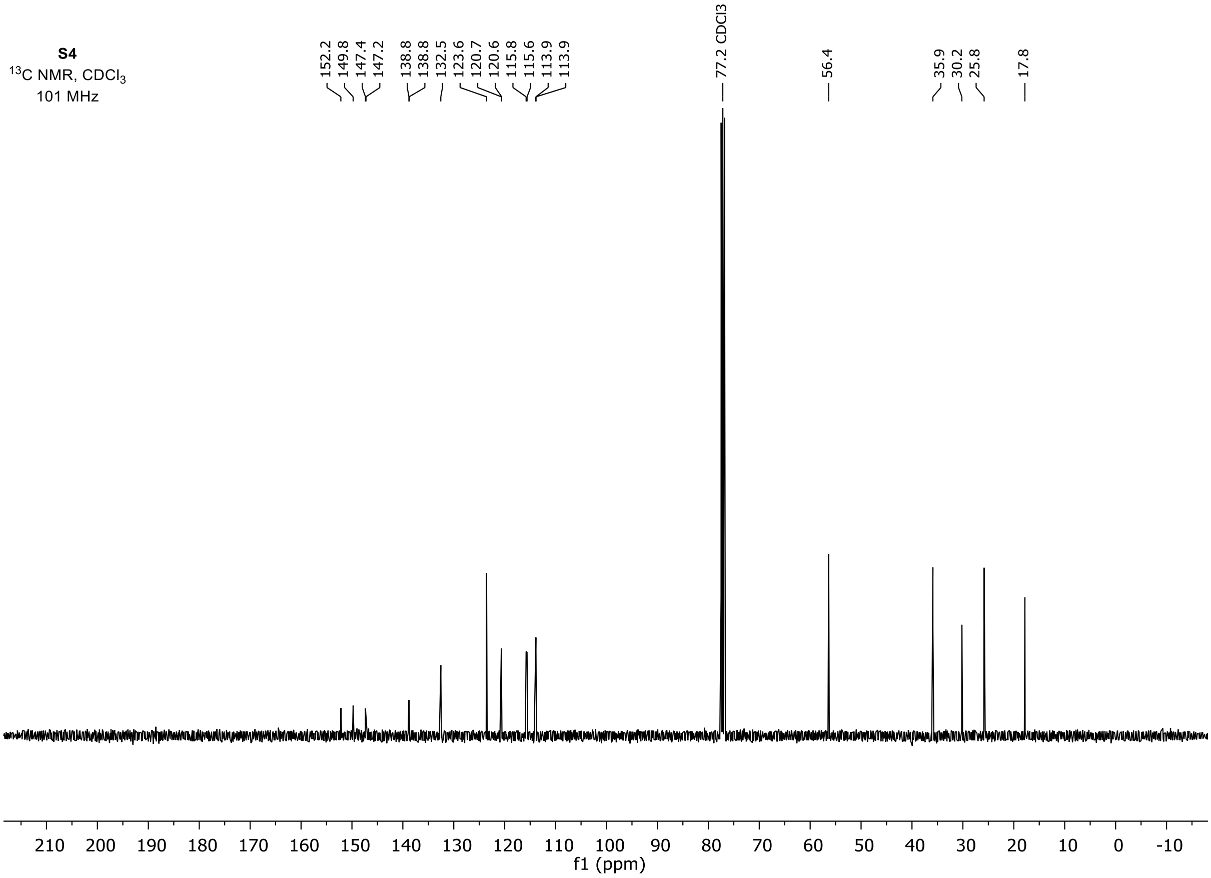

Figure 125. ^{13}C-NMR (101 MHz, $CDCl_3$) of alkene **S4**.

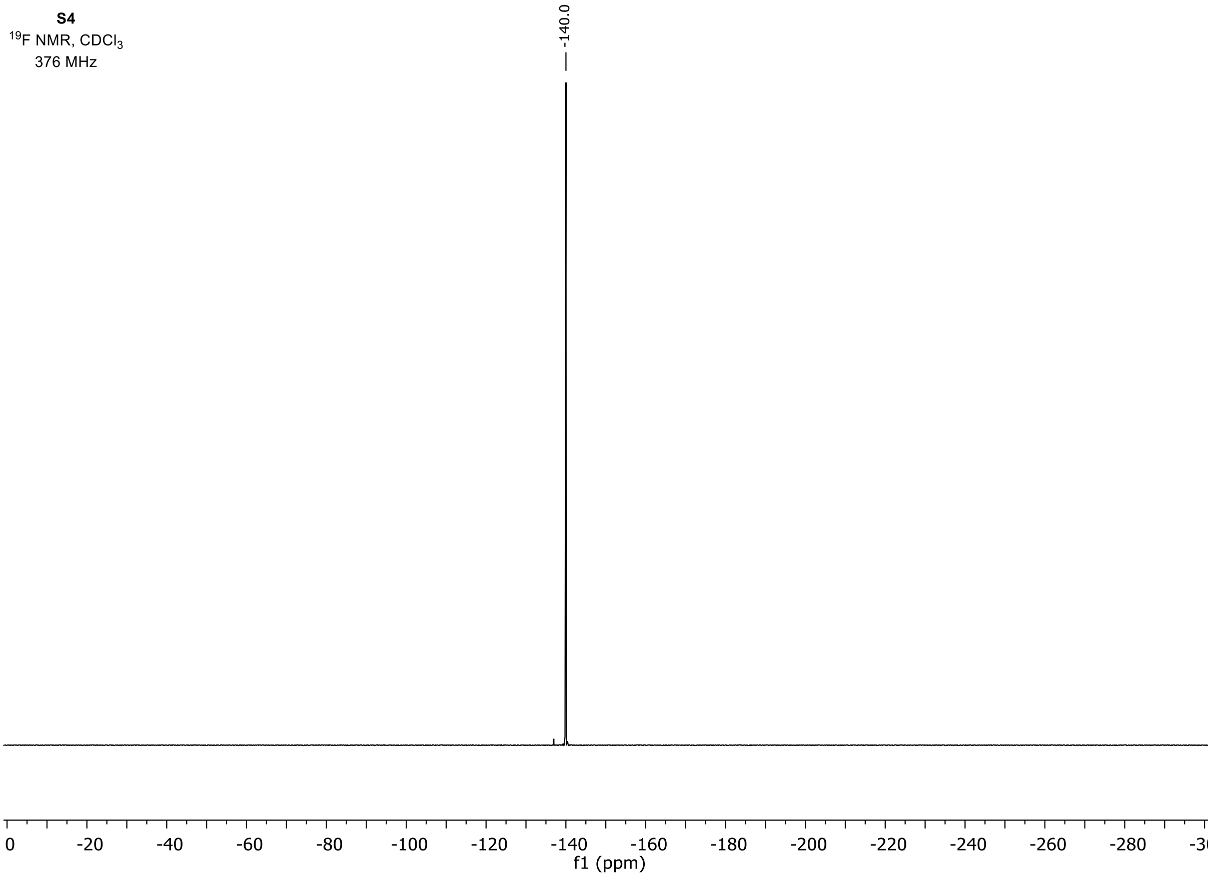

Figure 126. ^{19}F-NMR (376 MHz, $CDCl_3$) of alkene **S4**.

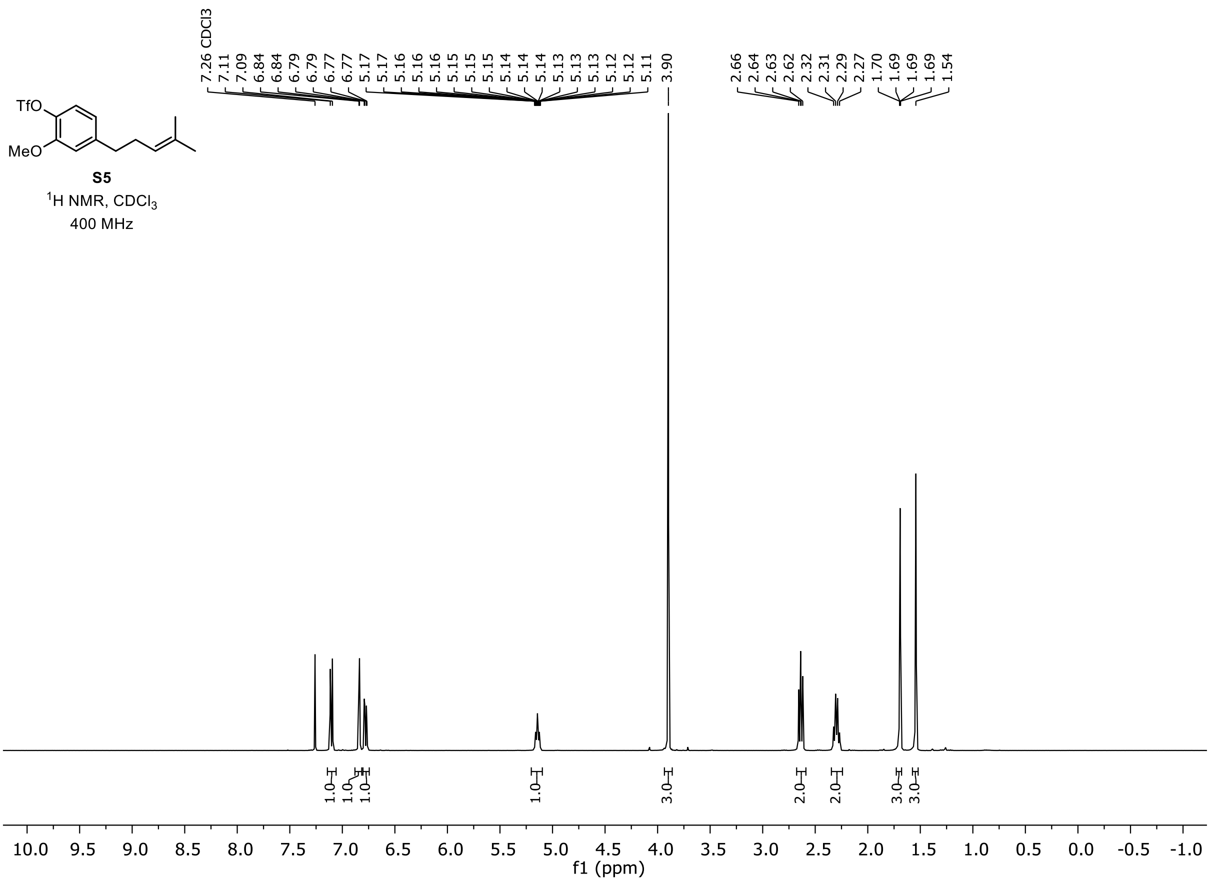

Figure 127. ^{1}H-NMR (400 MHz, $CDCl_3$) of alkene **S5**.

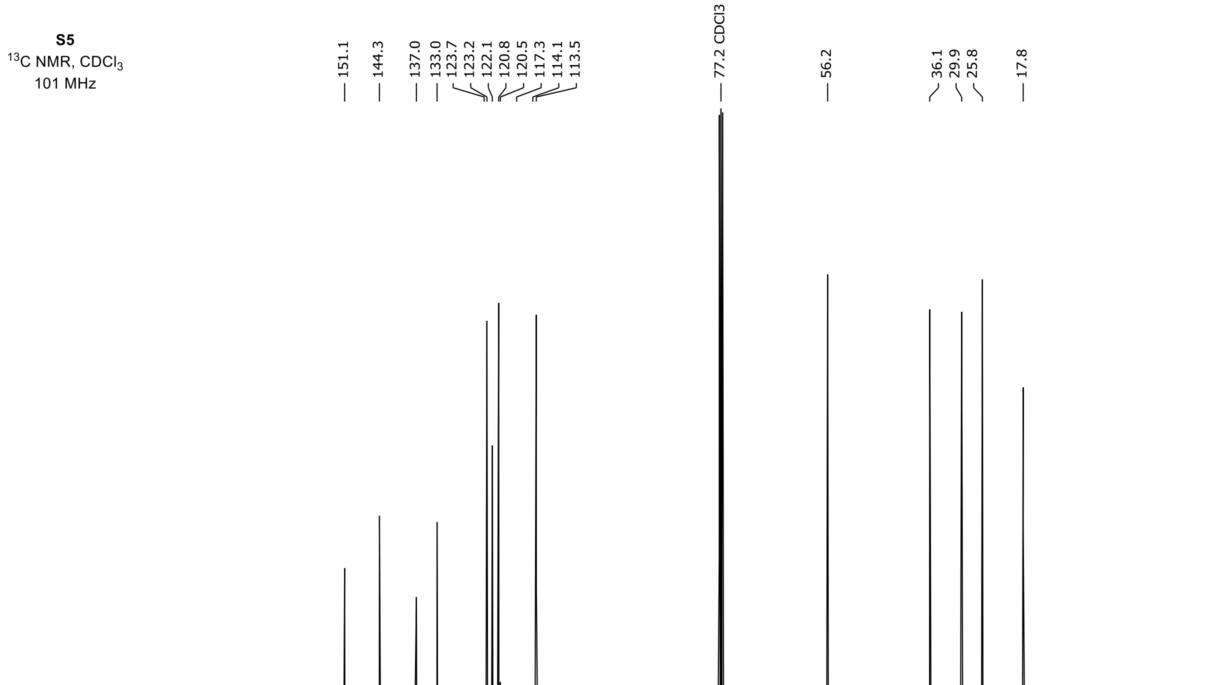

Figure 128. ^{13}C-NMR (101 MHz, $CDCl_3$) of alkene **S5**.

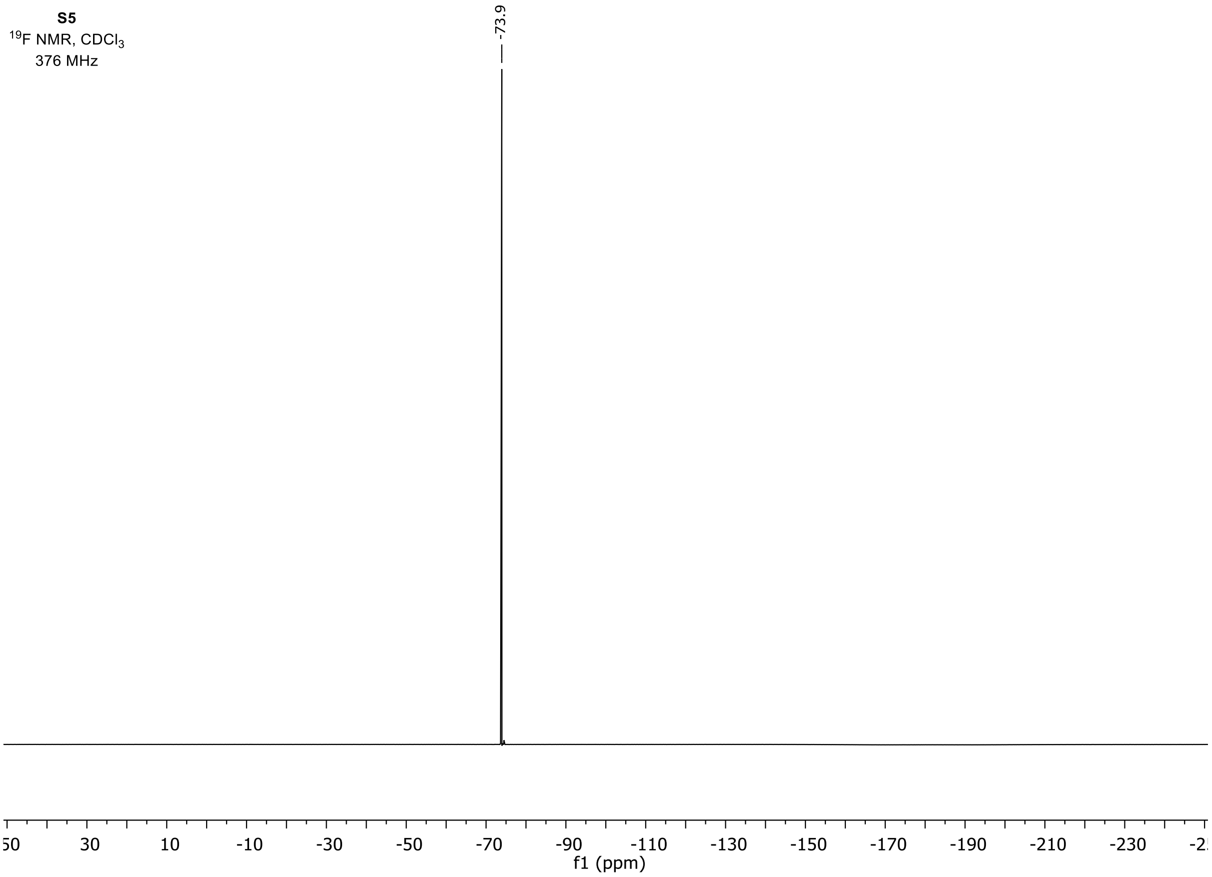

Figure 129. ^{19}F-NMR (376 MHz, $CDCl_3$) of alkene **S5**.

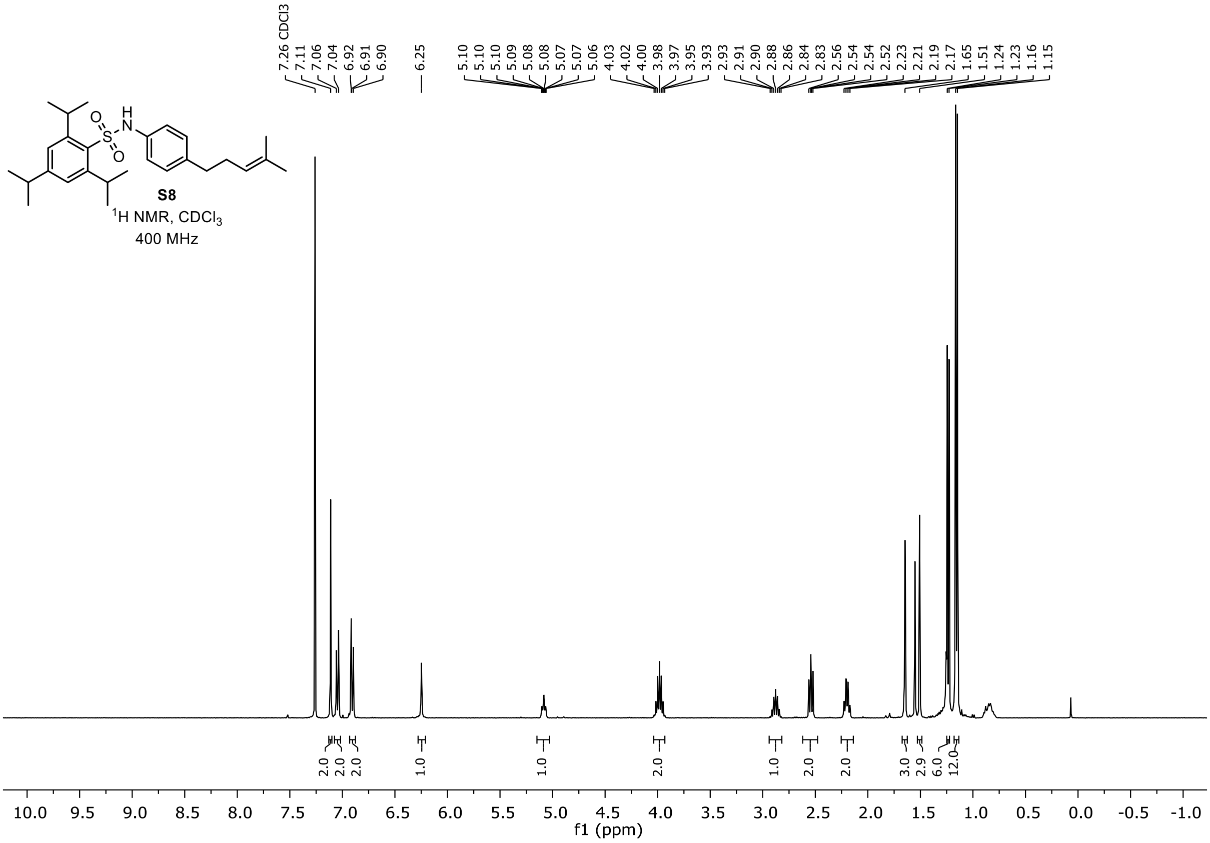

Figure 130. ^{1}H-NMR (400 MHz, $CDCl_3$) of alkene **S8**.

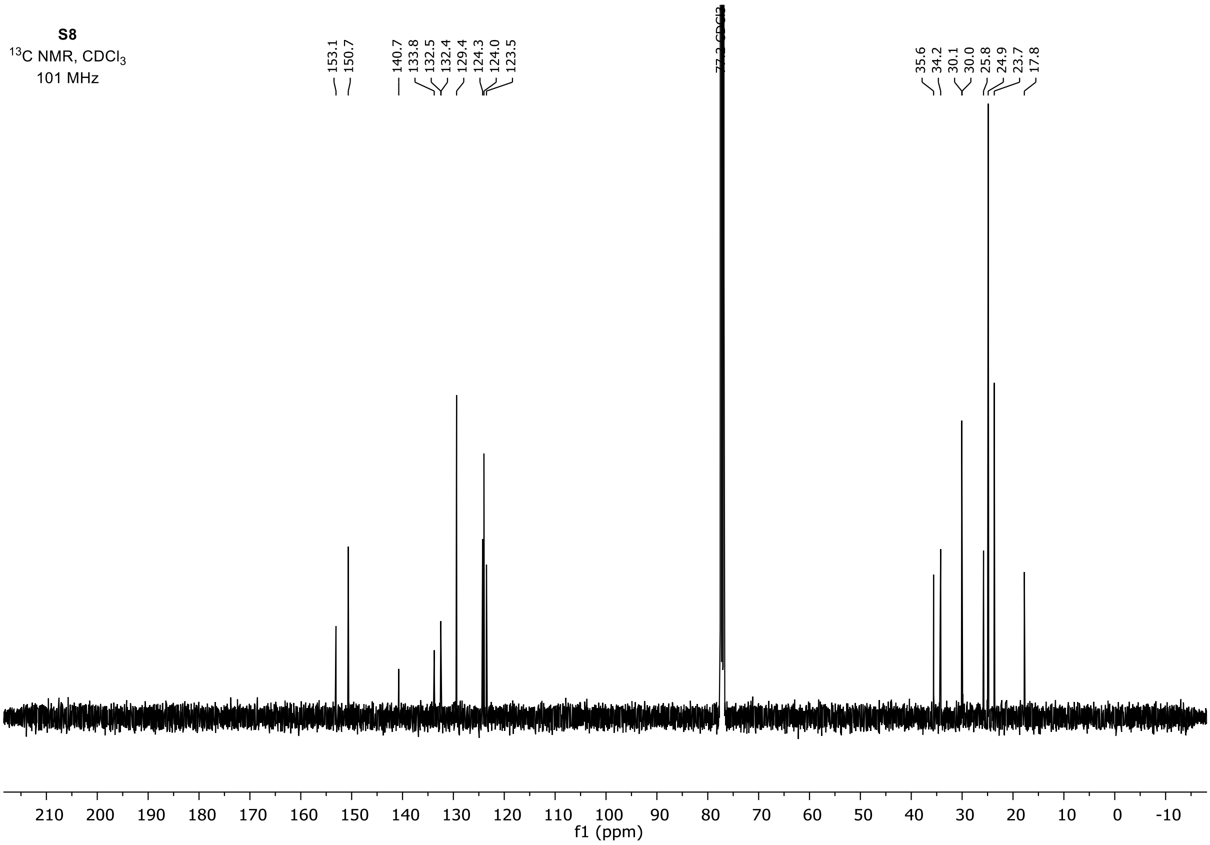

Figure 131. ^{13}C-NMR (101 MHz, $CDCl_3$) of alkene **S8**.

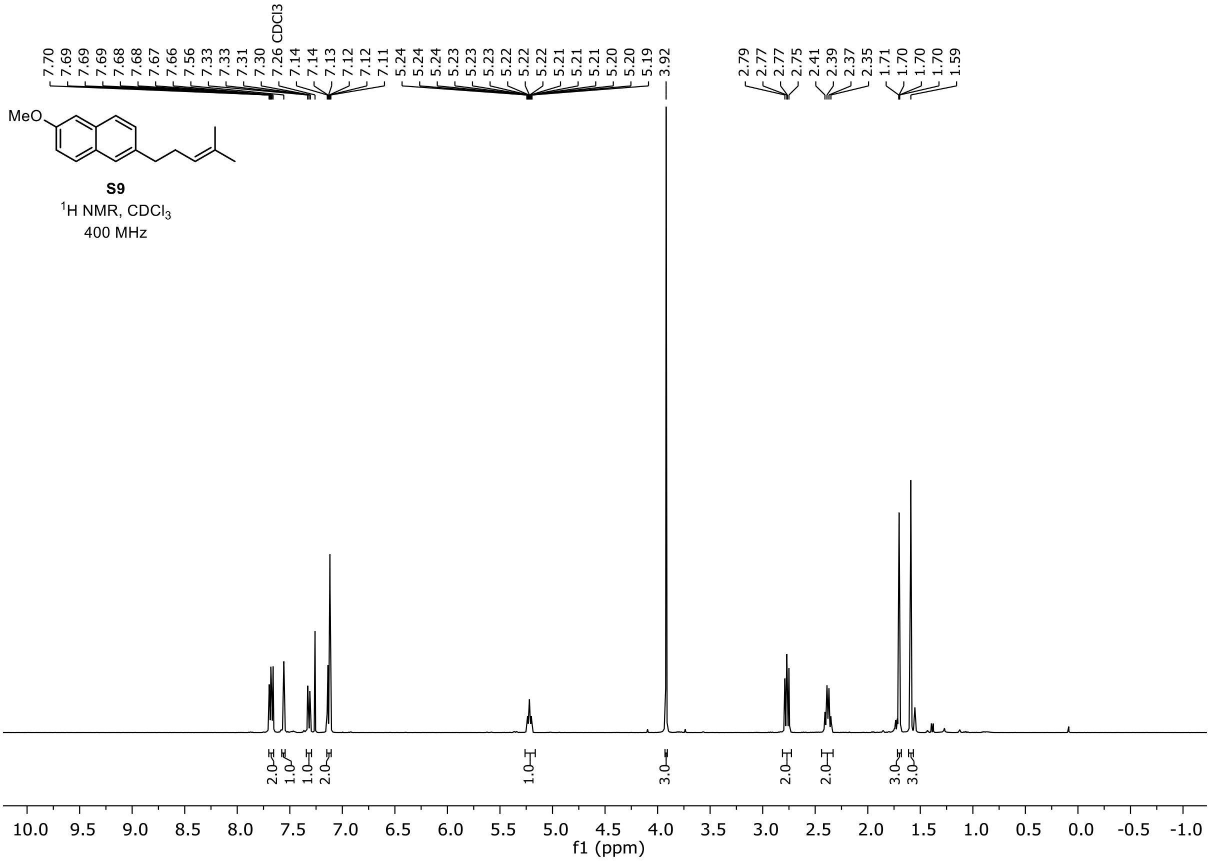

Figure 132. ^{1}H-NMR (400 MHz, $CDCl_3$) of alkene **S9**.

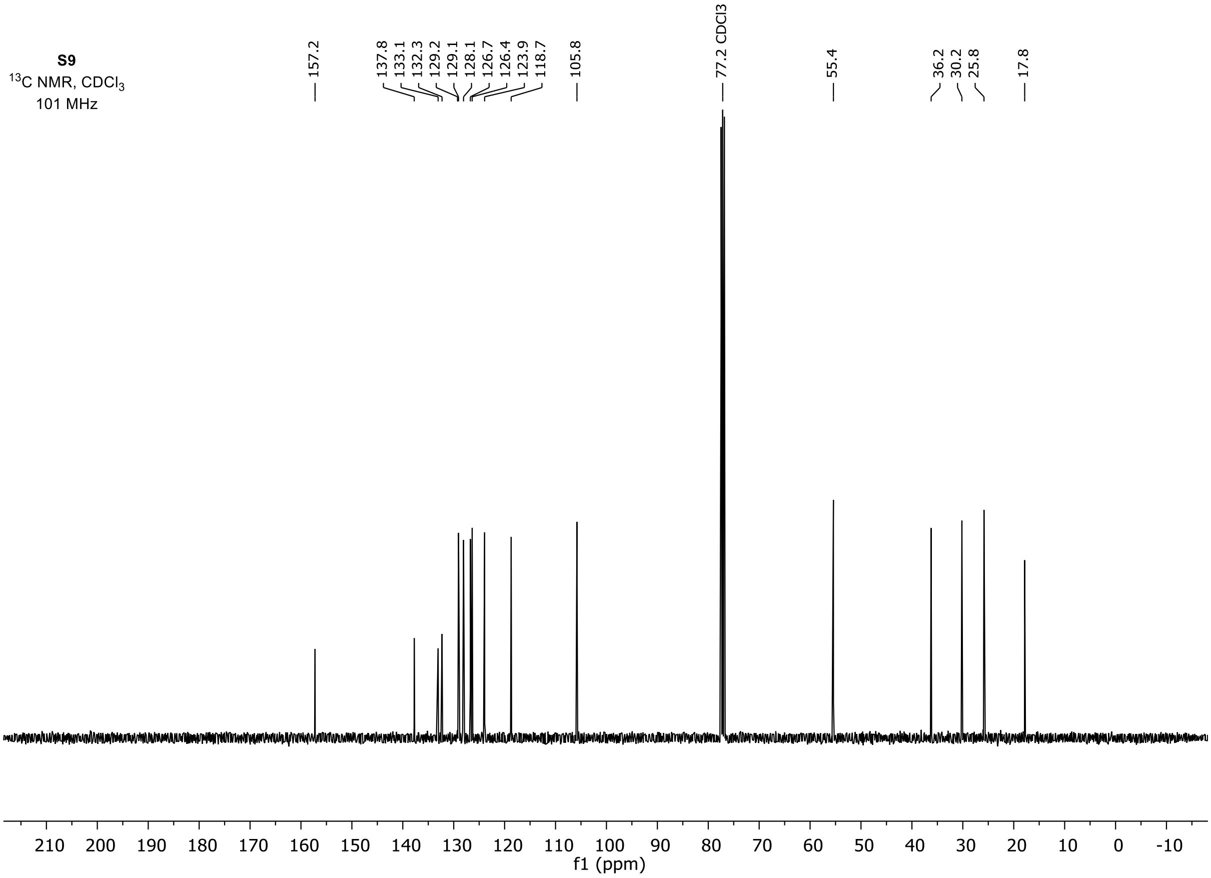

Figure 133. ^{13}C-NMR (101 MHz, $CDCl_3$) of alkene **S9**.

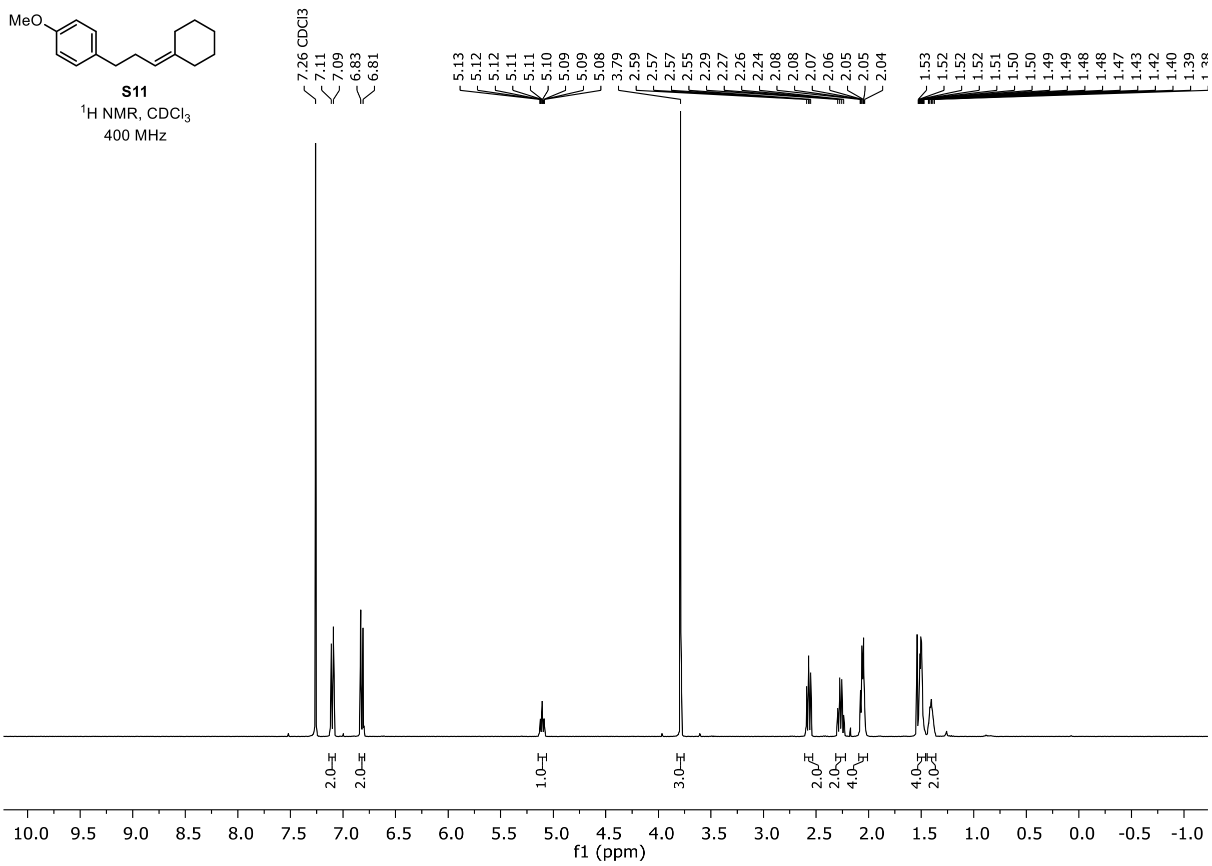

Figure 134. ^{1}H-NMR (400 MHz, $CDCl_3$) of alkene **S11**.

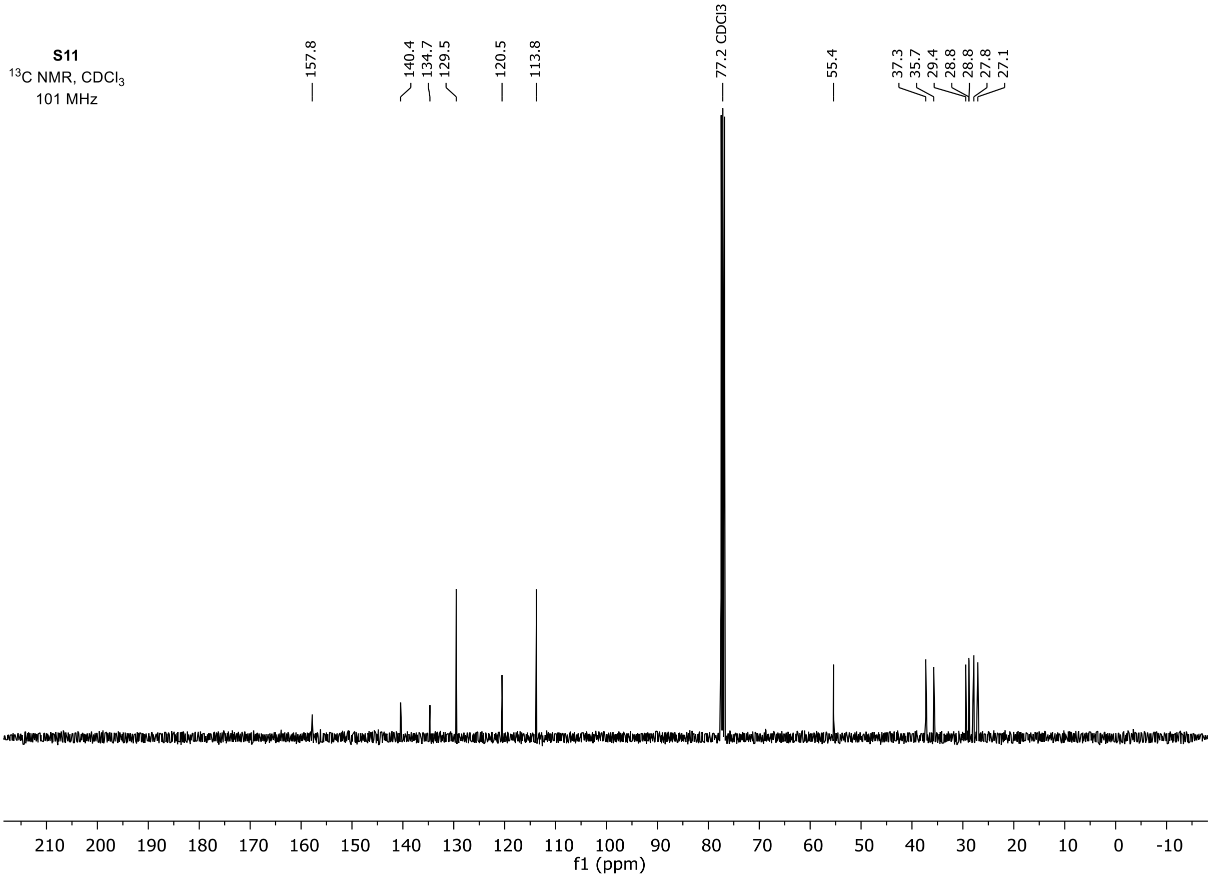

Figure 135. ^{13}C-NMR (101 MHz, $CDCl_3$) of alkene **S11**.

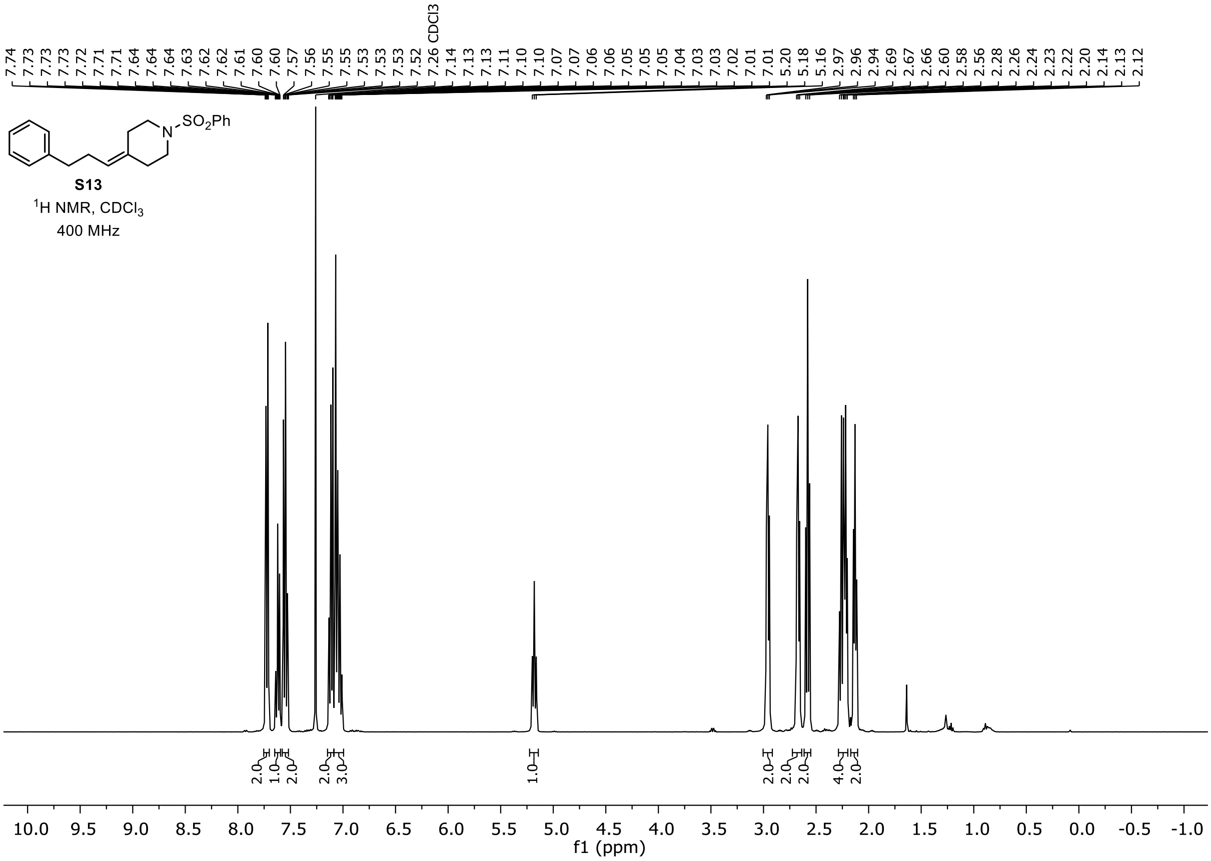

Figure 136. ^{1}H-NMR (400 MHz, $CDCl_3$) of piperidine **S13**.

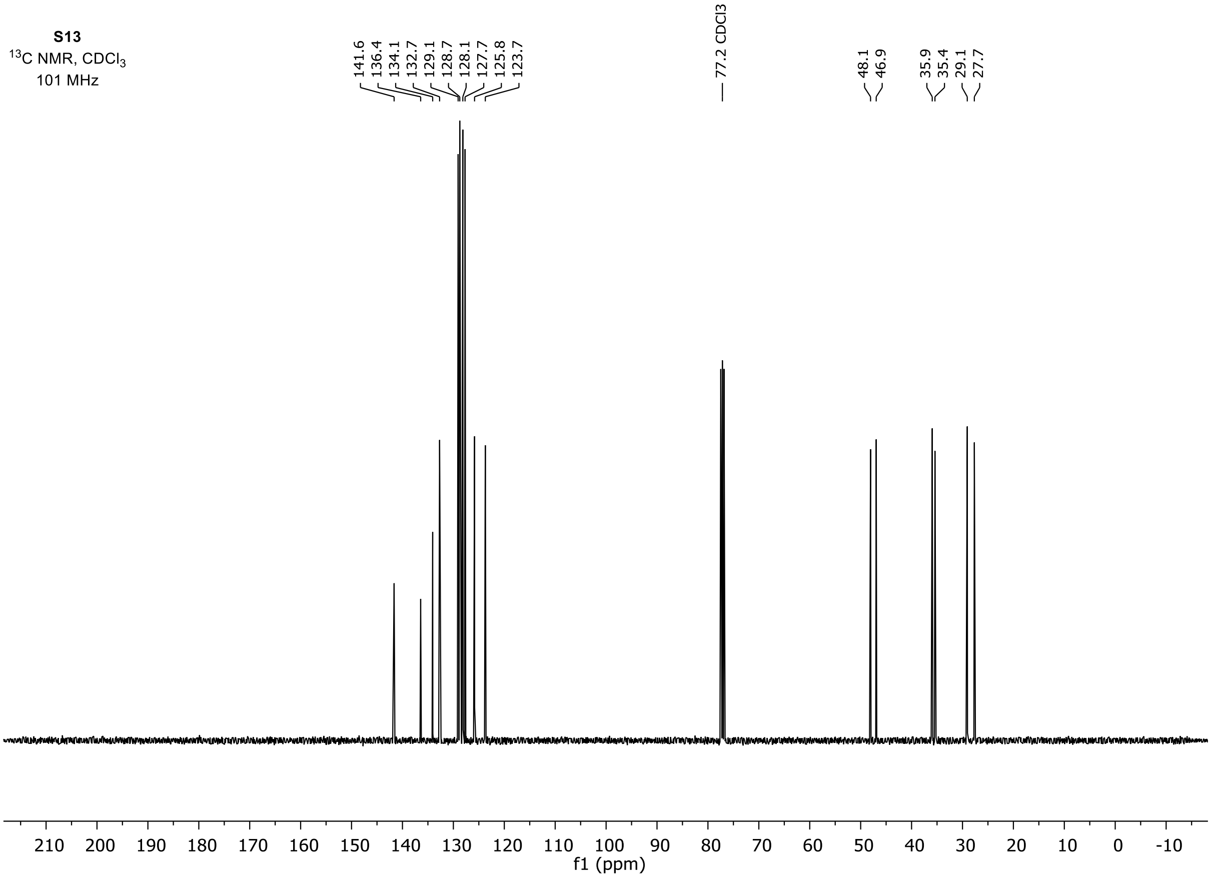

Figure 137. ^{13}C-NMR (101 MHz, $CDCl_3$) of piperidine **S13**.

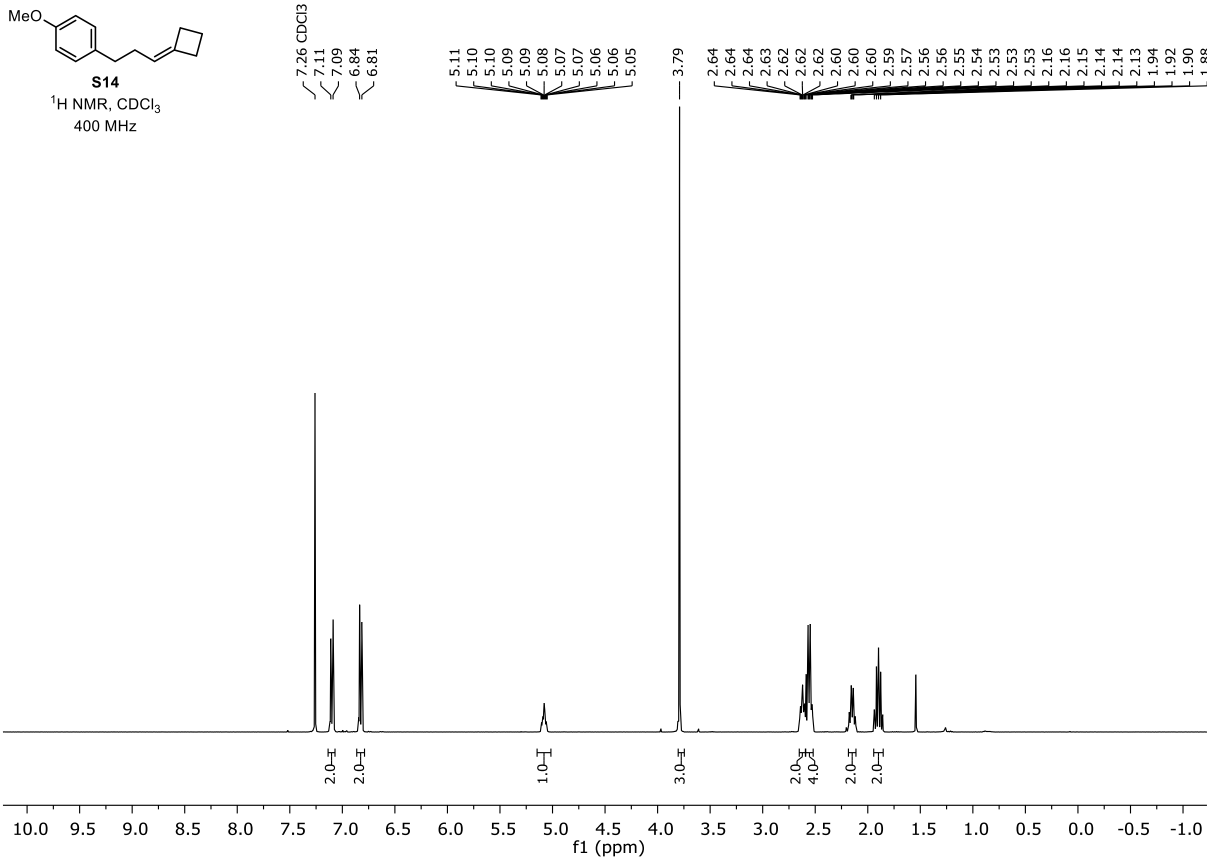

Figure 138. ^{1}H-NMR (400 MHz, $CDCl_3$) of alkene **S14**.

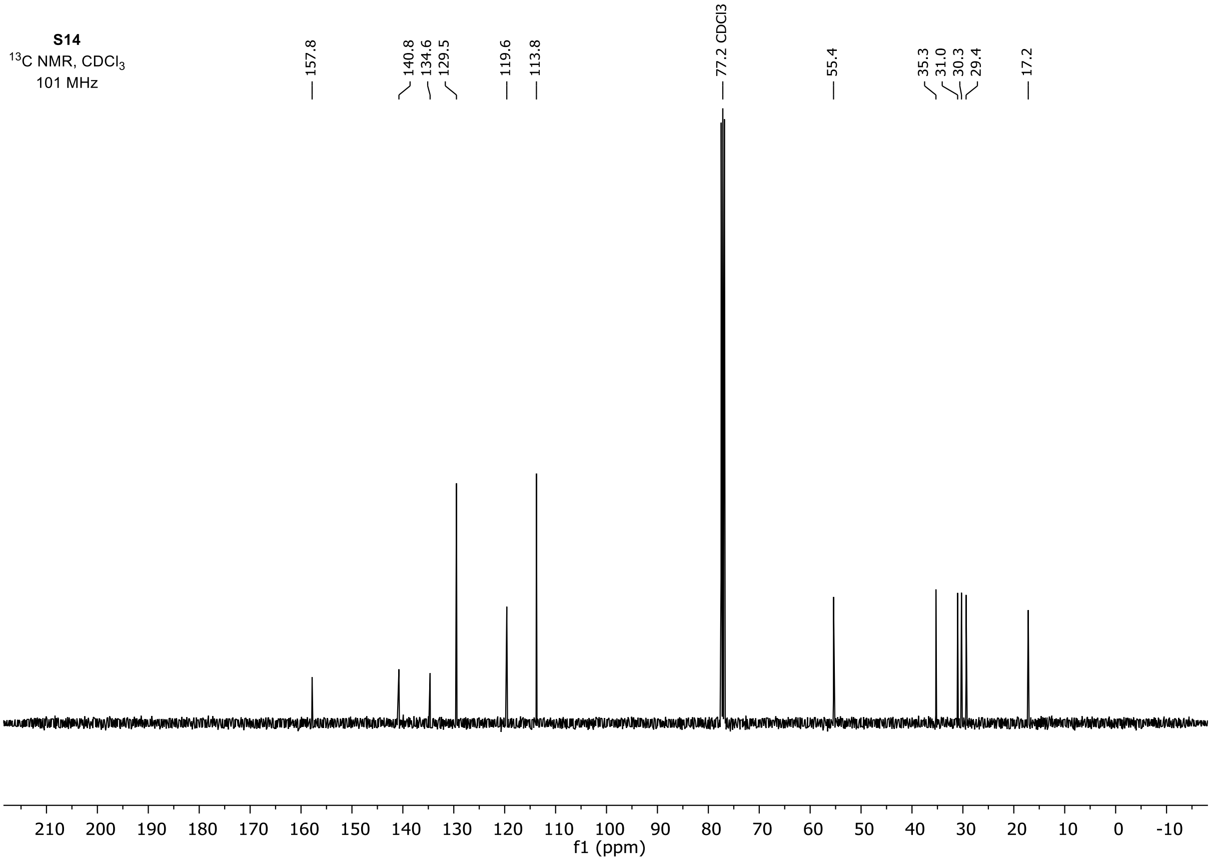

Figure 139. ^{13}C-NMR (101 MHz, $CDCl_3$) of alkene **S14**.

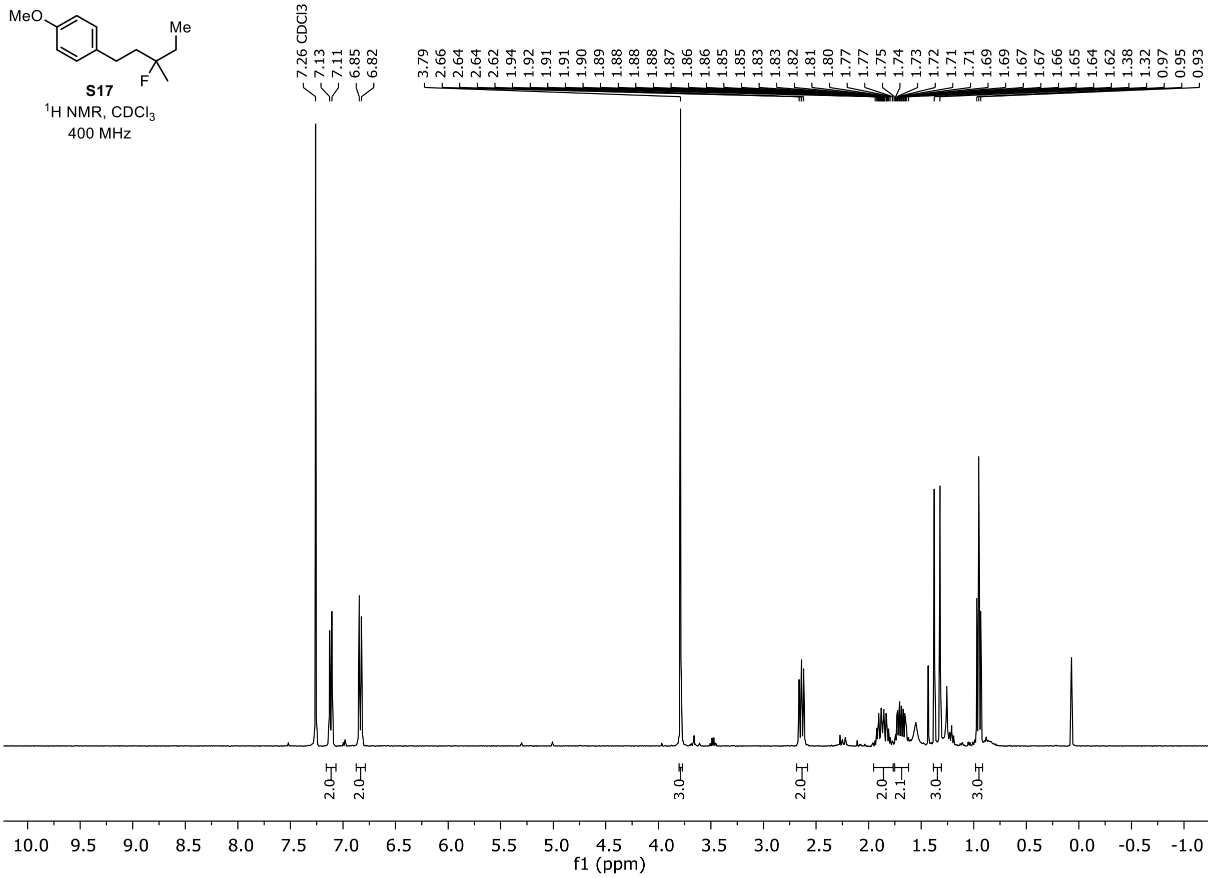

Figure 140. ^{1}H-NMR (400 MHz, $CDCl_3$) of fluoride **S17**.

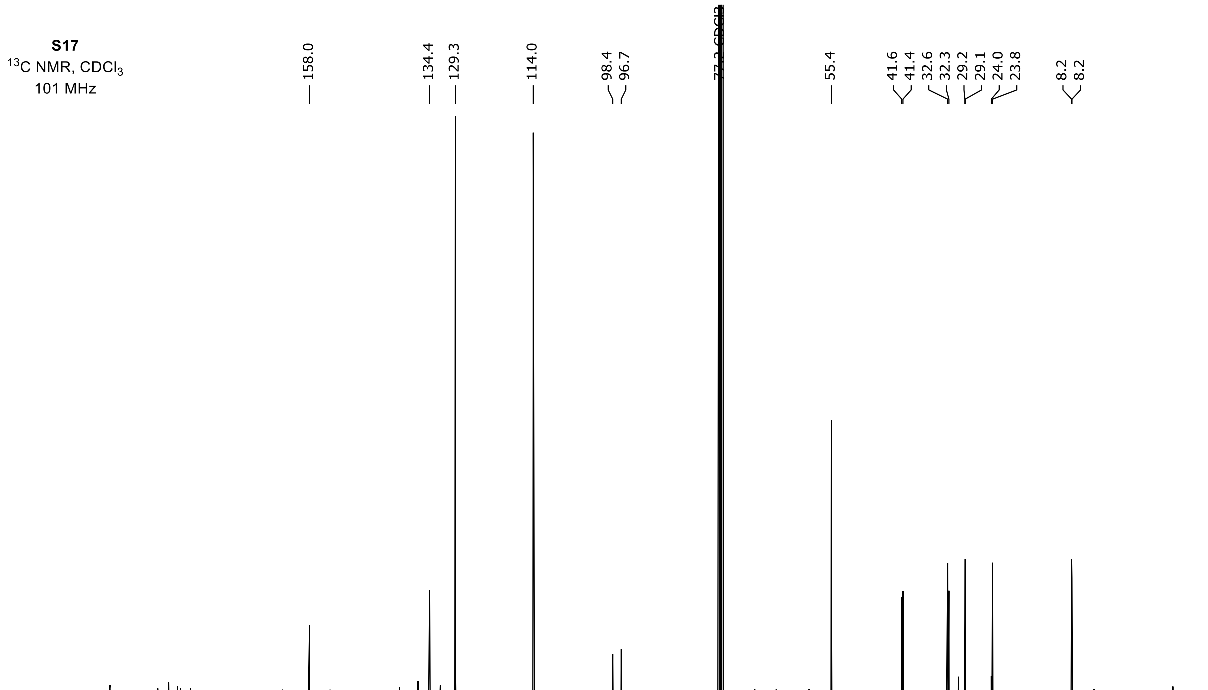

Figure 141. ^{13}C-NMR (101 MHz, $CDCl_3$) of fluoride **S17**.

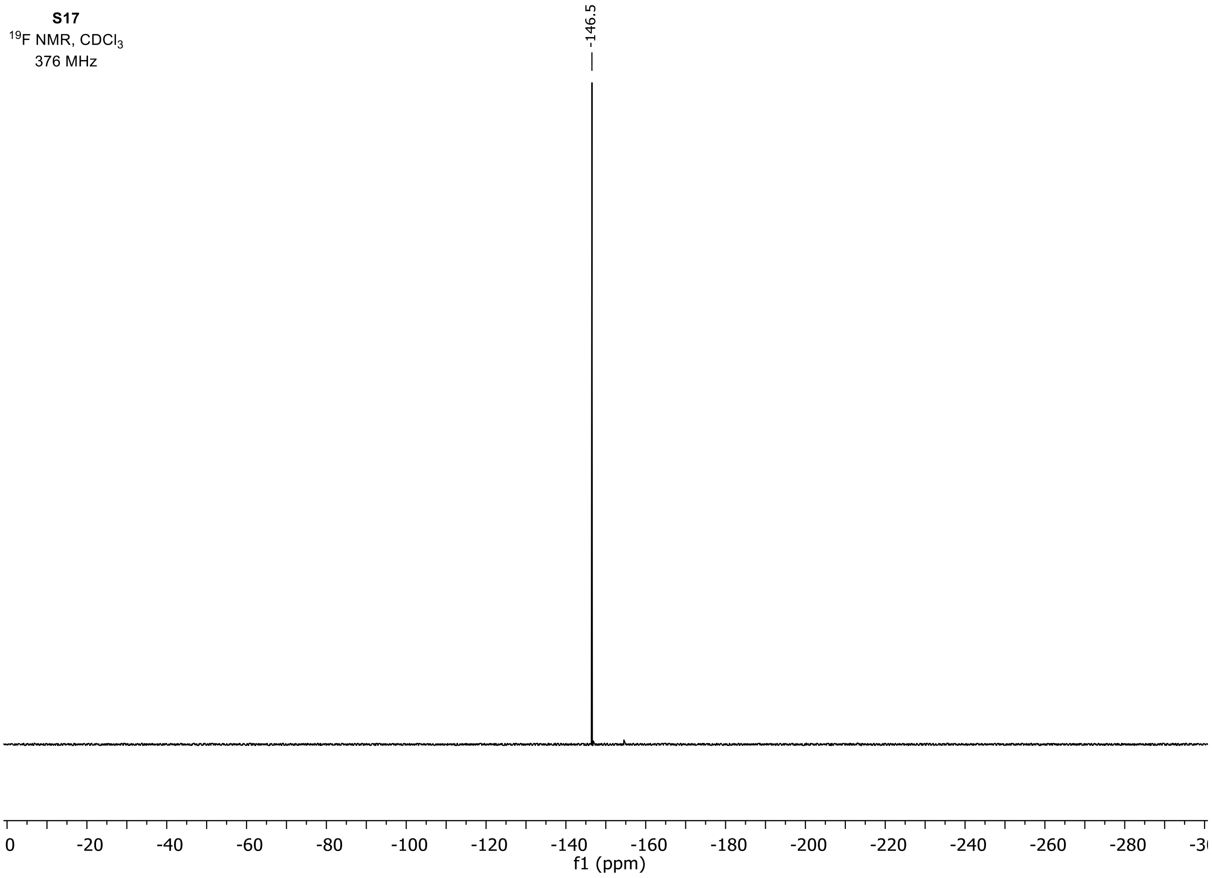

Figure 142. ^{19}F-NMR (376 MHz, $CDCl_3$) of fluoride **S17**.

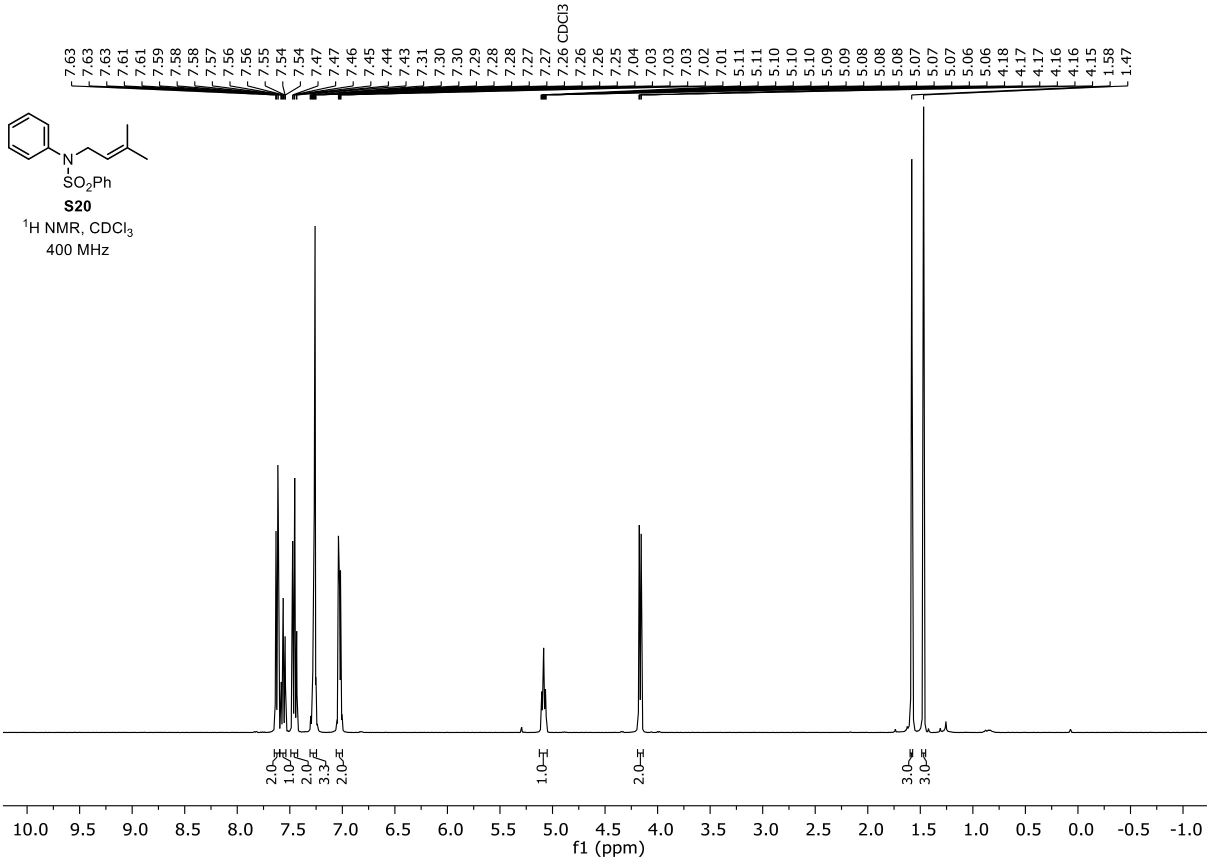

Figure 143. ^{1}H-NMR (400 MHz, $CDCl_3$) of alkene **S20**.

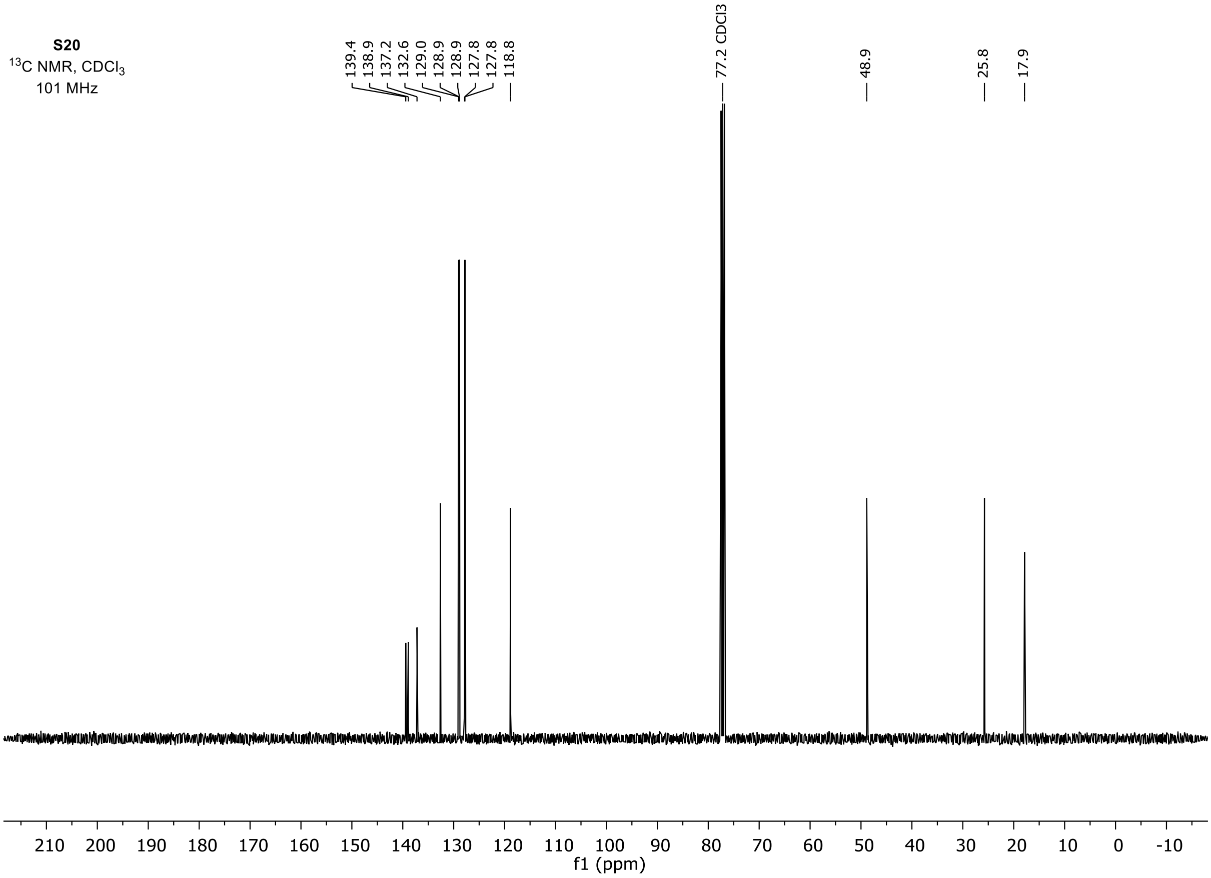

Figure 144. ^{13}C-NMR (101 MHz, $CDCl_3$) of alkene **S20**.

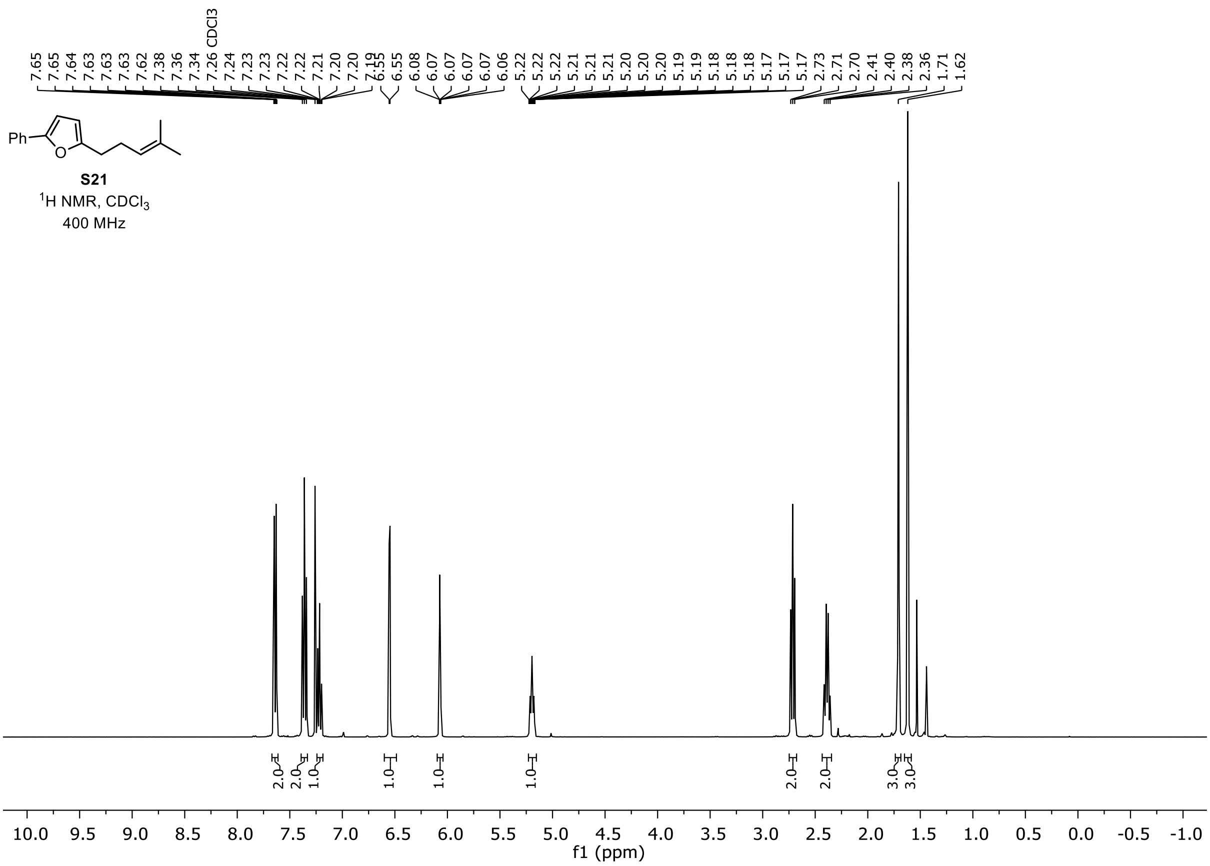

Figure 145. ^{1}H-NMR (400 MHz, $CDCl_3$) of alkene **S21**.

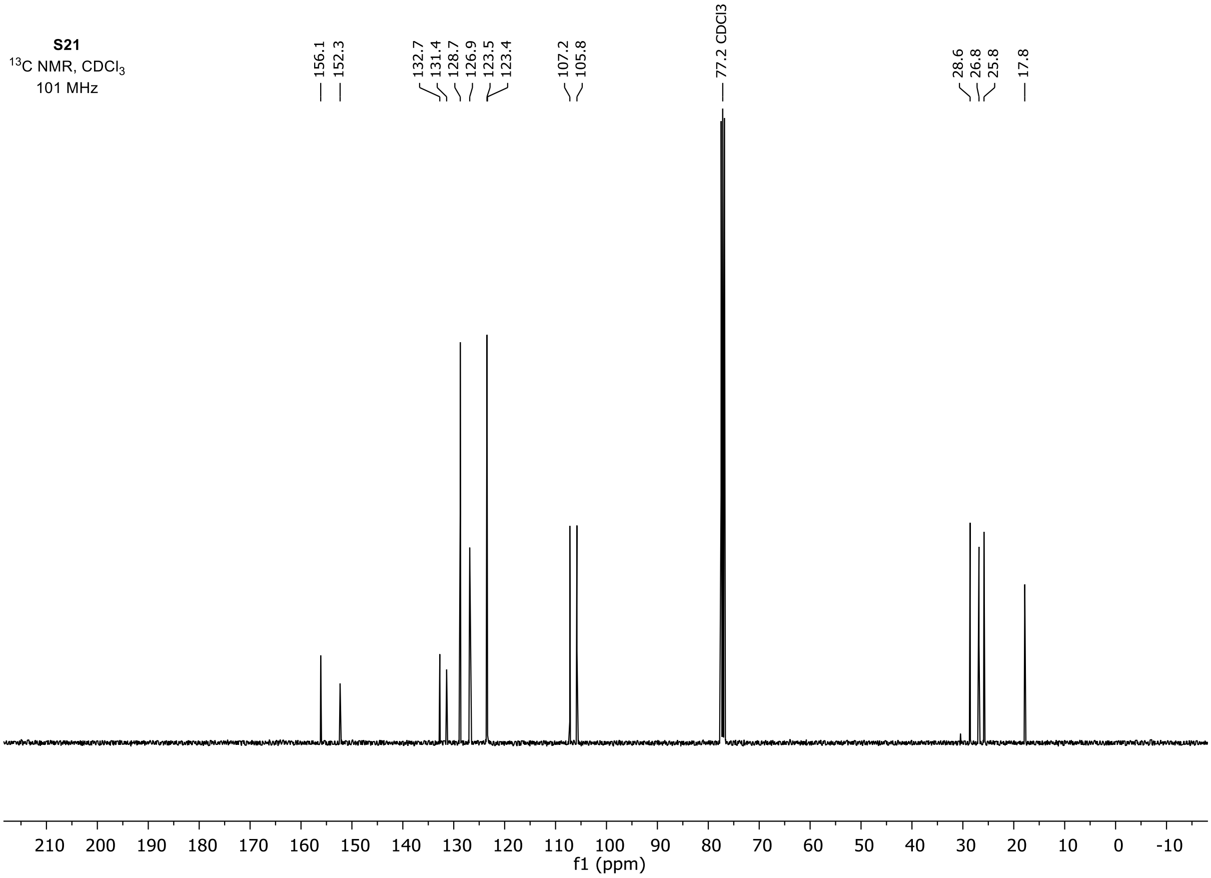

Figure 146. ^{13}C-NMR (101 MHz, $CDCl_3$) of alkene **S21**.

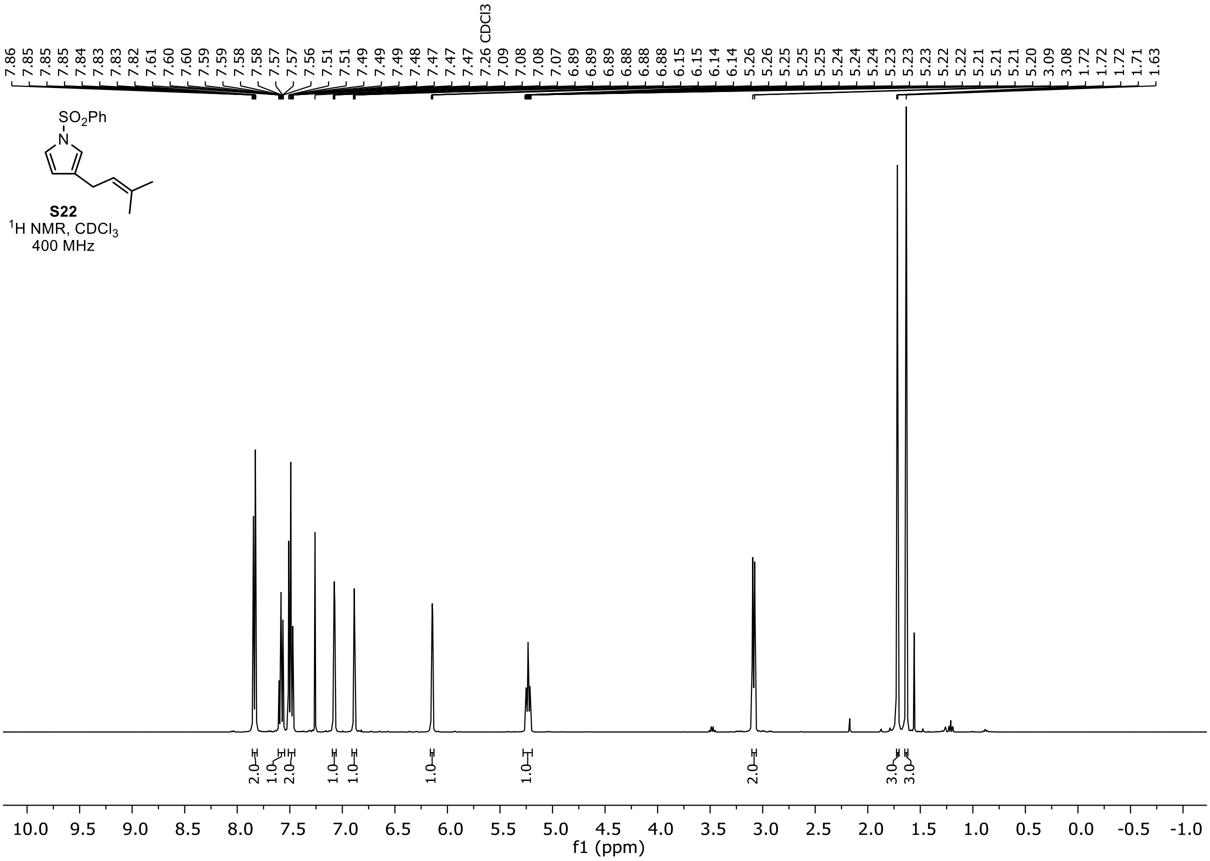

Figure 147. ^{1}H-NMR (400 MHz, $CDCl_3$) of alkene **S22**.

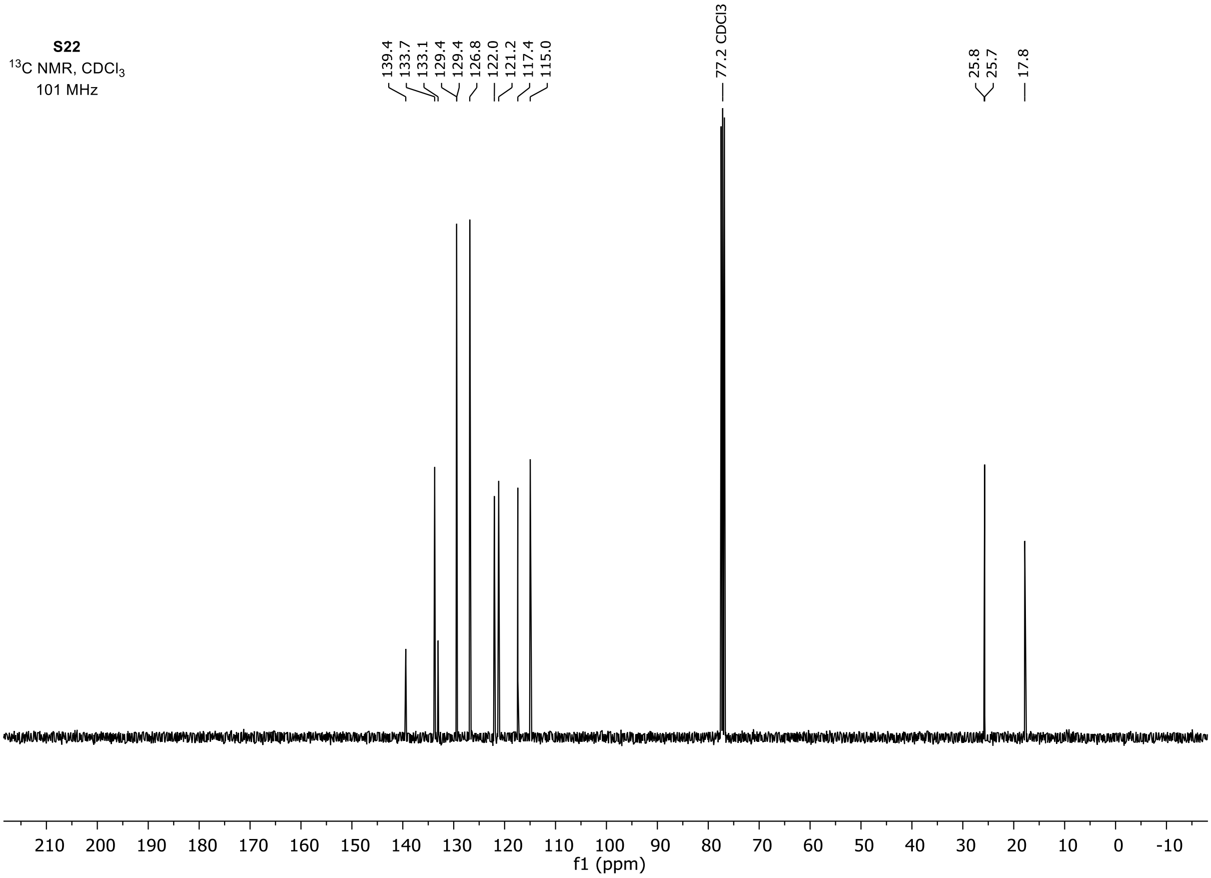

Figure 148. ^{13}C-NMR (101 MHz, $CDCl_3$) of alkene **S22**.

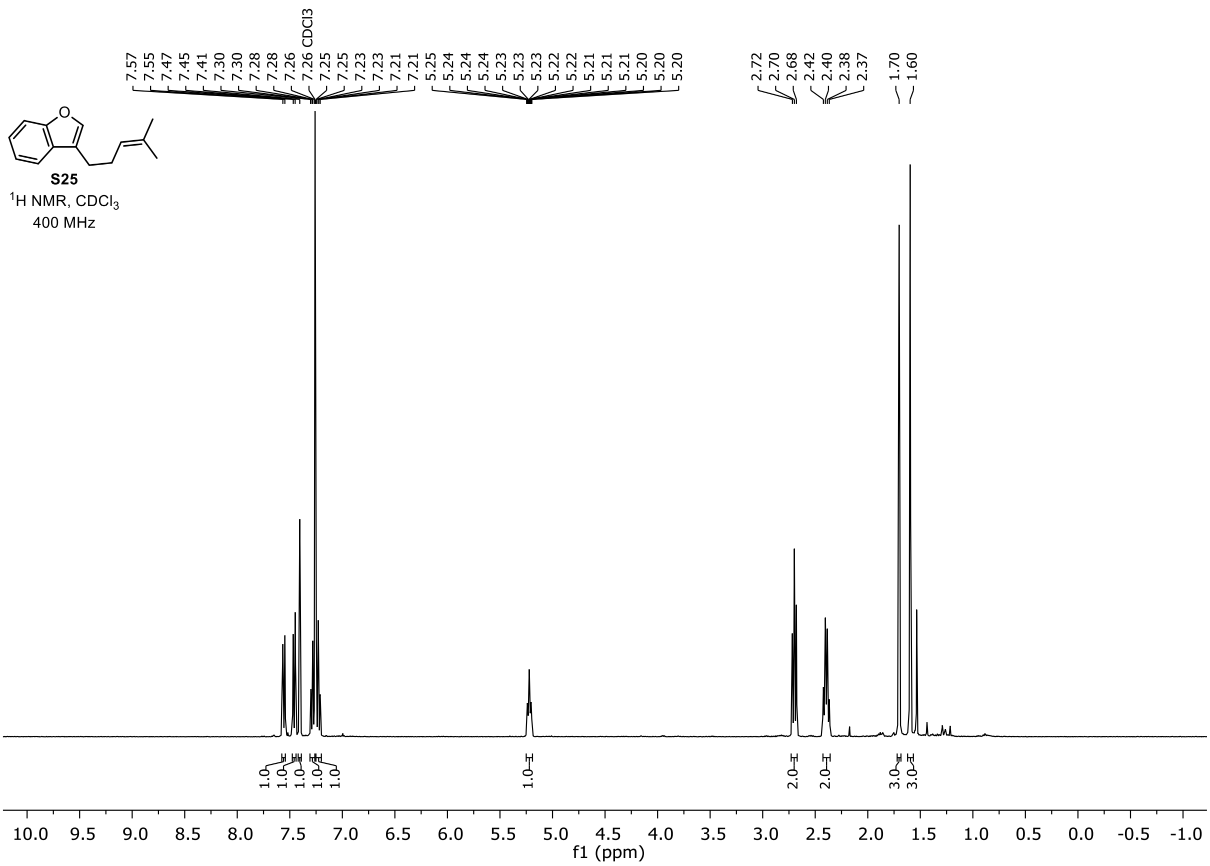

Figure 149. ^{1}H-NMR (400 MHz, CDCl$_3$) of alkene **S25**.

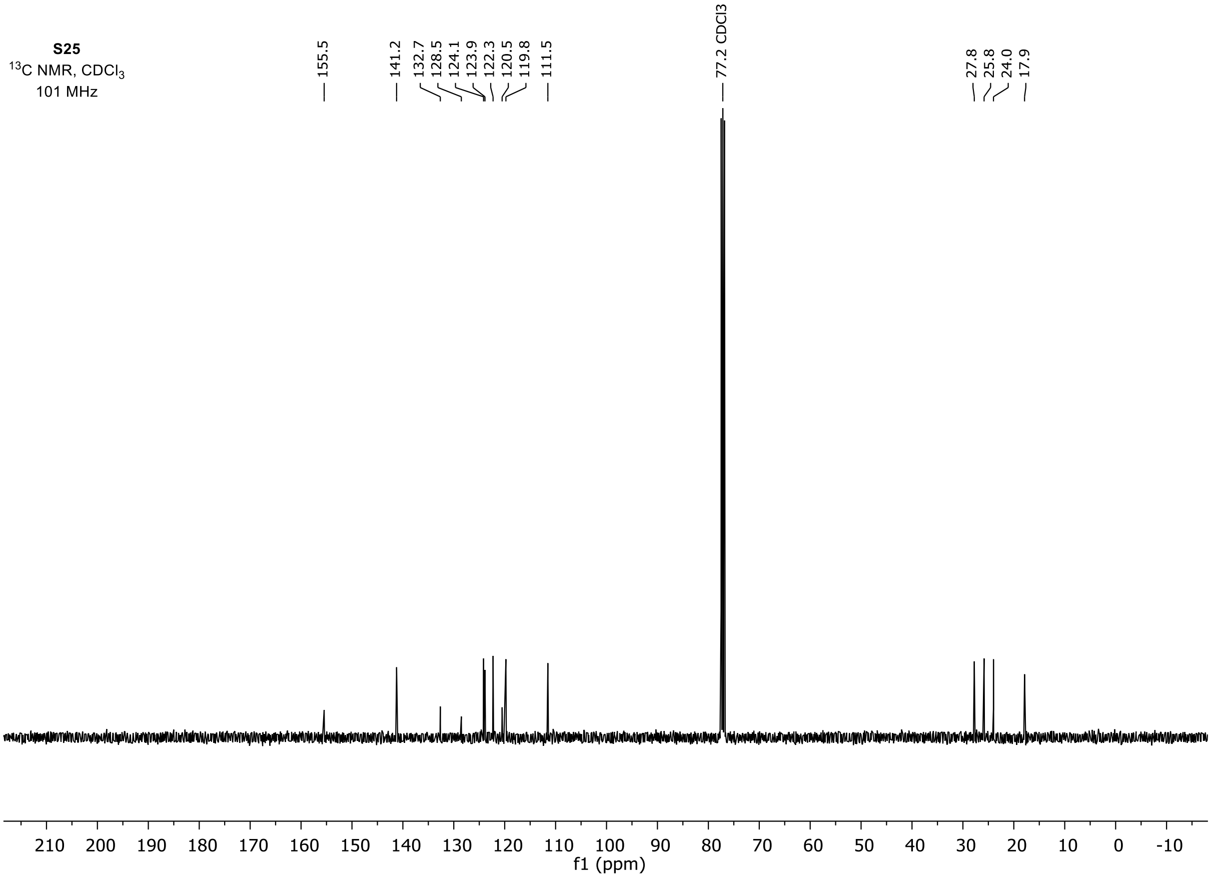

Figure 150. ^{13}C-NMR (101 MHz, CDCl$_3$) of alkene **S25**.

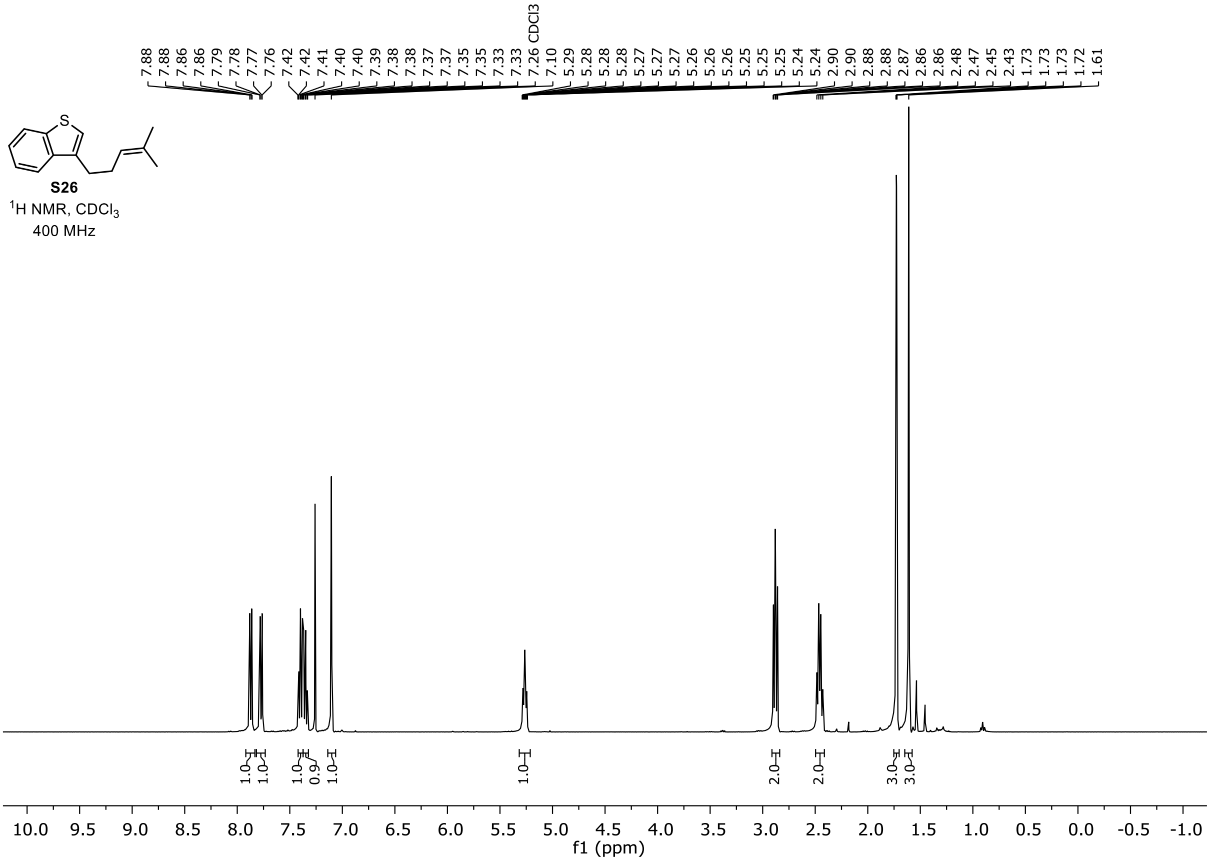

Figure 151. ^{1}H-NMR (400 MHz, $CDCl_3$) of alkene **S26**.

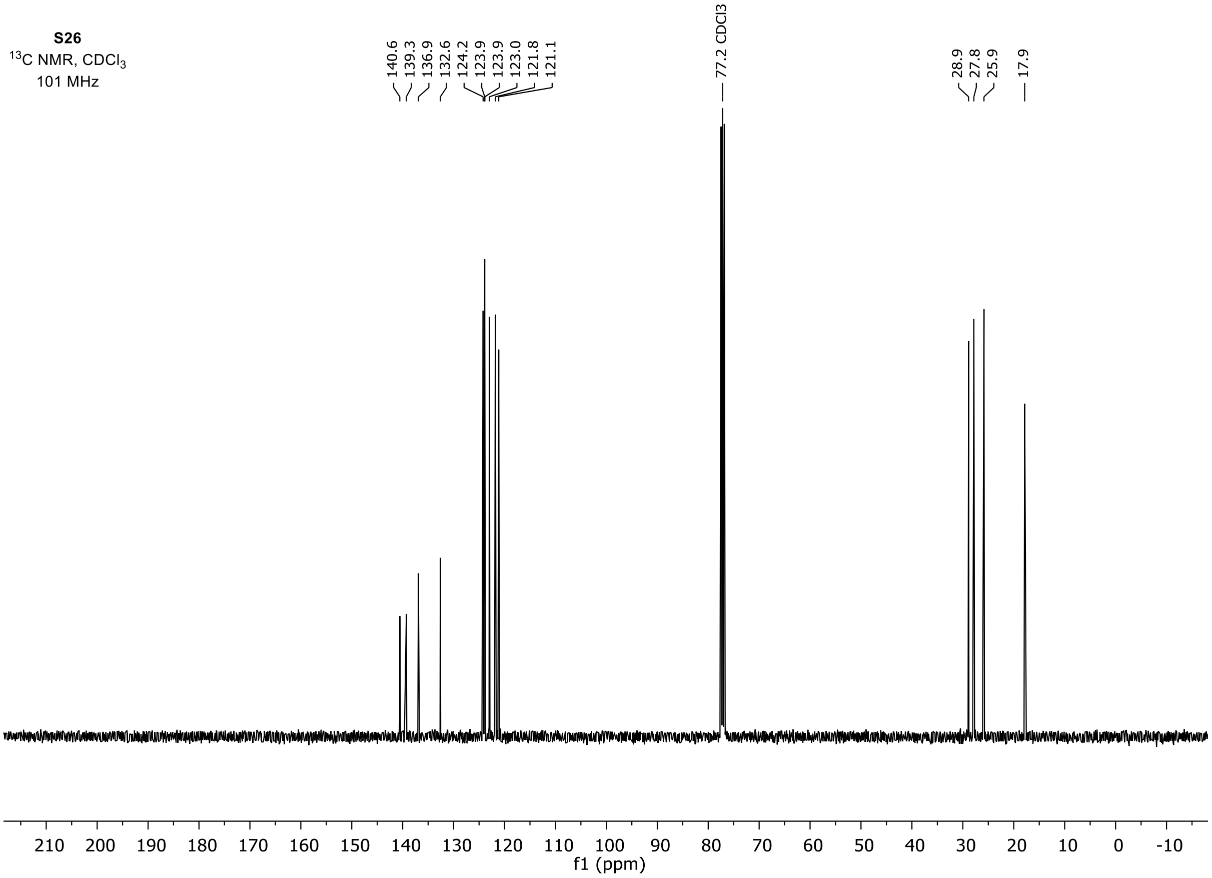

Figure 152. ^{13}C-NMR (101 MHz, $CDCl_3$) of alkene **S26**.

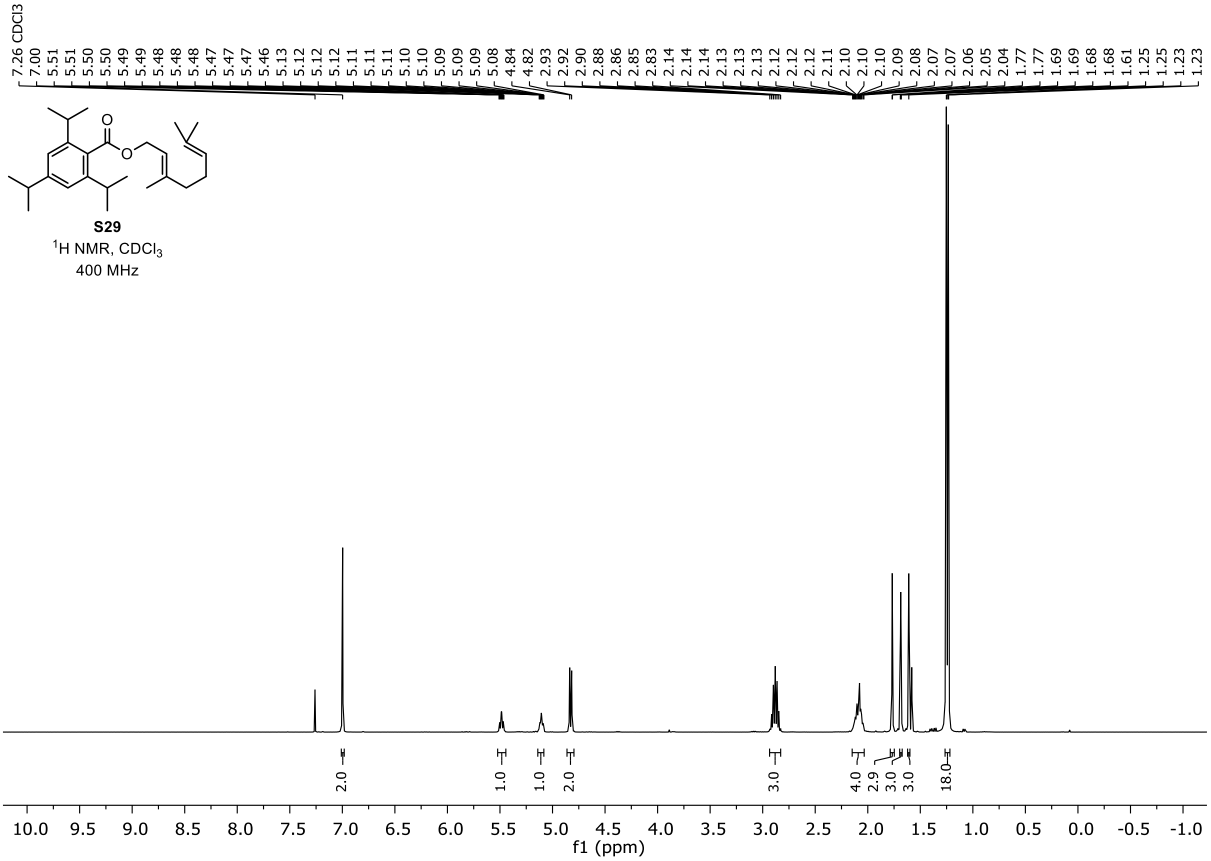

Figure 153. ^{1}H-NMR (400 MHz, $CDCl_3$) of TIB-protected geraniol **S29**.

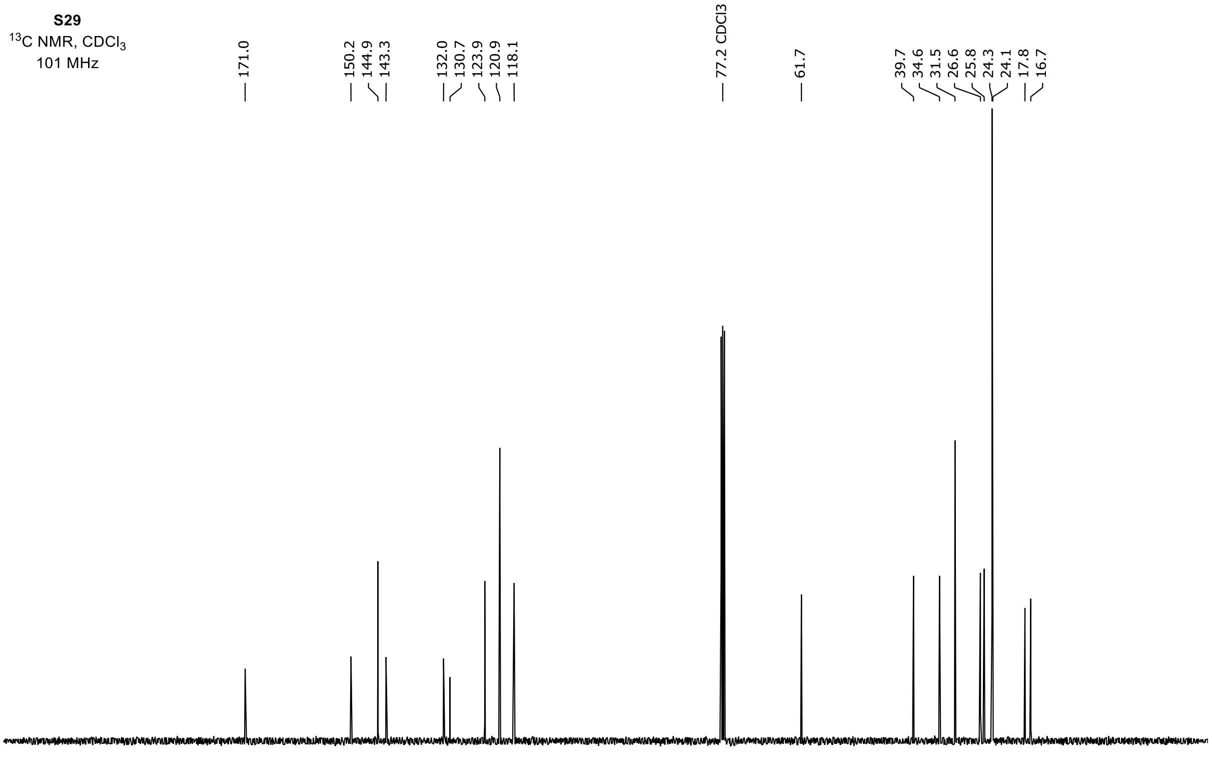

Figure 154. ^{13}C-NMR (101 MHz, $CDCl_3$) of TIB-protected geraniol **S29**.

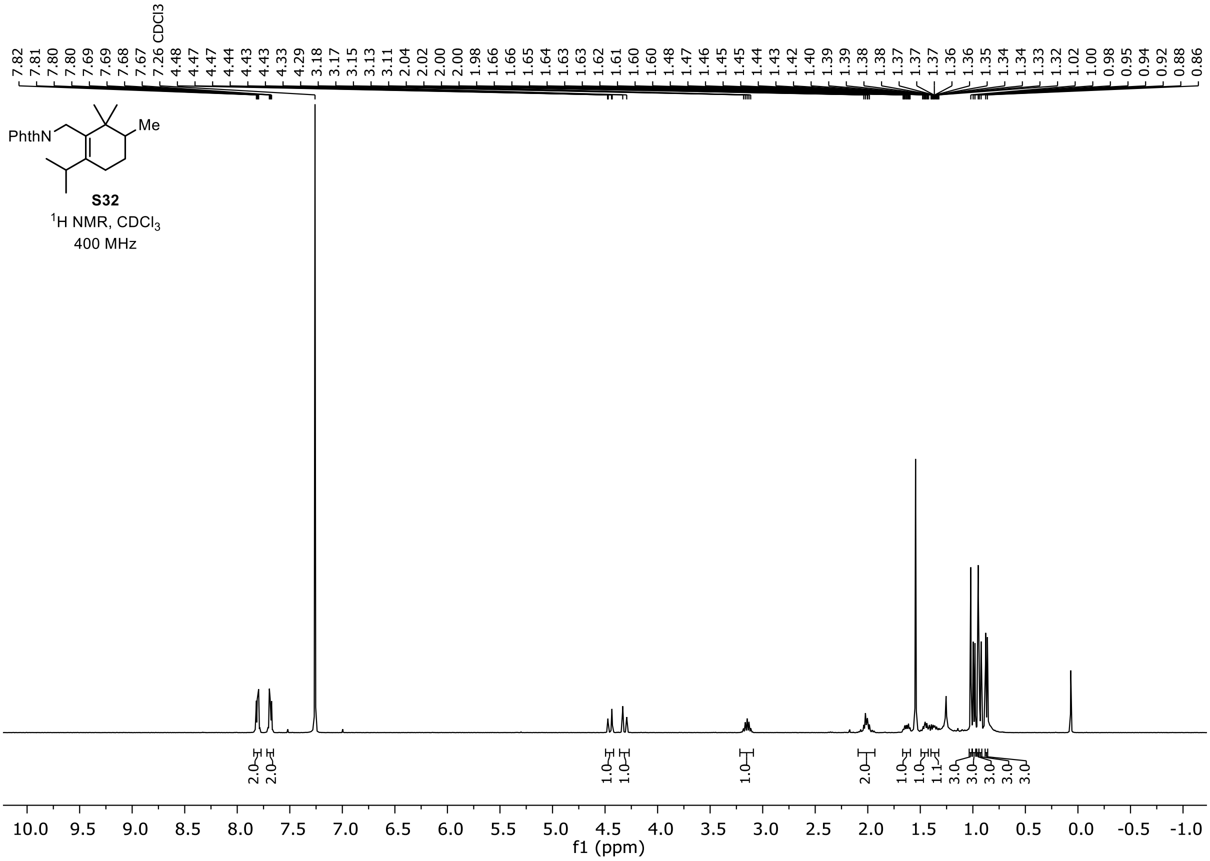

Figure 155. ^{1}H-NMR (400 MHz, $CDCl_3$) of phthalimide **S32**.

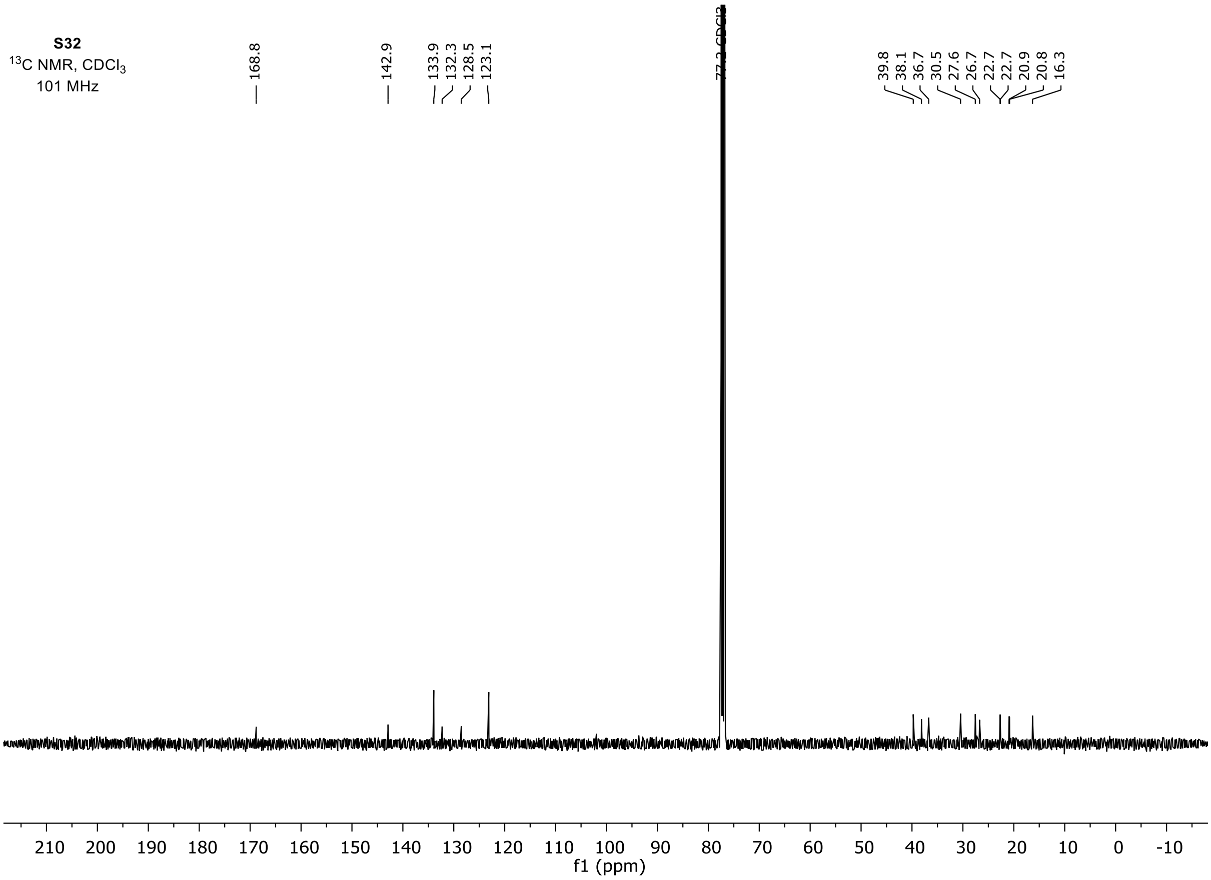

Figure 156. ^{13}C-NMR (101 MHz, $CDCl_3$) of phthalimide **S32**.

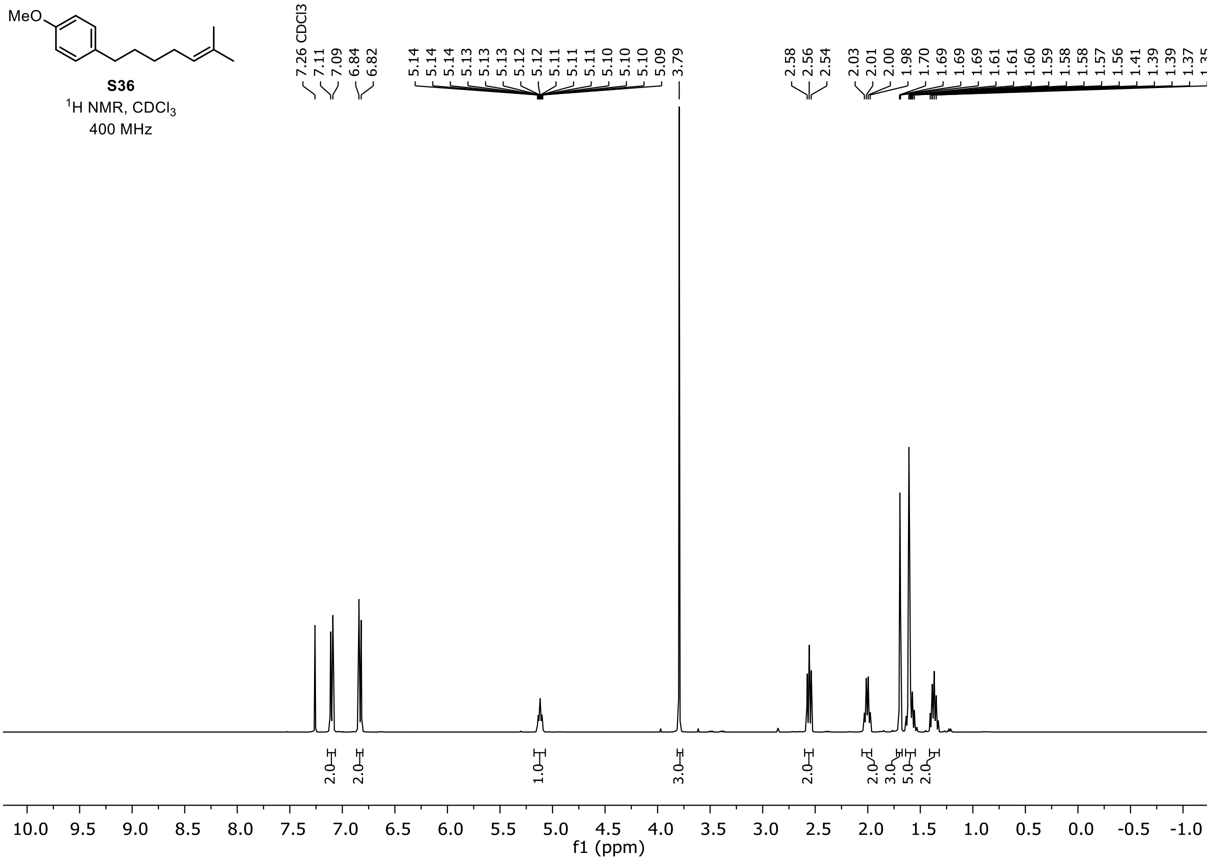

Figure 157. ^{1}H-NMR (400 MHz, $CDCl_3$) of alkene **S36**.

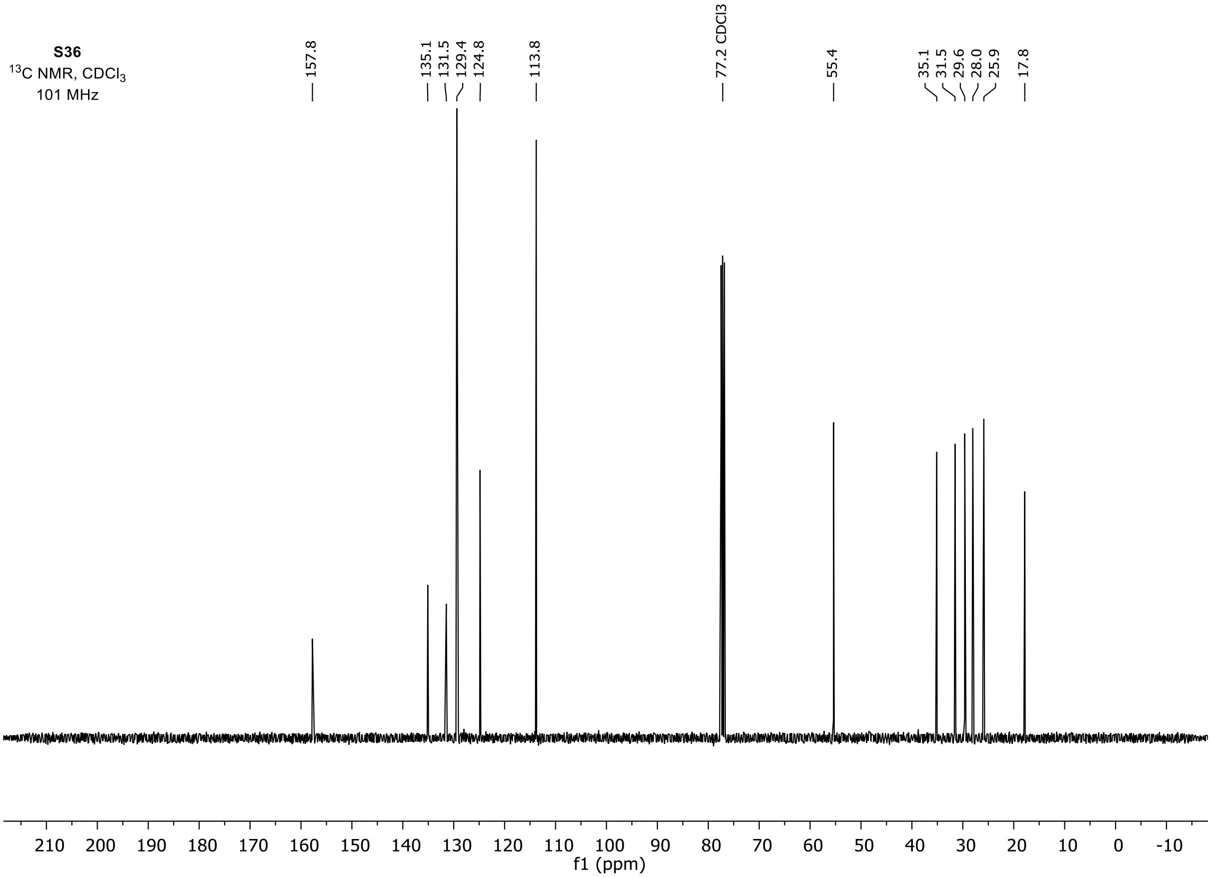

Figure 158. ^{13}C-NMR (101 MHz, $CDCl_3$) of alkene **S36**.

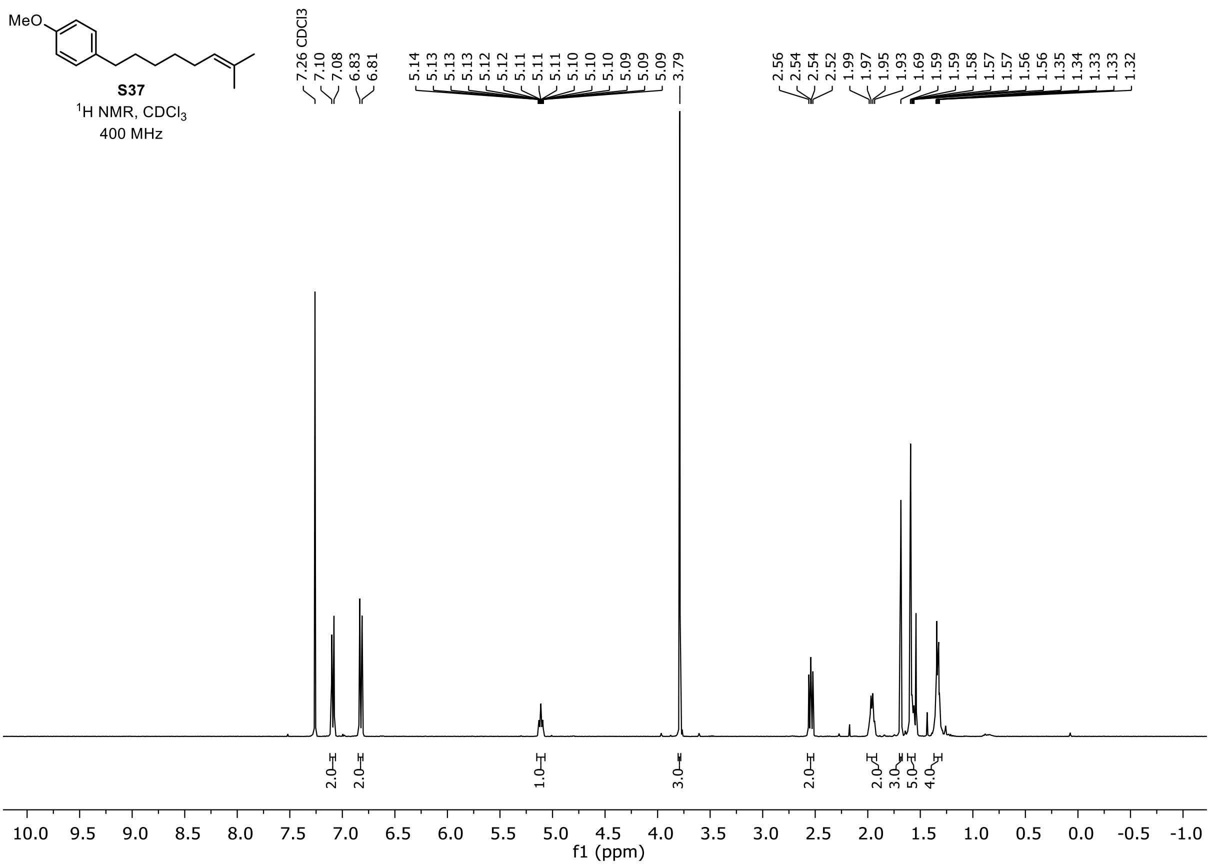

Figure 159. ^{1}H-NMR (400 MHz, $CDCl_3$) of alkene **S37**.

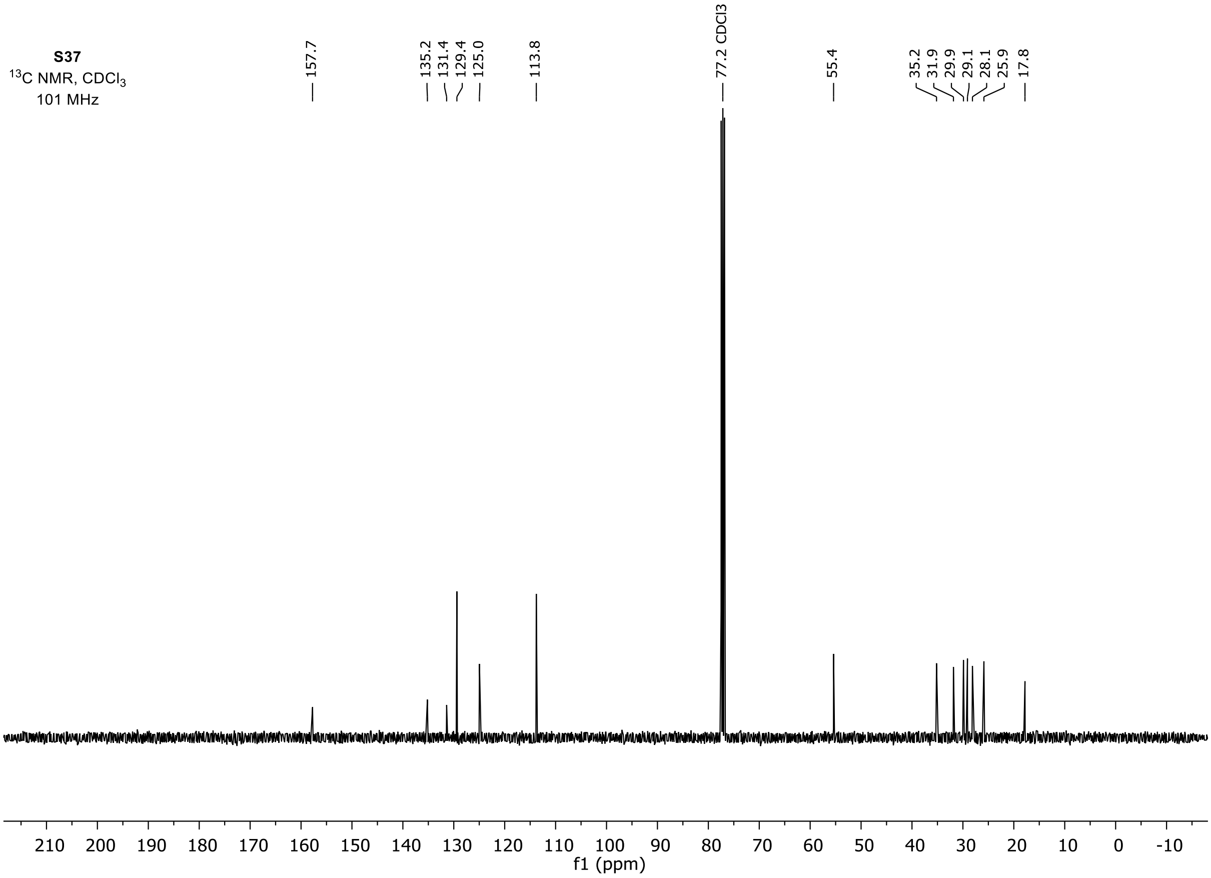

Figure 160. ^{13}C-NMR (101 MHz, $CDCl_3$) of alkene **S37**.

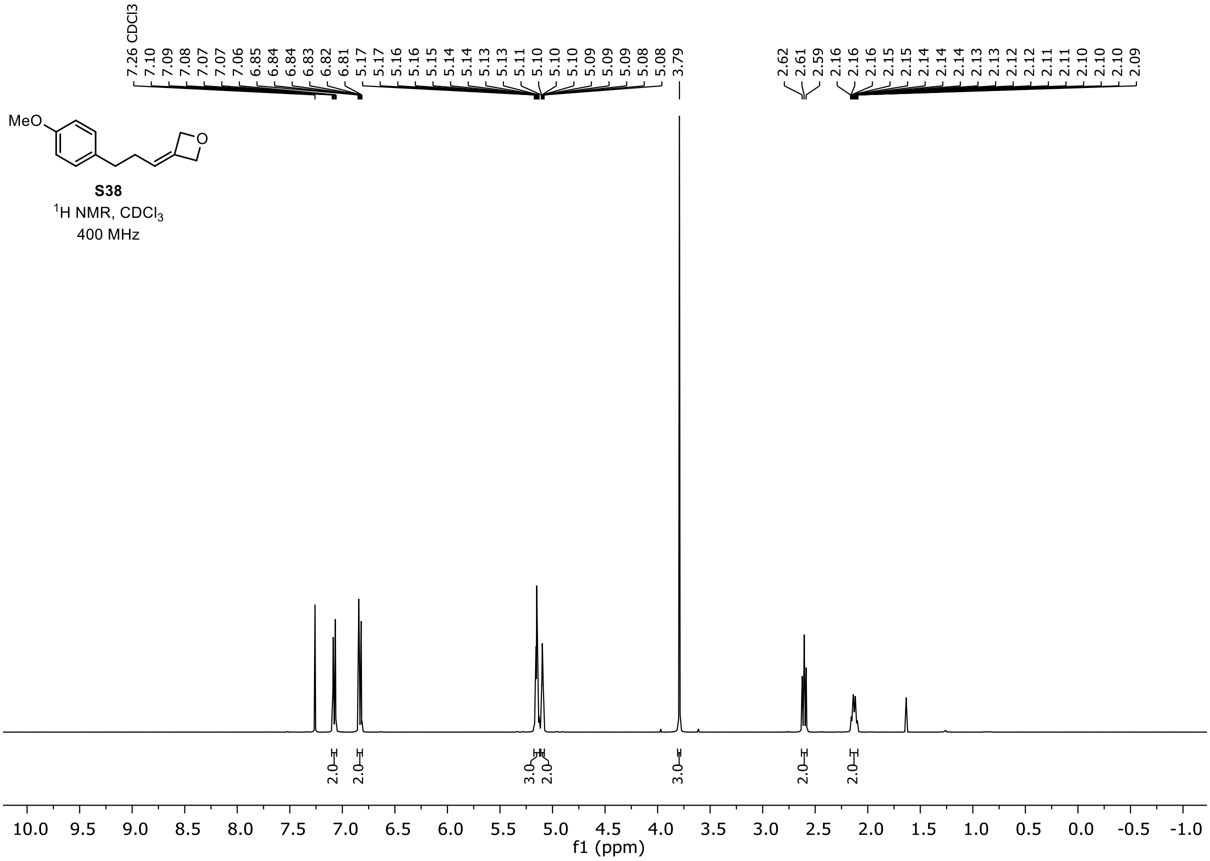

Figure 161. ^{1}H-NMR (400 MHz, $CDCl_3$) of alkene **S38**.

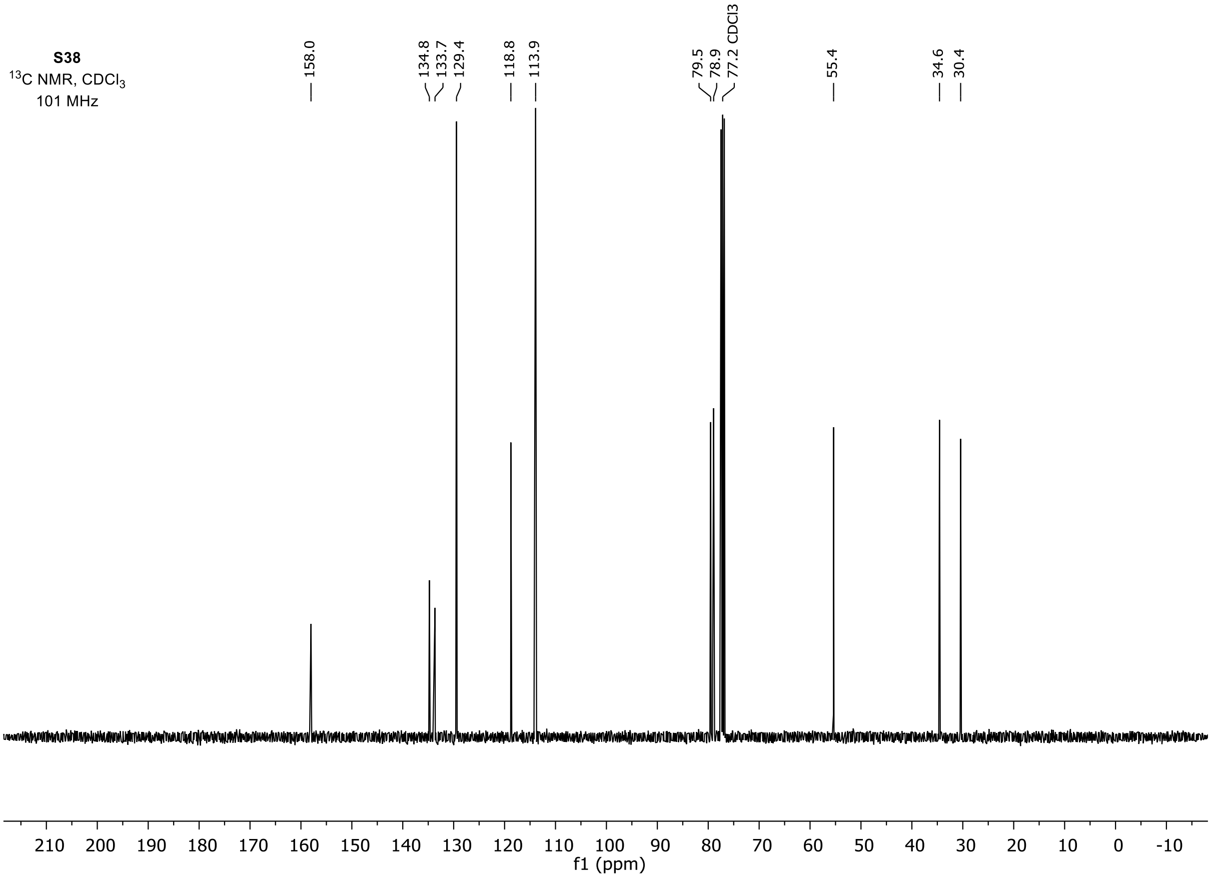

Figure 162. ^{13}C-NMR (101 MHz, $CDCl_3$) of alkene **S38**.

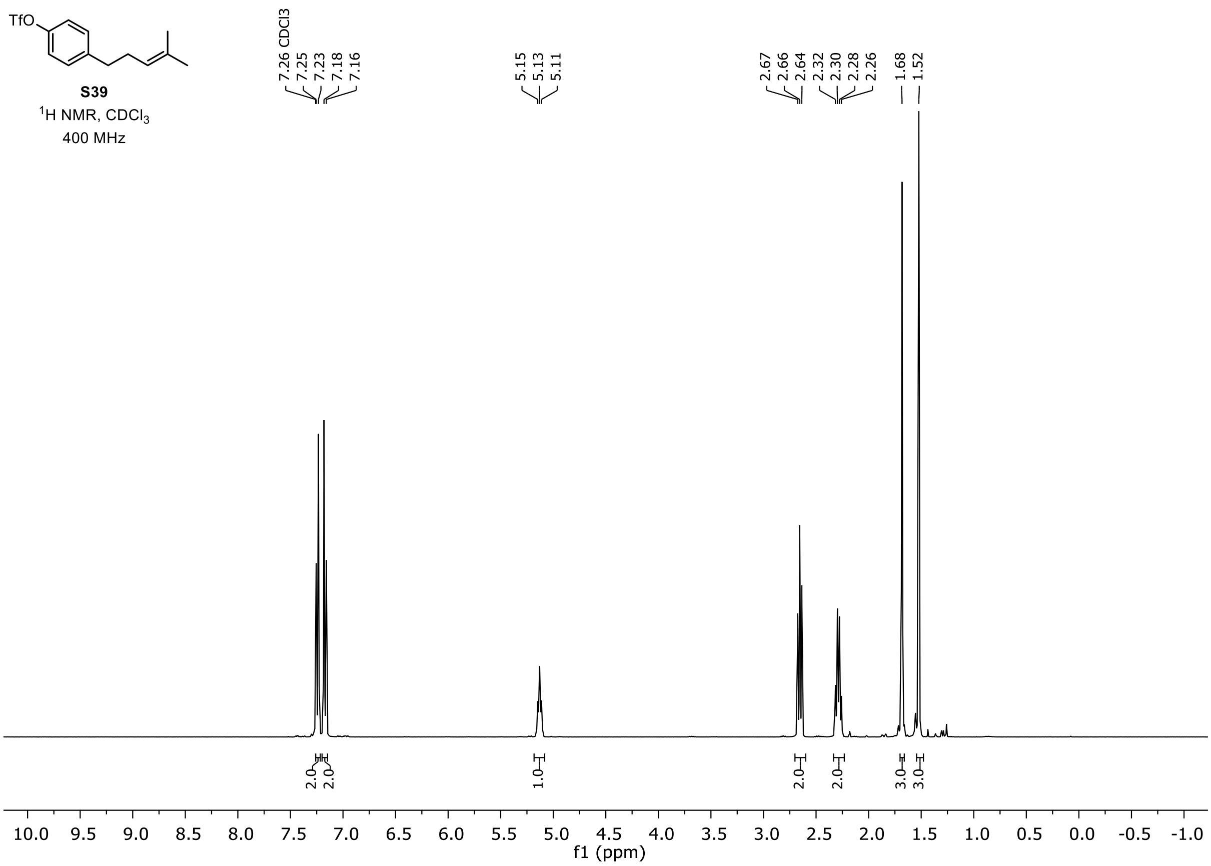

Figure 163. ^{1}H-NMR (400 MHz, $CDCl_3$) of alkene **S39**.

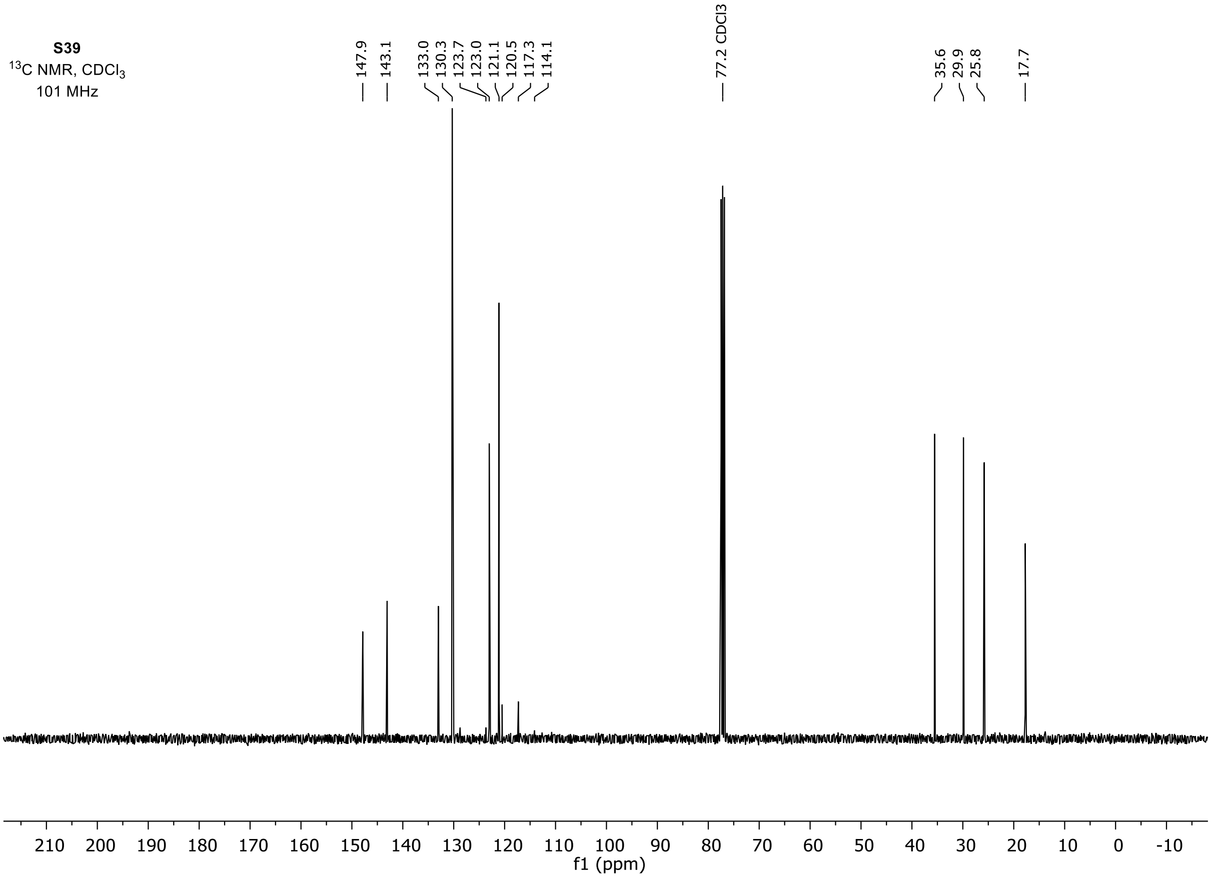

Figure 164. ^{13}C-NMR (101 MHz, $CDCl_3$) of alkene **S39**.

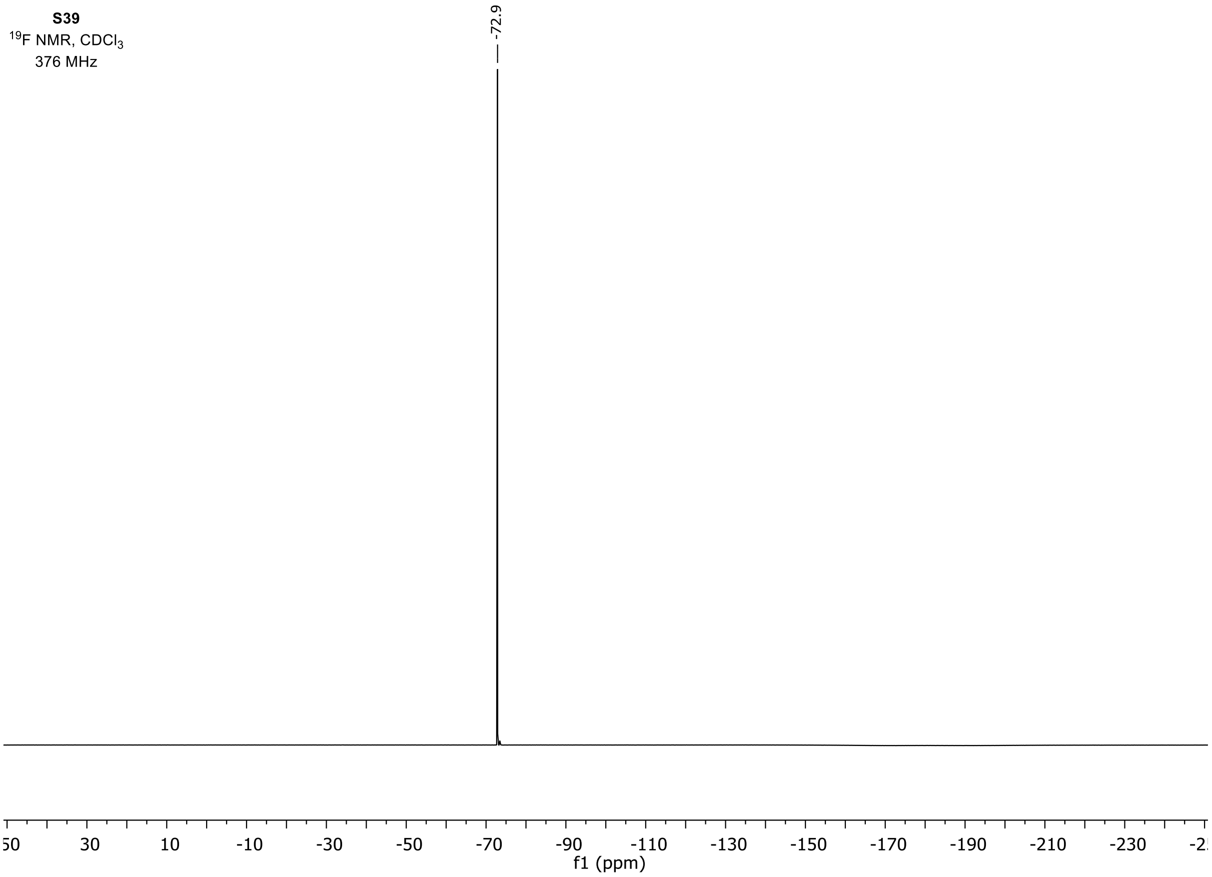

Figure 165. ^{19}F-NMR (376 MHz, $CDCl_3$) of alkene **S39**.

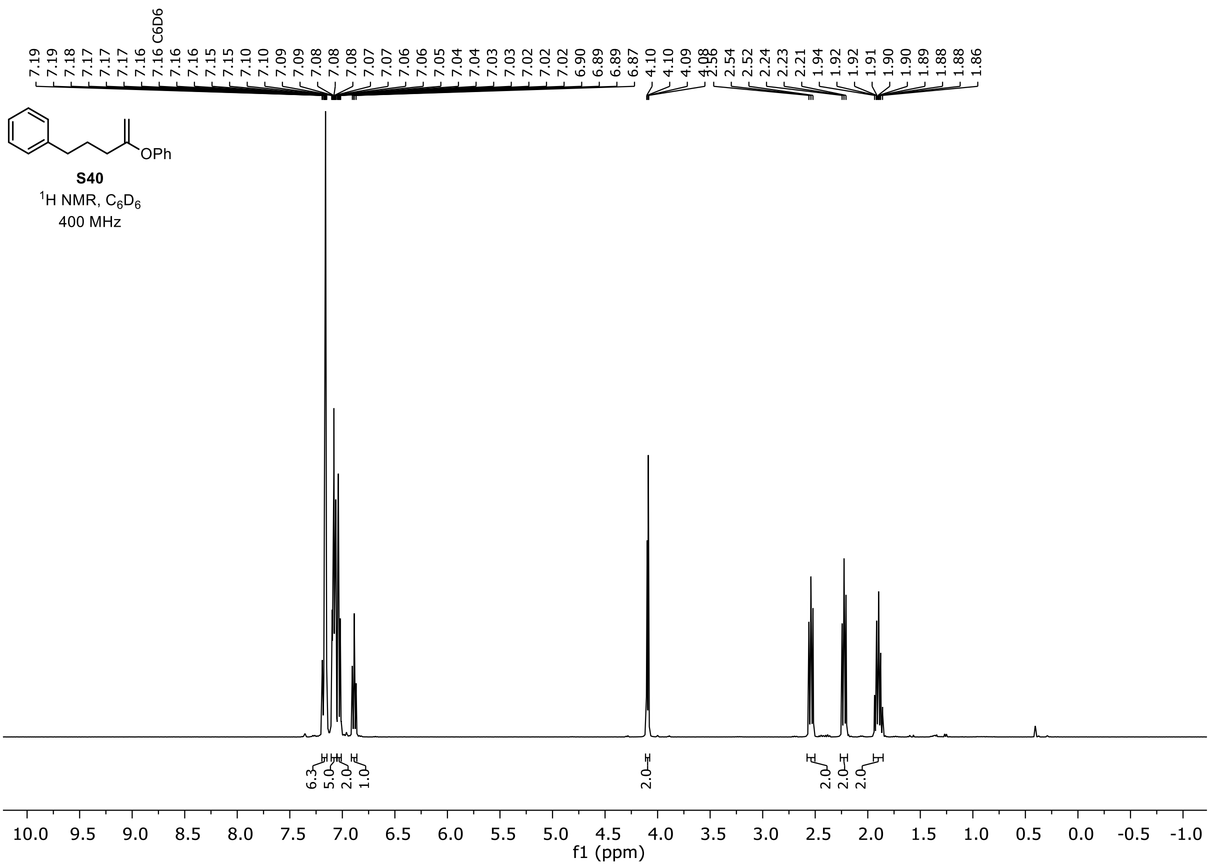

Figure 166. ^{1}H-NMR (400 MHz, C_6D_6) of enol ether **S40**.

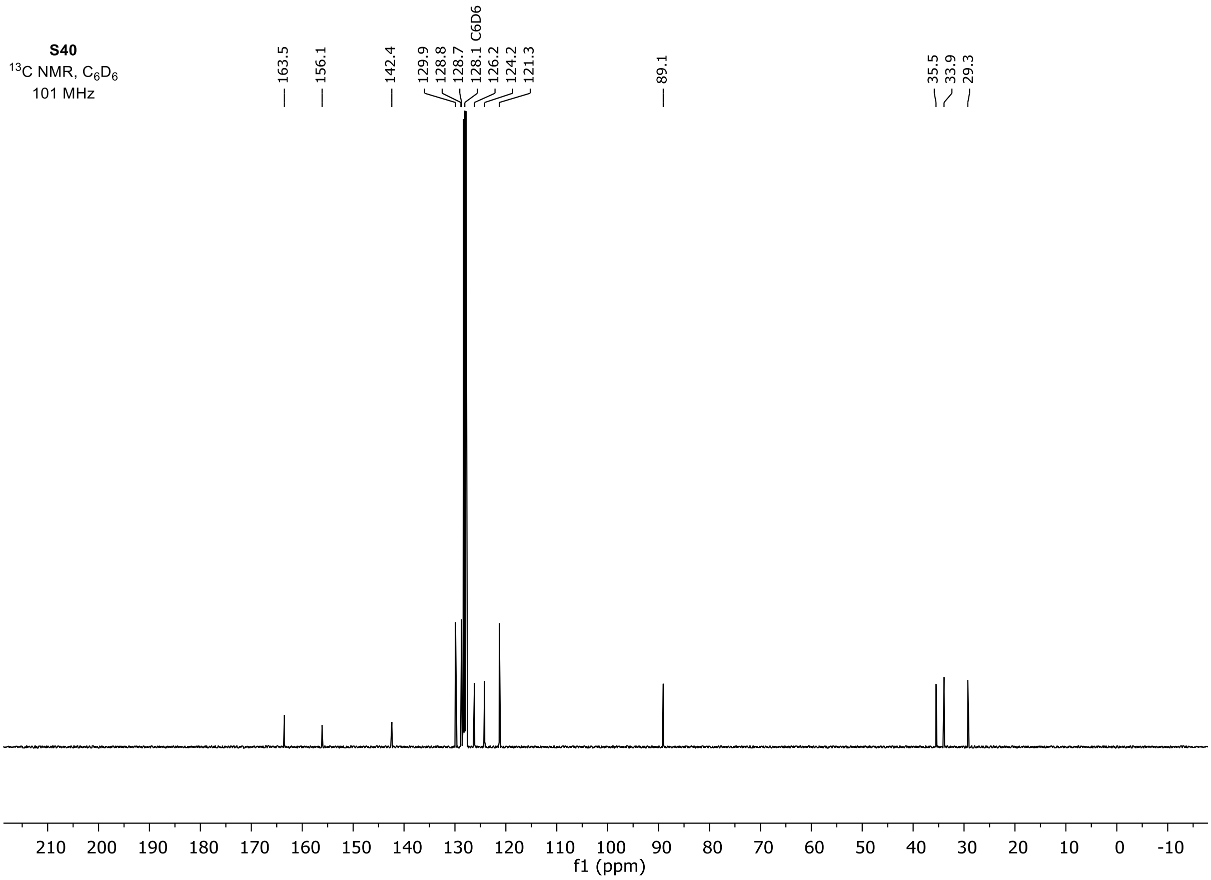

Figure 167. ^{13}C-NMR (101 MHz, C_6D_6) of enol ether **S40**.

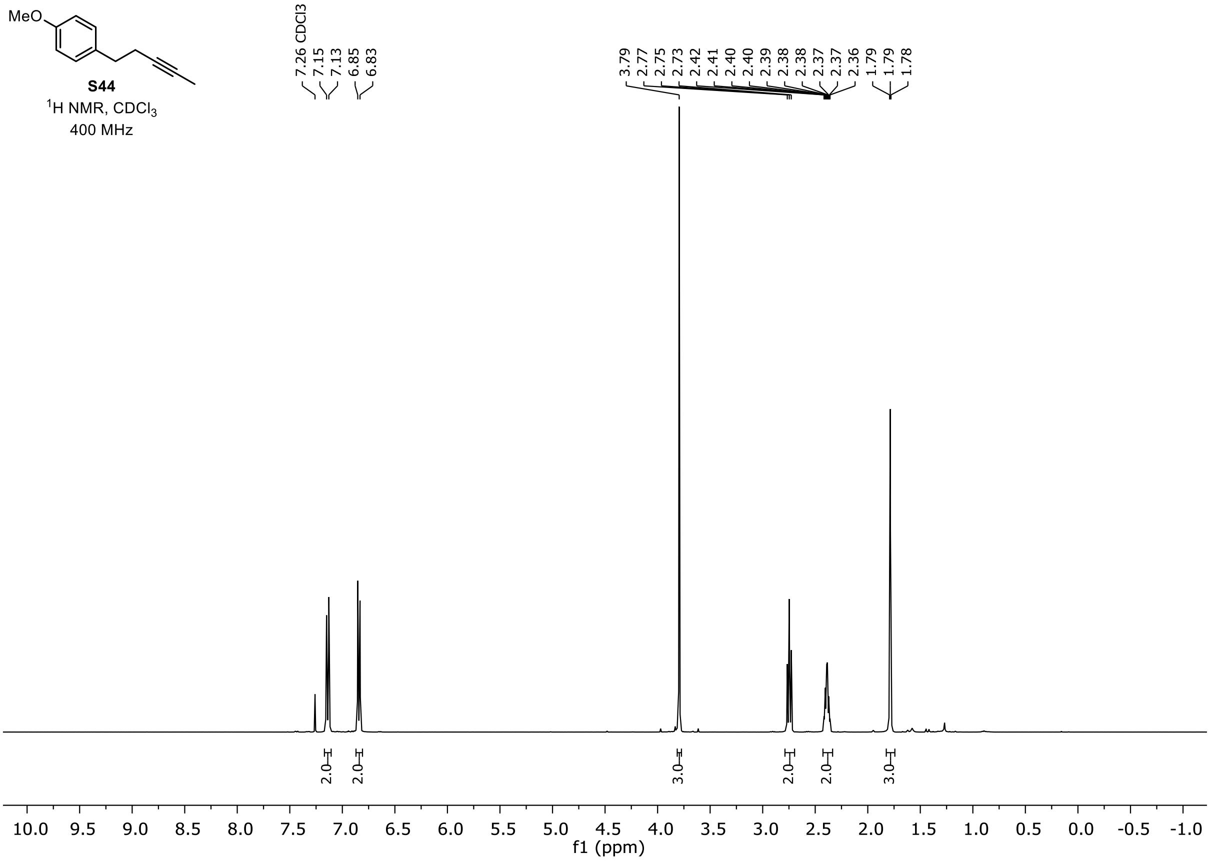

Figure 168. ^{1}H-NMR (400 MHz, $CDCl_3$) of alkyne **S44**.

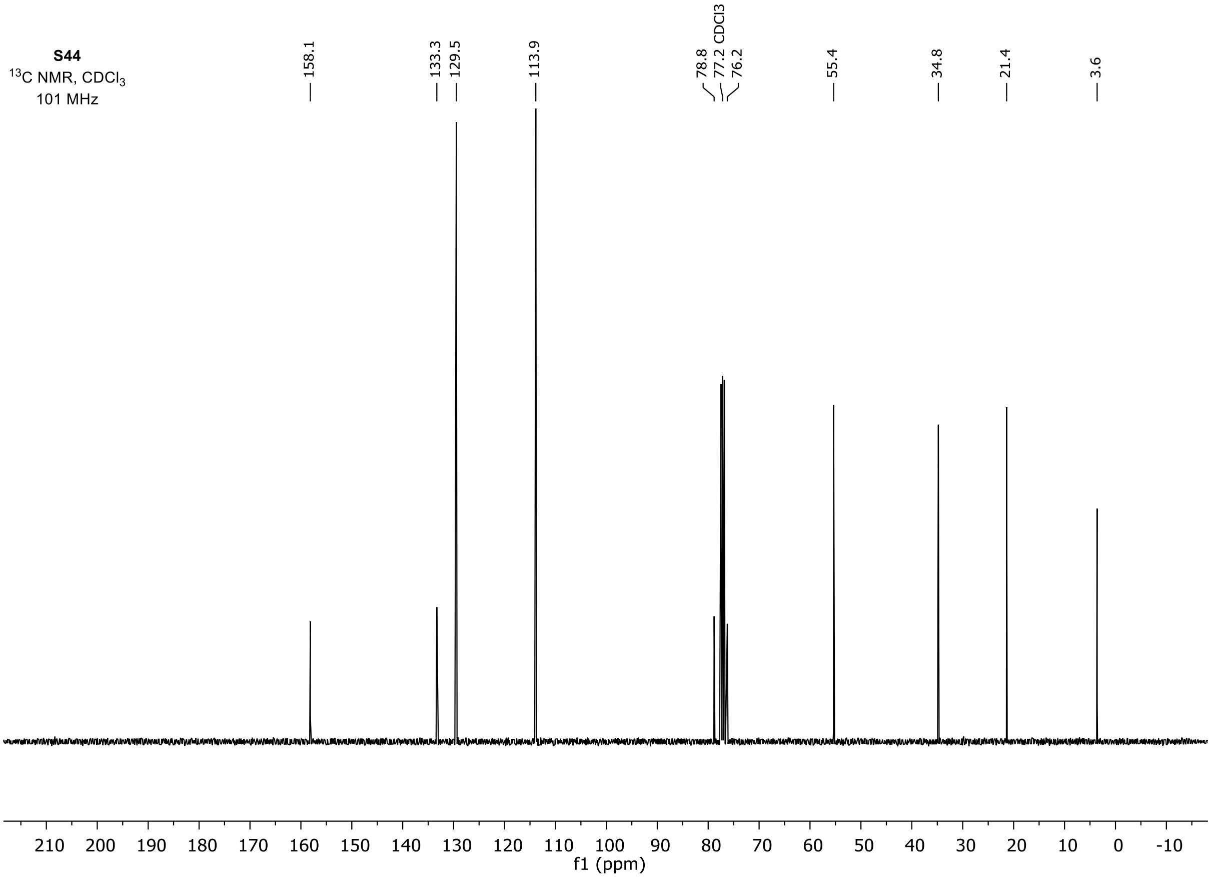

Figure 169. ^{13}C-NMR (101 MHz, $CDCl_3$) of alkyne **S44**.

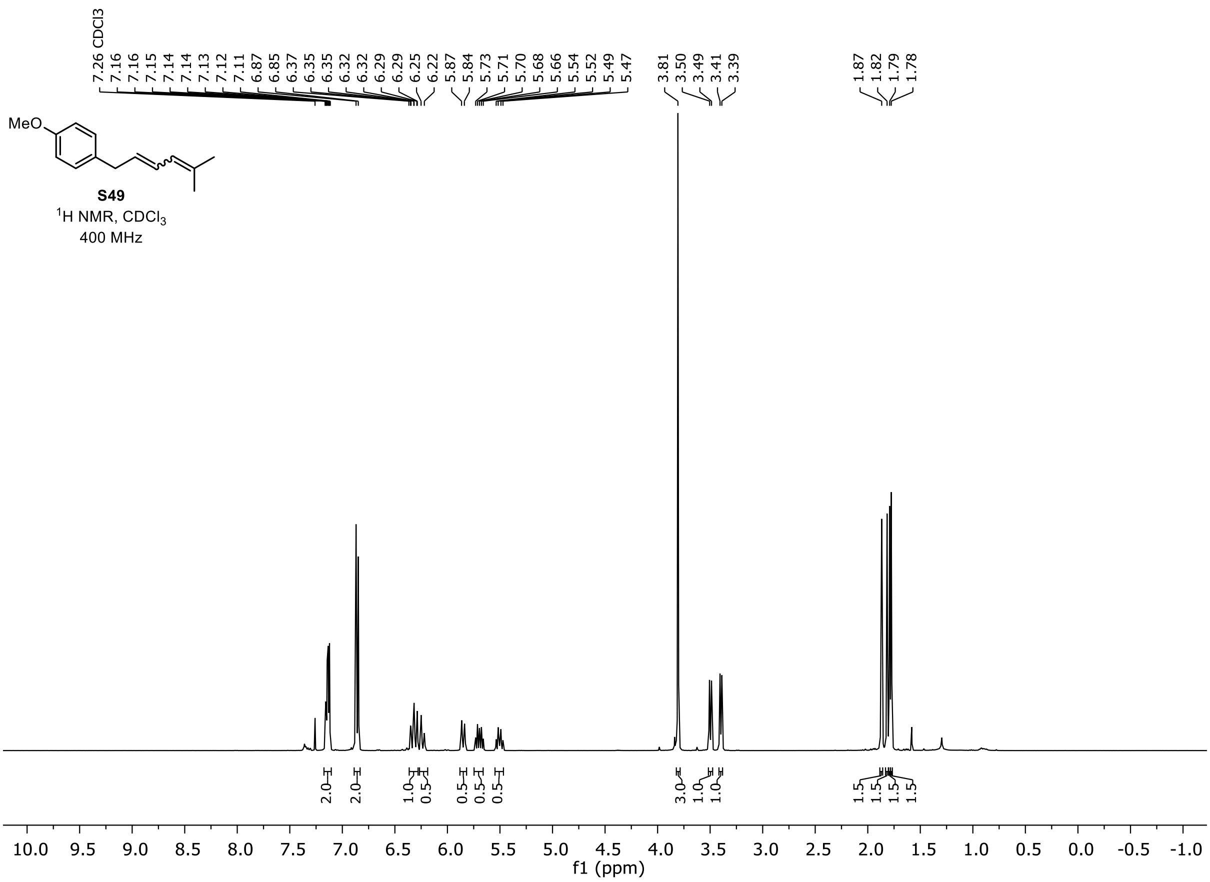

Figure 170. ^{1}H-NMR (400 MHz, $CDCl_3$) of diene **S49**.

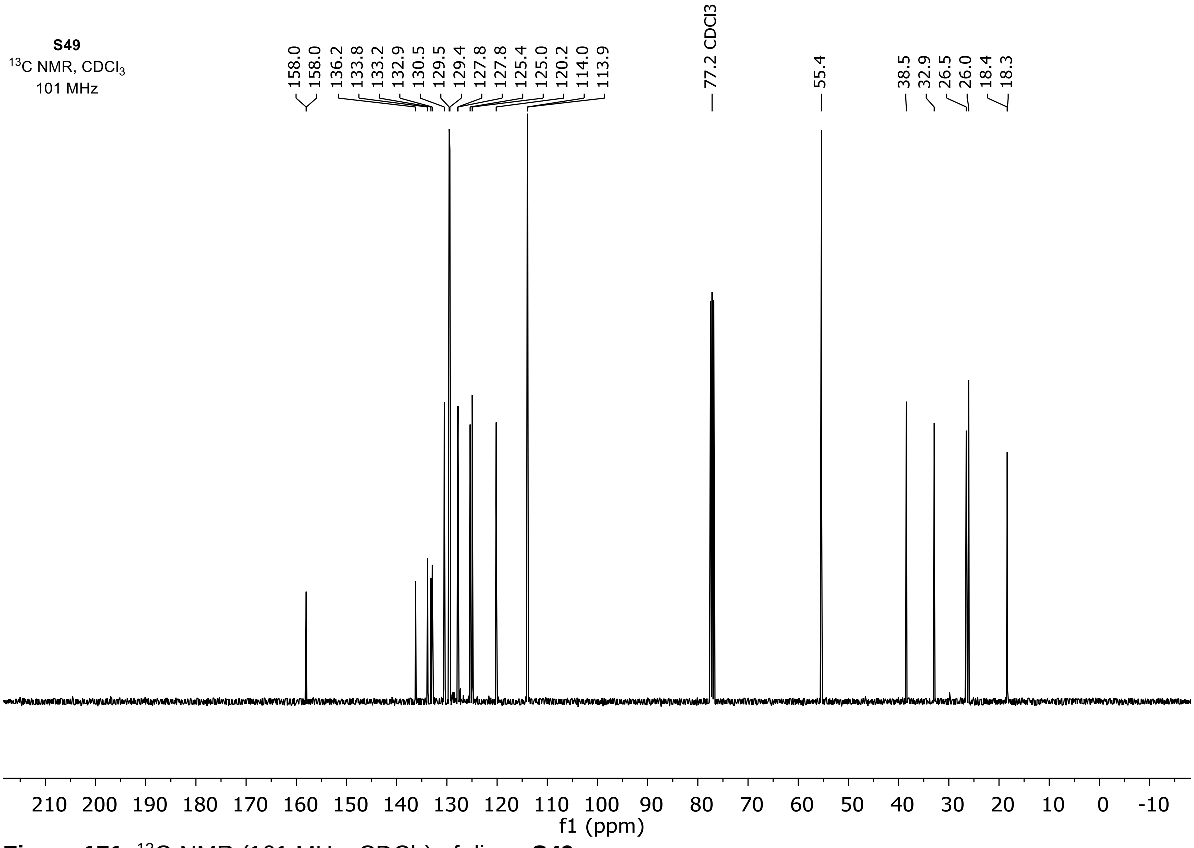

Figure 171. ^{13}C-NMR (101 MHz, $CDCl_3$) of diene **S49**.

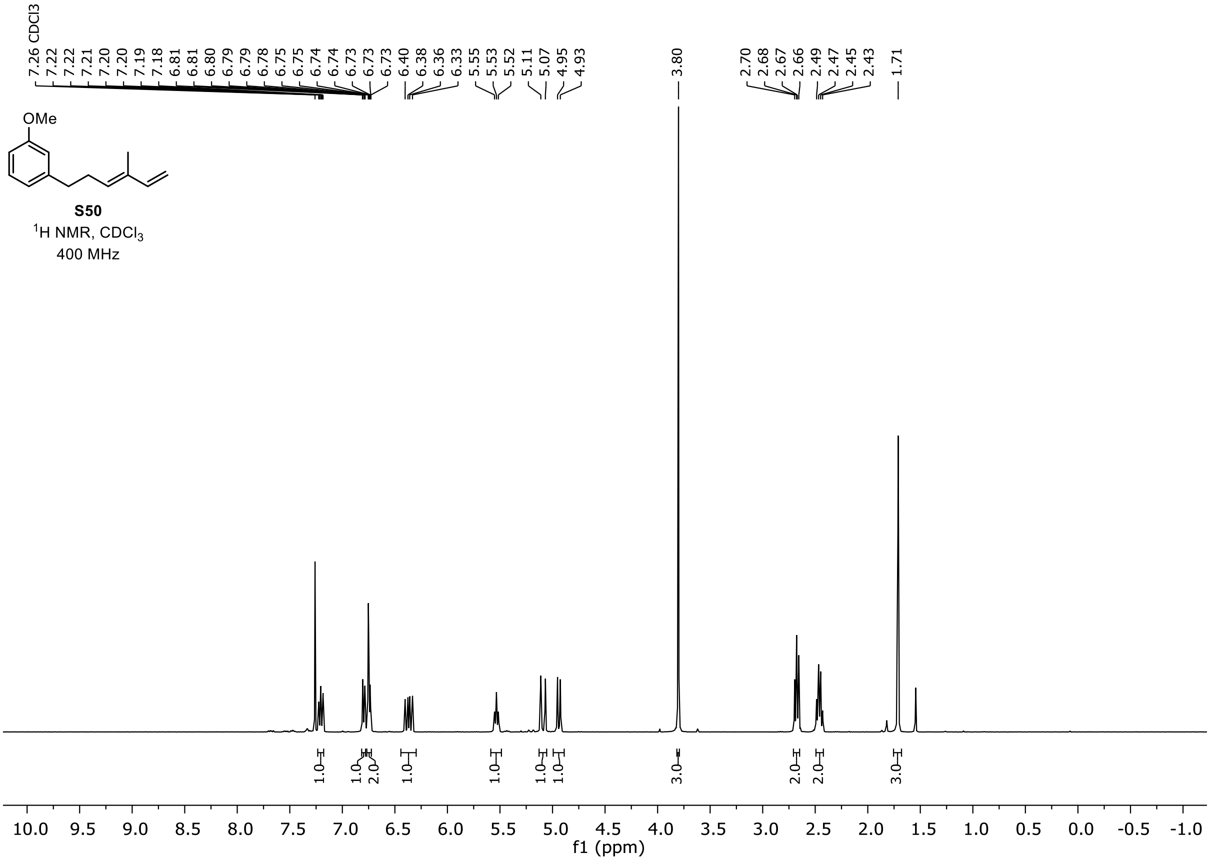

Figure 172. ^{1}H-NMR (400 MHz, $CDCl_3$) of diene **S50**.

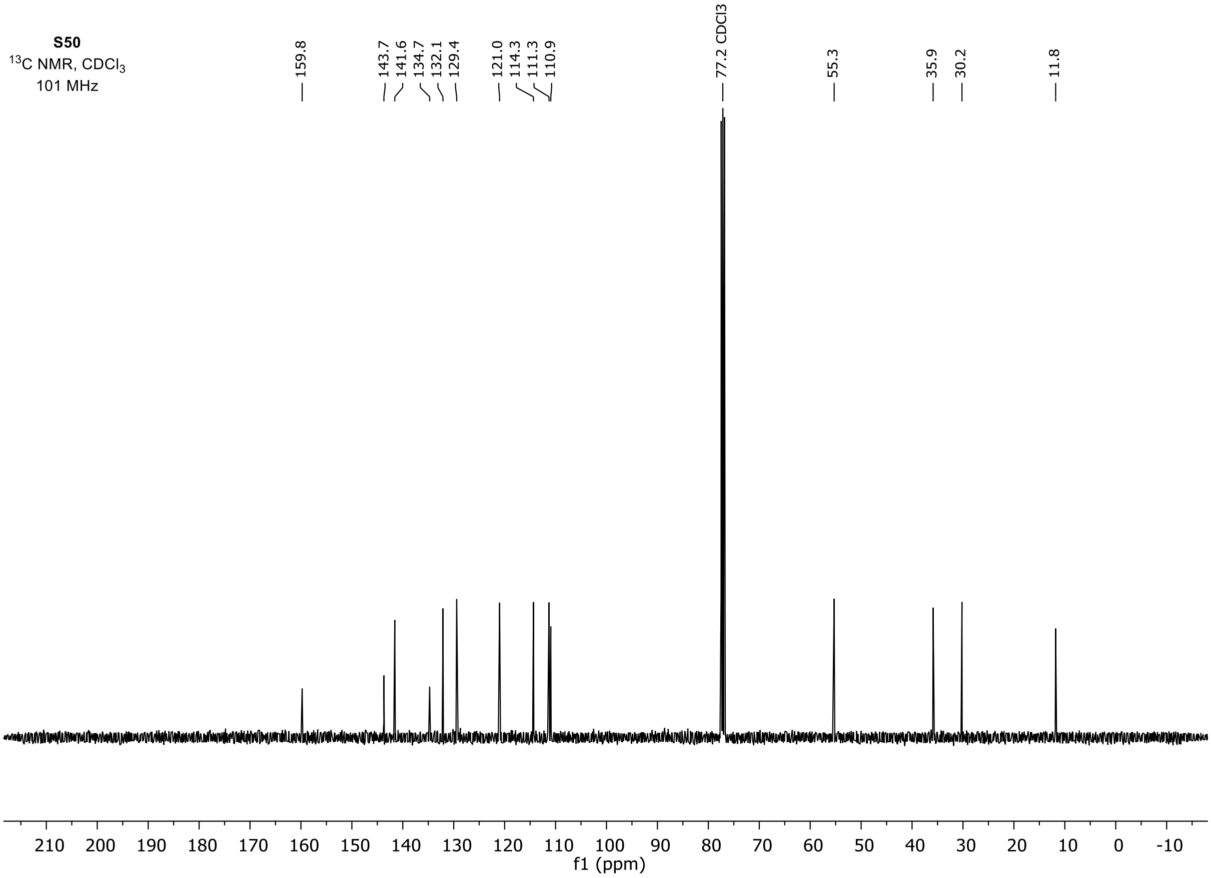

Figure 173. ^{13}C-NMR (101 MHz, $CDCl_3$) of diene **S50**.

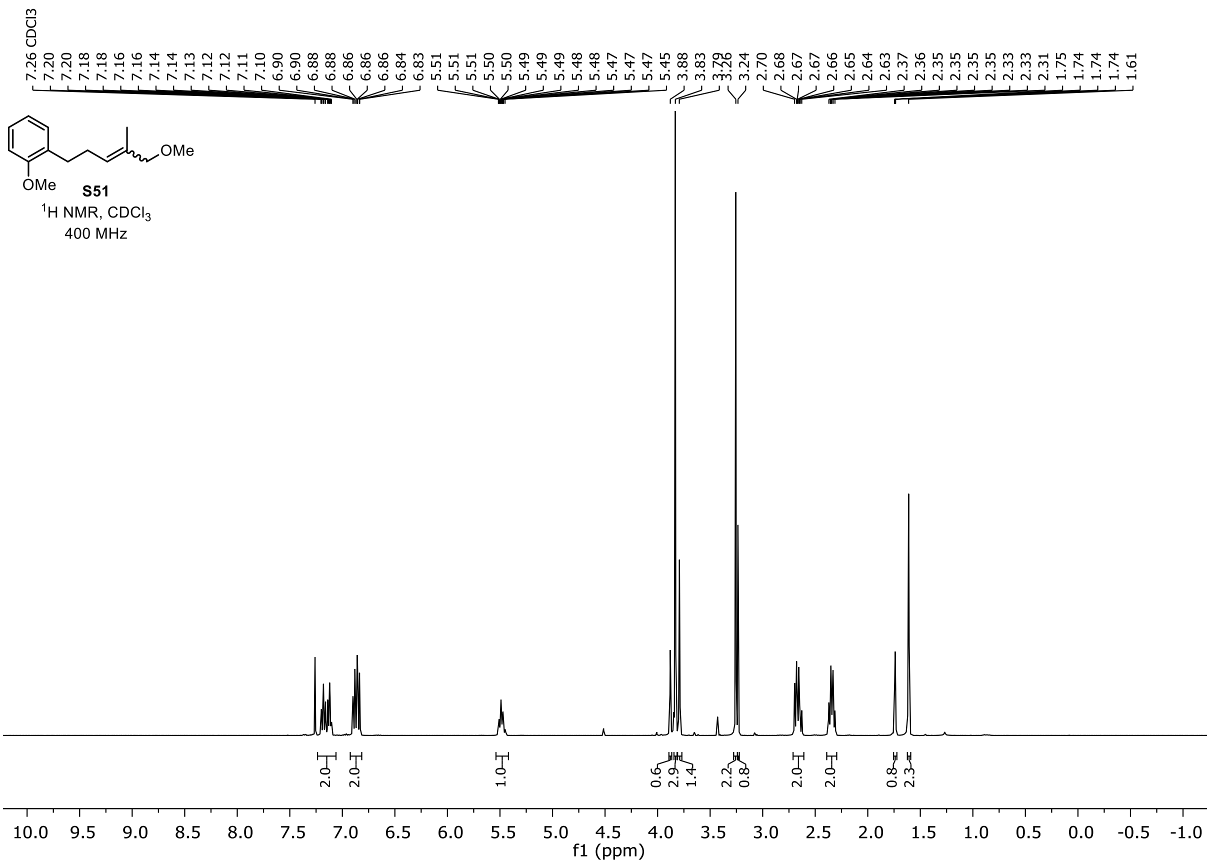

Figure 174. ^{1}H-NMR (400 MHz, $CDCl_3$) of alkene **S51**.

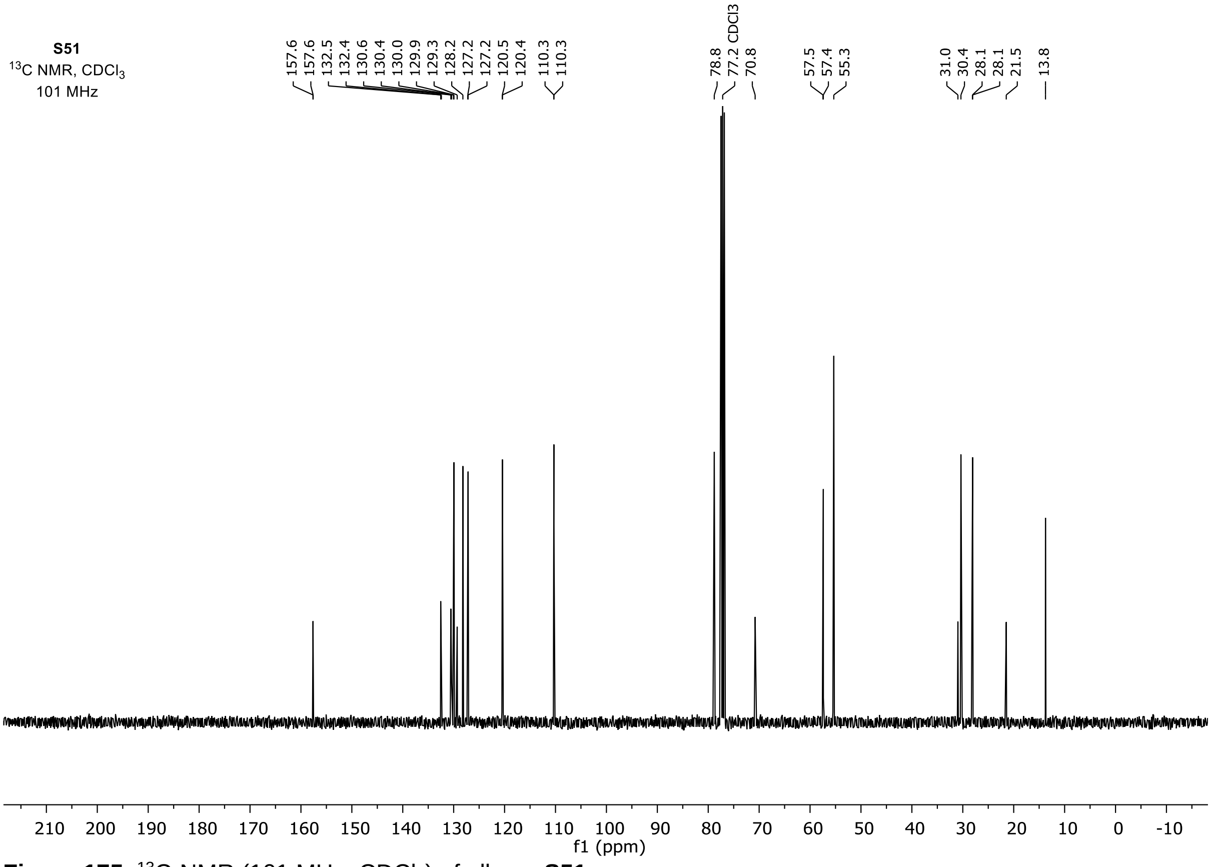

Figure 175. ^{13}C-NMR (101 MHz, $CDCl_3$) of alkene **S51**.

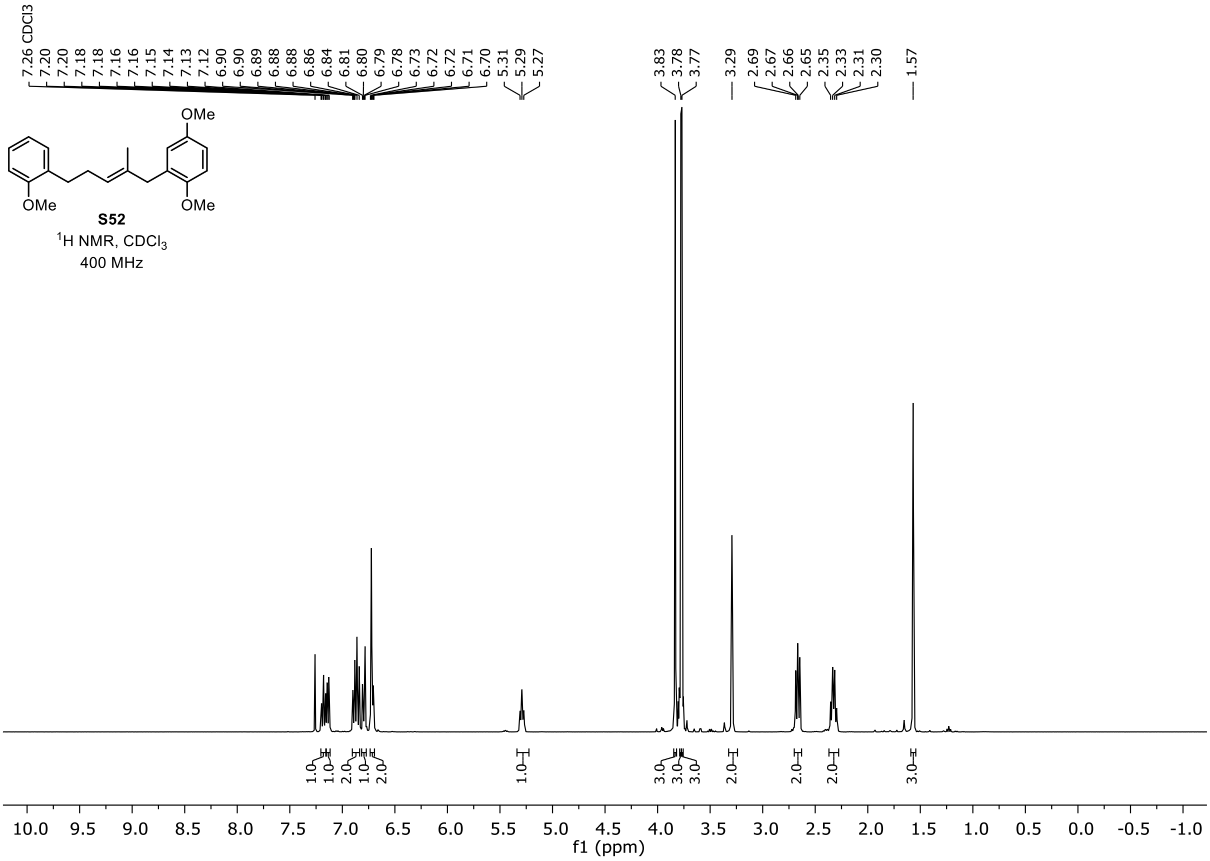

Figure 176. ^{1}H-NMR (400 MHz, $CDCl_3$) of alkene **S52**.

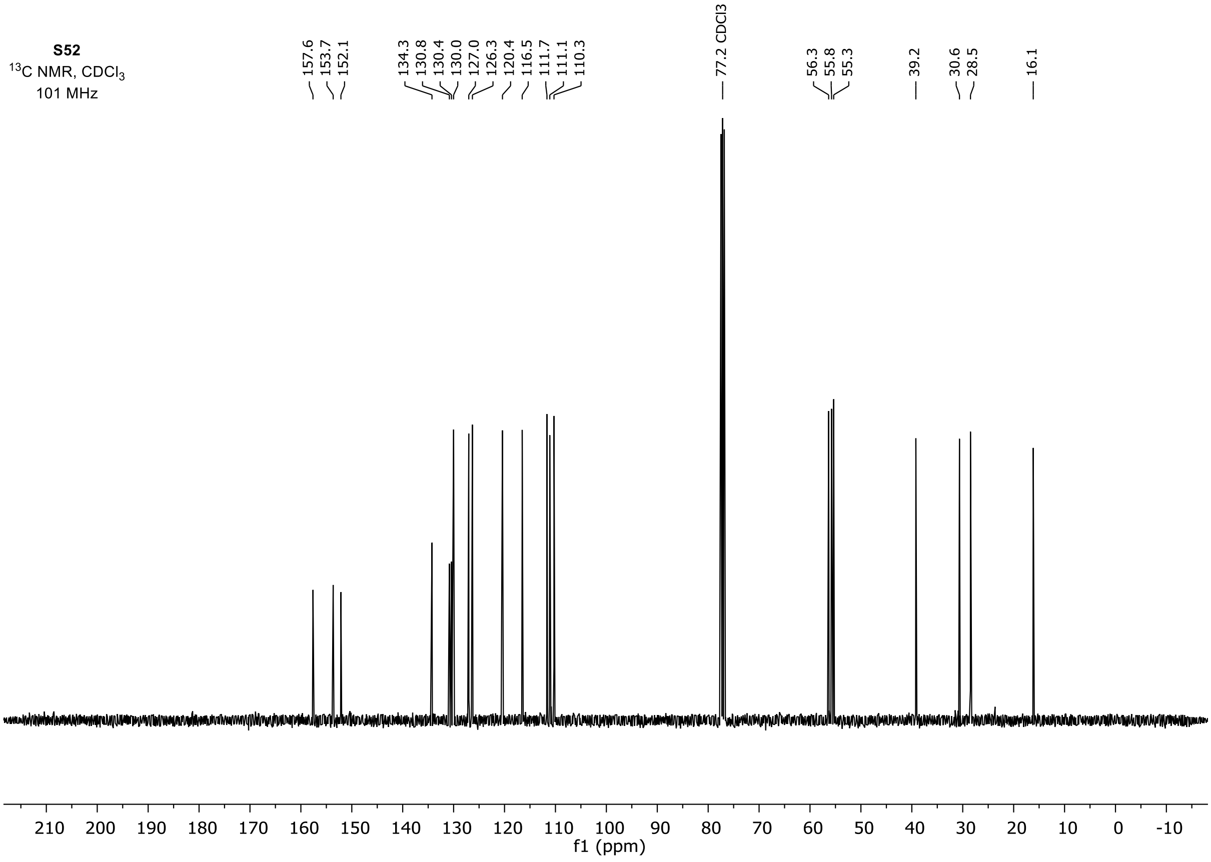

Figure 177. ^{13}C-NMR (101 MHz, $CDCl_3$) of alkene **S52**.

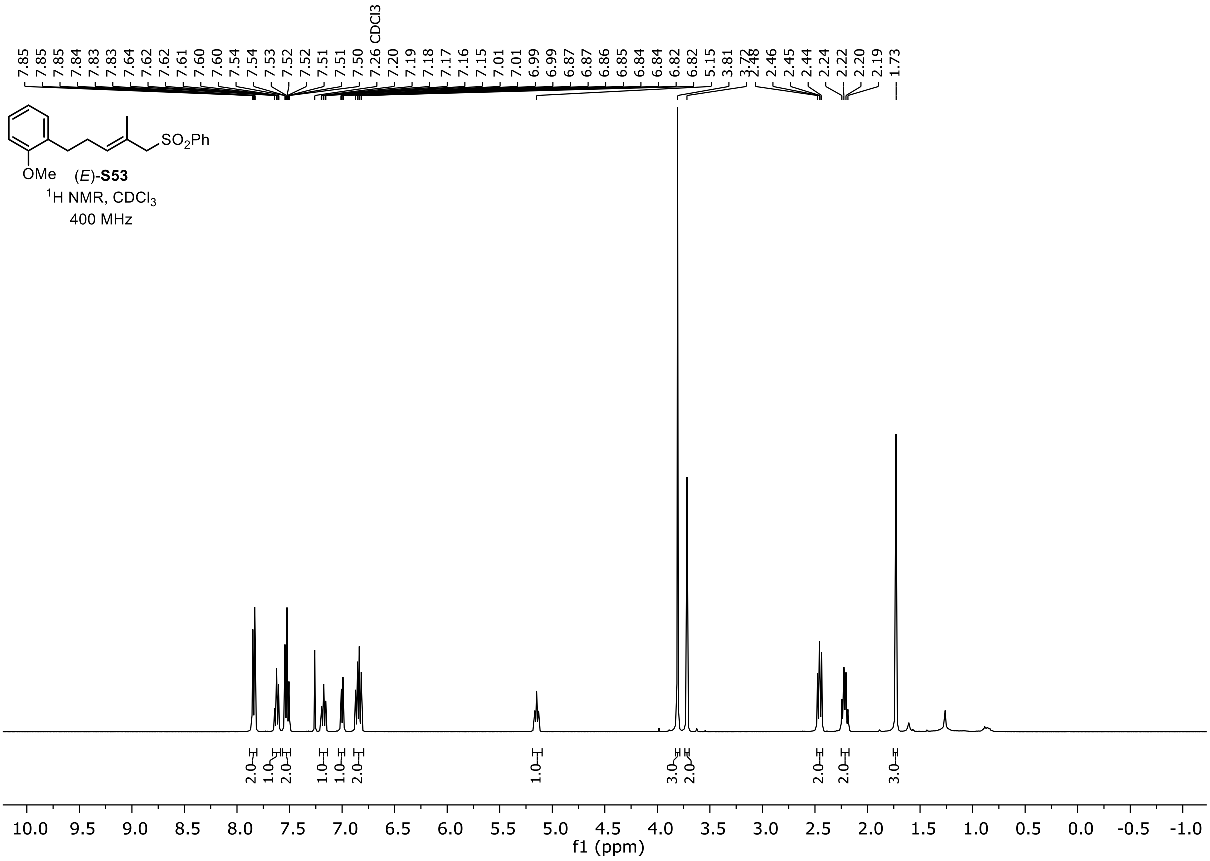

Figure 178. ^{1}H-NMR (400 MHz, $CDCl_3$) of alkene (*E*)-**S53**.

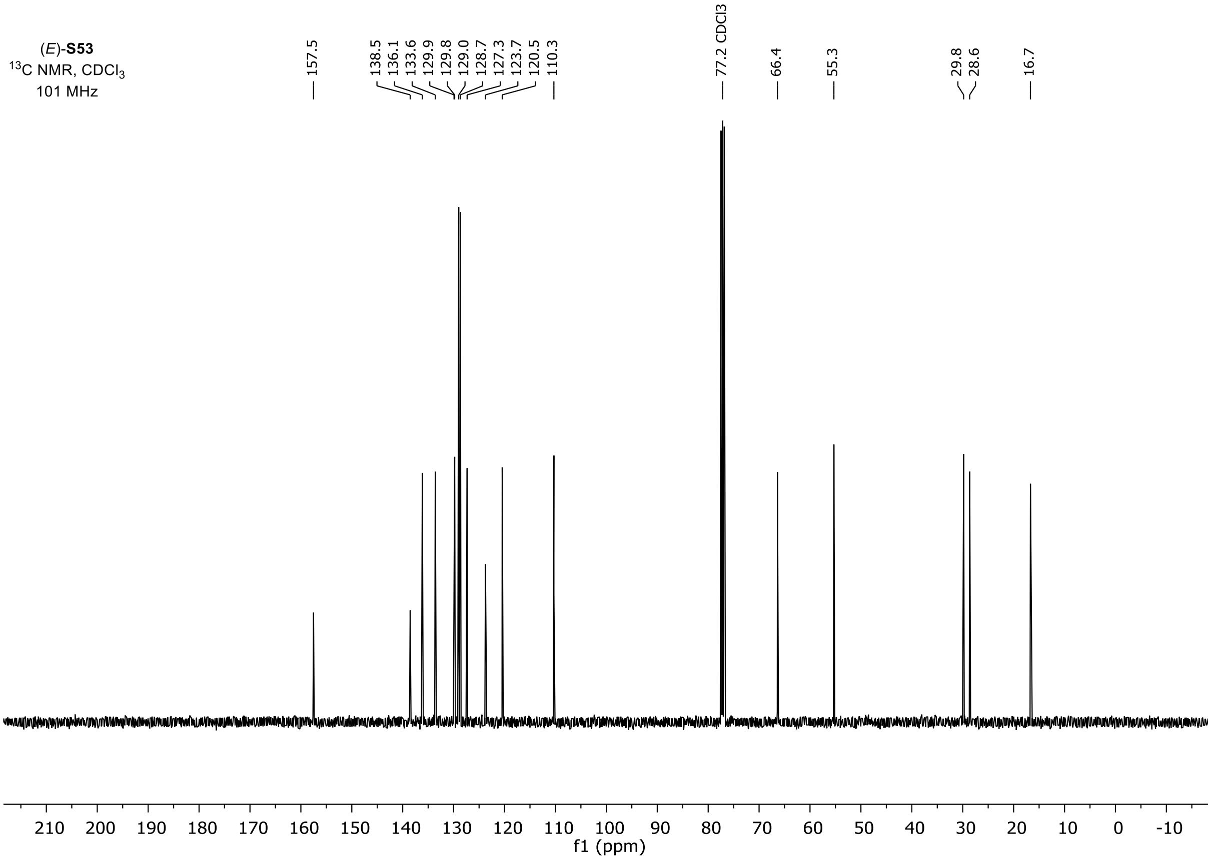

Figure 179. ^{13}C-NMR (101 MHz, $CDCl_3$) of alkene (*E*)-**S53**.

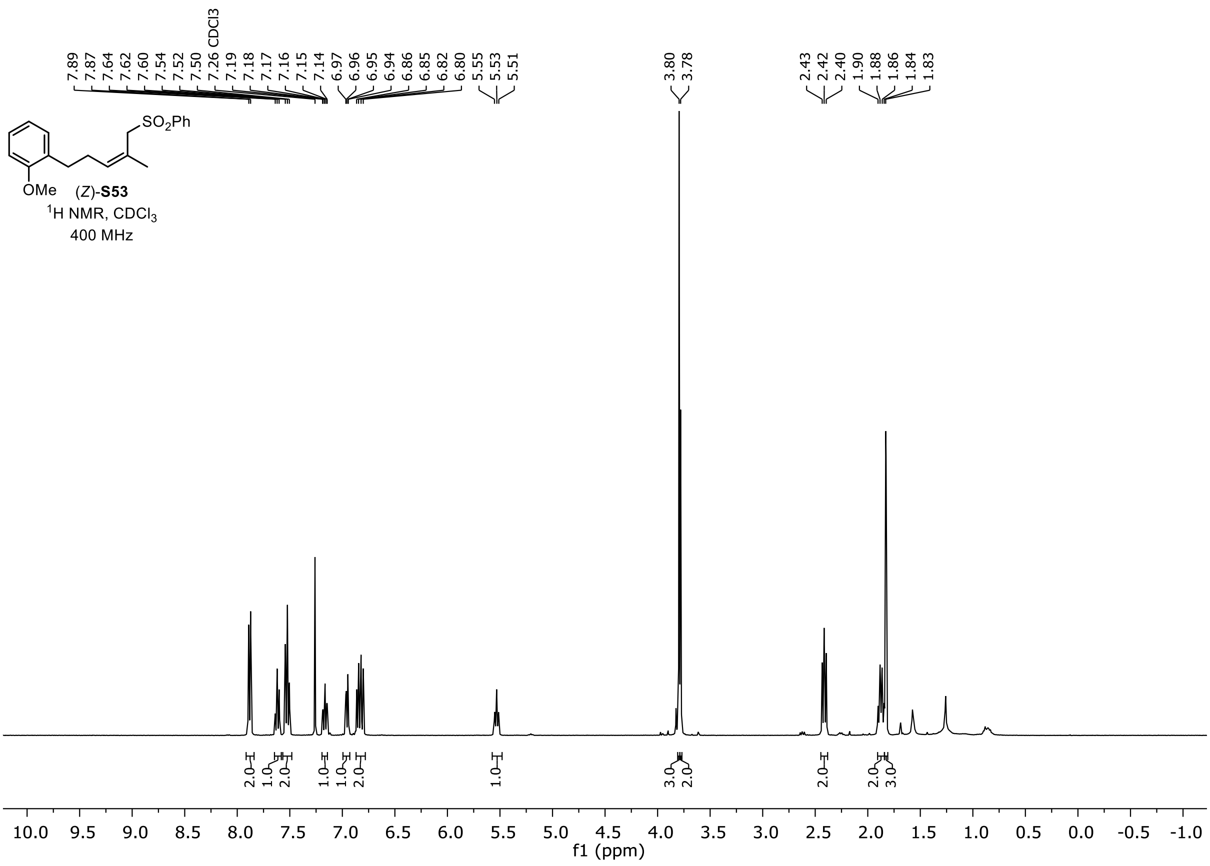

Figure 180. ^{1}H-NMR (400 MHz, $CDCl_3$) of alkene (*Z*)-**S53**.

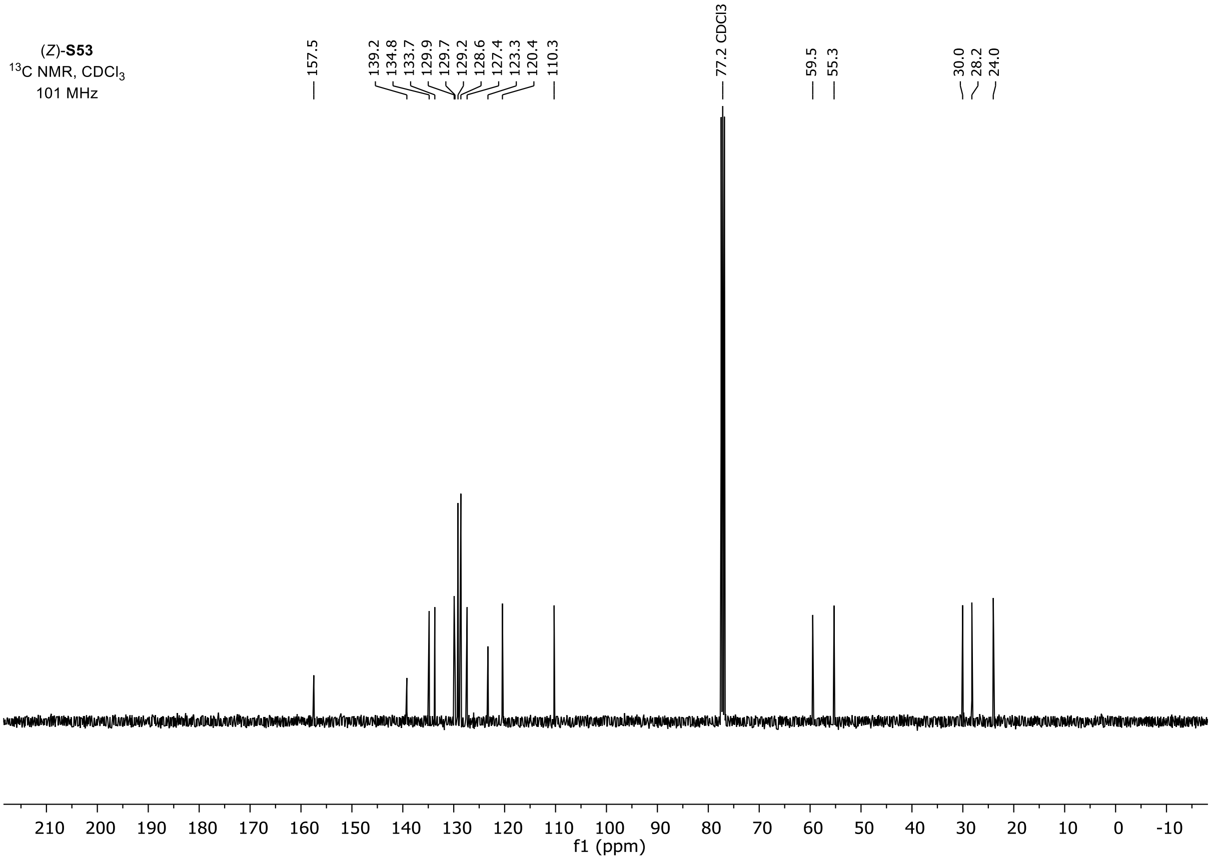

Figure 181. ^{13}C-NMR (101 MHz, $CDCl_3$) of alkene (*Z*)-**S53**.

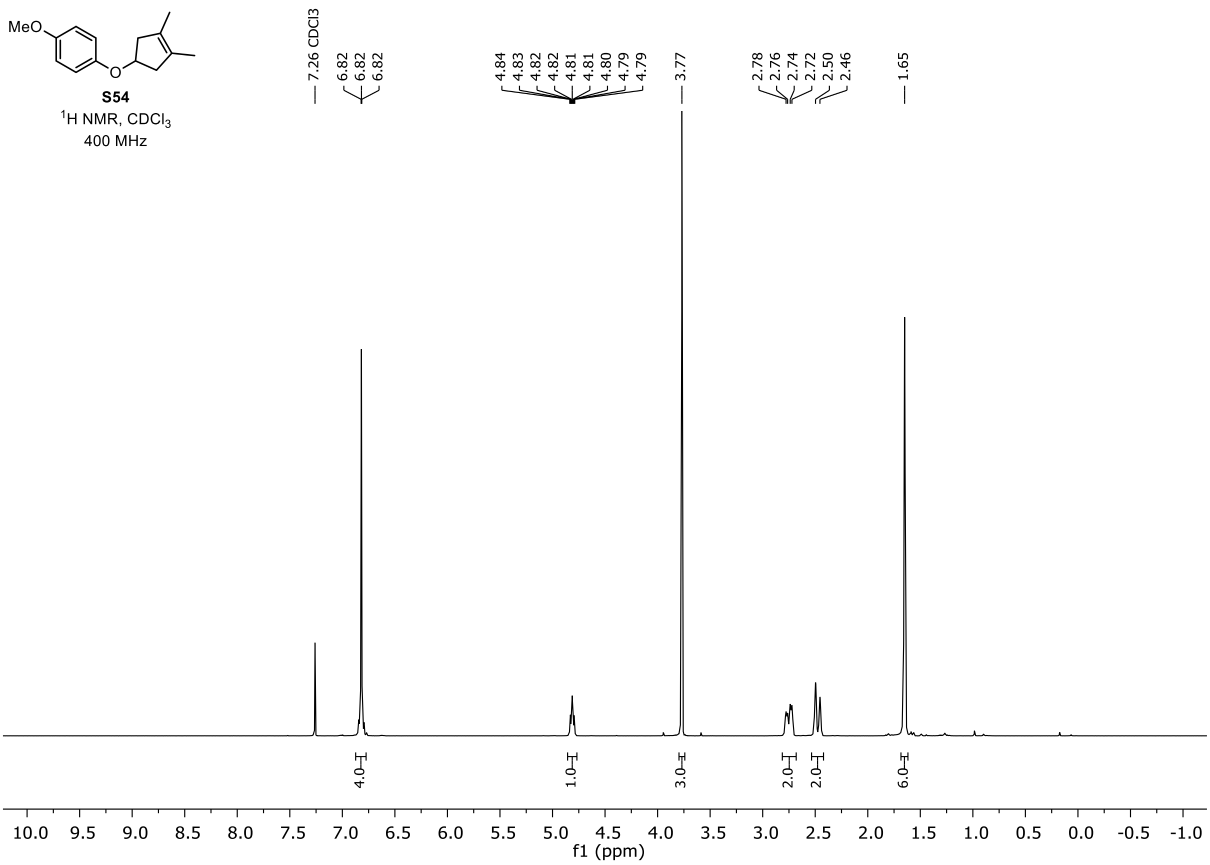

Figure 182. ^{1}H-NMR (400 MHz, $CDCl_3$) of cyclopentene ether **S54**.

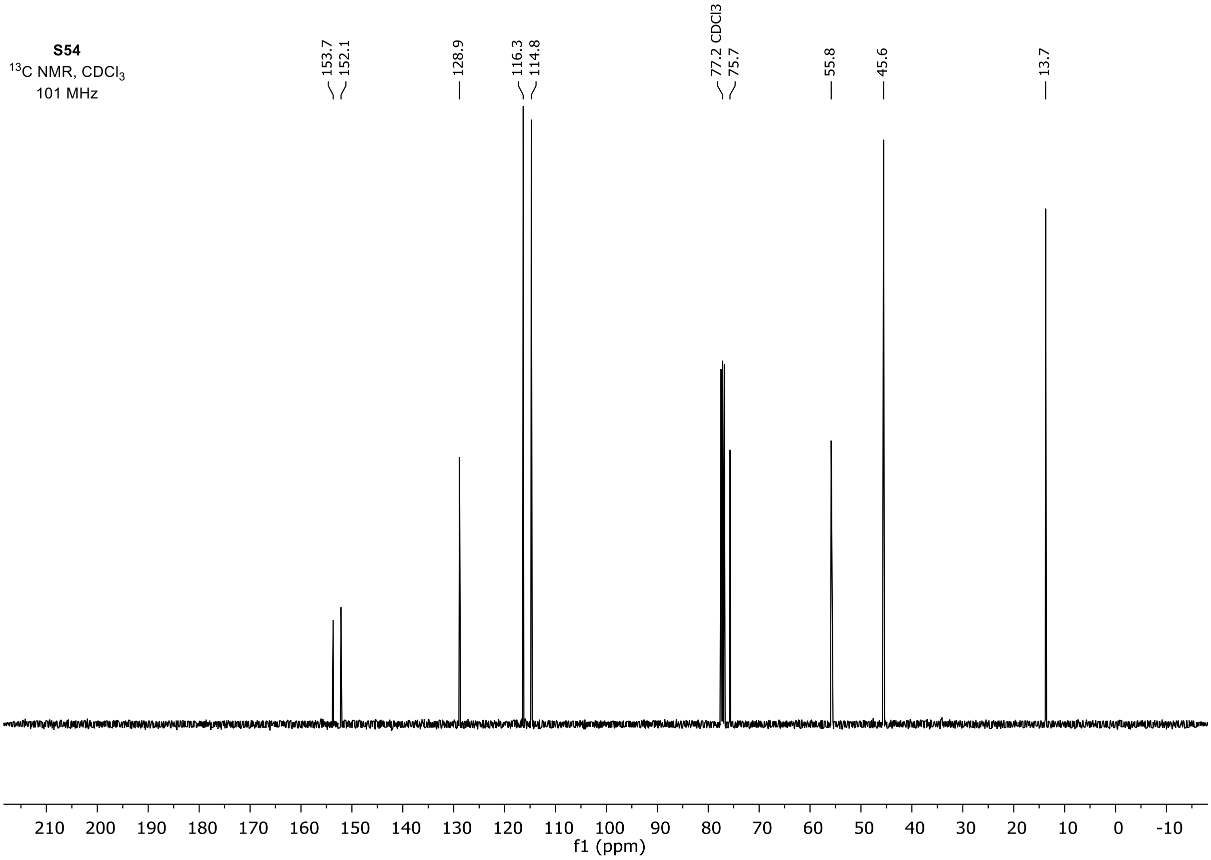

Figure 183. ^{13}C-NMR (101 MHz, $CDCl_3$) of cyclopentene ether **S54**.

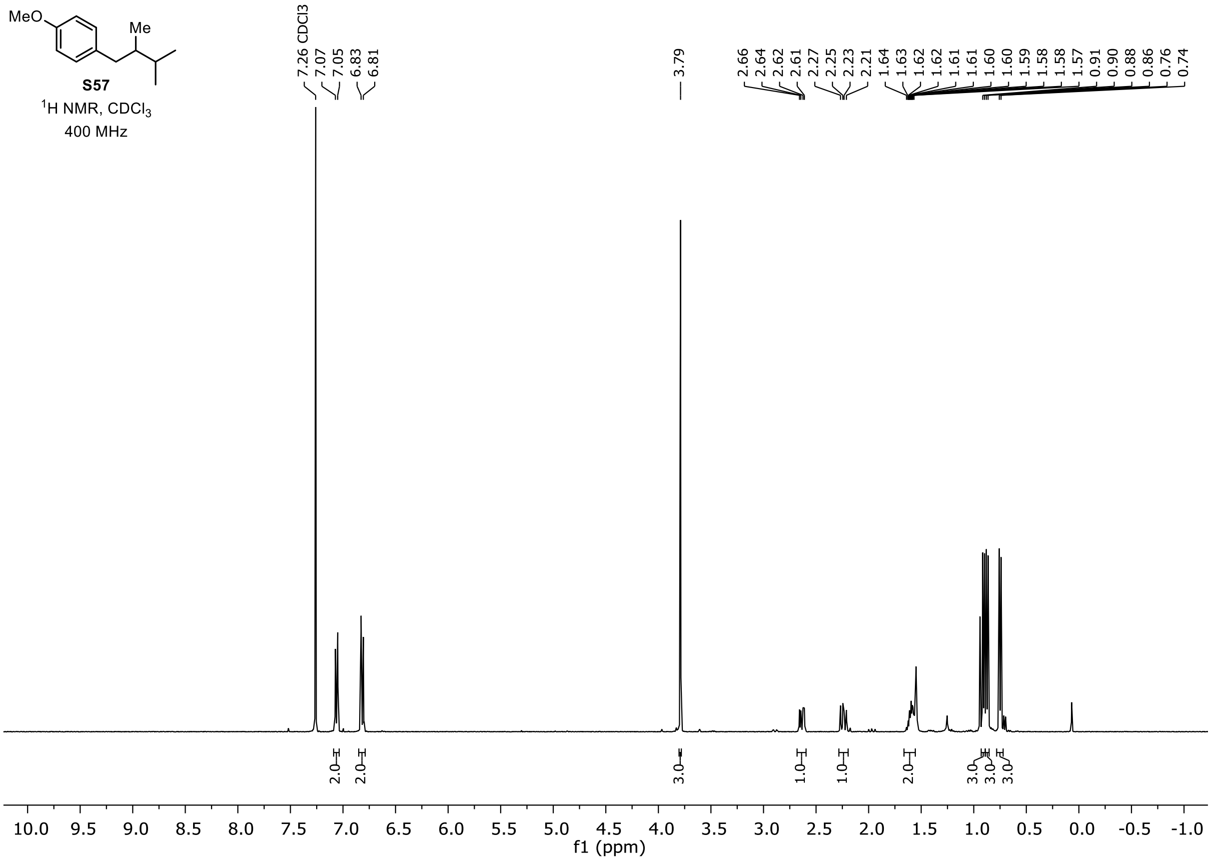

Figure 184. ^{1}H-NMR (400 MHz, $CDCl_3$) of arene **S57**.

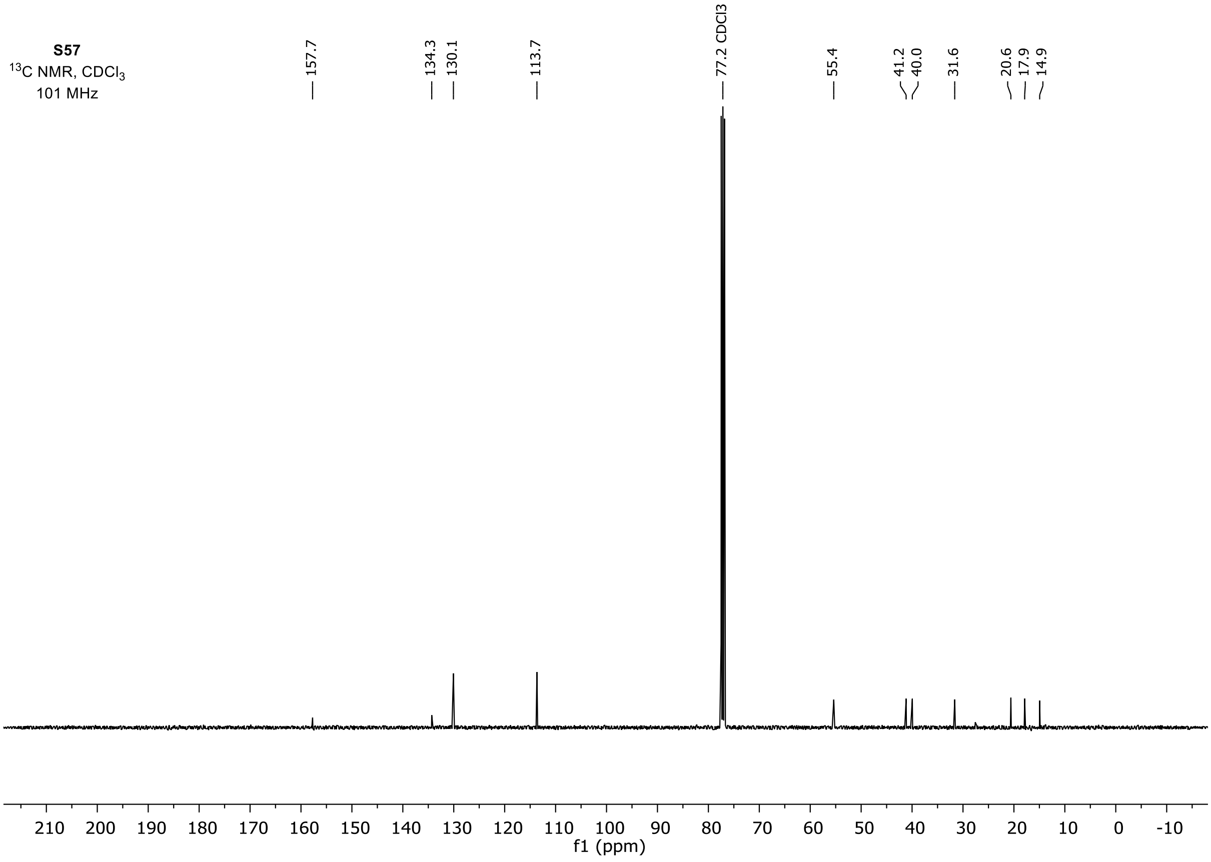

Figure 185. ^{13}C-NMR (101 MHz, $CDCl_3$) of arene **S57**.

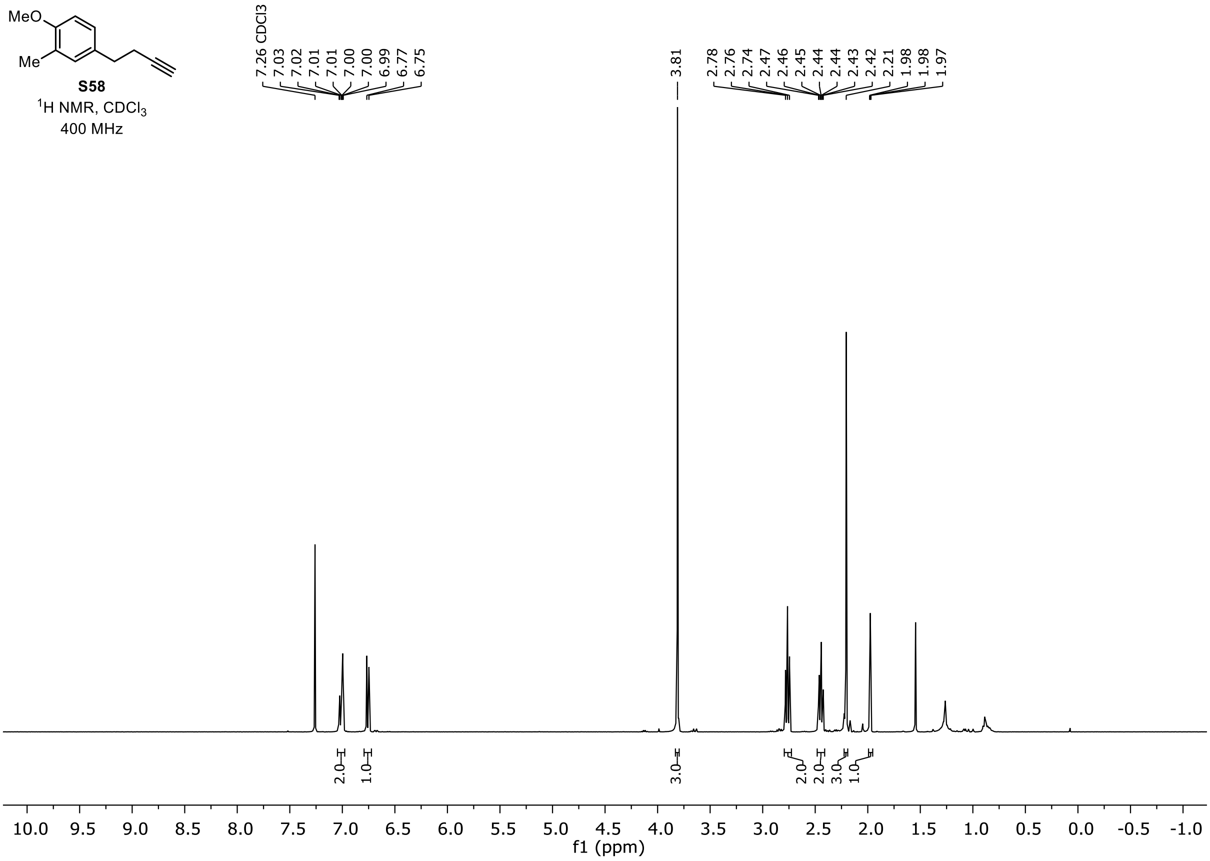

Figure 186. ^{1}H-NMR (400 MHz, $CDCl_3$) of alkyne **S58**.

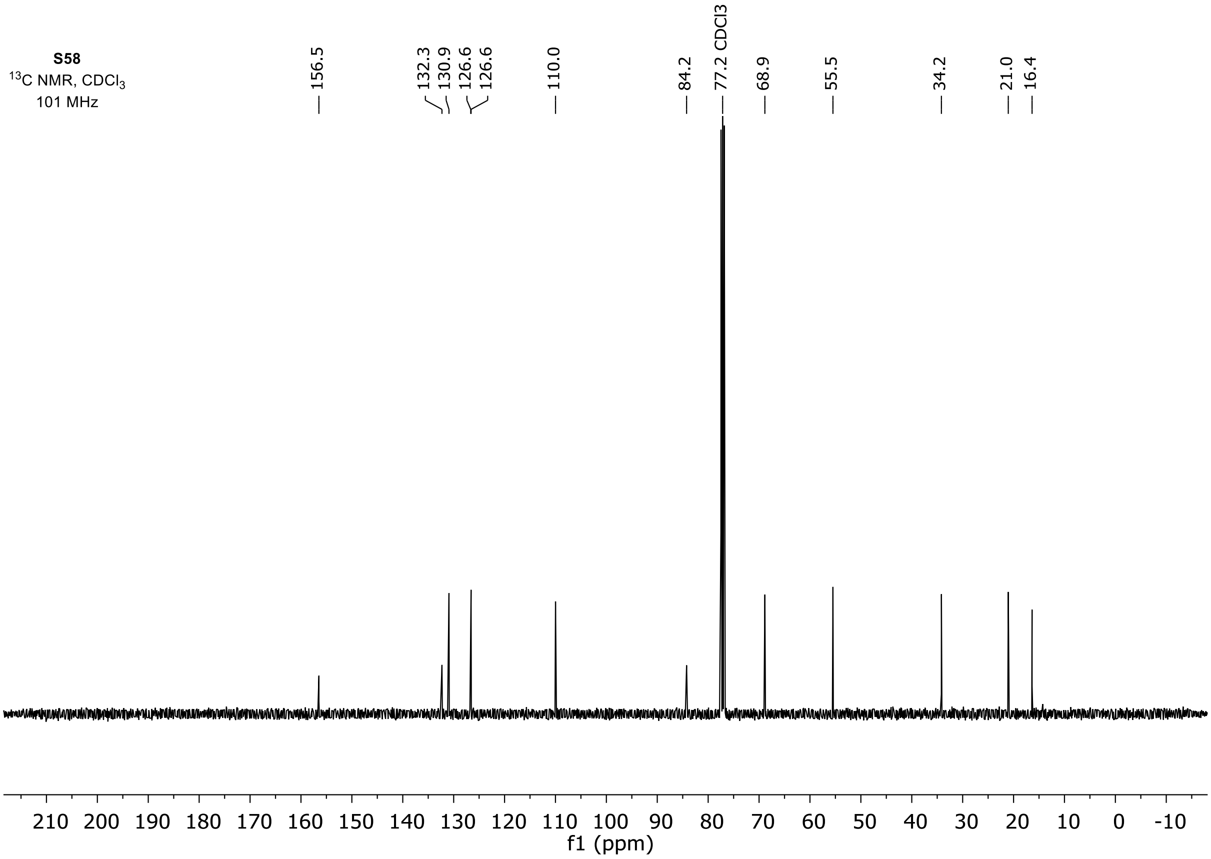

Figure 187. ^{13}C-NMR (101 MHz, $CDCl_3$) of alkyne **S58**.

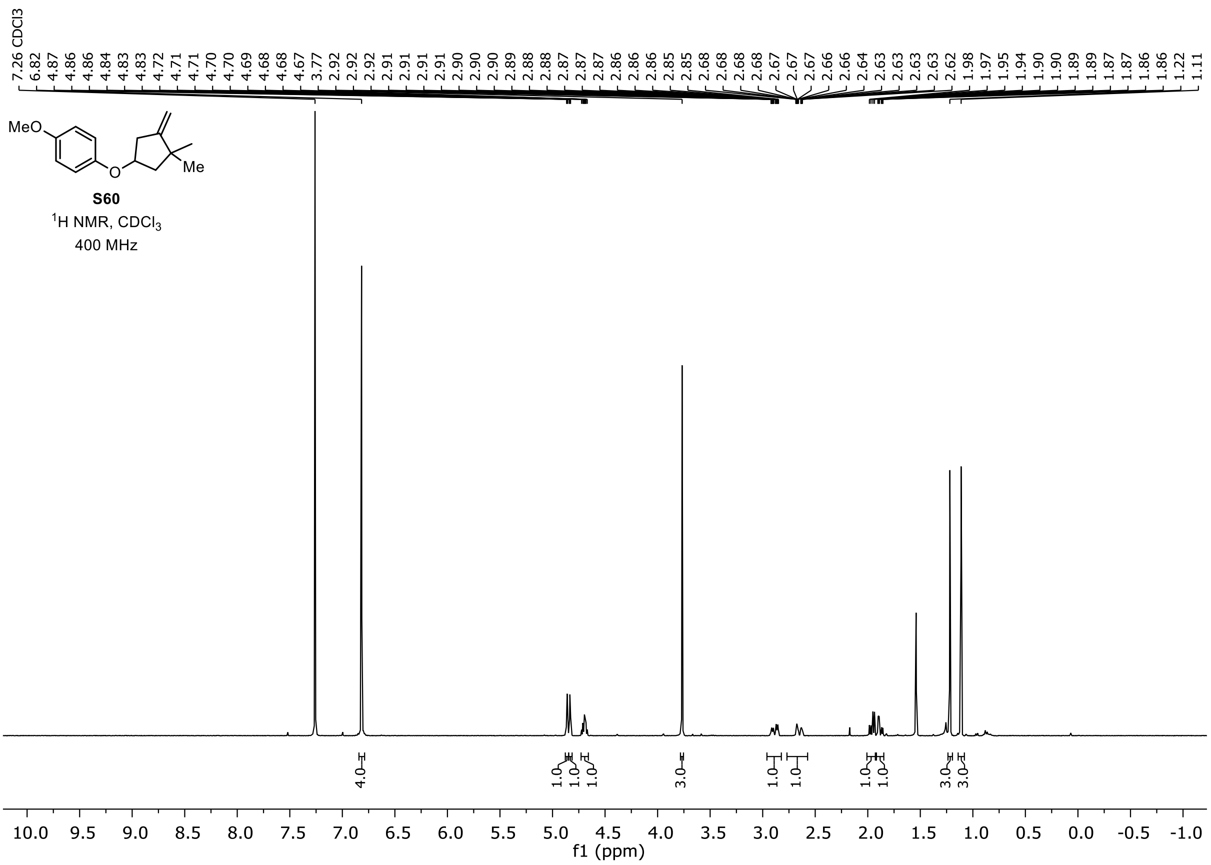

Figure 188. ^{1}H-NMR (400 MHz, $CDCl_3$) of cyclopentene **S60**.

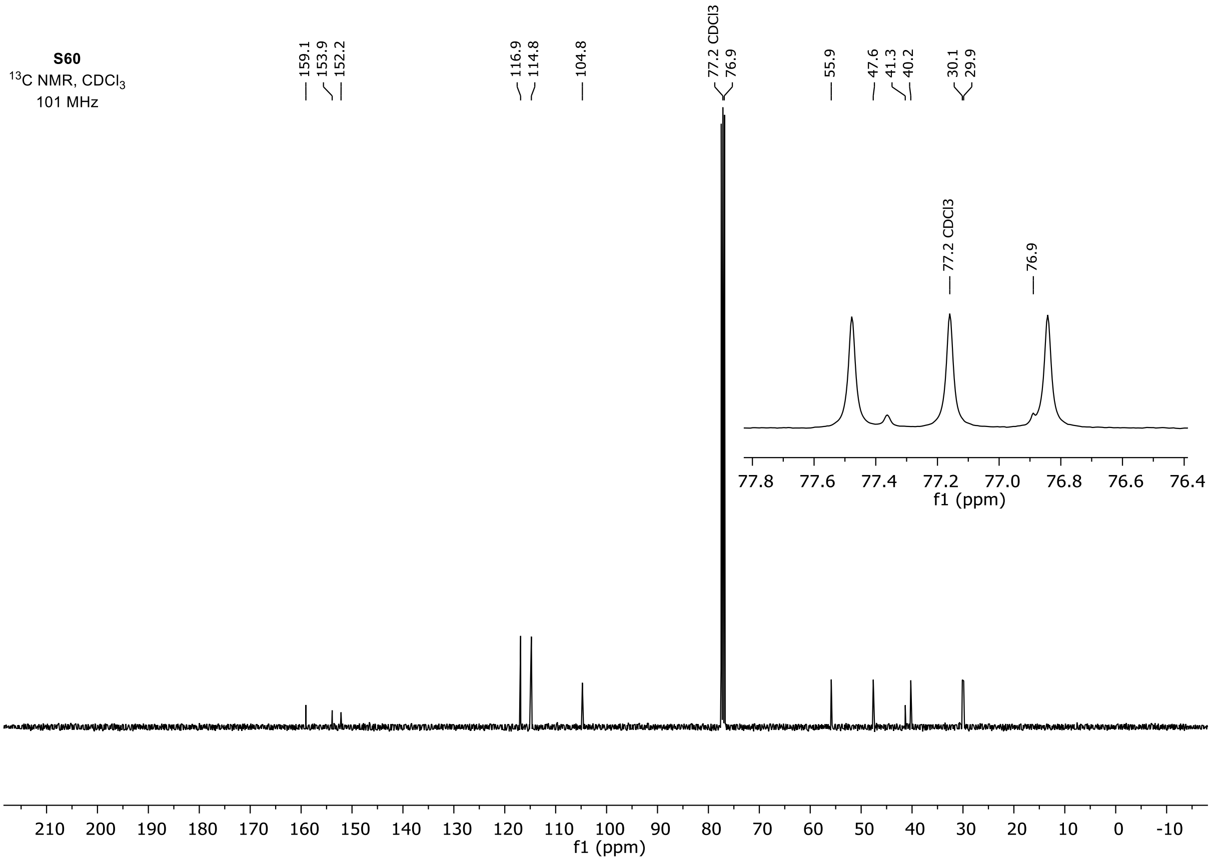

Figure 189. ^{13}C-NMR (101 MHz, $CDCl_3$) of cyclopentene **S60**.

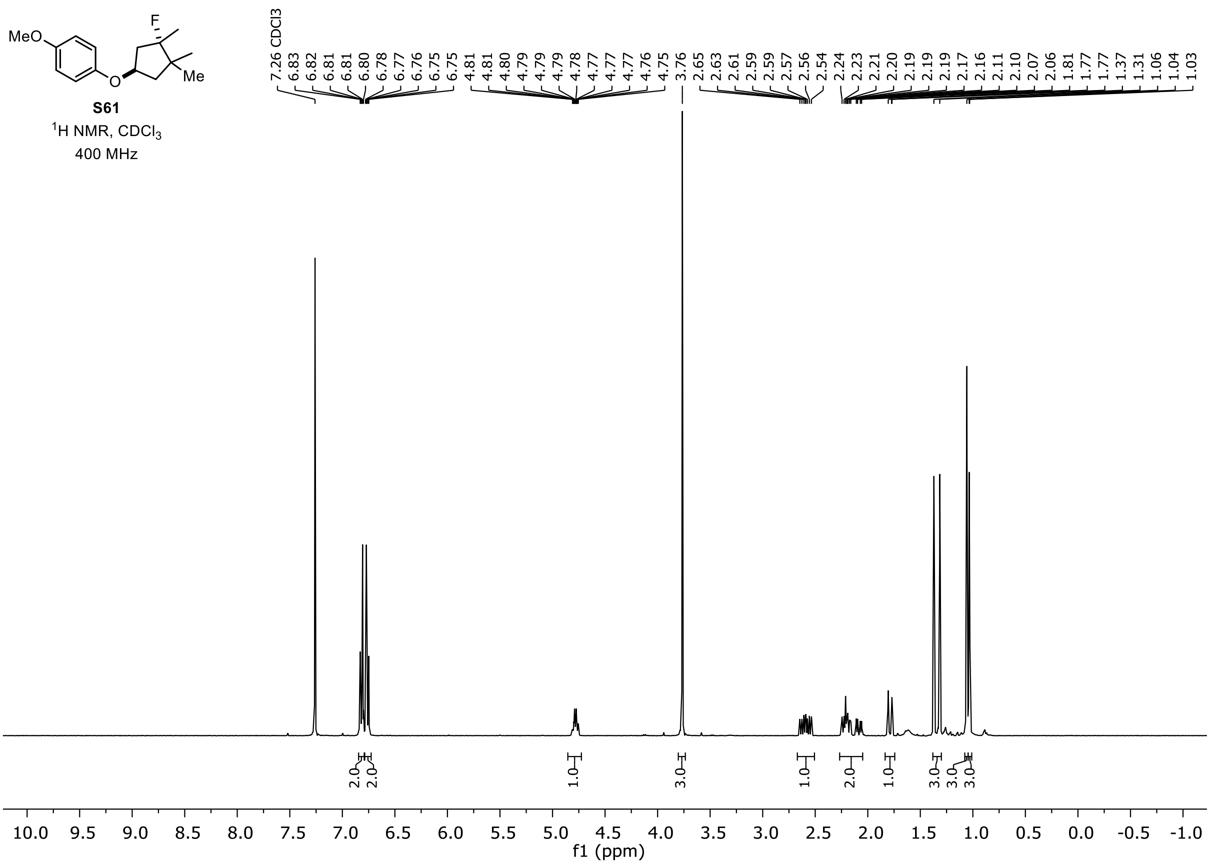

Figure 190. ^{1}H-NMR (400 MHz, $CDCl_3$) of fluoride **S61**.

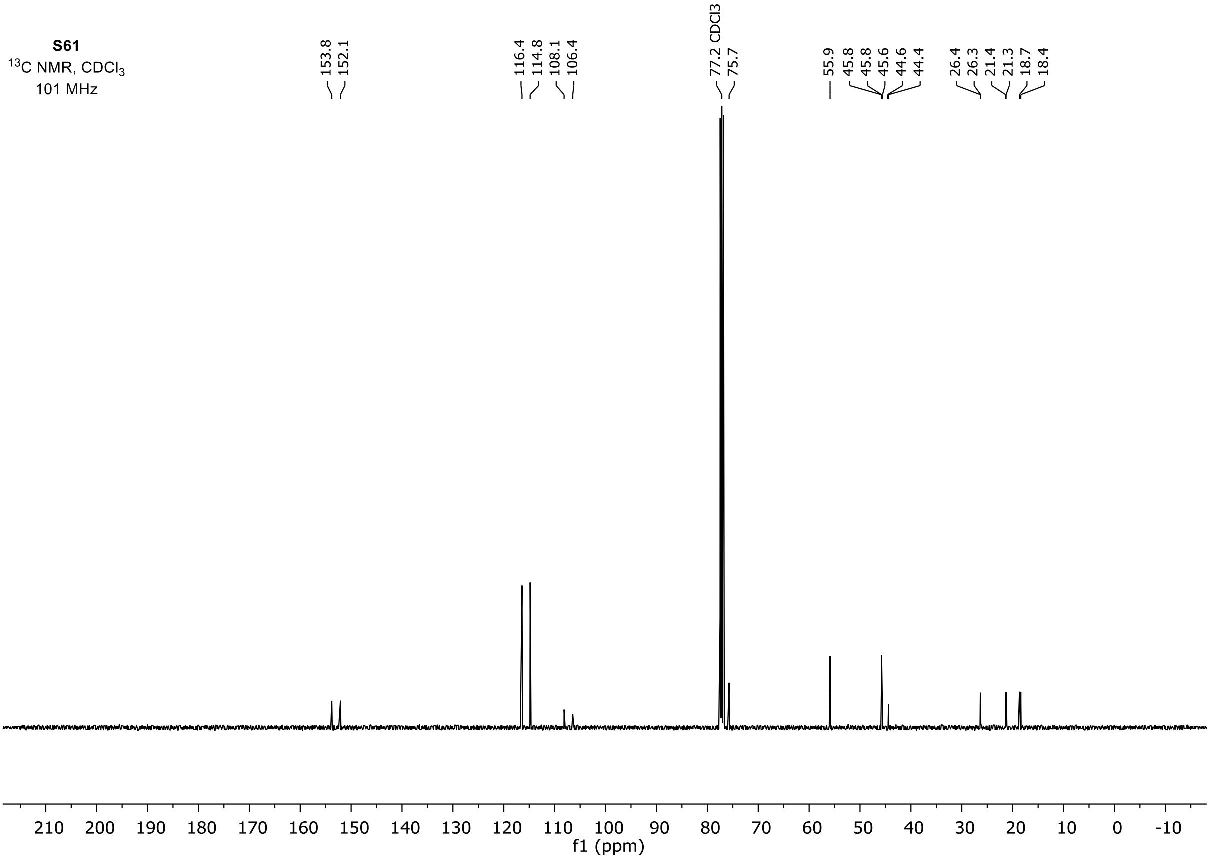

Figure 191. ^{13}C-NMR (101 MHz, $CDCl_3$) of fluoride **S61**.

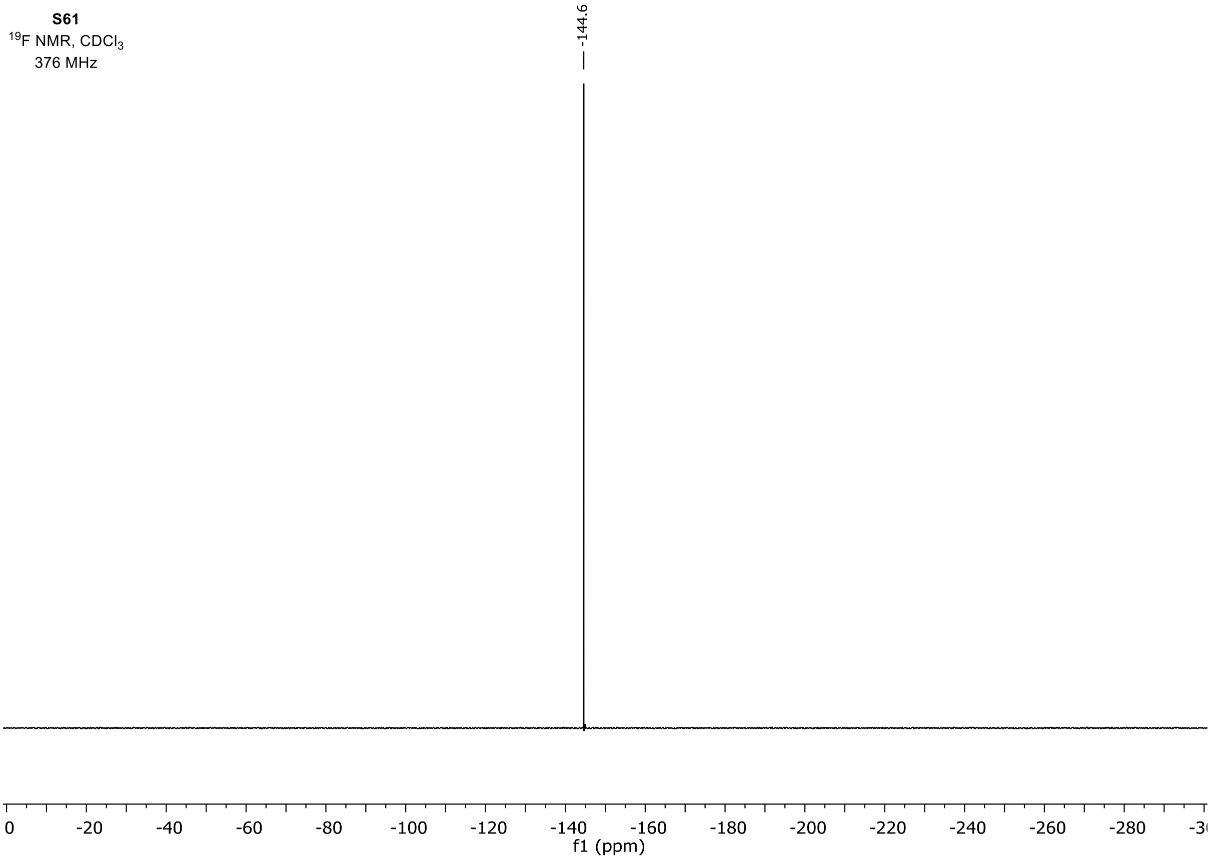

Figure 192. ^{19}F-NMR (376 MHz, $CDCl_3$) of fluoride **S61**.

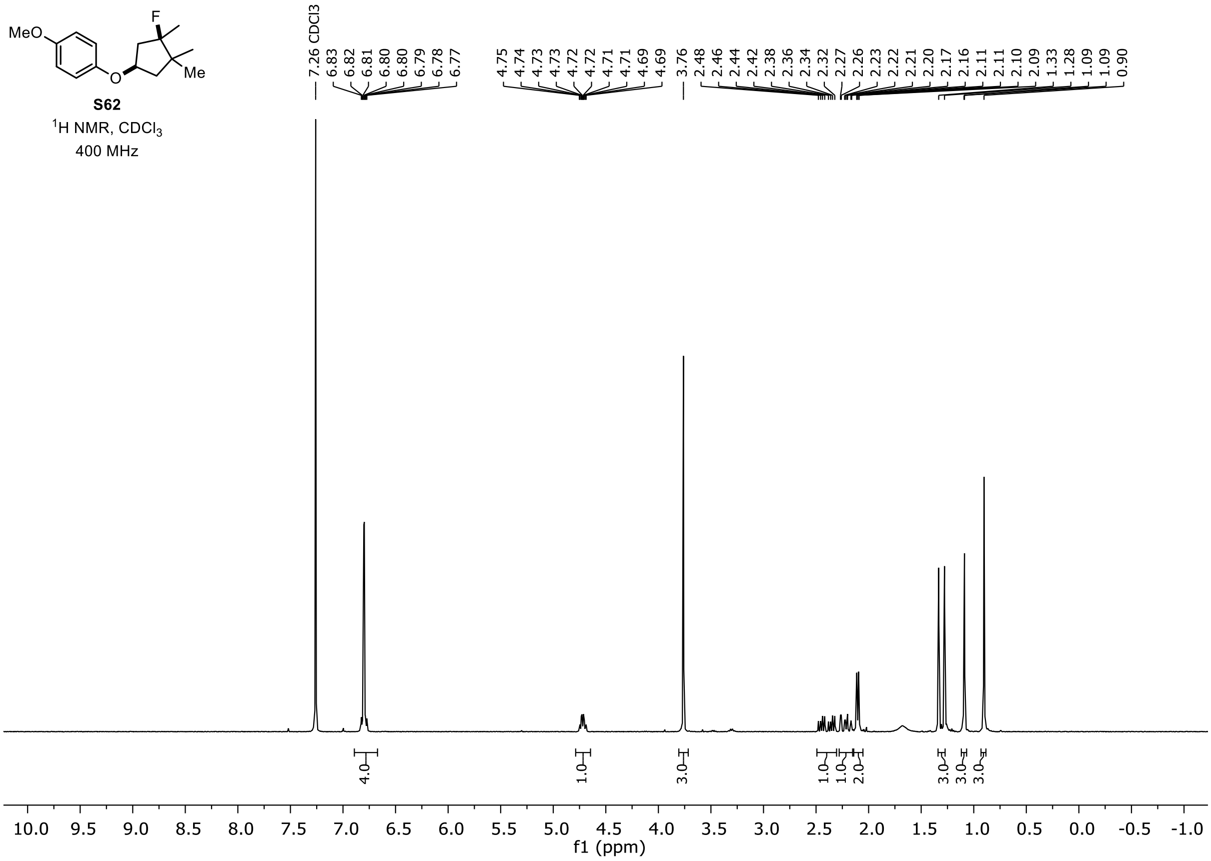

Figure 193. ^{1}H-NMR (400 MHz, $CDCl_3$) of fluoride **S62**.

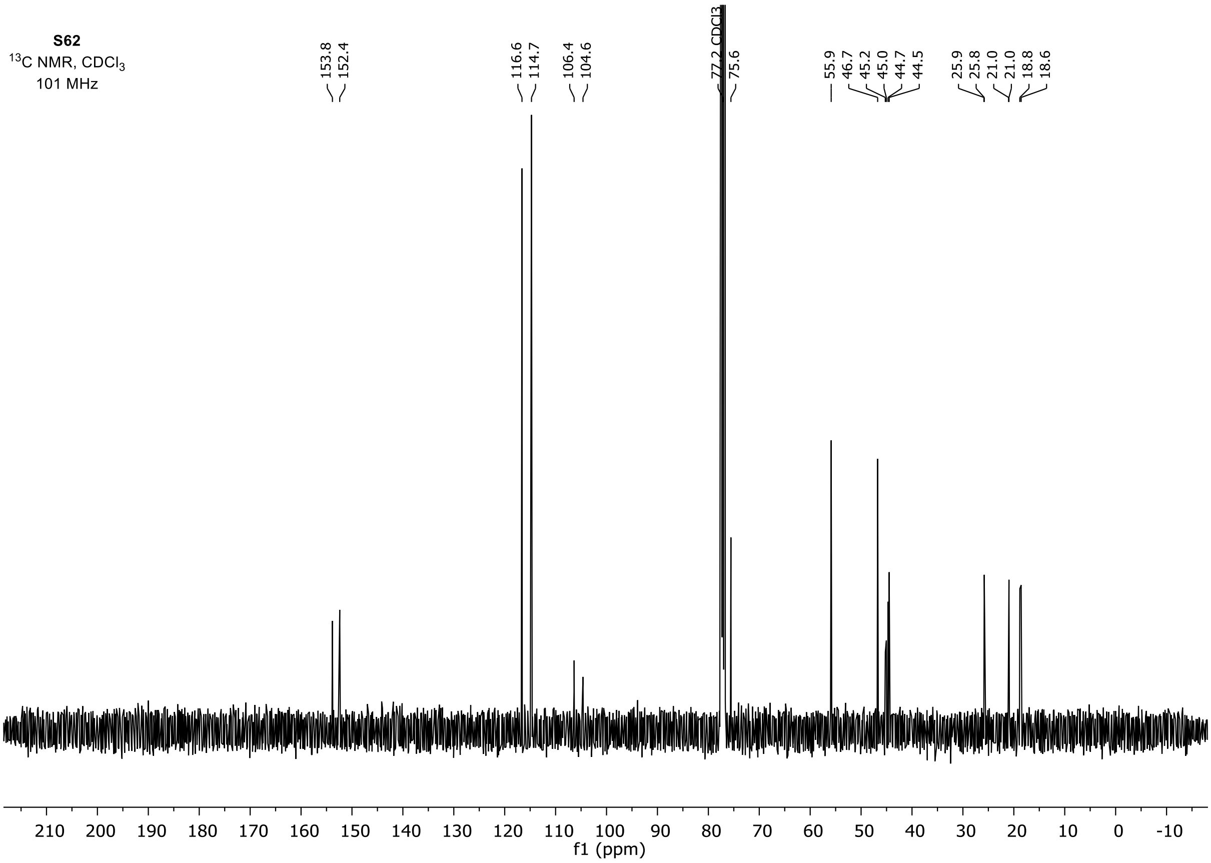

Figure 194. ^{13}C-NMR (101 MHz, $CDCl_3$) of fluoride **S62**.

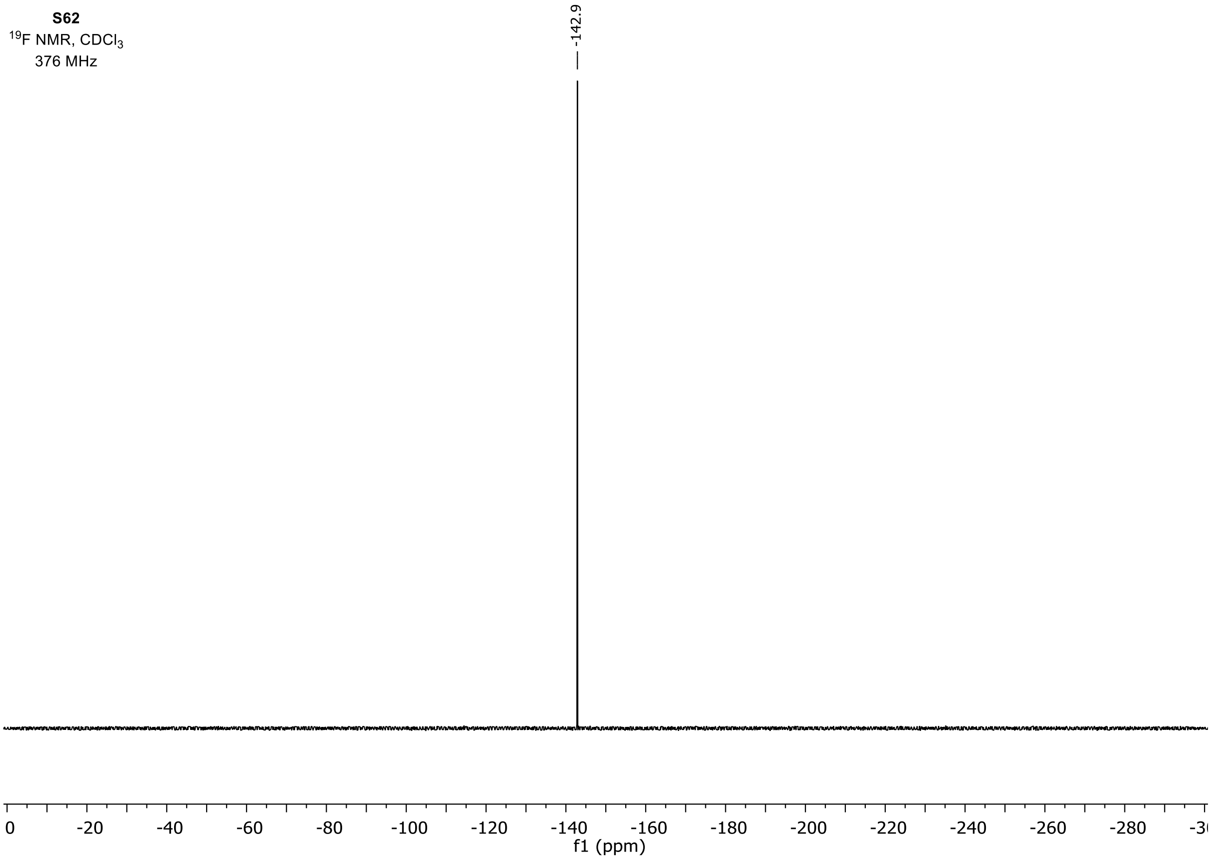

Figure 195. ^{19}F-NMR (376 MHz, $CDCl_3$) of fluoride **S62**.

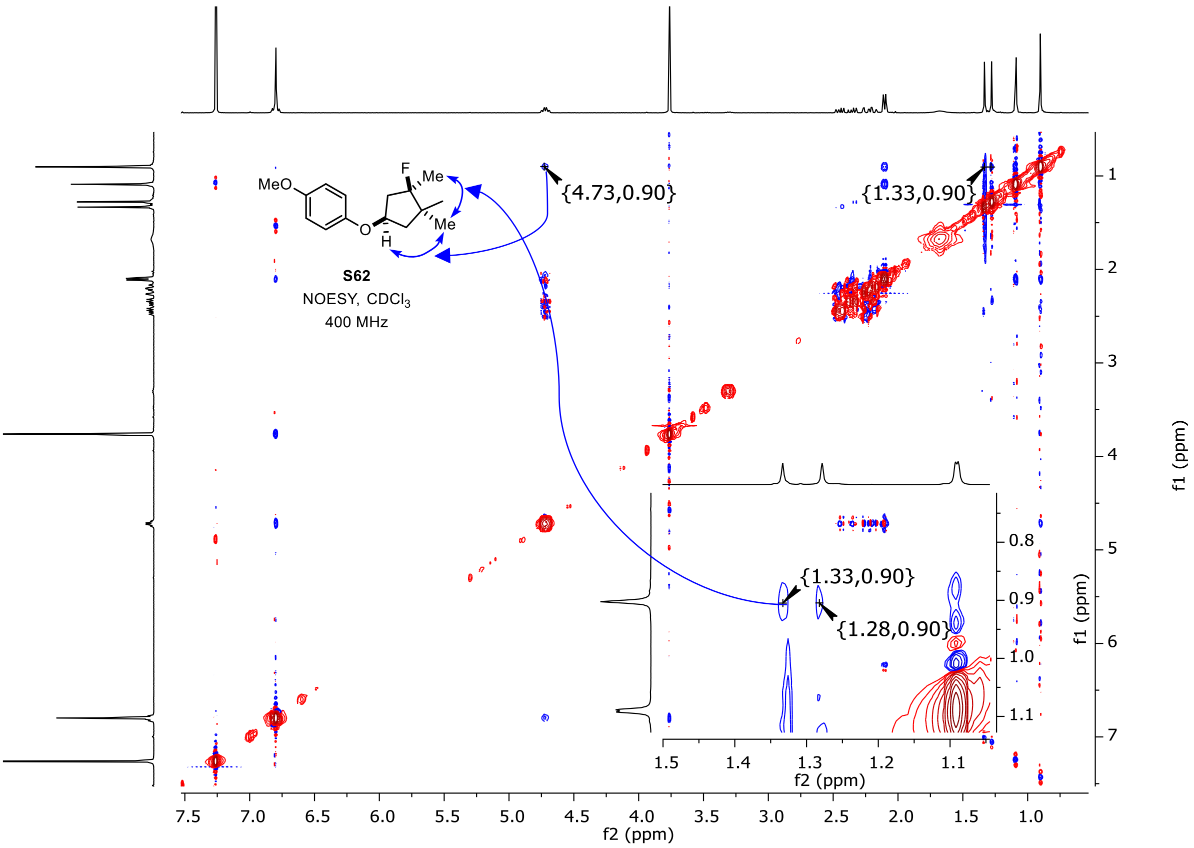

Figure 196. NOESY (400 MHz, $CDCl_3$) of fluoride **S62**.

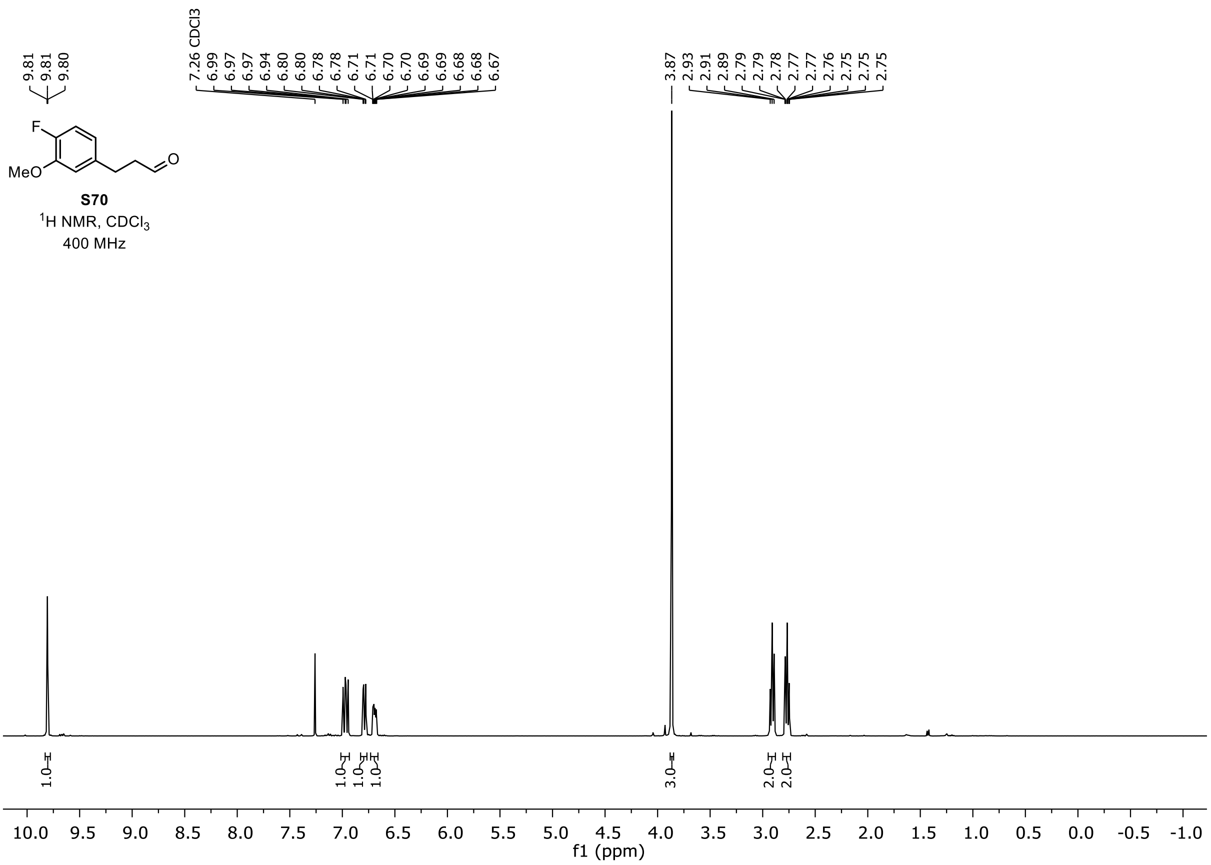

Figure 197. ^{1}H-NMR (400 MHz, $CDCl_3$) of aldehyde **S70**.

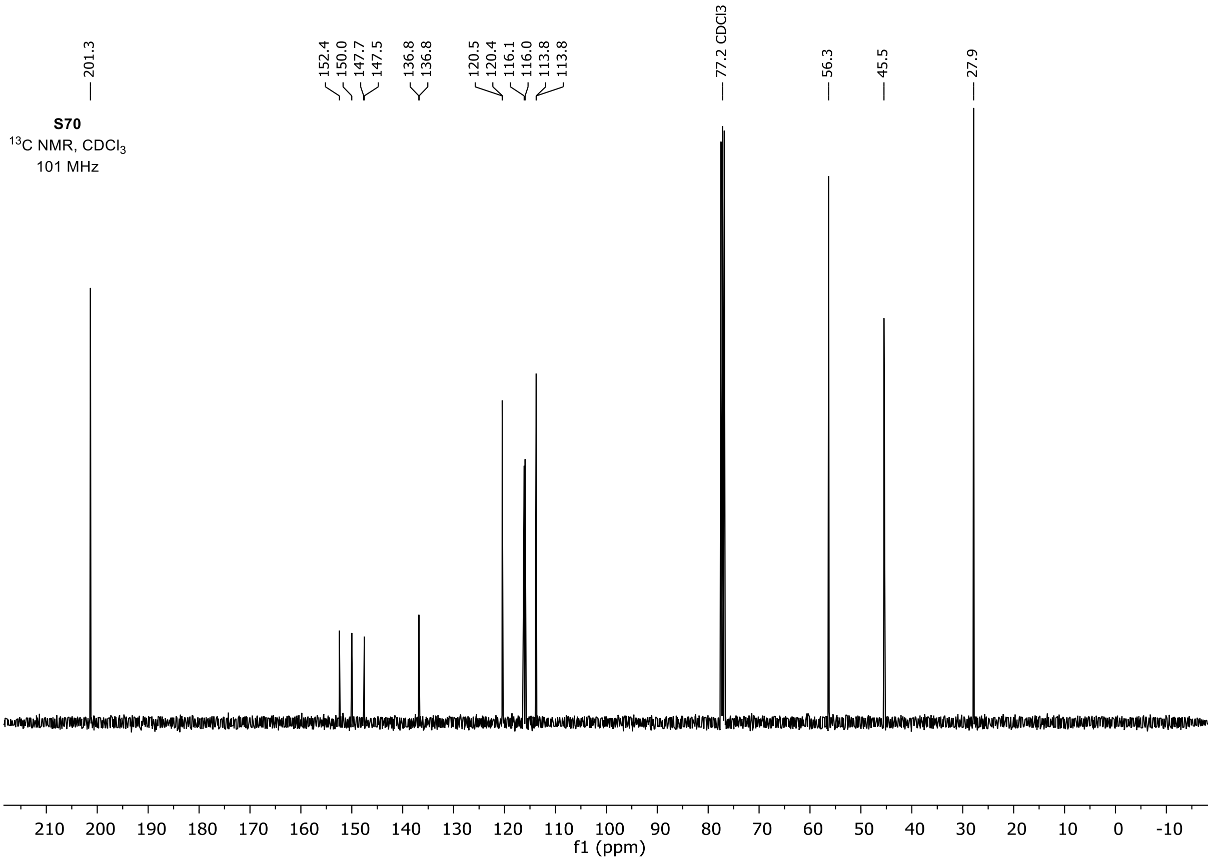

Figure 198. ^{13}C-NMR (101 MHz, $CDCl_3$) of aldehyde **S70**.

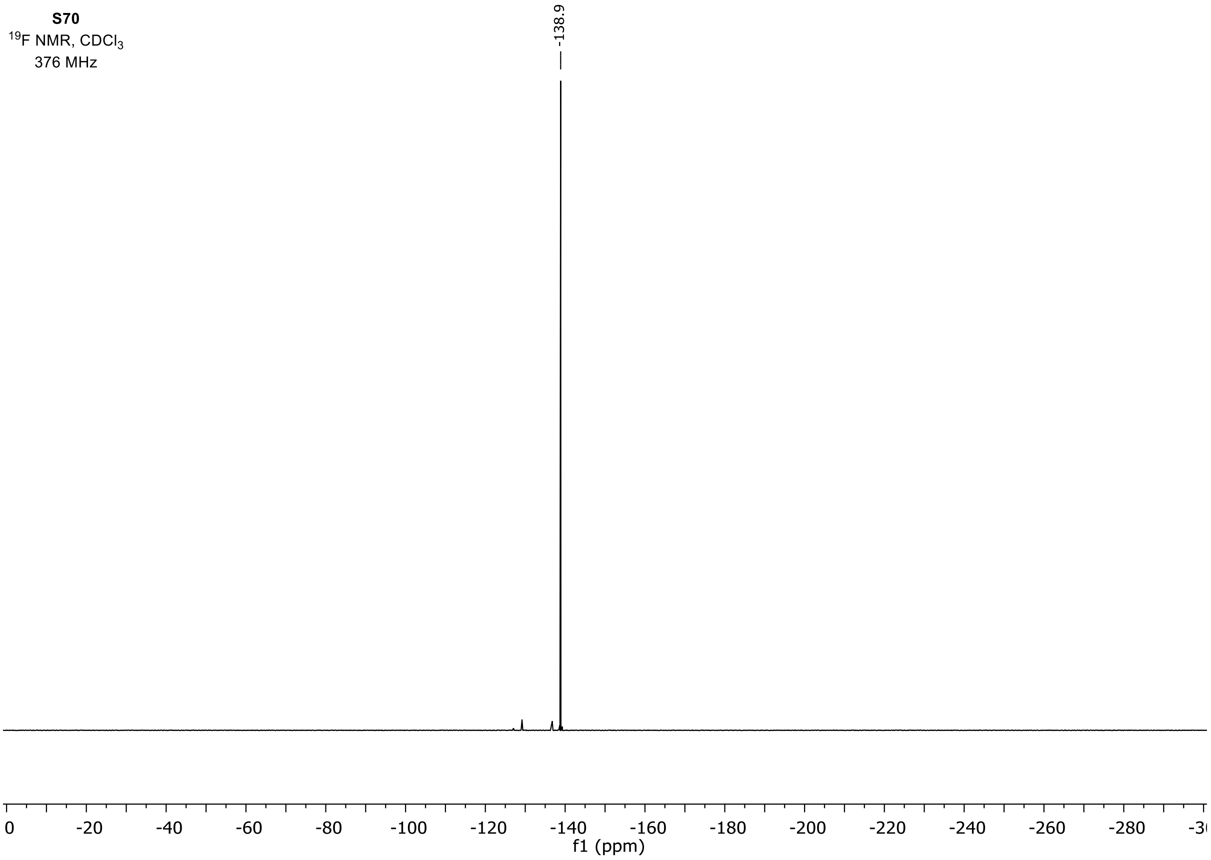

Figure 199. ^{19}F-NMR (376 MHz, $CDCl_3$) of aldehyde **S70**.

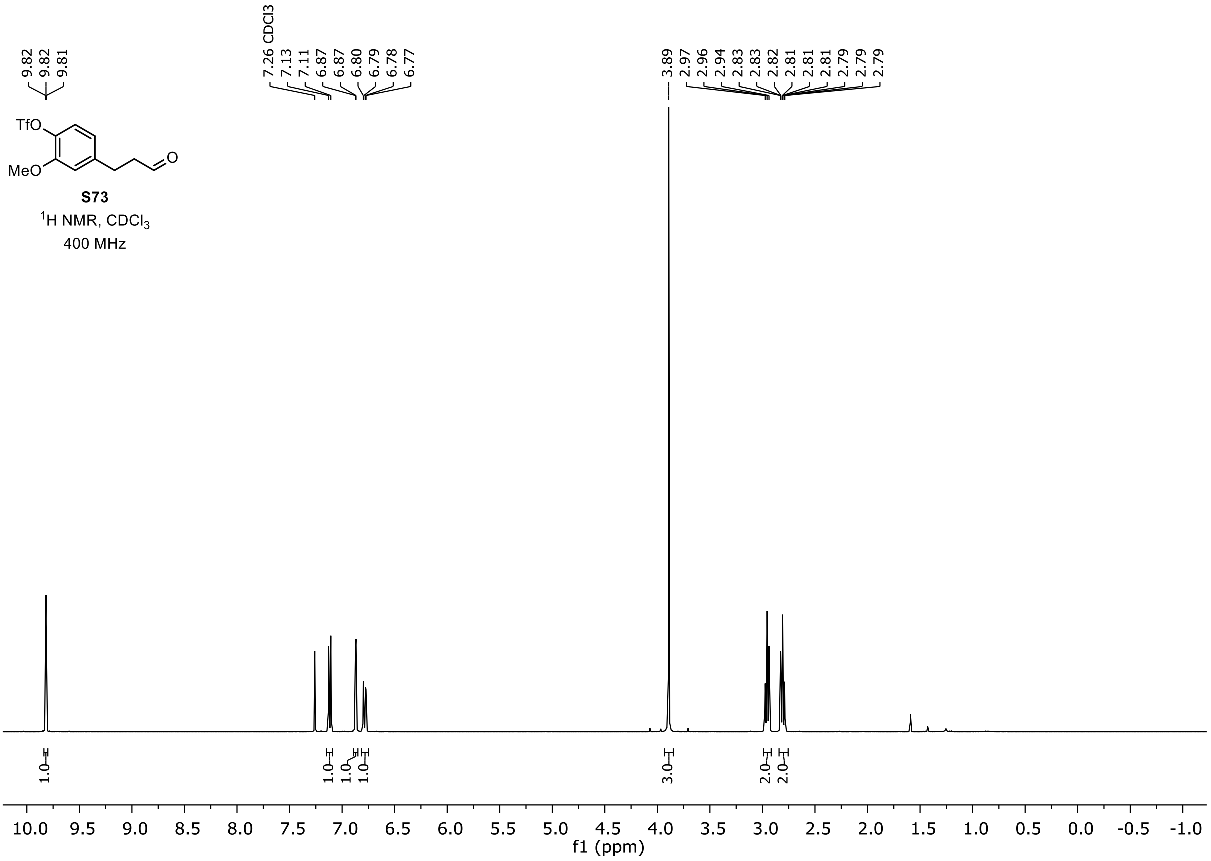

Figure 200. ^{1}H-NMR (400 MHz, $CDCl_3$) of aldehyde **S73**.

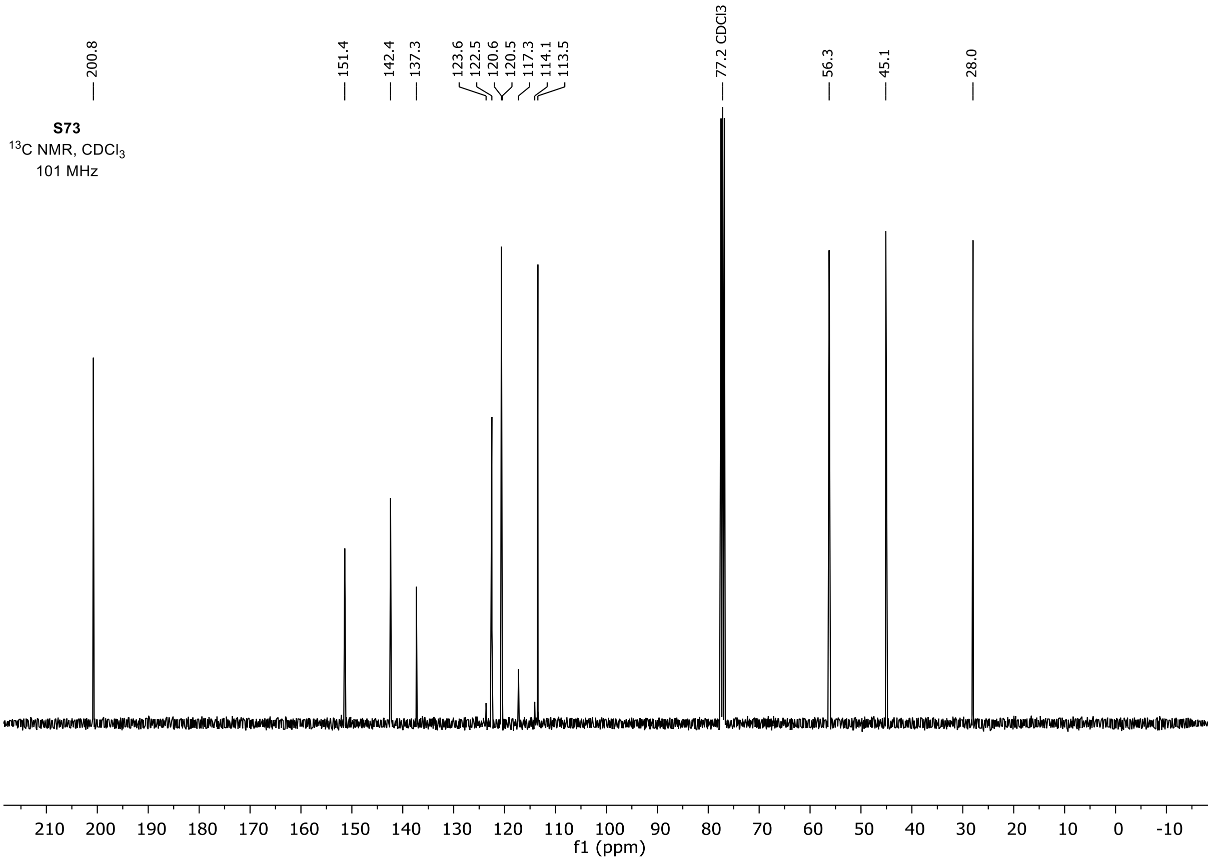

Figure 201. ^{13}C-NMR (101 MHz, $CDCl_3$) of aldehyde **S73**.

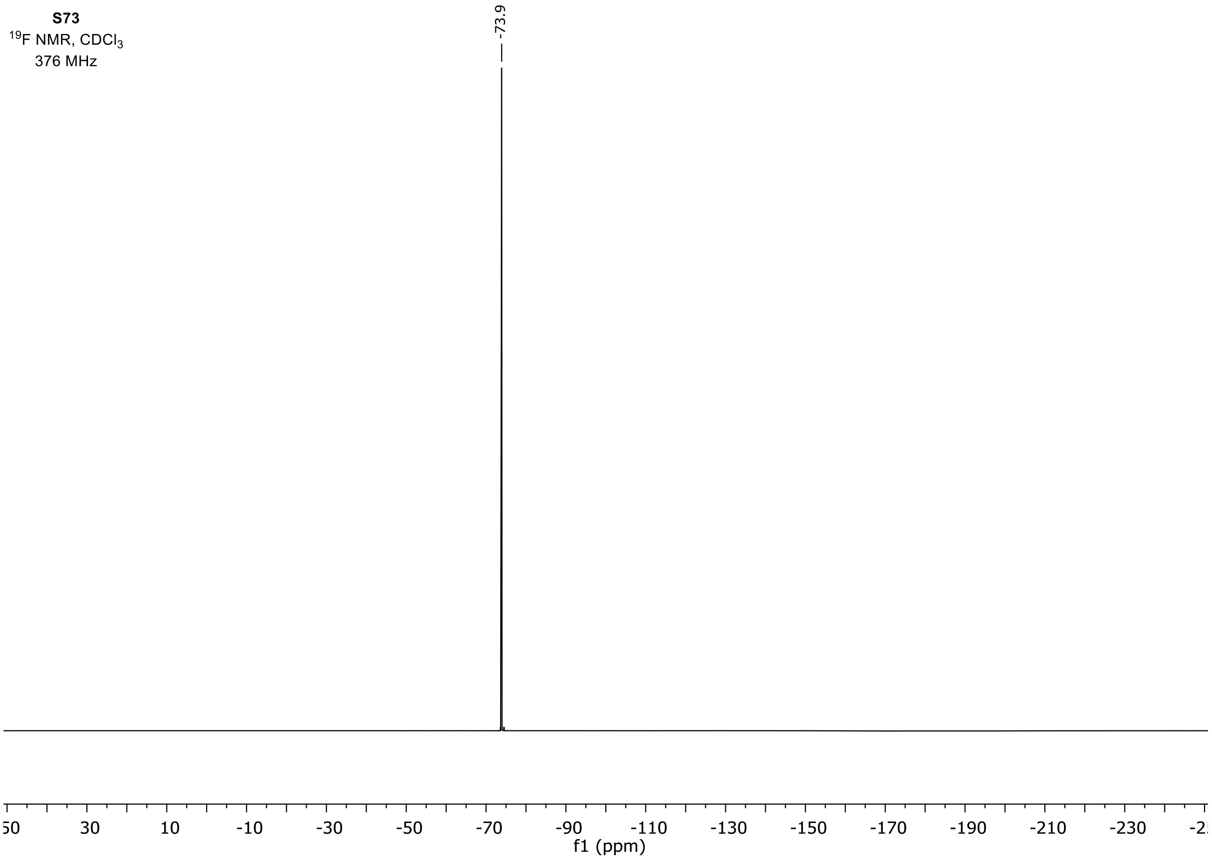

Figure 202. ^{19}F-NMR (376 MHz, $CDCl_3$) of aldehyde **S73**.

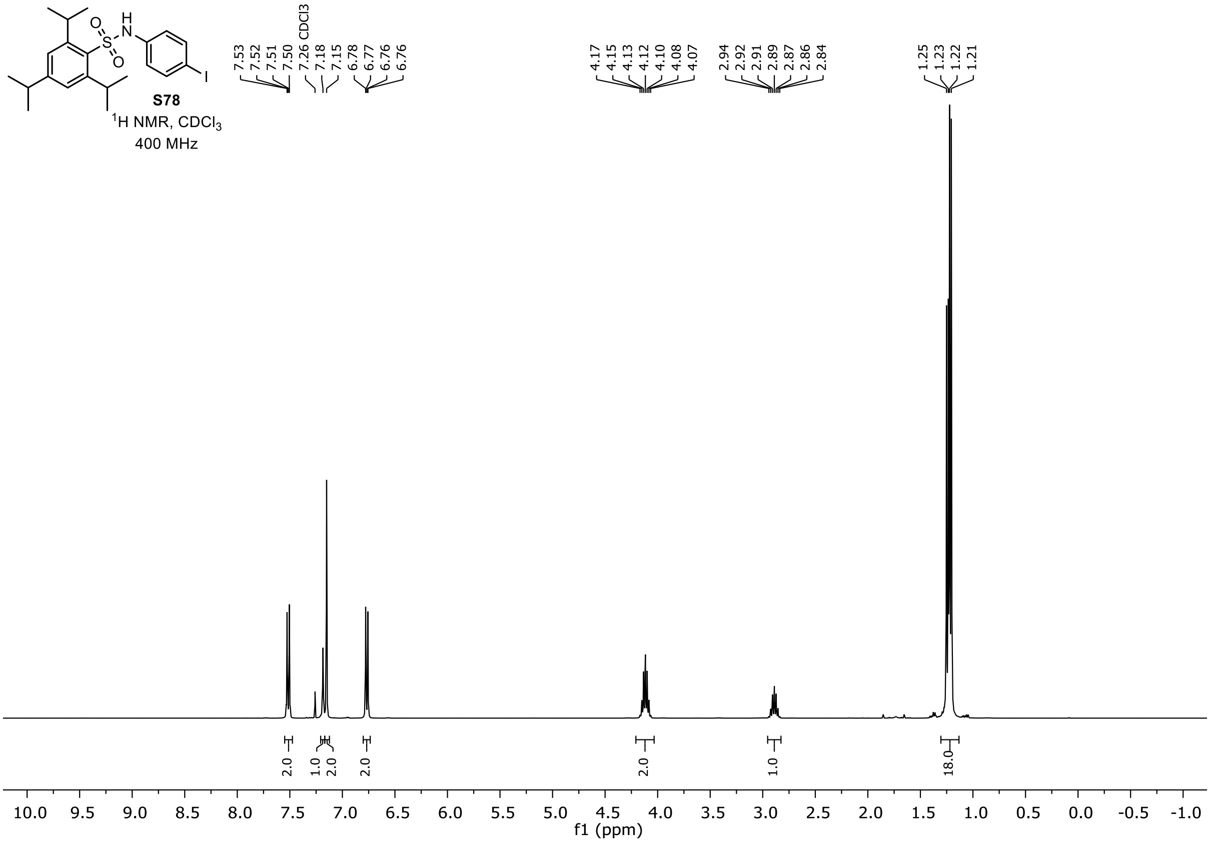

Figure 203. ^{1}H-NMR (400 MHz, $CDCl_3$) of sulfonamide **S78**.

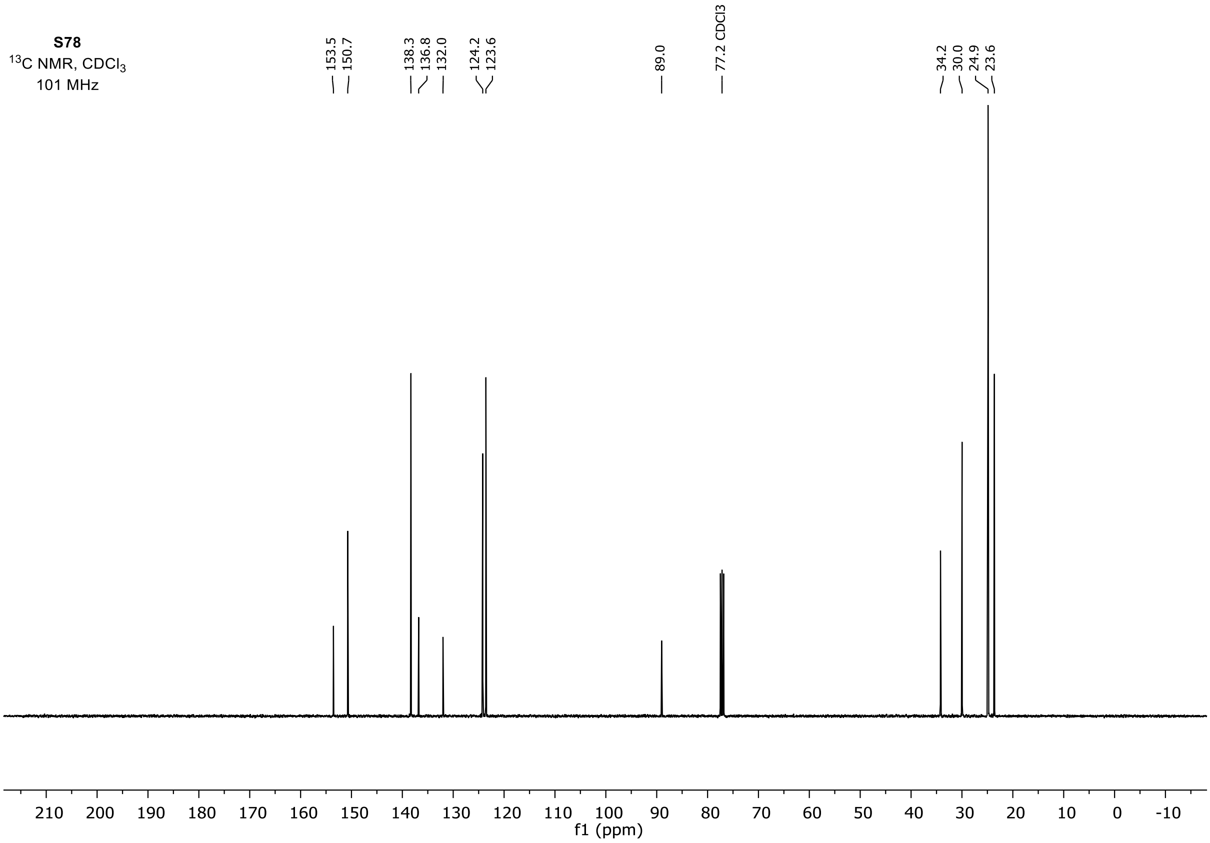

Figure 204. ^{13}C-NMR (101 MHz, $CDCl_3$) of sulfonamide **S78**.

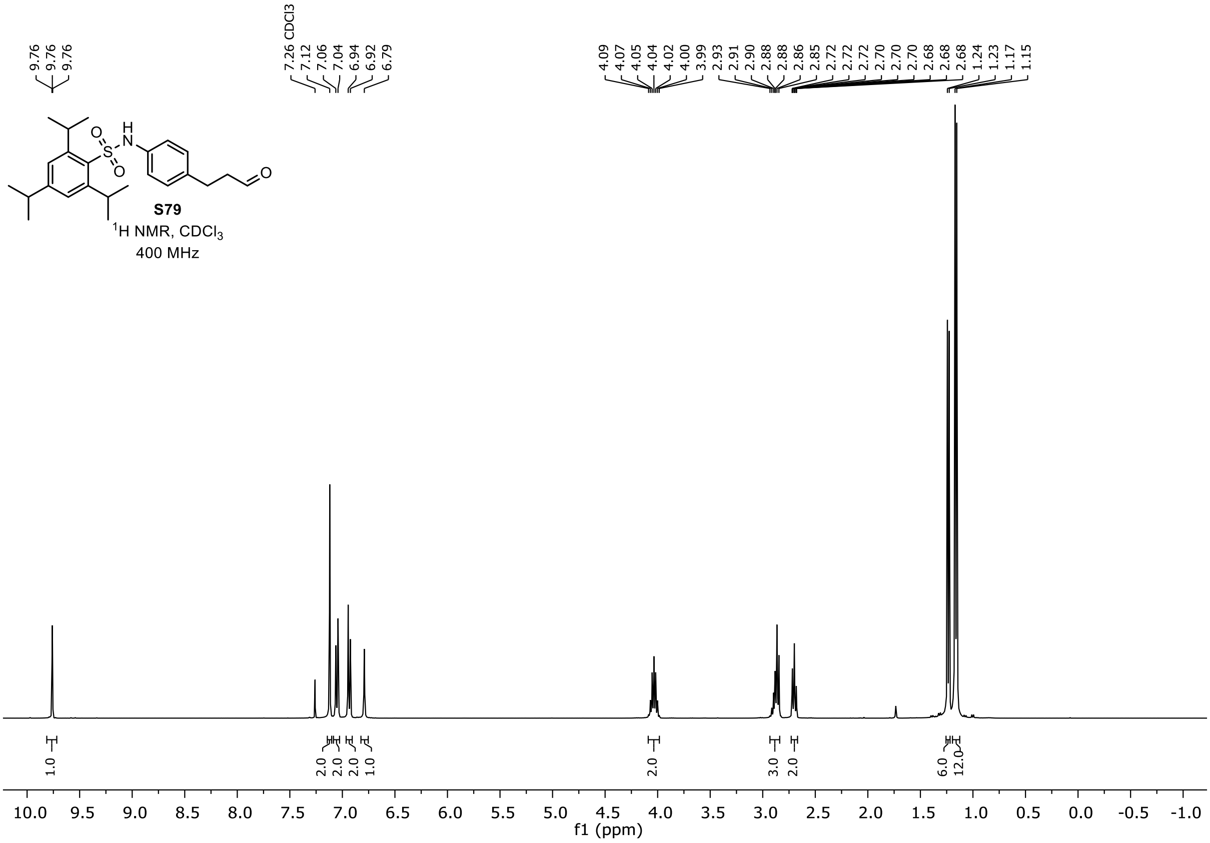

Figure 205. ^{1}H-NMR (400 MHz, $CDCl_3$) of aldehyde **S79**.

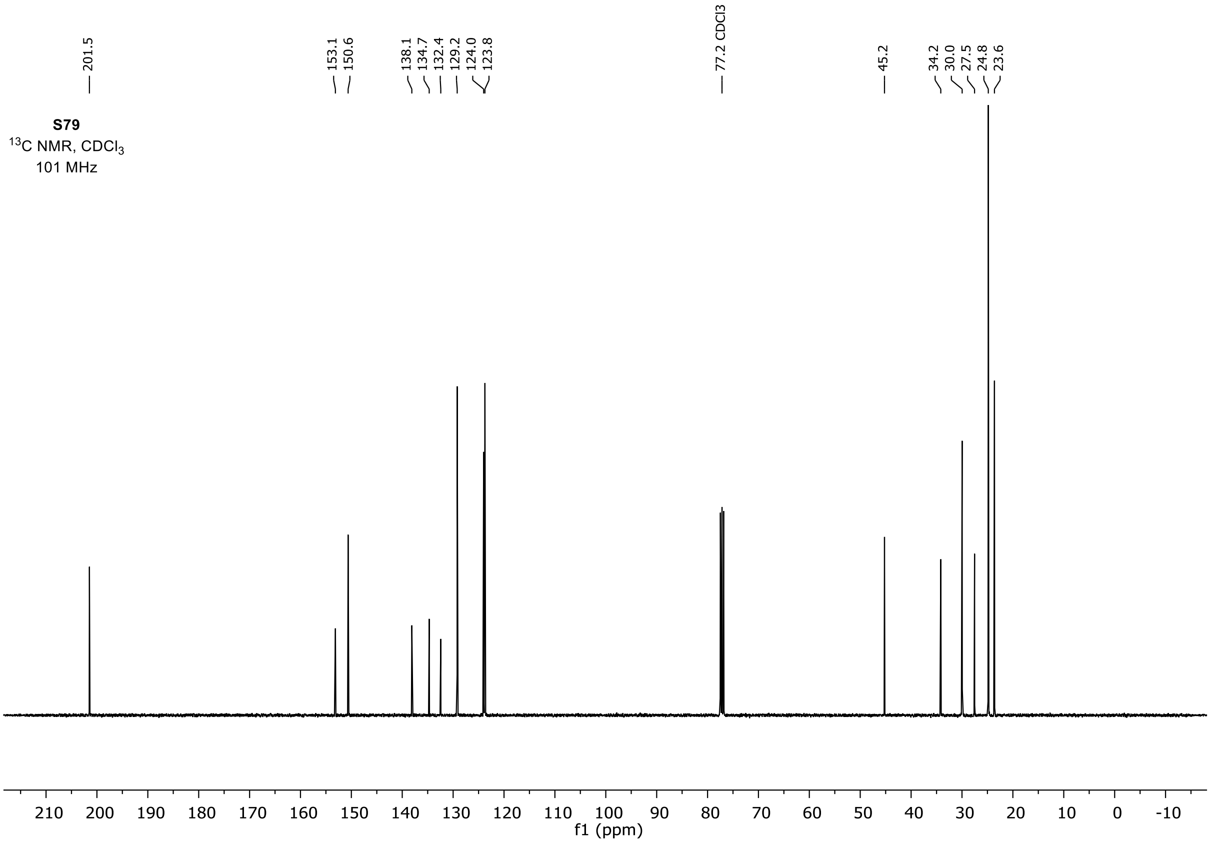

Figure 206. ^{13}C-NMR (101 MHz, $CDCl_3$) of aldehyde **S79**.

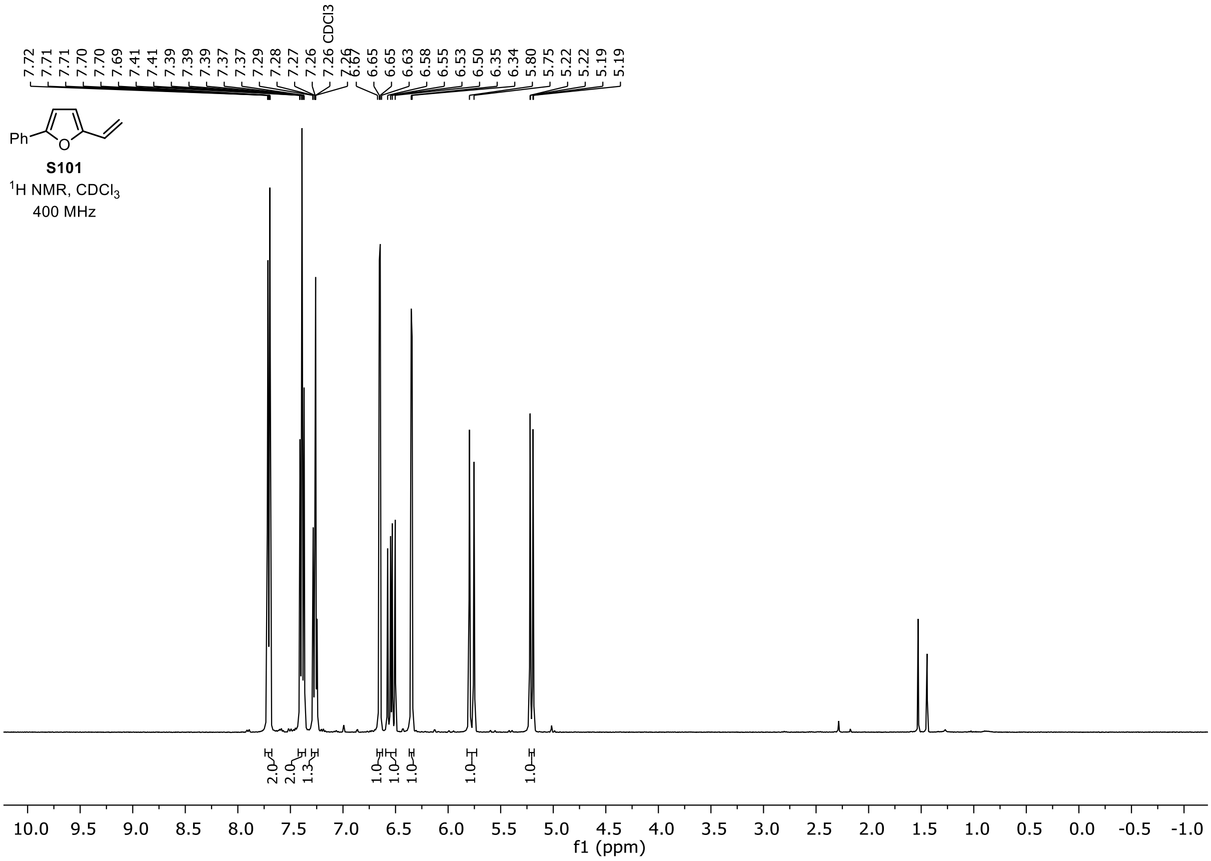

Figure 207. ^{1}H-NMR (400 MHz, $CDCl_3$) of 2-phenyl-5-vinylfuran **S101**.

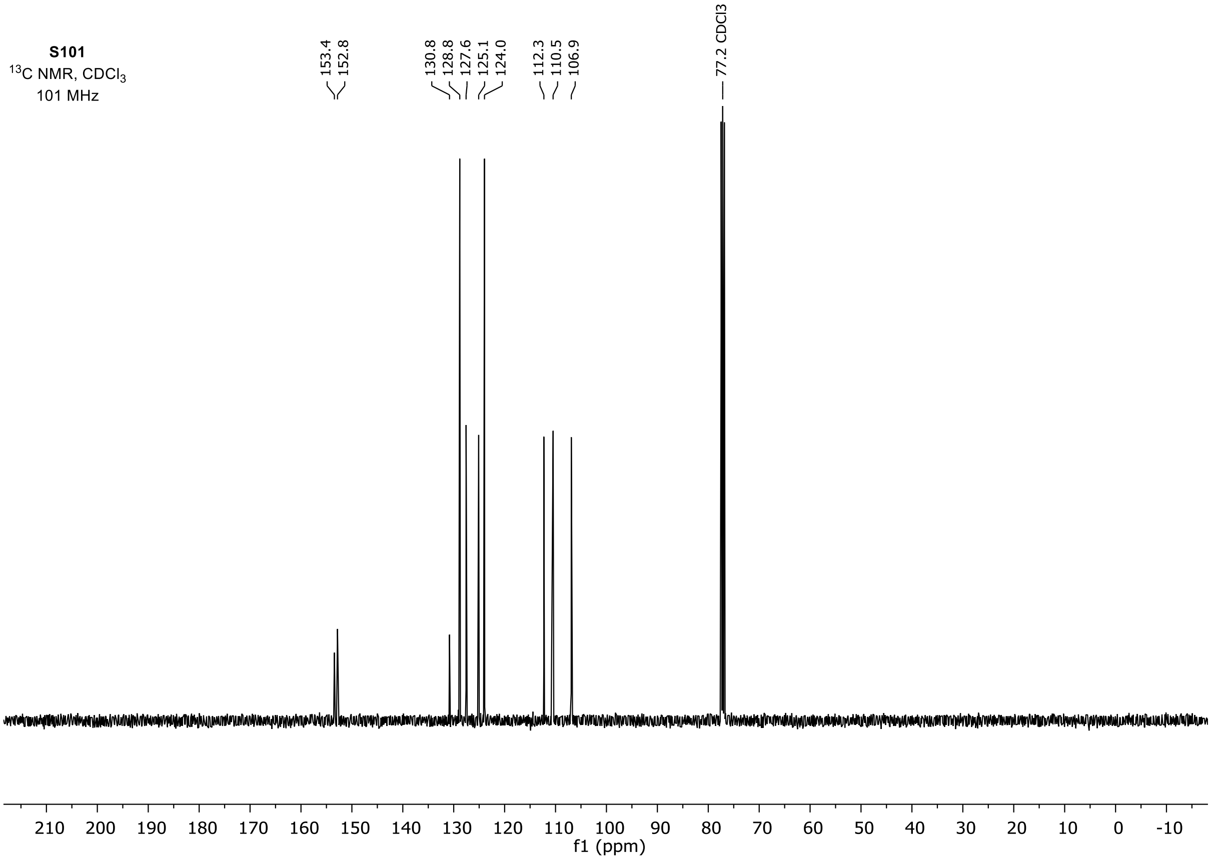

Figure 208. ^{13}C-NMR (101 MHz, $CDCl_3$) of 2-phenyl-5-vinylfuran **S101**.

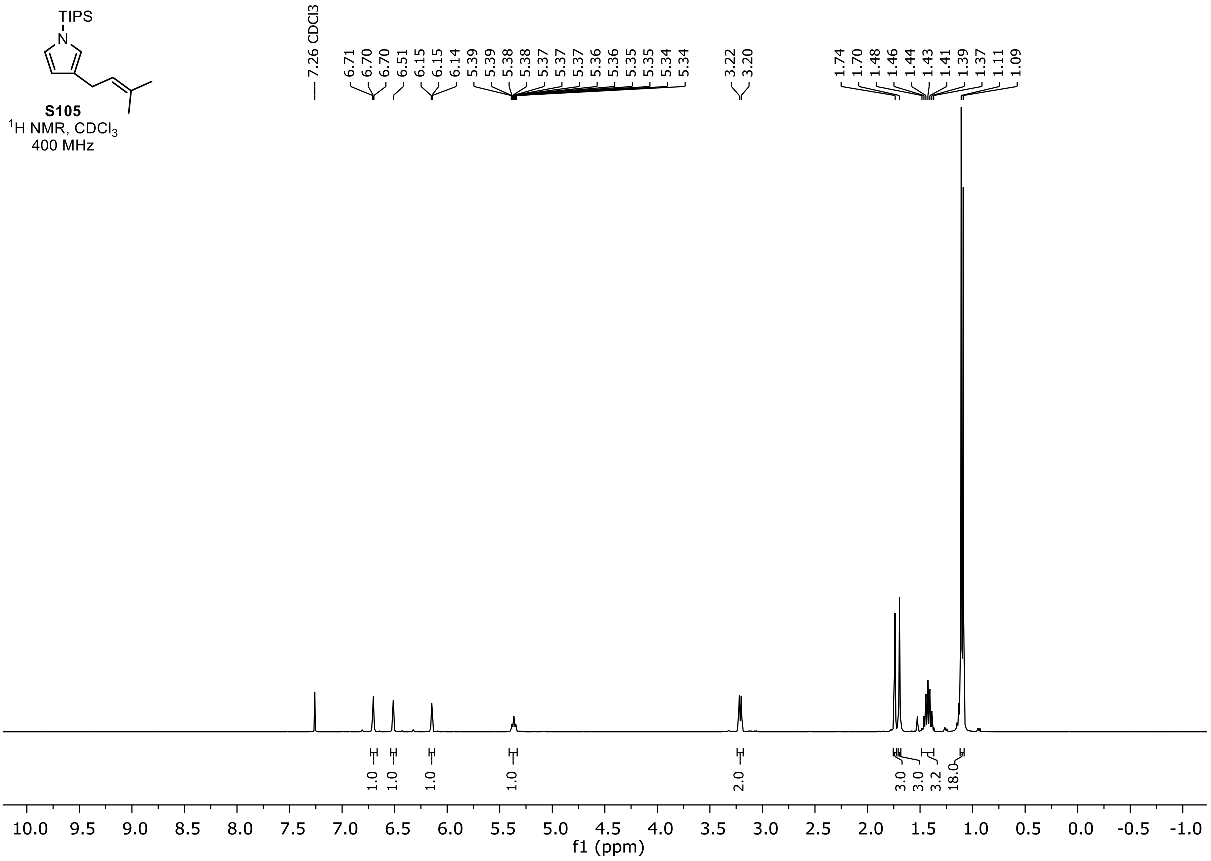

Figure 209. ^{1}H-NMR (400 MHz, $CDCl_3$) of 3-prenyl-*N*-TIPS pyrrole **S105**.

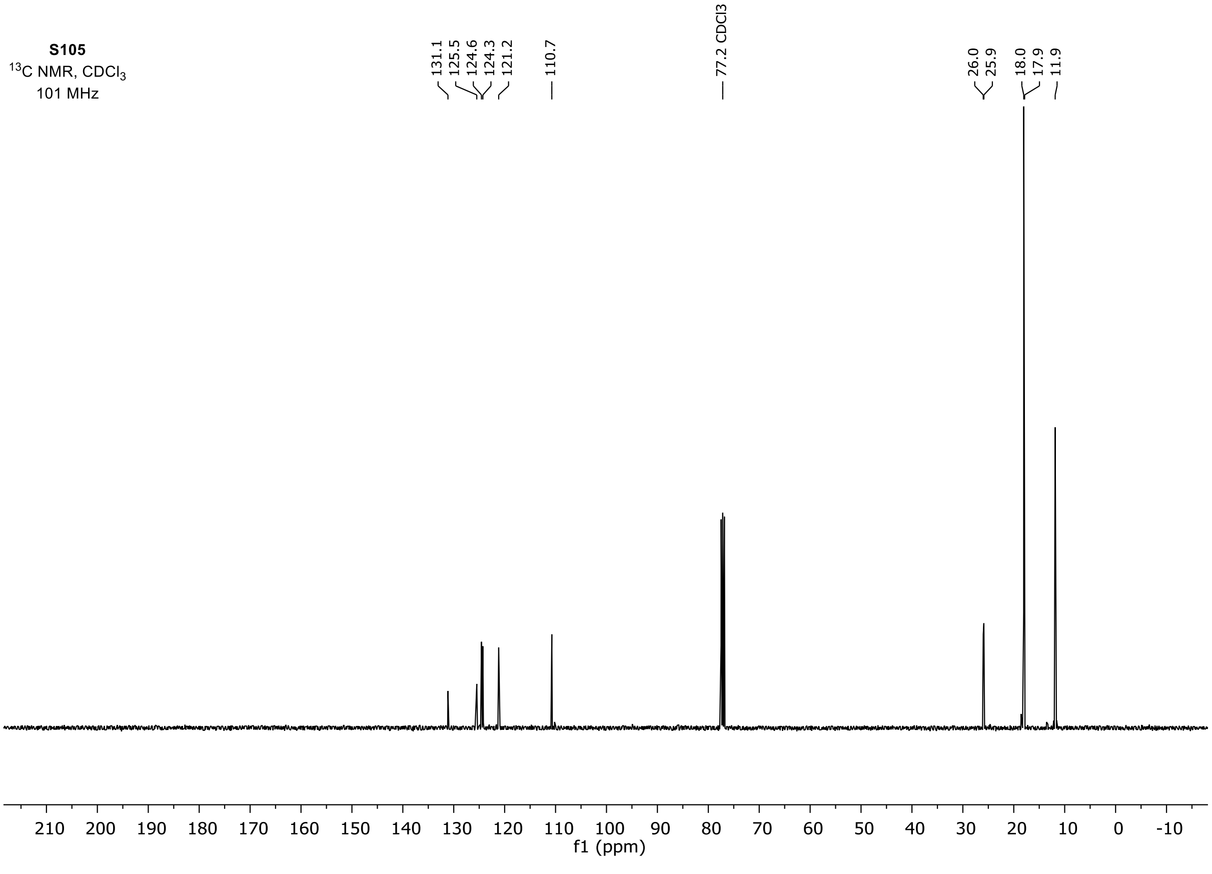

Figure 210. ^{13}C-NMR (101 MHz, $CDCl_3$) of 3-prenyl-*N*-TIPS pyrrole **S105**.

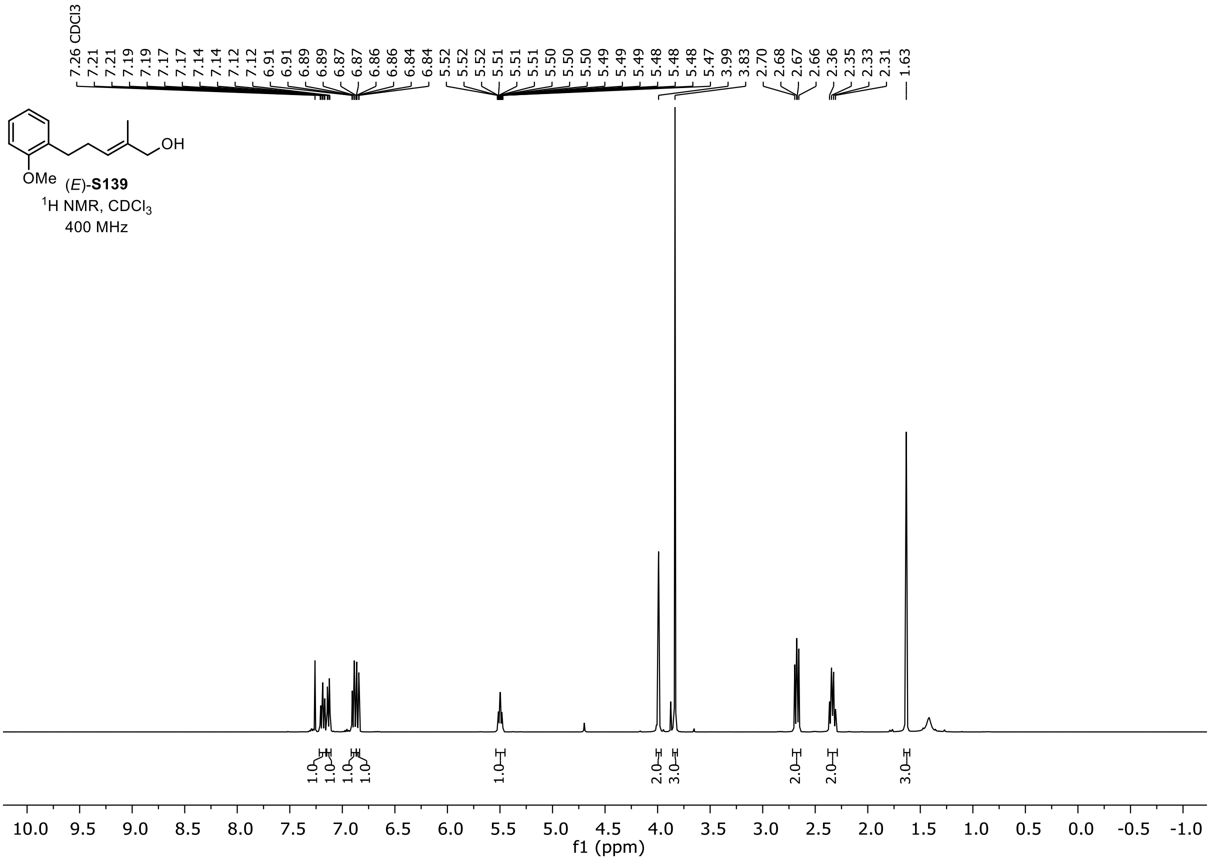

Figure 211. ^{1}H-NMR (400 MHz, $CDCl_3$) of allylic alcohol (*E*)-**S139**.

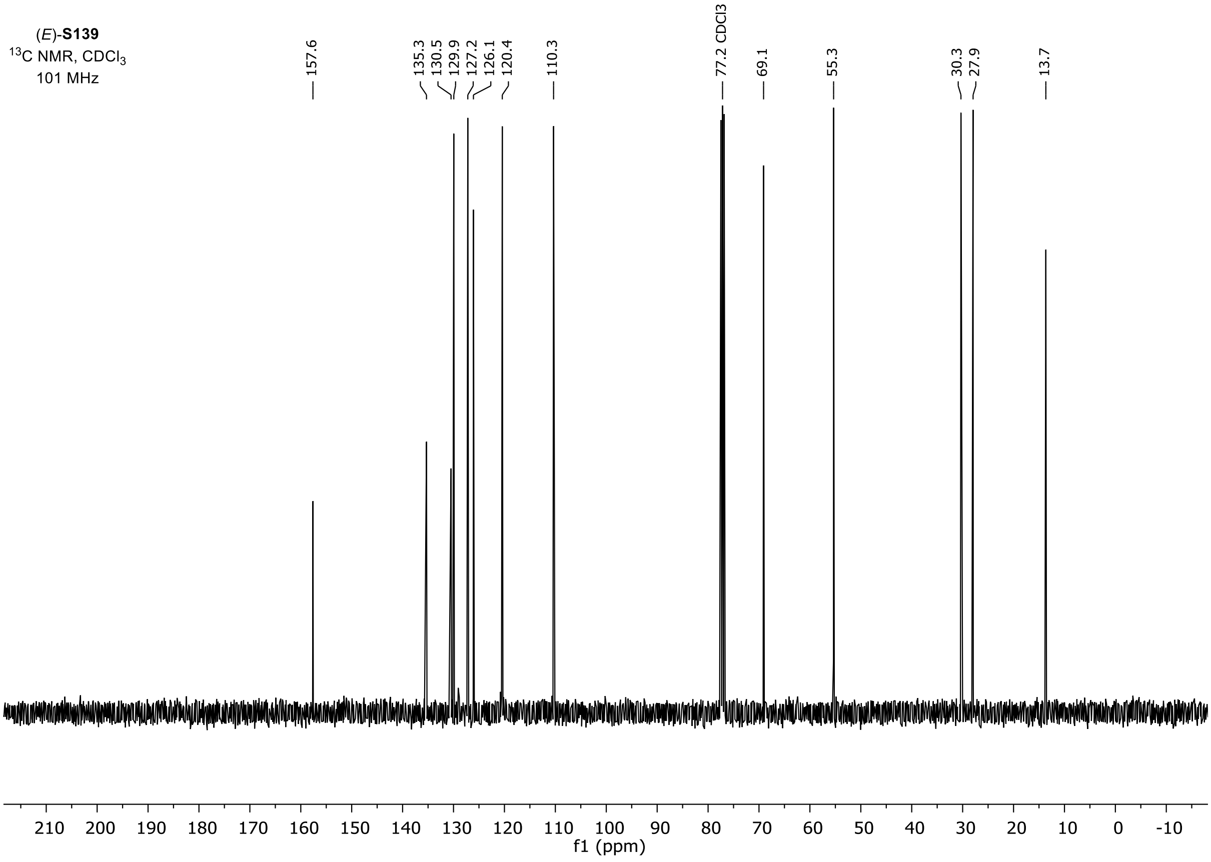

Figure 212. ^{13}C-NMR (101 MHz, $CDCl_3$) of allylic alcohol (*E*)-**S139**.

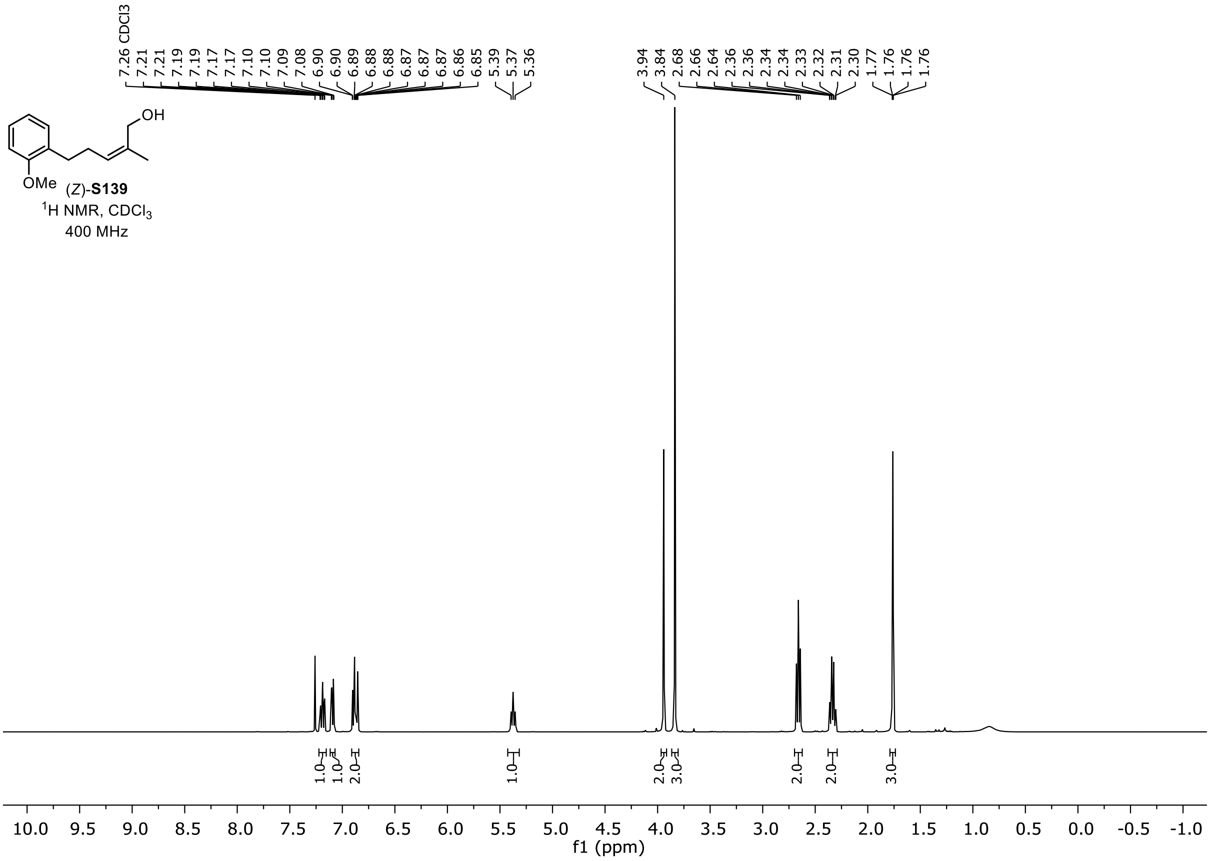

Figure 213. ^{1}H-NMR (400 MHz, $CDCl_3$) of allylic alcohol (*Z*)-**S139**.

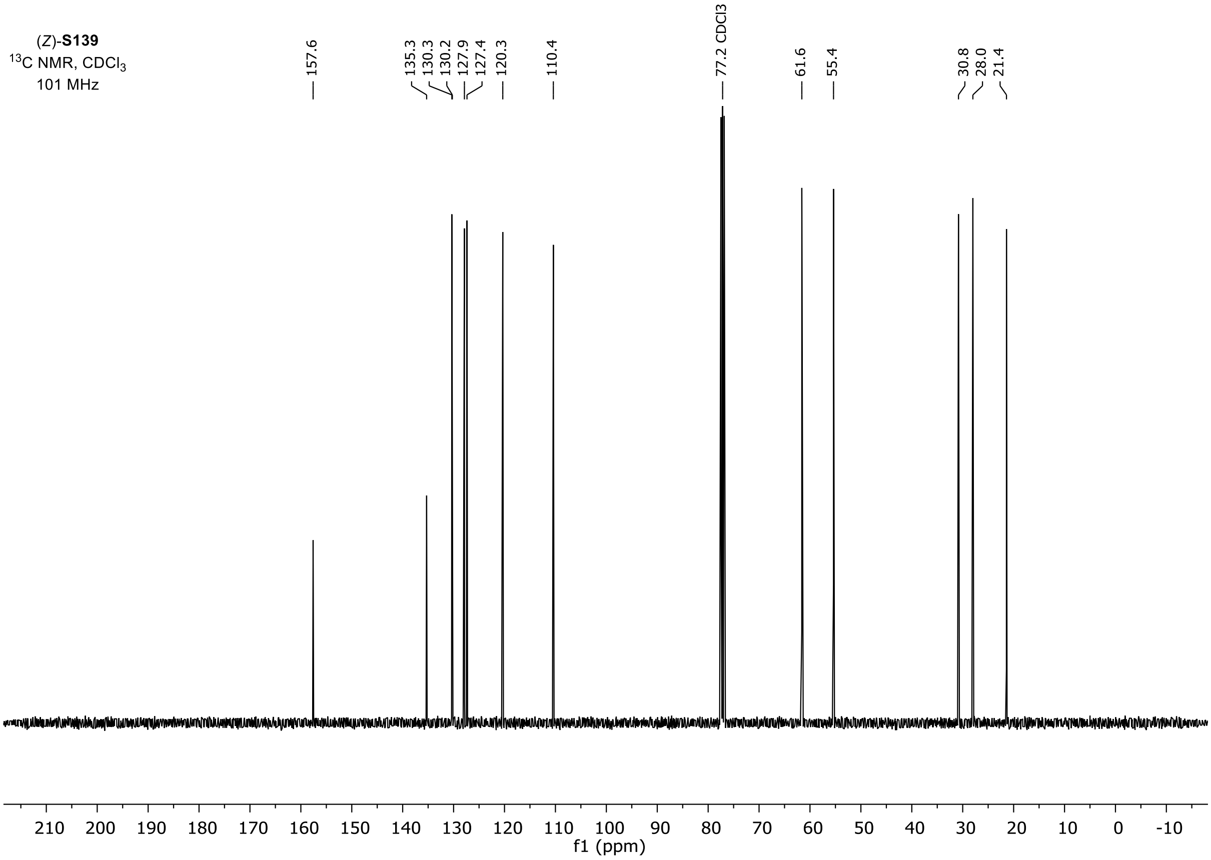

Figure 214. ^{13}C-NMR (101 MHz, $CDCl_3$) of allylic alcohol (*Z*)-**S139**.

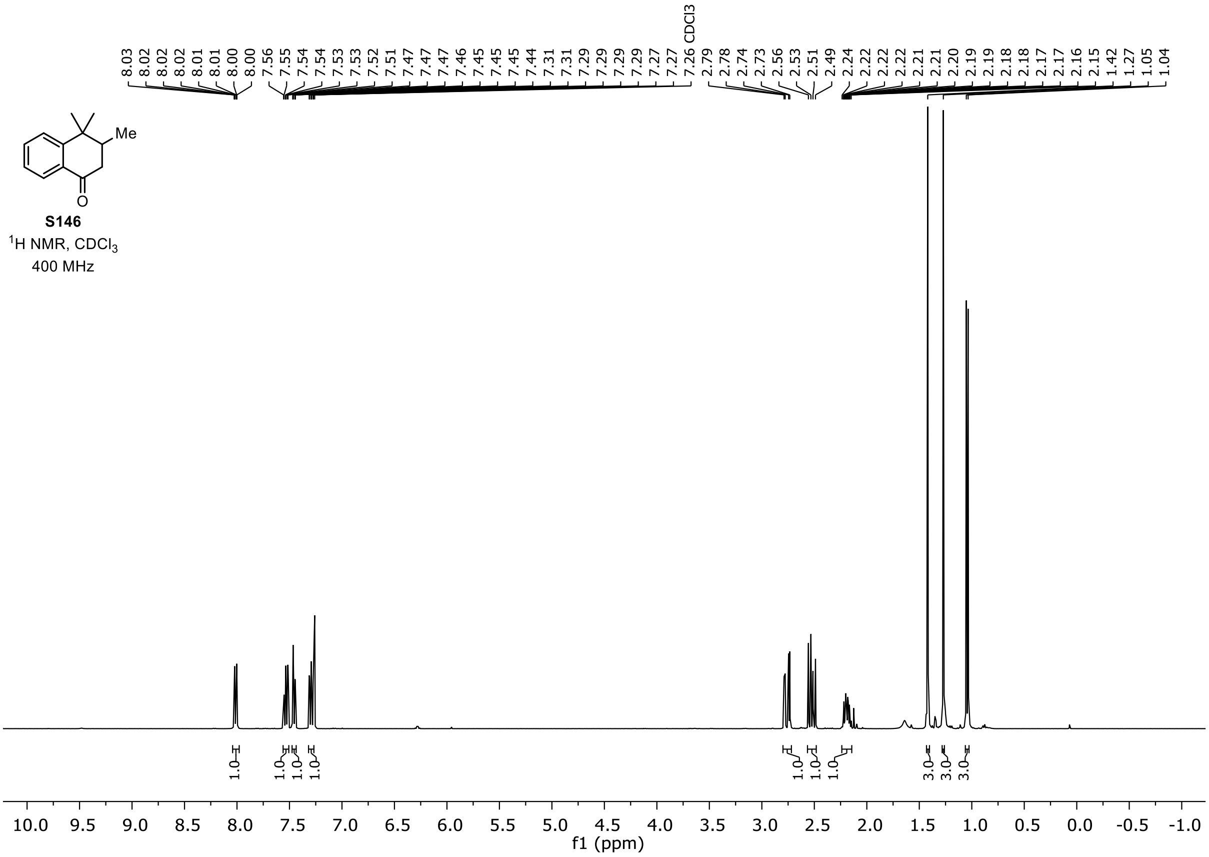

Figure 215. ^{1}H-NMR (400 MHz, $CDCl_3$) of ketone **S146**.

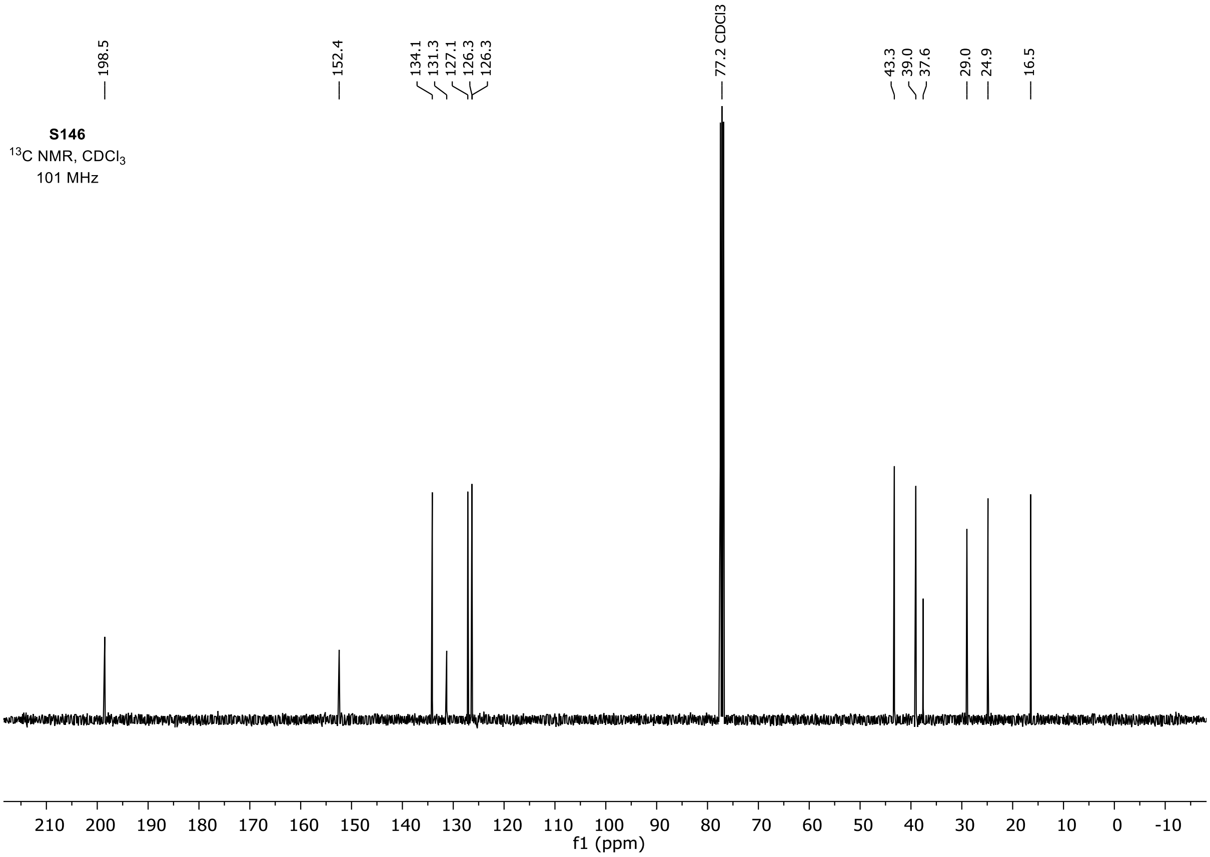

Figure 216. ^{13}C-NMR (101 MHz, $CDCl_3$) of ketone **S146**.

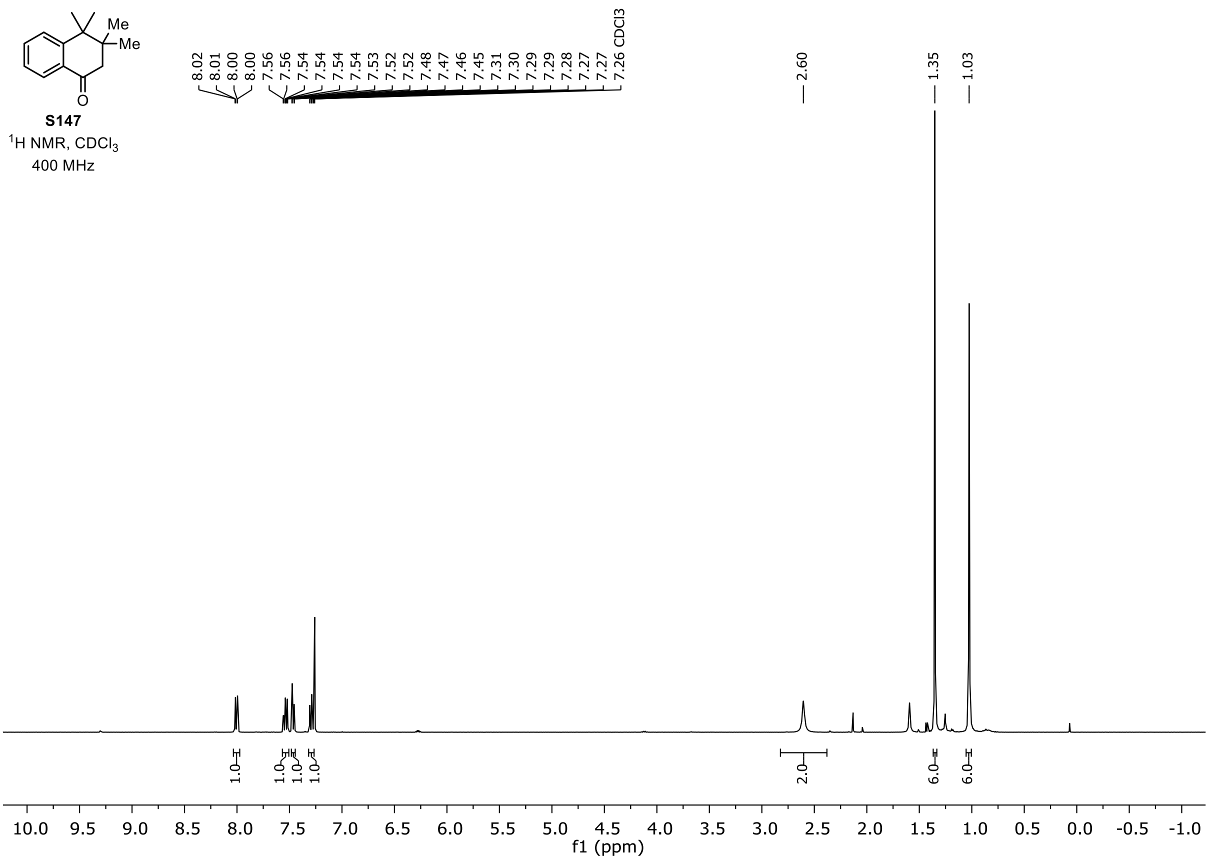

Figure 217. ^{1}H-NMR (400 MHz, $CDCl_3$) of ketone **S147**.

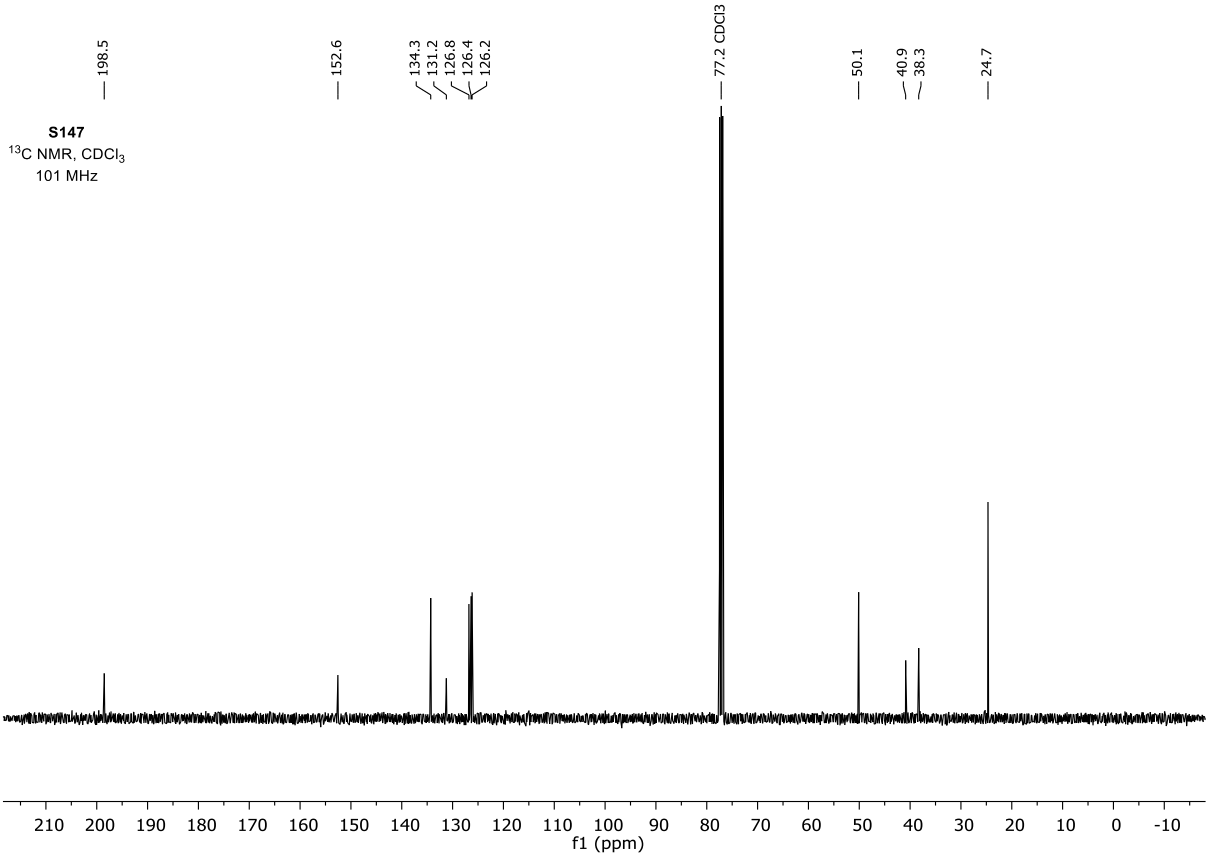

Figure 218. ^{13}C-NMR (101 MHz, $CDCl_3$) of ketone **S147**.

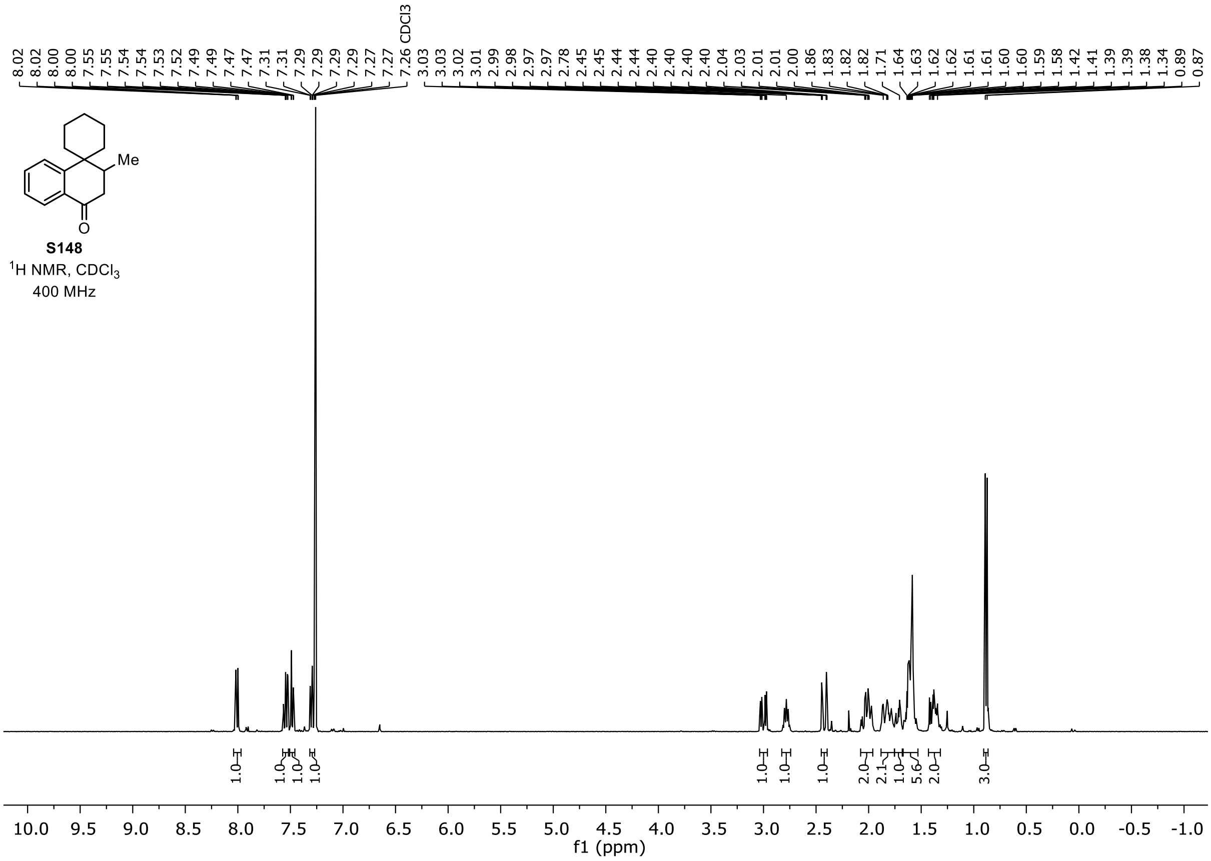

Figure 219. ^{1}H-NMR (400 MHz, $CDCl_3$) of ketone **S148**.

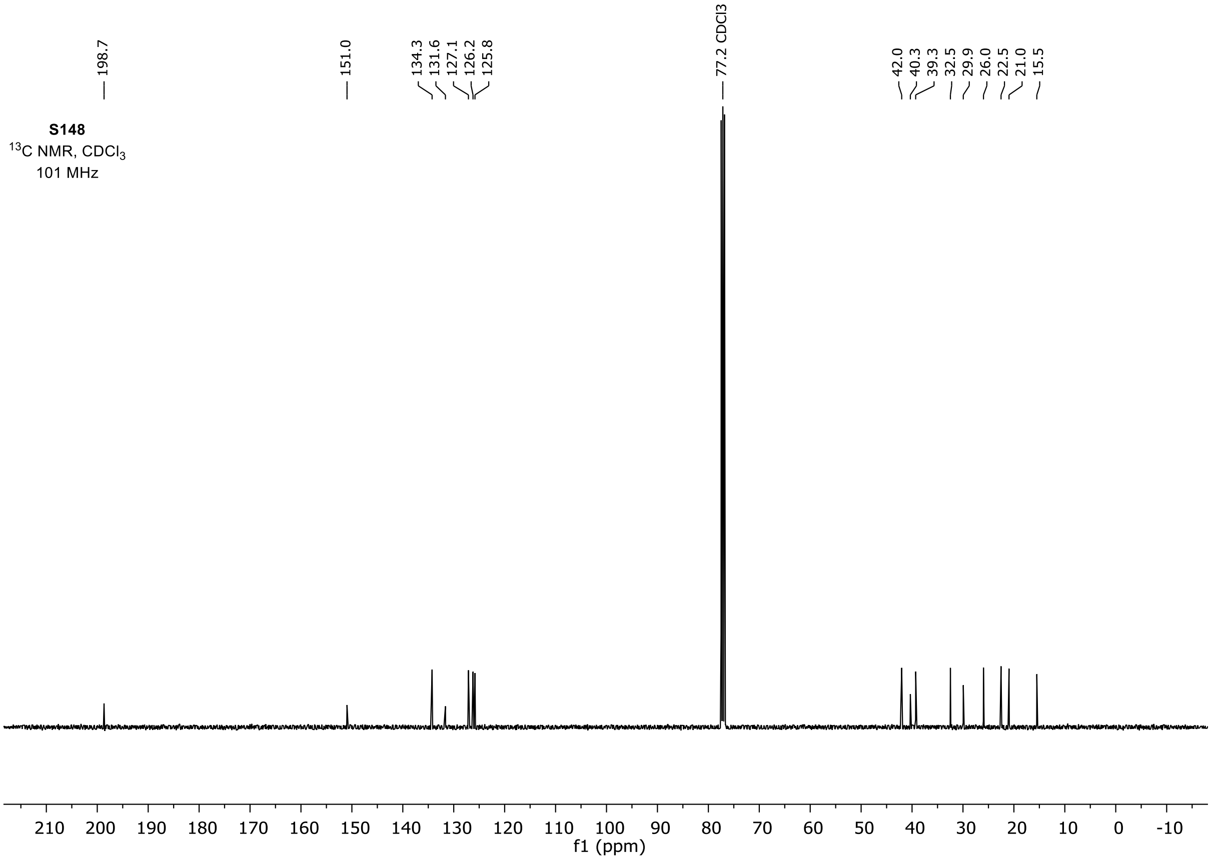

Figure 220. ^{13}C-NMR (101 MHz, $CDCl_3$) of ketone **S148**.

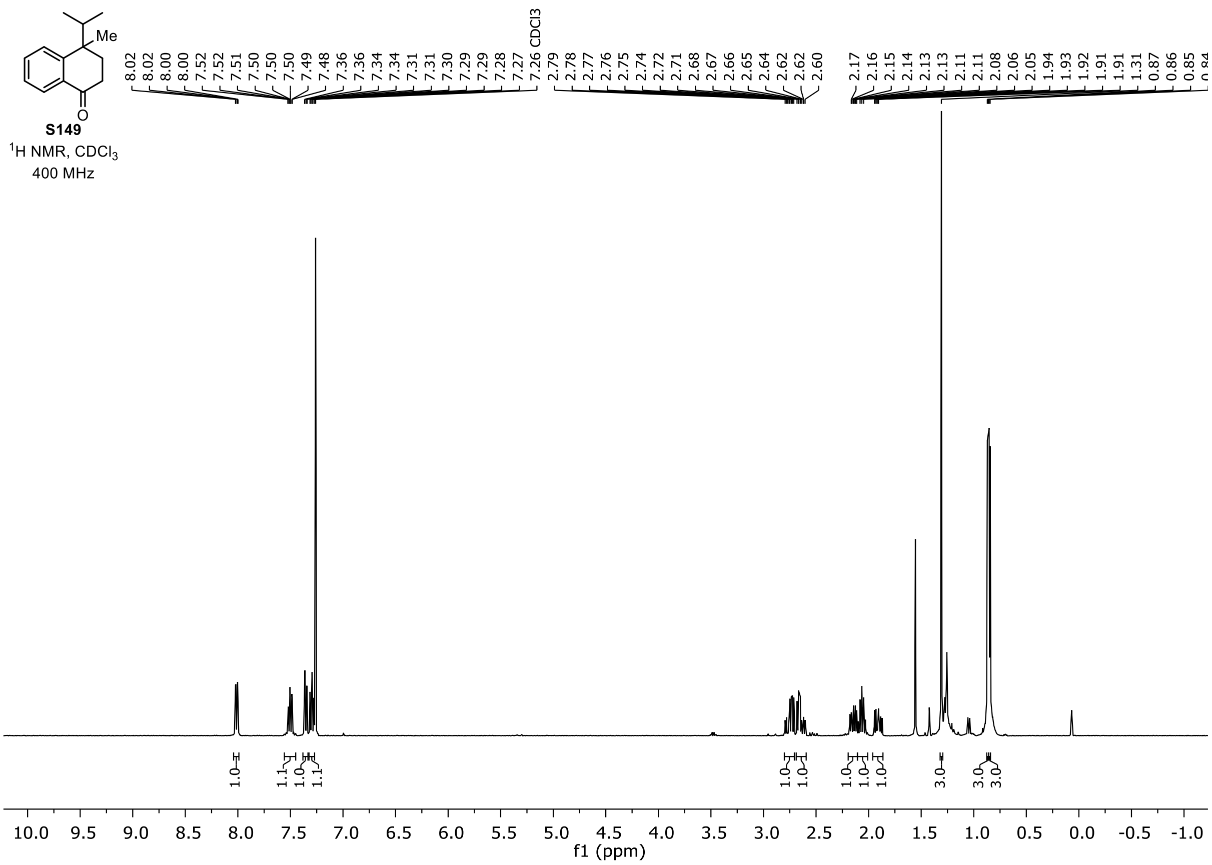

Figure 221. ^{1}H-NMR (400 MHz, $CDCl_3$) of ketone **S149**.

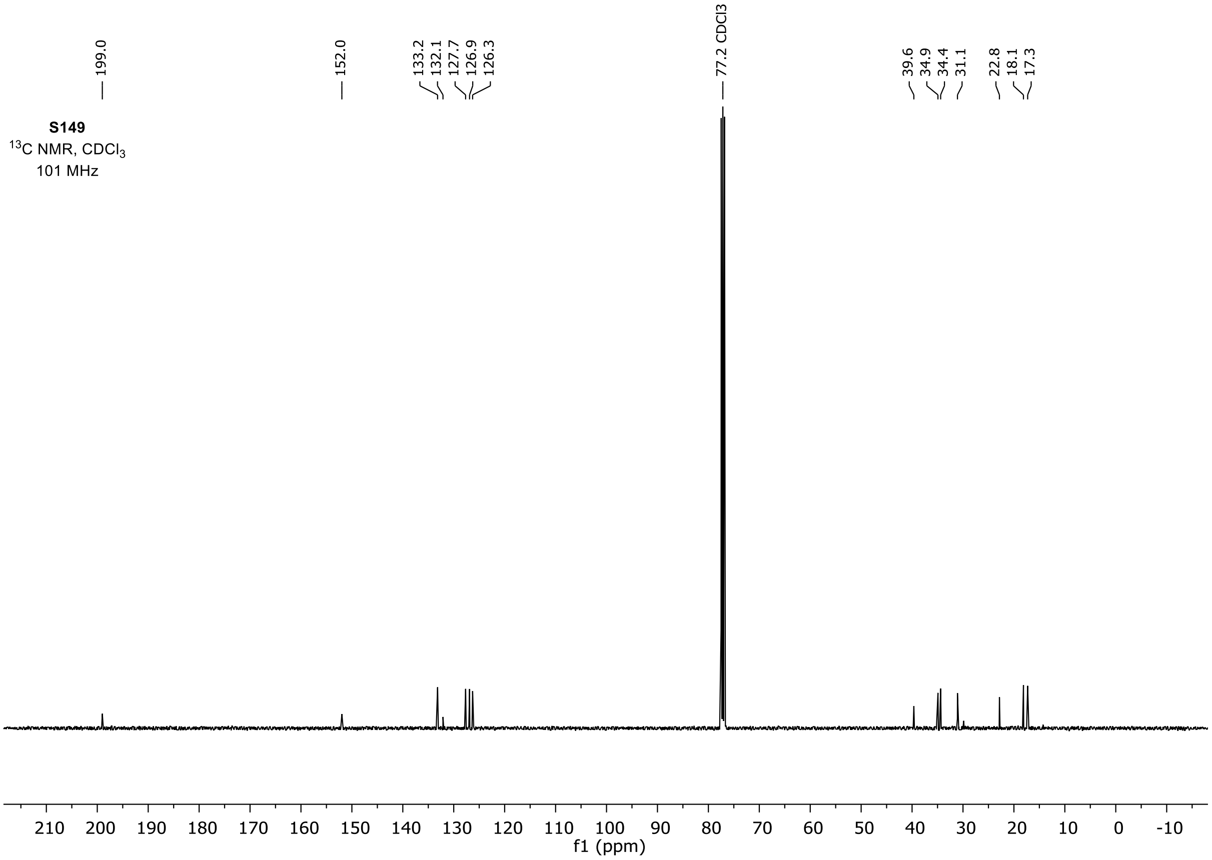

Figure 222. ^{13}C-NMR (101 MHz, $CDCl_3$) of ketone **S149**.

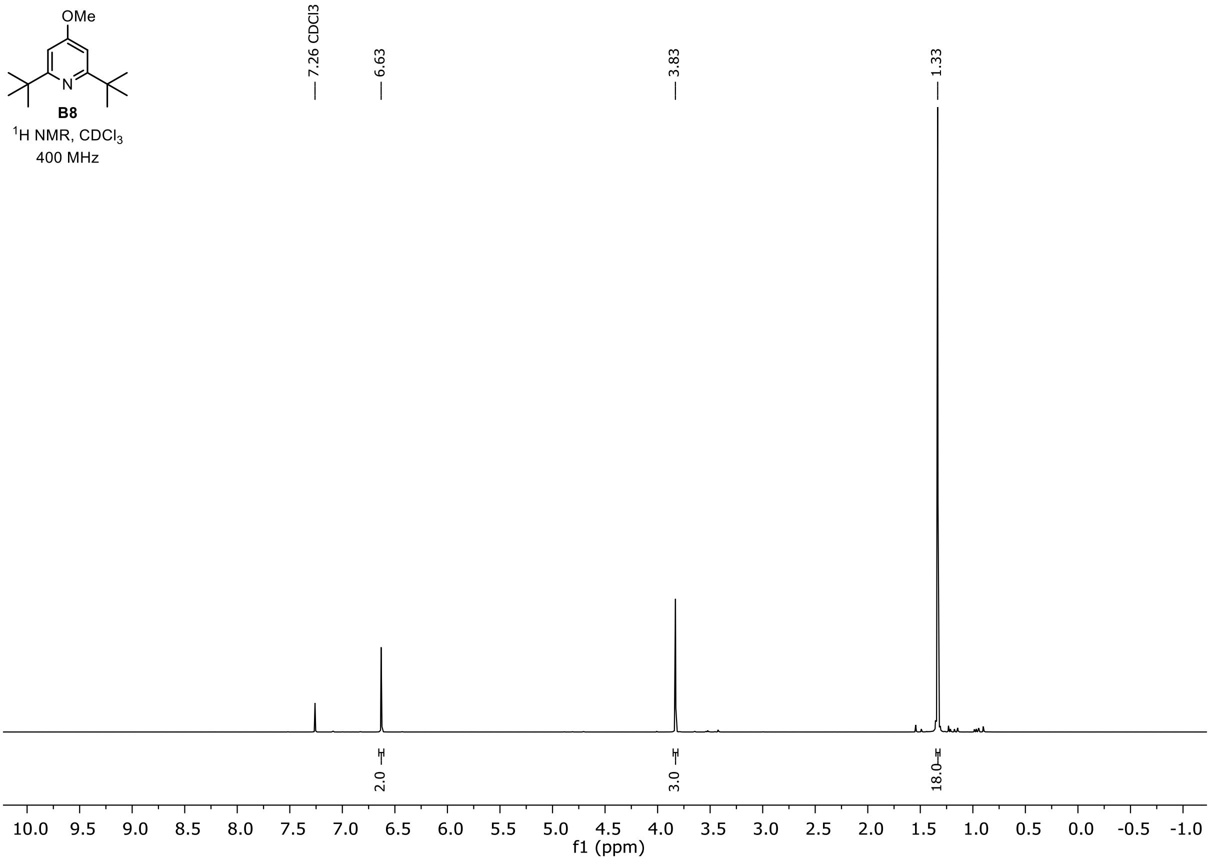

Figure 223. ^{1}H-NMR (400 MHz, $CDCl_3$) of 2,6-(*t*-Bu)$_2$-4-MeO-pyridine **B8**.

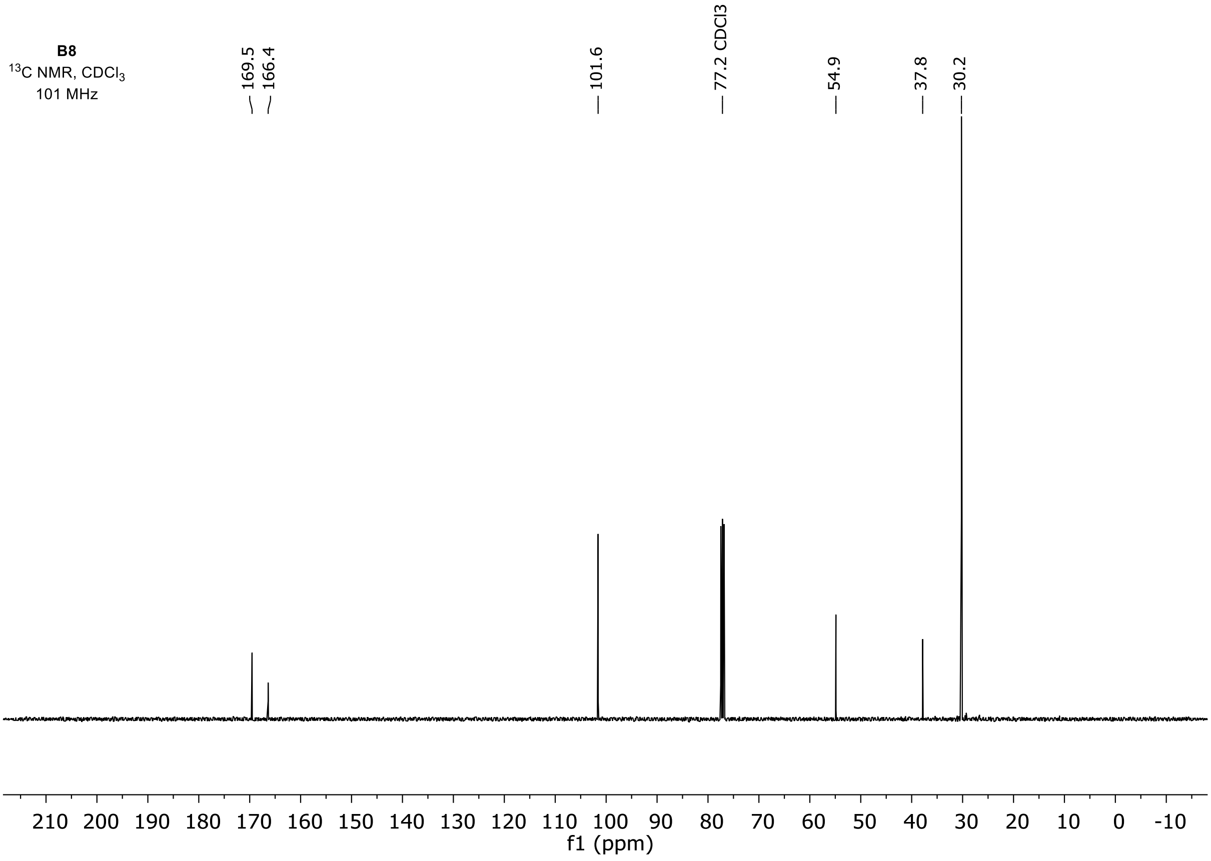

Figure 224. ^{13}C-NMR (101 MHz, $CDCl_3$) of 2,6-(*t*-Bu)$_2$-4-MeO-pyridine **B8**.

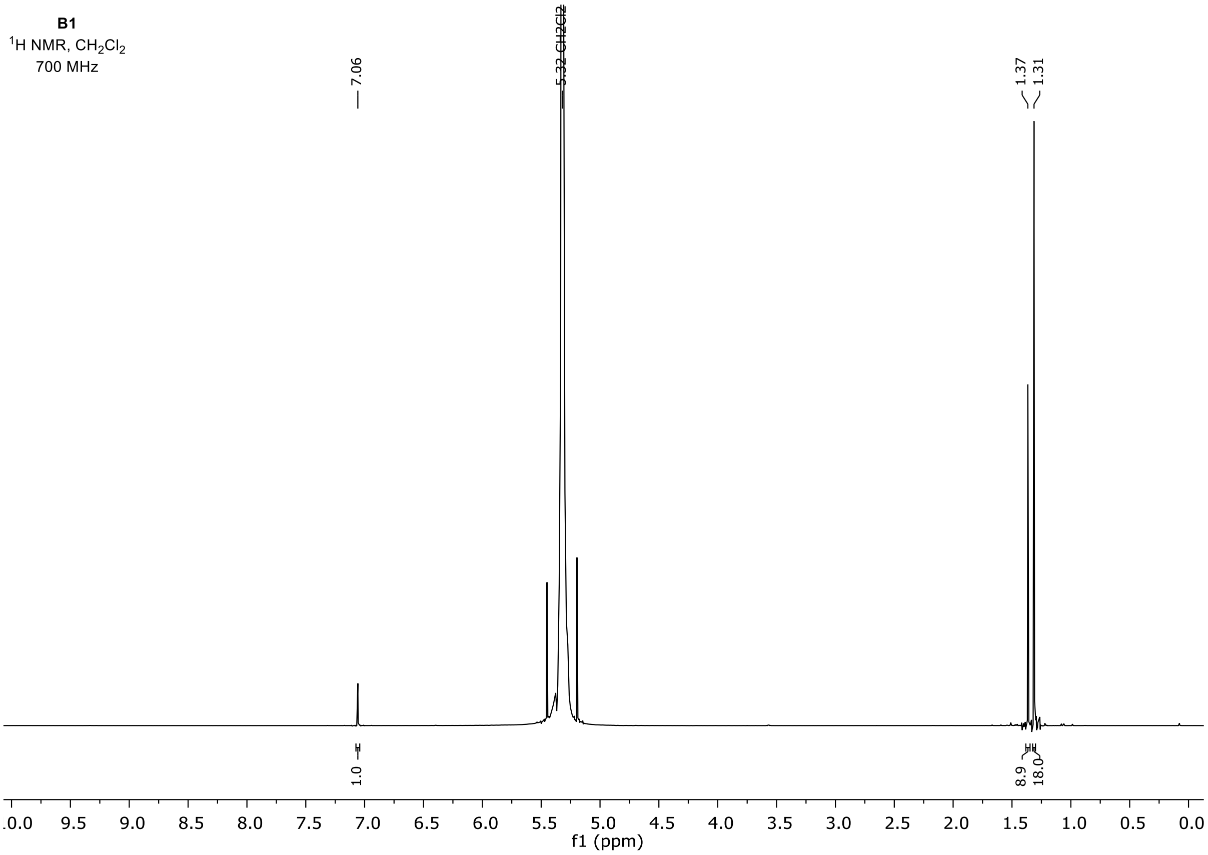

Figure 225. ^{1}H-NMR (700 MHz, CH_2Cl_2) of 2,4,6-tri-*tert*-butylpyrimidine **B1**.

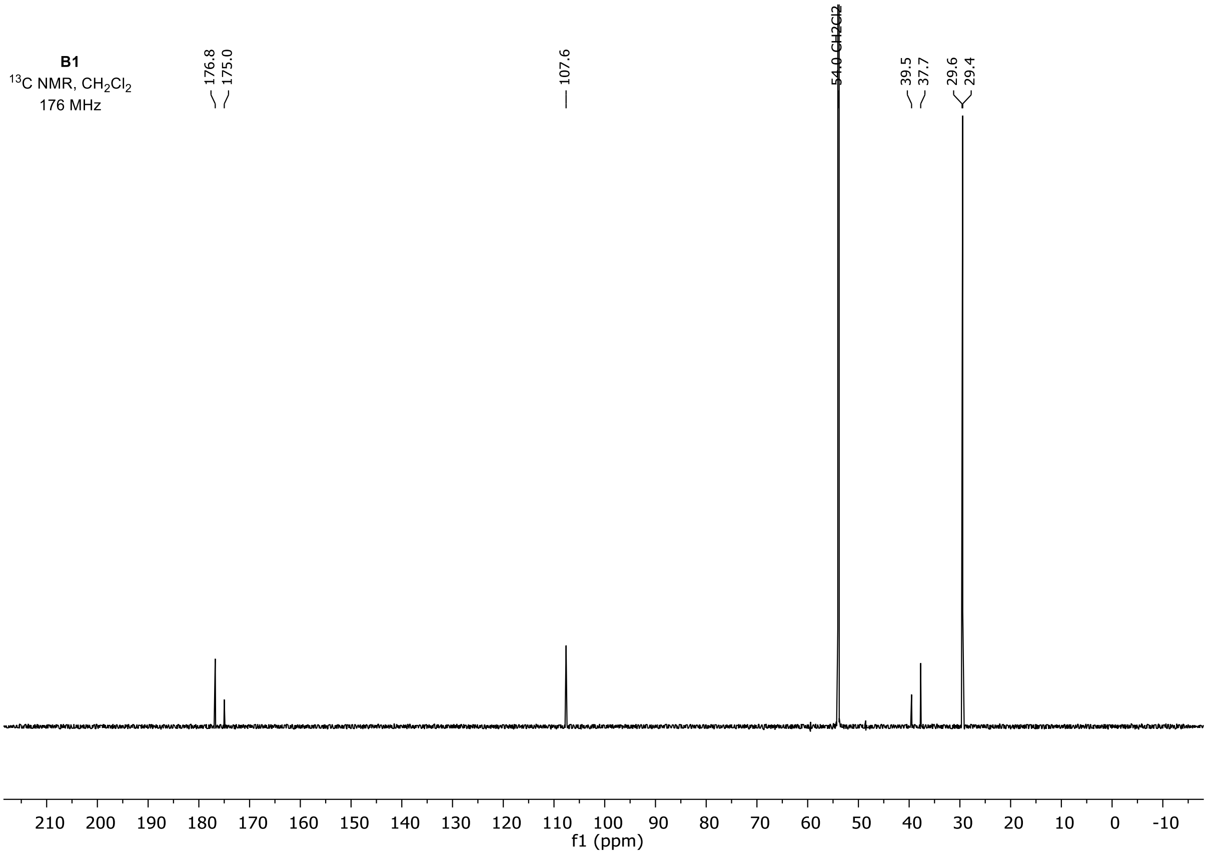

Figure 226. ^{13}C-NMR (176 MHz, CH_2Cl_2) of 2,4,6-tri-*tert*-butylpyrimidine **B1**.

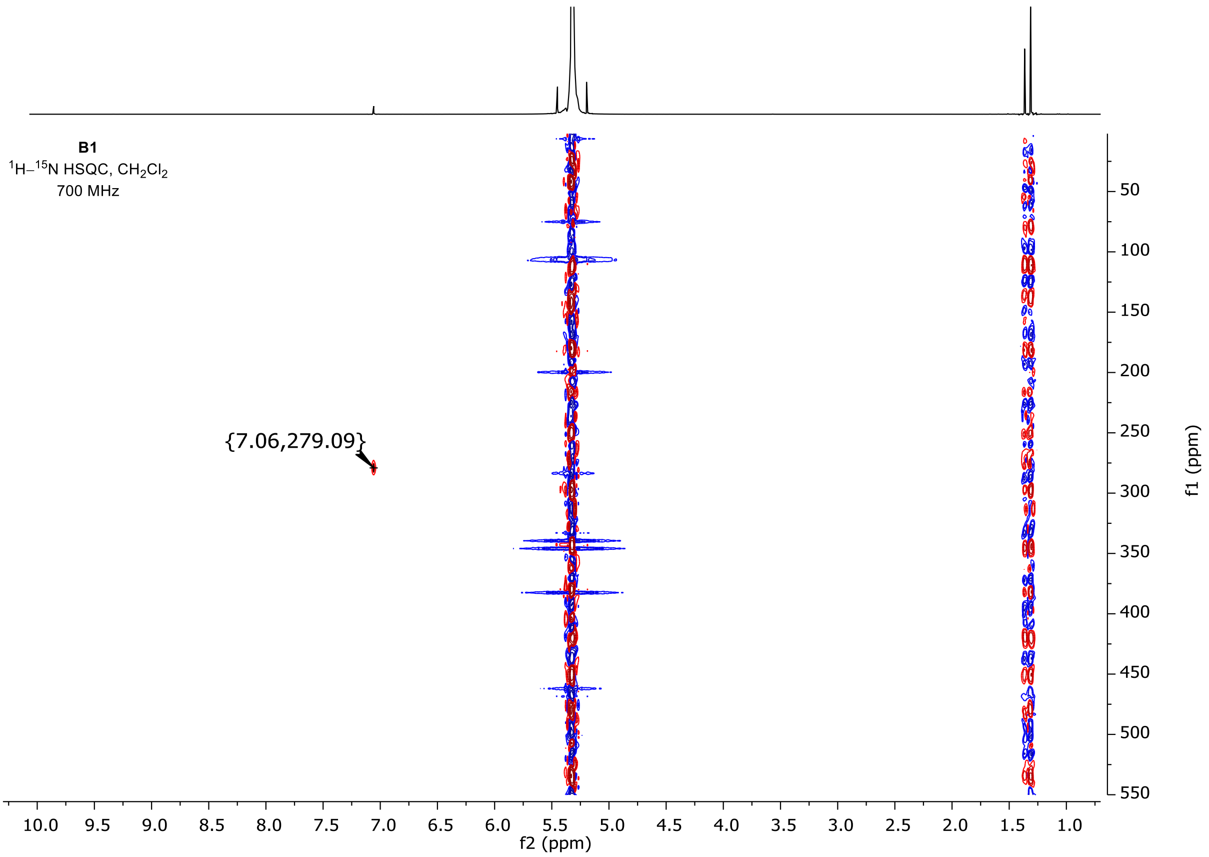

Figure 227. ^{1}H-^{15}N-HSQC (700 MHz, CH_2Cl_2) of 2,4,6-tri-*tert*-butylpyrimidine **B1**.

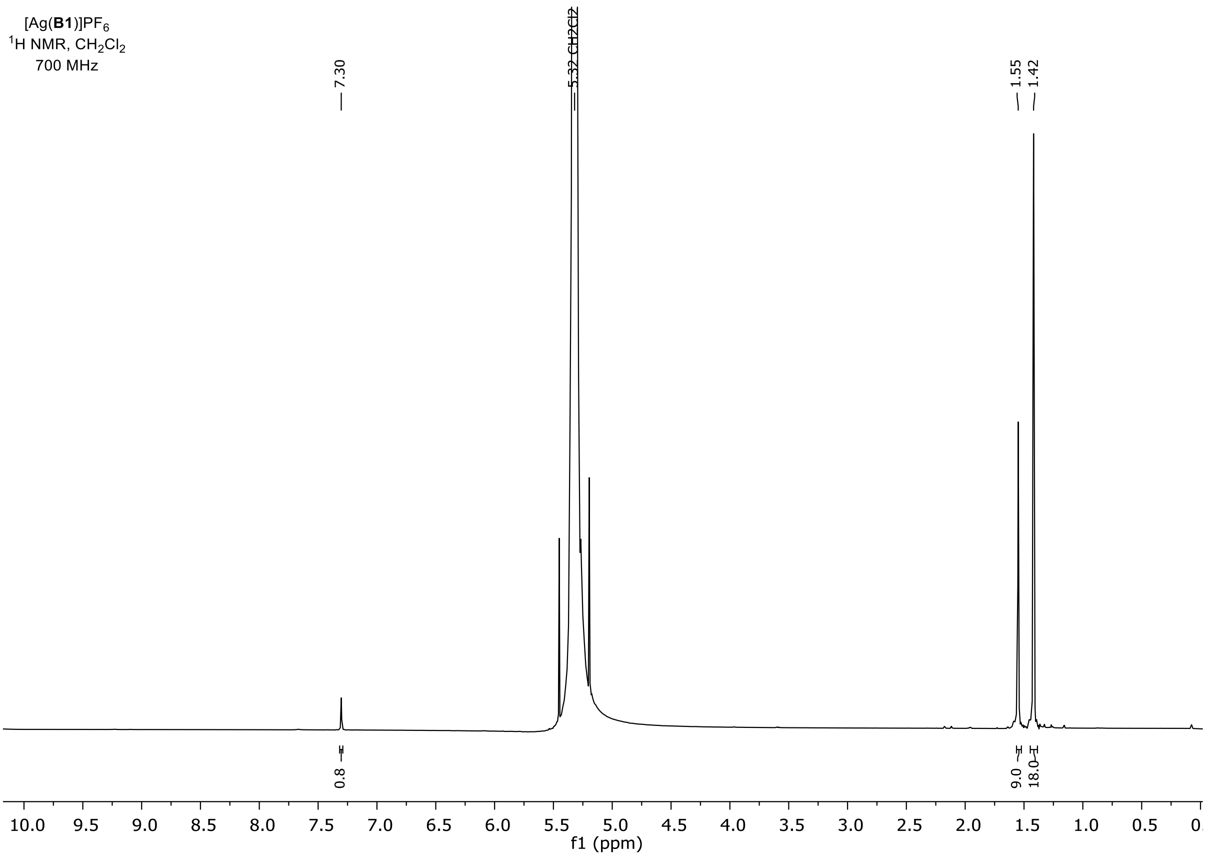

Figure 228. ^{1}H-NMR (700 MHz, CH_2Cl_2) of a solution of 2,4,6-tri-*tert*-butylpyrimidine **B1** and $AgPF_6$ (1:1).

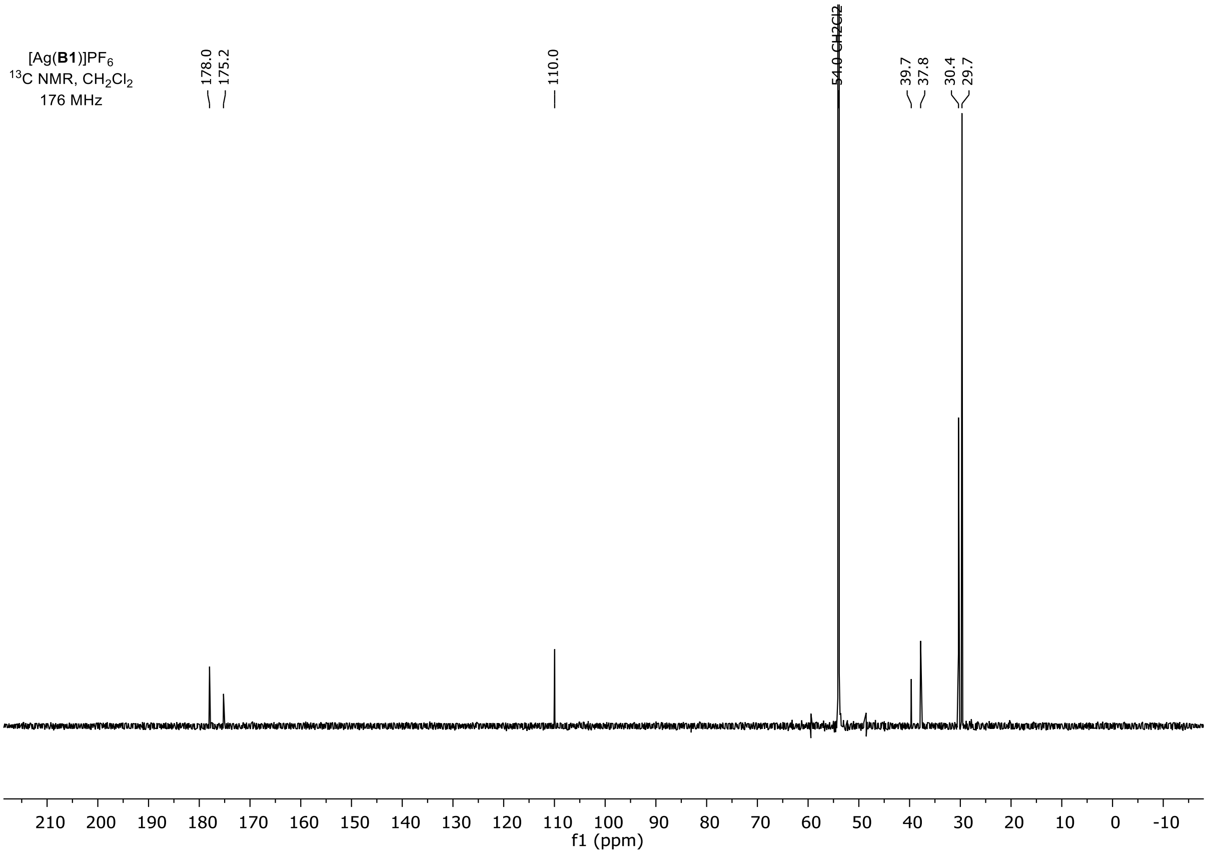

Figure 229. ^{13}C-NMR (176 MHz, CH_2Cl_2) of a solution of 2,4,6-tri-*tert*-butylpyrimidine **B1** and $AgPF_6$ (1:1).

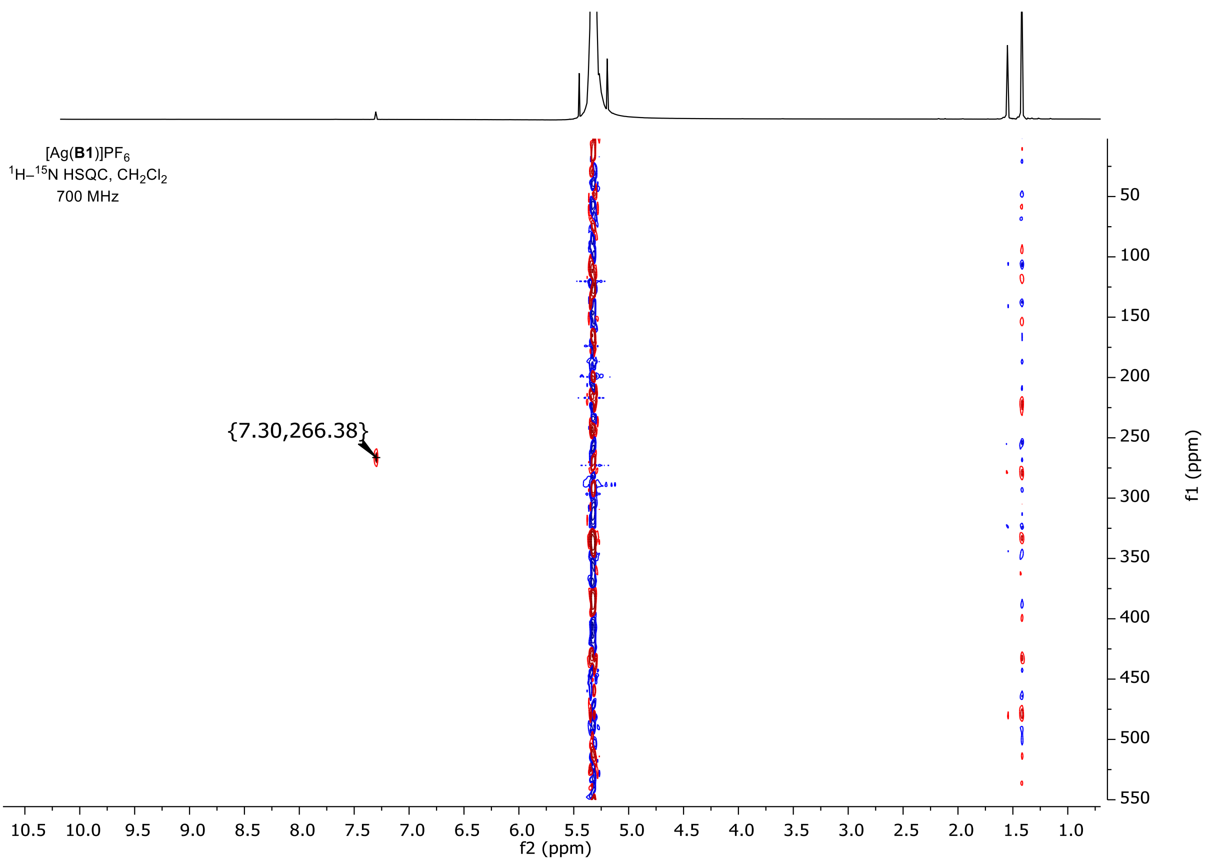

Figure 230. ^{1}H-^{15}N-HSQC (700 MHz, CH_2Cl_2) of a solution of 2,4,6-tri-*tert*-butylpyrimidine **B1** and $AgPF_6$ (1:1).

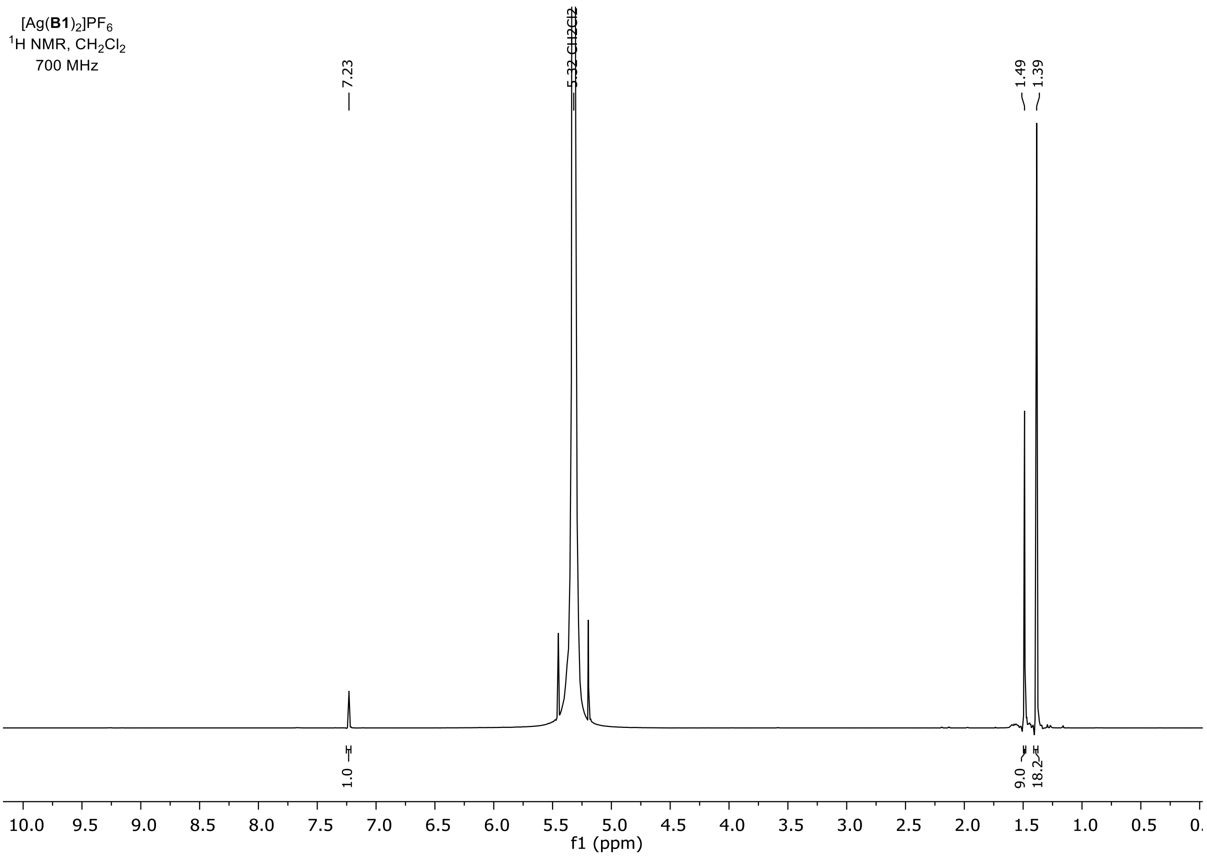

Figure 231. ^{1}H-NMR (700 MHz, CH_2Cl_2) of a solution of 2,4,6-tri-*tert*-butylpyrimidine **B1** and $AgPF_6$ (2:1).

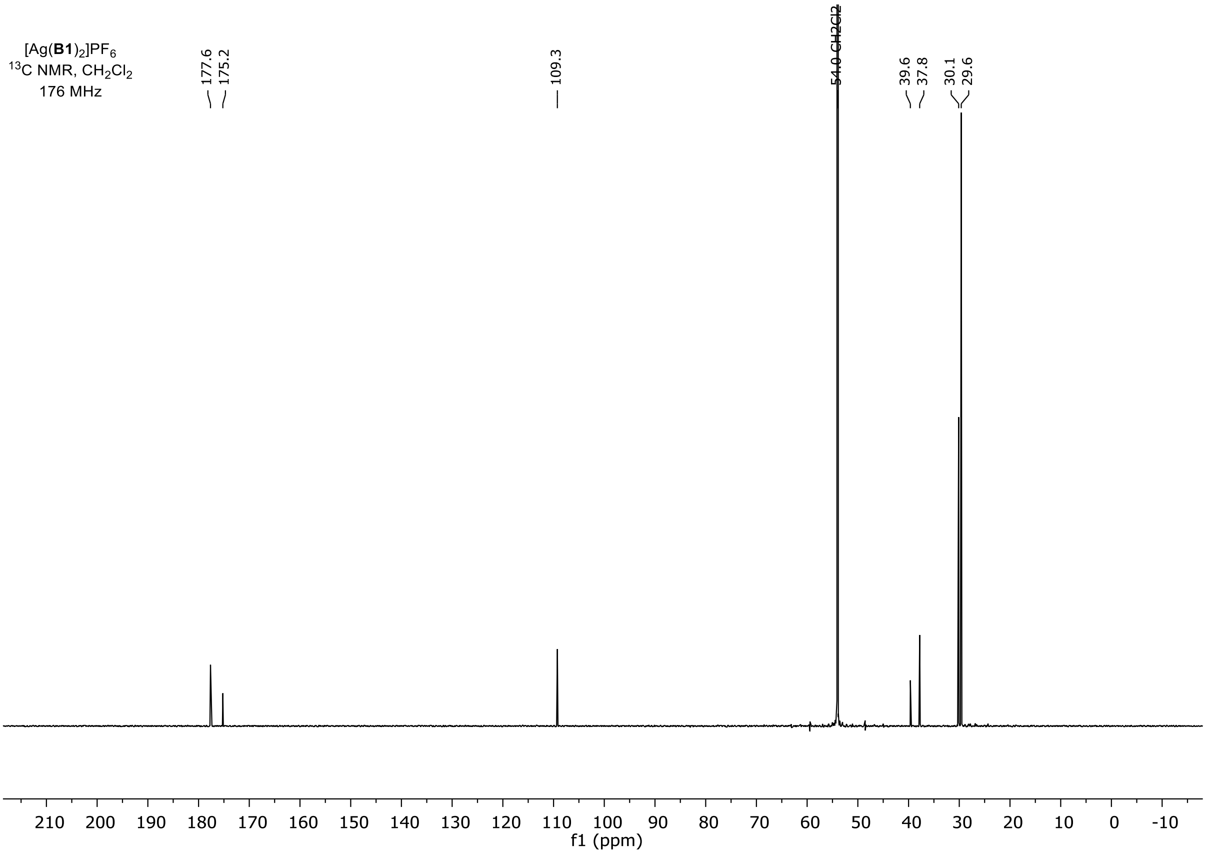

Figure 232. ^{13}C-NMR (176 MHz, CH_2Cl_2) of a solution of 2,4,6-tri-*tert*-butylpyrimidine **B1** and $AgPF_6$ (2:1).

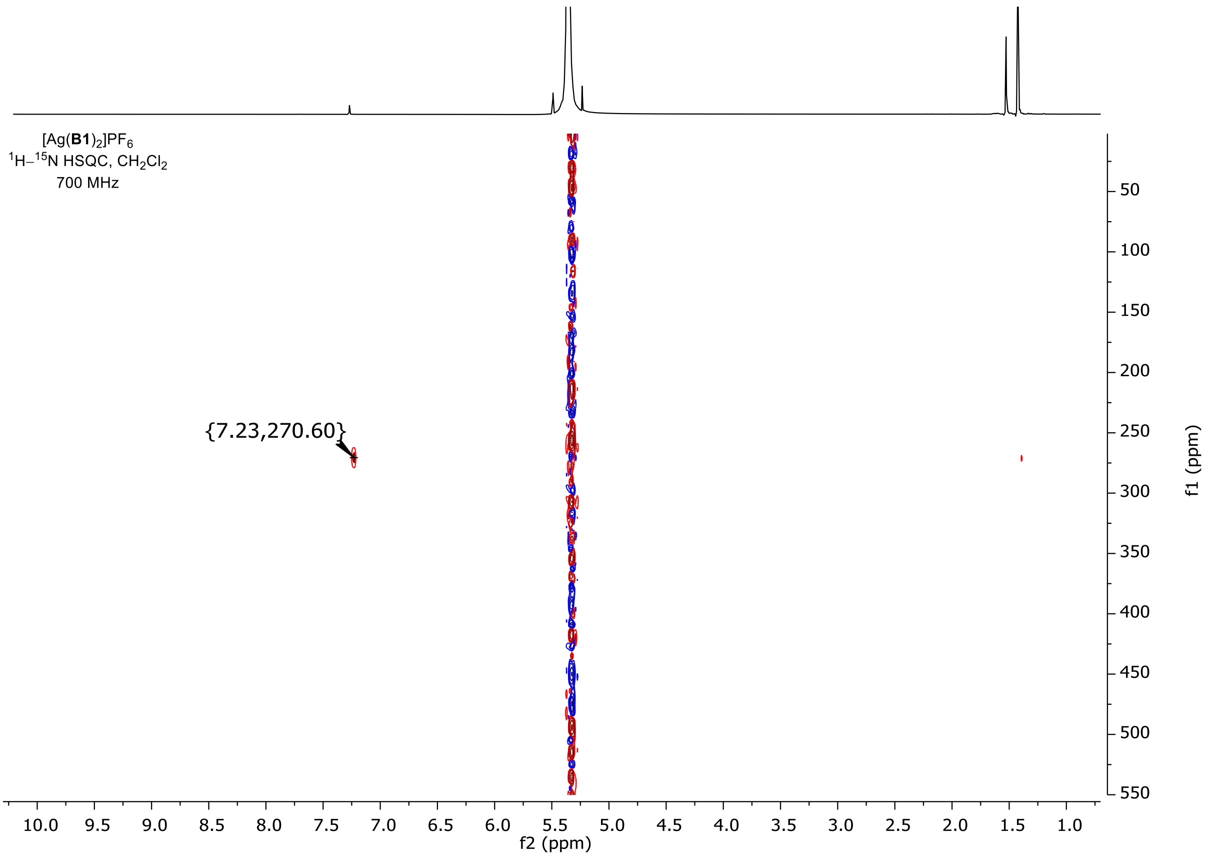

Figure 233. ^{1}H-^{15}N-HSQC (700 MHz, CH_2Cl_2) of a solution of 2,4,6-tri-*tert*-butylpyrimidine **B1** and $AgPF_6$ (2:1).

6. References

1. Biermann, U. & Metzger, J. O. Alkylation of alkenes: ethylaluminum sesquichloride-mediated hydro-alkyl additions with alkyl chloroformates and di-tert-butylpyrocarbonate. *J. Am. Chem. Soc.* **126**, 10319–10330 (2004).
2. Zhang, F. *et al.* Cu-catalyzed cascades to carbocycles: union of diaryliodonium salts with alkenes or alkynes exploiting remote carbocations. *J. Am. Chem. Soc.* **136**, 8851–8854 (2014).
3. Mathur, N. C., Snow, M. S., Young, K. M. & Pincock, J. A. Substituent effects on the rate of carbene formation by the pyrolysis of rigid aryl substituted diazomethanes. *Tetrahedron* **41**, 1509–1516 (1985).
4. Dai, Y., Feng, X. & Du, H. B(C6F5)3-Catalyzed Highly Stereoselective Hydrogenation of Unfunctionalized Tetrasubstituted Olefins. *Org. Lett.* **21**, 6884–6887 (2019).
5. Shimomaki, K., Murata, K., Martin, R. & Iwasawa, N. Visible-Light-Driven Carboxylation of Aryl Halides by the Combined Use of Palladium and Photoredox Catalysts. *J. Am. Chem. Soc.* **139**, 9467–9470 (2017).
6. Schevenels, F. T., Shen, M. & Snyder, S. A. Alkyldisulfanium Salts: Isolable, Electrophilic Sulfur Reagents Competent for Polyene Cyclizations. *Org. Lett.* **19**, 2–5 (2017).
7. Satyanarayana, G. & Maier, M. E. Tricyclic isoindolines by Heck cyclization. *Tetrahedron* **68**, 1745–1749 (2012).
8. Bahou, K. A. *et al.* A Relay Strategy Actuates Pre-Existing Trisubstituted Olefins in Monoterpenoids for Cross-Metathesis with Trisubstituted Alkenes. *J. Org. Chem.* **85**, 4906–4917 (2020).
9. Czaplik, W. M., Mayer, M. & Jacobi von Wangelin, A. Domino iron catalysis: direct aryl-alkyl cross-coupling. *Angew. Chem. Int. Ed.* **48**, 607–610 (2009).
10. Shi, X. *et al.* Asymmetric total synthesis of (+)-isocryptotanshinone and formal synthesis of (−)-cryptotanshinone. *Tetrahedron* **75**, 3962–3967 (2019).
11. Kobatake, M., Miyoshi, N. & Ueno, M. One-Pot Tandem Coupling Method for the Short-Step Formal Synthesis of Riccardin C. *Chem. Eur. J.* **29**, e202203805 (2023).
12. Huang, H.-M. *et al.* Three-Component, Interrupted Radical Heck/Allylic Substitution Cascade Involving Unactivated Alkyl Bromides. *J. Am. Chem. Soc.* **142**, 10173–10183 (2020).

13. Denmark, S. E. & Jaunet, A. Catalytic, enantioselective, intramolecular carbosulfenylation of olefins. Preparative and stereochemical aspects. *J. Org. Chem.* **79**, 140–171 (2014).

14. Hatakeyama, T., Nakagawa, N. & Nakamura, M. Iron-catalyzed Negishi coupling toward an effective olefin synthesis. *Org. Lett.* **11**, 4496–4499 (2009).

15. Bouquillon, S., Ganchegui, B., Estrine, B., Hénin, F. & Muzart, J. Heck arylation of allylic alcohols in molten salts. *J. Organomet. Chem.* **634**, 153–156 (2001).

16. Wylie, P. L., Prowse, K. S. & Belill, M. A. Synthesis by SO2 extrusion: photochemical and thermal reactions of cinnamyl benzyl sulfone. *J. Org. Chem.* **48**, 4022–4025 (1983).

17. Chen, X., Cheng, Z., Guo, J. & Lu, Z. Asymmetric remote C-H borylation of internal alkenes via alkene isomerization. *Nat. Commun.* **9**, 3939 (2018).

18. Taber, D. F., Paquette, C. M., Gu, P. & Tian, W. Cyclohexanones by Rh-mediated intramolecular C-H insertion. *J. Org. Chem.* **78**, 9772–9780 (2013).

19. Nomura, S., Endo-Umeda, K., Makishima, M., Hashimoto, Y. & Ishikawa, M. Development of Tetrachlorophthalimides as Liver X Receptor β (LXRβ)-Selective Agonists. *ChemMedChem* **11**, 2347–2360 (2016).

20. Jones, D. H., Smith, K., Elliott, M. C. & El-Hiti, G. A. Factors affecting reactions of trialkylcyanoborates with imidoyl chlorides/trifluoroacetic anhydride. *Tetrahedron* **71**, 6285–6289 (2015).

21. Engler, T. A. & Wanner, J. Lewis acid-directed cyclocondensation of piperidone enol ethers with 2-methoxy-4-(N-phenylsulfonyl)-1,4-benzoquinoneimine: a new regioselective synthesis of oxygenated carbolines. *J. Org. Chem.* **65**, 2444–2457 (2000).

22. Lee, T. & Jones, J. B. Probing the Abilities of Synthetically Useful Serine Proteases To Discriminate between the Configurations of Remote Stereocenters Using Chiral Aldehyde Inhibitors. *J. Am. Chem. Soc.* **118**, 502–508 (1996).

23. Nagasawa, S., Sasano, Y. & Iwabuchi, Y. Catalytic Oxygenative Allylic Transposition of Alkenes into Enones with an Azaadamantane-Type Oxoammonium Salt Catalyst. *Chem. Eur. J.* **23**, 10276–10279 (2017).

24. McCalmont, W. F. *et al.* Investigation into the structure-activity relationship of novel concentration dependent, dual action T-type calcium channel agonists/antagonists. *Bioorg. Med. Chem.* **13**, 3821–3839 (2005).

25. Datta, S., Chang, C.-L., Yeh, K.-L. & Liu, R.-S. A new ruthenium-catalyzed cleavage of a carbon-carbon triple bond: efficient transformation of ethynyl alcohol into alkene and carbon monoxide. *J. Am. Chem. Soc.* **125**, 9294–9295 (2003).

26. Molander, G. A. & Sandrock, D. L. Utilization of potassium vinyltrifluoroborate in the development of a 1,2-dianion equivalent. *Org. Lett.* **11**, 2369–2372 (2009).

27. Johnston, B. D., Czyzewska, E. & Oehlschlager, A. C. [2 + 2] Cycloaddition of dichloroketene to allyl ethers and thioethers. *J. Org. Chem.* **52**, 3693–3697 (1987).

28. Klaper, M., Fudickar, W. & Linker, T. Role of Distance in Singlet Oxygen Applications: A Model System. *J. Am. Chem. Soc.* **138**, 7024–7029 (2016).

29. Schmidt, J. & Stark, C. B. W. Synthetic endeavors toward 2-nitro-4-alkylpyrroles in the context of the total synthesis of heronapyrrole C and preparation of a carboxylate natural product analogue. *J. Org. Chem.* **79**, 1920–1928 (2014).

30. Wei, S., Mao, Y. & Shi, S.-L. Nickel-Catalyzed Ligand-Free Hiyama Coupling of Aryl Bromides and Vinyltrimethoxysilane. *Synlett* **32**, 1670–1674 (2021).

31. Trippé, G. *et al.* Crown-tetrathiafulvalenes attached to a pyrrole or an EDOT unit: synthesis, electropolymerization and recognition properties. *Chem. Eur. J.* **10**, 6497–6509 (2004).

32. García-Rubia, A., Urones, B., Gómez Arrayás, R. & Carretero, J. C. Pd(II)-catalysed C-H functionalisation of indoles and pyrroles assisted by the removable N-(2-pyridyl)sulfonyl group: C2-alkenylation and dehydrogenative homocoupling. *Chem. Eur. J.* **16**, 9676–9685 (2010).

33. Bock, J., Daniliuc, C. G. & Hennecke, U. Stable Bromiranium Ion Salts as Reagents for Biomimetic Indole Terpenoid Cyclizations. *Org. Lett.* **21**, 1704–1707 (2019).

34. Westermaier, M. & Mayr, H. Electrophilic allylations and benzylations of indoles in neutral aqueous or alcoholic solutions. *Org. Lett.* **8**, 4791–4794 (2006).

35. Duan, Y., Lin, J.-H., Xiao, J.-C. & Gu, Y.-C. A Trifluoromethylcarbene Source. *Org. Lett.* **18**, 2471–2474 (2016).

36. Shallu, M. L. S. & Singh, J. First total synthesis of a guanidine alkaloid Nitensidine D using immobilized ionic liquid, microwaves and formamidinesulfinic acid. *J. Chem. Sci.* **126**, 1869–1874 (2014).

37. Hanessian, S., Cooke, N. G., DeHoff, B. & Sakito, Y. The total synthesis of (+)-ionomycin. *J. Am. Chem. Soc.* **112**, 5276–5290 (1990).

38. Albitz, K., Csókás, D., Dobi, Z., Pápai, I. & Soós, T. Late-Stage Formal Double C-H Oxidation of Prenylated Molecules to Alkylidene Oxetanes and Azetidines by Strain-Enabled Cross-Metathesis. *Angew. Chem. Int. Ed.* **62**, e202216879 (2023).

39. Larock, R. C., Leung, W.-Y. & Stolz-Dunn, S. Synthesis of aryl-substituted aldehydes and ketones via palladium-catalyzed coupling of aryl halides and non-allylic unsaturated alcohols. *Tetrahedron Lett.* **30**, 6629–6632 (1989).
40. Meka, B., Ravada, S. R., Muthyala, M. K. K., Kurre, P. N. & Golakoti, T. Synthesis, in vitro and in silico evaluation of diaryl heptanones as potential 5LOX enzyme inhibitors. *Bioorg. Chem.* **80**, 408–421 (2018).
41. Mei, T.-S., Werner, E. W., Burckle, A. J. & Sigman, M. S. Enantioselective redox-relay oxidative heck arylations of acyclic alkenyl alcohols using boronic acids. *J. Am. Chem. Soc.* **135**, 6830–6833 (2013).
42. Urgaonkar, S. & Verkade, J. G. Ligand-, copper-, and amine-free sonogashira reaction of aryl iodides and bromides with terminal alkynes. *J. Org. Chem.* **69**, 5752–5755 (2004).
43. Czyzyk, D. J. *et al.* Structure activity relationship towards design of cryptosporidium specific thymidylate synthase inhibitors. *Eur. J. Med. Chem.* **183**, 111673 (2019).
44. Yamada, T., Watanabe, Y. & Okamoto, S. 6-Halo-2-pyridone as an efficient organocatalyst for ester aminolysis. *RSC Adv.* **11**, 24588–24593 (2021).
45. Satoh, T., Unno, H., Mizu, Y. & Hayashi, Y. Ligand exchange reaction of sulfoxides in organic synthesis: A versatile procedure for one-carbon homologation of methylesters to esters, thioesters, carboxylic acids and amides. *Tetrahedron* **53**, 7843–7854 (1997).
46. Elings, J. A., Downing, R. S. & Sheldon, R. A. Cyclialkylation of Arylalkyl Epoxides with Solid Acid Catalysts. *Eur. J. Org. Chem.* **1999**, 837–846 (1999).
47. An, S., Zhang, Z. & Li, P. Metal-Free Synthesis of Selenodihydronaphthalenes by Selenoxide-Mediated Electrophilic Cyclization of Alkynes. *Eur. J. Org. Chem.* **2021**, 3059–3070 (2021).
48. Ling, H.-B. *et al.* Gold-Catalyzed Oxidation Terminal Alkyne: An Approach to Synthesize Substituted Dihydronaphthalen-2(1H)-ones and Phenanthrenols. *J. Org. Chem.* **82**, 7070–7076 (2017).
49. Kuang, J. & Ma, S. An efficient synthesis of terminal allenes from terminal 1-alkynes. *J. Org. Chem.* **74**, 1763–1765 (2009).
50. Kawade, R. K., Huang, P.-H., Karad, S. N. & Liu, R.-S. Gold-catalyzed annulations of allenes with N-hydroxyanilines to form indole derivatives with benzaldehyde as a promoter. *Org. Biomol. Chem.* **12**, 737–740 (2014).

51. Bigi, M. A. & White, M. C. Terminal olefins to linear α,β-unsaturated ketones: Pd(II)/hypervalent iodine co-catalyzed Wacker oxidation-dehydrogenation. *J. Am. Chem. Soc.* **135**, 7831–7834 (2013).

52. Satoh, T., Kondo, A. & Musashi, J. Generation of magnesium carbenoids from 1-chloroalkyl phenyl sulfoxides with a Grignard reagent and applications to alkylation and olefin synthesis. *Tetrahedron* **60**, 5453–5460 (2004).

53. Okamura, T. *et al.* Synthetic Access to gem-Difluoropropargyl Vinyl Ethers and Their Application to Propargyl Claisen Rearrangement. *J. Org. Chem.* **86**, 1911–1924 (2021).

54. Cherney, E. C., Green, J. C. & Baran, P. S. Synthesis of ent-kaurane and beyerane diterpenoids by controlled fragmentations of overbred intermediates. *Angew. Chem. Int. Ed.* **52**, 9019–9022 (2013).

55. Wang, X.-Z., Wu, Y.-L., Jiang, S. & Singh, G. Synthesis of (2S,3R,4E,8E)-9-methyl-4,8-sphingadienine via a novel SN2′ type reaction mediated by a thioether carbanion. *Tetrahedron Lett.* **40**, 8911–8914 (1999).

56. Nagumo, S. *et al.* Intramolecular Friedel–Crafts type reaction of vinyloxiranes linked to an ester group. *Tetrahedron* **65**, 9884–9896 (2009).

57. Browder, C. C., Marmsäter, F. P. & West, F. G. Highly efficient trapping of the Nazarov intermediate with substituted arenes. *Org. Lett.* **3**, 3033–3035 (2001).

58. Gao, Q. *Novel Lewis Acid-promoted cyclization reactions and synthesis of triptolide analogs. (Thesis).* (The University of Hong Kong, Pokfulam, Hong Kong SAR., 2003).

59. Breit, B. & Breuninger, D. Desymmetrizing hydroformylation with the aid of a planar chiral catalyst-directing group. *J. Am. Chem. Soc.* **126**, 10244–10245 (2004).

60. Dai, M. & Danishefsky, S. J. The total synthesis of spirotenuipesines A and B. *J. Am. Chem. Soc.* **129**, 3498–3499 (2007).

61. Dickmeiss, G. *et al.* Organocatalytic asymmetric desymmetrization-fragmentation of cyclic ketones. *Angew. Chem. Int. Ed.* **48**, 6650–6653 (2009).

62. Wolter, M., Nordmann, G., Job, G. E. & Buchwald, S. L. Copper-catalyzed coupling of aryl iodides with aliphatic alcohols. *Org. Lett.* **4**, 973–976 (2002).

63. Bartoszewicz, A., Kalek, M. & Stawinski, J. Iodine-promoted silylation of alcohols with silyl chlorides. Synthetic and mechanistic studies. *Tetrahedron* **64**, 8843–8850 (2008).

64. Trost, B. M., Malhotra, S. & Chan, W. H. Exercising regiocontrol in palladium-catalyzed asymmetric prenylations and geranylation: Unifying strategy toward flustramines A and B. *J. Am. Chem. Soc.* **133**, 7328–7331 (2011).

65. Xiao, L., Pöthig, A. & Hintermann, L. 2-Amino-1,3,5-triazine chemistry: Hydrogen-bond networks, Takemoto thiourea catalyst analogs, and olfactory mapping of a sweet-smelling triazine. *Monatsh. Chem.* **146**, 1529–1539 (2015).

66. Stowers, K. J., Fortner, K. C. & Sanford, M. S. Aerobic Pd-catalyzed sp3 C-H olefination: A route to both N-heterocyclic scaffolds and alkenes. *J. Am. Chem. Soc.* **133**, 6541–6544 (2011).

67. Sato, T. & Tamura, K. Metal-catalyzed organic photoreactions. Photoreaction of olefins with 2-chloroacetophenone in the presence of silver trifluoromethanesulfonate. *Tetrahedron Lett.* **25**, 1821–1824 (1984).

68. Zuidema, D. R. *et al.* Deoxygenation of Aromatic Ketones Using Transfer Hydrogenolysis with Raney Nickel in 2-Propanol. *Synth. Commun.* **41**, 2927–2931 (2011).

69. Lai, S. & Lee, D. G. Lewis acid assisted permanganate oxidations. *Tetrahedron* **58**, 9879–9887 (2002).

70. Pracht, P., Bohle, F. & Grimme, S. Automated exploration of the low-energy chemical space with fast quantum chemical methods. *Phys. Chem. Chem. Phys.* **22**, 7169–7192 (2020).

71. Bannwarth, C., Ehlert, S. & Grimme, S. GFN2-xTB-An Accurate and Broadly Parametrized Self-Consistent Tight-Binding Quantum Chemical Method with Multipole Electrostatics and Density-Dependent Dispersion Contributions. *J. Chem. Theory Comput.* **15**, 1652–1671 (2019).

72. Ehlert, S., Stahn, M., Spicher, S. & Grimme, S. Robust and Efficient Implicit Solvation Model for Fast Semiempirical Methods. *J. Chem. Theory Comput.* **17**, 4250–4261 (2021).

73. Chai, J.-D. & Head-Gordon, M. Long-range corrected hybrid density functionals with damped atom-atom dispersion corrections. *Phys. Chem. Chem. Phys.* **10**, 6615–6620 (2008).

74. Weigend, F. Accurate Coulomb-fitting basis sets for H to Rn. *Phys. Chem. Chem. Phys.* **8**, 1057–1065 (2006).

75. Weigend, F. & Ahlrichs, R. Balanced basis sets of split valence, triple zeta valence and quadruple zeta valence quality for H to Rn: Design and assessment of accuracy. *Phys. Chem. Chem. Phys.* **7**, 3297–3305 (2005).

76. Gaussian 16, Revision C.01, Frisch, M. J.; Trucks, G. W.; Schlegel, H. B.; Scuseria, G. E.; Robb, M. A.; Cheeseman, J. R.; Scalmani, G.; Barone, V.; Petersson, G. A.; Nakatsuji, H.; Li, X.; Caricato, M.; Marenich, A. V.; Bloino, J.; Janesko, B. G.; Gomperts, R.;

Mennucci, B.; Hratchian, H. P.; Ortiz, J. V.; Izmaylov, A. F.; Sonnenberg, J. L.; Williams-Young, D.; Ding, F.; Lipparini, F.; Egidi, F.; Goings, J.; Peng, B.; Petrone, A.; Henderson, T.; Ranasinghe, D.; Zakrzewski, V. G.; Gao, J.; Rega, N.; Zheng, G.; Liang, W.; Hada, M.; Ehara, M.; Toyota, K.; Fukuda, R.; Hasegawa, J.; Ishida, M.; Nakajima, T.; Honda, Y.; Kitao, O.; Nakai, H.; Vreven, T.; Throssell, K.; Montgomery, J. A., Jr.; Peralta, J. E.; Ogliaro, F.; Bearpark, M. J.; Heyd, J. J.; Brothers, E. N.; Kudin, K. N.; Staroverov, V. N.; Keith, T. A.; Kobayashi, R.; Normand, J.; Raghavachari, K.; Rendell, A. P.; Burant, J. C.; Iyengar, S. S.; Tomasi, J.; Cossi, M.; Millam, J. M.; Klene, M.; Adamo, C.; Cammi, R.; Ochterski, J. W.; Martin, R. L.; Morokuma, K.; Farkas, O.; Foresman, J. B.; Fox, D. J. Gaussian, Inc., Wallingford CT, 2016.

77. Miertuš, S., Scrocco, E. & Tomasi, J. Electrostatic interaction of a solute with a continuum. A direct utilizaion of AB initio molecular potentials for the prevision of solvent effects. *Chem. Phys.* **55**, 117–129 (1981).

78. Miertuš, S. & Tomasi, J. Approximate evaluations of the electrostatic free energy and internal energy changes in solution processes. *Chem. Phys.* **65**, 239–245 (1982).

79. Pascual-ahuir, J. L., Silla, E. & Tuñon, I. GEPOL: An improved description of molecular surfaces. III. A new algorithm for the computation of a solvent-excluding surface. *J. Comput. Chem.* **15**, 1127–1138 (1994).

80. Legault, C.: CylView. http://www.cylview.org.

81. Seeman, J. I. Effect of conformational change on reactivity in organic chemistry. Evaluations, applications, and extensions of Curtin-Hammett Winstein-Holness kinetics. *Chem. Rev.* **83**, 83–134 (1983).

82. Curtin, D. Y. Sterochemical Control of Organic Reactions Differences in Behavior of Diastereomers. *Rec. Chem. Prog.* **15**, 111–128 (1954).
